# Redescription of three fossil baleen whale skulls from the Miocene of Portugal reveals new cetotheriid phylogenetic insights

**DOI:** 10.1371/journal.pone.0298658

**Published:** 2024-03-13

**Authors:** Rodrigo Figueiredo, Mark Bosselaers, Liliana Póvoas, Rui Castanhinha

**Affiliations:** 1 Center for Environmental and Marine Studies (CESAM) & Biology Department, University of Aveiro, Aveiro, Portugal; 2 Directorate of Earth and History of Life, Royal Belgian Institute of Natural Sciences (RBINS), Brussels, Belgium; 3 Royal Zeeland Scientific Society, Middelburg, The Netherlands; 4 Museu Nacional de História Natural e da Ciência (MUHNAC), University of Lisbon (UL), Lisbon, Portugal; 5 Grupo de Etnologia e Arquelogia da Lourinhã (GEAL), Museu da Lourinhã, Lourinhã, Portugal; University of Szeged Institute of Biology: Szegedi Tudomanyegyetem Biologia Intezet, HUNGARY

## Abstract

Cetotheriidae is a family of baleen whales that went nearly extinct during the Pleistocene (excluding Caperea marginata). For a long time, the Cetotheriidae family has been seen as a problematic clade, but in the past two decades there have been various studies trying to resolve the phylogeny of this group. In 1831, Alexandre Vandelli described three cetotheriid skulls, found during a gold exploration at Adiça beach (Portugal). These specimens constituted the first Portuguese vertebrate fossils ever published in the literature. Another skull was added to the “Vandelli skulls” by Jacinto Pedro Gomes, in 1914, during a survey of the Museu Nacional de História Natural collections without giving information on the origin of this skull. In 1941, Remington Kellogg states that one of the original “Vandelli skulls” is no longer present in the Museu Nacional de História Natural collections. Until today, there is no information on how, or exactly when, the fourth skull and one of the original three “Vandelli skulls” appeared and disappeared, respectively. Since their discovery, all the attempts to describe these specimens were not based on direct observations and no comprehensive phylogenetic analysis have included the three skulls. Here we provide a detailed anatomic description, a new phylogenetic analysis and a palaeoecological reconstruction of these specimens, clarifying their relationships within the Cetotheriidae family and fostering the importance of these historical specimens to the modern comprehension of fossil whale evolution. In addition, our results support that Cephalotropis nectus is a valid species with an emended diagnosis. We also concluded that two specimens belong to a new genus, forming two new fossil species (new combinations).

## Introduction

Since the first description of Cetotheriidae [1], a lot of new studies, methodologies and tools have been published. We now have more accessibility to information, high-definition images, CT-scans and softwares that allow us to perform accurate relationship studies between the existing species. Therefore, there is an urgent need to revise the specimens that were described a long time ago, either to verify their taxonomy or to update their anatomical features. It is also relevant, to study specimens that were collected in locations that are no longer accessible.

Cetotheriidae was first described in 1872 by Brandt [1] based on its type genus *Cetotherium* (*Cetotherium rathkii* as type species) [2,3]. This clade included several extinct taxa, namely multiple Chaeomysticetes that could not be affiliated with other Mysticeti families. A great number of species that were assigned as Cetotheriidae were described from isolated fragments of skulls or postcranial elements [4]. In more recent years the position on Cetotheriidae has been revised and clarified [5–20]. The Cetotheriidae is currently divided in two major groups, the species closely related to *Cetotherium rathkii* (Cetotheriidae *sensu stricto*) and the species whose relationships are still dubious (“Cetotheres” *sensu lato*) [10,21].

**Fig 1:**
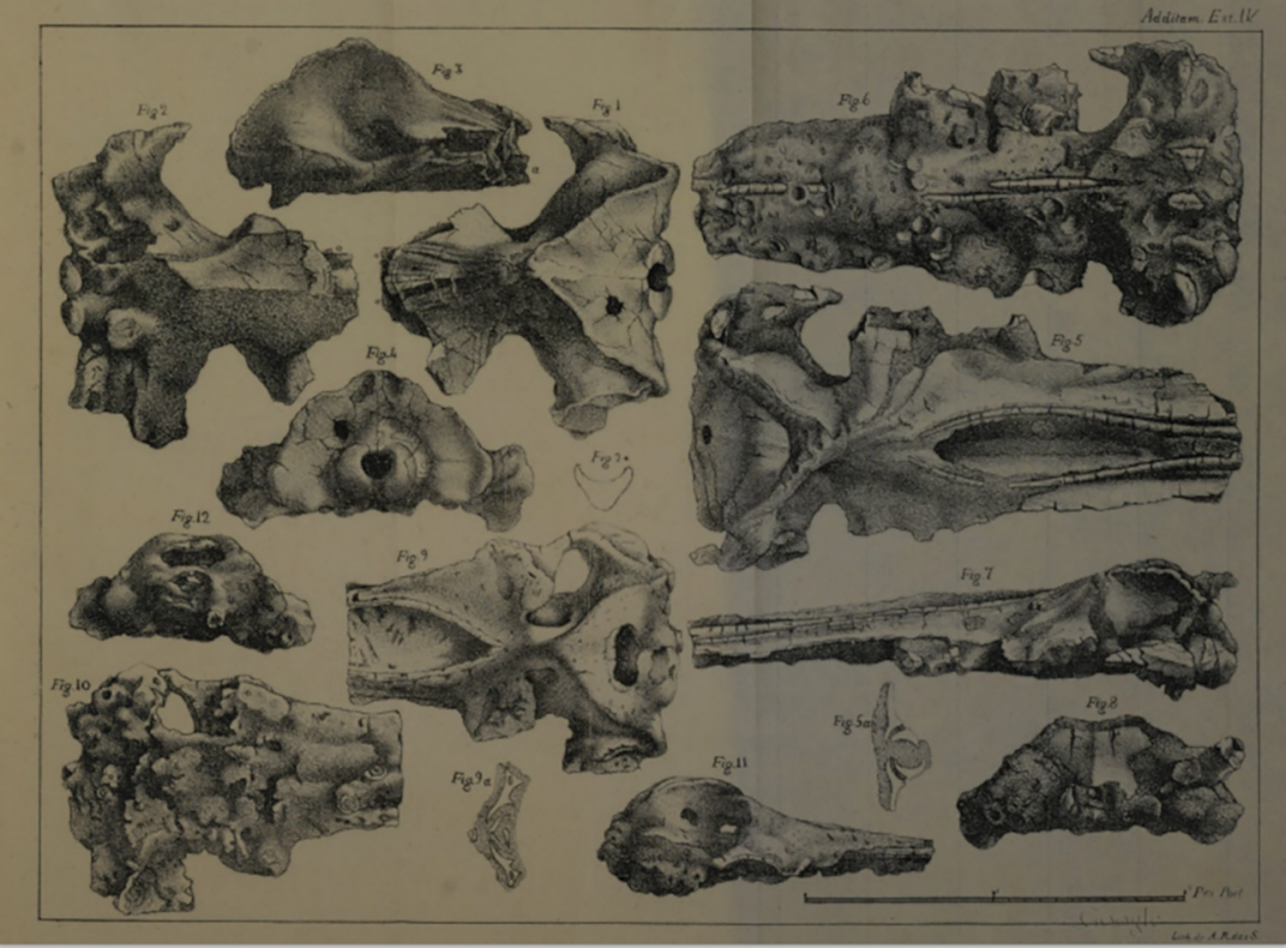
Plate IV from Vandelli, 1831. *Figs 1–4* MNHN/UL.C0 (Missing skull); *Fig 5–8* MNHN/UL.C1; *Fig 9–12* MNHN/UL.C2.

The first reference to a vertebrate fossil in the Portuguese literature, was made by Wilhelm Ludwig von Eschwege in 1831 [22], and at Adiça beach, Costa de Caparica, Almada, Portugal. However, the author did not describe nor attribute a specimen number. Eschwege was the head of the mining inspectorate of Portugal at that time [23] charged Alexandre António Vandelli to review and publish his notes. In 1831, Vandelli reports two other fossil whale skulls from the same site [24] Fig 1) [24] and highlights the good preservation of the skulls although they were damaged during the excavation, transport, and preparation (*p*. *292* from [24]). He classifies one of the specimens as *Balenae* (Fig 1, Plate IV from [24]) and the others as *Physeter* (Fig 1, Plate IV *Fig 5–12*) (*p*. *296* from [24]), making the “Vandelli skulls” the first Portuguese vertebrate fossils to be described in the literature [25].

**Fig 2:**
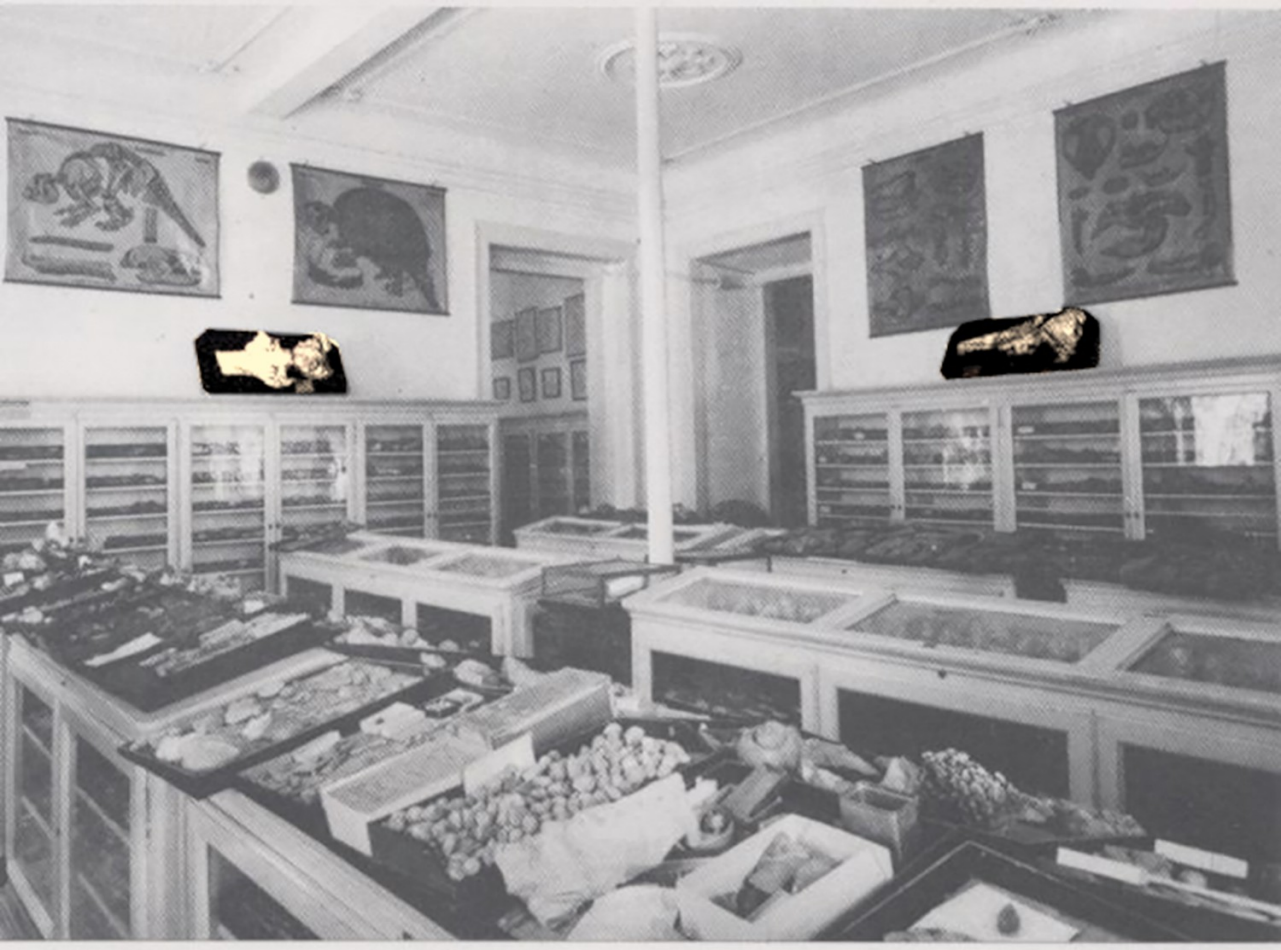
**Highlighted MNHN/UL.C3 and MNHN/UL.C1 left and right respectively, exhibited on the old Portuguese stratigraphy section of the Museu Nacional de História Natural (Photo by Abreu Nunes, 1958; Photo in the MUHNAC archives).** Reprinted from [27] under a CC BY license, with permission from MUHNAC, original copyright [1958]).

In 1914, Jacinto Pedro Gomes reports for the first time the presence of four skulls of fossil whales at the Museu Nacional de História Natural e da Ciência (MUHNAC), previously known as Museu Nacional de História Natural (MNHN), during a survey on the collections of paleontology and geology [26]. Gomes refers the skulls as *Balaenoptera* and points out that those four skulls were collected in the same location. This is the only record regarding the origin of this fourth skull (MNHN/UL.C3; Fig 2).

**Fig 3:**
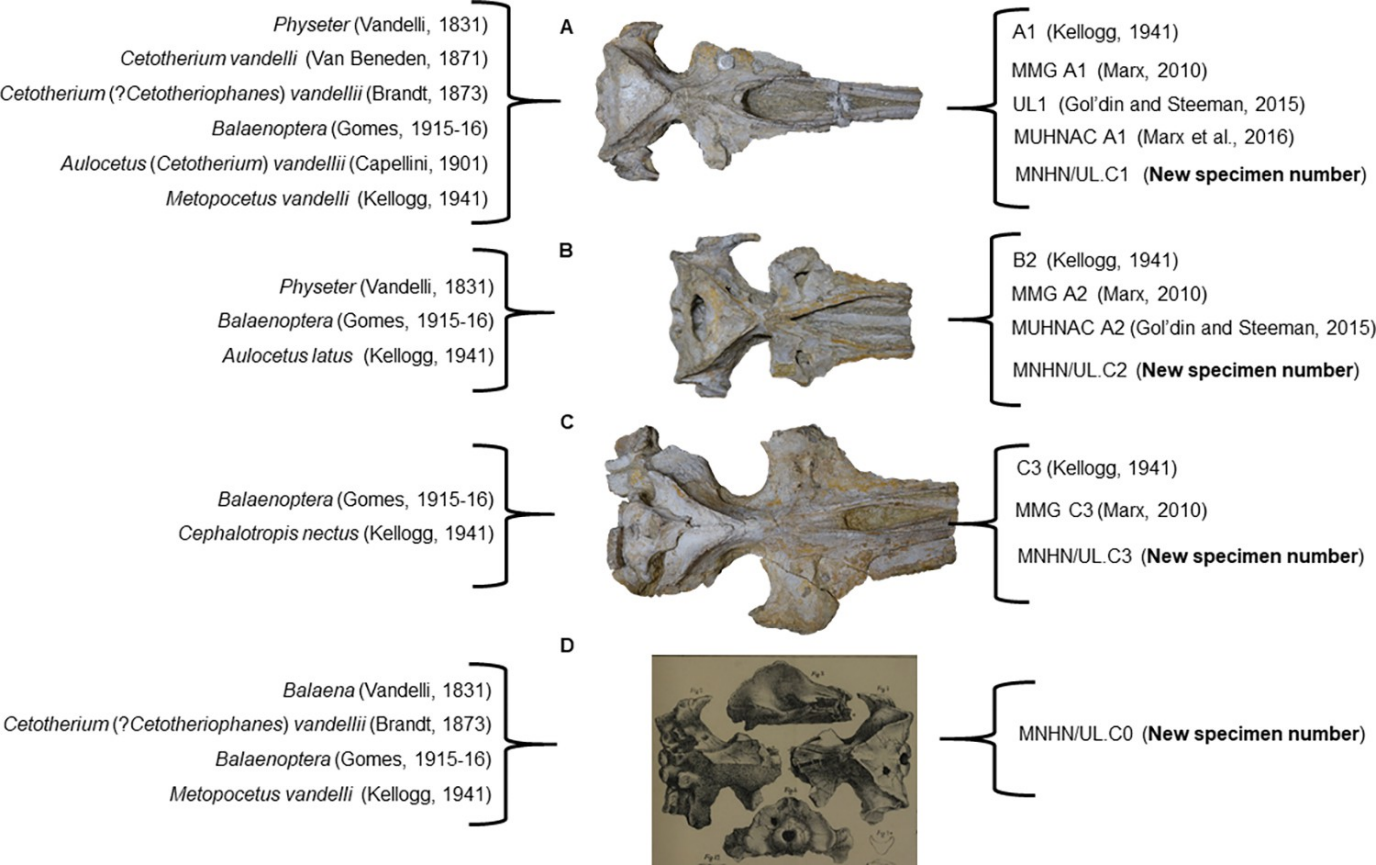
References of each specimen, MNHN/UL.C1 (A), MNHN/UL.C2 (B), MNHN/UL.C3 (C) and MNHN/UL.C0 (D), made by various authors. (MNHN/UL.C0 image adapted from [24]).

The “Vandelli skulls” have been named by several authors over the years (Fig 3). After Vandelli, in 1871, Van Beneden and Gervais publish an illustration and brief general description of MNHN/UL.C1 reporting it as a *Cetotherium vandelli* skull [28]. Two years later, Brandt adds some characteristics to the description of Van Beneden and Gervais, distinguishes it from the already existing species of *Cetotherium*, illustrates MNHN/UL.C0 and MNHN/UL.C1, and names the two skulls as "*Cetotherium* (?*Cetotheriophanes*) *vandellii"* (*sic*) [29]. However, Brandt proposed *Cetotheriophanes* as a "subgenus" of *Cetotherium*. In 1901, Capellini refers to the specimen MNHN/UL.C1 as *Aulocetus* (*Cetotherium*) *vandelii* and briefly compares some of its anatomical features with *Aulocetus lovisati* [30] with no further comments on this specimen.

The first detailed description of the “Vandelli skulls” was made by Kellogg [31]. In this study, Kellogg states that one of the skulls (specimen MNHN/UL.C0) was no longer present in the collections of the Museu Nacional de História Natural e da Ciência. We can confirm that MNHN/UL.C0 is currently still missing, and we could not collect any information or record about when or how this skull disappeared. Kellogg names two new species, the specimen MNHN/UL.C2 as the holotype of *Aulocetus latus* and the specimen MNHN/UL.C3 as the holotype of *Cephalotropis nectus* and proposes a new genus to "*Cetotherium" vandellii* (specimens MNHN/UL.C1 and MNHN/UL.C0), creating a new combination: *Metopocetus vandelli* [31]. The classification attributed by Kellogg is the most commonly accepted in the most recent literature. However, in 2010, Mocho and Póvoas found similarities between the specimen MNHN/UL.C3 and *Cephalotropis coronatus*, stating that some of the characteristics and measurements applied in the study of 1941 are not accurate since Kellogg only had access to pictures [32]. It is important to note that only Vandelli in 1831 and Mocho and Póvoas in 2010, had direct access to the specimens, all other authors based their classifications on illustrations, pictures or indirect information.

Several authors suggested the urgent need for a new and detailed redescription of the specimens [5,6,12,13,16,33]. Bisconti stated that all species belonging to *Aulocetus* should be redescribed, because the genus *Aulocetus* became a junior synonym of *Cetotheriopsis* since these two genera have the same type species (*Balaenodon lintianus* Von Meyer, 1849) [33]. Bouetel and Muizon [5] point out some features regarding the specimens MNHN/UL.C1 and MNHN/UL.C2 and state that they are probably part of the Cetotheriidae *s*.*s*. but need a taxonomic revision to corroborate this hypothesis. Whitmore and Barnes [6] disagree with the attribution of the genus *Metopocetus* to MNHN/UL.C1 and reclassify this specimen as *Cetotherium*. Gol’din and Startsev [13] agree that the MNHN/UL.C1 is not *Metopocetus* but do not concur with an attribution to *Cetotherium*. El Adli et al. [12] affirmed that the specimen MNHN/UL.C1 identified as *Metopocetus vandelli* may be incorrectly diagnosed and possibly belongs to a new genus. A study by Marx et al. [16] describes a new species of *Metopocetus* and lists several characteristics that distinguish MNHN/UL.C1 from the other *Metopocetus* species, suggesting that the specimen does not belong in fact to the *Metopocetus* genus. The specimens MNHN/UL.C1 and MNHN/UL.C2 often cluster together with “*Cetotherium” megalophysum* in several phylogenetic analyses from different publications [12,15,16,19] and recent studies proposed that they may represent the same genus or even the same species [16].

The work here presented seeks to solve the taxonomical problems concerning the "Vandelli skulls" found in Adiça by doing a detailed anatomical description of the specimens with direct observations and by running an updated phylogenetic analysis. We have also included a description of the paleohabitat by identifying the fossil invertebrates associated with each specimen.

## Material and methods

### Specimens

All specimens here redescribed, namely MNHN/UL.C1, MNHN/UL.C2 and MNHN/UL.C3, are housed, and accessible, at the paleontological collections of the MUNHAC, Universidade de Lisboa, Portugal.

The three specimens were collected by Alexandre Vandelli [26] from a fossil cliff on Adiça beach, during a gold exploration that occurred between 1814 and 1826 or 1836 [23,34].

The specimens show signs of mechanical preparation, namely, there are grooves probably made by a small chisel or air-scribes. Various types of glue were used to glue/fix some fractures. Some fragments are glued with plaster, paraloid or a mix of other unidentified substances.

To estimate the total body length, we used the stem balaenopteroid equation of Pyenson and Sponberg [35]. The measurements were obtained using a measuring tape (+-1mm) and firm joint calipers of different sizes.

The specimen numbers assigned to the specimens here studied were reformulated according to the general collection code of the palaeontology collections of the Museu Nacional de História Natural e da Ciência. As such, as is represented in Fig 3, specimen A is here referred as MNHN/UL.C1, specimen B as MNHN/UL.C2, specimen C as MNHN/UL.C3, and the missing skull, specimen D as MNHN/UL.C0.

One interesting detail is that the tag glued to the right frontal of MNHN/UL.C2, that identifies the specimen according to Vandelli (Plate IV from [24]), belongs to the missing specimen and not to MNHN/UL.C2.

No permits were required for the described study, which complied with all relevant regulations.

### Phylogenetic analysis and anatomical description

For the phylogenetic analysis, we used the same methodology and total evidence matrix from Duboys *et al*. [36] (updated version of the matrix from Marx and Fordyce (2015) [14]). The coding of MNHN/UL.C1 and MNHN/UL.C2 were partially amended and *Cephalotropis nectus* (MNHN/UL.C3) was added to the total evidence matrix. The analysis was performed using MrBayes 3.2.7 [37] on the Cyberinfrastructure for Phylogenetic Research Science Gateway (CIPRES) [38]. To the morphological data a maximum-likelihood model was assigned [39] and a gamma parameter (Mk + Γ), and the coding bias was set as “informative”. As in Duboys *et al*. [36], the analysis was set to run for 20 million generations, three separate runs with four chains each, sampling every 1000 generations and discarding the first 25% as burn-in. The trees obtained were summarized using SumTrees [40]. The synapomorphies of the consensus tree were accessed using Mesquite [41].

The photographs and the phylogenetic trees were edited using the software Gimp [42] and FigTree [43], respectively.

The anatomical terminology used in the description generally follows Mead and Fordyce (2009), and in some cases, anatomical terms from Marx, Bosselaers and Louwye (2016) (Posteroventral flange) [44].

We constructed a portfolio with illustrations of all codable characters and respective character states (201 of 278 characters). This portfolio clearly shows our interpretation of each character (see S1 File).

### Nomenclature acts

The electronic edition of this article conforms to the requirements of the amended International Code of Zoological Nomenclature, and hence the new names contained herein are available under that Code from the electronic edition of this article. This published work and the nomenclatural acts it contains have been registered in ZooBank, the online registration system for the ICZN. The ZooBank LSIDs (Life Science Identifiers) can be resolved and the associated information viewed through any standard web browser by appending the LSID to the prefix "http://zoobank.org/". The LSID for this publication is: urn:lsid:zoobank.org:pub:5DE53250-D899-472D-BB79-67AB9DD4BDE4. The electronic edition of this work was published in a journal with an ISSN, and has been archived and is available from the following digital repositories: PubMed Central, LOCKSS.

## Results

### *1. Adicetus* (New genus)

#### (a) Systematic paleontology

**Cetacea** Brisson, 1762 [45]

**Neoceti** Fordyce and de Muizon, 2001 [46]

**Mysticeti** Gray, 1864 [47]

**Chaeomysticeti** Mitchell, 1989 [48]

**Cetotheriidae** Brandt, 1872 [1]

***Adicetus***, **gen. nov.** *urn*:*lsid*:*zoobank*.*org*:*act*:*E592BBBD-BD6C-4114-901F-EC97ED27A2A0*

### Type species

*Adicetus vandelli*, as represented by MNHN/UL.C1, that comprises a nearly complete skull with braincase, periotic bones, and almost complete rostrum.

### Included species

*Adicetus latus*, new combination

*Adicetus vandelli*, new combination

### Diagnosis

Small to medium-sized cetotheriid sharing with the other members of the family an elongated and convergent ascending process of the maxillae, nasals with posteriorly convergent edges and an enlarged compound posterior process of the tympanoperiotic with its facial sulcus floored by a posteroventral flange. Shares with all cetotheriids except *Cephalotropis* a wider than long temporal fossa; with all cetotheriids except *Cephalotropis* and *Tiucetus* the presence of a primary dorsal infraorbital foramen on the ascending processes of the maxillae; with all cetotheriids except *Herpetocetus* and *Nannocetus* the presence of a ventrally oriented postglenoid process, in posterior view; with all cetotheriids except *Herpetocetus*, *Nannocetus*, *Mithridatocetus* and *Ciuciulea* a well-developed conical process of the tympanic bulla; with all cetotheriids except *Herpetocetus*, *Nannocetus*, *Kurdalagonus* and *Brandtocetus* a facial sulcus running along the posterior margin of the compound posterior process. Further differs from all cetotheriids by having a pair of faint tubercles on the supraoccipital; from all cetotheriids except *Piscobalaena* by having a sigmoidal shaped suture between the maxillae and palatines; from *Cephalotropis*, *Tiucetus*, *Joumocetus* and *Cetotherium* by having a concave lateral profile of the facial region of the rostrum; from *Cephalotropis*, *Tiucetus*, *Joumocetus*, *Ciuciulea*, *Mithridatocetus* and *Metopocetus* by having roughly parallel-sided ascending processes of the maxillae; from *Cephalotropis*, *Tiucetus*, *Joumocetus* and *Ciuciulea* for not having the parietal exposed on the skull vertex; from *Tiucetus*, *Tranatocetus*, *Cetotherium*, *Brandtocetus* and *Kurdalagonus* for lacking a squamosal prominence; from *Herentalia*, *Kurdalagonus*, *Mithridatocetus*, *Piscobalaena*, *Cetotherium*, *Ciuciulea* and *Brandtocetus* by lacking a well-defined external occipital crest; and from *Tranatocetus*, *Metopocetus*, *Herpetocetus*, *Nannocetus* and *Piscobalaena* for having a cylindrical shaped compound posterior process.

### Etymology

*Adicetus* derives from the words “Adiça”, the name of the beach where these specimens were discovered, and “Cetus”, latinized from the ancient Greek “κῆτος”, which means sea monster or any other large marine creature.

### *2*. *Adicetus latus*

#### (a) Systematic palaeontology

***Adicetus latus***, **new combination** *urn*:*lsid*:*zoobank*.*org*:*act*:*26E3B665-EE9C-4DDA-BDD1-16DE0036BF03*

### Holotype

MNHN/UL.C2, that comprises a nearly complete skull with braincase, tympanoperiotic bones and the posterior portion of the rostrum.

### Diagnosis

Shares with *Adicetus vandelli* a: elongate and roughly parallel-sided ascending processes of the maxillae; faint pair of tubercles on the supraoccipital; bent supraoccipital with its anterodorsal portion transversely concave; pointed supraoccipital; sigmoidal suture between the palatines and the maxillae; concave lateral profile of the anterior portion of the rostrum, in lateral view; and a wide narial fossa. Differs from *Adicetus vandelli* in having: the compound posterior process firmly integrated in the lateral wall of the skull; a proportionally bigger squamosal even though *Adicetus latus* is smaller; a significantly less laterally projected nuchal crest; a more excavated squamosal fossa with an acute posterior angle; a less rounded palatal keel; and it has the paired tubercles in the occipital region placed more laterally and ventrally. In addition, it also differs from *Adicetus vandelli* in lacking a groove between the ascending process of the maxilla and the frontal and a well-developed crest in the anterolateral portion of the maxilla.

### Locality and horizon

The specimen here described was collected in 1831 by Vandelli in a gold mine located at Adiça beach, between Fonte da Telha and Lagoa da Albufeira, in Costa de Caparica, Almada, Portugal (38° 33’ 9’‘ N, 9° 11’ 12’‘ W. [25]). The skull was retrieved from the Miocene, lower Tortonian [49], Lisbon Miocene Cotter’s division VIIb (ca. 10 Ma) [50]. According to Vandelli [24], the skull was found in thick dark green limestone, full of invertebrate shells.

### (b) Anatomical Description

***Adicetus latus* (*Aulocetus latus*, *sensu* Kellogg 1941 [31]; *Balenoptera* sp, Gomes 1915–16 [26]; *Physeter* sp, Vandelli 1831 [24])**

#### Overview

**Fig 4:**
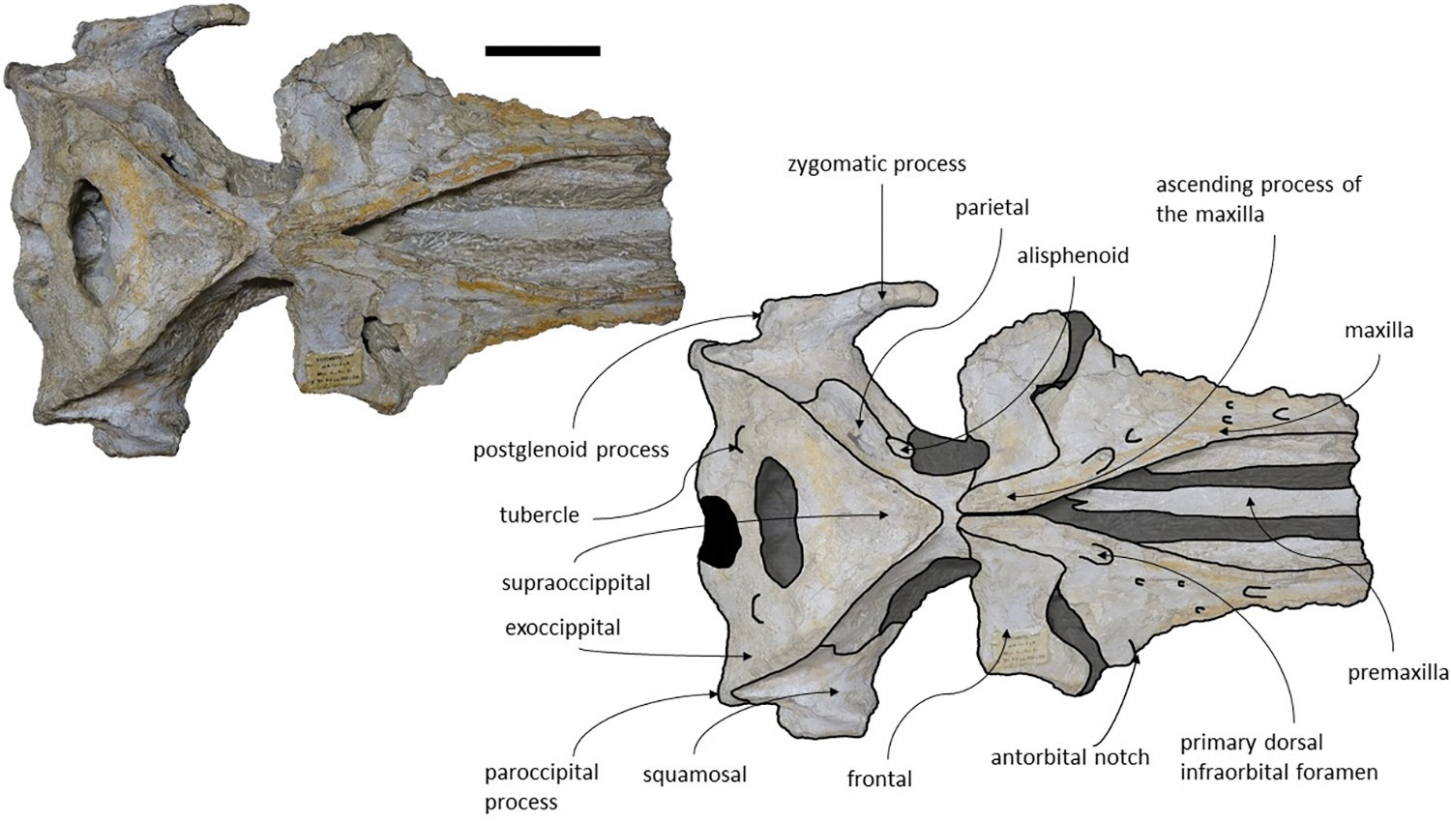
*Adicetus latus* (MNHN/UL.C2) in dorsal view. Scale– 100 mm.

**Fig 5:**
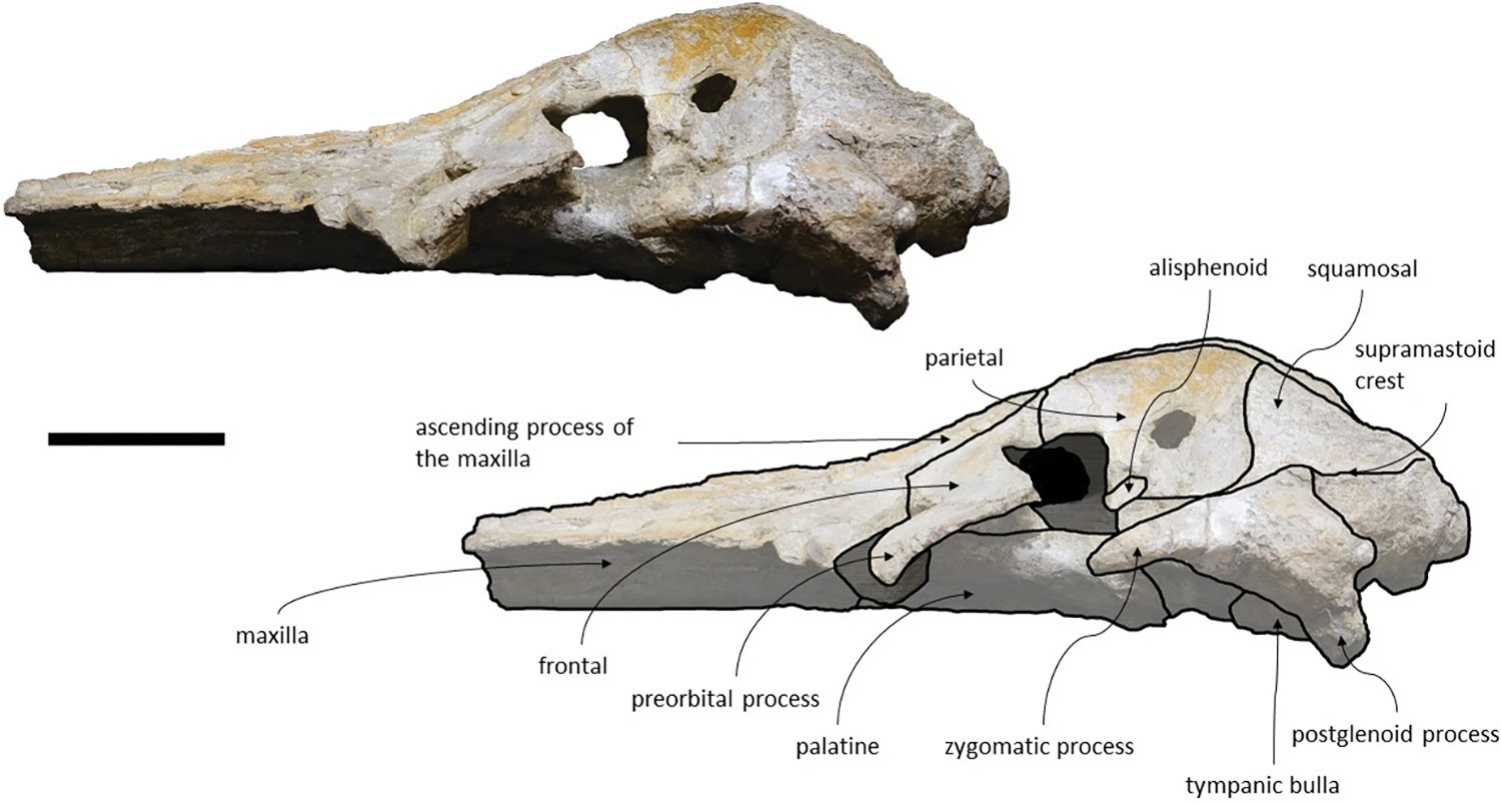
*Adicetus latus* (MNHN/UL.C2) in left lateral view. Scale– 100 mm.

**Fig 6:**
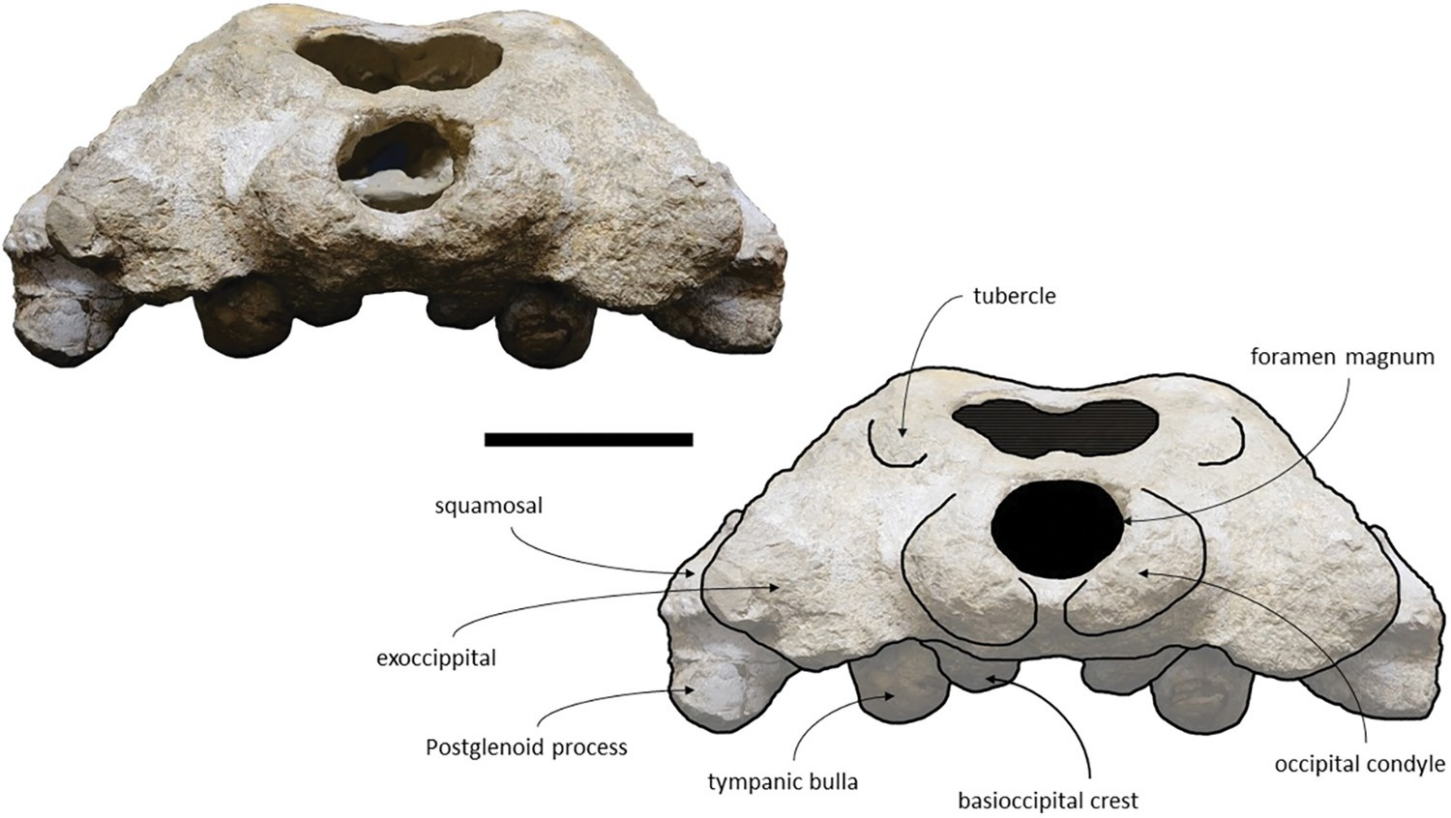
*Adicetus latus* (MNHN/UL.C2) in posterior view. Scale– 100 mm.

The *Adicetus latus* skull is almost complete, however the anterior portion of the rostrum, nasals and lateral margins of the skull are missing (Fig 4). The non-preserved rostrum would probably correspond to the anterior half and was broken leaving a coronal fracture exposing a cross section of the rostrum approximately at midpoint. The right zygomatic process is missing, and the lateral margins of the maxillae and frontals are damaged. The right premaxilla is missing, and the posterior half of the left premaxilla is medially displaced. In lateral view, the temporal wall is damaged where the frontal, parietal and alisphenoid would contact (Fig 5). This damage forms a wide opening connecting both sides of the skull. The supraoccipital bears an opening above the foramen magnum (Fig 6). There are no mandibular nor post-cranial elements associated. The measurements of the specimen are represented in Table 1.

**Table 1:** Measurements of *Adicetus latus* (MNHN/UL.C2) (in mm), e—estimated measurements.

	MNHN/UL.C2
Bizygomatic width	396 (e)
Width across exoccipitals	314
Bicondylar width	135
Width of the base of ascending processes of the maxillae	81
Length of the compound posterior process (*in situ*; from the posterior tip to the posterior pedicle)	70
Length of tympanic bulla	79
Width of the tympanic bulla at sigmoidal process	51
Width of the tympanic bulla anterior to sigmoidal process	38
Width of the tympanic bulla posterior to sigmoidal process	49
Length of the neurocranium	360
Width between antorbital notches	260
Anteroposterior length of parietal exposure on skull vertex	18
Distance between lateral margins of basioccipital crests	97
Greatest height of neurocranium	360
Diameter of primary infraorbital foramen	9
Diameter of the foramen pseudovale	18
Total body length based on bizygomatic width (estimation using the balaenopteroid equation of Pyenson and Sponberg [35])	4280 (e)

**Fig 7:**
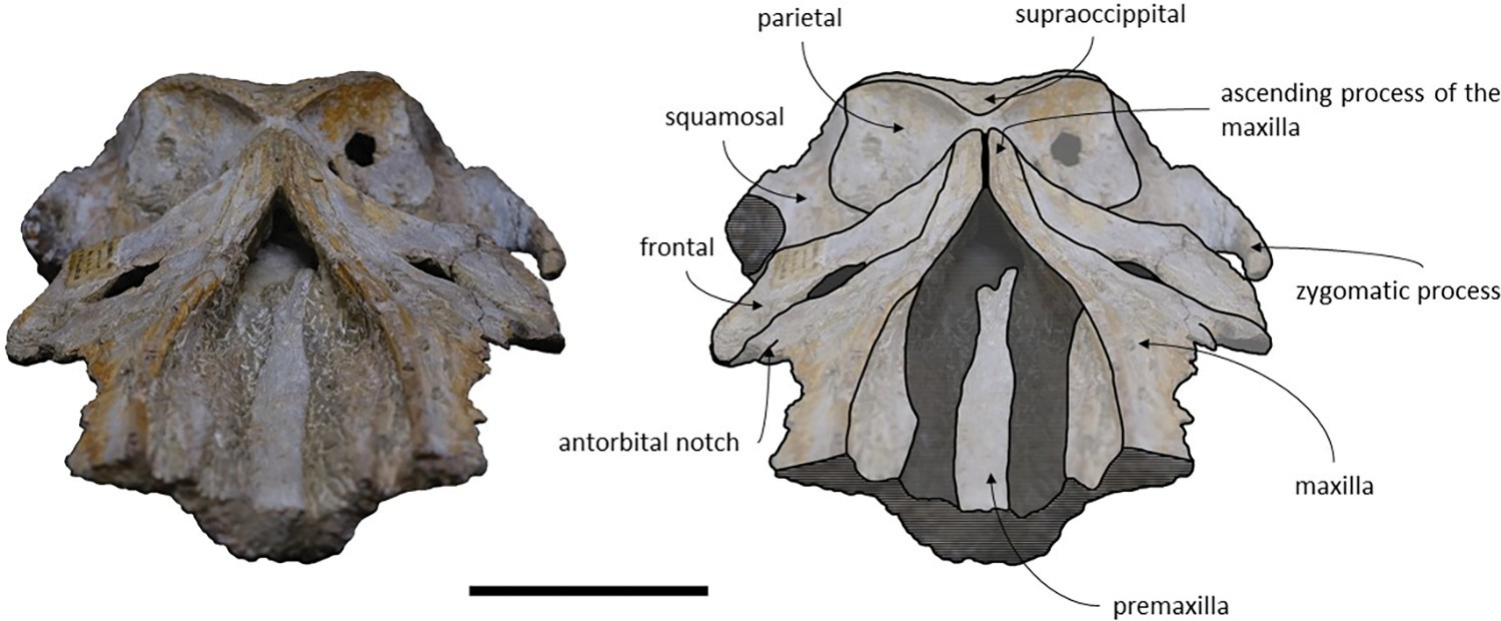
*Adicetus latus* (MNHN/UL.C2) in anterior view.

**Fig 8:**
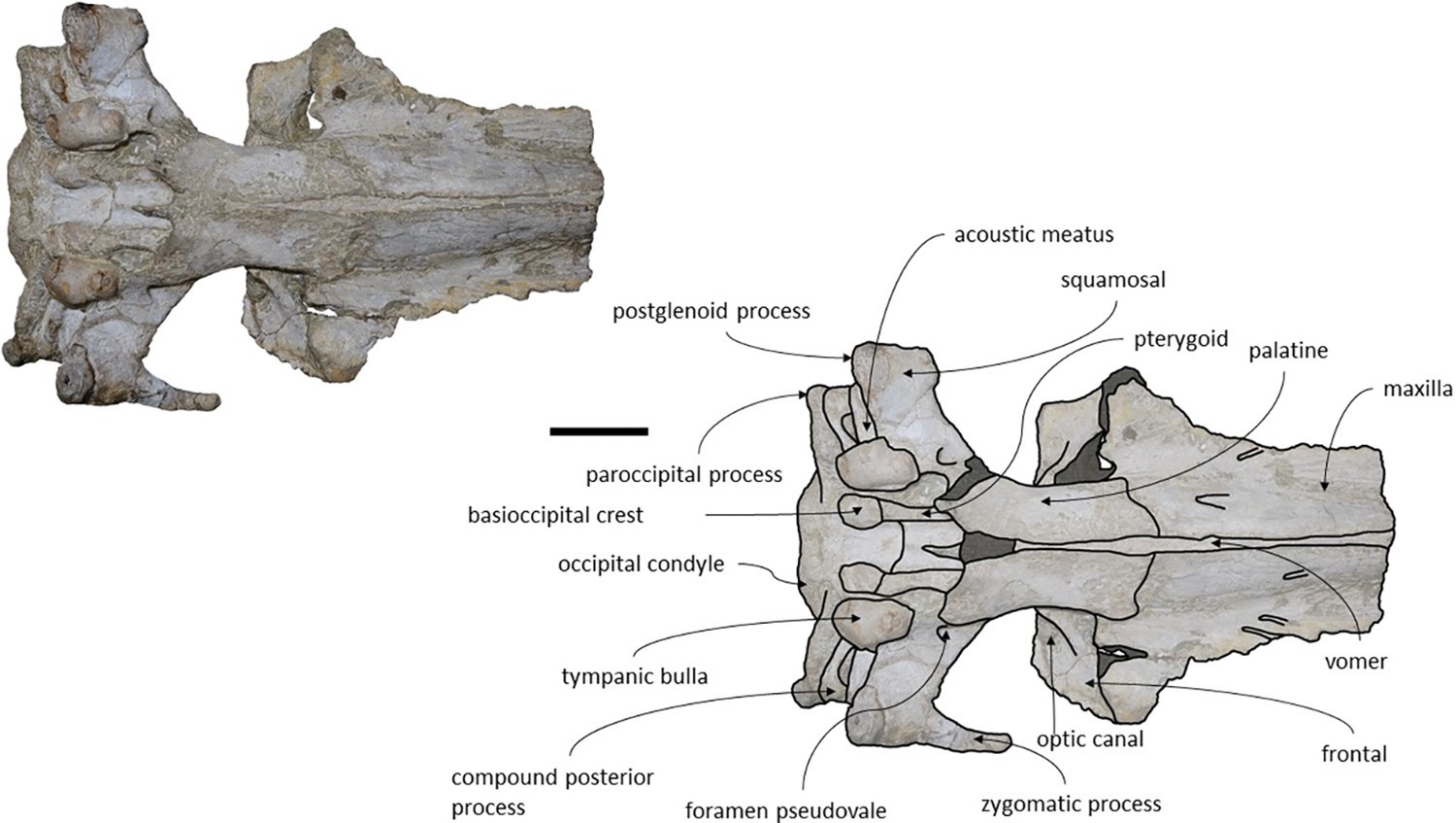
*Adicetus latus* (MNHN/UL.C2) in ventral view. Scale– 100 mm.

*Maxilla*. Although the anterior portion of the maxillae is missing, and the lateral edges are eroded, it is possible to infer that the maxilla, if complete, would be longer than the bizygomatic width, in dorsal view. The maxillae contact the frontals and parietals posteriorly, the premaxillae and vomer dorsomedially and the palatines ventrally. The suture between the maxillae and palatines is sigmoidal. The maximum width between the medial margins of the maxillae (narial fossa) surpass the maximum width of the ascending processes of the maxillae. In cross section, the maxillae are sub-triangular with a flattened dorsal surface (Fig 7). The premaxillae are not *in situ*, as such, the maxillae-premaxillae suture facets are dorsomedially exposed. Ventrally, the maxillae and vomer form a round palatal keel. In ventral view, there is a groove in the ventral surface of the maxillae posterolateral portions, immediately posterior to the antorbital notch. The ascending processes of the maxillae contact medially at the posterior half, for approximately a third of the total length of the ascending processes of the maxillae. The medial edges of the ascending process of the maxillae are approximately parallel with each other, similar to *Herentalia*, *Piscobalaena*, *Cetotherium* and *Tranatocetus* [5,20,51,52]. There is a primary infraorbital foramen near the base of the ascending process of the maxilla. The maxilla-frontal suture presents a slight overriding of the frontal medially while the maxillae underlie the frontal laterally. The lacrimals are not preserved, however, there is a gap between the lateral portions of the frontal and maxilla suggesting that the lacrimals would be lateral to the ascending process of the maxilla, and that the lateral processes of the maxillae would underlap the lacrimals. Although the lateral processes of the maxillae are damaged, the antorbital notches are preserved. In dorsal view, there are ten foramina in the maxillae. In ventral view, there are five foramina and multiple well excavated sulci, some of which start at the ventral foramina (Fig 8).

*Premaxilla*. The premaxilla is elongated, flat, a nearly sheet-like bone. Only the left premaxilla is preserved, but it is displaced medially between the maxillae and rests on the vomer, with the medial surface facing dorsally whereas the lateral surface faces ventrally. The anterior portion of the left premaxilla is missing. The premaxillae would contact the maxillae ventrally and probably the nasals posteriorly. There are no foramina visible. The ventral margin of the left premaxilla is medially twisted, anteriorly. The dorsal margin of the premaxilla is ticker and rounder than the ventral margin. There is a fragment on the posteromedial portion of the ascending process of the left maxilla that may correspond to a portion of the left premaxilla or nasal.

*Nasal*. The nasals are not preserved. However, there is a fragment on the posteromedial portion of the ascending process of the left maxilla that may correspond to a portion of the left nasal or premaxilla. It is possible to infer that the lateral edges of the nasals were posteriorly convergent due to the medial edges of the ascending processes of the maxillae. It is important to note that due to the absence of the nasals, any anatomical inference about these bones is merely tentative.

*Frontal*. Both frontals are nearly complete and present a subtrapezoidal shape, in dorsal view. The posterolateral portion of the supraorbital process of both frontals are damaged and the postorbital processes are not preserved. Both preorbitals processes are partially damaged. The frontals contact the maxillae anteriorly, the parietals posteriorly, and the palatines ventrally. The frontal is excluded from the skull vertex. There is an orbitotemporal crest near the posterior edge of the frontal and lateral to the ascending process of the maxilla. The preserved posterior edges of the supraorbital processes of the frontals are roughly straight. The anterolateral portion of the frontal overlaps the maxilla while the anteromedial portion of the frontal underlies the maxilla. The anterior border of the supraorbital process of the frontal would be bordered by the lacrimal laterally and by the maxilla medially. The optic canal runs adjacent to the posterior border of the supraorbital process of the frontal and opens ventrally.

*Alisphenoid*. Both alisphenoids are subcircular in lateral view, they are approximately 2,5 cm in diameter. The alisphenoids are slightly damaged and partially covered by sediment. The alisphenoid contacts the parietal dorsally and the squamosal ventrally. The alisphenoid is exposed on the temporal wall and does not contribute to the orbital fissure.

*Parietal*. The parietals are nearly complete. In anterior view, each parietal expands laterally forming a subtriangular-shape, being longer than high, as in *Tranatocetus maregermanicum* for example [20]. There is damage on the anteroventral portion of the parietals and at the temporal wall, where each parietal has an opening. The parietals contact the supraoccipital posteriorly, the squamosals ventrally, the alisphenoids anteroventrally, and the frontals and maxillae anteriorly. The suture between the parietal and squamosal is sigmoidal, similar to *Cetotherium*, *Herentalia*, *Herpetocetus*, *Piscobalaena*, *Tranatocetus* and *Tiucetus* [5,12,18,20,34,52]. The fronto-parietal suture is irregular. On the skull vertex, the parietals separate the posterior tips of the ascending processes of the maxillae.

**Fig 9:**
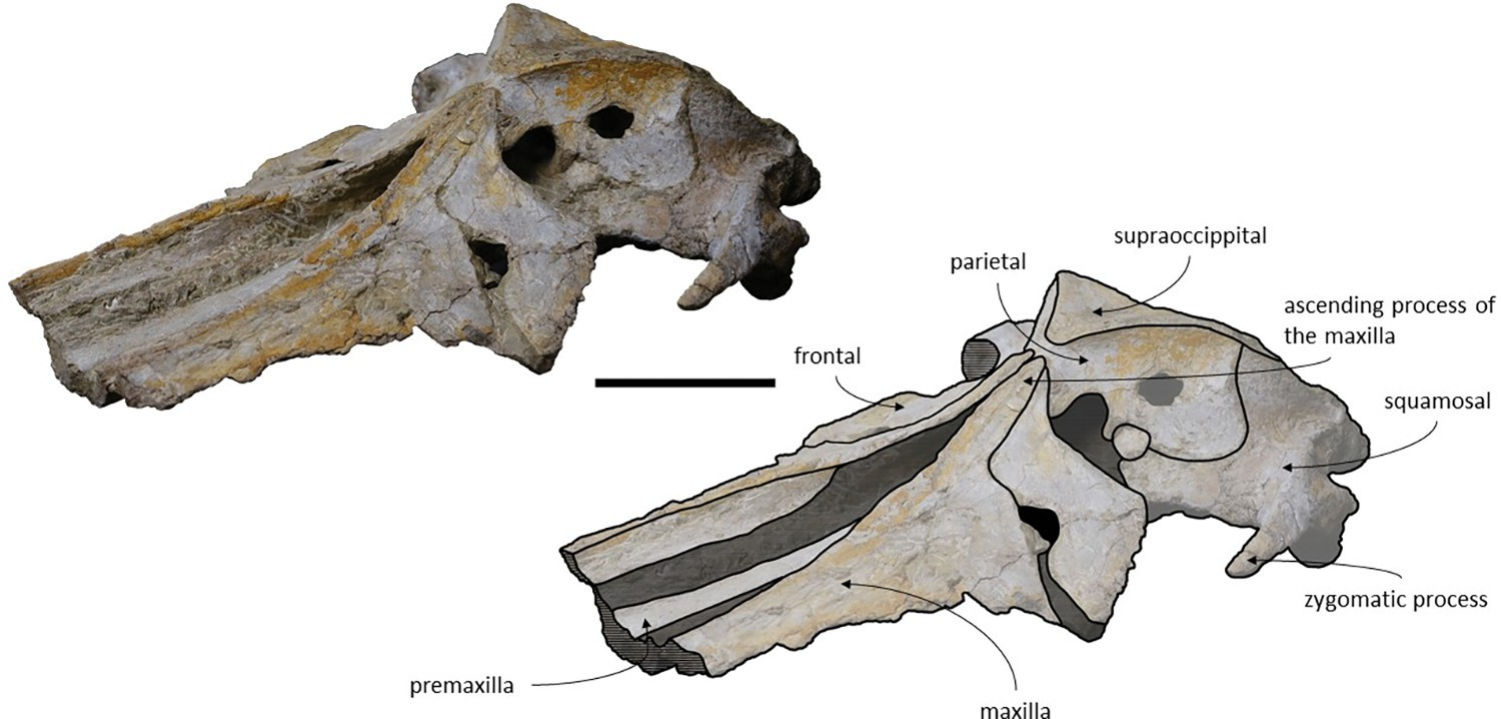
*Adicetus latus* (MNHN/UL.C2) in left anterolateral view.

*Squamosal*. Both squamosals are almost complete, however, on the right side the zygomatic process is missing. The squamosal is longer than high and contacts the exoccipitals posteriorly, the periotics ventromedially, pterygoids and alisphenoids anteriorly, and the parietals anterodorsally. The lateral border of the zygomatic process is not confluent with the exoccipitals, in posterior view. The zygomatic process is parallel to the sagittal plane, is anteroposteriorly thin and posteriorly expanded dorsoventrally (Fig 9), as in *Cetotherium*, *Herpetocetus*, *Piscobalaena* and *Tranatocetus* [5,12,20,34]. In lateral view, the zygomatic process apex deflects slightly anteroventrally, similar to *Herpetocetus morrowi* [12]. The supramastoid crest is only present in the posterior portion of the zygomatic process. In lateral view, the postglenoid process is posteriorly oriented and is ventral to the ventral edge of the exoccipital. In posterior view, the postglenoid process is ventrally oriented and presents a parabolic shape, these two traits also being present in, *Metopocetus hunteri*, Tranatocetus, *Tiucetus and Piscobalaena* [5,16,18,20]. The base of the postglenoid process is in transverse line with the middle of the tympanic bulla, in ventral view, and is in line with the lateral margin of the skull, in posterior view. The foramen pseudovale is located on the anteroventral edge of the squamosal and is almost exclusively formed by the squamosal, however, the pterygoid contributes to the anterior margin of the foramen pseudovale.

*Supraoccipital and exoccipital*. In the supraoccipital, there is an opening, dorsal to the foramen magnum. The supraoccipital contacts the exoccipitals posteriorly and the parietals anteroventrally. The exoccipitals contact the basioccipital posterolaterally, the squamosals posteriorly, the periotics ventrolaterally, and the supraoccipital anteriorly. In dorsal view, the supraoccipital has a triangular shape with a pointed apex, forming an angle of 70°. Anteriorly, the dorsal surface of the supraoccipital is transversely concave. The nuchal crests are slightly sigmoidal, with its anterior portion bowed inward and its posterior portion bowed outward, similar to *Tiucetus rosae* [18] rather than *Kurdalagonus mchedlidzei* [53], where the inverse is observed. There are two faint symmetrical tubercles, lateral to the opening of the supraoccipital. These tubercles are much less pronounced compared to those of *Archaebalaenoptera castriarquati* [54]. There is also a faint external occipital crest that is visible with a tangent light and noticeable by touch. The supraoccipital and exoccipitals are fused. The posterior apex of the nuchal crest is slightly anterior to the posteriormost point of the occipital condyles. The occipital condyles are confluent with the exoccipitals.

*Palatines*. The palatines have a trapezoidal shape, in ventral view. The palatines contact the pterygoids and squamosals posteriorly, the vomer dorsally, the frontals laterally and the maxillae anteriorly. The sutures between the palatines and maxillae are slightly sigmoidal. The anterior suture is roughly straight medially and bends posteriorly in the lateralmost portion, as in MNHN/UL.C1. The choanal margins of the palatines are concave. The anterior portion of the palatines are flattened and the posterior portion points posteroventrally. The medial edges of the palatines form a sharp keel. The anteriormost point of the palatines is posterior to the antorbital notch.

*Pterygoid*. Only the lateralmost portions of the ventral laminae of the pterygoids are preserved, although partially covered by sediment. The pterygoid hamuli are not preserved. The pterygoids contact the basioccipital and squamosals posteriorly, the palatines anteriorly, and the vomer dorsally. The ventral laminae of the pterygoid contact the anteromedial edge of the foramen pseudovale. The anterior most point of pterygoid sinus fossa is approximately in line with the anterior edge of foramen pseudovale.

**Fig 10:**
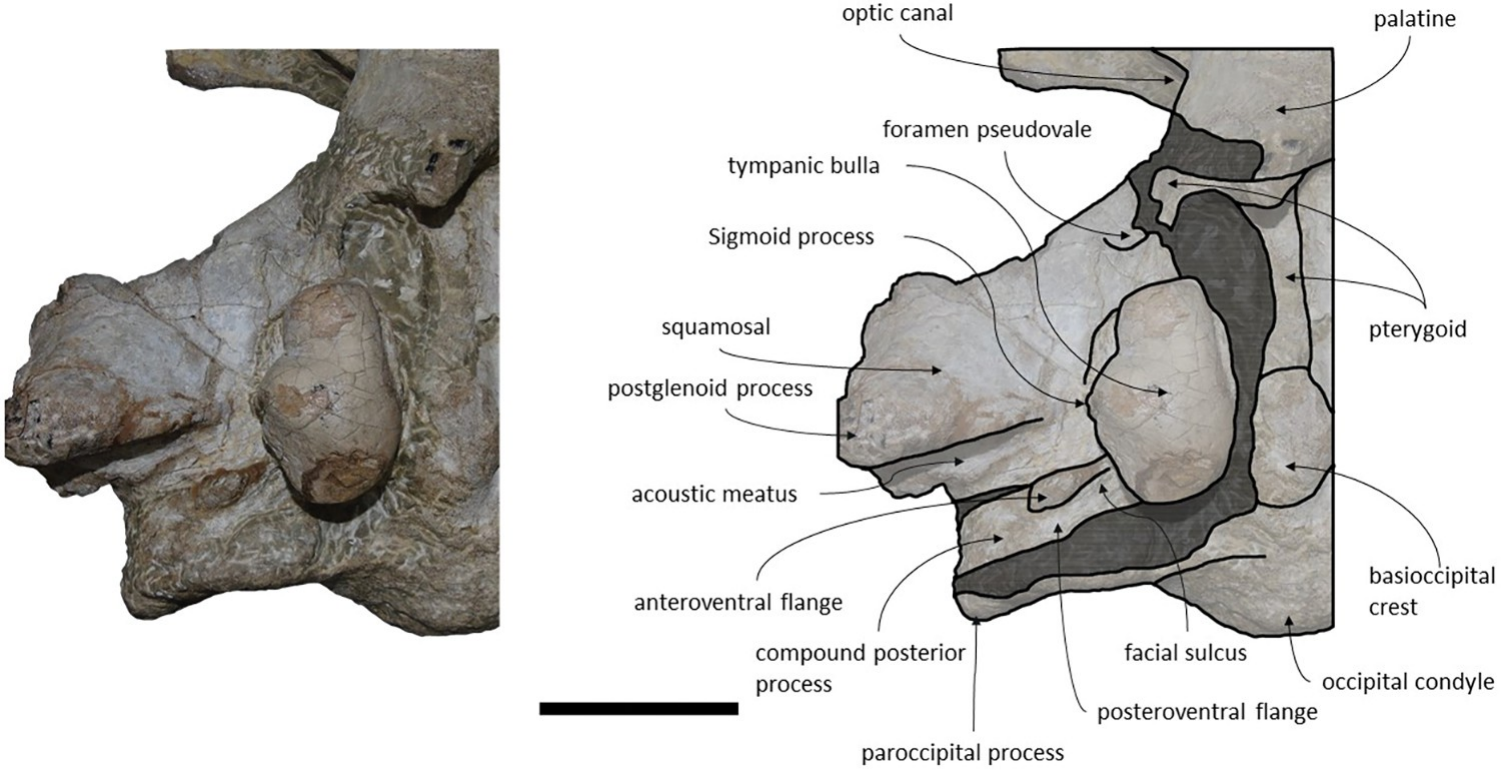
**Posterolateral portion (right side) of *Adicetus latus* (MNHN/UL.C2) cranium in ventral view.** Scale– 100 mm.

*Periotic and Tympanic Bulla*. The tympanic bullae remain *in situ* and are well preserved (Fig 10). The periotics contact the exoccipitals posteriorly and the squamosals laterally. The tympanic bullae contact the periotics dorsally. The posterior portion of the tympanic bulla is wider than its anterior portion. The compound posterior process has a cylindrical shape and is posterolaterally oriented relative to the sagittal axis, in ventral view. In lateral view, the posterior process is firmly integrated on the lateral skull wall, as in *Cetotherium*, *Kurdalagonus mchedlidzei*, *Brandtocetus chongulek* and *Caperea marginata* [11,13,34,53]. The fusion of the anterior and posterior pedicles of the tympanic bulla to the periotic is visible *in situ*. The posterior pedicle is located nearly adjacent to the posterior edge of the tympanic bulla. The anterior edge of the tympanic bulla is squared with the anterolateral corner inflated. The anteroposterior outline of the main ridge is straight anteriorly and convex posteriorly. In ventral view, the main axes of the tympanic bulla are roughly parallel to the longitudinal axis. The tympanic bulla presents a ventrally oriented lateral furrow, in lateral view, and an outer posterior prominence keel facing medially. The conical process is well pronounced and dorsally convex in lateral view. The medial width of the tympanic bulla is wider than its anterior and posterior width, in ventral view.

*Basioccipital*. Both basioccipital crests are partially eroded, however, it is possible to observe that the basioccipital crests are long and bulbous, with straight lateral borders. The basioccipital contacts the exoccipitals posteriorly and the pterygoids and vomer anteriorly. The basioccipital and the exoccipital are fused. The basioccipital/basisphenoid contact is not exposed.

### *1*. *Adicetus vandelli*

#### (a) Systematic palaeontology

***Adicetus vandelli*, new combination** *urn*:*lsid*:*zoobank*.*org*:*act*:*528FE137-AF9B-4D0D-BF59-94E7B1D75248*

### Diagnosis

Shares with *Adicetus latus* an elongate and roughly parallel-sided ascending processes of the maxillae; faint pair of tubercles on the supraoccipital; bent supraoccipital with its anterodorsal portion transversely concave; pointed supraoccipital; sigmoidal suture between the palatines and the maxillae; concave lateral profile of the anterior portion of the rostrum and a wide narial fossa. Differs from *Adicetus latus* in having: the compound posterior process integrated, but distinct, in the lateral wall of the skull; a proportionally smaller squamosal even though *Adicetus latus* is smaller; a well-defined laterally projected nuchal crest; a shallower squamosal fossa with a wider posterior angle; a rounder palatal keel; and more medially and dorsally positioned paired tubercles in the occipital region. In addition, it also differs from *Adicetus latus* in having a groove between the ascending process of the maxilla and the frontal and a well-developed crest in the anterolateral portion of the maxilla.

### Holotype

MNHN/UL.C1, partial skull comprising the braincase, periotic bones, and almost complete rostrum.

### Locality and horizon

The specimen here described was collected in 1831 by Vandelli in a gold mine located at Adiça beach, between Fonte da Telha and Lagoa da Albufeira, in Costa de Caparica, Almada, Portugal (38° 33’ 9’‘ N, 9° 11’ 12’‘ W. [25]). The skull was retrieved from the Miocene, lower Tortonian [49], Lisbon Miocene Cotter’s division VIIb (ca. 10 Ma) [50]. According to Vandelli [24], the skull was found in thick, dark green limestone, full of fossil invertebrate shells.

### (b) Anatomical description

***Adicetus vandelli* (*Metopocetus vandelli*, *sensu* Kellogg 1941 [31]; *Balenoptera* sp, Jacinto Gomes 1915–16 [26]; *Aulocetus* (*Cetotherium*) *vandelli*, *sensu* Capellini 1901 [30]; *Cetotherium vandellii*, *sensu* Brandt 1873 [55]; *Cetotherium vandelli*, *sensu* Van Beneden & Gervais 1871 [28]; *Physeter* sp, Vandelli 1831 [24])**.

### Overview

The specimen MNHN/UL.C1 consists of an almost complete and well-preserved skull with some missing parts, namely: the tip of the rostrum, the zygomatic processes of the squamosal and both tympanic bullae. All sutures are fused, which suggest that this specimen represents an adult individual. The lateral margins are damaged especially on rostral bones and the most anterior portion of the rostrum, as preserved, is fractured. This coronal fracture on the rostrum is not represented on the original illustration made by Vandelli in 1831 (Fig 2, Plate IV *Fig 5–8*) [24] nor in a later illustration made by Brandt in 1873 [55], suggesting that the fracture occurred after these publications and was probably caused by preparation or accident. The illustrations made in 1831 show that the lateral margins of the maxillae, the left frontal and left zygomatic process of the specimen were well preserved. The ventral surface of the skull shows signs of oxidation. There are no reports of mandibular or post-cranial elements associated. The measurements of the specimen are represented in Table 2.

**Table 2:** Measurements of MNHN/UL.C1 (in mm), e—estimated measurements.

	MNHN/UL.C1
Bizygomatic width (Minimum)	450 (e)
Width across exoccipitals	374
Bicondylar width	153
Width of the foramen magnum	55
Height of the foramen magnum	46
Width of ascending process of the maxilla	135
Length of the compound posterior process	78
Length of the neurocranium	429
Width between antorbital notches	273
Distance between dorsal margin of foramen magnum and supraoccipital apex	216
Distance between lateral margins of basioccipital crests	104
Greatest height of neurocranium	440
Diameter of primary infraorbital foramen	12
Diameter of foramen pseudovale	21
Total body length (Estimation by Pyenson and Sponberg (2011) equation [35])	4810

**Fig 11:**
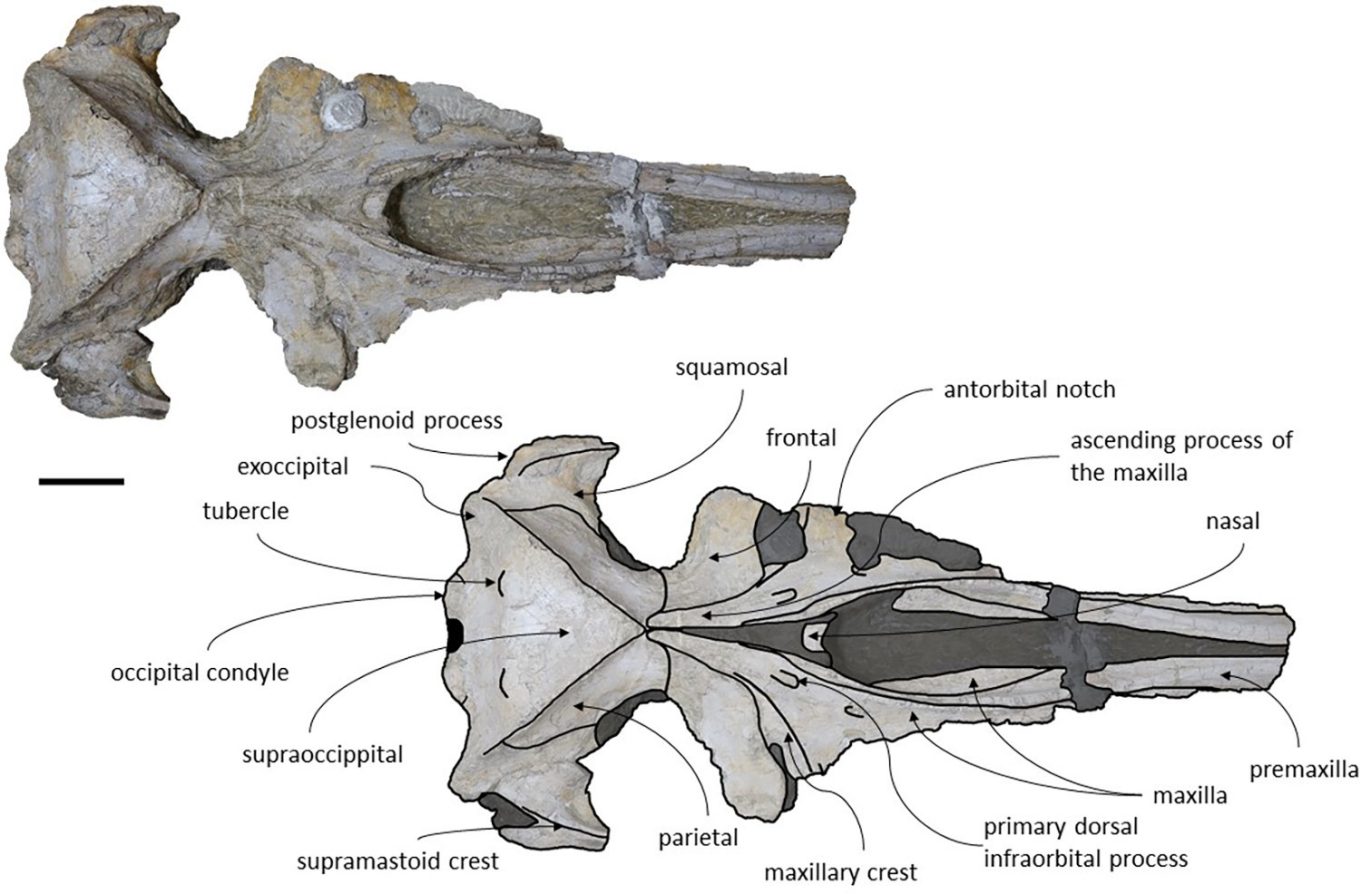
*Adicetus vandelli* (MNHN/UL.C1) in dorsal view. Scale– 100 mm.

*Maxilla*. The maxillae are almost complete. However, the remnants of the zygomatic processes, as preserved, suggest that the portion of maxilla anterior to the antorbital notches would be greater than the bizygomatic width. The lateral edges are damaged and show no evidence of teeth or alveoli. The maxillae contact: the frontals and parietals posteriorly, the nasals posteromedially, the premaxillae and vomer dorsomedially and the palatines ventrally. The suture between the maxillae and premaxillae is loose and they are separated by sediment. The maximum width between the medial margins of the maxillae (narial fossa) surpass the maximum width of the ascending processes of the maxillae. The suture between the maxillae and palatines forms a sigmoidal line. Dorsally the maxillae have four foramina preserved, two on each maxilla symmetrical to each other, while in ventral view there are no conspicuous foramina. The maxillae are sub-triangular in cross section, with a flattened dorsal surface. In dorsal view, the maxillae are exposed on the narial fossa medially to the premaxilla, forming a leaf shape (Fig 11). The antorbital process is present, although it is not fully preserved. There is a crest visible on the right maxilla parallel to the posterolateral edge near to the contact with the frontal, located in the posterior margin, that extends from the beginning of the ascending process to the lateral edge. The lateral portion of maxilla underlies the frontal and overrides it anteromedially. There is a gap between the frontal and maxilla which suggests that the lacrimal would be lateral to the ascending process of the maxilla and would overlap the maxilla, if preserved. The ascending processes of the maxillae contact each other posteromedially and are approximately parallel-sided is dorsal view, as in *Herentalia*, *Piscobalaena*, *Cetotherium* and *Tranatocetus* [5,20,34,52]. A primary dorsal infraorbital foramen is present at the base of the ascending process of the maxilla. Ventrally, the maxillae and vomer form a rounded palatal keel.

**Fig 12:**
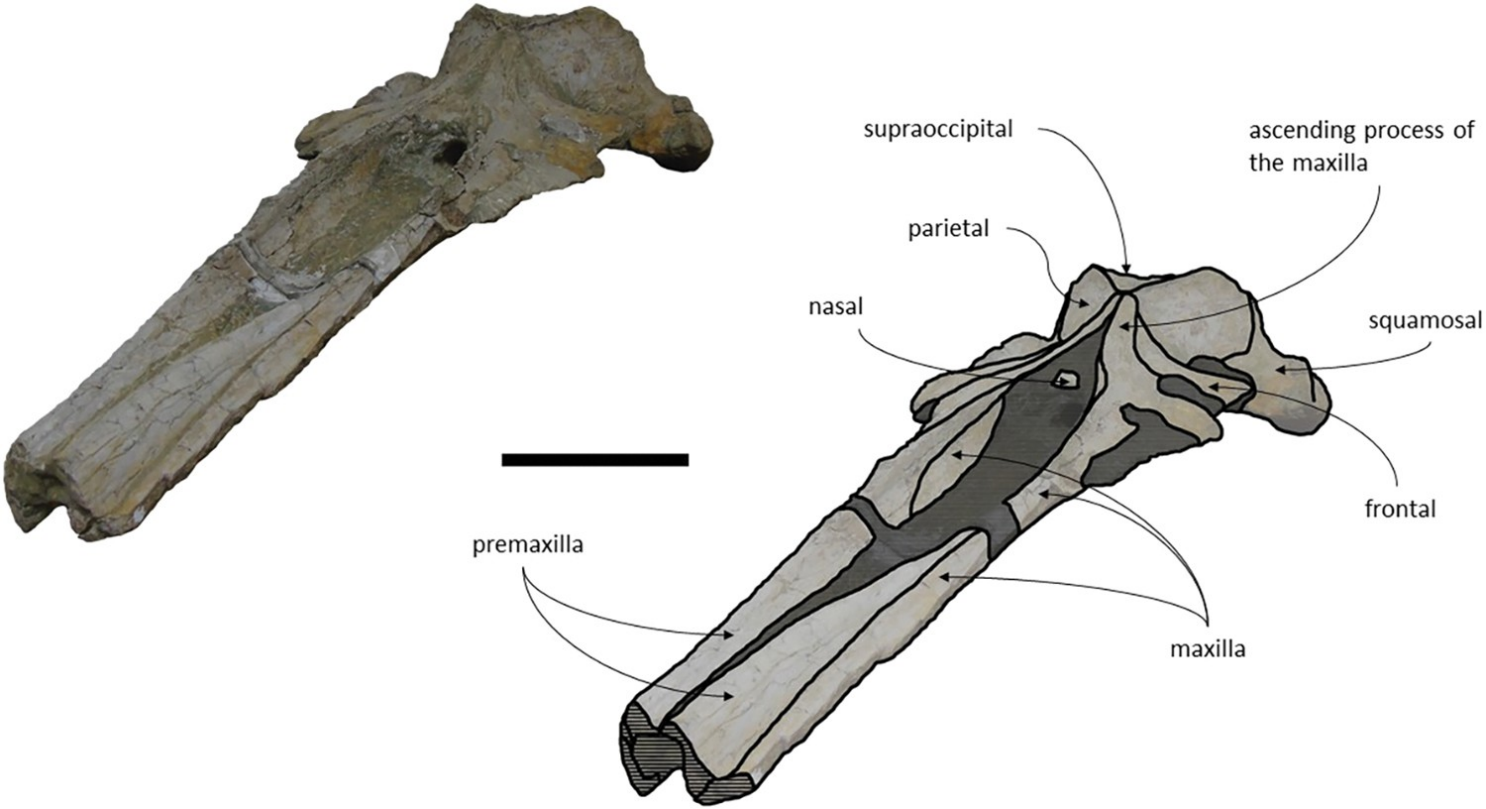
*Adicetus vandelli* (MNHN/UL.C1) in left anterolateral view.

*Premaxilla*. The anterior tips of the premaxillae are missing. The premaxillae contact: the nasals posteriorly and the maxilla and vomer ventrally. The premaxillae become wider and robust anteriorly in dorsal and lateral view. The premaxilla does not overhang the maxilla. The premaxillae nearly contact each other in the most anterior preserved portion. Contrarily to Gol’din and Steeman [15] interpretation, the premaxillae does not separate the maxillae from the nasals for all its length (Fig 12). There is a portion of premaxilla exposed on ventral view and there is no evidence of foramina in the premaxilla.

*Nasal*. The nasals are well preserved but are partially covered by sediment with the more anterior portion exposed. The nasals contact: the maxillae posterolaterally and the premaxillae laterally. In dorsal view, the nasals have a triangular shape with the lateral edges converging posteriorly. The anterior edge of the nasals is straight, and their dorsal surface is flat, as in *Cetotherium riabinini* and *Ciuciulea davidi* [19,34].

**Fig 13:**
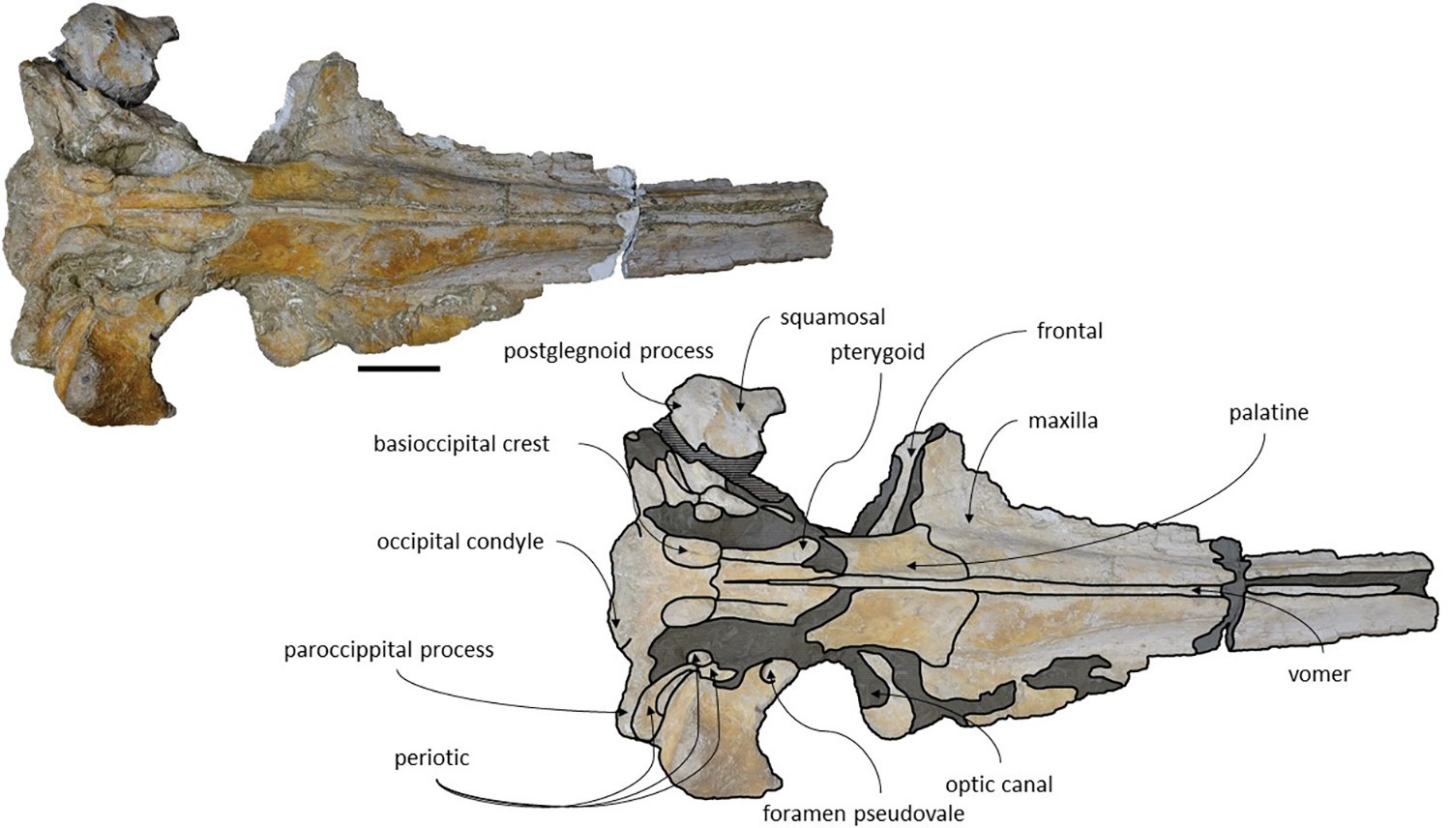
*Adicetus vandelli* (MNHN/UL.C1) in ventral view. Scale– 100 mm.

*Palatine*. The palatines are almost complete and are only slightly damaged posteriorly. The palatines contact: the pterygoids posteriorly, the vomer dorsally, the frontals laterally and the maxillae anteriorly. The suture between the palatines and maxillae is sigmoidal, similar to *Piscobalaena* [5]. Each palatine has a trapezoidal shape and is longer than wide (Fig 13). The anterior most point of the palatines is located posterior to the level of the antorbital notch. Although the left zygomatic process is not complete, the posterior most point of the palatines would be located behind the tip of the zygomatic process since it is in line with the preserved end of the zygomatic process. The lateral margins of the palatines underlap the medial margin of the infraorbital plate of maxilla. The choanal margin is concave. The medial portion of the palatines forms an angular keel.

*Vomer*. The vomer is complete in length and only the posteroventral margins are eroded. The vomer contacts the basioccipital posteriorly, the pterygoids and palatines ventrally, and the premaxillae and maxillae dorsally. In cross section it is “V” shaped. In ventral view, the vomer is posteriorly aligned with the anterior edge of the basioccipital crest. In ventral view, it is visible through all its length. The ventral edge of the palatine is more ventral than the ventral edge of the vomer. The sagittal length of the vomerine crest decreases gradually from the palatines to the anterior most point of the basioccipital crest.

*Pterygoid*. The medial laminae of the pterygoids are almost entirely preserved and exposed. The ventral laminae are covered by sediment and the pterygoid hamuli are not preserved. The pterygoids contact the basioccipital and squamosals posteriorly, the palatines anteriorly, and the vomer dorsally. The pterygoids contribute to the anterior wall of the foramen pseudovale. The anterior most point of pterygoid sinus fossa is in line with the anterior edge of foramen pseudovale.

*Basioccipital*. The basioccipital crests are long and bulbous, with straight lateral borders. The basioccipital is well preserved with only the left crest slightly eroded. The basioccipital contacts the exoccipitals posteriorly and the pterygoids, vomer and basisphenoid anteriorly. The basioccipital and the exoccipital are fused. The anterior most point of the basioccipital crest is in line with the anterior edge of the postglenoid process in ventral view. The basioccipital/basisphenoid contact is not exposed.

**Fig 14:**
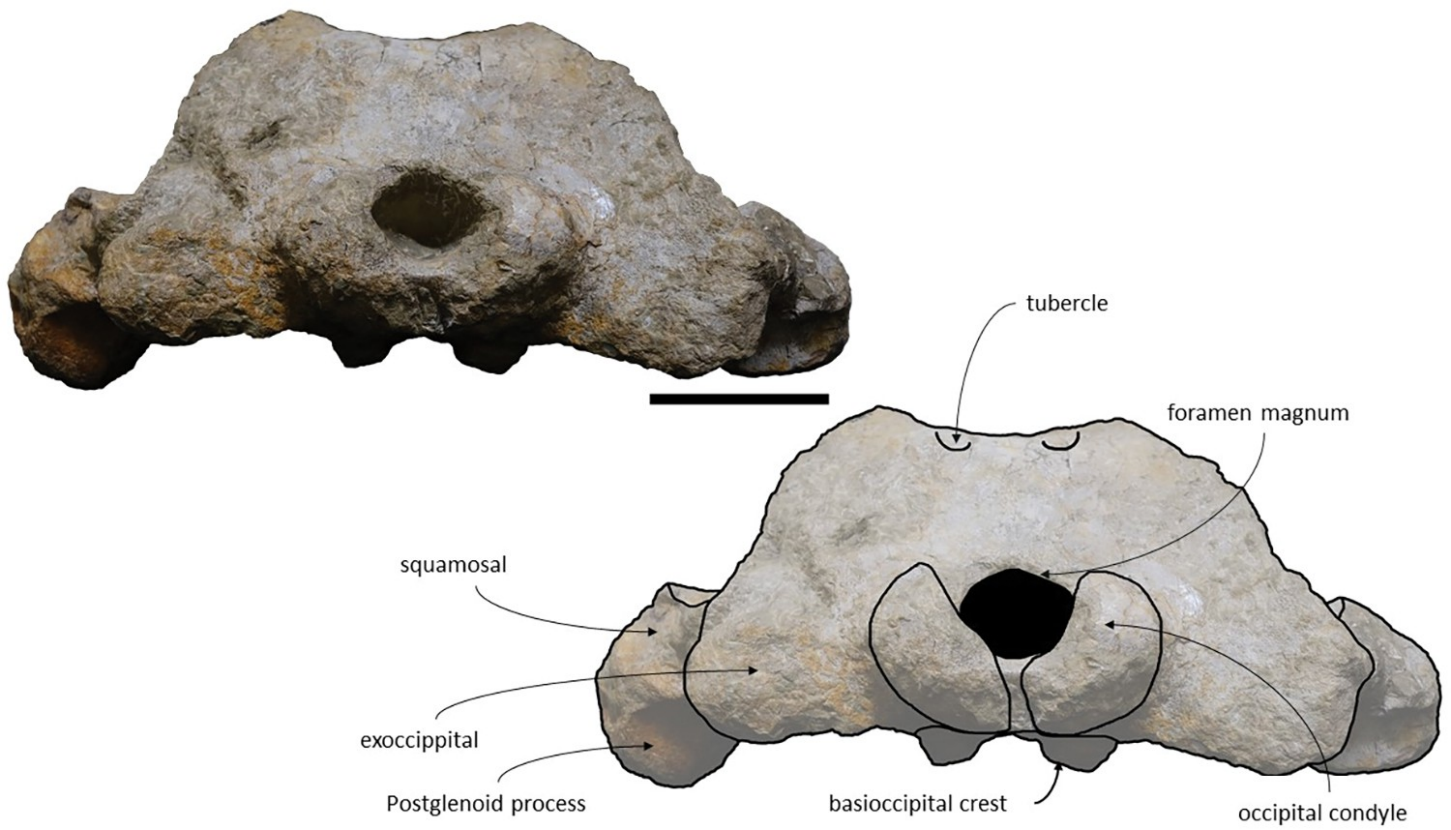
*Adicetus vandelli* (MNHN/UL.C1) in posterior view. Scale– 100 mm.

*Squamosal*. The squamosals are almost complete, however, the anterior portions of the zygomatic processes of the squamosal are missing. The squamosals contact the exoccipitals posteriorly, the periotics ventromedially, pterygoids and alisphenoids anteriorly, and the parietals anterodorsally. The squamosal is longer, anteroposteriorly, than high. The foramen pseudovale is located almost entirely in the anterior portion of squamosal, with the pterygoid contributing partially to the anteromedial portion of the foramen pseudovale wall. The lateral border of the squamosal is not continuous with the lateral edge of the exoccipital. The squamosal is wider than high, including the postglenoid and zygomatic processes. In posterior view, the postglenoid process is in line with the lateral edge of the skull and is orientated ventrally (Fig 14). In lateral view, the tip of the postglenoid process points posteriorly and it is more ventral than the ventral edge of the exoccipital. In ventral view, the squamosal opens a window around the anterior process, anterior to the acoustic meatus and posterior to the foramen pseudovale. There is no squamosal cleft.

*Frontal*. The frontals are partially damaged, mainly in the posterolateral margins. The frontals contact the parietals posteriorly, the palatines ventrally and the maxillae anteriorly. The frontal is excluded from the skull vertex, being completely overlapped by the ascending processes of the maxillae. Although the posterior portion of the supraorbital process of the frontal is damaged and partially covered by sediment, the medial portion of the postorbital ridge is anteroposteriorly thin. The right preorbital is thicker than the more central portion of the preserved orbit. The anterior and posterior borders of the supraorbital process of the frontal converge medially. The anterior edge of the supraorbital process of the frontal is concave and transversely oriented. The optic canal is ventrally open, and it is adjacent to the posterior border of the supraorbital process of the frontal. Although the lacrimal is not preserved, it is possible to infer that the anterior border of the supraorbital process of the frontal was not bordered by the lacrimal only, since there is contact with the maxilla.

**Fig 15:**
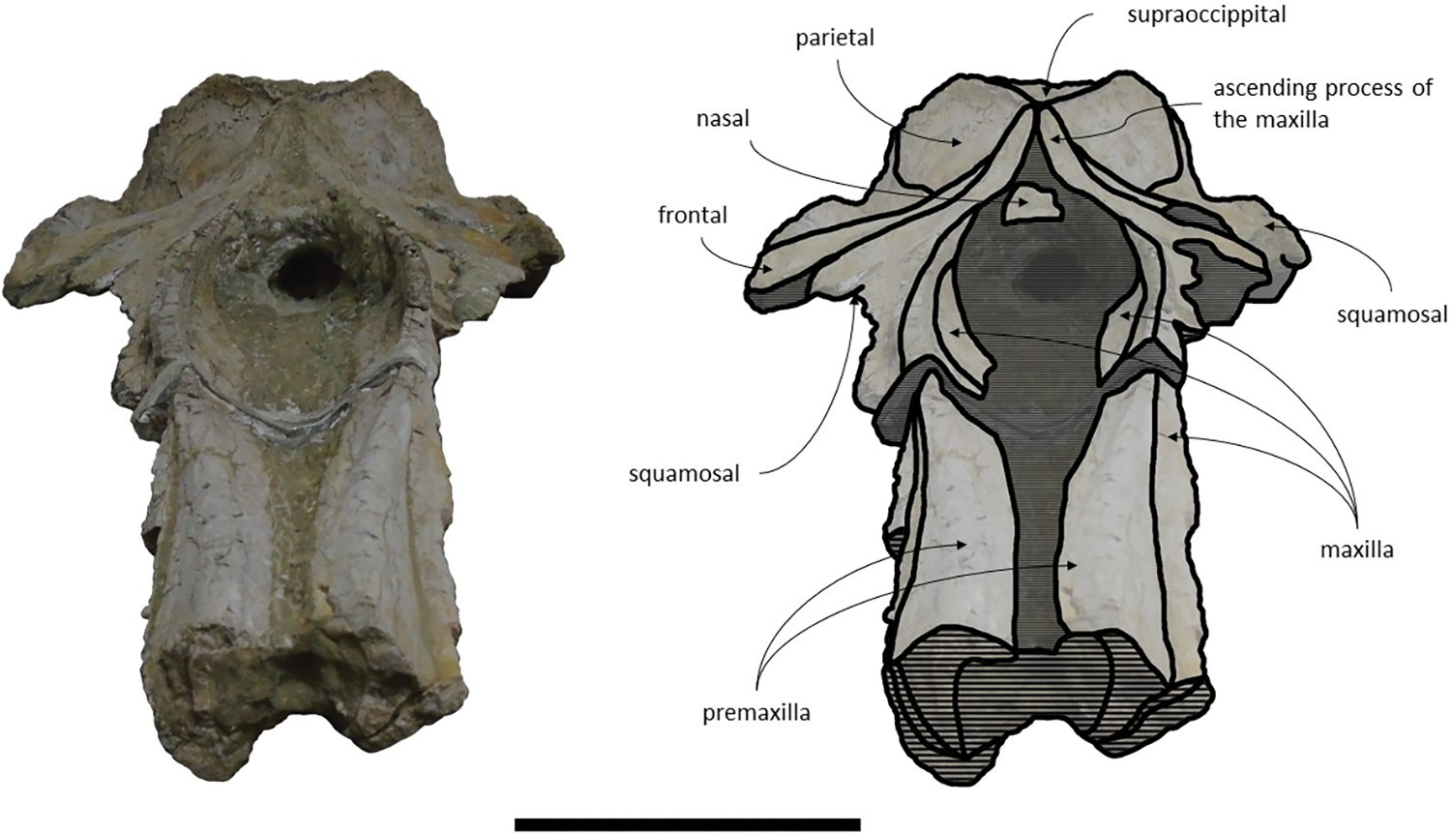
*Adicetus vandelli* (MNHN/UL.C1) in anterior view.

**Fig 16:**
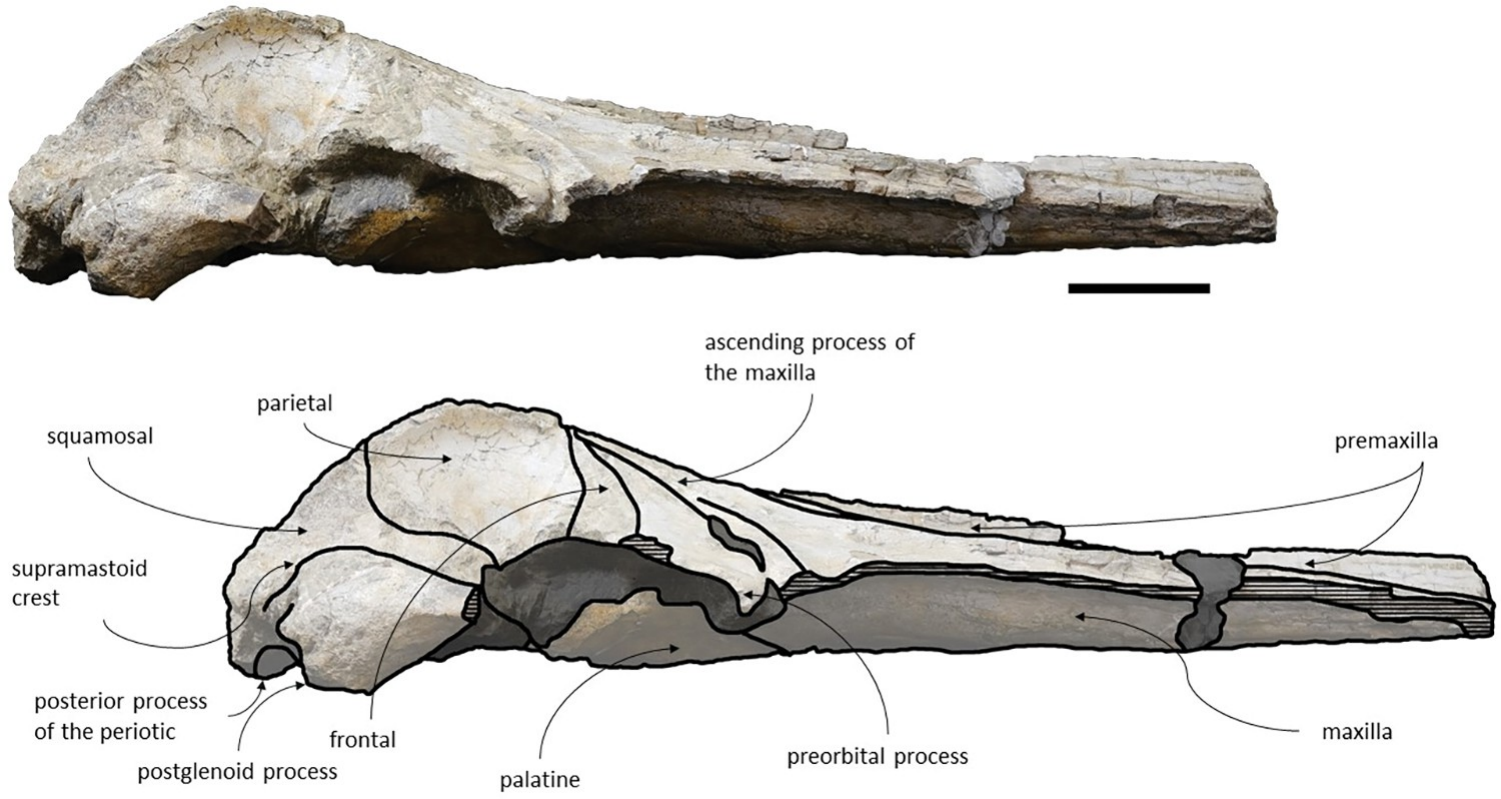
*Adicetus vandelli* (MNHN/UL.C1) in right lateral view. Scale– 100 mm.

*Parietal*. In anterior view, the two parietal bones expand laterally forming a subtriangular-shape (Fig 15). In lateral view, the parietal is longer than high (Fig 16). The parietals are almost totally complete, with only the anteroventral portions damaged. The parietals contact the supraoccipital dorsally and posteriorly, the squamosals ventrally, the alisphenoids anteroventrally, and the frontals and maxillae anteriorly. The fronto-parietal suture is irregular. The suture between the parietal and squamosal is sigmoidal, similar to *Cetotherium*, *Herentalia*, *Herpetocetus*, *Piscobalaena*, *Tranatocetus* and *Tiucetus* [5,12,18,20,34,52]. The parietals are more anterior than the posterior end of the ascending processes of the maxillae. The interparietal region is extremely reduced.

*Supraoccipital and exoccipital*. The supraoccipital and exoccipital have a trapezoidal shape in posterior view and a triangular shape in dorsal view. The supraoccipital is fully preserved, but the nuchal crest is partially damaged. The supraoccipital contacts the exoccipitals posteriorly and the parietals anteroventrally. The exoccipitals are almost complete, displaying some erosion mainly in the ventral portion of the left exoccipital. The exoccipitals contact the basioccipital ventrally, the squamosals laterally, the periotics ventrolaterally, and the supraoccipital anteriorly. The most anterior portion of the supraoccipital is concave dorsally and the posterior portion is straight. In dorsal view, the nuchal crest projects dorsolaterally and is slightly sigmoidal, with its anterior portion bowed inward and its posterior portion bowed outward, similar to *Tiucetus rosae* [18]. The supraoccipital apex is pointed. There are two faint tubercles situated between the supraoccipital and the exoccipitals, less marked than the paired tubercles observed in *Archaebalaenoptera castriarquati* [54]. A faint external occipital crest is visible with a tangent light and noticeable by touch, that runs from the concave portion to the anterior edges of the occipital condyles but vanishes between the paired tubercles. The posteriormost point of the nuchal crest is anterior to the posterior edge of the occipital condyles. The occipital condyles are confluent with the exoccipitals.

**Fig 17:**
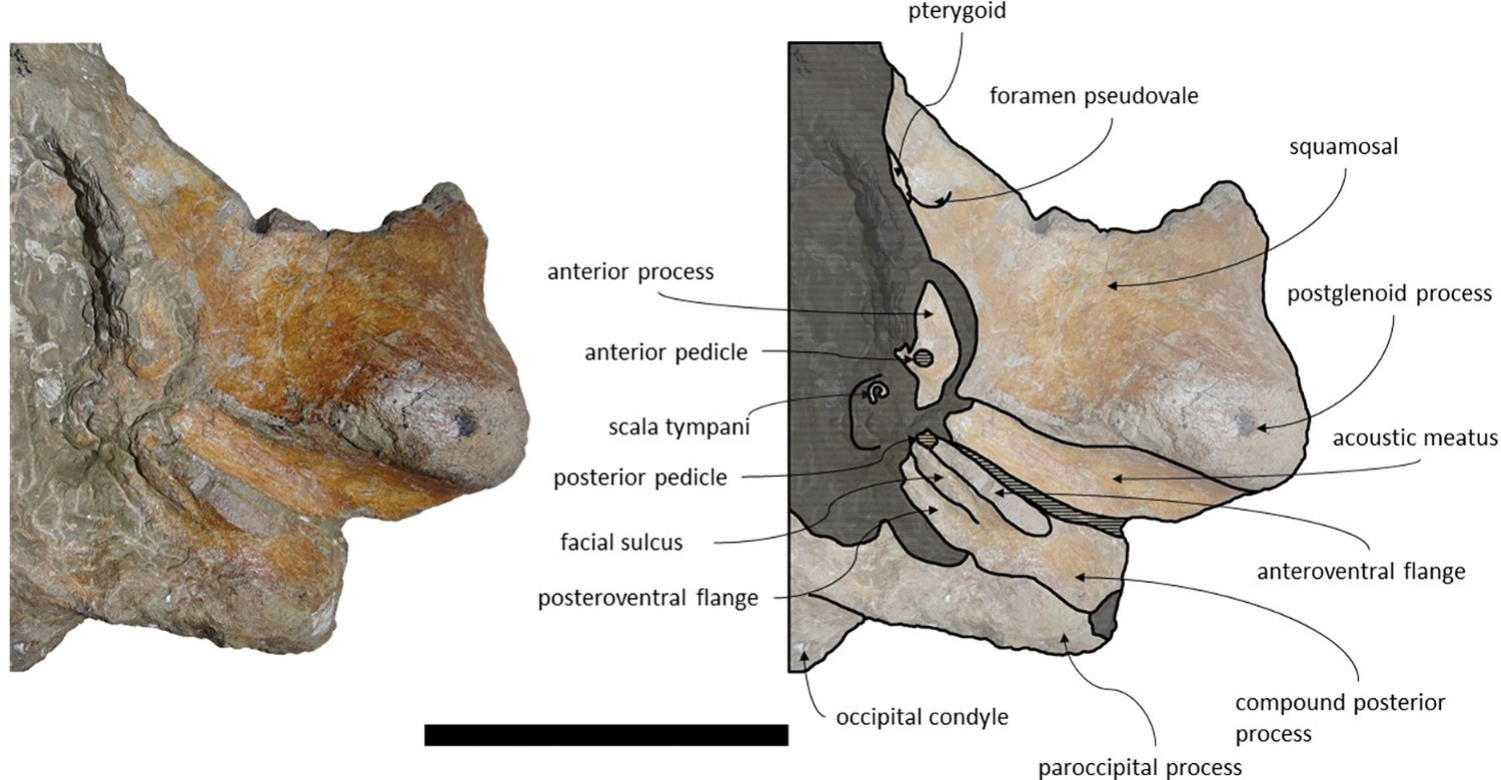
**Posterolateral portion (left side) of *Adicetus vandelli* (MNHN/UL.C1) cranium in ventral view.** Scale– 100 mm.

*Periotic and tympanic bulla*. Both periotics are exposed, however, partially damaged (Fig 17). The outer layer of the pars cochlearis is missing, exposing a portion of the scala tympani. Both tympanic bullae are missing. The tympanic bullae are located ventrally to the periotics. The periotics contact the exoccipitals posteriorly and the squamosals laterally. The posterior process is visible in lateral view. In medial view, the anterior process of the periotic presents a sub-squared shape with a rounded anteroventral angle. The anterior process is more ventral than the pars cochlearis, in medial view. It is possible to infer that the anterior process of the periotic is longer than the pars cochlearis since there are traces of the outer layer of the pars cochlearis. The posterior cochlear crest is close to contact with crista parotica. The lateral tuberosity is faint. The compound posterior process is cylindrical and is oriented posterolaterally relative to the longitudinal axis of the anterior process, sharing these two traits with *Brandtocetus*, *Cetotherium*, *Cephalotropis nectus*, *Kurdalagonus*, Joumocetus and *Tiucetus* [9,13,18,34,53]. The facial sulcus is located adjacent to the posterior border of the compound posterior process, is partially floored by a posteroventral flange and posteriorly open. The neck of compound posterior process is markedly constricted. The compound posterior process is exposed, but distinct, in the lateral skull wall.

### *1*. *Cephalotropis nectus*

#### (a) Systematic palaeontology

**Cetacea** Brisson, 1762 [44]

**Neoceti** Fordyce and de Muizon, 2001 [45]

**Mysticeti** Gray, 1864 [46]

**Chaeomysticeti** Mitchell, 1989 [47]

**Cetotheriidae** Brandt, 1872 [1]

***Cephalotropis*** Cope, 1896 [56]

**Type species.** *Cephalotopis nectus* Kellogg, 1941

### Emended diagnosis (Genus)

Medium-sized cetotheriid sharing with the other family members posteriorly convergent ascending processes of the maxillae and nasals and an enlarged compound posterior process of the tympanoperiotic with its facial sulcus floored by a posteroventral flange. Shares with all cetotheriids except *Brandtocetus* and *Cetotherium* a posteriorly wider portion of the tympanic bullae; with all cetotheriids except *Tiucetus*, *Nannocetus*, *Adicetus vandelli* and “*Cetotherium” megalophysum* a paroccipital process in line or posterior to the posterior edge of the occipital condyle, in dorsal view. Differs from all cetotheriids by lacking a wider than long temporal fossa; from all cetotheriids except *Tiucetus* by lacking a dorsal primary infraorbital foramen; from all cetotheriids except *Tiucetus*, *Brandtocetus*, *Kurdalagonus*, *Adicetus* in having a sigmoidal lateral edge of the supraoccipital, in dorsal view; from all cetotheriids except *Tiucetus*, *Joumocetus* and *Ciuciulea* in having a great portion of the parietal exposed on the skull vertex; from all cetotheriids except *Tiucetus*, *Joumocetus*, *Ciuciulea* and *Metopocetus* in having an approximately triangular shaped ascending processes of the maxillae. Further differs from *Tiucetus*, *Brandtocetus*, *Kurdalagonus*, *Cetotherium* and *Tranatocetus* by lacking a squamosal prominence; from *Herpetocetus* and *Piscobalaena* in having “U” shaped anterior margins of the nasals; from *Cetotherium*, *Piscobalaena* and *Adicetus* by having the anteromedial portion of the palatines forming a well-developed medial crest; from *Piscobalaena* and *Adicetus* by having a straight outline of the suture between the palatines and maxillae; from Herpetocetus for lacking a opening between the parietal and squamosal; and from *Adicetus* for lacking a faint pair of tubercles on the supraoccipital.

*Cephalotropis nectus* Kellogg, 1941 [31]

### Holotype

MNHN/UL.C3, partial skull comprising the braincase, incomplete periotic bones, the right bulla and incomplete rostrum.

### Emended diagnosis (species)

Shares with *Cephalotropis coronatus* the sigmoidal lateral edges of the supraoccipital, the exposure of the parietal in the skull vertex, elongated nasals, a rounded temporal fossa, protruding paroccipitals and a sub-triangular supraoccipital with a concave dorsal surface and a pointed apex. Differs from *Cephalotropis coronatus* in having a sharper external occipital crest, an irregular fronto-parietal suture, the maxilla separated by both nasals and premaxillae, the medial edges of the nasals forming a sagittal keel, the facial sulcus running along the posterior edge of the compound posterior process, a slightly conical compound posterior process, rounded tympanic bullae and also in lacking a squamosal cleft.

### Locality and horizon

The specimen here described was collected in 1831 by Vandelli in a gold mine located at Adiça beach, between Fonte da Telha and Lagoa da Albufeira, in Costa de Caparica, Almada, Portugal (38° 33’ 9’‘ N, 9° 11’ 12’‘ W. [25]). The skull [26] was retrieved from the Miocene, lower Tortonian [49], Lisbon Miocene Cotter’s division VIIb (ca. 10 Ma) [50]. According to Vandelli [24], the skull was found embedded in thick, dark green limestone, full of invertebrate shells.

### (b) Anatomical Description

**MNHN/UL.C3 (*Cephalotropis nectus*, Kellogg 1941 [31]; *Balenoptera* sp, Jacinto Gomes 1915–16 [26])**

#### Overview

**Fig 18:**
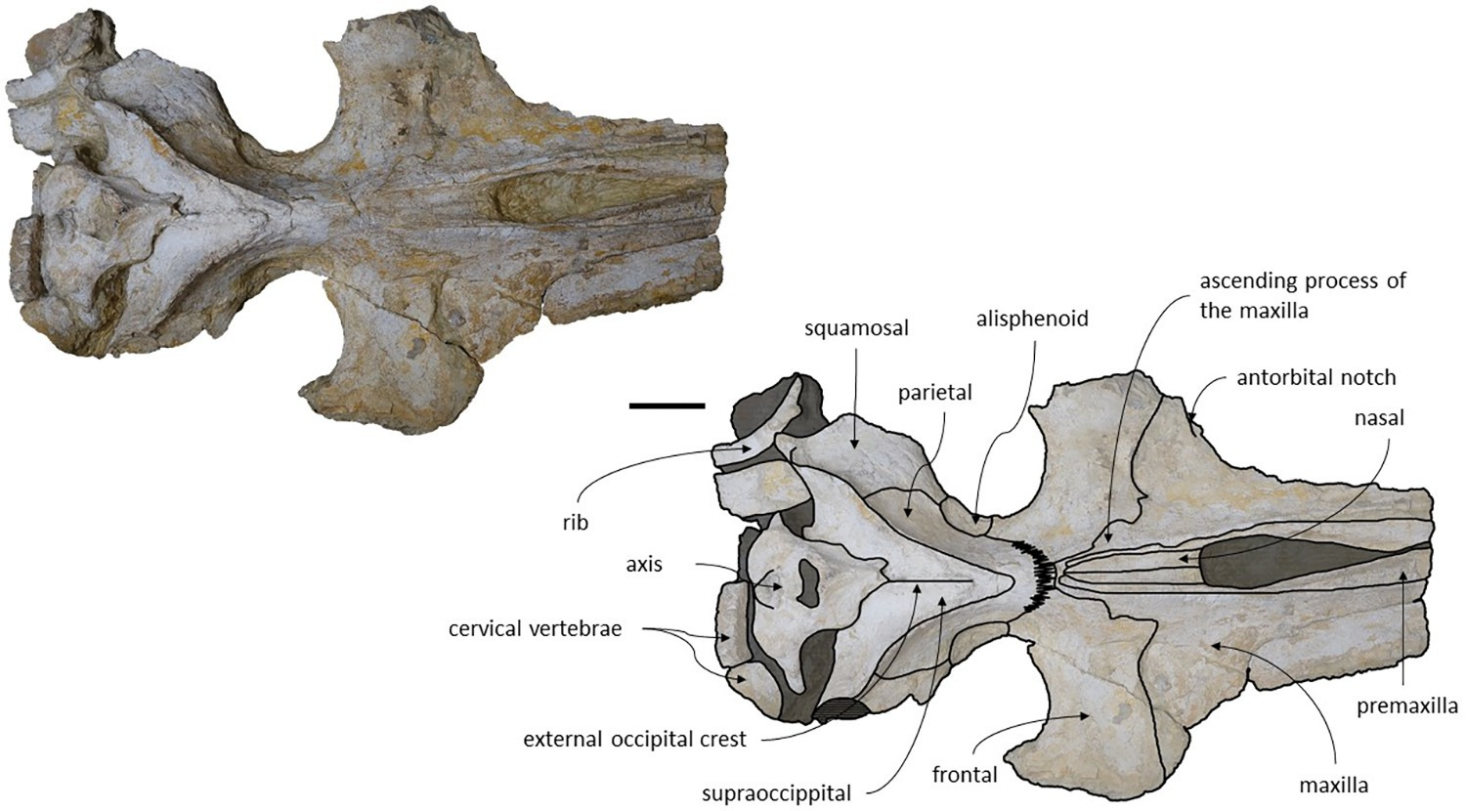
Holotype cranium of *Cephalotropis nectus* in dorsal view. Scale– 100 mm.

**Fig 19:**
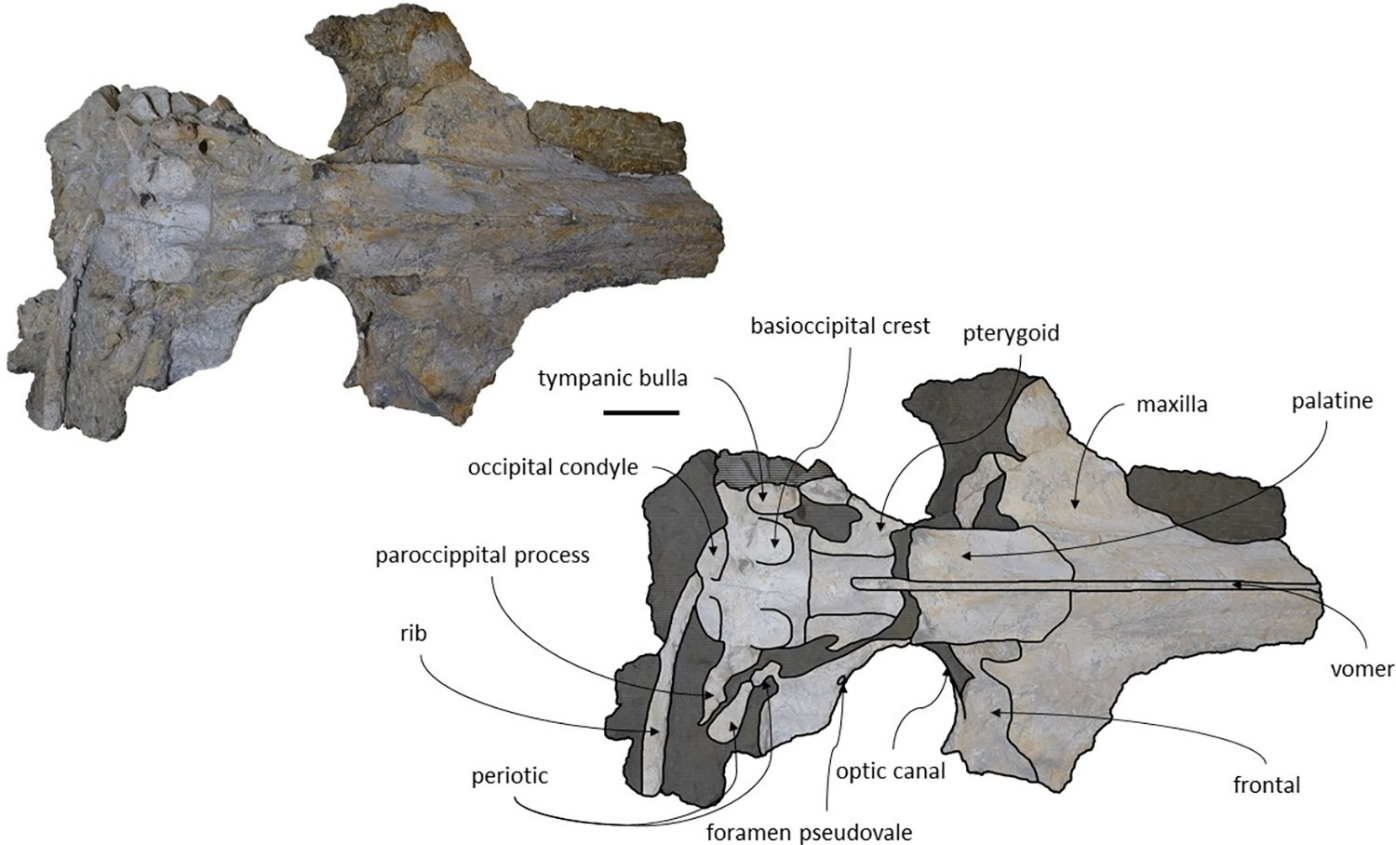
Holotype cranium of *Cephalotropis nectus* in ventral view. Scale– 100 mm.

The specimen MNHN/UL.C3 consists of an almost complete skull, missing the most anterior portion, a great portion of the squamosals (Fig 18), the left tympanic bulla (Fig 19), and the mandible. All sutures are fused, indicating that this specimen represents an adult individual. The bone surface is slightly eroded and fragile, however some of the muscle scars are still visible. There are two main fragments that are separated from the skull, the lateral portion of the right maxilla and lateral portion of the right frontal. Both fragments can be easily replaced according to the original post-mortem fractures. The ventral surface of the rostrum forms an angular keel. Posteriorly, the occiput is partially covered by sediment, the axis, cervical vertebrae, a possible fragment of a scapula and some unarticulated ribs. There are no other post-cranial elements present. The measurements of the specimen are represented in Table 3.

**Table 3:** Measurements of MNHN/UL.C3 (in mm), e—estimated measurements.

	MNHN/UL.C3
Bizygomatic width	472 (e)
Width across exoccipitals (minimum)	388 (e)
Bicondylar width	140 (e)
Width of the foramen magnum	34
Width of ascending process of the maxilla	144
Length of the compound posterior process (minimum)	84 (e)
Length of tympanic bulla	61
Width of the tympanic bulla at sigmoidal process	42
Width of the tympanic bulla anterior to sigmoidal process	36
Width of the tympanic bulla posterior to sigmoidal process	41
Length of the neurocranium	514
Width between antorbital notches	366
Length of nasals	172
Distal width of nasals	40
Distance between posterior margin of nasal and apex supraoccipital	59 (e)
Distance between lateral margins of basioccipital crests	151
Greatest height of neurocranium	499 (e)
Total body length (Estimation by Pyenson and Sponberg (2011) equation [35])	5026

**Fig 20:**
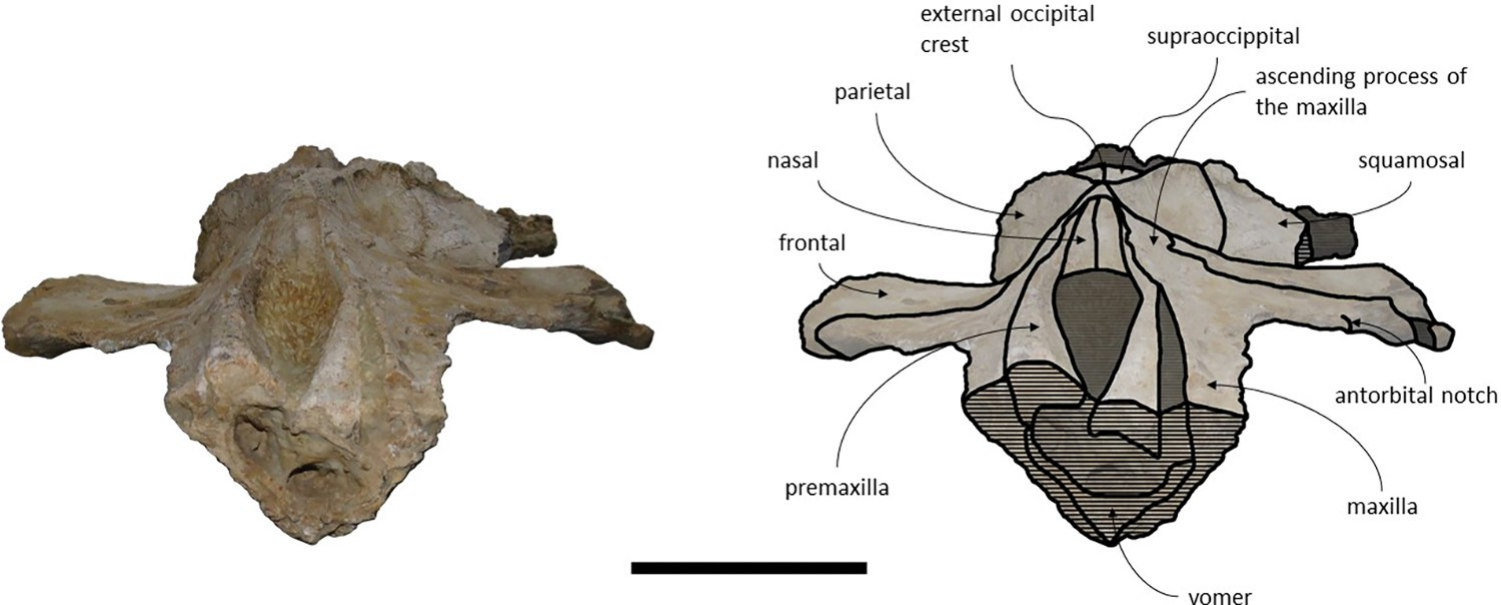
Holotype cranium of *Cephalotropis nectus* in anterior view.

**Fig 21:**
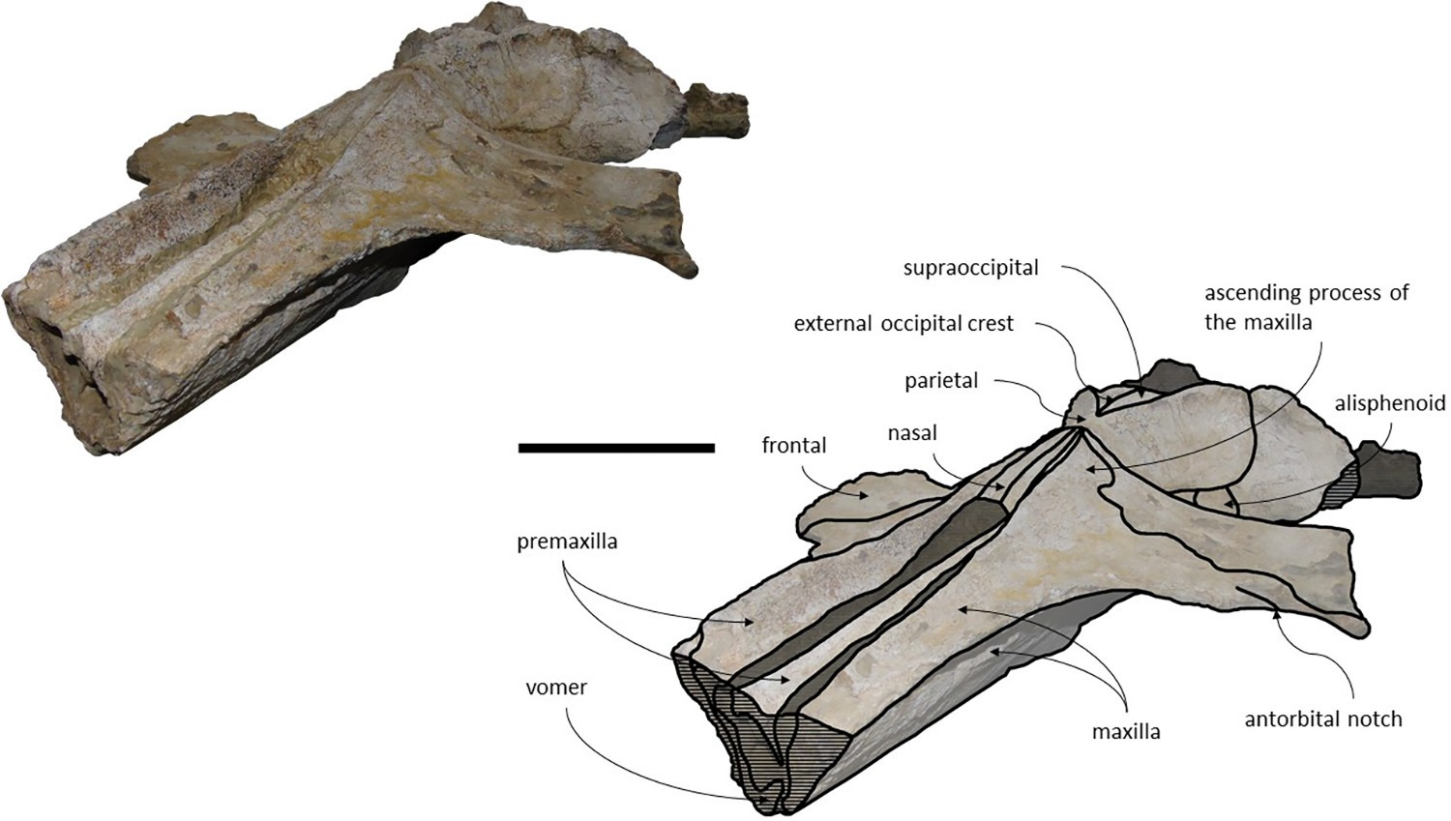
Holotype cranium of *Cephalotropis nectus* in left anterolateral view.

*Maxilla*. The maxillae are well preserved but the anterior most portion of the maxillae is missing. The lateral edges are eroded but not as much as MNHN/UL.C1 and MNHN/UL.C2. The maxilla is sub-triangular with a flattened dorsal surface in cross section (Fig 20). The maxillae contact the frontals and parietals posteriorly, the nasals posteromedially, the premaxillae and vomer dorsomedially and the palatines ventrally. The sutures between maxillae and premaxillae are loose. The antorbital process is well preserved and forms a steep facet posteriorly bordering the antorbital notch. The lateral process of the maxilla forms an obtuse angle in the antorbital notch with the rest of the maxilla (Fig 21). The lateral portion of the maxilla underlies the posterior margin of the frontal. Near the contact with the frontal, lateral to the ascending process of the maxilla and posterior to the antorbital notch, is a wide, leaf shape depression. There are some nutrient foramina visible in ventral view. In dorsal view, a foramen is visible in the left maxilla near the premaxilla but given that the bone texture is slightly eroded we could not identify other foramina beyond a reasonable doubt. The maxillae form, with the vomer, a pronounced and sharp keel, anteroposteriorly. The medial margin of the infraorbital plate is well pronounced. In ventral view, the bone surface of the maxilla presents a longitudinal sulcus anteroposteriorly oriented, on each side. However, the ventral surface of the right maxilla is more eroded and displays a less marked sulcus. There is a prominence in the ventral sutures between maxillae and palatines, on each side.

*Premaxilla*. The premaxillae are also largely preserved, although, the anterior portion is missing, and the left premaxilla is medially displaced. The premaxillae contact the nasals posteriorly, the maxilla and vomer ventrally. The premaxilla has detached from the maxilla. Although the anterior portion of the premaxillae are missing, it is possible to infer that the maxillae start to become wider in the anterior half and would be wider than the maxilla on the anterior end. The bone surface is eroded which precludes the identification of foramina.

*Nasals*. The nasals are completely preserved and contact the frontals posteriorly, and the maxillae and premaxillae anterolaterally. The nasals are longer than the ascending process of the maxilla, with a total length of 172 mm. The lateral edges are parallel over almost the entire length, converging slightly in the posteriormost portion. The anterior edge of the nasals is U shaped, as in *Archaebalaenoptera castriarquati* and Joumocetus shimizui [9,54]. The lateral and medial edges of each nasal are more elevated than their axis, originating depressed central portions.

*Palatines*. The palatines are longer than wide and have a sub-rectangular shape in lateral view. The palatines contact the maxilla and frontal anteriorly, the pterygoids posteriorly, and vomer dorsally. The suture between the palatines and maxillae are transversely straight, as in *Cetotherium*, *Ciuciulea*, *Herpetocetus morrowi*, *Joumocetus shimizui Metopocetus hunteri* and *Tiucetus rosae* [9,12,16,18,19,34]. The anteriormost point of the palatines is posterior to the antorbital notch. The shape of the choanal margin cannot be determined because it is not well preserved. The medial portion of the palatine forms an angular keel that represents the ventralmost point of the preserved skull. The palatines form the posterior half of a prominence present in the ventral sutures between maxillae and palatines, on each side.

*Vomer*. In anterior view, the vomer is V shaped in cross section and has its ventral apex exposed over all its length, between the maxillae. The vomer contacts the basioccipital posteriorly, the palatines ventrally, the pterygoids laterally, and the premaxillae and maxillae dorsally. The vomerine crest has approximately half the length of the portion of vomer between palatines and basioccipital.

*Pterygoid*. The pterygoid medial laminae are preserved but the hamuli are not preserved. The pterygoids contact the basioccipital and squamosals posteriorly, the palatines anteriorly, and the vomer medially. The anterior most point of pterygoid sinus fossa is approximately in line with the anterior edge of foramen pseudovale. The pterygoids are partially covered by palatines.

*Basioccipital*. The basioccipital is subrectangular in ventral view and the basioccipital crests are wide and bulbous with straight lateral borders. The basioccipital contacts the exoccipitals posteriorly and the pterygoids and vomer anteriorly. The basioccipital/basisphenoid contact is not exposed. The sutures between the basioccipital and exoccipital are absent which forms a basioccipital region completely fused.

*Squamosal*. The lateral and ventral portions of both squamosals are not preserved; however, it is possible to identify some sutures on the left side. The squamosals contact the exoccipitals posteriorly, the periotics ventromedially, pterygoids and alisphenoids anteriorly, and the parietals dorsally. The foramen pseudovale is visible on the left side of the anterior portion of squamosal, near the suture with the pterygoid. The most posterior point of the nuchal crest is situated anterior to the exoccipital. The squamosal fossa is wide and well excavated.

**Fig 22:**
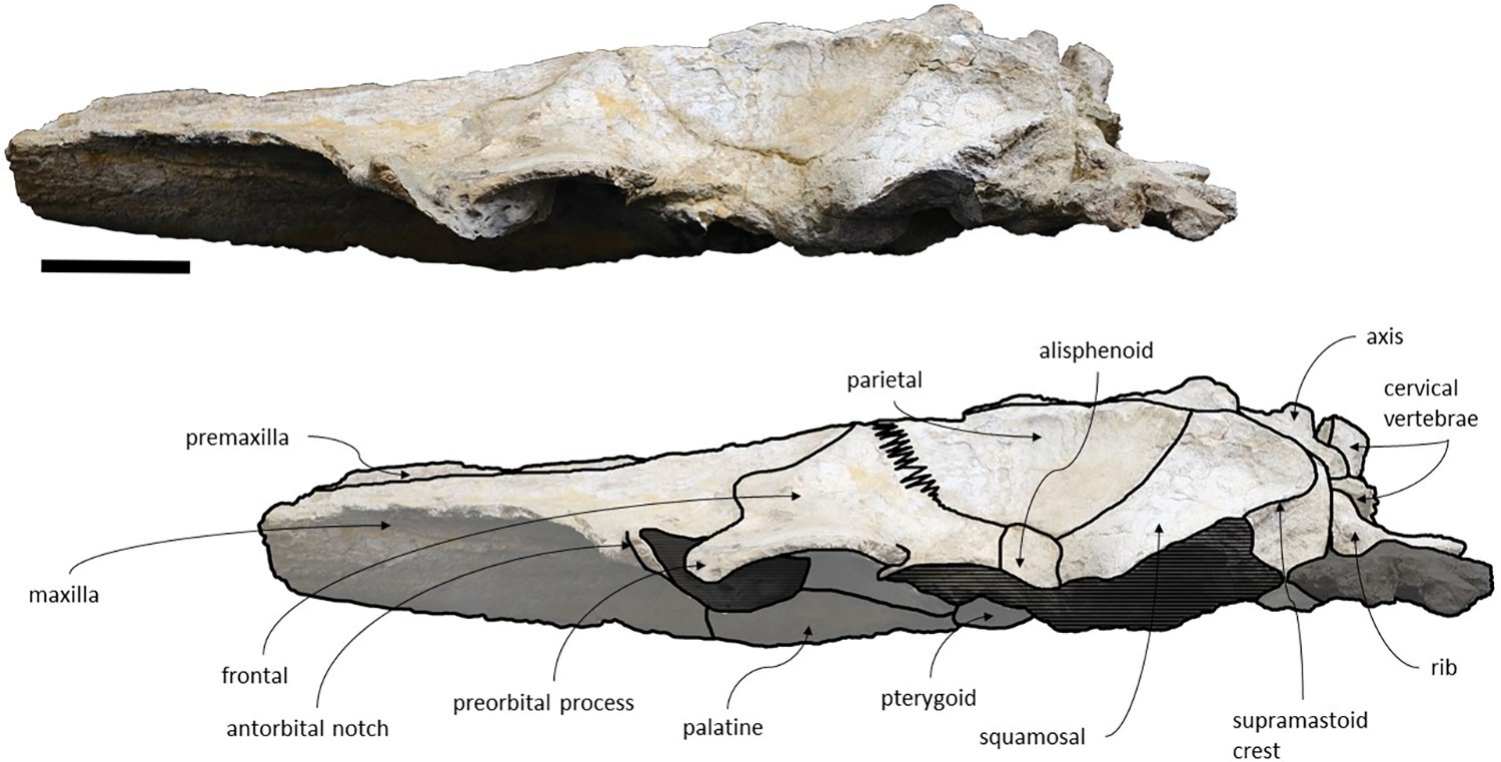
Holotype cranium of *Cephalotropis nectus* in left lateral view. Scale– 100 mm.

*Frontal*. The frontals are well preserved, yet the right frontal is fractured. The frontals contact the parietals posteriorly, the palatines and alisphenoids posteroventrally, and the maxillae and nasals anteriorly. The anteriormost point of the supraorbital process of the frontal is roughly in line with the middle of the nasals. Although the lacrimal is not preserved, it is possible the anterior border of the supraorbital process of the frontal was bordered by the lacrimal and maxilla, as there is a gap between the lateral portions of frontal and maxilla (Fig 22). In dorsal view, the anterior edge of the supraorbital process of the frontal is concave and pointing anteriorly, the posterior edge is concave, and both edges diverge medially and laterally. The postorbital process is posteriorly oriented, in dorsal view, and rounded, in lateral view. The dorsal surface of the supraorbital process of the frontal presents a faint orbitotemporal crest, subparallel to the posterior edge of the supraorbital process of the frontal. In ventral view, the optic canal is filled with sediment and is adjacent to the posterior border of the supraorbital process of the frontal.

**Fig 23:**
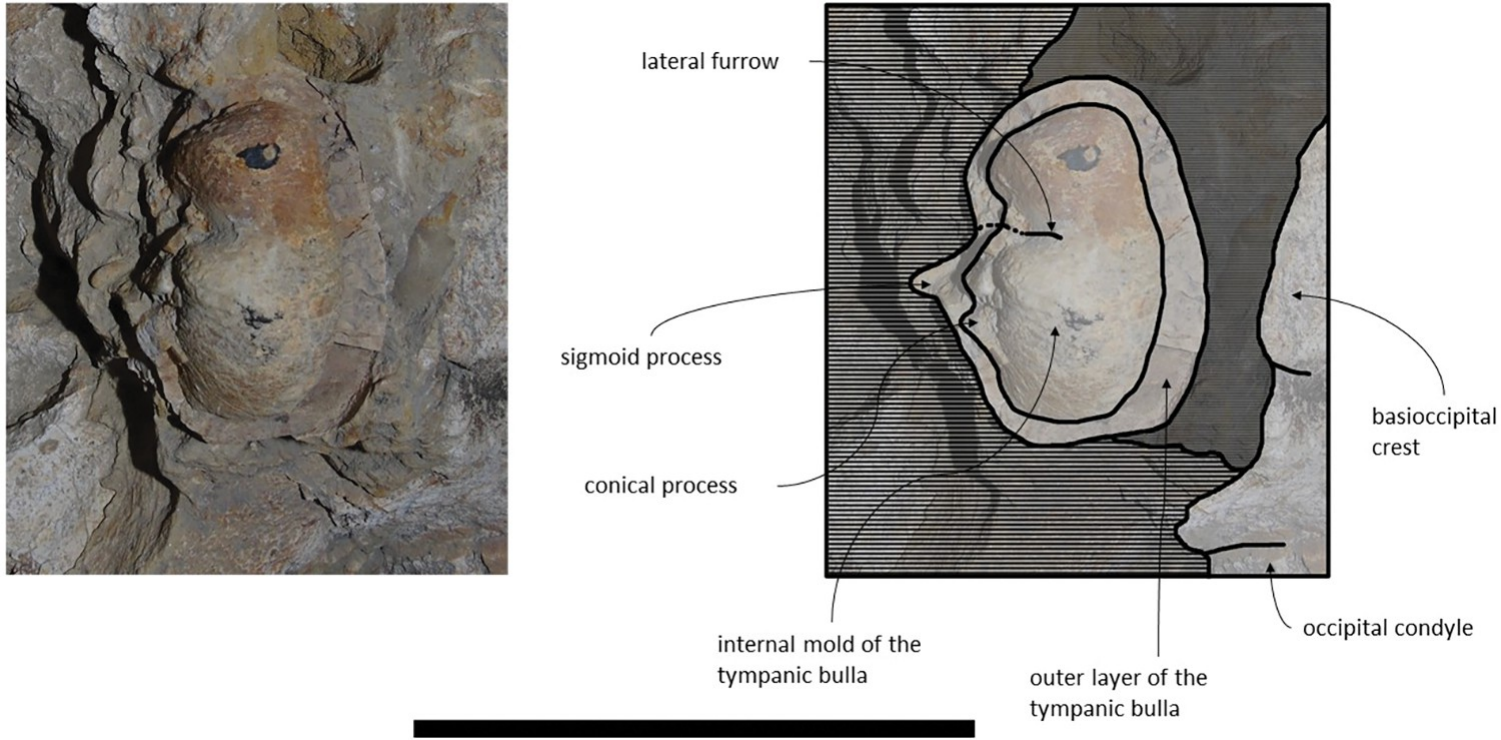
Right tympanic bulla of the holotype of *Cephalotropis nectus* in ventral view. Scale– 100 mm.

*Periotic and Tympanic Bulla*. Both periotics are incomplete and fragmented. The right tympanic bulla is preserved *in situ;* however, its ventral bony outer layer is missing (Fig 23), and the left bulla is missing. The periotics contact the exoccipitals posteriorly and the squamosals laterally. The right tympanic bulla is located ventrally to the right periotic. The anteroventral angle of the anterior process of the periotic is blunt. The compound posterior process of the periotic is exposed on the lateral skull wall, as in *Brandtocetus*, *Cetotherium*, *Kurdalagonus mchedlidzei* [13,34,53]. The compound posterior process is thick, cylindrical and is posterolaterally oriented relative to the anteroposterior axis of the skull. The facial sulcus is located adjacent to the posterior border of the posterior process and is anteriorly floored by a posteroventral flange. The main axis of the tympanic bulla is parallel to the sagittal plane of the skull. In dorsal view, the anterior border of the tympanic bulla is rounded, and the outline of the main ridge is convex. The posterior portion of the tympanic bulla is wider than the anterior portion (see Table 3). In lateral view, the lateral furrow is ventrally oriented. The shape of the preserved inner layer of the tympanic bulla suggests that the anteromedial portion of the ventral surface is transversely convex, that its anterolateral corner is inflated and that the ventral keel of the outer posterior prominence faces ventromedially. In lateral view, the conical process of the bulla is dorsally convex.

*Parietal*. In anterior view, the parietals display a subtriangular-shape, expanding laterally. The parietals are well preserved and contact the supraoccipital posteriorly, the squamosals ventrally, the alisphenoids anteroventrally, and the frontals and maxillae anteriorly. The parietal is widely exposed on the cranial vertex. The anteriormost point of the parietal is located anterior to the posterior edge of the ascending process of the maxilla. The fronto-parietal suture is irregular. In lateral view, the parietal is longer than high.

*Alisphenoid*. The shape of the alisphenoid is roughly circular, in lateral view. The alisphenoids contact the squamosals posteriorly, the parietals dorsally, the palatines anteroventrally, and the frontals anterodorsally. In the lateral surface, there is a horizontal crease between the alisphenoid and frontal. The alisphenoid is exposed on the temporal skull wall.

*Supraoccipital and exoccipital*. The posterior region of the supraoccipital and exoccipitals is covered by sediment and several postcranial elements, namely: the axis, three incomplete cervical vertebrae, two ribs and one possible fragment of a scapula. The supraoccipital and exoccipital form a fused occiput. The supraoccipital contacts the exoccipitals posteriorly and the parietals anteroventrally. The exoccipitals contact the basioccipital posteriorly, the squamosals laterally, the periotics ventrolaterally, and the supraoccipital anteriorly. The nuchal crest is pronounced and projects dorsally. In the anterior portion of the supraoccipital there is a well-marked external occipital crest partially covered by rock matrix at its posterior extension. The occipital condyles are strongly protruding.

### *1*. Phylogeny

According to our phylogenetic analysis the Cetotheriidae clade is supported by the anteromedial portion of the palatines forming a well-developed medial crest (Character 21), the presence of the squamosal cleft (Char. 107)(also present in Balaenopteridae), the presence of an external occipital crest (Char. 109), the parabolic outline of the postglenoid process in posterior view (Char. 118), the presence of a posteroventral flange flooring the facial sulcus on compound posterior process (Char. 184).

**Fig 24:**
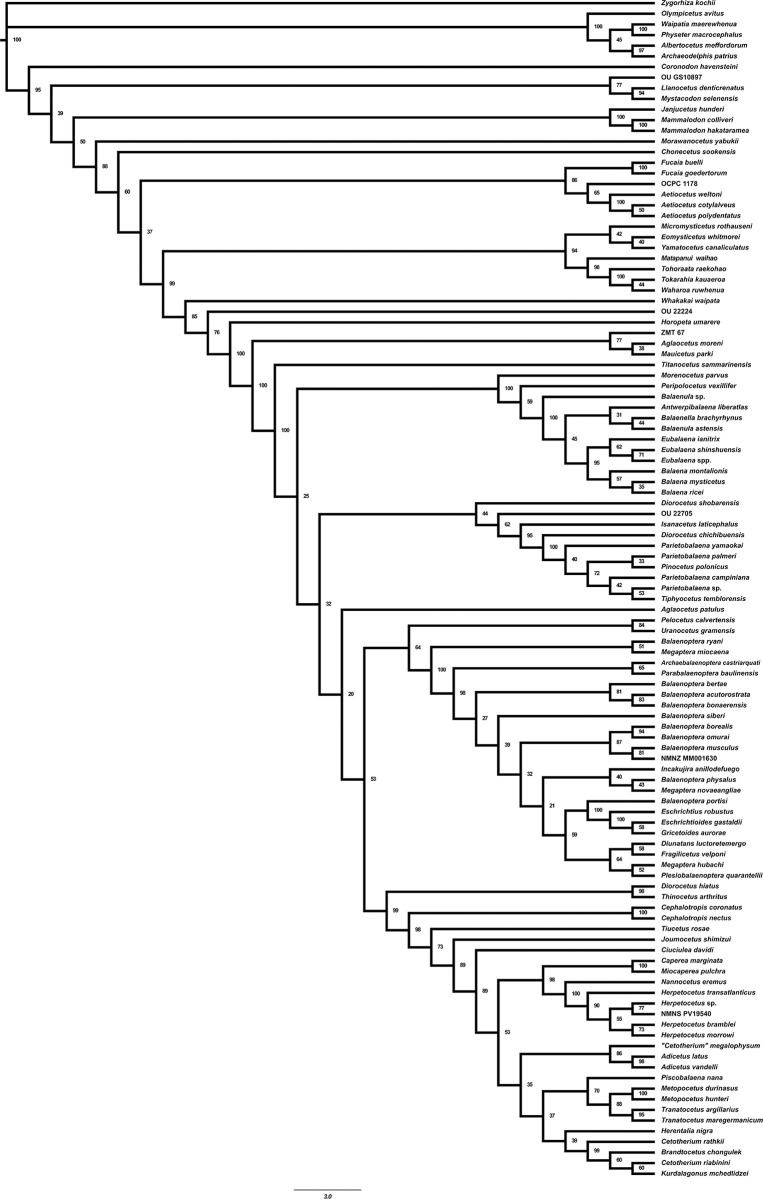
Strict Consensus tree obtained in the phylogenetic analysis using the total evidence matrix. The numbers next to nodes represent the posterior probabilities.

Contrary to Duboys *et al* [36], our analysis places the newly added *Cephalotropis nectus* and *Cephalotropis coronatus* in the most basal position of the Cetotheriidae clade, instead of *Tiucetus rosae* (Fig 24). The characters that are responsible for this shift are the narrow exposure of the vomer along the midline of the rostrum (Char. 17), a round temporal fossa (Char. 78), the transverse position of the posterior apex of the nuchal crest (Char. 91), the facial sulcus centrally located on the ventral surface of the compound posterior process bordered by a distinct ridge posteriorly (Char. 185), the external surface of the compound posterior process of the petrotympanic expanded and firmly integrated into the lateral wall of the skull (Char. 188), and, the dorsal origin of lateral furrow located along the posterior two thirds of the anteroposterior length of the bulla (Char. 194).

There is also a change in the position of *Ciuciulea davidi*, and instead of being included in the clade formed by *Metopocetus durinasus*, *Metopocetus hunteri*, *Tranatocetus argillarius*, *Tranatocetus maregermanicum* and *Piscobalaena nana*, it is now in a more basal position, diverging from *Joumocetus shimizui*.

*Cephalotropis nectus* differs from *Cephalotropis coronatus* (Cope, 1896) by seven characters: the width of the supraorbital process of the frontal relative to the diameter of the orbit (Char. 45), the separation of the posterior ends of the ascending process of the maxilla by premaxillae (Char. 67), the dorsal surface of the nasals (Char. 72), the outline of the fronto-parietal suture (Char. 84), the shape of the compound posterior process (Char. 187), and the shape of the anterior border of the tympanic bullae (Char. 191).

In the consensus tree generated (Fig 24), *Adicetus latus* and *Adicetus vandelli* cluster as sister taxa with a posterior probability of 98%. These two taxa diverge from “*Cetotherium” megalophysum* and form a clade supported by a posterior probability of 86%. These three taxa often cluster together in other phylogenetic analysis [19,20,37], but generally *Adicetus vandelli* or *Adicetus latus* form the outgroup. “*Cetotherium” megalophysum* differs from both *Adicetus vandelli* and *Adicetus latus* by the presence of a squamosal cleft (Char. 107), the outline of the supraoccipital in dorsal view (Char. 110), the presence of a distinct occipital crest on the anterior half of the supraoccipital (Char. 114), and the shape of the choanal margin (Char. 122). *Adicetus latus* differs from both *Adicetus vandelli* and “*Cetotherium” megalophysum* primarily by four characters, namely, the width of the supraorbital process of the frontal relative to the diameter of the orbit (Char. 45), the transverse alignment of the nuchal crest apex with the distal one third of the temporal fossa (Char. 91), the position of the posteriormost point of the paroccipital process relatively to the posterior edge of the occipital condyle (Char. 136), and the lateral surface of the compound posterior process expanded and firmly integrated in the lateral skull wall (Char. 188). *Adicetus latus* also differs from “*Cetotherium” megalophysum* by having a slightly deflected apex of the zygomatic process (Char. 100).

### *2*. Invertebrate species associated with the specimens

There are numerous marine invertebrates associated to the sedimentary matrix of all three skulls, some of them mentioned in previous studies relative to the same extraction locality [57,58].

**Fig 25:**
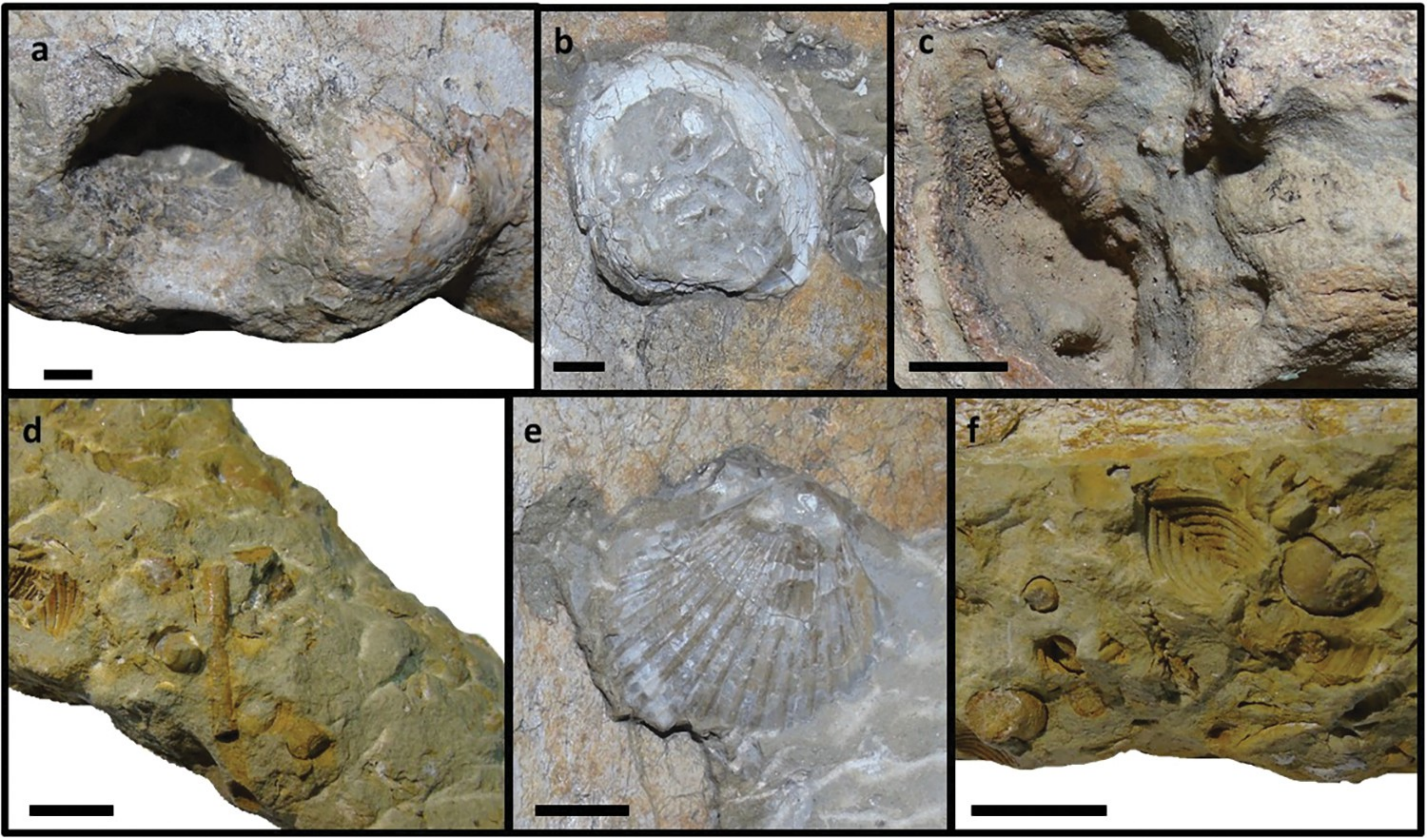
Invertebrate fossils found on the specimens MNHN/UL.C1 and MNHN/UL.C3. a—*Turritella* sp.; b—*Cardium* sp.; c—*Anomia ephippium;* d—*Chlamys macrotis;* e—Veneridae sp.; f–sea urchin spine. Scale– 10mm.

The most abundant species belongs to the genus *Turritella*, being present in the three skulls, as internal and external molds; sizes are ranging from 20mm to 50mm long to 5mm to 10mm wide (Fig 25C). Another fossil, also present in the three skulls as internal and external molds, belongs to the *Cardium* genus (Fig 25F). In MNHN/UL.C1 and MNHN/UL.C3 two specimens of *Anomia* are present on each skull, possibly *Anomia ephippium* (Fig 25A). In MNHN/UL.C1, there is one specimen of *Chlamys macrotis* (Fig 25E) on the posterior portion of the left maxilla and one internal mold of a Veneridae sp. between the left maxilla and frontal (Fig 25B). In MNHN/UL.C3, there is a cylindrical structure, in the loose fragment of the right maxilla, that seems to be a sea urchin spine (Fig 25D).

## Discussion

### *1*. Taxonomy

Since the discovery of the Vandelli specimens there has been a disagreement regarding their taxonomic status mainly for two reasons: some descriptions were based on illustrations or photos and the lack of a robust phylogenetic analysis. Kellogg [31] makes the first taxonomic description of these specimens simply by analysing photos and measurements provided by Sousa Torres, and proposed two new species, *Cephalotropis nectus* and *Aulocetus latus*. Several decades later, Mocho and Póvoas [32] had direct access to the type specimens and noted that Kellogg misinterpreted the position and shape of some bones probably because he did not have access to photos and details regarding the ventral view of the specimens. Kellogg probably inferred some anatomical details from posterior illustrations from other authors made when the skulls were even more underprepared (they still need further preparation). Nevertheless, according to our results, *Cephalotropis nectus* does in fact represent a valid species but the diagnosis had to be corrected and expanded.

In more recent years, various authors suggested that both MNHN/UL.C1 and MNHN/UL.C2 needed to be redescribed [5,6,12,13,16,33]. For example, the specimen MNHN/UL.C3 has been referred "provisionally" as *“Aulocetus”* [31]. However, *Aulocetus* should be considered invalid since this genus shared the same type species with *Cetotheriopsis*, *Cetotheriopsis lintianus* (formerly known as *Balaenodon lintianus*) [33,59]. In fact, more recently, *Aulocetus* was considered as a junior synonym of *Cetotheriopsis* (due to precedence) [33,59,60]. As a result, all specimens attributed to *“Aulocetus”* must be classified as *Cetotheriopsis* or attributed to different genera [33], however, that goes beyond the scope of the present work.

As for MNHN/UL.C1, many phylogenetic studies position this specimen far from the *Metopocetus* clade [12,15,16,19,20,37] and Marx et al. [16] pointed several features that emphasize the differences between MNHN/UL.C1 and other *Metopocetus* species, clarifying the need of a new taxonomic classification of MNHN/UL.C1. The missing Vandelli skull (here coded as MNHNL.UL.C0 and illustrated in Vandelli, 1831, Plate IV, Fig 1 to Fig 4 [24]; Fig 2) was also ascribed to *Metopocetus vandelli* and all authors that mentioned MNHNL.UL.C0 have assumed this specimen to be the same species as MNHN/UL.C1 [31,55]. Nevertheless, by analysing the illustration made by Vandelli ([24], Plate IV; Fig 2) there are some major differences from MNHN/UL.C1 ([24], Plate IV, Fig 5 to Fig 8; Fig 2), namely: i) the ascending processes of the maxillae and the lateral profile of the parietals in the missing skull are triangular, ii) the supraoccipital is continuously concave in the missing skull, while in MNHN/UL.C1 it is only dorsally concave in the anterior portion, and iii) the occipital condyles are more protruding in the missing skull. Curiously, Vandelli [24], attributed the same genus to MNHN/UL.C1 and MNHN/UL.C2 and a different one to MNHNL.UL.C0. This strongly suggests that MNHNL.UL.C0 could be a different species yet to be described but, in the absence of the specimen it is not prudent to propose a diagnosis and respective taxonomic classification.

### *2*. Two species one genus

It is clear that *Adicetus vandelli* and *Adicetus latus* share several features that indicate that they are closely related. There are only three characters on the matrix that distinguish *Adicetus vandelli* from *Adicetus latus*, but, at the same time, there are several other conspicuous characteristics differing these two specimens.

Some differences could be explained by erosion or intraspecific variations related to ontogeny or sexual dimorphism, for example size differences [21,61,62]. In our opinion these differences should not be used as diagnostic features, namely: the slight difference of the ascending processes of the maxilla being more rounded in *Adicetus vandelli*; more slender and gracile bones in *Adicetus latus* (postglenoid process and exoccipitals); size and position of the dorsal infraorbital foramen (smaller and more distal in *Adicetus vandelli*); dorsal “corner” on the supraoccipital (sharper in *Adicetus vandelli*); and the orientation of the acoustic meatus (more posteriorly oriented in *Adicetus vandelli*).

One interesting detail in *Adicetus latus* holotype is the bilobated cavity situated on the posterior region of the supraoccipital that could be associated with a fontanel, which would indicate that this specimen is less than a year old [61], however, there are no suture lines visible between the supraoccipital and the exoccipitals near the opening. This opening is similar in shape and location to the one on the holotype of *Morenocetus parvus* [63]. Additionally, *Adicetus latus* also presents some adult traits, such as: well fused sutures (including the basioccipital/exoccipital and basioccipital/basisphenoid sutures [61]), sharp nuchal crest and smooth surface of the occipital condyles [63]. There are also other openings in the parietal bones that are not related to fontanels, which suggests that damage originated the supraoccipital opening.

**Fig 26:**
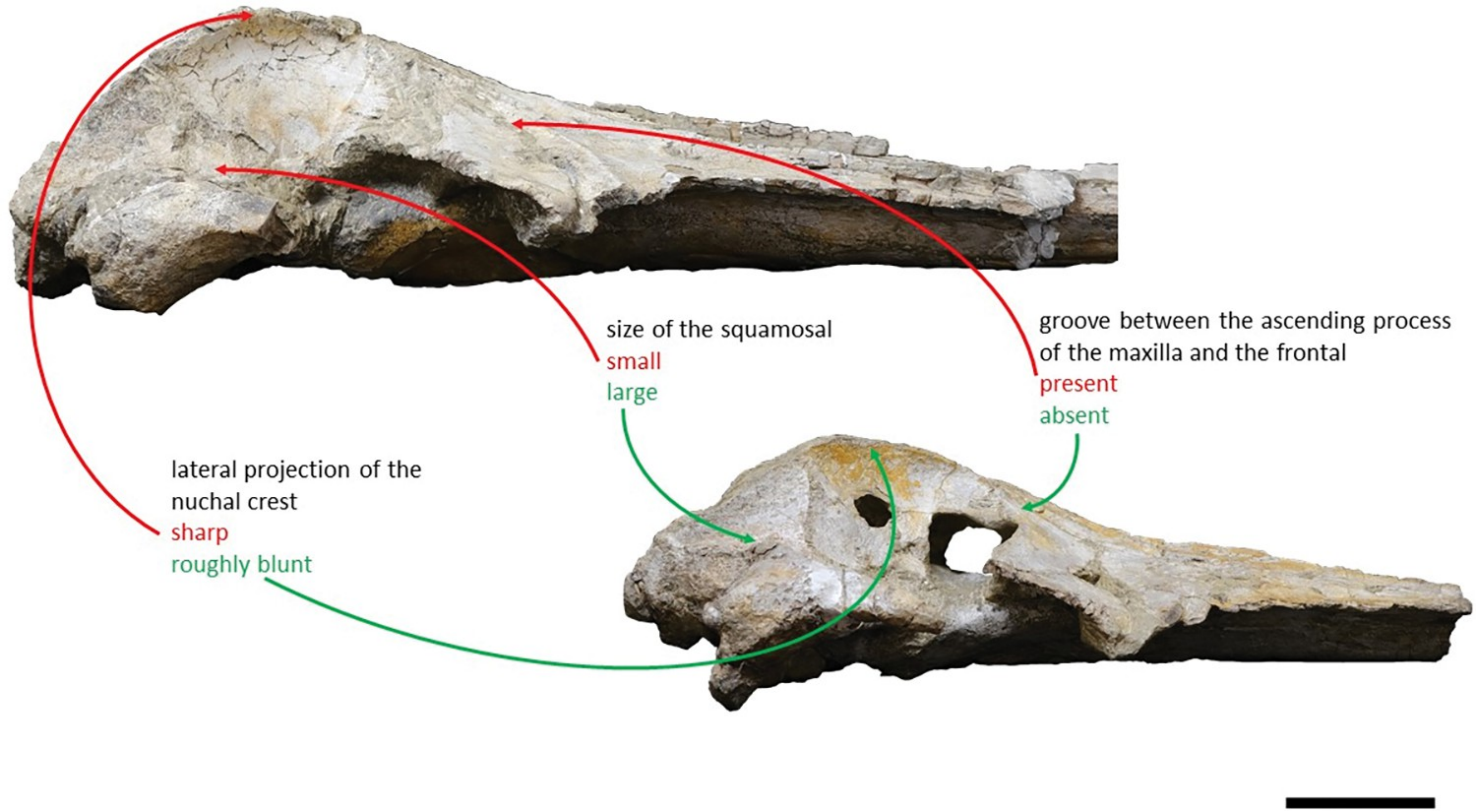
**Differences between *Adicetus vandelli* (MNHN/UL.C1) (above) and *Adicetus latus* (MNHN/UL.C2) (bellow). Skulls in lateral view.** Scale– 100 mm.

**Fig 27:**
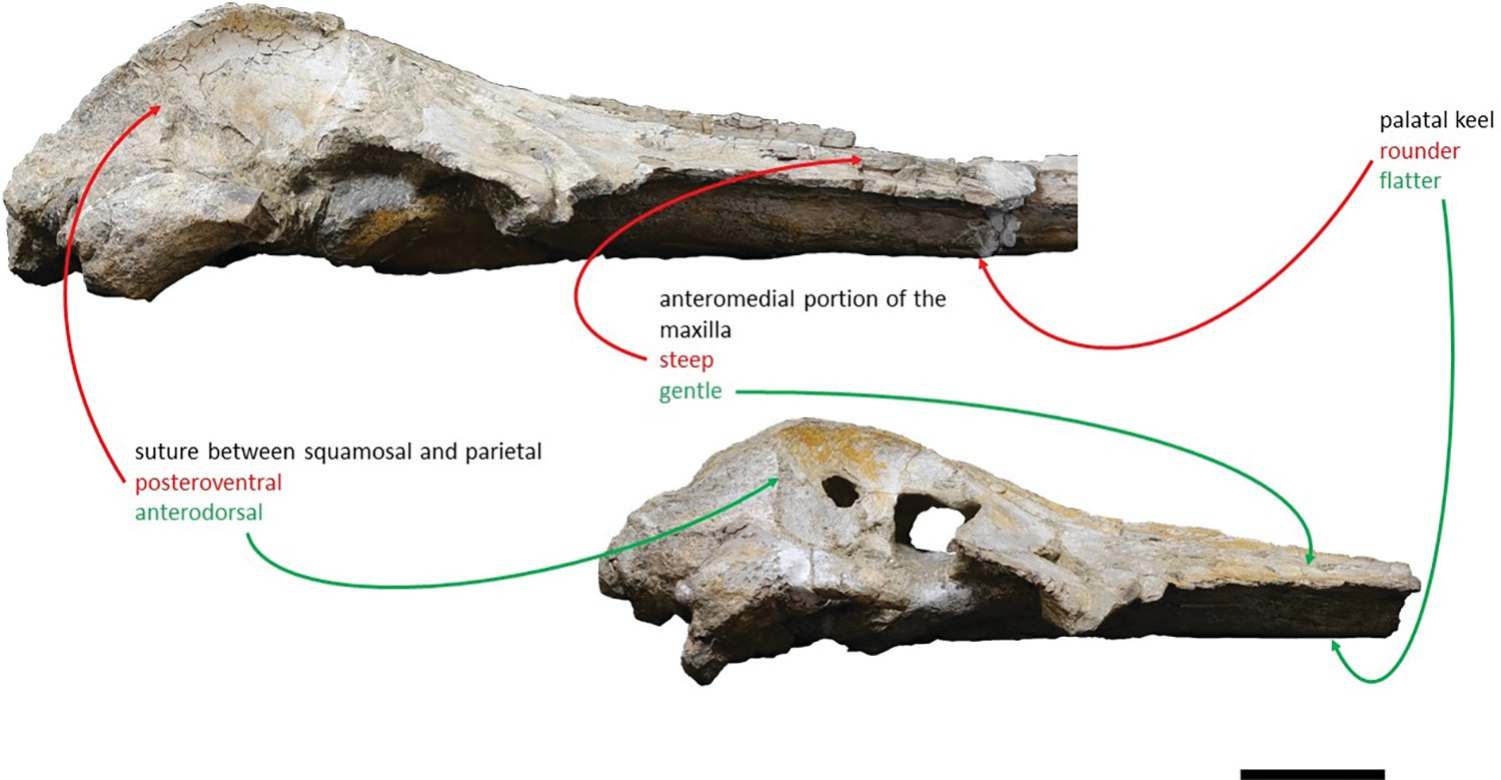
**Differences between *Adicetus vandelli* (MNHN/UL.C1) (above) and *Adicetus latus* (MNHN/UL.C2) (bellow). Skulls in lateral view.** Scale– 100 mm.

**Fig 28:**
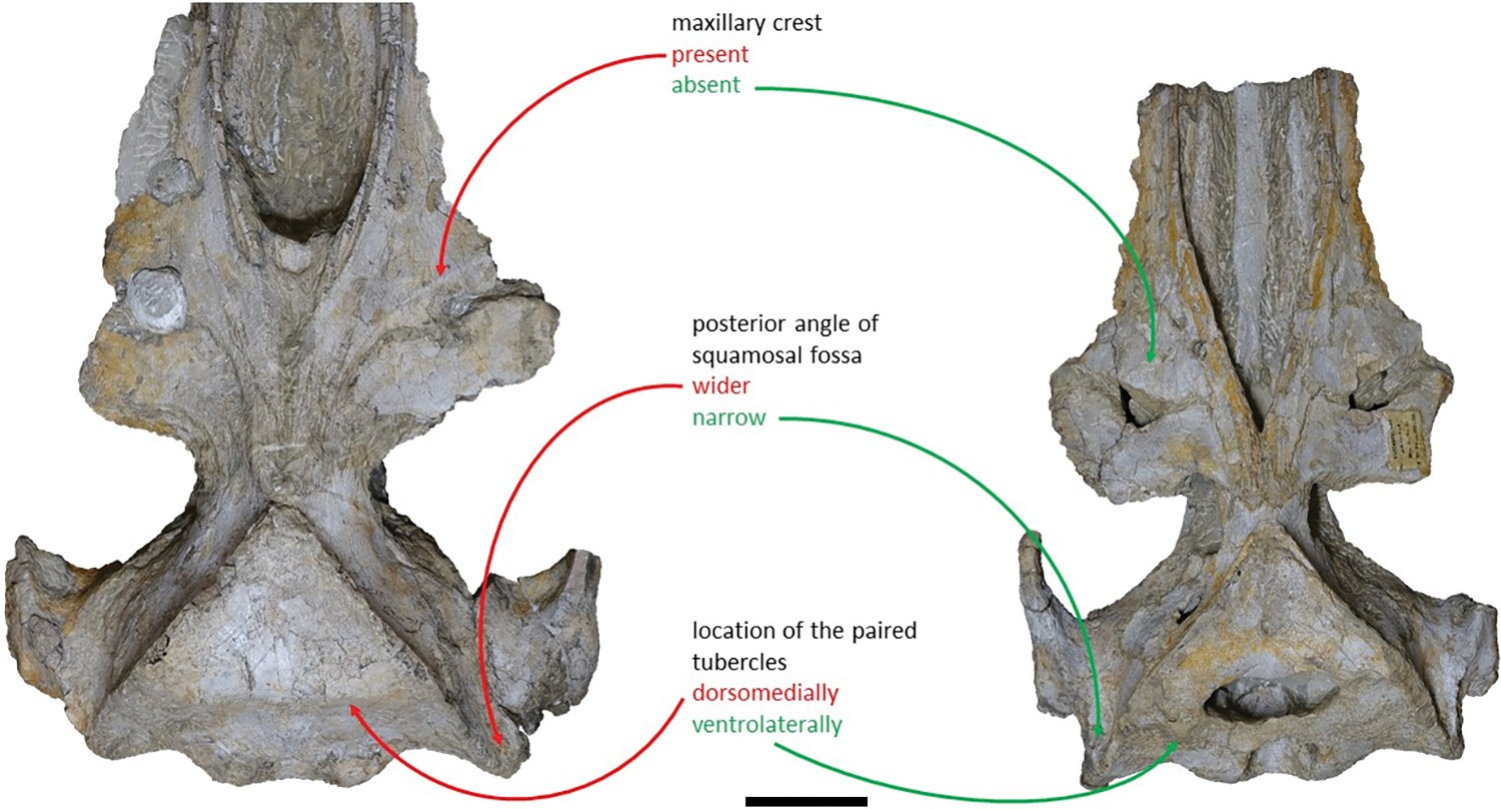
**Differences between *Adicetus vandelli* (MNHN/UL.C1) (left) and *Adicetus latus* (MNHN/UL.C2) (right). Skulls in dorsal view.** Scale– 100 mm.

Nevertheless, there are clear differences that are here considered as interspecific, namely: the absolute size of the squamosal (bigger on *Adicetus latus* although the skull is smaller than *Adicetus vandelli*) (Fig 26); groove between the ascending process of the maxilla and the frontal (present in *Adicetus vandelli*) (Fig 26); the lateral projection of the nuchal crest (more accentuated in *Adicetus vandelli*) (Fig 26); the palatal keel (flatter in *Adicetus latus*) (Fig 27); position of the suture between squamosal and parietal (more anterodorsal on *Adicetus latus*) (Fig 27); anteromedial portion of the maxilla adjacent to the premaxilla (steeper in *Adicetus vandelli*, making an almost 90° angle) (Fig 27); the relative position of the paired tubercles (more lateral and ventral in *Adicetus latus*) (Fig 28); excavation of the squamosal fossa with its posterior angle shallower and wider, respectively, in *Adicetus vandelli* (Fig 28); and the presence of a well-developed crest in the anterolateral portion of the maxilla in *Adicetus vandelli* (Fig 28).

We concluded that there are in fact conspicuous differences that justify the separation of *Adicetus latus* and *Adicetus vandelli* into two different species. If new empirical studies on mysticetes intraspecific differences can show that some (or all) of the interspecific differences pointed above should be interpreted as intraspecific variation, then these two specimens may be reunited in one single species preserving the specific name “*vandelli*”, since it was the first to be assigned.

### *3*. Phylogeny

The phylogeny of the Cetotheriidae family as been very debated along the last decades. It is observed various shifts in the Cetotheriidae clade when a new species is added to the analysis, which suggests that this debate is far from over.

The major difference between the phylogenetic results of the present study and the results from Duboys *et al*. (2020) are the most basal species. Since the methodology was the same in both studies, this suggests that the addition of *Cephalotropis nectus* to the phylogenetic analysis caused the shift of the *Cephalotropis* clade to the most basal position of the Cetotheriidae family.

Our results also emphasize the affinities between *Adicetus latus*, *Adicetus vandelli* and *“Cetotherium” megalophysum*, suggesting that these taxa could in fact belong to the same genus, as Marx et al. [20] have suggested. However, the last description of “*Cetotherium” megalophysum* was very incomplete and made a long time ago [64]. As a result, it is urgent to revise the anatomical description of *Cetotherium*, particularly, to clarify its phylogenetic relationships. Also, because there are significant differences with *Cephalotropis coronatus* the attribution of MNHN/UL.C3 to *Cephalotropis nectus* [31], although based on insufficient and some incorrect data, is here supported as valid, against the hypothesis of Mocho and Póvoas, in 2010 [32].

### *4*. Paleoecology

According to the ecological characteristics of the fossil invertebrates found in association, it is possible to infer the paleohabitat where the three cetacean specimens fossilized.

The genus *Turritella* generally occurs in depths less than 100 meters, in waters with normal marine salinity and temperatures varying from 15-20°C, but some species can live in habitats with 0–1500 m deep, estuarine type salinity and 2-27°C [65]. *Cardium* species occurs in waters with variable temperatures, from 0-25°C, and with estuarine and marine type salinities [66]. *Anomia ephippium* occurs between the intertidal zone and 30 m deep [67]. Because the portion of a sea urchin spine preserved does not give enough information, we could not identify the species and since the echinoids can occupy habitats with variable depth and temperatures [68], the only information that can be retrieved from this fossil is that these groups occur in waters with marine type salinity.

Combining all these data gives us a paleohabitat characterized by shallow waters up to 30 m deep, with typical sea salinity, temperatures between 0-20°C and little wave action.

Mocho *et al* [57] also analysed the paleoecology of the region of Foz do Rego deposit (about 10 km north of the fossil cliff of the Adiça) through the identification of 39 species of marine invertebrates (including *Chlamys macrotis* and *Anomia ephippium*), concluding that the region was characterized by waters with typical marine salinity and temperatures relatively higher than the ones presently registered in that region.

It is important to point out that *Cephalotropis coronatus* described by Cope was collected in Maryland, United States of America [56] while *Cephalotropis nectus* was collected in Adiça, Portugal, meaning that this genus occurred in both coastlines of the Atlantic Ocean. In addition, based on our phylogenetic analysis, *Cephalotropis nectus* and *Cephalotropis coronatus* belong to the same genus and they form the most basal clade of the Cetotheriidae family.

## Concluding remarks

In the present study we retrieved a new phylogenetic tree for the Cetotheriidae and conclude that the genus names previously attributed to both *Adicetus vandelli* (*Metopocetus*) and *Adicetus latus* (*Aulocetus*) are not valid, and therefore should be renamed. In fact, *Adicetus vandelli* and *Adicetus latus* represent two distinct species belonging to the same genus and we retained their current species names, "*vandelli*" and "*latus*", respectively. MNHN/UL.C3 presents conspicuous differences from *Cephalotropis coronatus* and for that fact, should be considered a valid taxon: *Cephalotropis nectus* (Kellogg, 1941) [31]. With the addition of MNHN/UL.C3 to the phylogenetic analysis, *Cephalotropis coronatus* and *Cephalotropis nectus* become the most basal species of the Cetotheriidae family.

This study brings new pieces to the Cetotheriidae puzzle and clarify the relationships of the species within this clade. In addition, we included a comprehensive portfolio with illustrations of the 201 characters coded (out of 278) (see S1 File) which should be extremely useful to future researchers in this field [5,11–13,16,18,20,34,54,59,62,69–83].

We also concluded that the paleohabitat where the three specimens were preserved was characterized by shallow waters up to 30 m deep, with typical sea salinity, temperatures between 0-20°C and low wave impact.

Finally, it is also important to emphasize the urgent need of preparation of the specimens here studied, in order to preserve the first fossil vertebrates ever published in Portugal, that became, with the present work and probably for a short period, the most recent new species in our country.

## Supporting information

S1 File Portfolio with the illustration of the morphological characters coded. (PDF)

S2 File Matrix of morphological characters (adapted from Duboys *et al*., 2020) [36]. (PDF)
