# Correction: Redescription of three fossil baleen whale skulls from the Miocene of Portugal reveals new cetotheriid phylogenetic insights

**DOI:** 10.1371/journal.pone.0308735

**Published:** 2024-08-08

**Authors:** Rodrigo Figueiredo, Mark Bosselaers, Liliana Póvoas, Rui Castanhinha

The word “*Cephalotropis*” is misspelled in the line labeled “Type species” in the subsection labeled “1. Cephalotropis nectus” within the Results section. The correct line is: “**Type species.** *Cephalotropis nectus* Kellogg, 1941”
