# Supplementary material for: Redescription of three fossil baleen whale skulls from the Miocene of Portugal reveals new cetotheriid phylogenetic insights
Source: PLoS One. 2024 Mar 13;19(3):e0298658. doi: 10.1371/journal.pone.0298658 (PMC10936793; doi:10.1371/journal.pone.0298658)
Supplement: S1 File — (PDF) [file pone.0298658.s001.pdf]

[1] 'Length of rostral portion of maxilla anterior to antorbital notch'

(0)'less than bizygomatic width'

(1)'equal to or greater than bizygomatic width'

(2)'more than one and a half times the bizygomatic width'

(0)

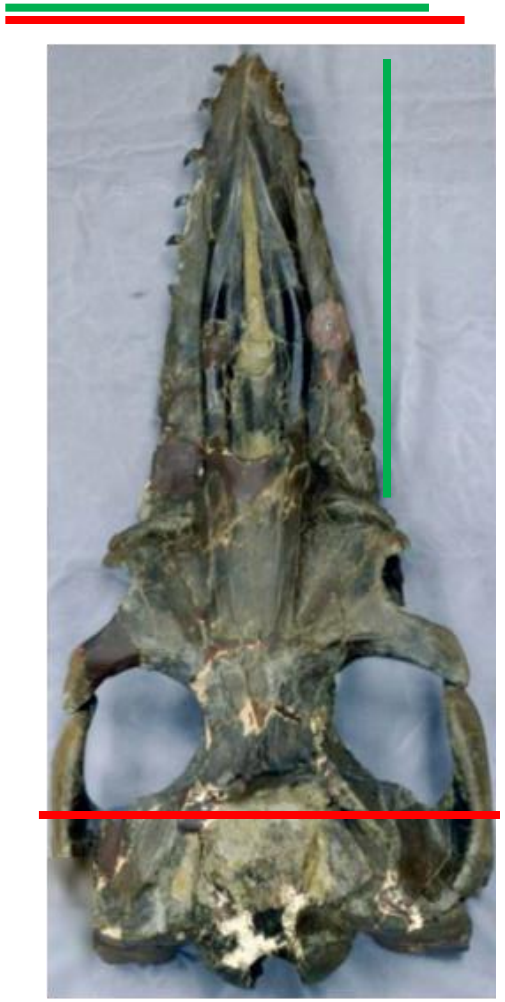

Aetiocetus weltoni

Copyright holder: Felix G. Marx/ University of California Museum of Paleontology, Berkeley, USA

(1)

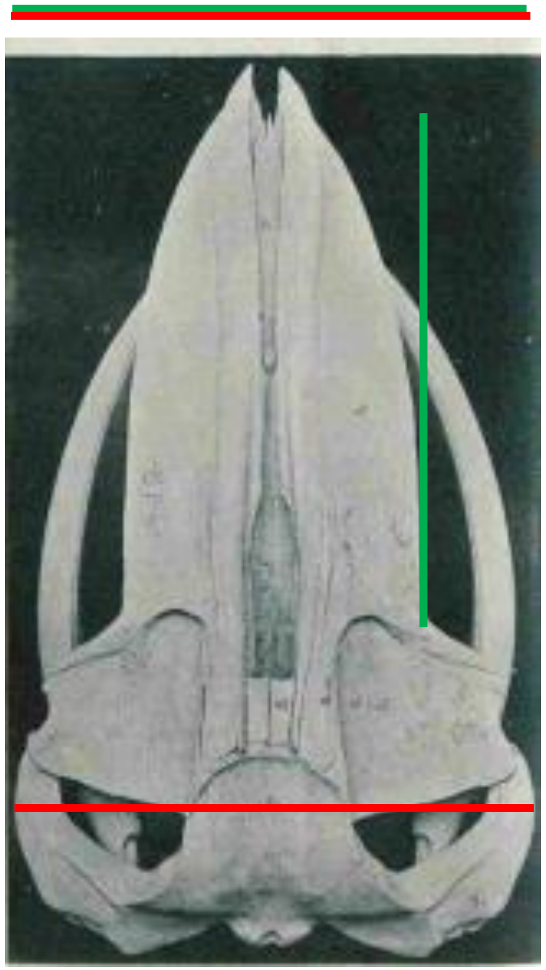

Balaenoptera musculus

Adapted from: "Catalogue of marine mammals of the Mammalogical collection of the Museo de La Plata, Argentina", Olivares et al, 2016. (original plate II of Lahille (1898))

(2)

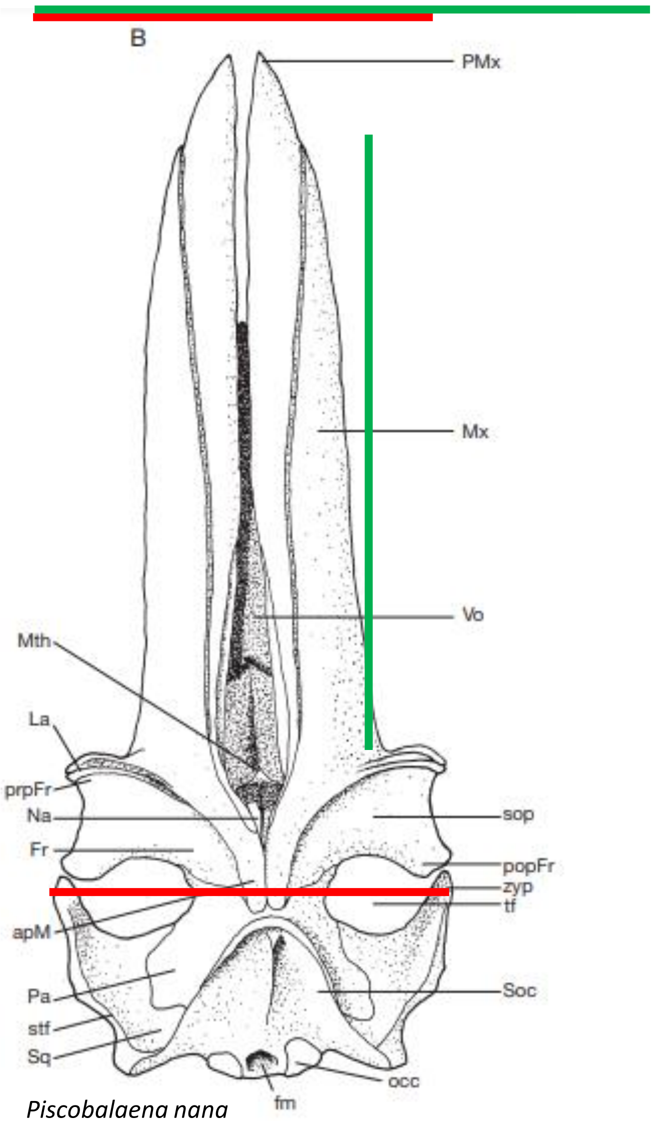

Piscobalaena nana

Adapted from: "The anatomy and relationships of *Piscobalaena nana* (Cetacea, Mysticeti), a Cetotheriidae s.s. from the early Pliocene of Peru", Bouetel and Muizon, 2006. *Geodiversitas* 28.2 (2006): 319-395.

[2] 'Portion of rostrum anterior to nasals in lateral view'

(0)'below the level of the supraoccipital'

(1)'raised to or above the supraoccipital'

(0)

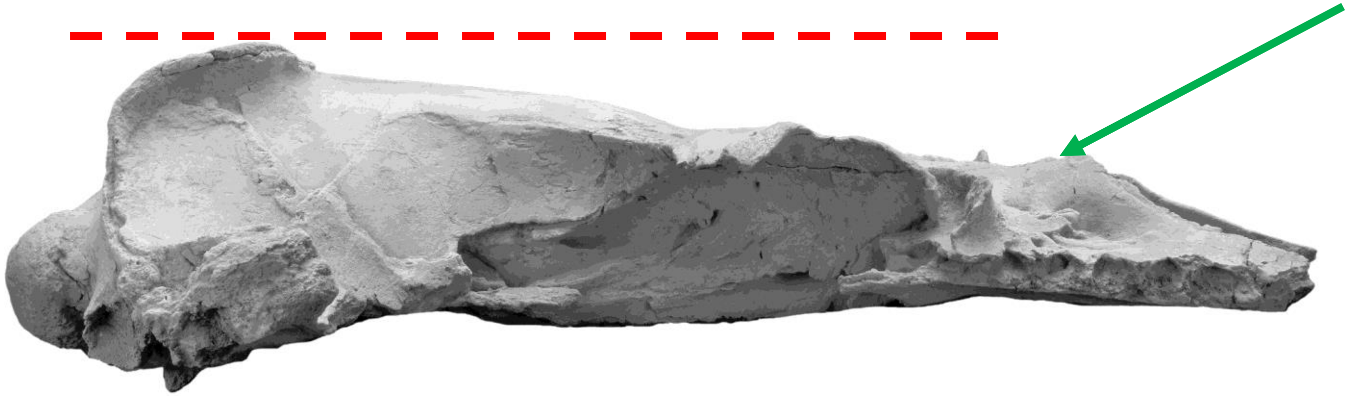

Mammalodon colliveri

(1)

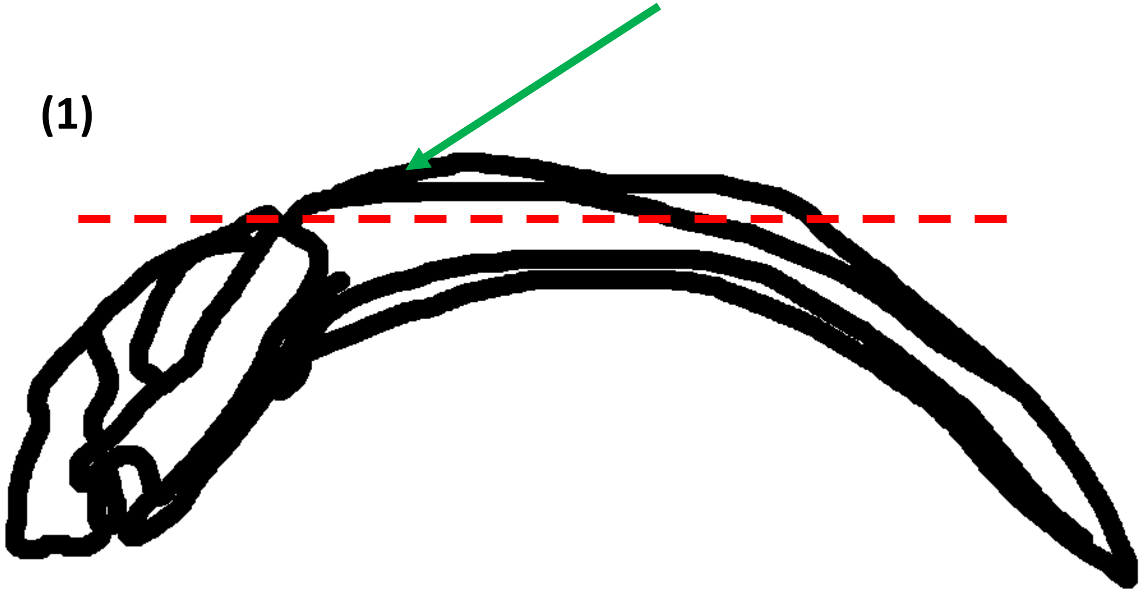

Example based on *Balaena mysticetus*

Copyright holder: Erich M. G. Fitzgerald/ Museums Victoria, Melbourne, Australia

[3] 'Maxilla in cross section'

(0) 'robust'

(1) 'markedly flattened'

(0)

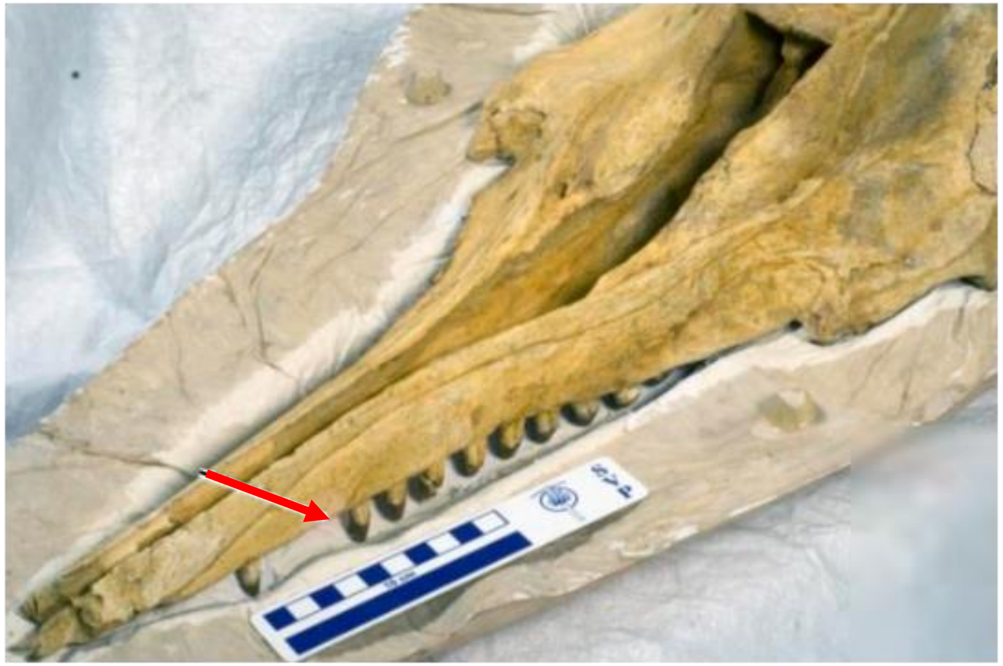

Waipatia maerewhenua

Copyright holder: Felix G. Marx/ University of Otago Museum of Geology, Dunedin, New Zealand

(1)

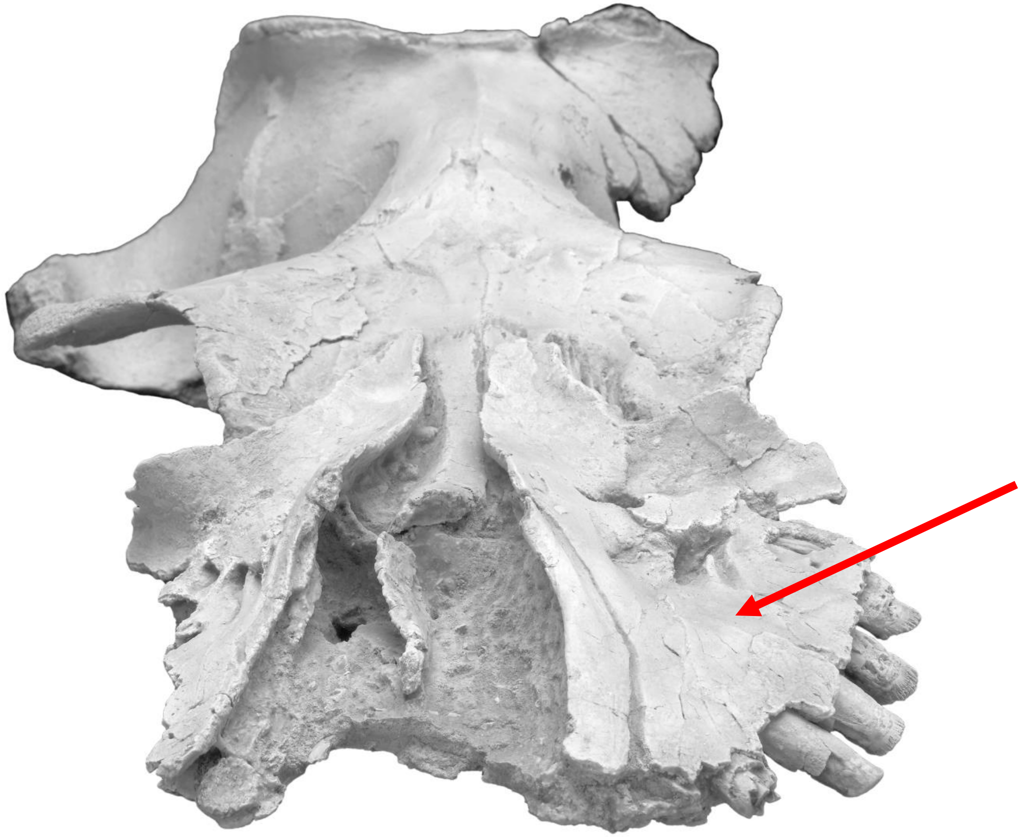

Mammalodon colliveri

Copyright holder: Erich M. G. Fitzgerald/ Museums Victoria, Melbourne, Australia

[4] 'Lateral border of maxilla anterior to antorbital notch or homologous point on rostrum in dorsal view'

(0) 'concave'

(1) 'straight or slightly convex'

(2) 'broadly convex'

(0)

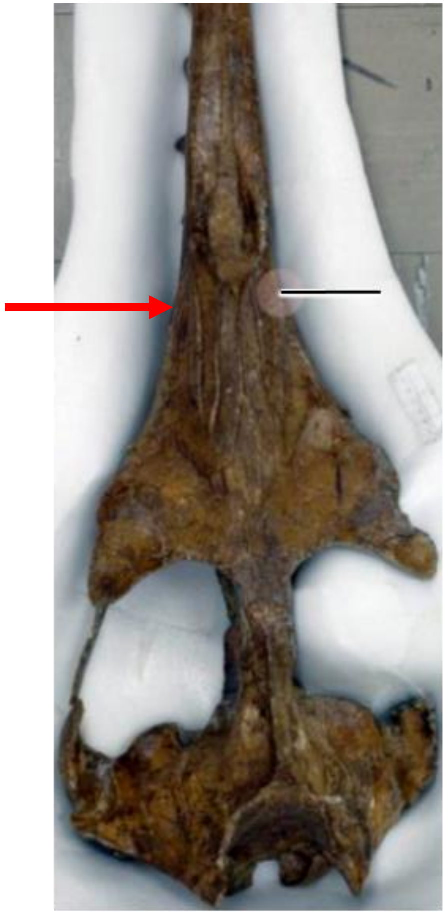

Zygorhiza kochii

Copyright holder: Felix G. Marx/ United States National Museum of Natural History, Washington DC, USA

(1)

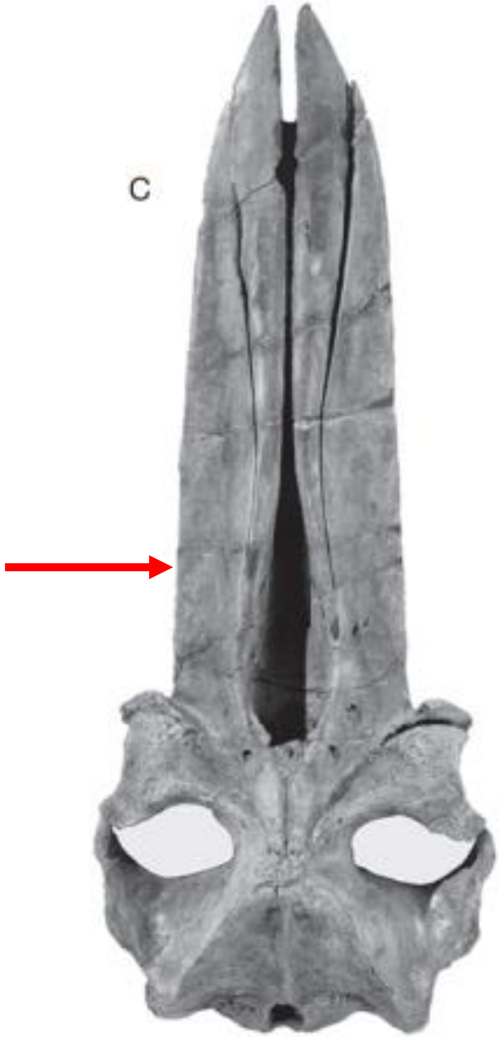

Piscobalaena nana

Adapted from: “The anatomy and relationships of *Piscobalaena nana* (Cetacea, Mysticeti), a Cetotheriidae s.s. from the early Pliocene of Peru”, Bouetel and Muizon, 2006. *Geodiversitas* 28.2 (2006): 319-395.

(2)

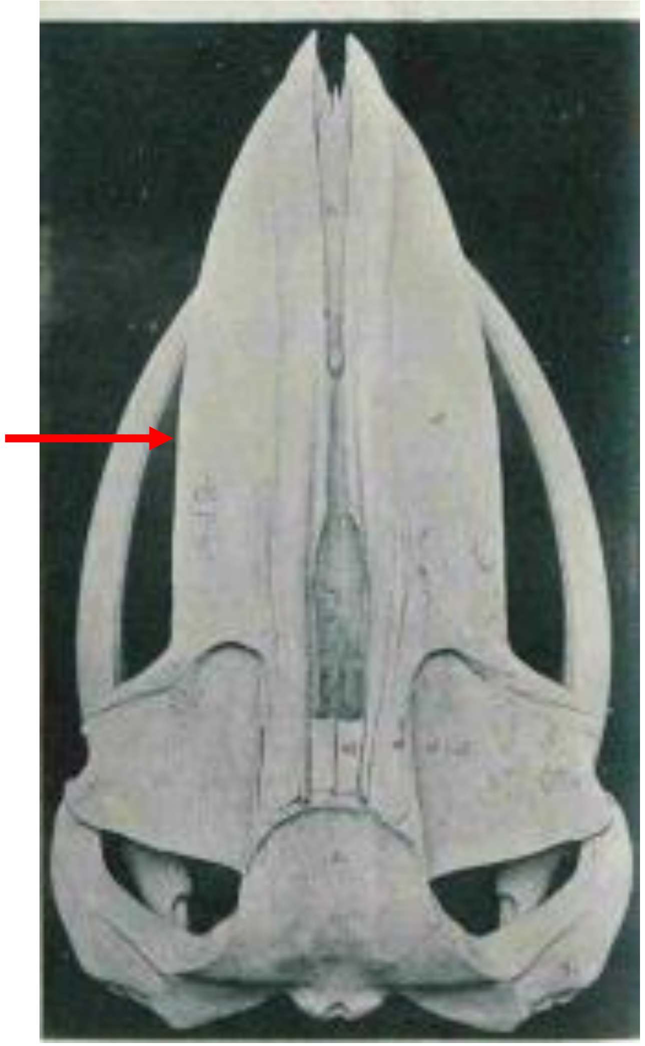

Balaenoptera musculus

Adapted from: “Catalogue of marine mammals of the Mammalogical collection of the Museo de La Plata, Argentina”, Olivares et al, 2016. (original plate II of Lahille (1898))

[5] 'Transverse width of maxilla at midpoint'

(0)'distinctly less than that of the premaxilla'

(1)'roughly equal to or up to twice the width of the premaxilla'

(2)'more than twice the width of the premaxilla'

(0)

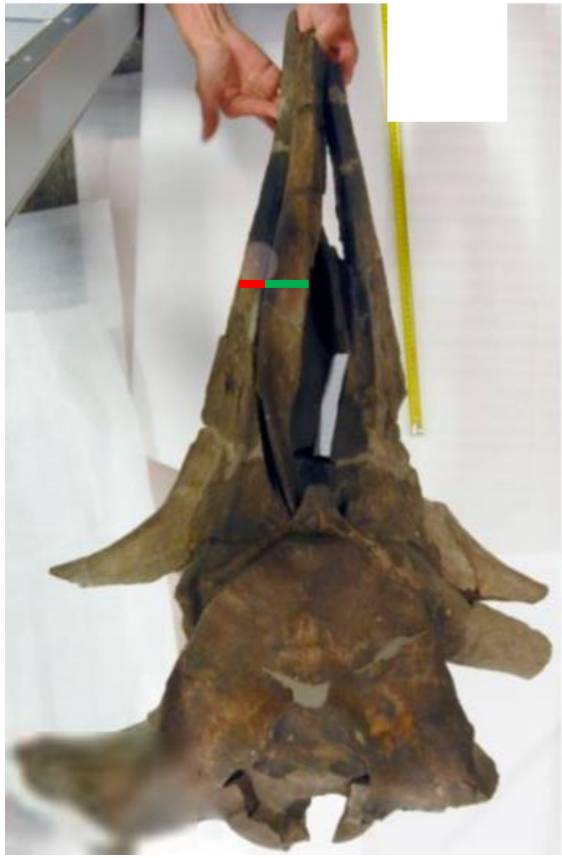

Balaenella brachyrhynus

Copyright holder: Felix G. Marx/Natuurmuseum Brabant, Tilburg, the Netherlands

(1)

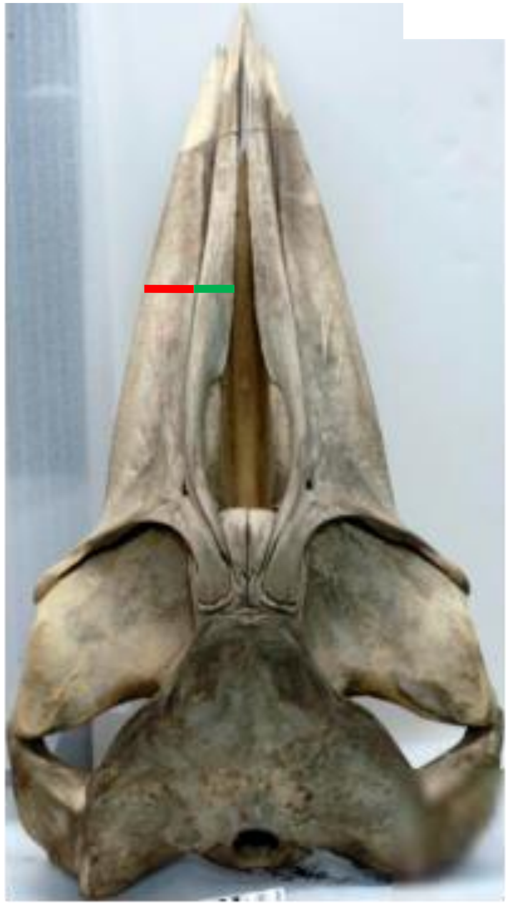

Balaenoptera acutorostrata

Copyright holder: Felix G. Marx/ The Charleston Museum, Charleston, South Carolina, USA

(2)

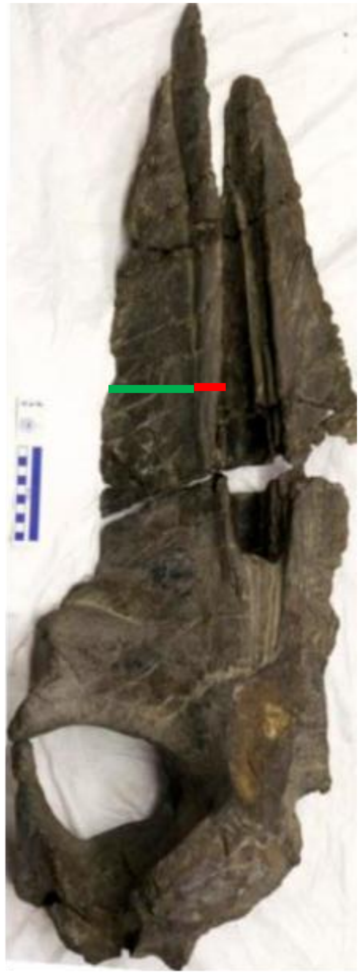

Isanacetus laticephalus

Copyright holder: Felix G. Marx/ Mizunami Fossil Museum, Gifu, Japan

[6] 'Premaxilla in dorsal view'

(0)'widens at anterior end'

(1)'portion anterior to nasal opening narrows or remains the same width anteriorly'

(0)

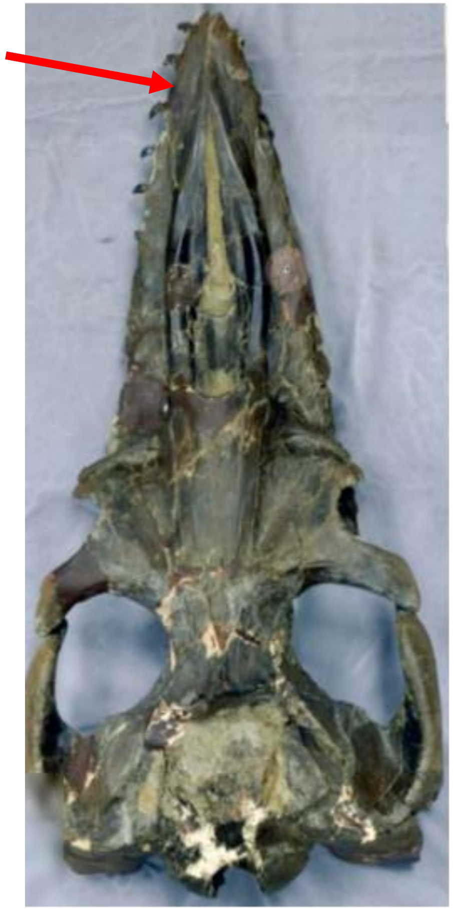

Aetiocetus weltoni

Copyright holder: Felix G. Marx/ University of California Museum of Paleontology, Berkeley, USA

(1)

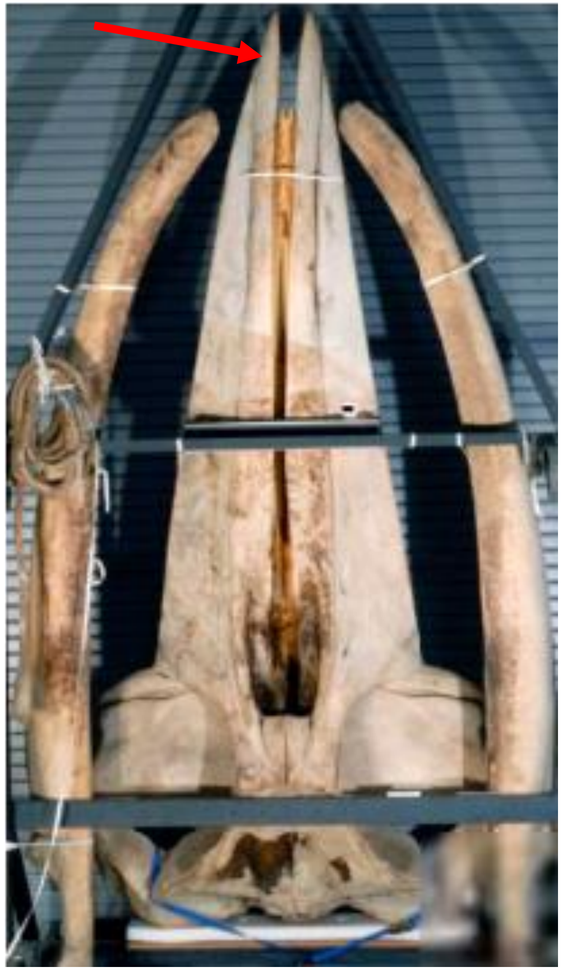

Balaenoptera borealis

Copyright holder: Felix G. Marx/ United States National Museum of Natural History, Washington DC, USA

[7] 'Premaxilla adjacent to and anterior to narial fossa'

(0)'elevated above the maxilla and forming a distinct lateral face'

(1)'continuous or nearly continuous with the maxilla'

(0)

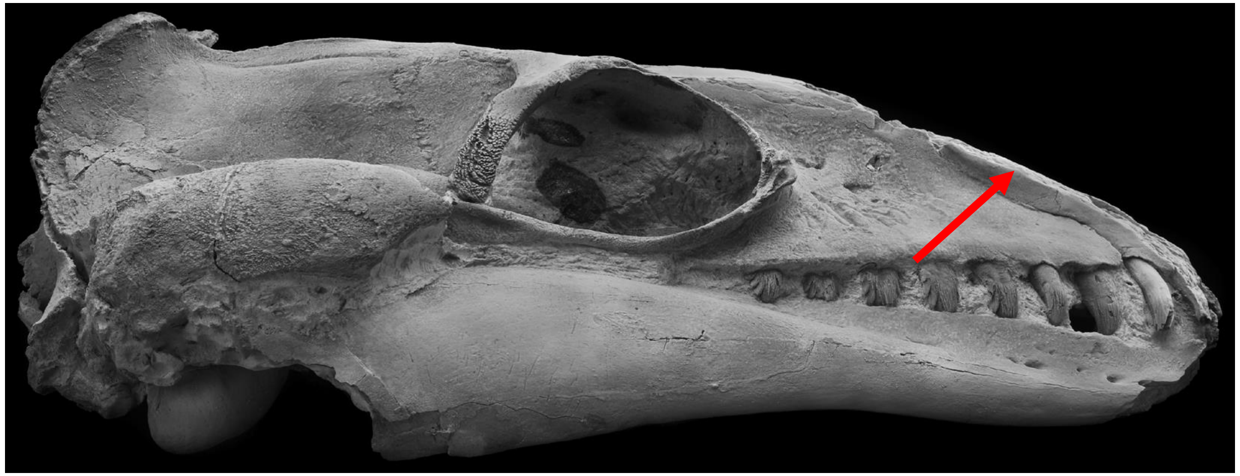

Janjucetus hunderi

(1)

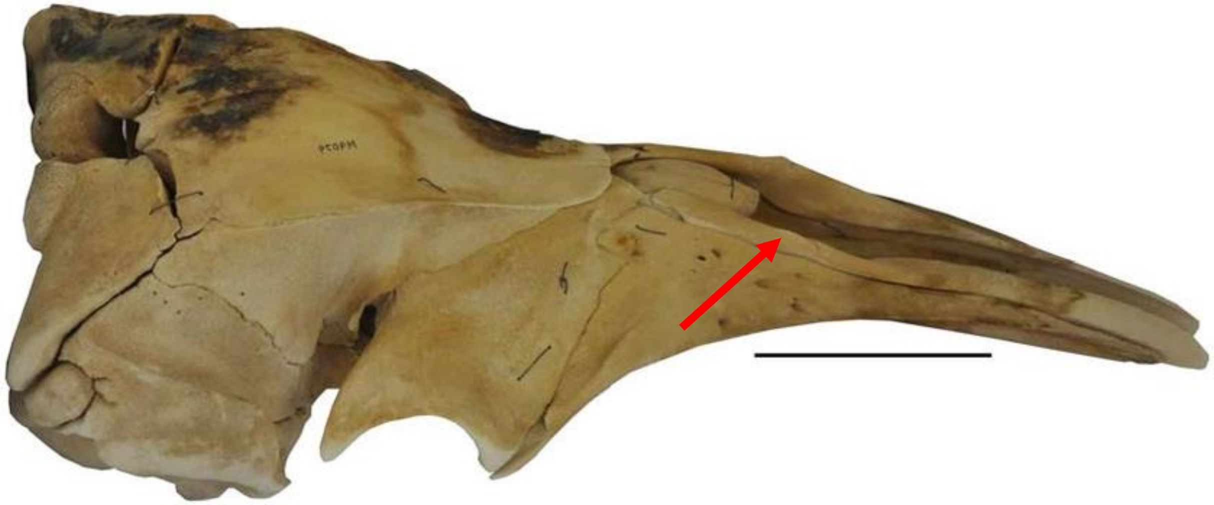

Caperea marginata (mirrored)

Copyright holder: Erich M. G. Fitzgerald/ Museums Victoria, Melbourne, Australia

Adapted from: "Juvenile morphology in baleen whale phylogeny.Tsai, Cheng-Hsiu, and R. Ewan Fordyce, 2014. *Naturwissenschaften* 101.9 : 765-769. "

[8] 'Premaxilla adjacent to and at posterior edge of nasal opening'

(0)'does not clearly overhang maxilla'

(1)'premaxilla overhangs maxilla'

(0)

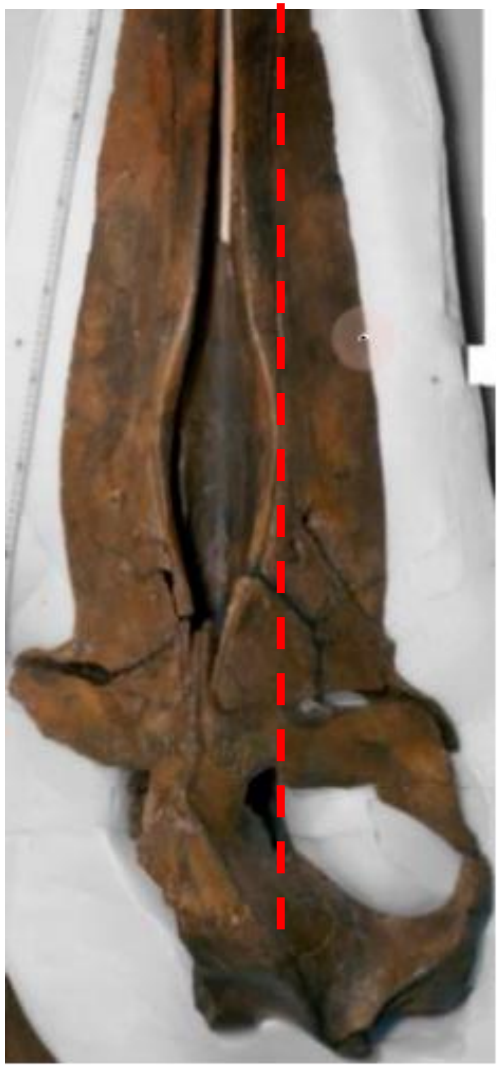

Diorocetus hiatus

Copyright holder: Felix G. Marx/ United States National Museum of Natural History, Washington DC, USA

(1)

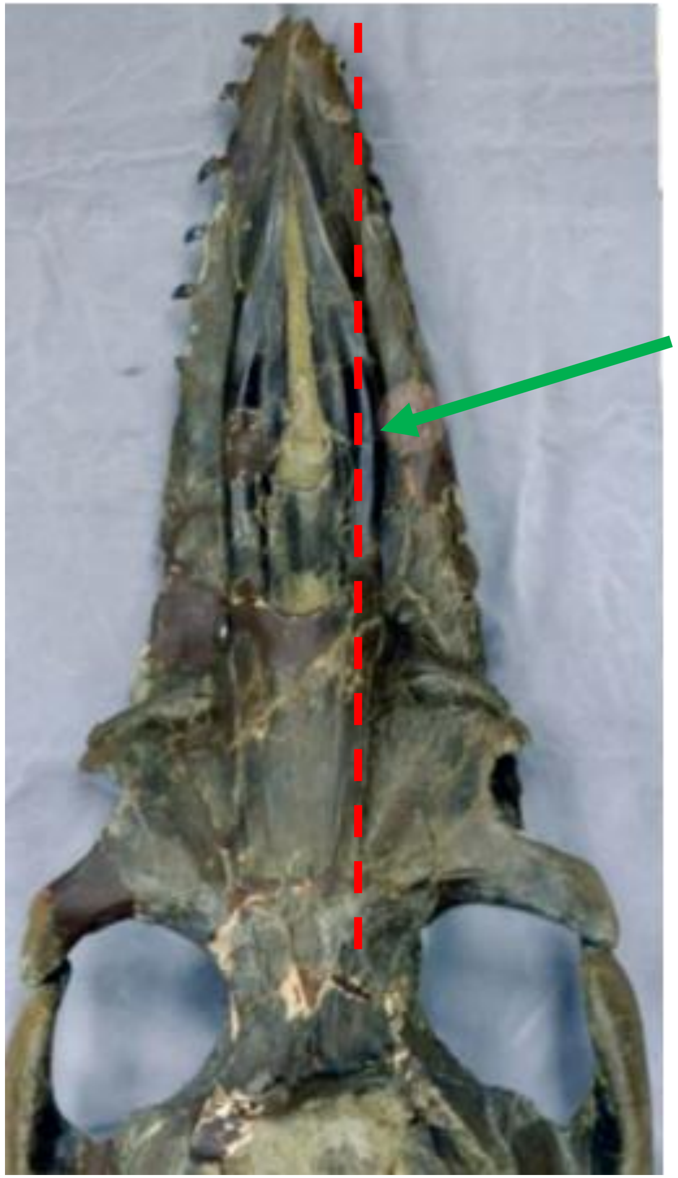

Aetiocetus weltoni

Copyright holder: Felix G. Marx/ University of California Museum of Paleontology, Berkeley, USA

[9] 'Anterior portions of premaxillae'

(0)'firmly contact each other'

(1)'premaxillae are separated or only loosely contact along their entire length'

(0)

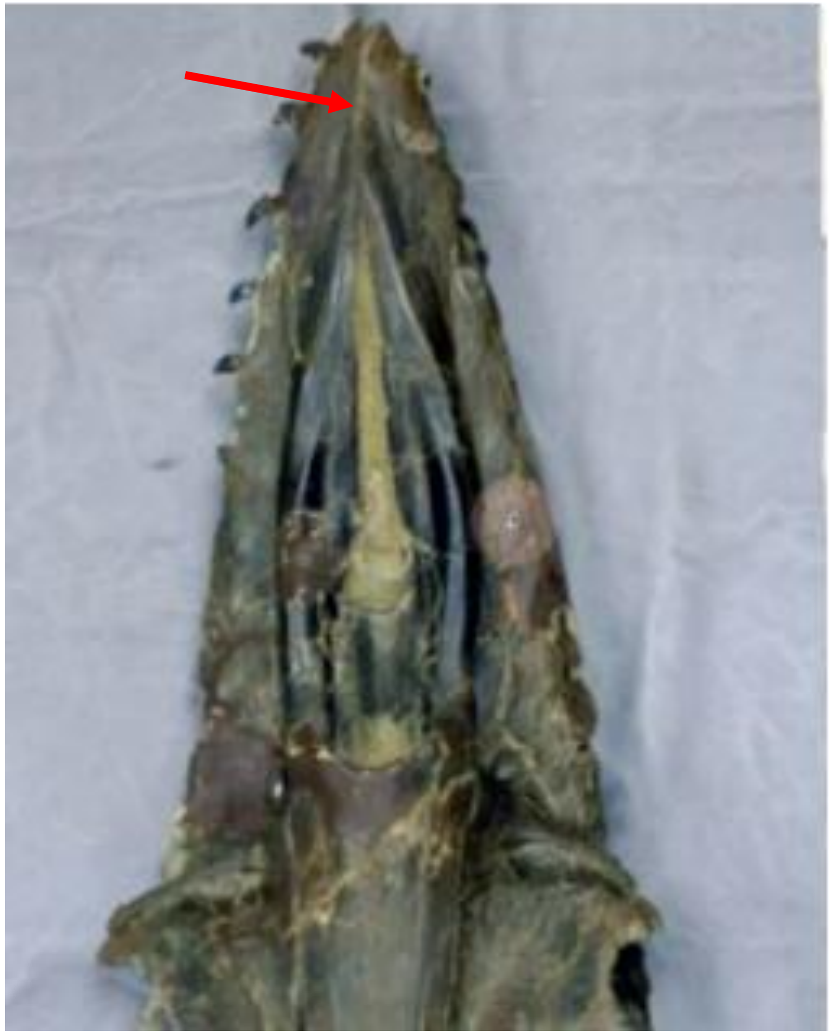

Aetiocetus weltoni

Copyright holder: Felix G. Marx/ University of California Museum of Paleontology, Berkeley, USA

(1)

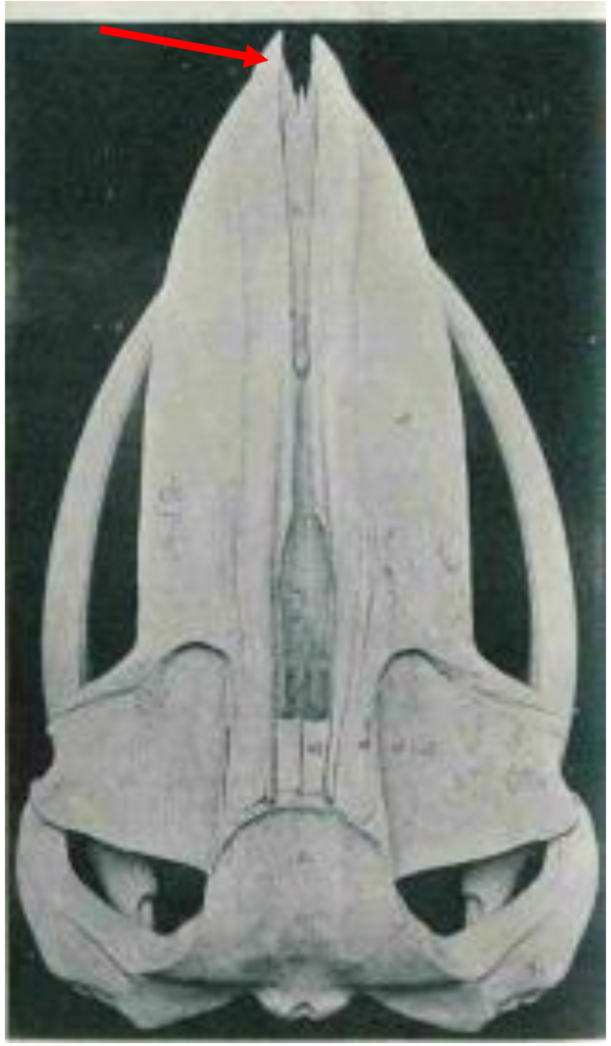

Balaenoptera musculus

Adapted from: “Catalogue of marine mammals of the Mammalogical collection of the Museo de La Plata, Argentina”, Olivares et al, 2016. (original plate II of Lahille (1898))

[10] 'Suture between maxilla and premaxilla on rostrum'

(0)'firmly articulated’

(1)'loose'

(0)

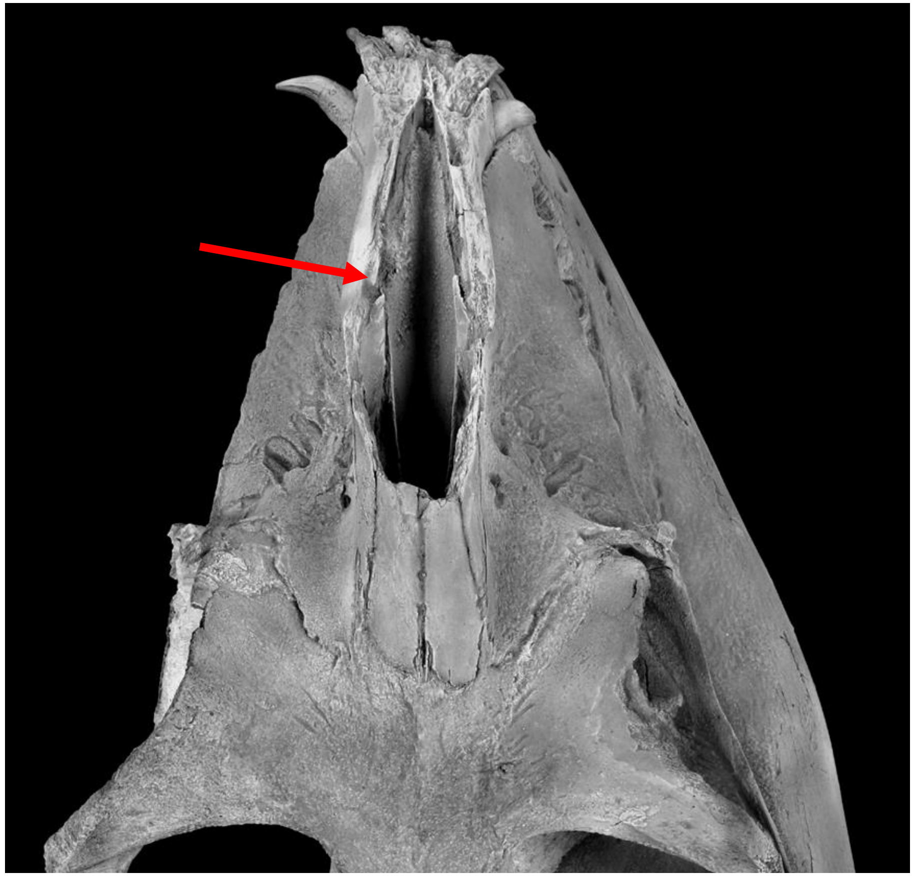

Janjucetus hunderi

Copyright holder: Erich M. G. Fitzgerald/ Museums Victoria, Melbourne, Australia

(1)

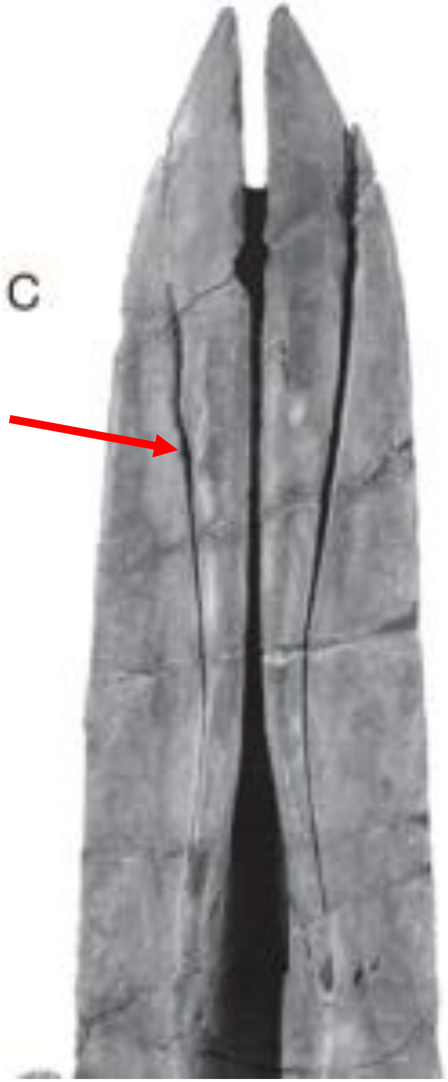

Piscobalaena nana

Adapted from: “The anatomy and relationships of *Piscobalaena nana* (Cetacea, Mysticeti), a Cetotheriidae s.s. from the early Pliocene of Peru”, Bouetel and Muizon, 2006. *Geodiversitas* 28.2 (2006): 319-395.

[11] 'Antorbital process'

(0)'absent'

(1)'present as steep face on the maxilla posteriorly bordering the antorbital notch'

(2)'present as a distinct anterior projection lateral to antorbital notch'

(0)

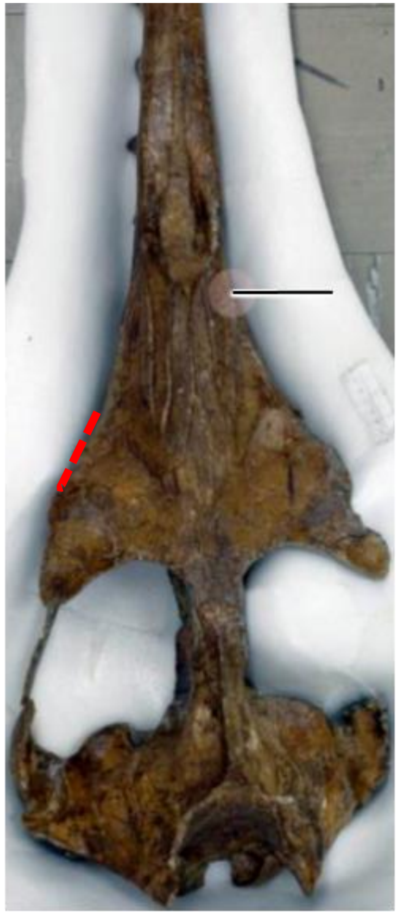

Zygorhiza kochii

Copyright holder: Felix G. Marx/ United States National Museum of Natural History, Washington DC, USA

(1)

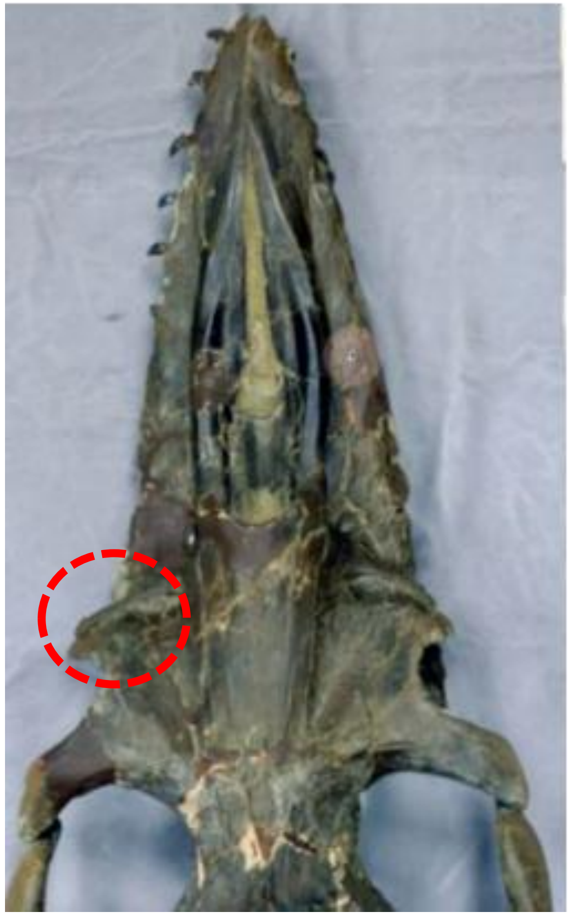

Aetiocetus weltoni

Copyright holder: Felix G. Marx/ University of California Museum of Paleontology, Berkeley, USA

(2)

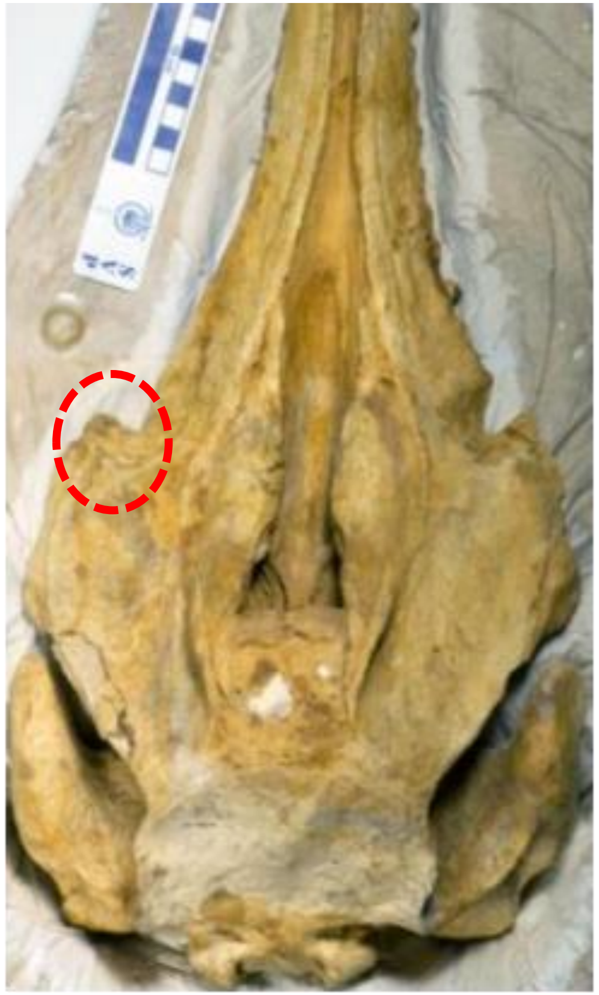

Waipatia maerewhenua

Copyright holder: Felix G. Marx/ University of Otago Museum of Geology, Dunedin, New Zealand

[12] 'Anterior border of supraorbital process lateral to ascending process of the maxilla'

(0) 'bordered by lacrimal only'

(1) 'bordered by lacrimal and maxilla'

(0)

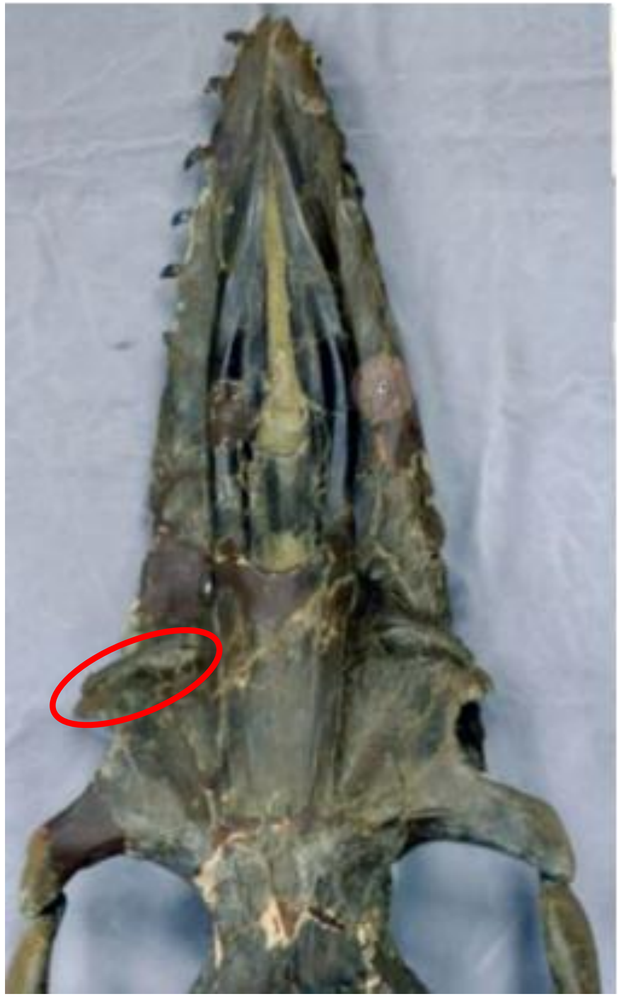

Aetiocetus weltoni

Copyright holder: Felix G. Marx/ University of California Museum of Paleontology, Berkeley, USA

(1)

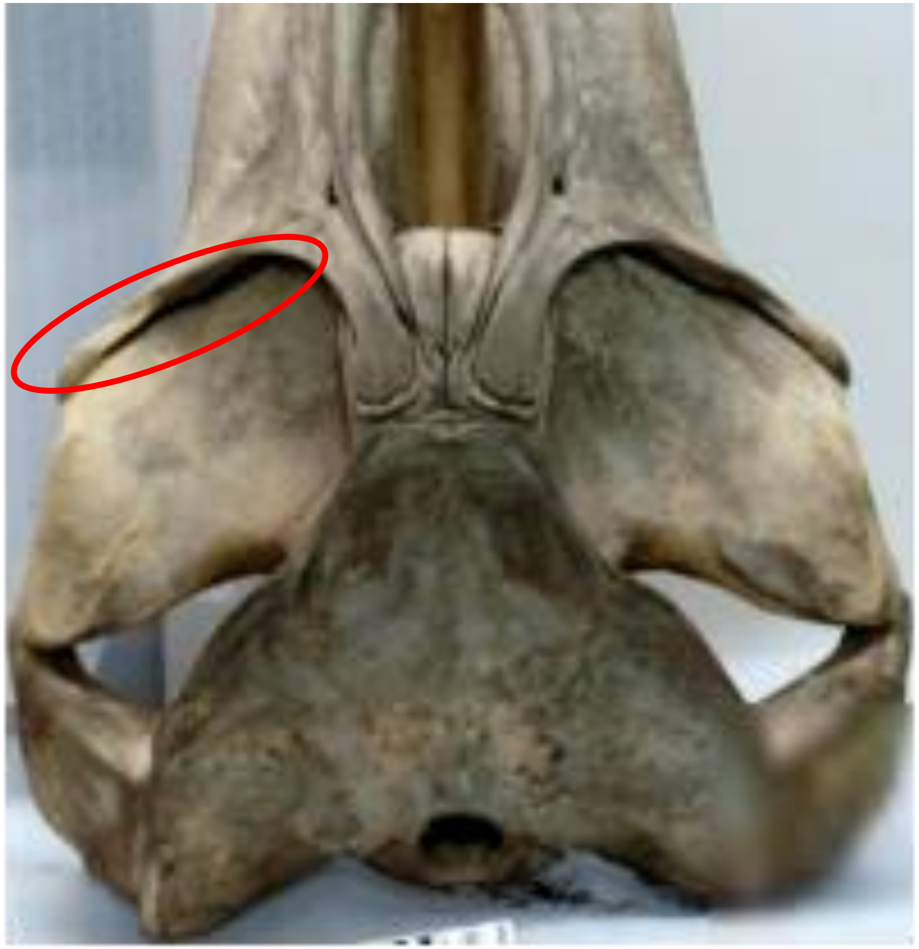

Balaenoptera acutorostrata

Copyright holder: Felix G. Marx/ The Charleston Museum, Charleston, South Carolina, USA

[13] 'Distinct pocket between the ascending process of the maxilla dorsally and the supraorbital process ventrally'

(0)'absent'

(1)'present'

(0)

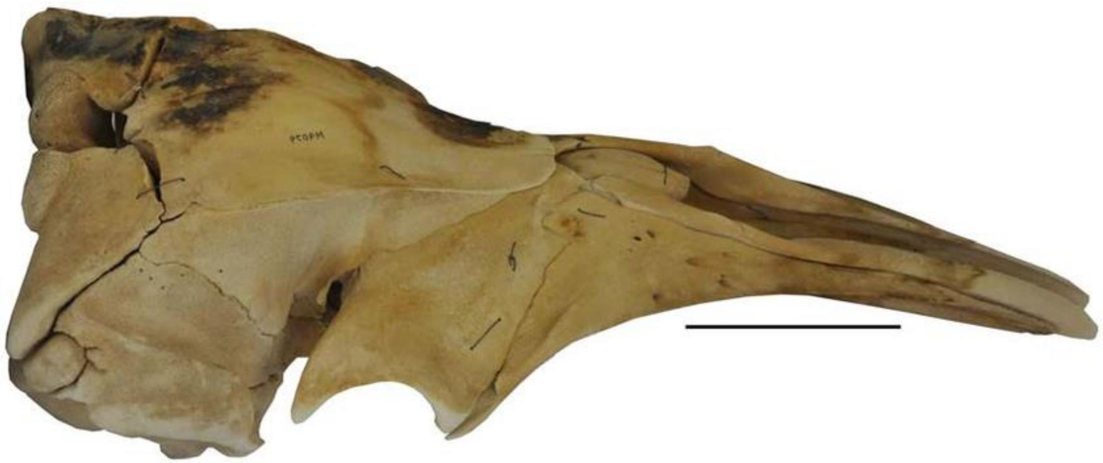

Caperea marginata (mirrored)

Adapted from: "Juvenile morphology in baleen whale phylogeny.Tsai, Cheng-Hsiu, and R. Ewan Fordyce, 2014. *Naturwissenschaften* 101.9 : 765-769. "

(1)

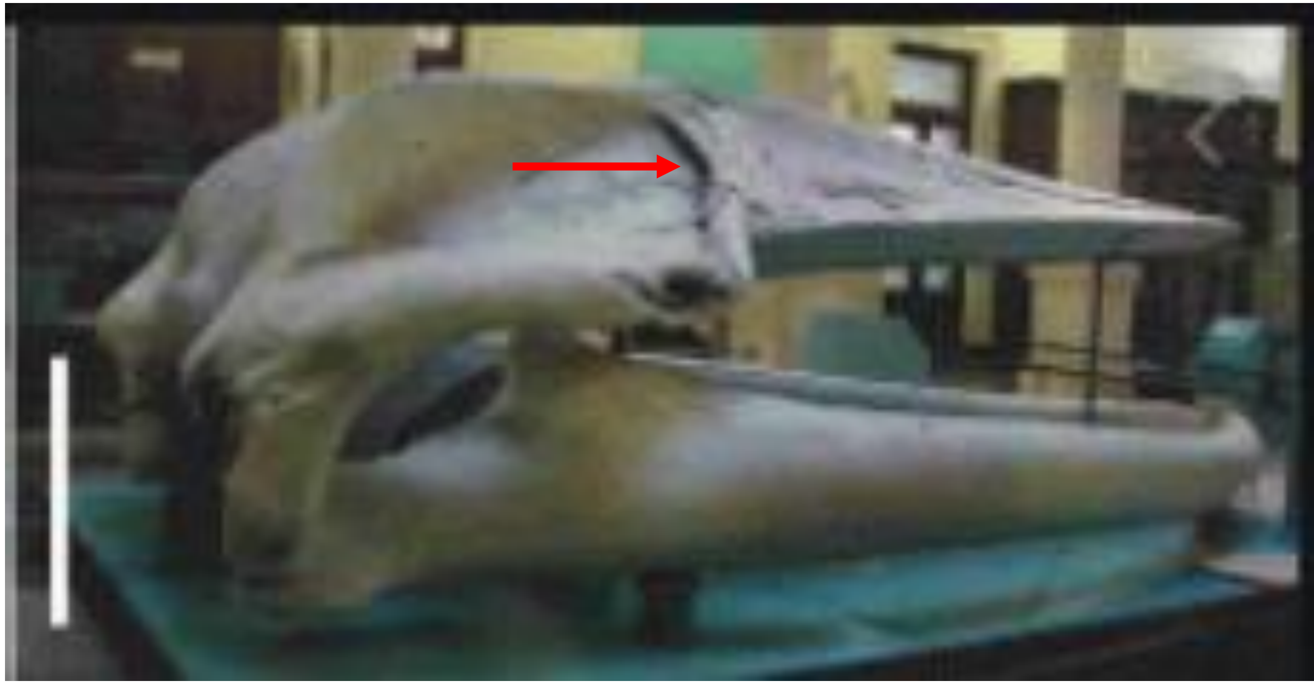

Balaenoptera musculus

Adapted from: "Catalogue of marine mammals of the Mammalogical collection of the Museo de La Plata, Argentina", Olivares et al, 2016. (original plate II of Lahille (1898))

[14] 'Lateral process of maxilla underlapping lacrimal'

(0)'absent'

(1)'present'

(0)

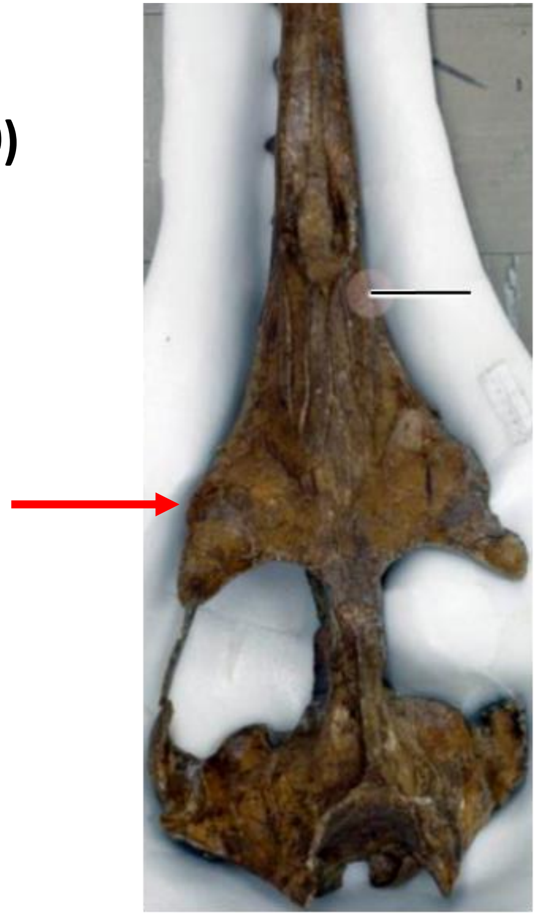

Zygorhiza kochii

Copyright holder: Felix G. Marx/ United States National Museum of Natural History, Washington DC, USA

(1)

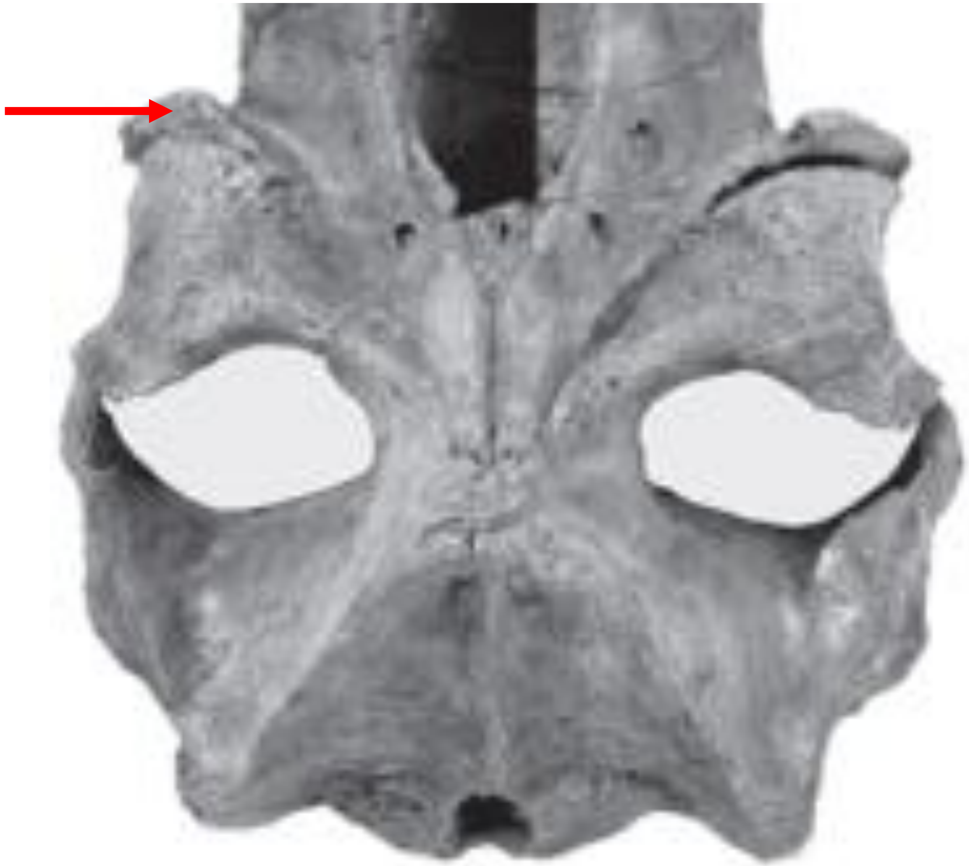

Piscobalaena nana

Adapted from: “The anatomy and relationships of *Piscobalaena nana* (Cetacea, Mysticeti), a Cetotheriidae s.s. from the early Pliocene of Peru”, Bouetel and Muizon, 2006. *Geodiversitas* 28.2 (2006): 319-395.

[15] 'Palatal keel formed by vomer and medial edges of maxillae'

(0)'absent'

(1)'present'

(0)

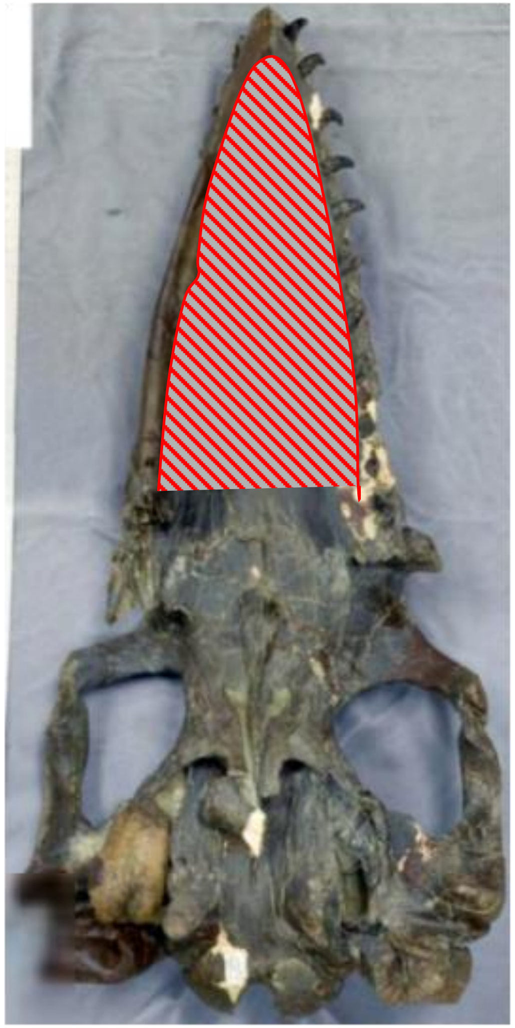

Aetiocetus weltoni

Copyright holder: Felix G. Marx/ University of California Museum of Paleontology, Berkeley, USA

(1)

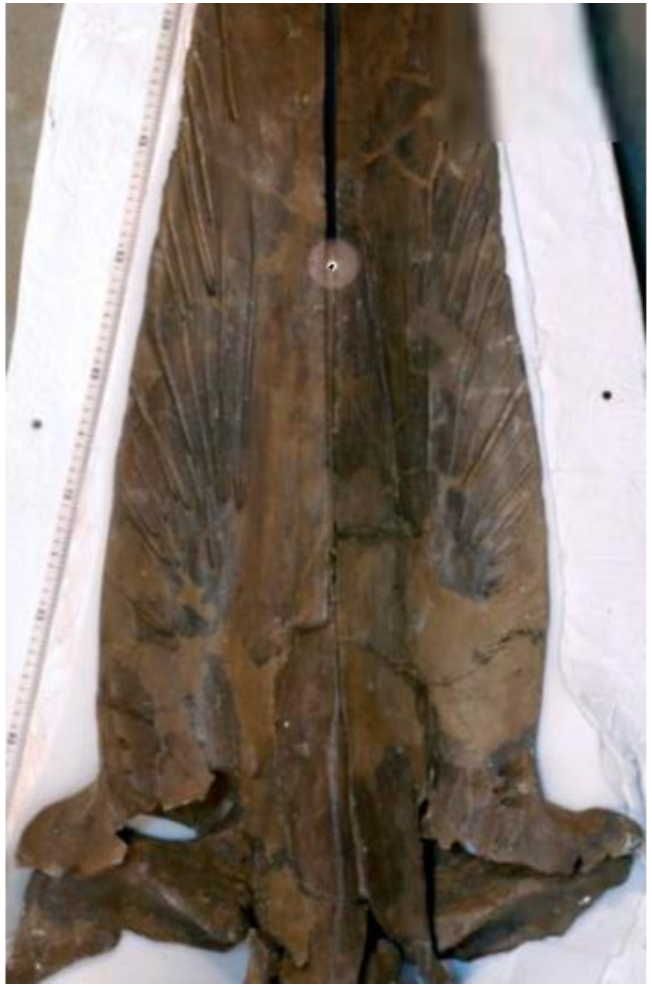

Diorocetus hiatus

Copyright holder: Felix G. Marx/ United States National Museum of Natural History, Washington DC, USA

[16] 'Exposure of premaxilla on palate'

(0) 'exposed along at least one third of the medial border of the maxilla'

(1) 'limited in extent to less than one third of the medial border of the maxilla'

(0)

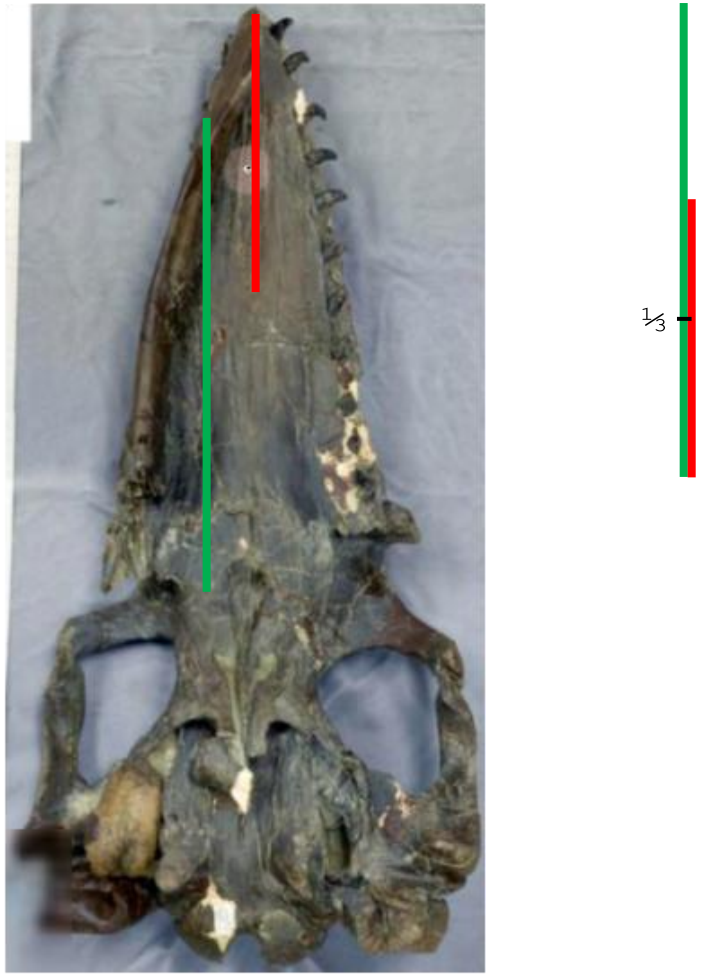

Aetiocetus weltoni

Copyright holder: Felix G. Marx/ University of California Museum of Paleontology, Berkeley, USA

(1)

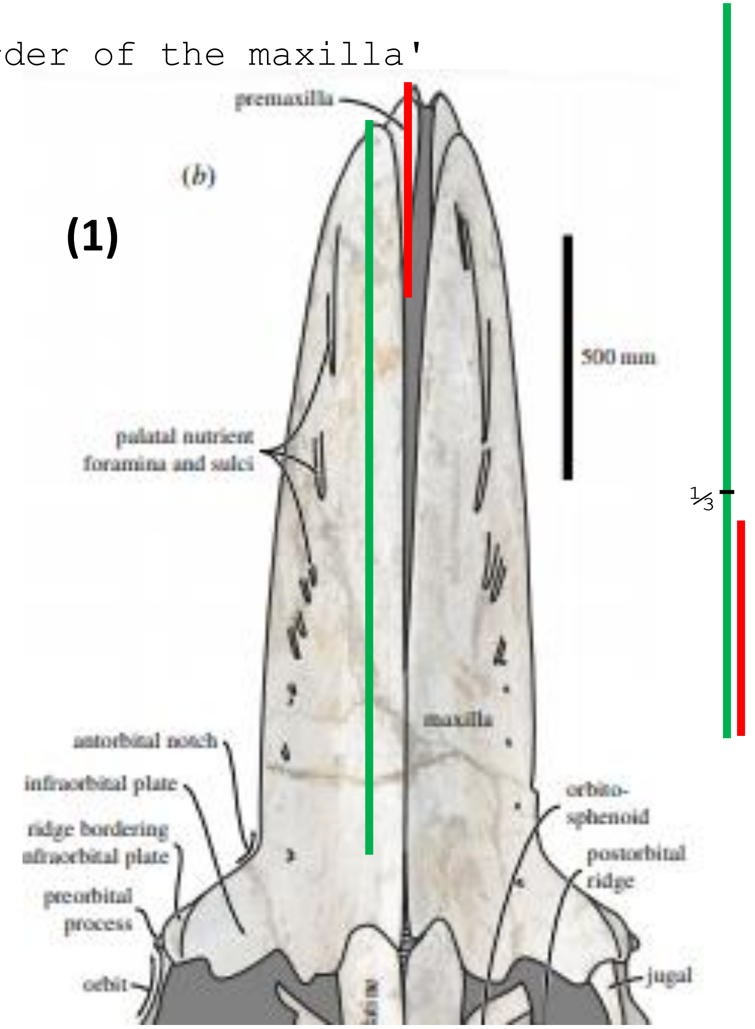

Incakujira anillodefuego

Adapted from: "A new Miocene baleen whale from the Peruvian desert." Marx and Kohno, 2016. *Royal Society Open Science* 3.10 (2016): 160542.

[17] 'Palatal window exposing vomer'

(0) 'present'

(1) 'narrow and variable exposure of vomer along most or all of the midline of the rostrum'

(2) 'vomer broadly exposed along the midline of the rostrum'

(0)

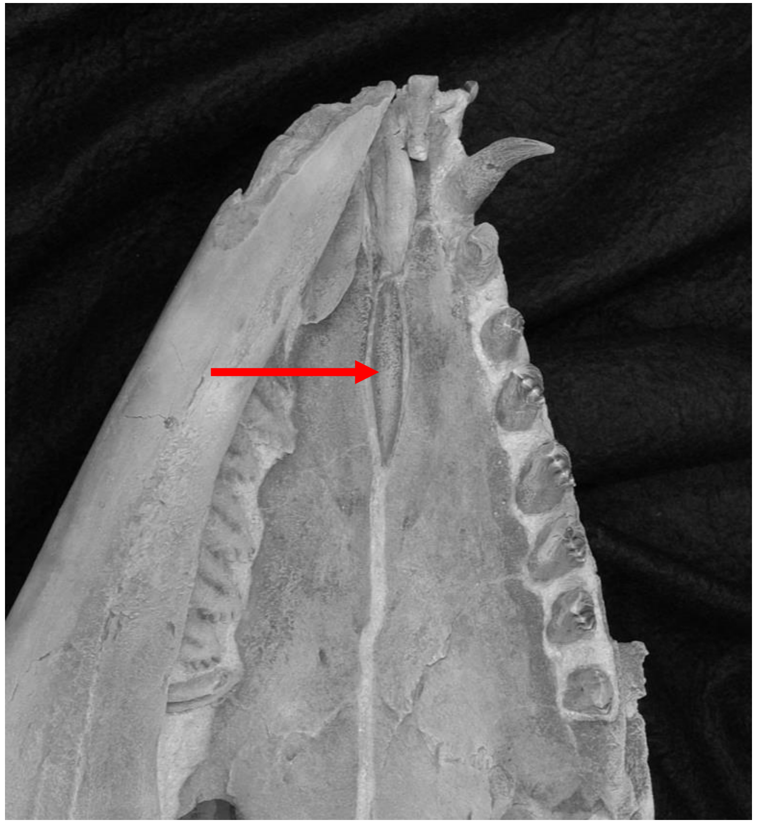

Janjucetus hunderi

Copyright holder: Erich M. G. Fitzgerald/ Museums Victoria, Melbourne, Australia

(1)

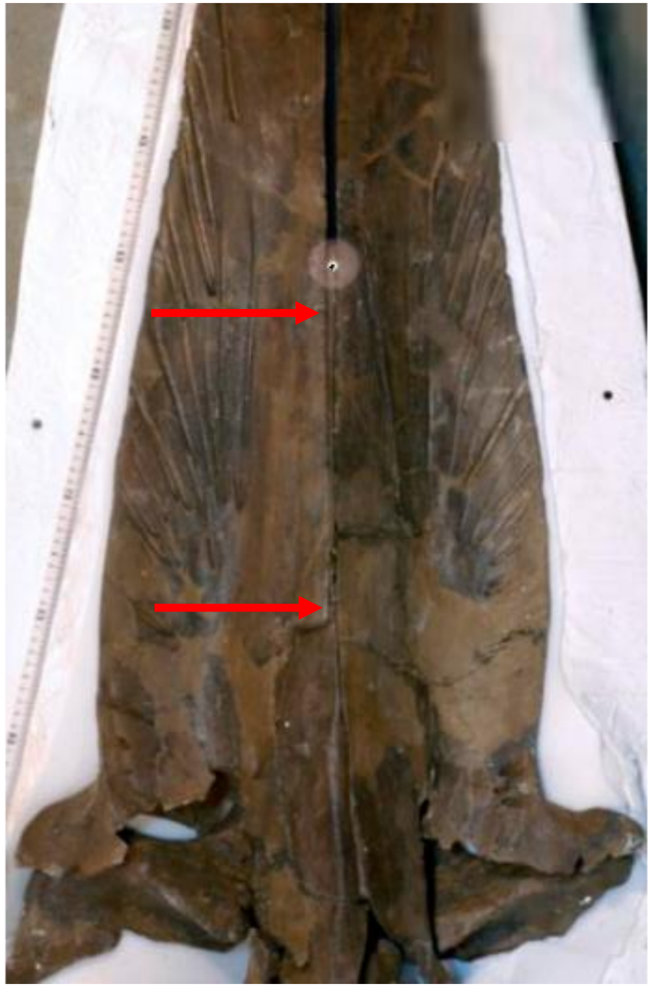

Diorocetus hiatus

Copyright holder: Felix G. Marx/ United States National Museum of Natural History, Washington DC, USA

(2)

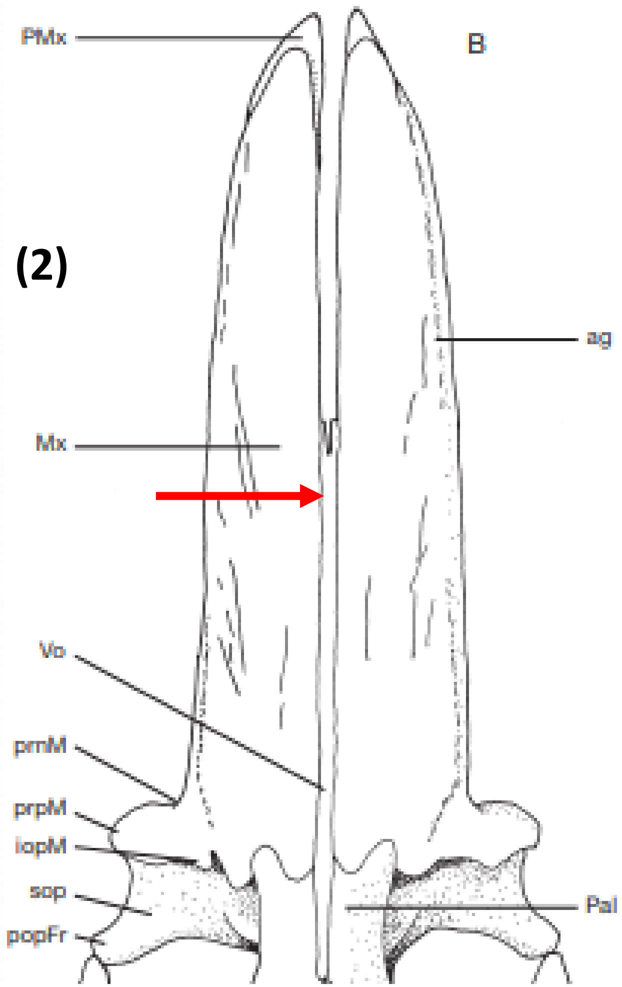

Piscobalaena nana

Adapted from: "The anatomy and relationships of *Piscobalaena nana* (Cetacea, Mysticeti), a Cetotheriidae s.s. from the early Pliocene of Peru", Bouetel and Muizon, 2006 *Geodiversitas* 28.2 (2006): 319-395.

[18] 'Palatal nutrient foramina and sulci'

(0)'absent'

(1)'present'

(0)

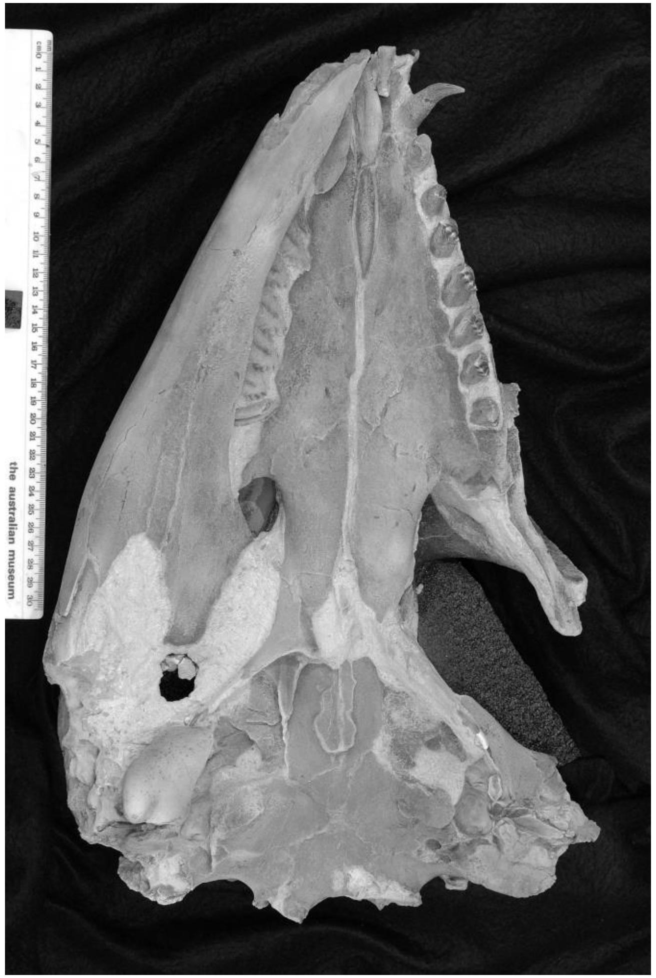

Janjucetus hunderi

Copyright holder: Erich M. G. Fitzgerald/ Museums Victoria, Melbourne, Australia

(1)

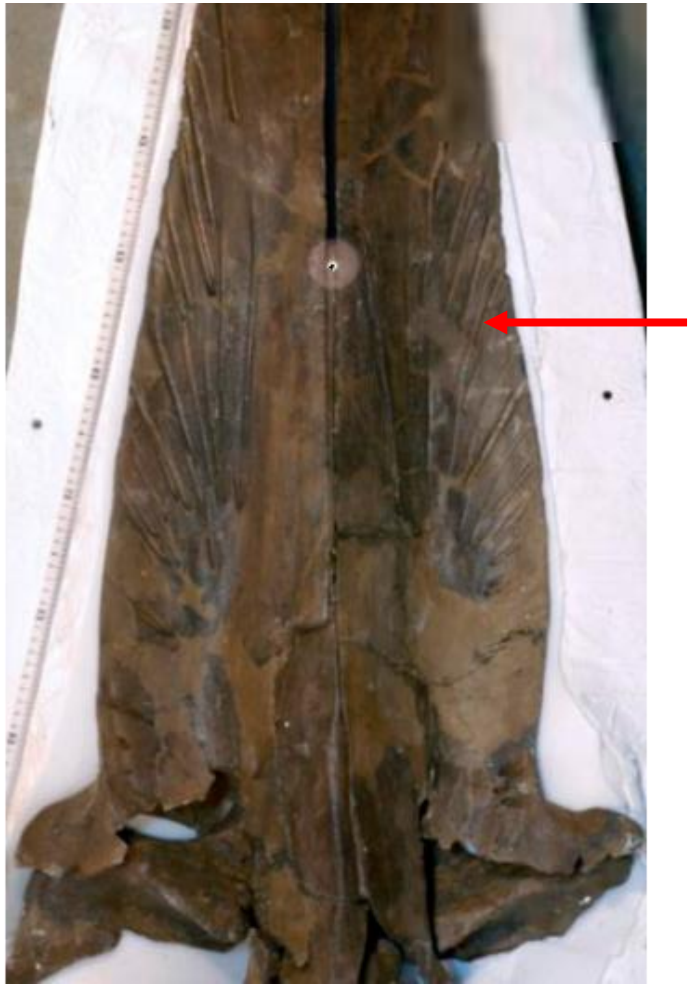

Diorocetus hiatus

Copyright holder: Felix G. Marx/ United States National Museum of Natural History, Washington DC, USA

[19] 'Outline of suture between maxillae and palatines'

(0) 'roughly straight transversely or bowed anteriorly'

(1) 'forms a posteriorly pointing V shape'

(2) 'anterior margins of palatines form two separate and posteriorly pointing U shapes'

(0)

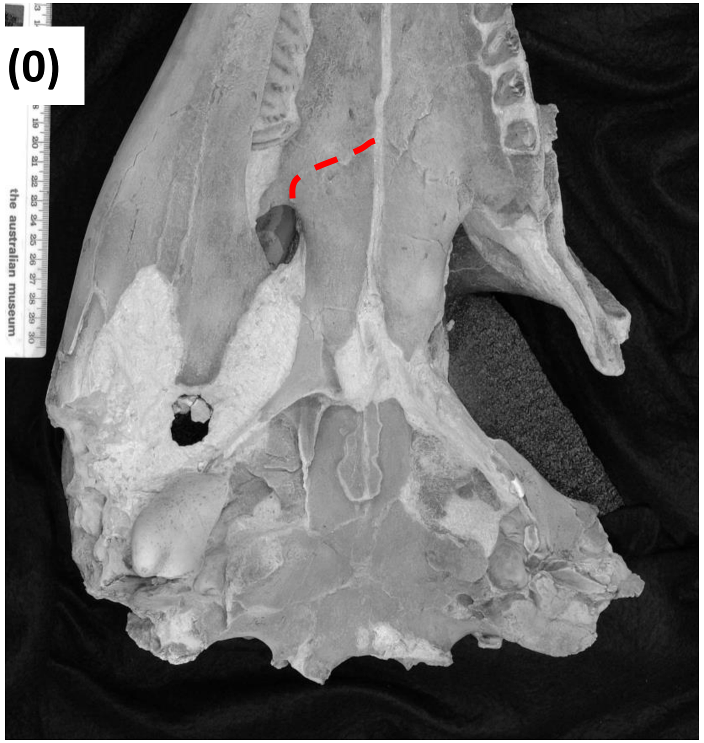

Janjucetus hunderi

Copyright holder: Erich M. G. Fitzgerald/ Museums Victoria, Melbourne, Australia

(1)

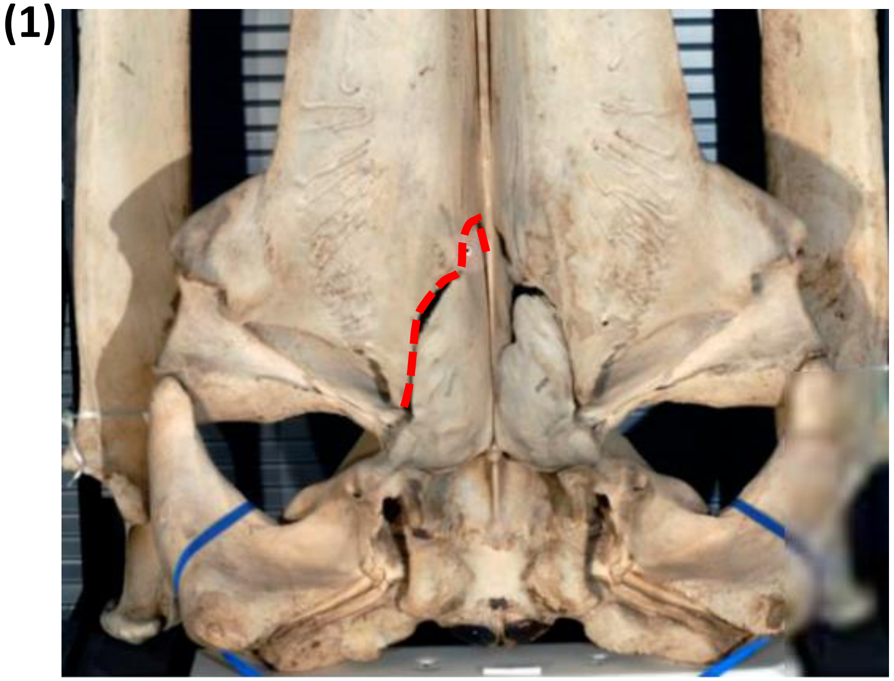

Balaenoptera borealis

Copyright holder: Felix G. Marx/ United States National Museum of Natural History, Washington DC, USA

(2)

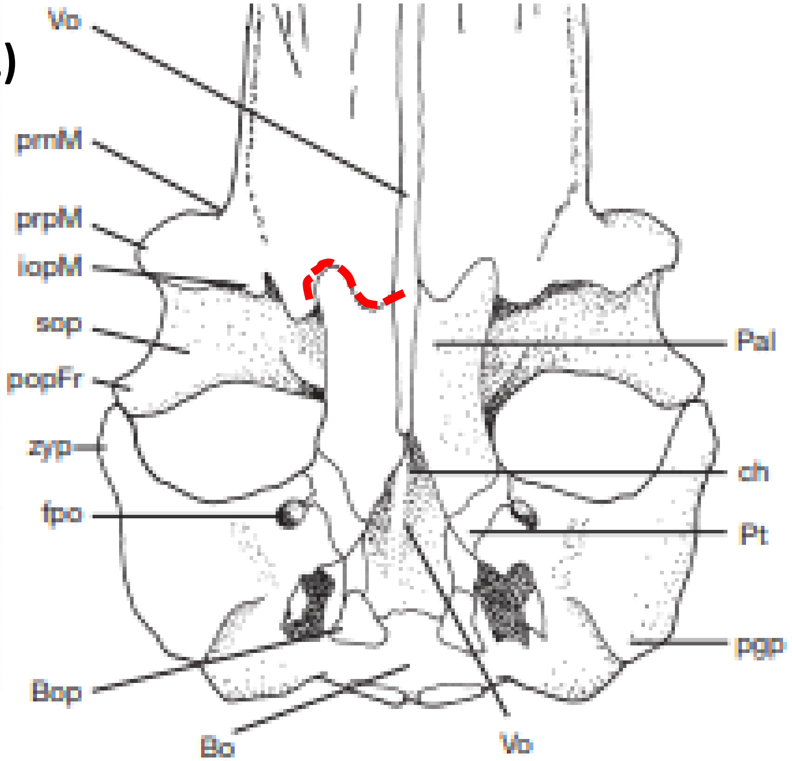

Piscobalaena nana

Adapted from: “The anatomy and relationships of *Piscobalaena nana* (Cetacea, Mysticeti), a Cetotheriidae s.s. from the early Pliocene of Peru”, Bouetel and Muizon, 2006. *Geodiversitas* 28.2 (2006): 319-395.

[20] 'Anteriormost point of palatine'

(0)'located in line with or posterior to the level of the antorbital notch or equivalent point on rostrum'

(1)'located anterior to the level of the antorbital notch'

(0)

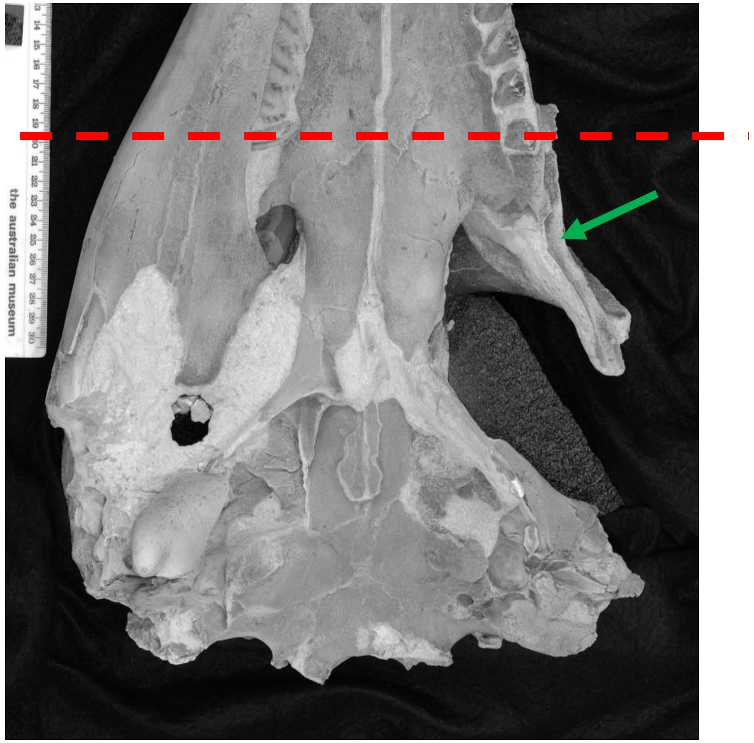

Janjucetus hunderi

Copyright holder: Erich M. G. Fitzgerald/ Museums Victoria, Melbourne, Australia

(1)

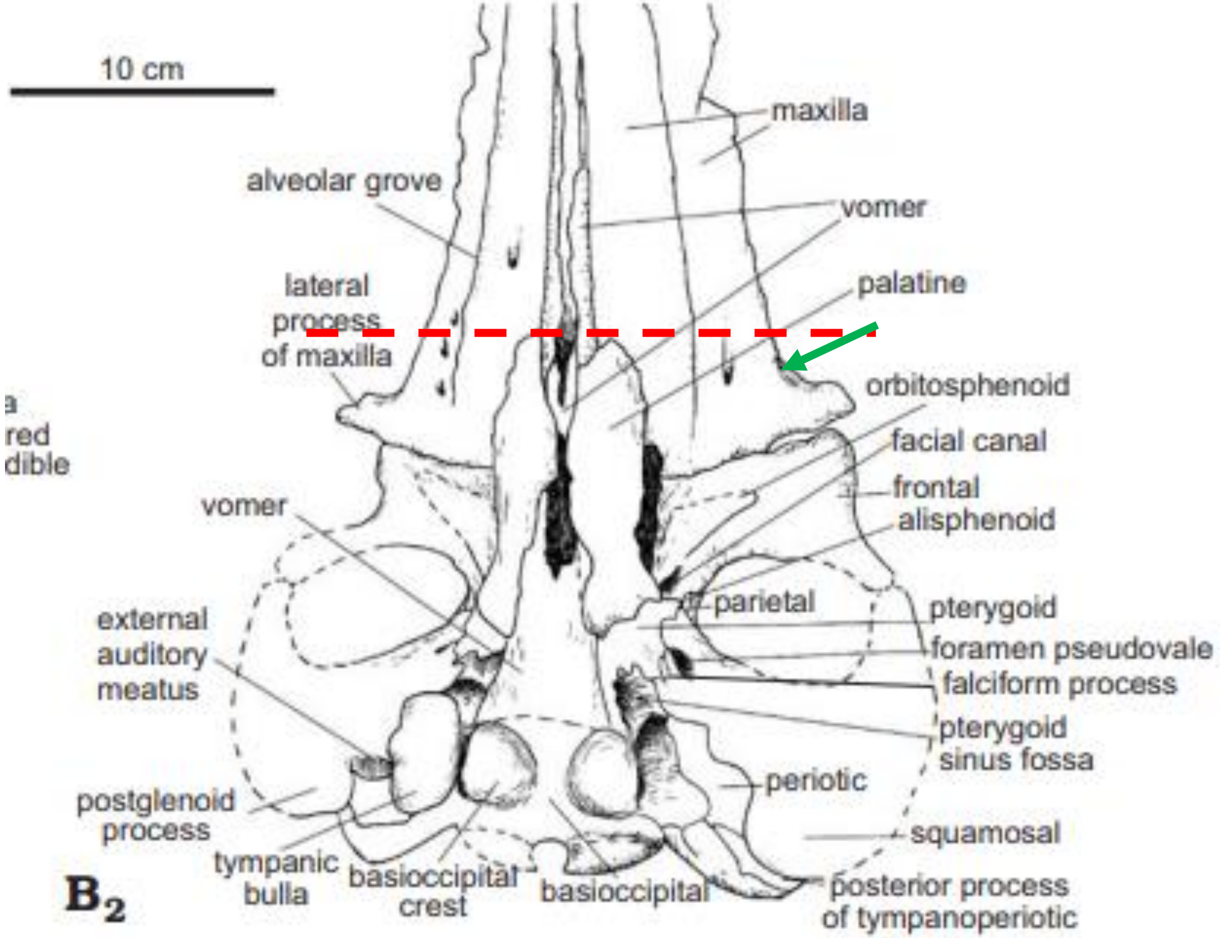

Cetotherium riabinini

Adapted from: "The anatomy of the Late Miocene baleen whale Cetotherium riabinini from Ukraine." Gol'din et al., 2013. *Acta Palaeontologica Polonica* 59.4: 795-814.

[21] 'Anteromedial portion of palatine'

(0) 'flattened or meeting at a relatively blunt angle in cross section'

(1) 'transversely pinched and forming a well-developed medial crest'

(0)

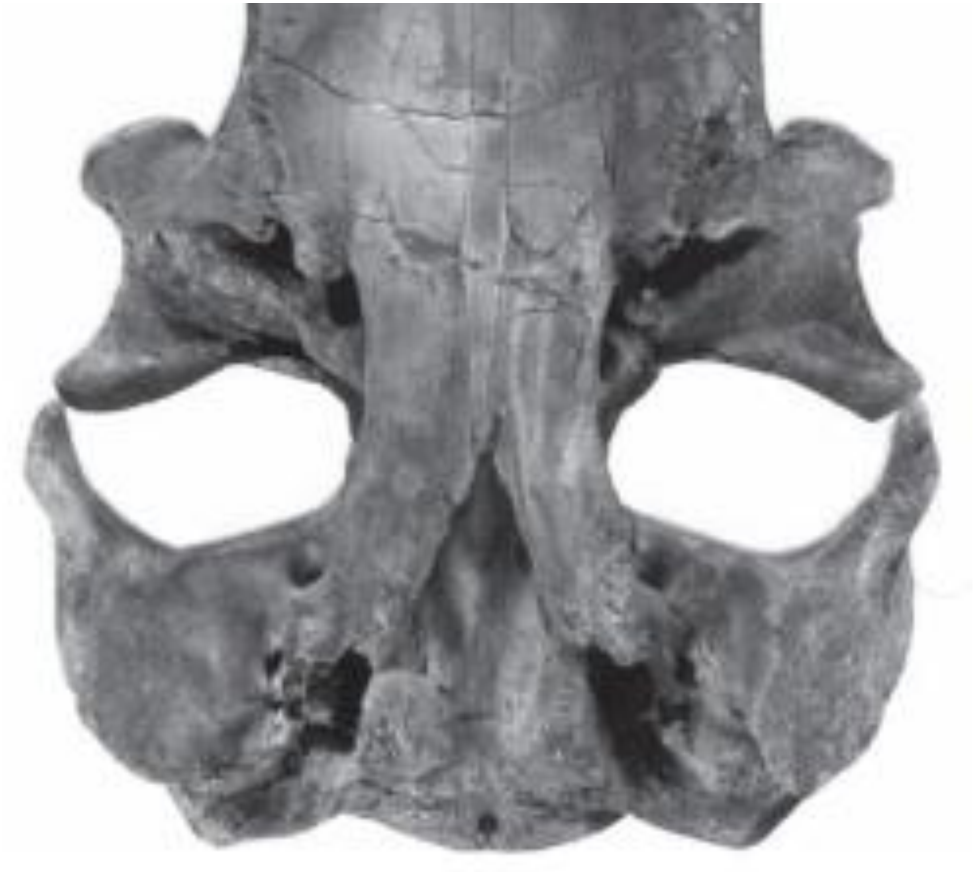

Piscobalaena nana

Adapted from: "The anatomy and relationships of *Piscobalaena nana* (Cetacea, Mysticeti), a Cetotheriidae s.s. from the early Pliocene of Peru", Bouetel and Muizon, 2006. *Geodiversitas* 28.2 (2006): 319-395.

(1)

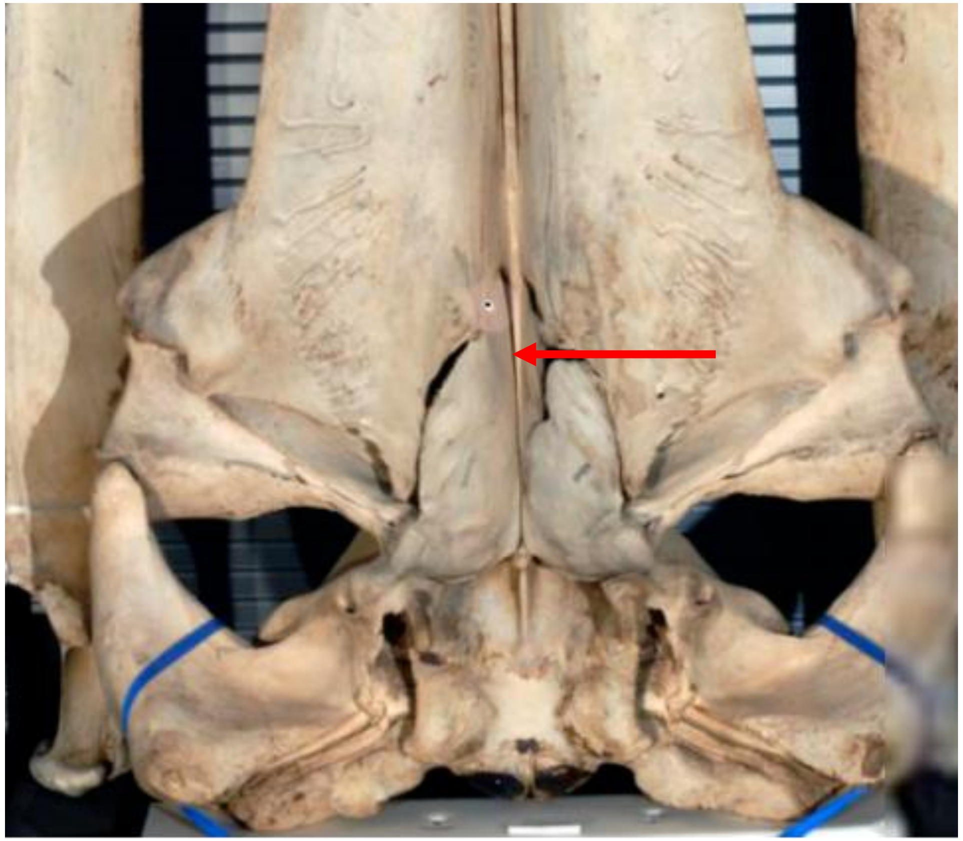

Balaenoptera borealis

Copyright holder: Felix G. Marx/ United States National Museum of Natural History, Washington DC, USA

[22] 'Anterior edge of narial fossa'

(0) 'located in posterior three quarters of rostrum'

(1) 'located in anterior quarter of rostrum'

(0)

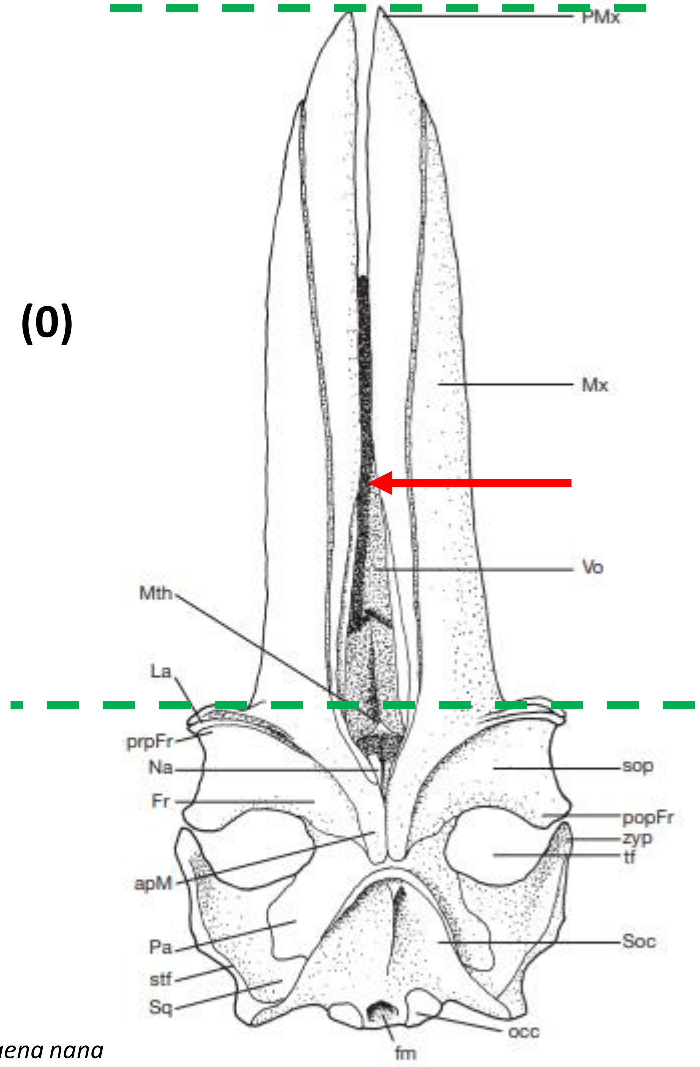

Piscobalaena nana

Adapted from: "The anatomy and relationships of *Piscobalaena nana* (Cetacea, Mysticeti), a Cetotheriidae s.s. from the early Pliocene of Peru", Bouetel and Muizon, 2006. *Geodiversitas* 28.2 (2006): 319-395.

(1)

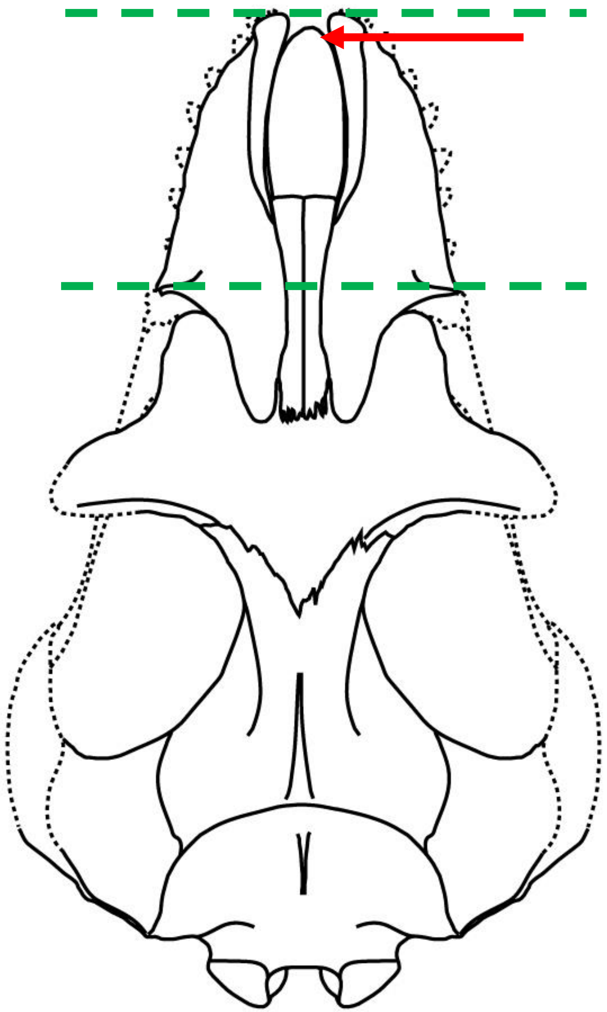

Mammalodon colliveri

Copyright holder: Erich M. G. Fitzgerald/ Museums Victoria, Melbourne, Australia

[23] 'Facial portion of rostrum in lateral view'

(0) 'step-like'

(1) 'straight'

(2) 'concave'

(0)

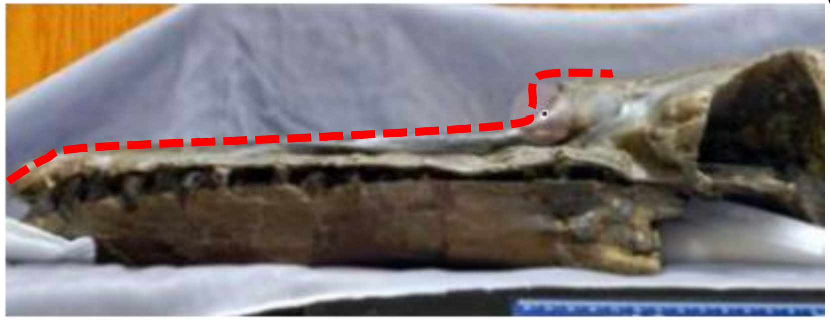

Aetiocetus weltoni

Copyright holder: Felix G. Marx/ University of California Museum of Paleontology, Berkeley, USA

(1)

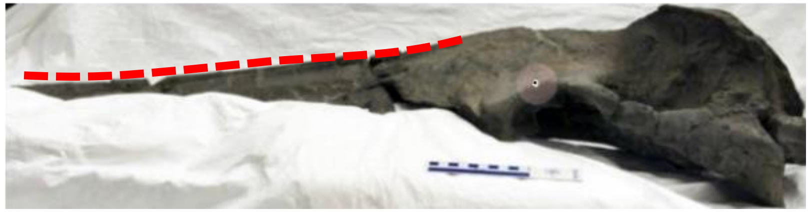

Isanacetus laticephalus

Copyright holder: Felix G. Marx/ Mizunami Fossil Museum, Gifu, Japan

(2)

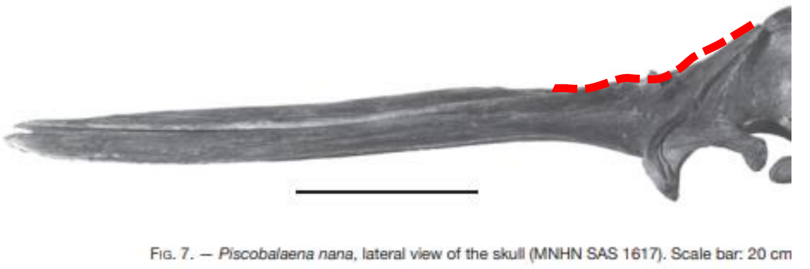

Piscobalaena nana

Adapted from: "The anatomy and relationships of *Piscobalaena nana* (Cetacea, Mysticeti), a Cetotheriidae s.s. from the early Pliocene of Peru", Bouetel and Muizon, 2006. *Geodiversitas* 28.2 (2006): 319-395.

[24] 'Rostrum shape'

(0)'width at antorbital notches or equivalent point on rostrum less than 80% the length of the rostrum as measured from its tip to the antorbital notches'

(1)'width at antorbital notches or equivalent point more than 80% the length of the rostrum'

(0)

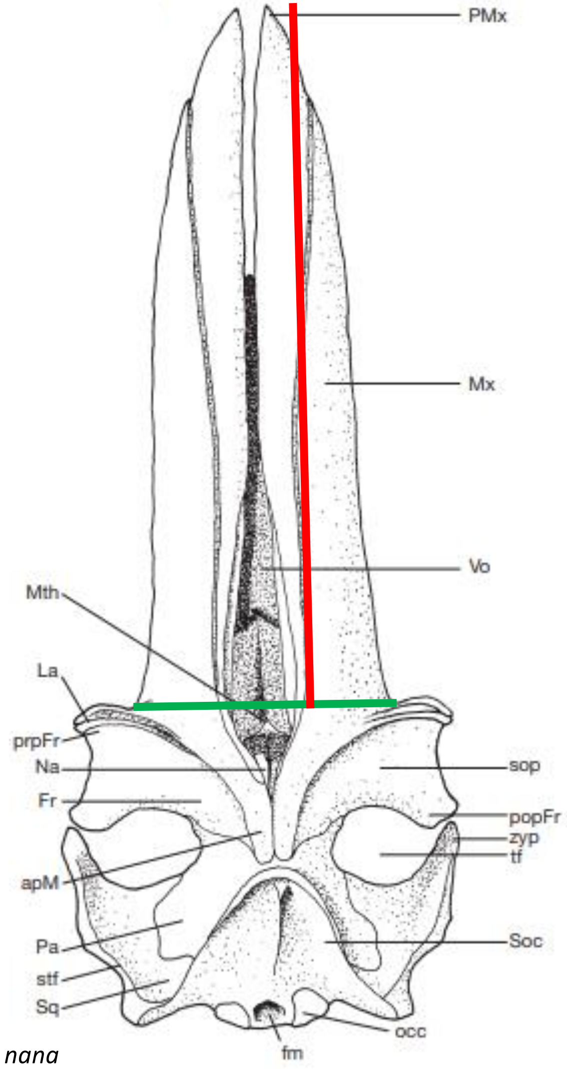

Piscobalaena nana

Adapted from: "The anatomy and relationships of *Piscobalaena nana* (Cetacea, Mysticeti), a Cetotheriidae s.s. from the early Pliocene of Peru", Bouetel and Muizon, 2006. *Geodiversitas* 28.2 (2006): 319-395.

(1)

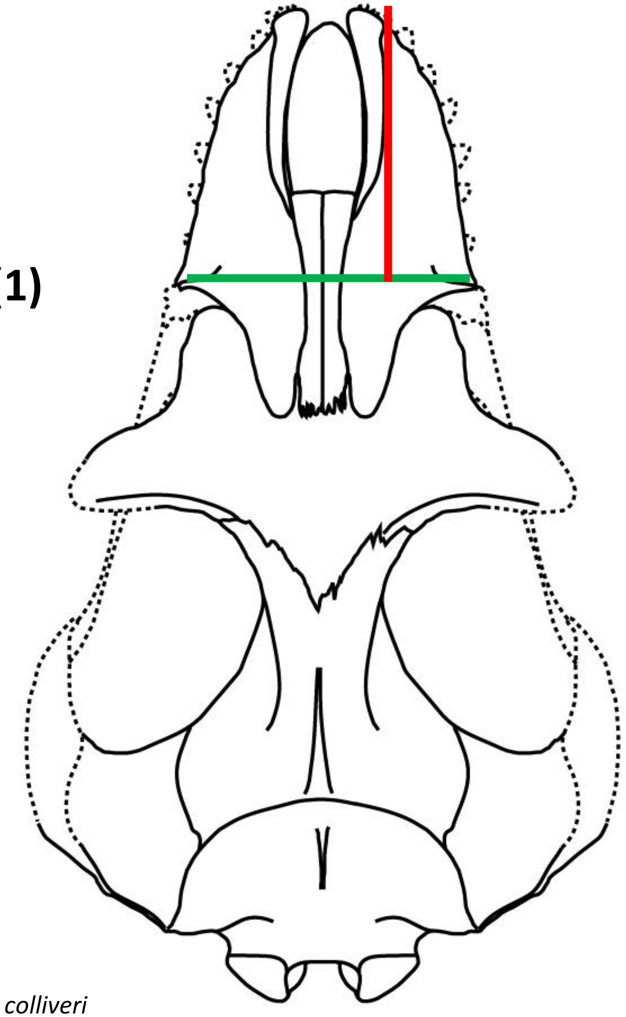

Mammalodon colliveri

Copyright holder: Erich M. G. Fitzgerald/ Museums Victoria, Melbourne, Australia

[25] 'Teeth in adult individuals'

(0)'present'

(1)'absent or vestigial'

(0)

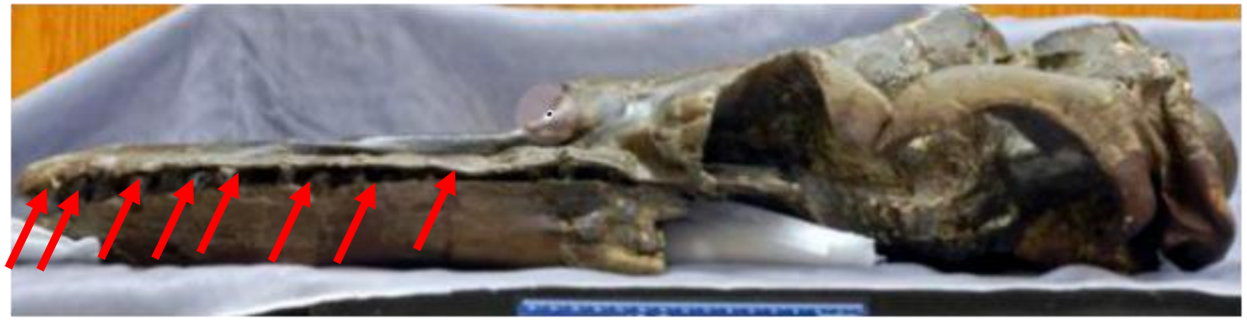

Aetiocetus weltoni

Copyright holder: Felix G. Marx/ University of California Museum of Paleontology, Berkeley, USA

(1)

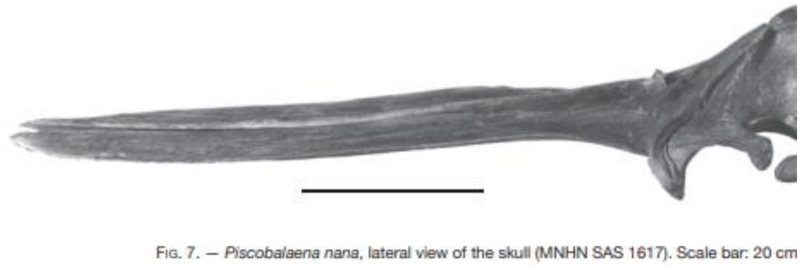

Piscobalaena nana

Adapted from: “The anatomy and relationships of *Piscobalaena nana* (Cetacea, Mysticeti), a Cetotheriidae s.s. from the early Pliocene of Peru”, Bouetel and Muizon, 2006. *Geodiversitas* 28.2 (2006): 319-395.

[37] 'Skull length about one third of total body length'

(0)'absent'

(1)'present'

(0)

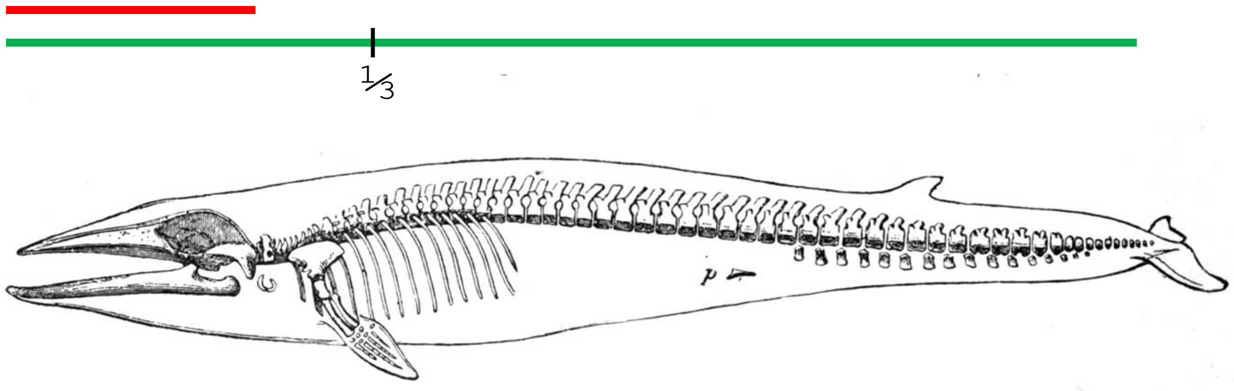

Balaenoptera physalus

Adapted from: Lydekker, 1894. Royal Natural History. Vol 3, Page 18.

(1)

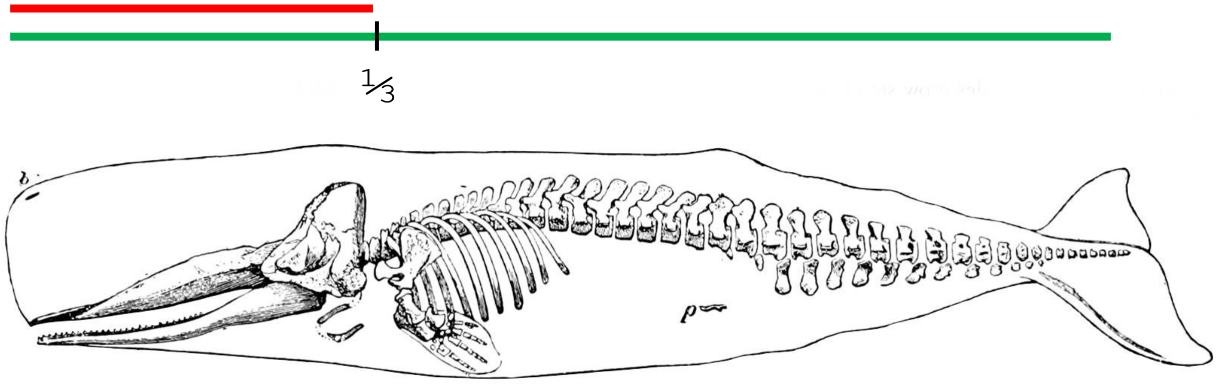

Physeter macrocephalus

Adapted from: Lydekker, 1894. Royal Natural History. Vol 3, Page 24.

[38] 'Facial asymmetry'

(0)'absent'

(1)'present'

(0)

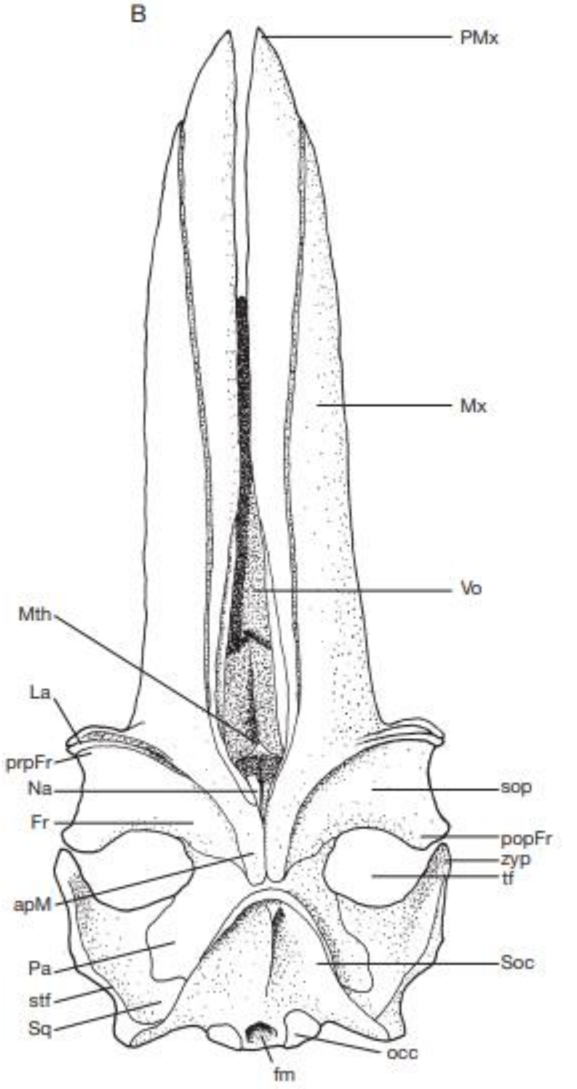

Piscobalaena nana

Adapted from: "The anatomy and relationships of *Piscobalaena nana* (Cetacea, Mysticeti), a Cetotheriidae s.s. from the early Pliocene of Peru", Bouetel and Muizon, 2006. *Geodiversitas* 28.2 (2006): 319-395.

(1)

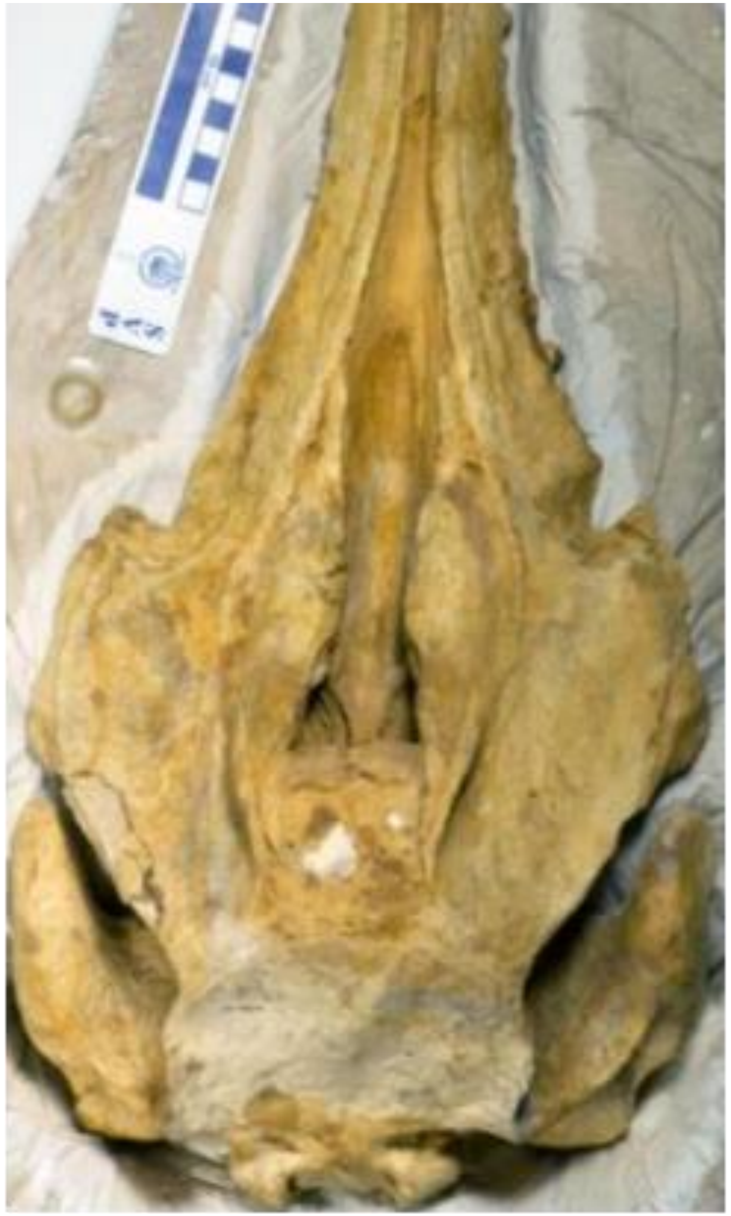

Waipatia maerewhenua

Copyright holder: Felix G. Marx/ University of Otago Museum of Geology, Dunedin, New Zealand

[39] 'Diameter of orbit as measured between the distalmost points of the preorbital and postorbital processes'

(0)'less than 25% of bizygomatic width'

(1)'25% or more'

(0)

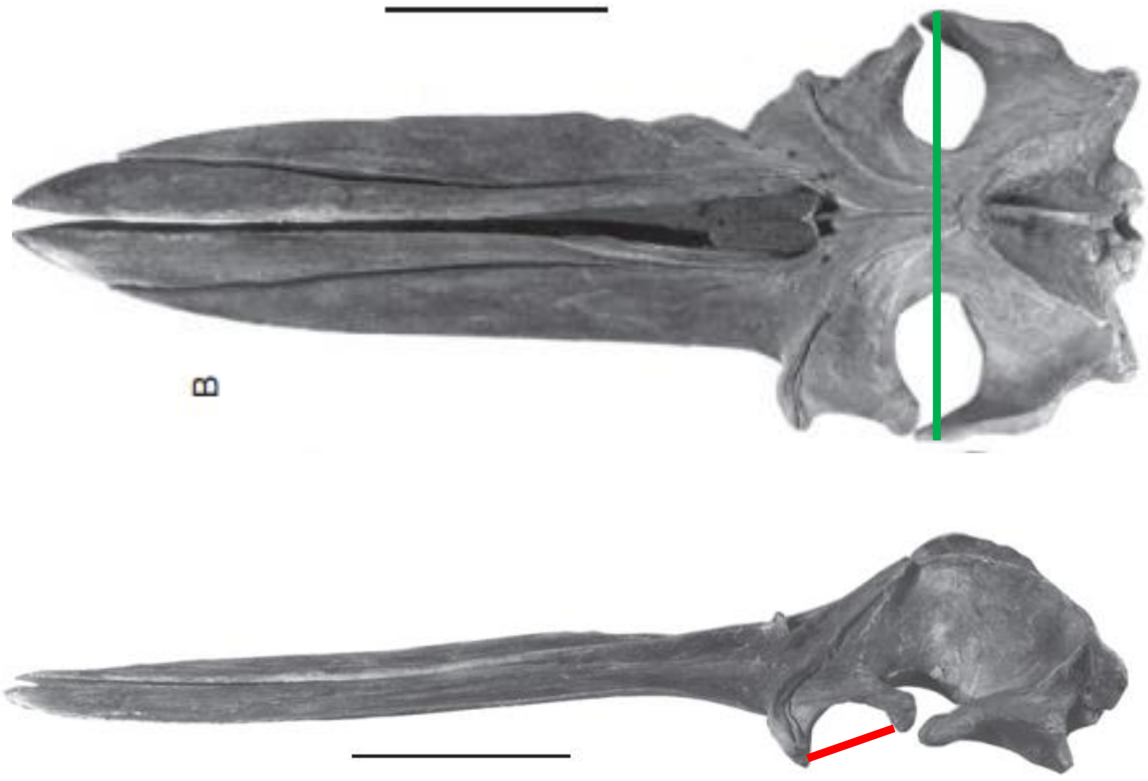

Piscobalaena nana

Adapted from: "The anatomy and relationships of *Piscobalaena nana* (Cetacea, Mysticeti), a Cetotheriidae s.s. from the early Pliocene of Peru", Bouetel and Muizon, 2006. *Geodiversitas* 28.2 (2006): 319-395.

(1)

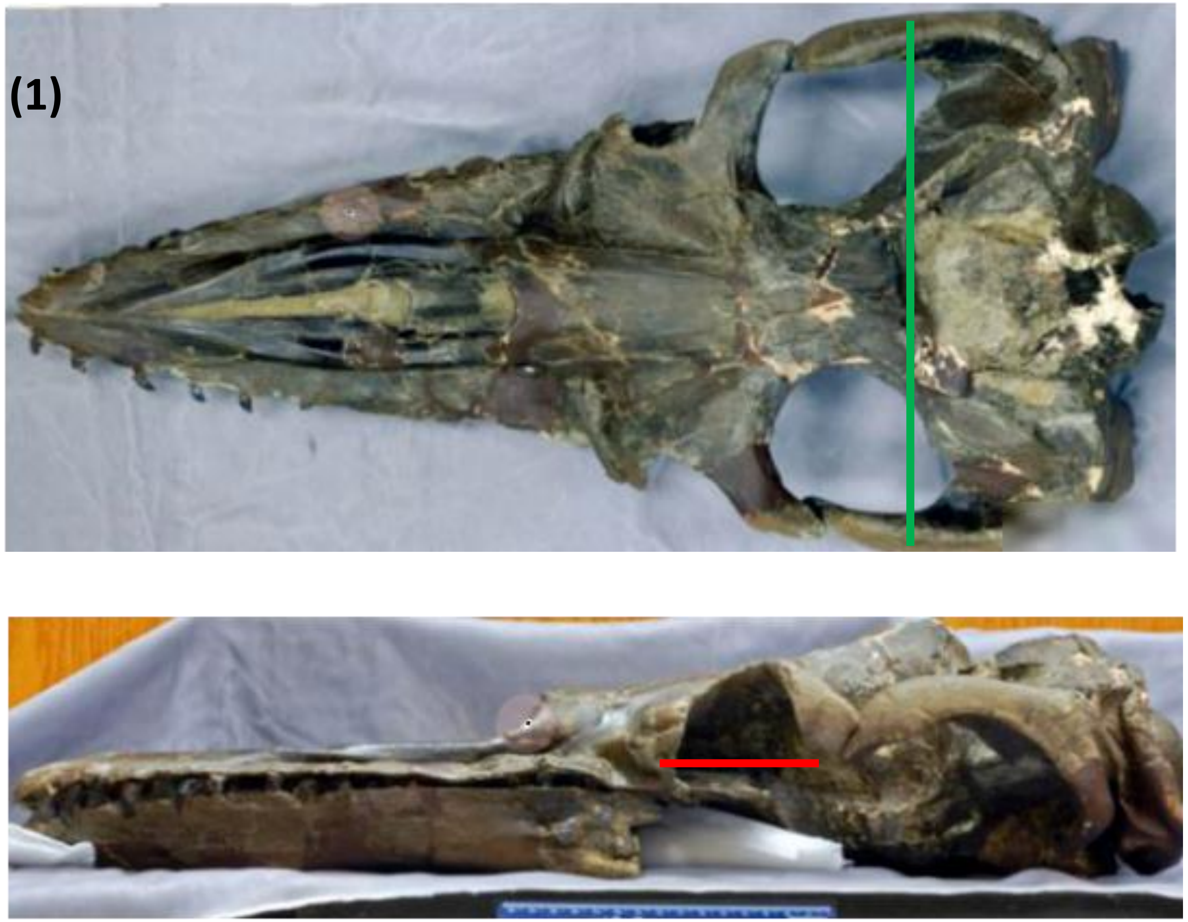

Aetiocetus weltoni

Copyright holder: Felix G. Marx/ University of California Museum of Paleontology, Berkeley, USA

[40] 'Anterior edge of supraorbital process lateral to ascending process of maxilla'

(0) 'pointing posteriorly'

(1) 'oriented transversely or pointing anteriorly'

(2) 'linguiform and tapering to a point'

(0)

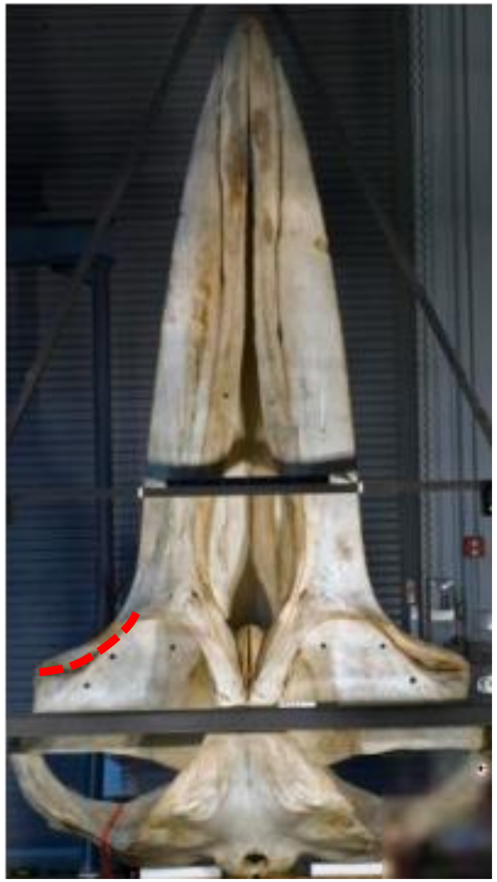

Megaptera novaeangliae

Copyright holder: Felix G. Marx/ United States National Museum of Natural History, Washington DC, USA

(1)

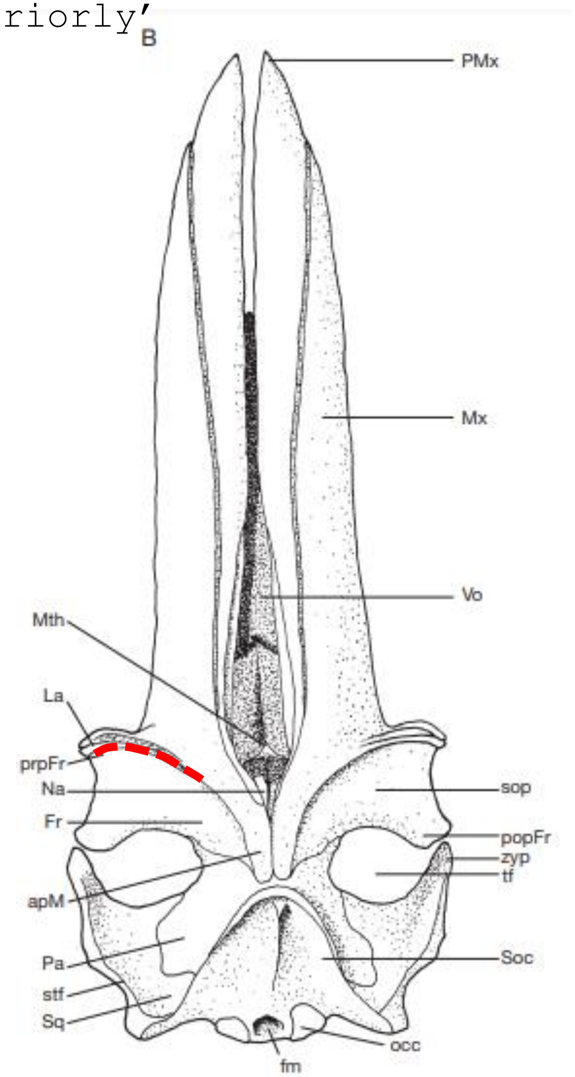

Piscobalaena nana

Adapted from: "The anatomy and relationships of *Piscobalaena nana* (Cetacea, Mysticeti), a Cetotheriidae s.s. from the early Pliocene of Peru", Bouetel and Muizon, 2006. *Geodiversitas* 28.2 (2006): 319-395.

(2)

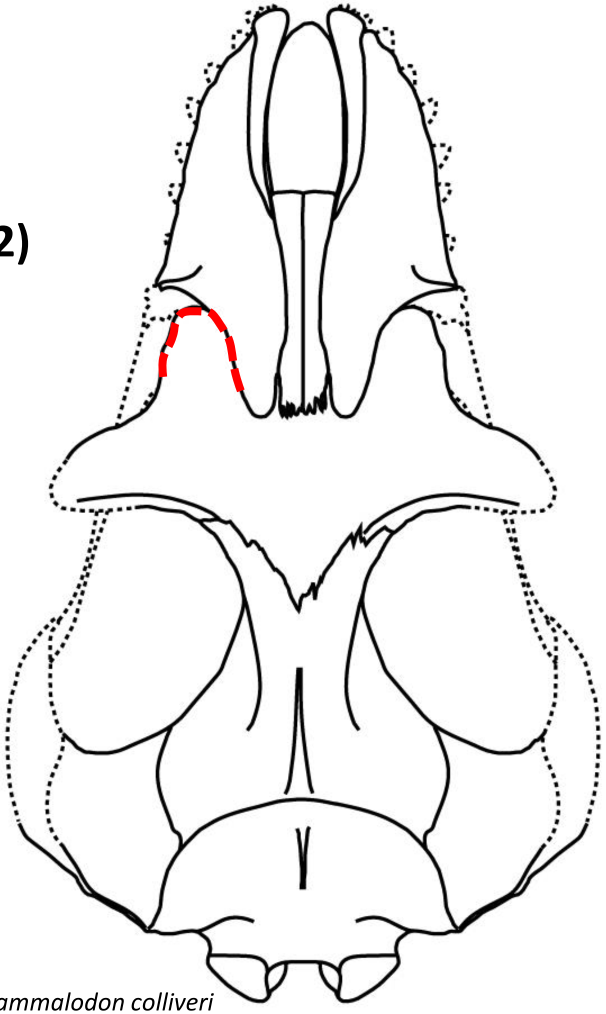

Mammalodon colliveri

Copyright holder: Erich M. G. Fitzgerald/ Museums Victoria, Melbourne, Australia

[41] 'Outline of anterior edge of supraorbital process in dorsal view'

(0)'roughly straight or concave'

(1)'distinctly sinusoidal'

(0)

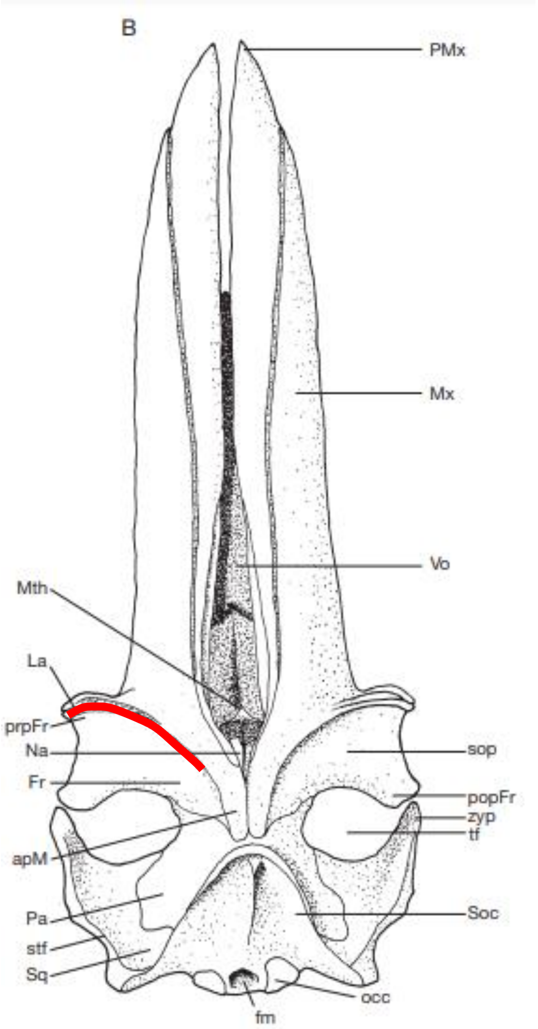

Piscobalaena nana

Adapted from: “The anatomy and relationships of *Piscobalaena nana* (Cetacea, Mysticeti), a Cetotheriidae s.s. from the early Pliocene of Peru”, Bouetel and Muizon, 2006. *Geodiversitas* 28.2 (2006): 319-395.

(1)

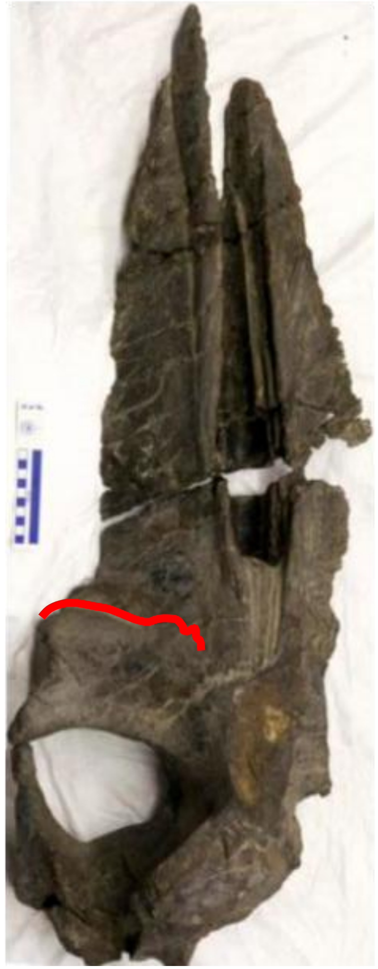

Isanacetus laticephalus

Copyright holder: Felix G. Marx/ Mizunami Fossil Museum, Gifu, Japan

[42] 'Posterior border of supraorbital process in dorsal view'

(0) 'concave'

(1) 'straight'

(0)

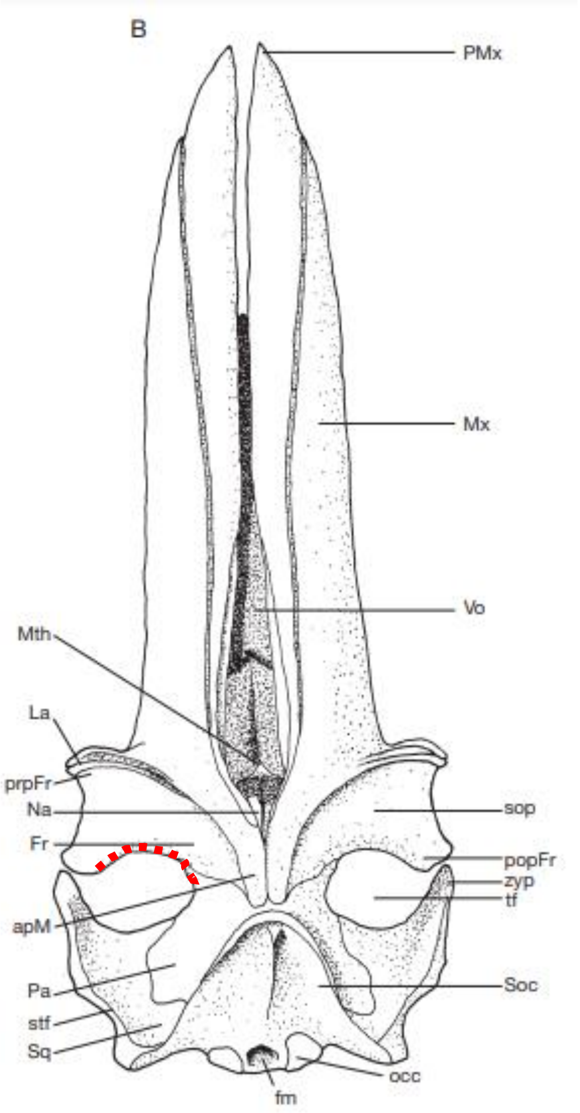

Piscobalaena nana

Adapted from: “The anatomy and relationships of *Piscobalaena nana* (Cetacea, Mysticeti), a Cetotheriidae s.s. from the early Pliocene of Peru”, Bouetel and Muizon, 2006. *Geodiversitas* 28.2 (2006): 319-395.

(1)

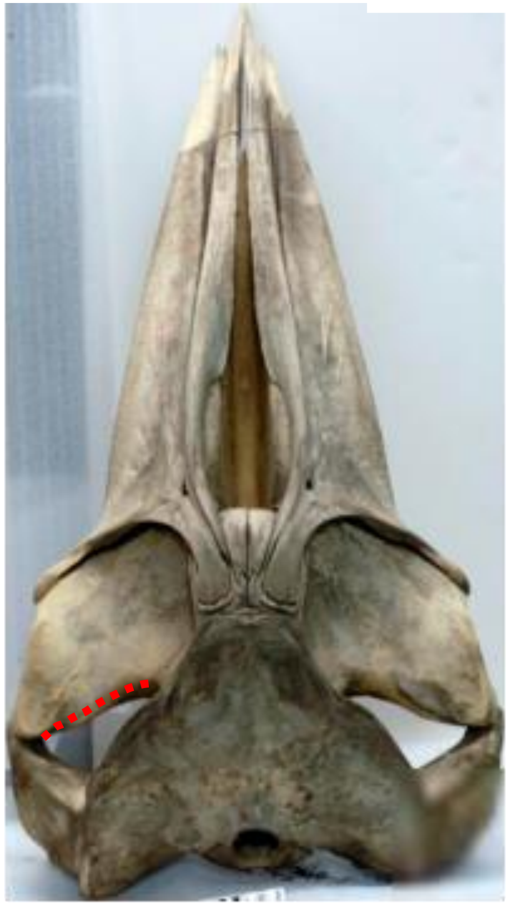

Balaenoptera acutorostrata

Copyright holder: Felix G. Marx/ The Charleston Museum, Charleston, South Carolina, USA

[43] 'Supraorbital process of frontal in anterior view'

(0)'horizontal or nearly horizontal'

(1)'gradually slopes away lateroventrally from the skull vertex'

(2)'abruptly depressed to a level noticeably below the vertex with the lateral skull wall above the supraorbital formed by both parietal and frontal'

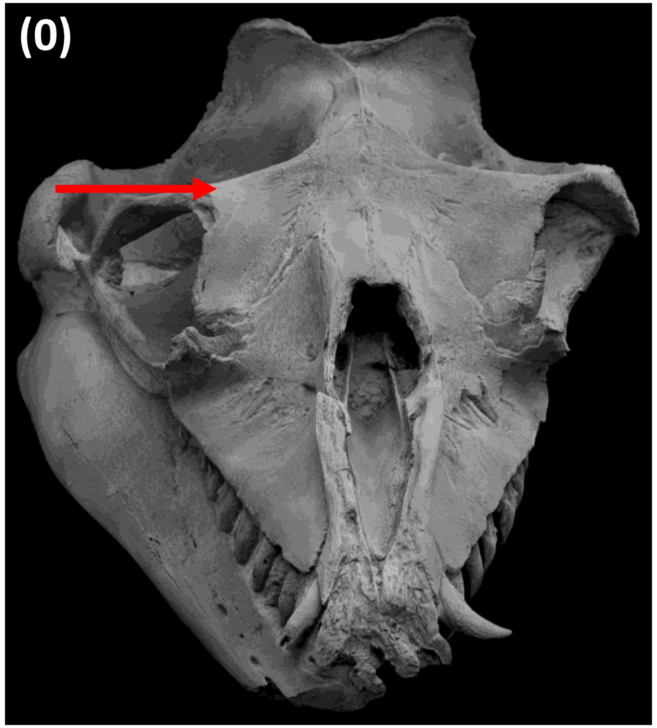

Janjucetus hunderi

Copyright holder: Erich M. G. Fitzgerald/ Museums Victoria, Melbourne, Australia

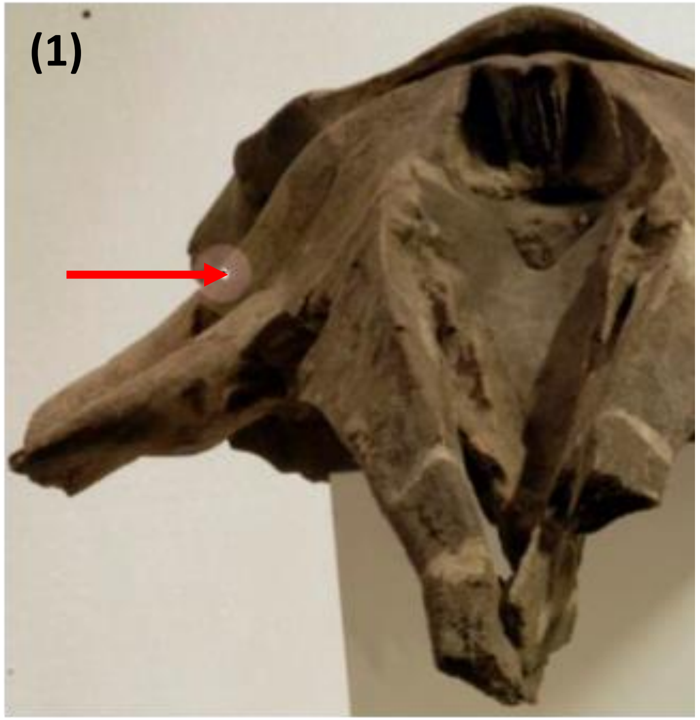

Balaenella brachyrhynus

Copyright holder: Felix G. Marx/Natuurmuseum Brabant, Tilburg, the Netherlands

(2)

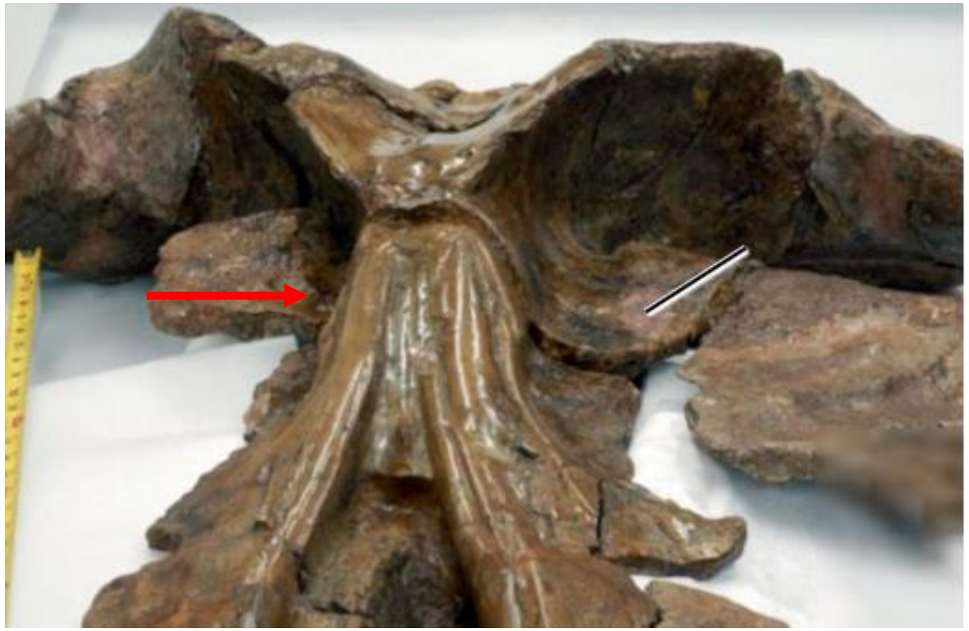

Parabalaenoptera baulinensis

Copyright holder: Felix G. Marx/ California Academy of Sciences, San Francisco, USA

[44] 'Anterior and posterior borders of supraorbital process in dorsal view'

(0)'roughly parallel or converging medially'

(1)'converging laterally'

(0)

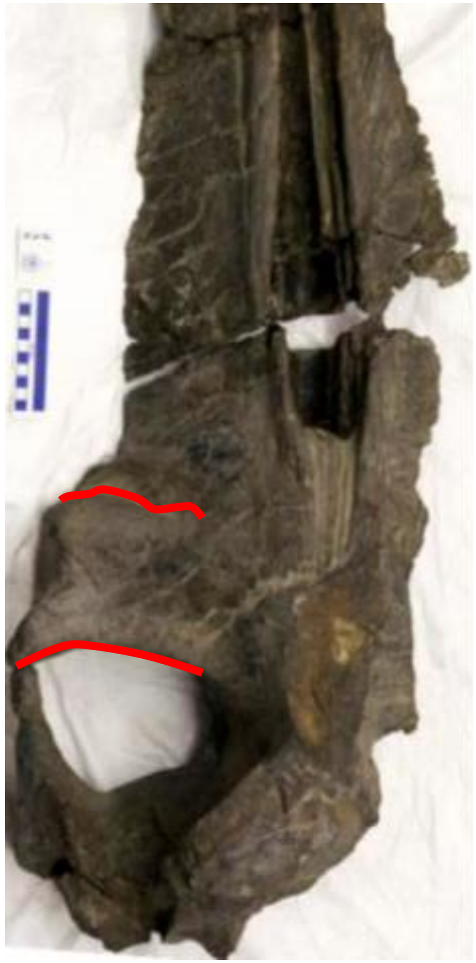

Isanacetus laticephalus

Copyright holder: Felix G. Marx/ Mizunami Fossil Museum, Gifu, Japan

(1)

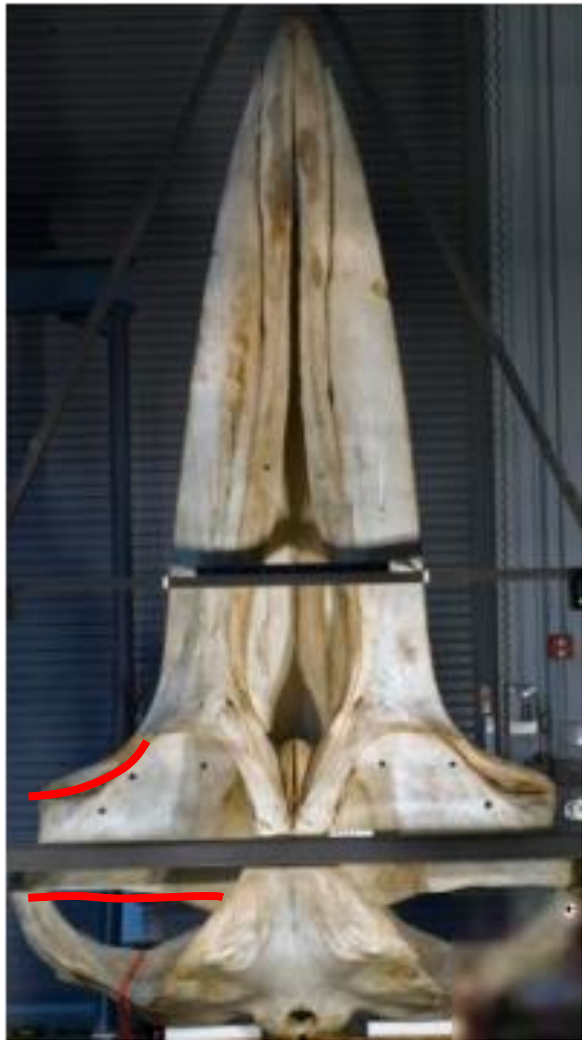

Megaptera novaeangliae

Copyright holder: Felix G. Marx/ United States National Museum of Natural History, Washington DC, USA

[45] 'Width of supraorbital process as measured in a straight line from the lateralmost point of the postorbital process to the intertemporal constriction'

(0)'equal to or shorter than the diameter above the orbit’

(1)'up to twice the length above the orbit’

(2)'more than twice the length above the orbit'

(0)

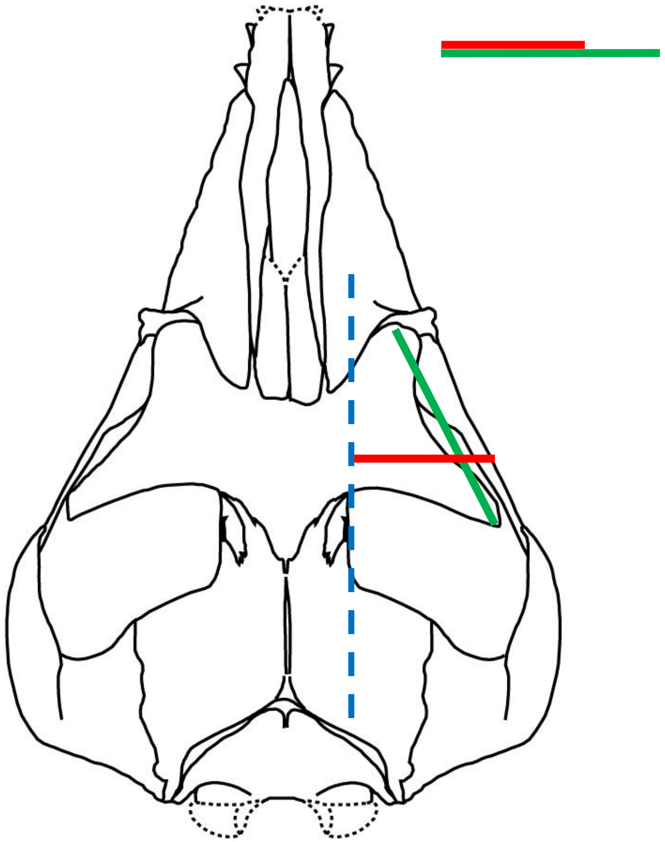

Mammalodon colliveri

Copyright holder: Erich M. G. Fitzgerald/ Museums Victoria, Melbourne, Australia

(1)

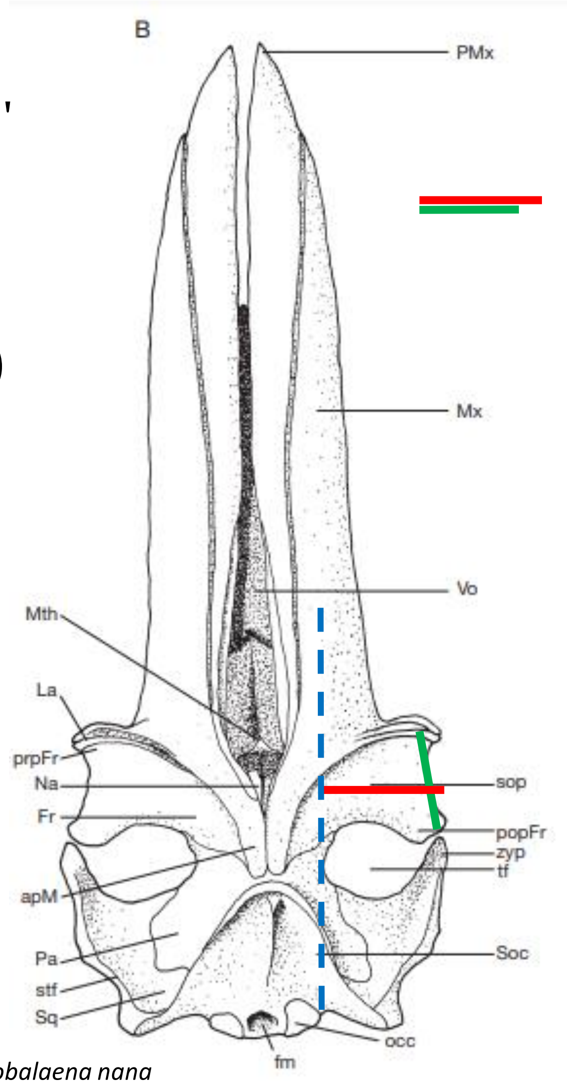

Piscobalaena nana

Adapted from: “The anatomy and relationships of *Piscobalaena nana* (Cetacea, Mysticeti), a Cetotheriidae s.s. from the early Pliocene of Peru”, Bouetel and Muizon, 2006. *Geodiversitas* 28.2 (2006): 319-395.

(2)

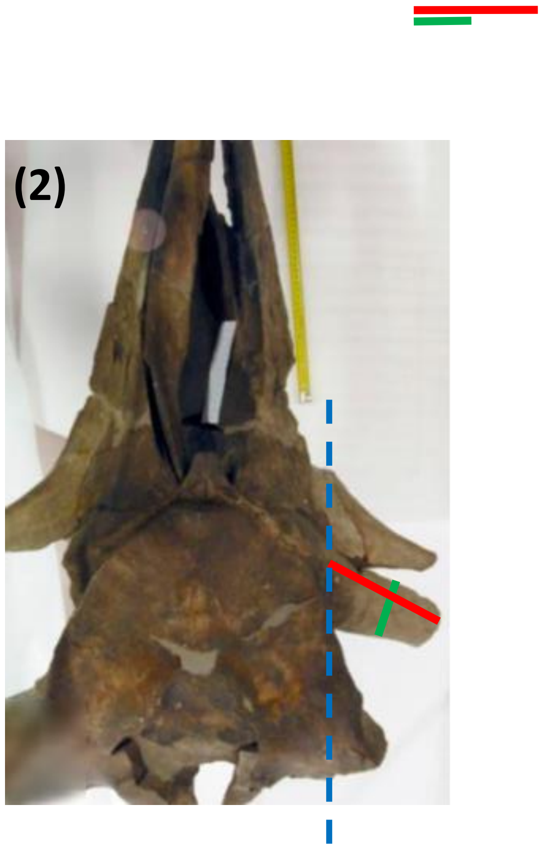

Balaenella brachyrhynus

Copyright holder: Felix G. Marx/Natuurmuseum Brabant, Tilburg, the Netherlands

[46] 'Postorbital process in dorsal view'

(0)'oriented posteriorly'

(1)'oriented posterolaterally'

(2)'oriented laterally'

(3)'short and not markedly projecting in any direction'

(0)

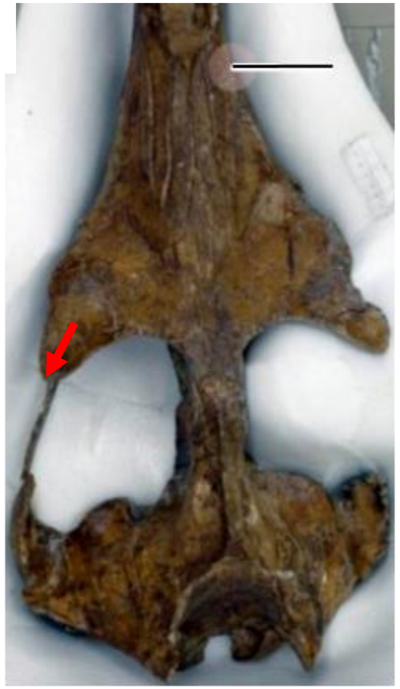

Zygorhiza kochii

Copyright holder: Felix G. Marx/ United States National Museum of Natural History, Washington DC, USA

(1)

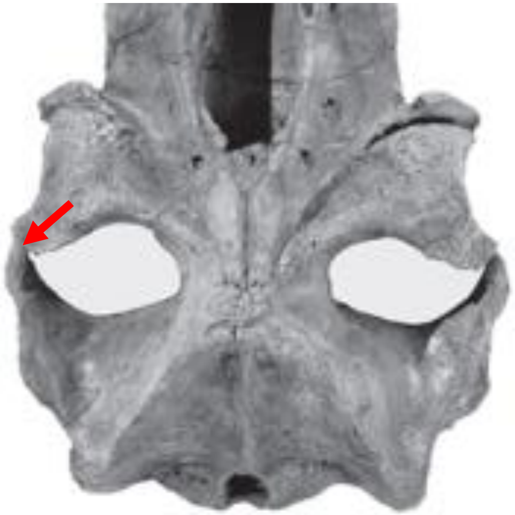

Piscobalaena nana

Adapted from: "The anatomy and relationships of *Piscobalaena nana* (Cetacea, Mysticeti), a Cetotheriidae s.s. from the early Pliocene of Peru", Bouetel and Muizon, 2006. *Geodiversitas* 28.2 (2006): 319-395.

(2)

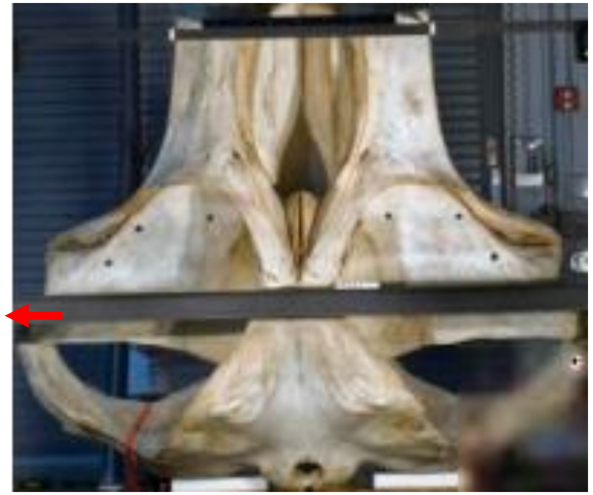

Megaptera novaeangliae

Copyright holder: Felix G. Marx/ United States National Museum of Natural History, Washington DC, USA

(3)

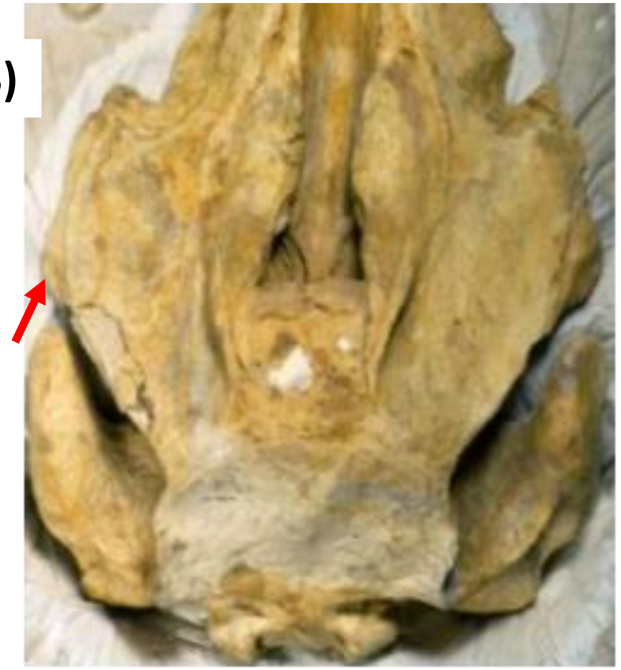

Waipatia maerewhenua

Copyright holder: Felix G. Marx/ University of Otago Museum of Geology, Dunedin, New Zealandc

[47] 'Postorbital process in lateral view'

(0) 'pointed or rounded’

(1) 'forms and anteroposteriorly elongate triangle with a flattened posterior face'

(0)

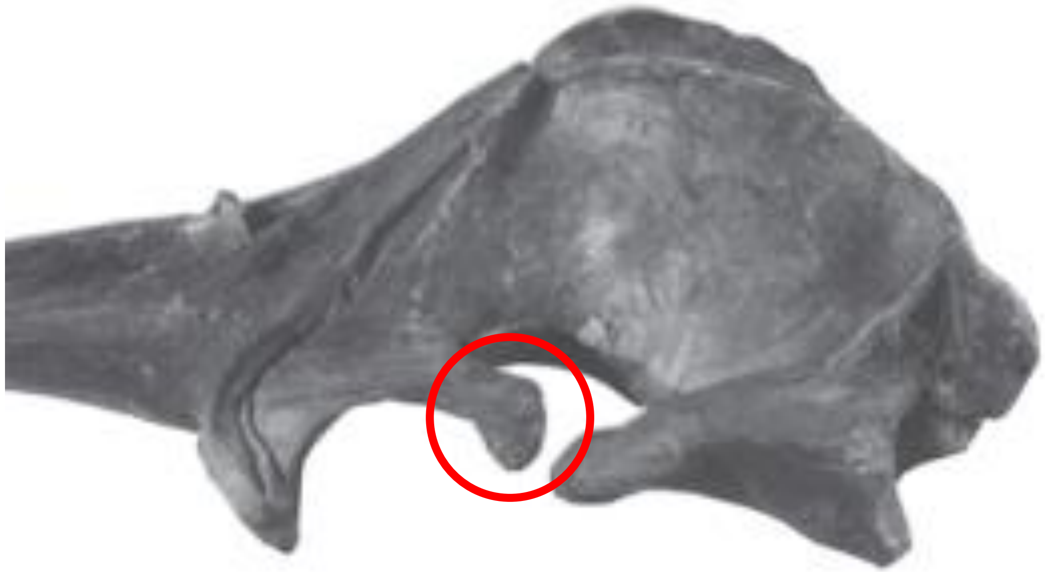

Piscobalaena nana

Adapted from: “The anatomy and relationships of *Piscobalaena nana* (Cetacea, Mysticeti), a Cetotheriidae s.s. from the early Pliocene of Peru”, Bouetel and Muizon, 2006. *Geodiversitas* 28.2 (2006): 319-395.

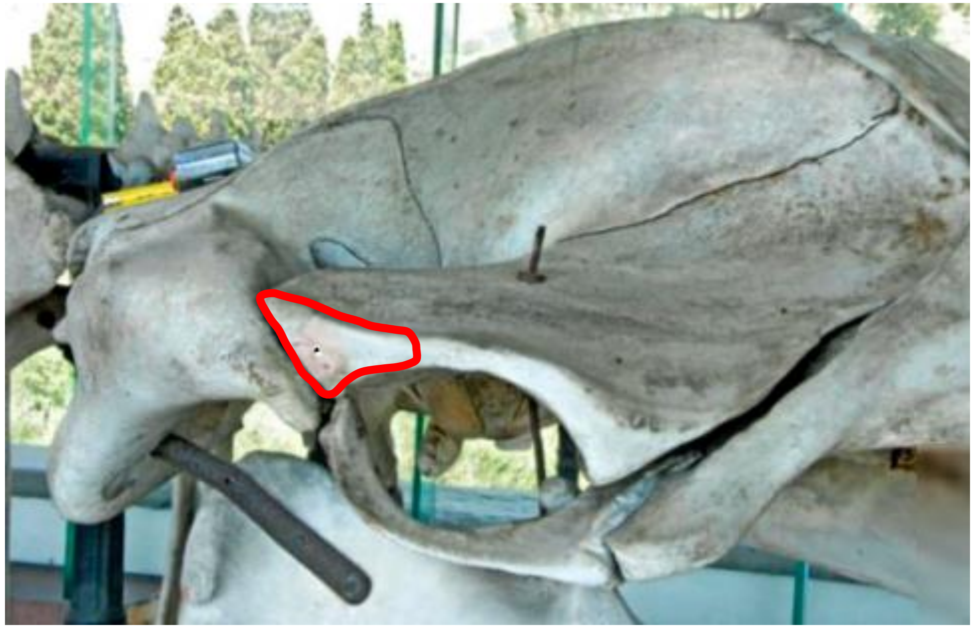

Balaenoptera acutorostrata

Copyright holder: Felix G. Marx/ Museo di Storia Naturale e del Territorio, Università di Pisa, Calci, Italy

[48] 'Orbital rim of supraorbital process of frontal in lateral view'

(0) 'dorsoventrally thin'

(1) 'thickened with a flat lateral surface'

(2) 'thickened with a rounded lateral surface'

(0)

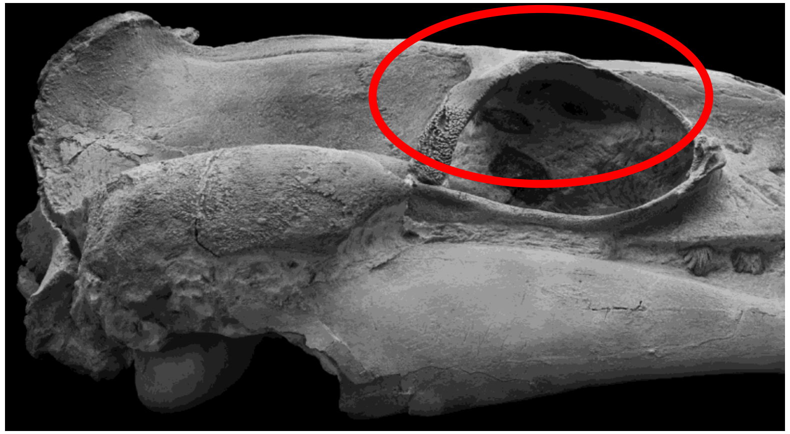

Janjucetus hunderi

(1)

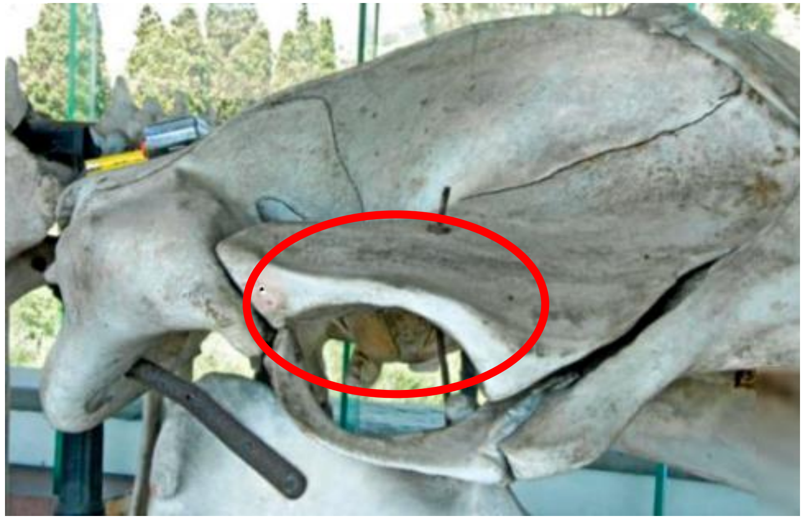

Balaenoptera acurostrata

(2)

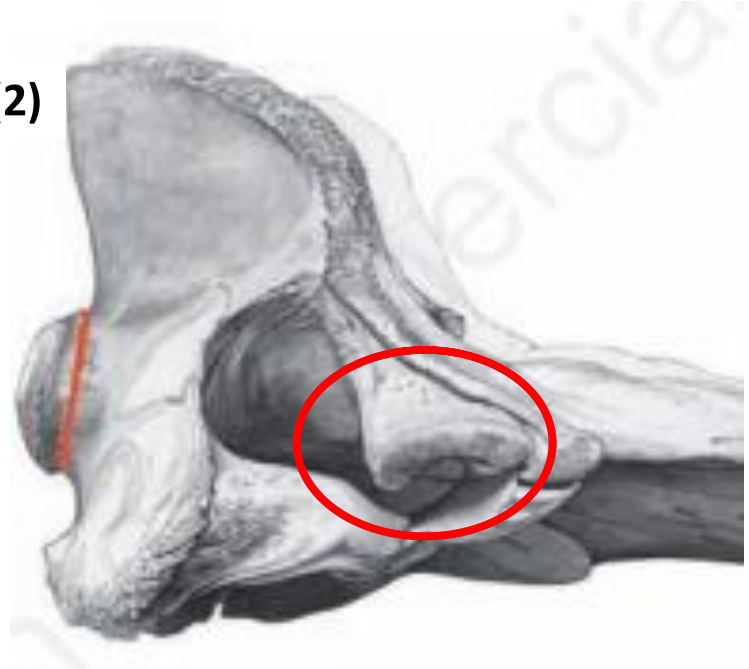

Physeter macrocephalus

Copyright holder: Erich M. G. Fitzgerald/ Museums Victoria, Melbourne, Australia

Copyright holder: Felix G. Marx/ Museo di Storia Naturale e del Territorio, Università di Pisa, Calci, Italy

Adapted from: "Notes on the brain and encephalization quotient of two sperm whales with a synthesis of the literature and indications of a new method of extraction." Povinelli et al., 2014. *Natural History Sciences* 1.2: 131-138.
Author of the drawing: Massimo Demma

[49] 'Position of anteriormost point of supraorbital process in dorsal view'

(0) 'in line with the posterior extremity of the nasals or passing through the nasals'

(1) 'at the same level as the anterior extremity of the nasals'

(2) 'anterior to the anterior extremity of the nasals'

(0)

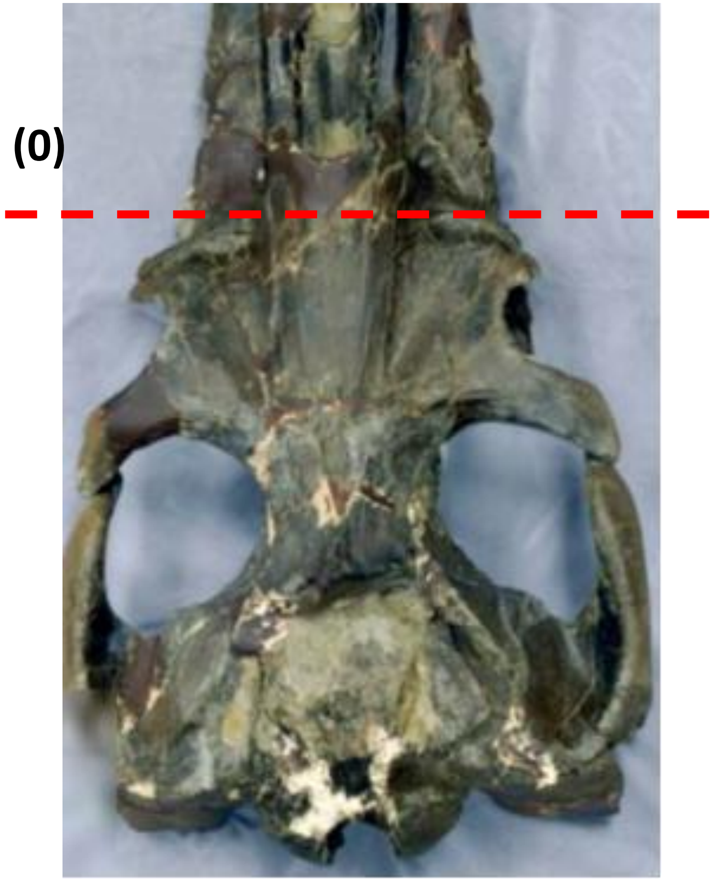

Aetiocetus weltoni

Copyright holder: Felix G. Marx/ University of California Museum of Paleontology, Berkeley, USA

(1)

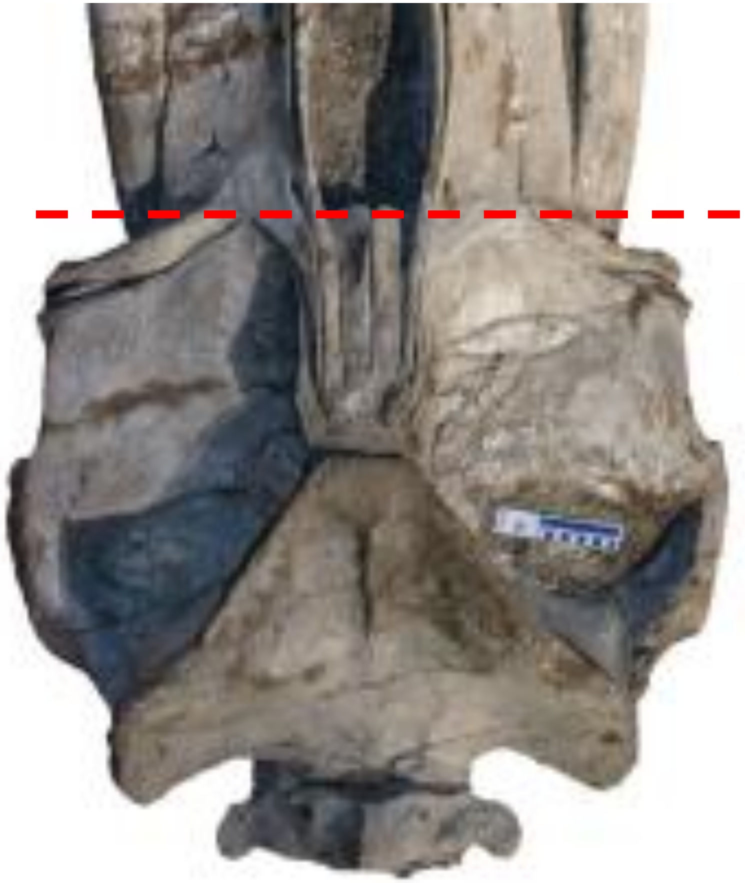

Incakujira anillodefuego

Adapted from: "A new Miocene baleen whale from the Peruvian desert." Marx and Kohno, 2016. *Royal Society Open Science* 3.10 (2016): 160542.

(2)

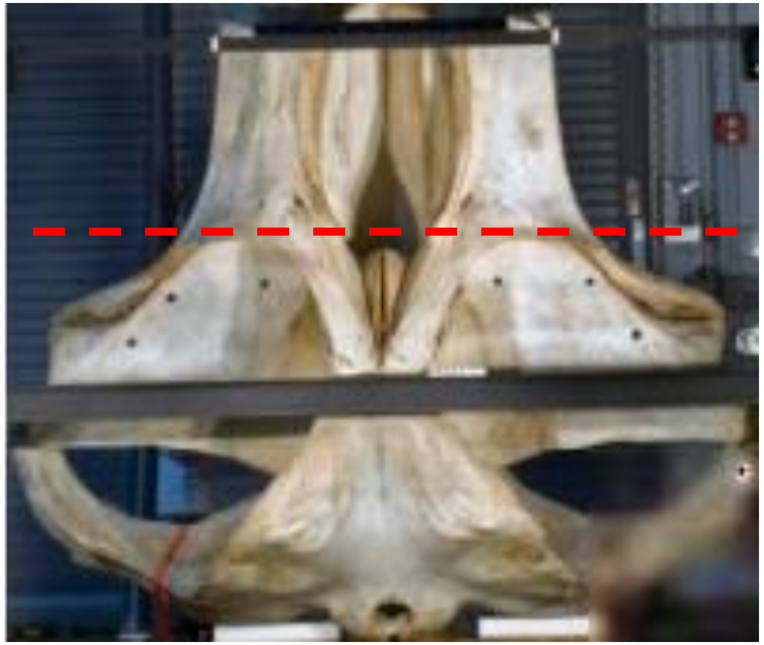

Megaptera novaeangliae

Copyright holder: Felix G. Marx/ United States National Museum of Natural History, Washington DC, USA

[50] 'Dorsalmost point of orbit relative to lateral edge of rostrum in lateral view, with skull resting on a horizontal surface'

(0)'elevated above or roughly in line with the lateral edge of the rostrum'

(1)'located well below the lateral edge of the rostrum'

(0)

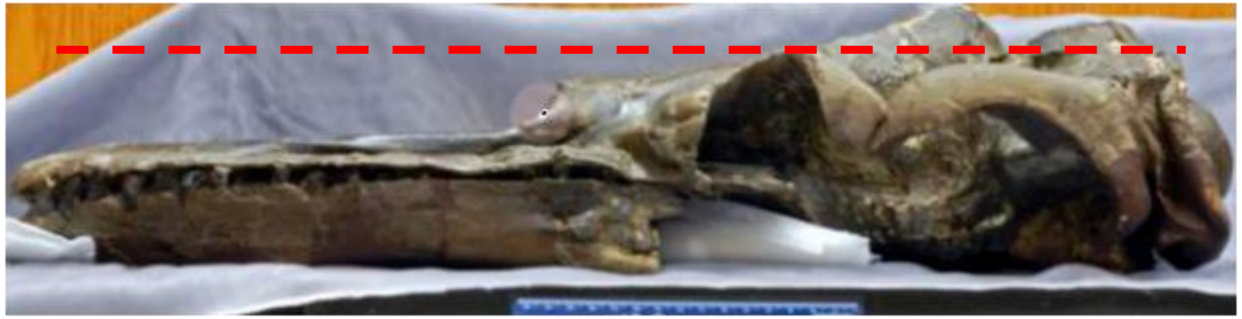

Aetiocetus weltoni

Copyright holder: Felix G. Marx/ University of California Museum of Paleontology, Berkeley, USA

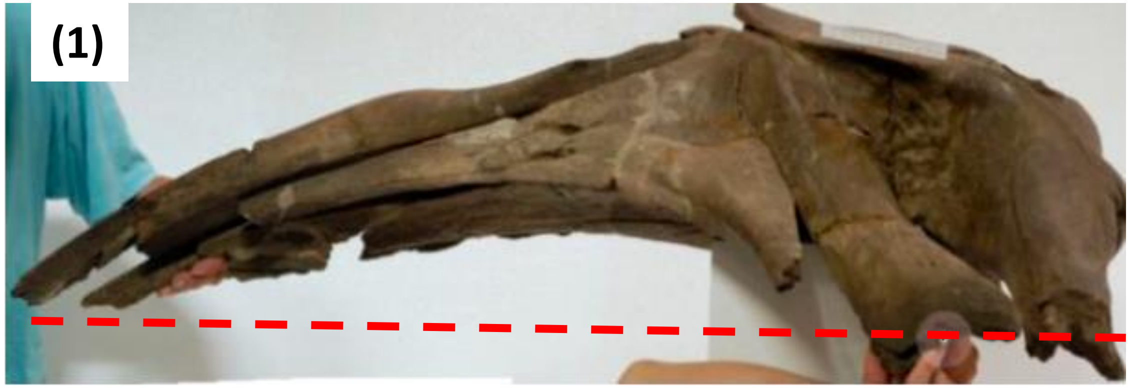

Balaenella brachyrhynus

Copyright holder: Felix G. Marx/Natuurmuseum Brabant, Tilburg, the Netherlands

[51] 'Lacrimal in dorsal view'

(0) 'situated entirely lateral to the ascending process of the maxilla’

(1) 'lacrimal incised into the lateral border of the maxilla’

(2) 'lacrimal enlarged and partially covering the supraorbital process of the frontal'

(0)

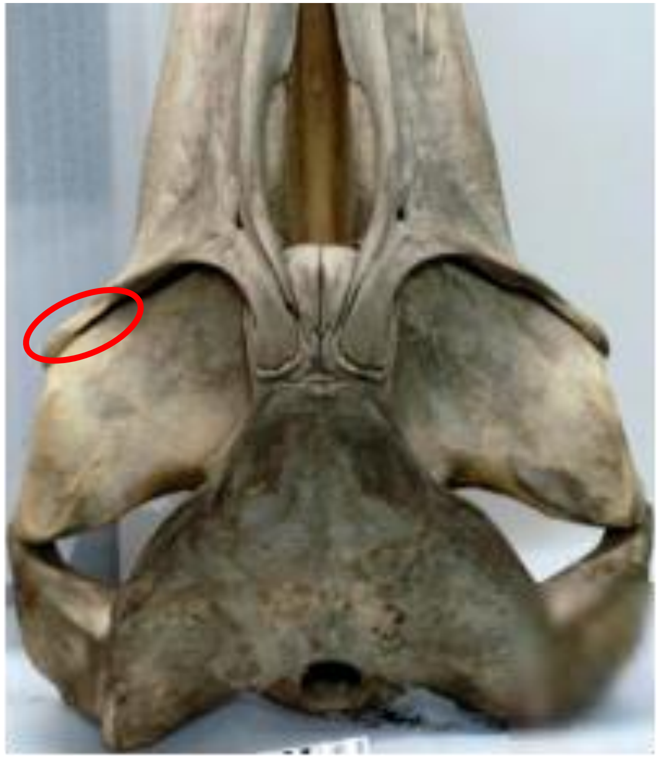

Balaenoptera acutorostrata

Copyright holder: Felix G. Marx/ The Charleston Museum, Charleston, South Carolina, USA

(1)

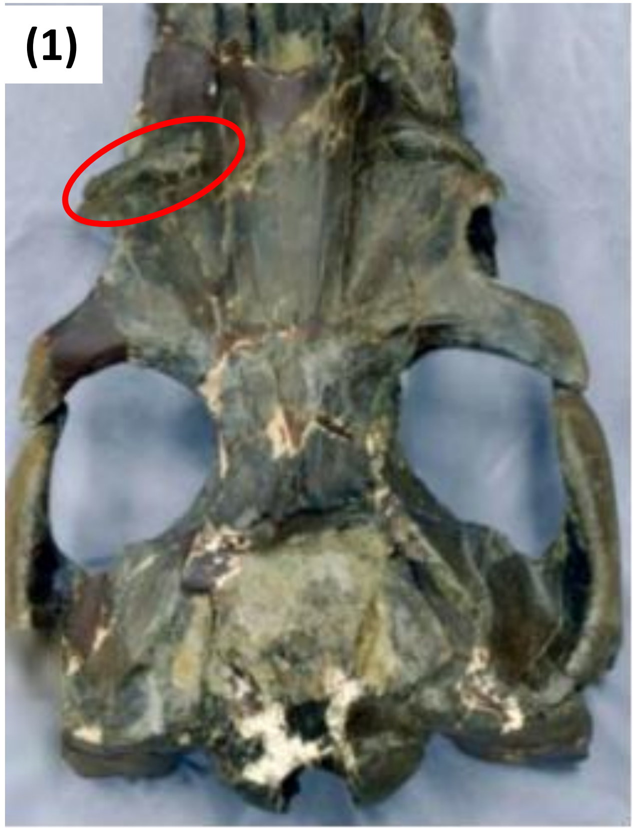

Aetiocetus weltoni

Copyright holder: Felix G. Marx/ University of California Museum of Paleontology, Berkeley, USA

(2)

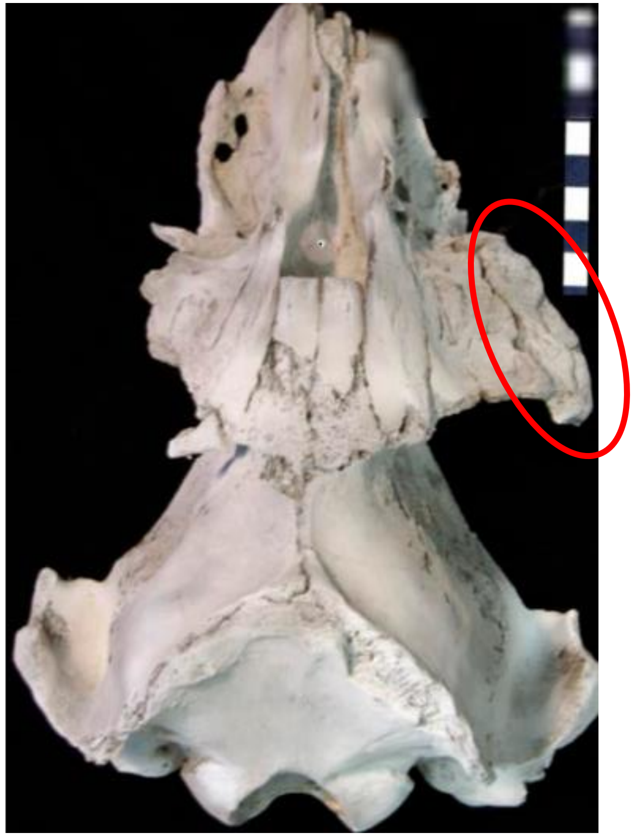

Albertocetus meffordorum

Copyright holder: Mark D. Uhen/ United States National Museum of Natural History, Washington DC, USA

[52] 'Contact of jugal with zygomatic process of squamosal'

(0)'the two bones overlap dorsoventrally’

(1)'little or no overlap'

(0)

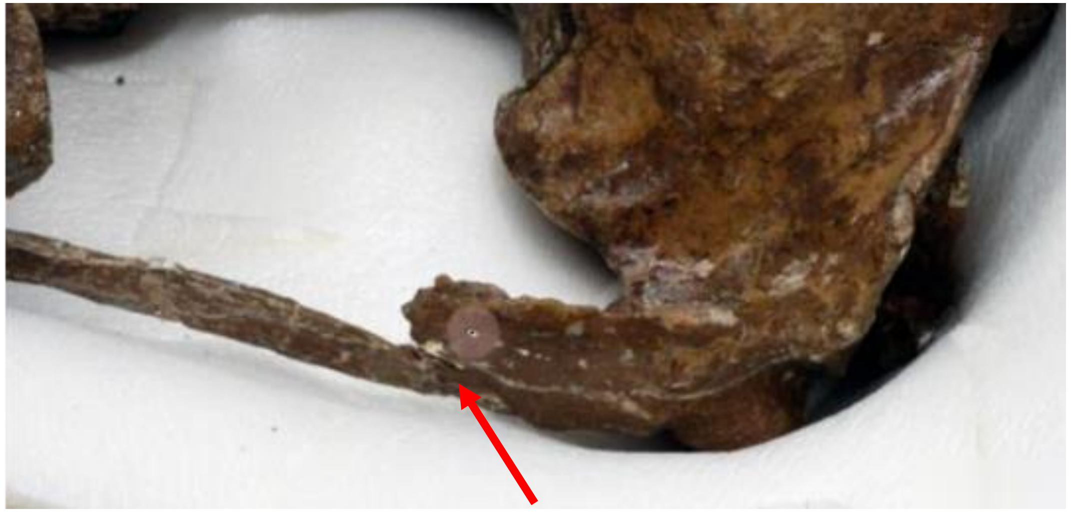

Zygorhiza kochii

(1)

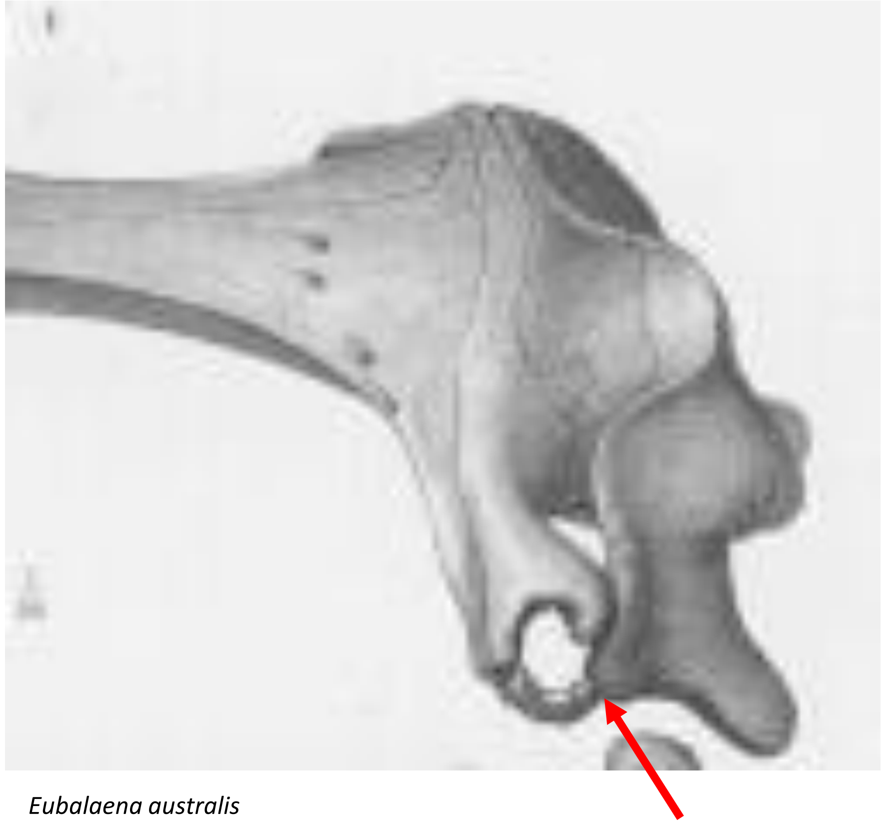

Eubalaena australis

Copyright holder: Felix G. Marx/ United States National Museum of Natural History, Washington DC, USA

Adapted from: “ Ostéographie des cétacés vivants et fossiles: comprenant la description et l'iconographie du squelette et du système dentaire de ces animaux ainsi que des documents relatifs à leur histoire naturelle ”, Beneden, 1880. A. Bertrand.

[53] 'Anteriormost portion of jugal broadly underlapped by maxilla'

(0)'absent'

(1)'present'

(0)

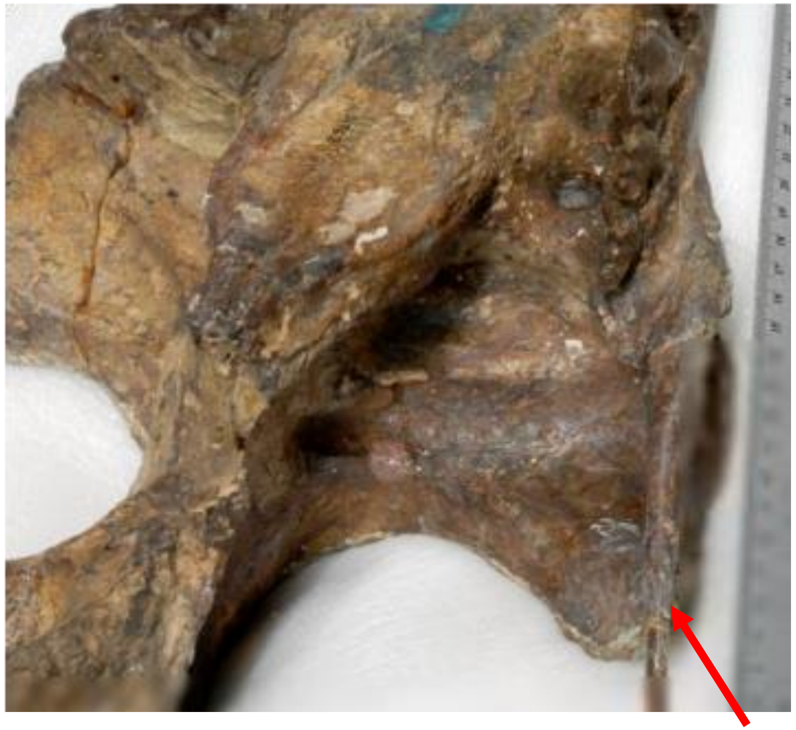

Zygorhiza kochii

(1)

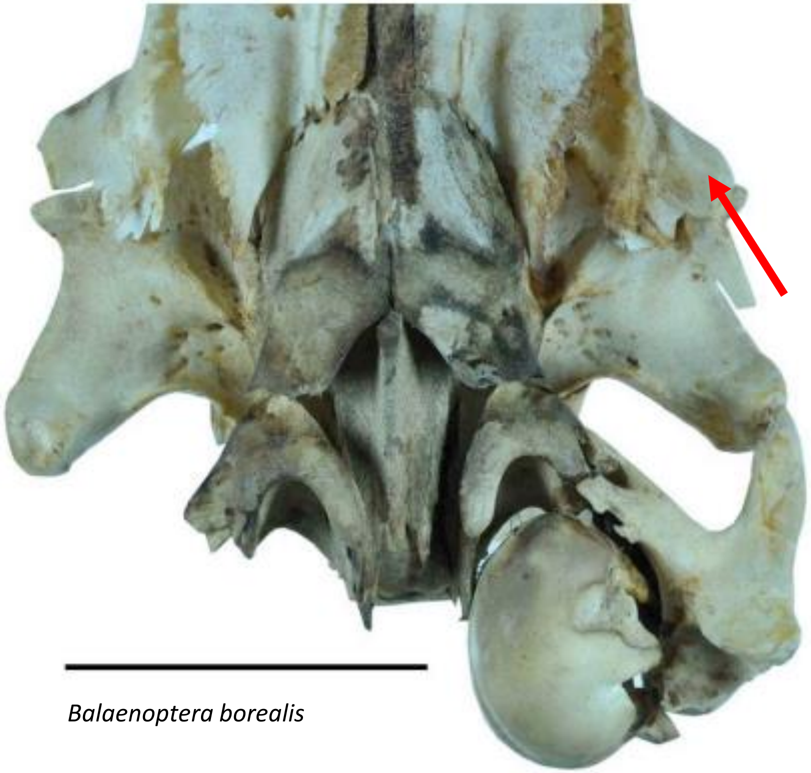

Balaenoptera borealis

Copyright holder: Felix G. Marx/ United States National Museum of Natural History, Washington DC, USA

Adapted from: "Juvenile morphology in baleen whale phylogeny.Tsai, Cheng-Hsiu, and R. Ewan Fordyce, 2014. *Naturwissenschaften* 101.9 : 765-769. "

[54] 'Optic canal in ventral view'

(0) 'ventrally open'

(1) 'medial portion is enclosed by anterior and/or posterior bony laminae'

(0)

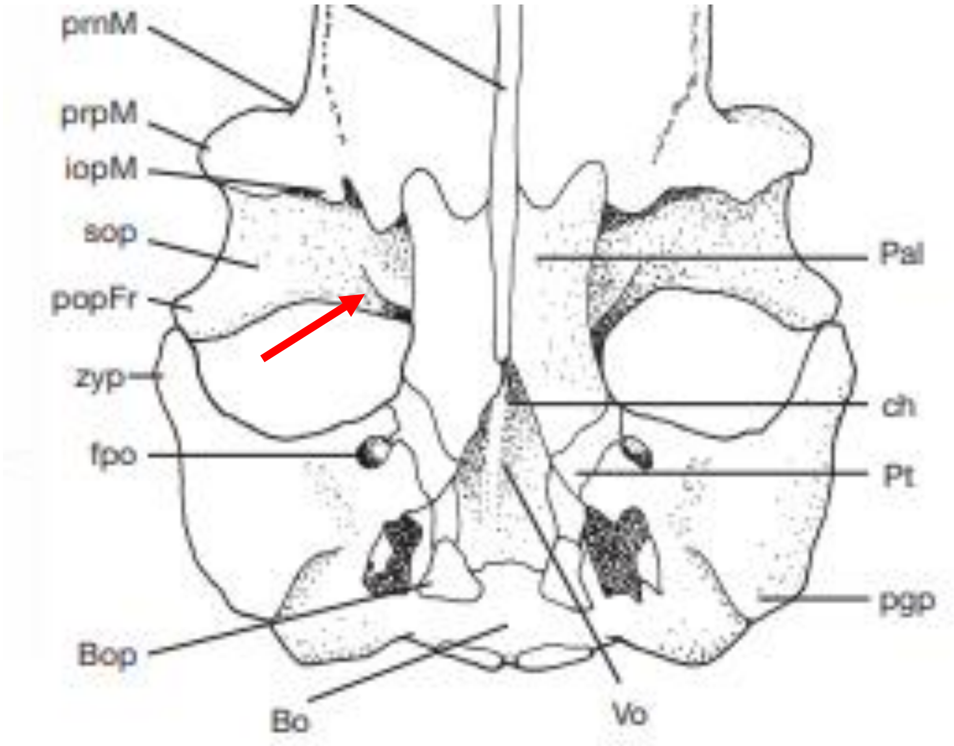

Piscobalaena nana

(1)

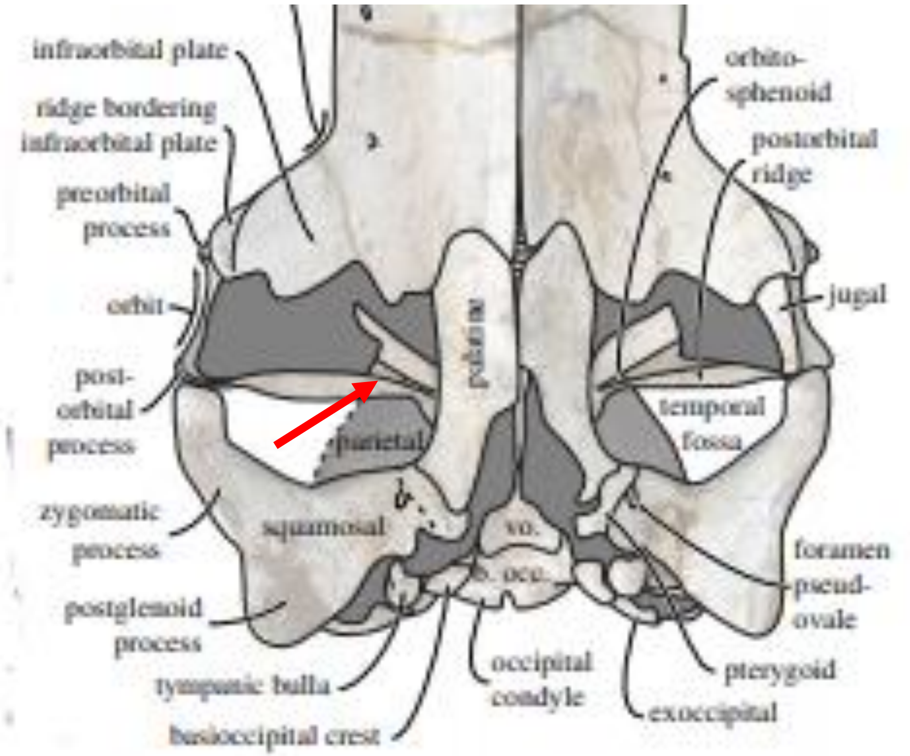

Incakujira anillodefuego

Adapted from: "The anatomy and relationships of *Piscobalaena nana* (Cetacea, Mysticeti), a Cetotheriidae s.s. from the early Pliocene of Peru", Bouetel and Muizon, 2006. *Geodiversitas* 28.2 (2006): 319-395.

Adapted from: "A new Miocene baleen whale from the Peruvian desert." Marx and Kohno, 2016. *Royal Society Open Science* 3.10 (2016): 160542.

[55] 'Medial portion of postorbital ridge'

(0)'absent or anteroposteriorly thin, with the optic canal running adjacent to the posterior border of the supraorbital process'

(1)'well developed and thickened, thus resulting in the displacement of the optic canal away from the posterior border of the supraorbital process'

(0)

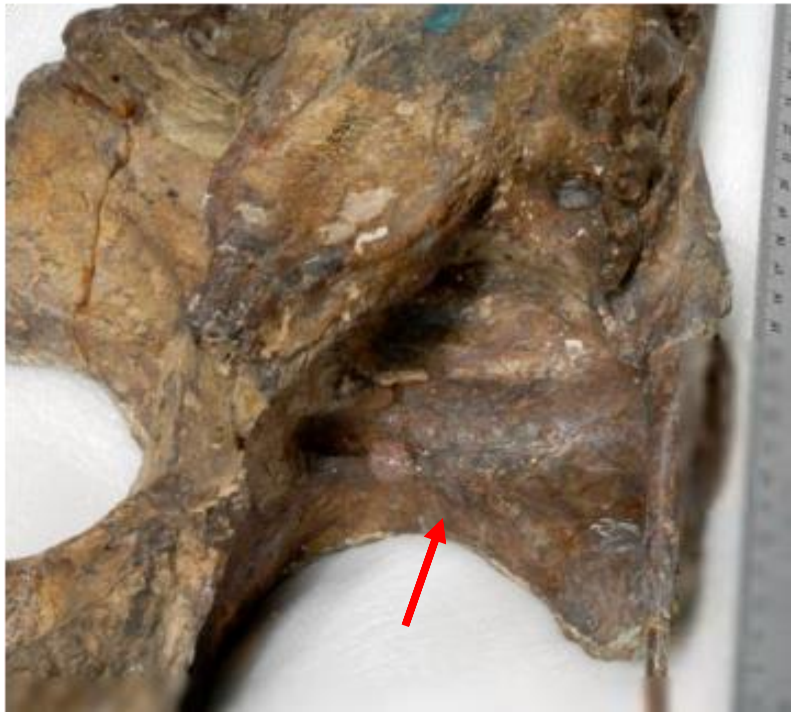

Zygorhiza kochii

Copyright holder: Felix G. Marx/ United States National Museum of Natural History, Washington DC, USA

(1)

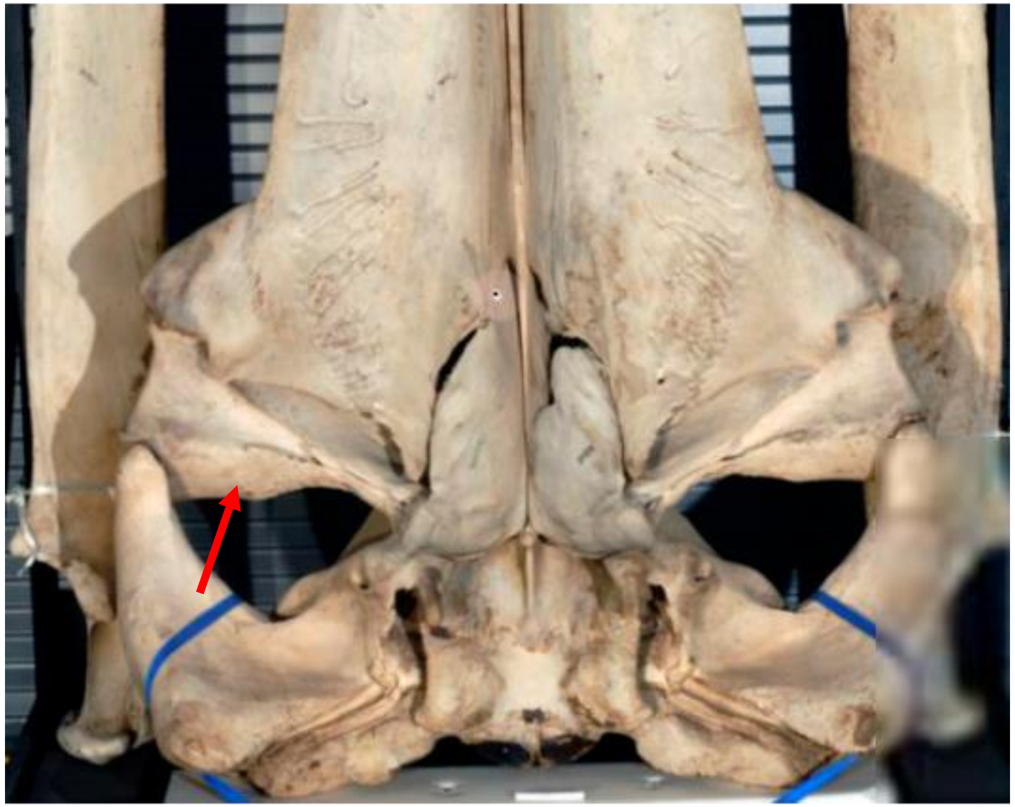

Balaenoptera borealis

Copyright holder: Felix G. Marx/ United States National Museum of Natural History, Washington DC, USA

[56] 'Maxillary infraorbital plate'

(0) 'absent or small'

(1) 'present'

(0)

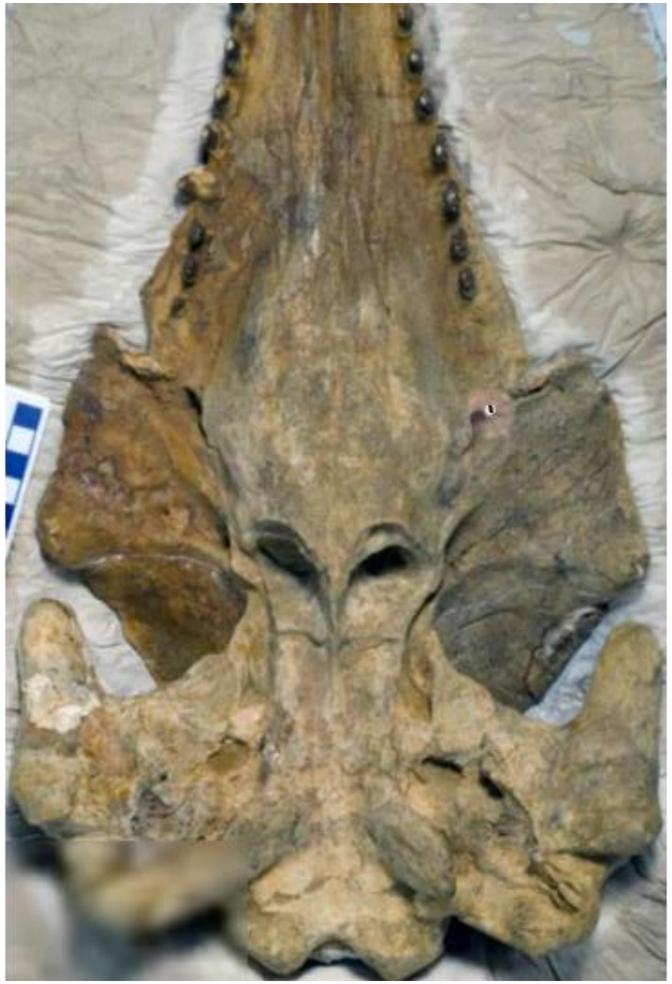

Waipatia maerewhenua

Copyright holder: Felix G. Marx/ University of Otago Museum of Geology, Dunedin, New Zealand

(1)

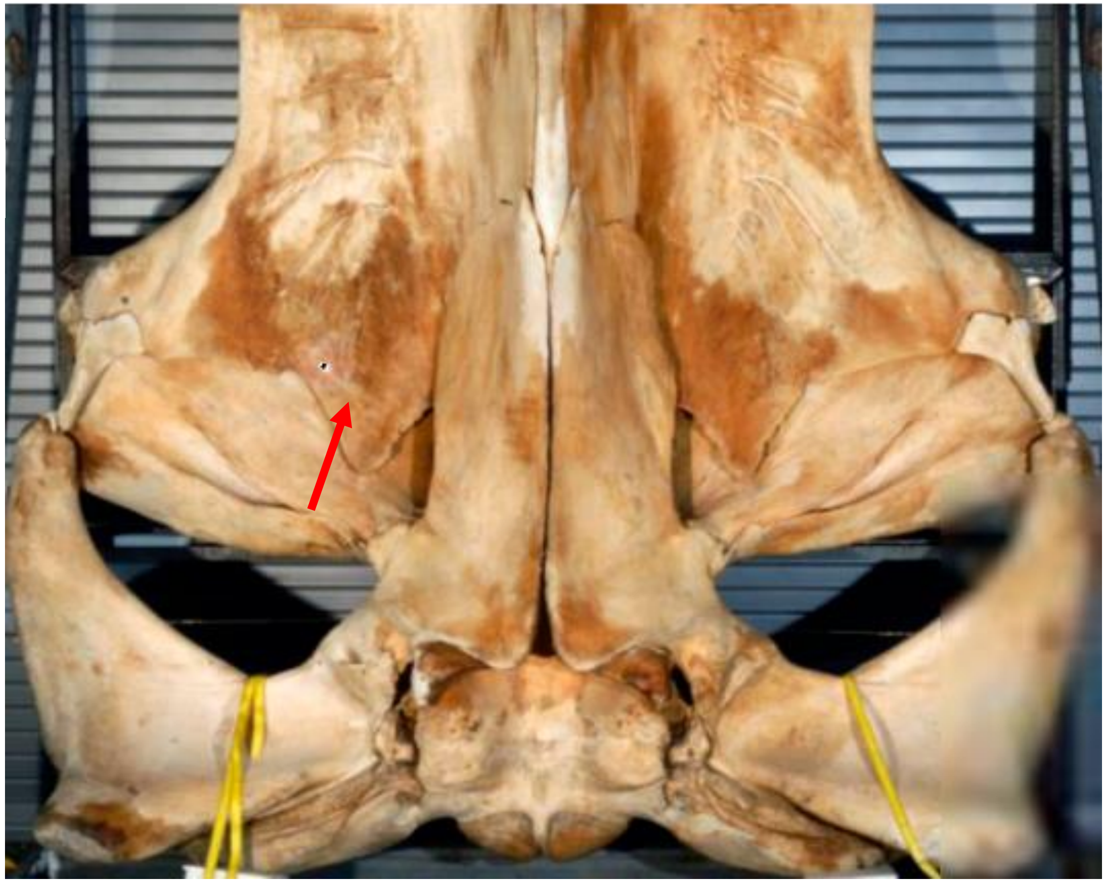

Balaenoptera musculus

Copyright holder: Felix G. Marx/ United States National Museum of Natural History, Washington DC, USA

[57] 'Maxillary window originating from posterior border of infraorbital plate'

(0) 'absent'

(1) 'present'

(0)

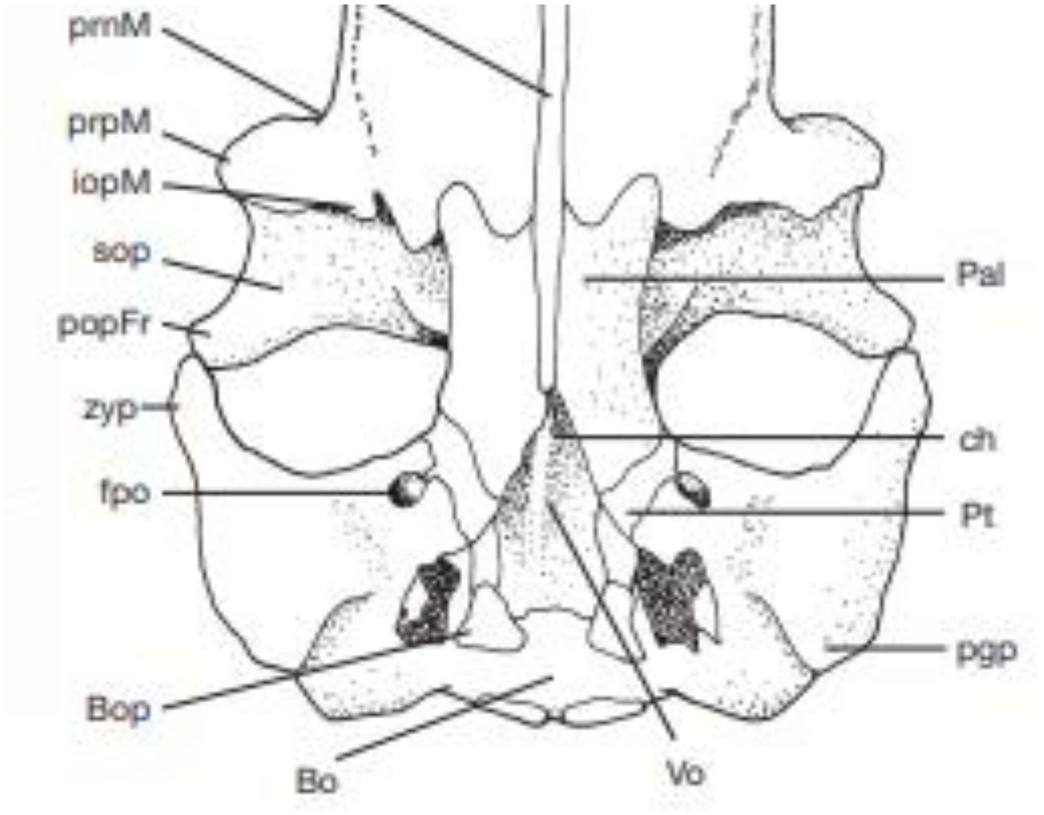

Piscobalaena nana

Adapted from: "The anatomy and relationships of *Piscobalaena nana* (Cetacea, Mysticeti), a Cetotheriidae s.s. from the early Pliocene of Peru", Bouetel and Muizon, 2006. *Geodiversitas* 28.2 (2006): 319-395.

(1)

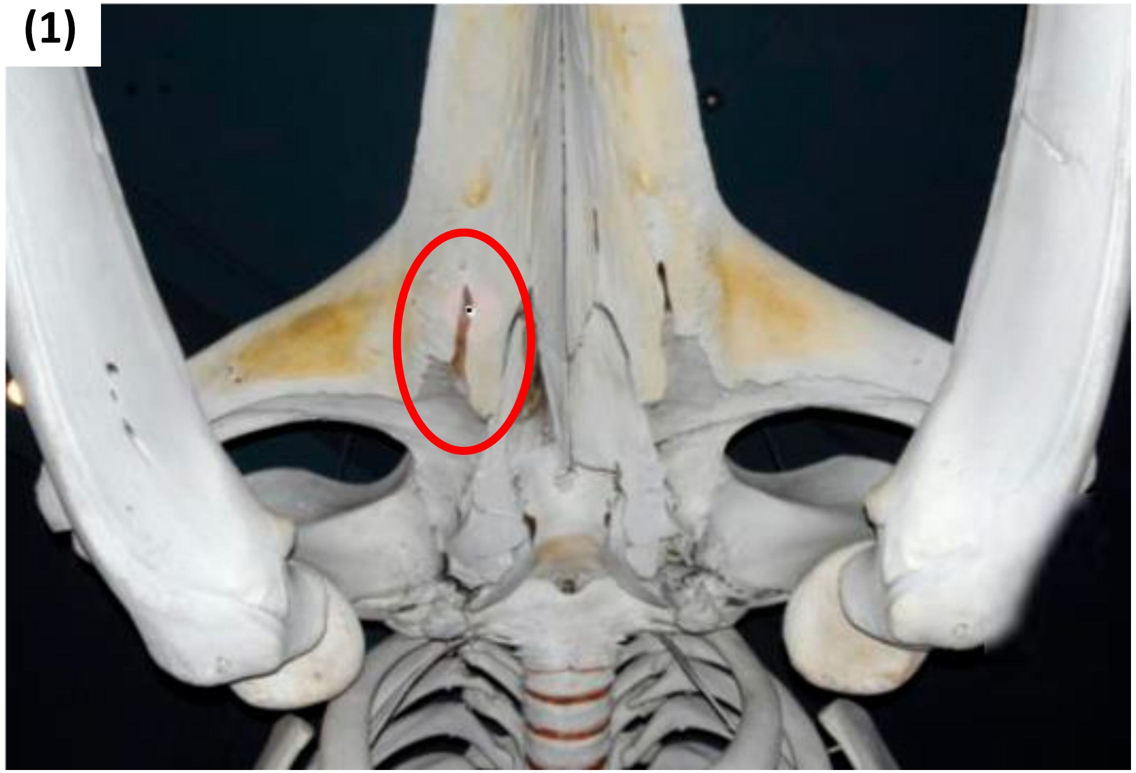

Eubalaena spp.

Copyright holder: Felix G. Marx/ Field Museum of Natural History, Chicago, USA

[58] 'Anteromedial corner of supraorbital process extending to a point medial to antorbital notch'

(0)'absent'

(1)'present'

(0)

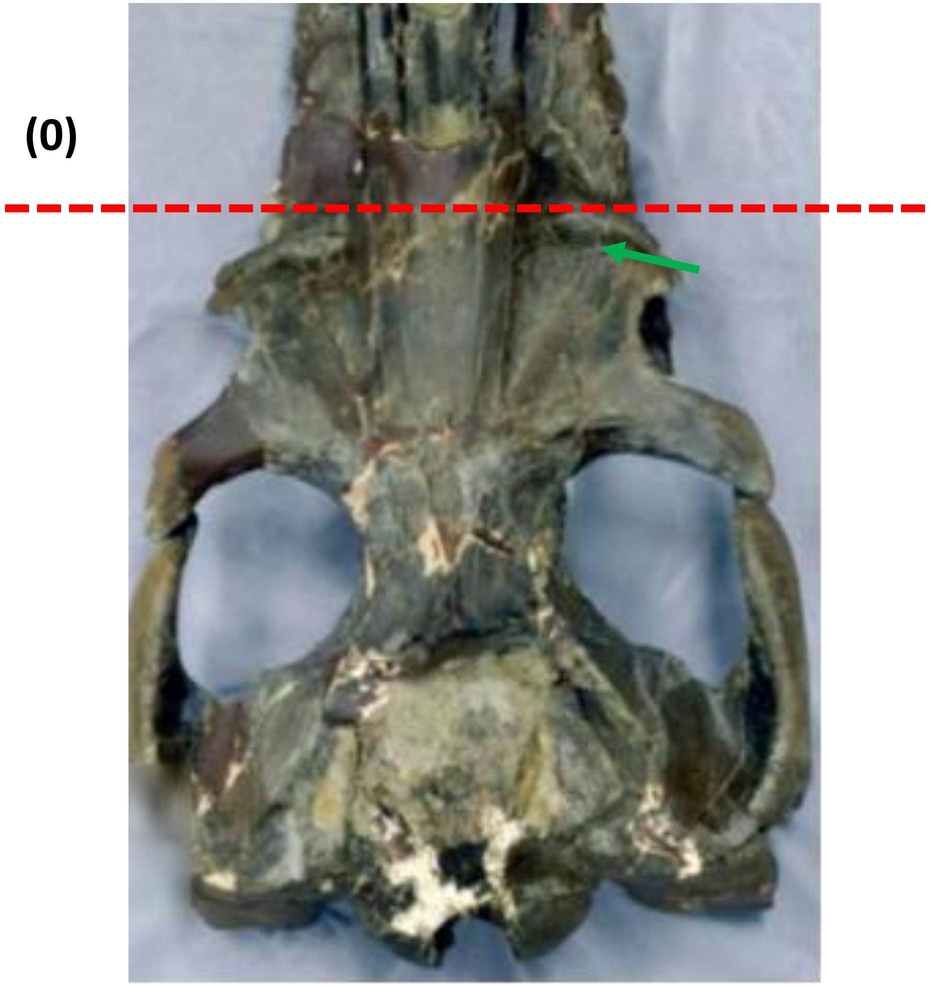

Aetiocetus weltoni

Copyright holder: Felix G. Marx/ University of California Museum of Paleontology, Berkeley, USA

(1)

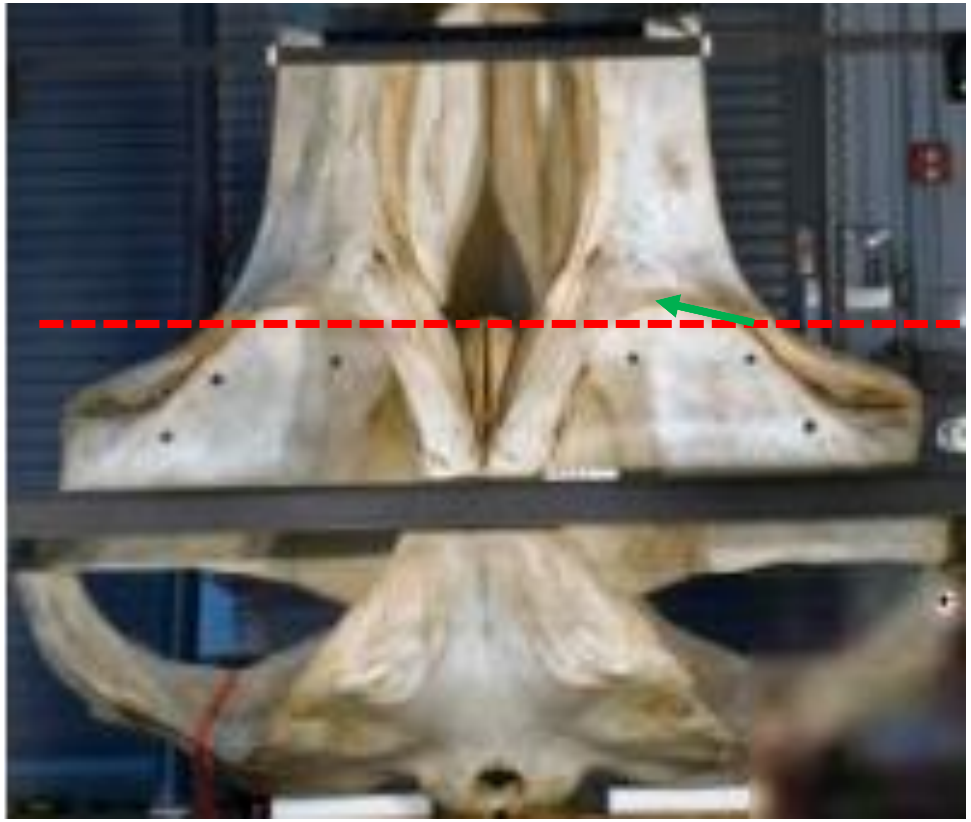

Megaptera novaeangliae

Copyright holder: Felix G. Marx/ United States National Museum of Natural History, Washington DC, USA

[59] 'Preorbital process of frontal in lateral view'

(0) 'thickened compared to more central portions of the orbit'

(1) 'dorsoventrally flat'

(0)

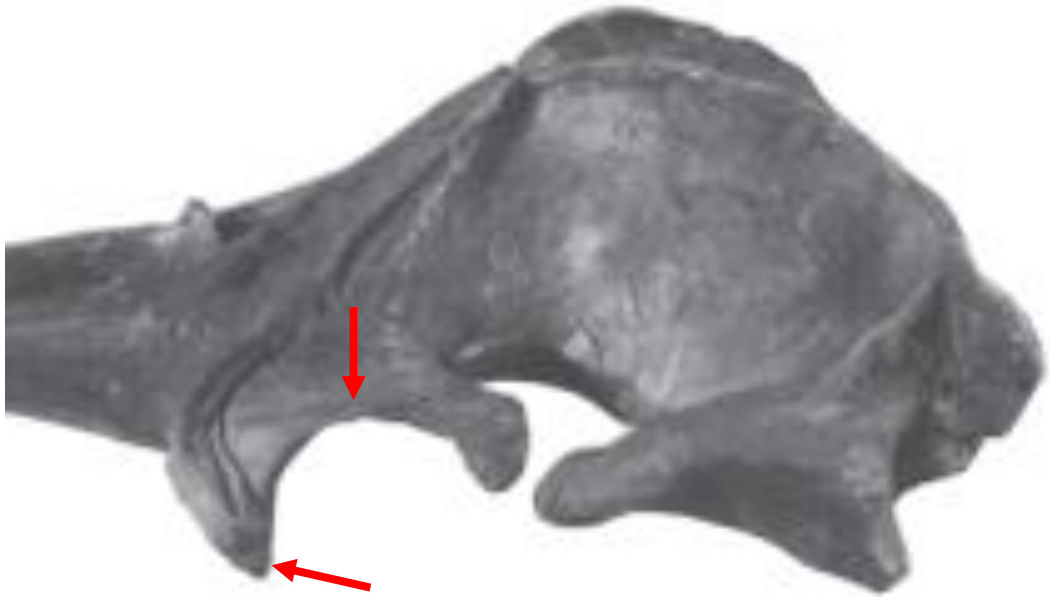

Piscobalaena nana

Adapted from: "The anatomy and relationships of *Piscobalaena nana* (Cetacea, Mysticeti), a Cetotheriidae s.s. from the early Pliocene of Peru", Bouetel and Muizon, 2006. *Geodiversitas* 28.2 (2006): 319-395.

(1)

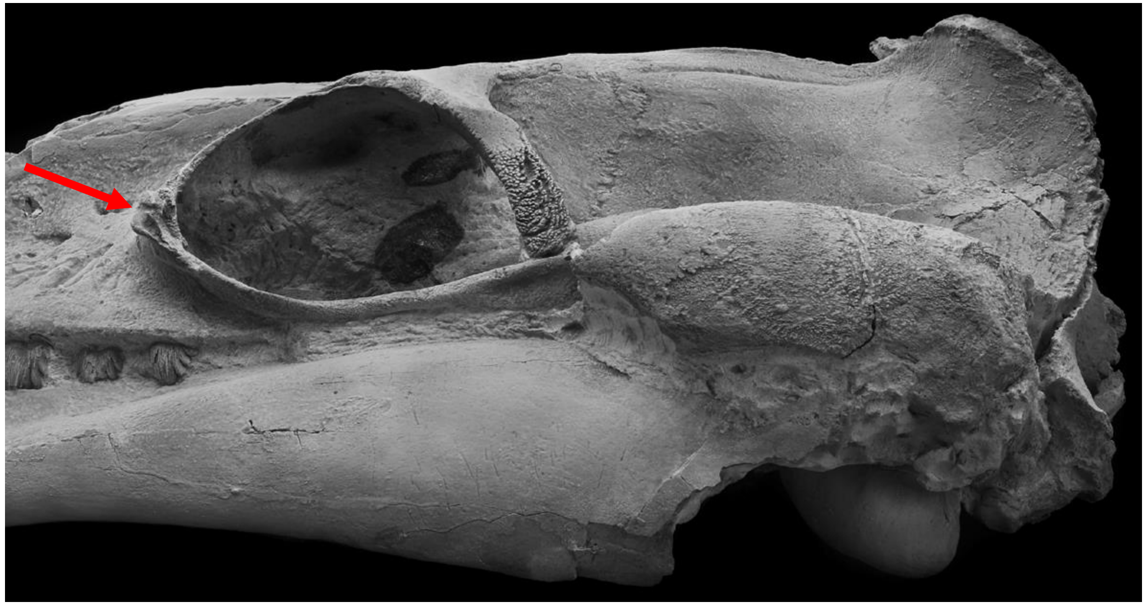

Janjucetus hunderi (mirrored)

Copyright holder: Erich M. G. Fitzgerald/ Museums Victoria, Melbourne, Australia

[60] 'Primary dorsal infraorbital foramen on ascending process of maxilla, opening into a posterodorsally directed sulcus'

(0)'absent'

(1)'present'

(0)

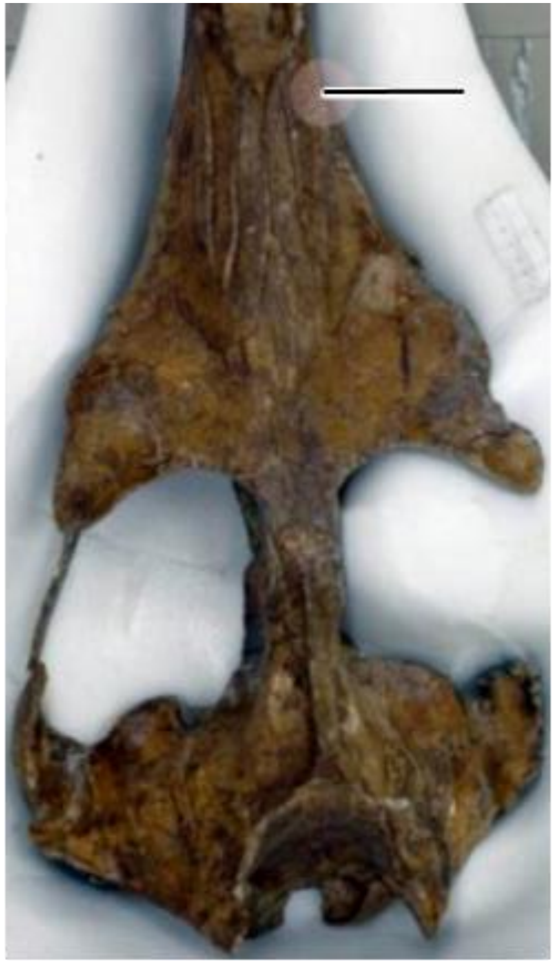

Zygorhiza kochii

Copyright holder: Felix G. Marx/ United States National Museum of Natural History, Washington DC, USA

(1)

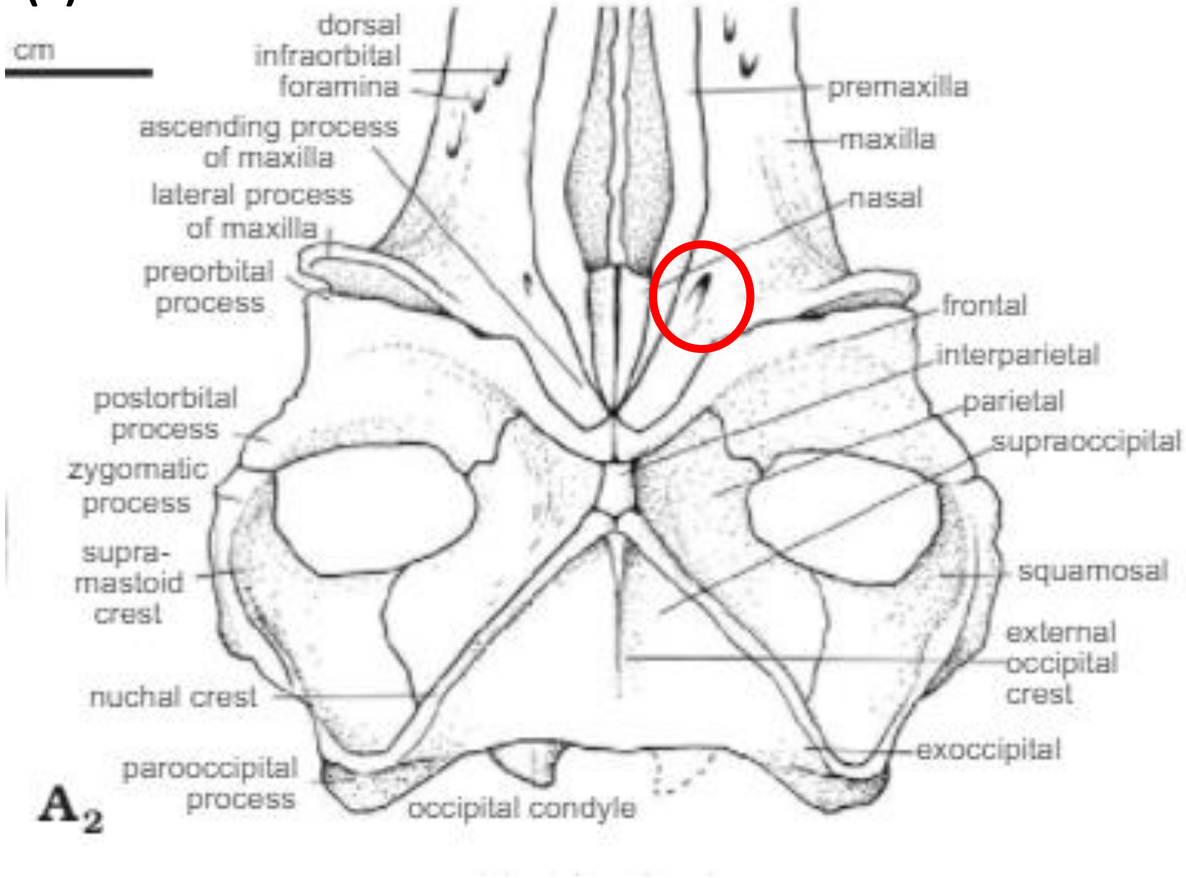

Cetotherium riabinini

Adapted from: "The anatomy of the Late Miocene baleen whale Cetotherium riabinini from Ukraine." Gol'din et al., 2013. *Acta Palaeontologica Polonica* 59.4: 795-814.

[61] 'Premaxillary sac fossa'

(0) 'absent'

(1) 'present'

(0)

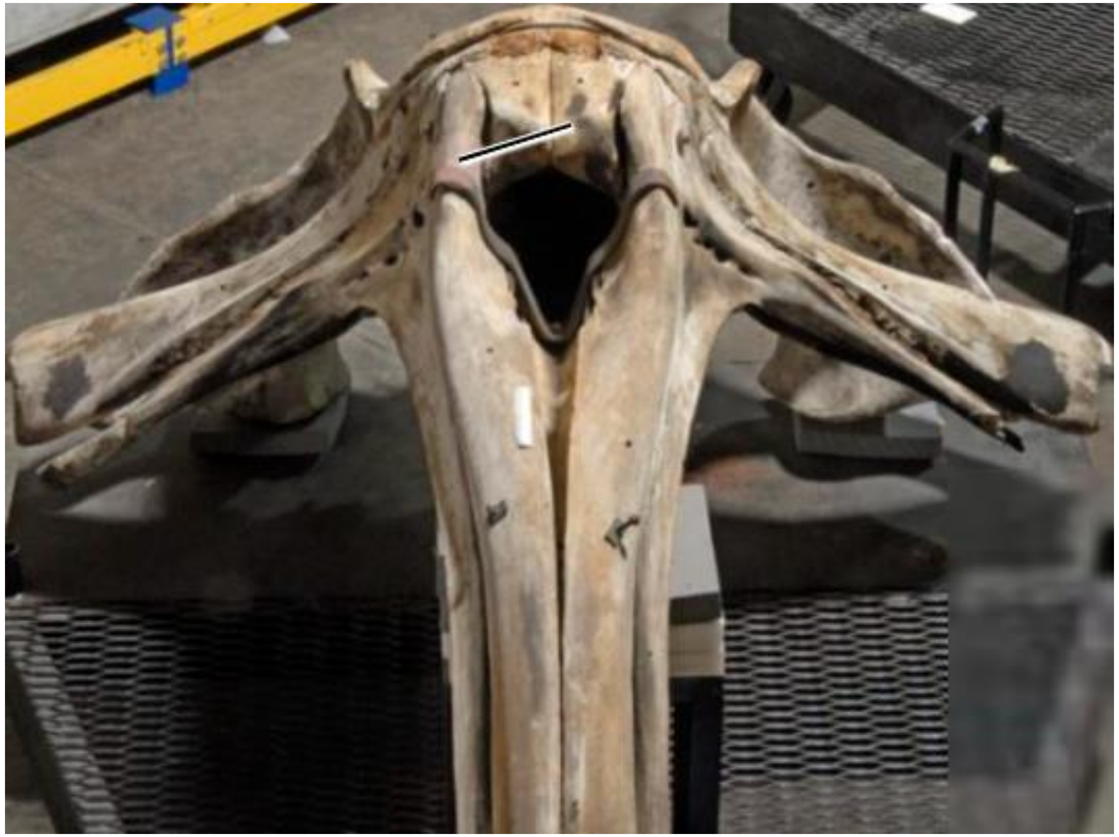

Eubalaena spp. (USNM:267612)

Copyright holder: Felix G. Marx/ United States National Museum of Natural History, Washington DC, USA

(1)

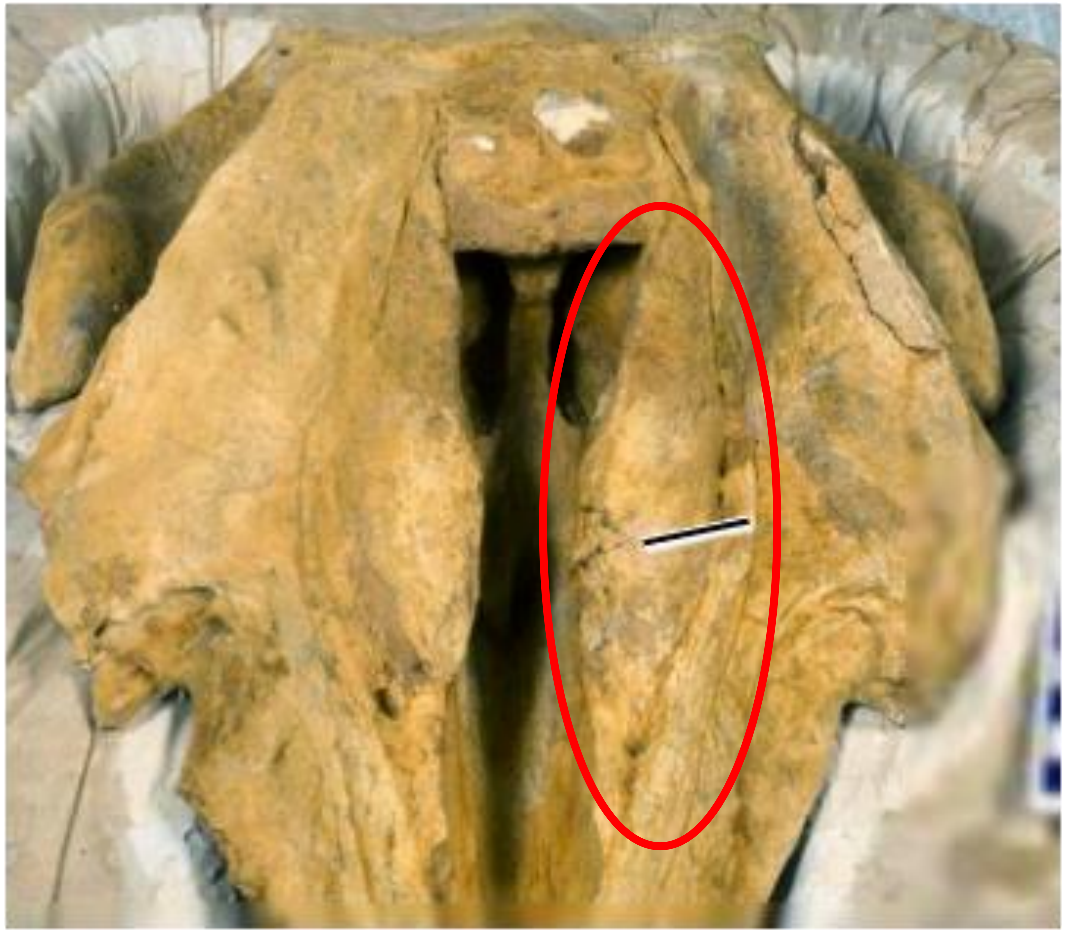

Waipatia maerewhenua

Copyright holder: Felix G. Marx/ University of Otago Museum of Geology, Dunedin, New Zealand

[62] 'Premaxillary foramen'

(0)'absent'

(1)'present'

(0)

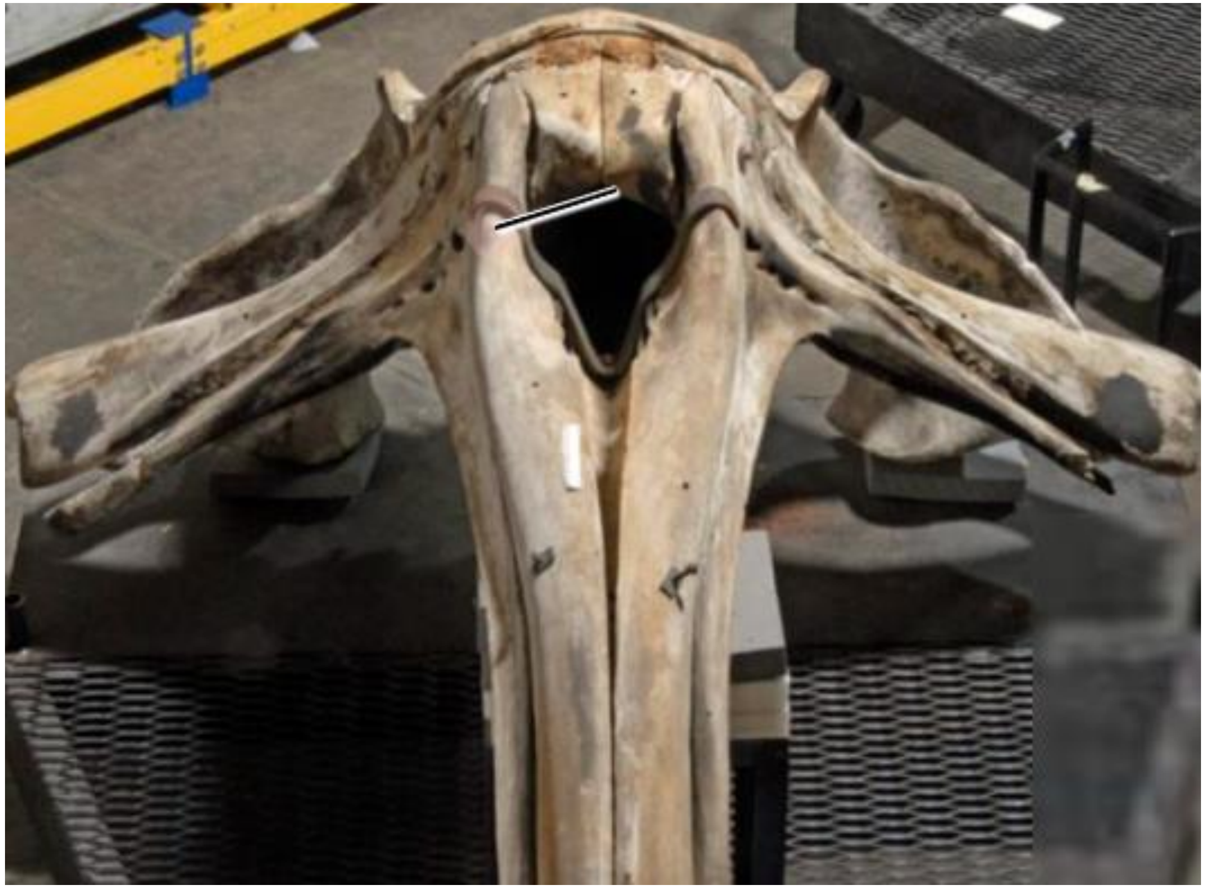

Eubalaena spp. (USNM:267612)

Copyright holder: Felix G. Marx/ United States National Museum of Natural History, Washington DC, USA

(1)

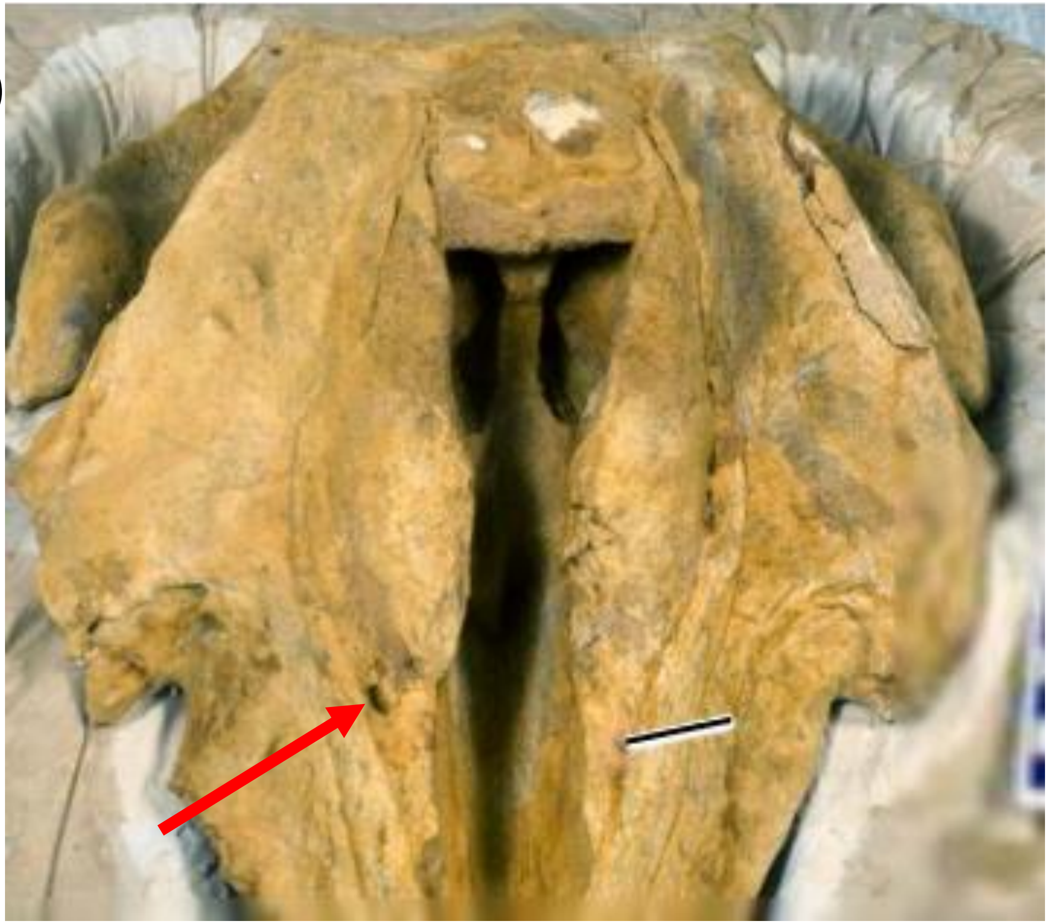

Waipatia maerewhenua

Copyright holder: Felix G. Marx/ University of Otago Museum of Geology, Dunedin, New Zealand

[63] 'Suture between maxilla and frontal'

(0) 'contact between the bones is straight or maxilla overrides anteromedial corner of the frontal'

(1) 'maxilla overrides half or more of the frontal'

(0)

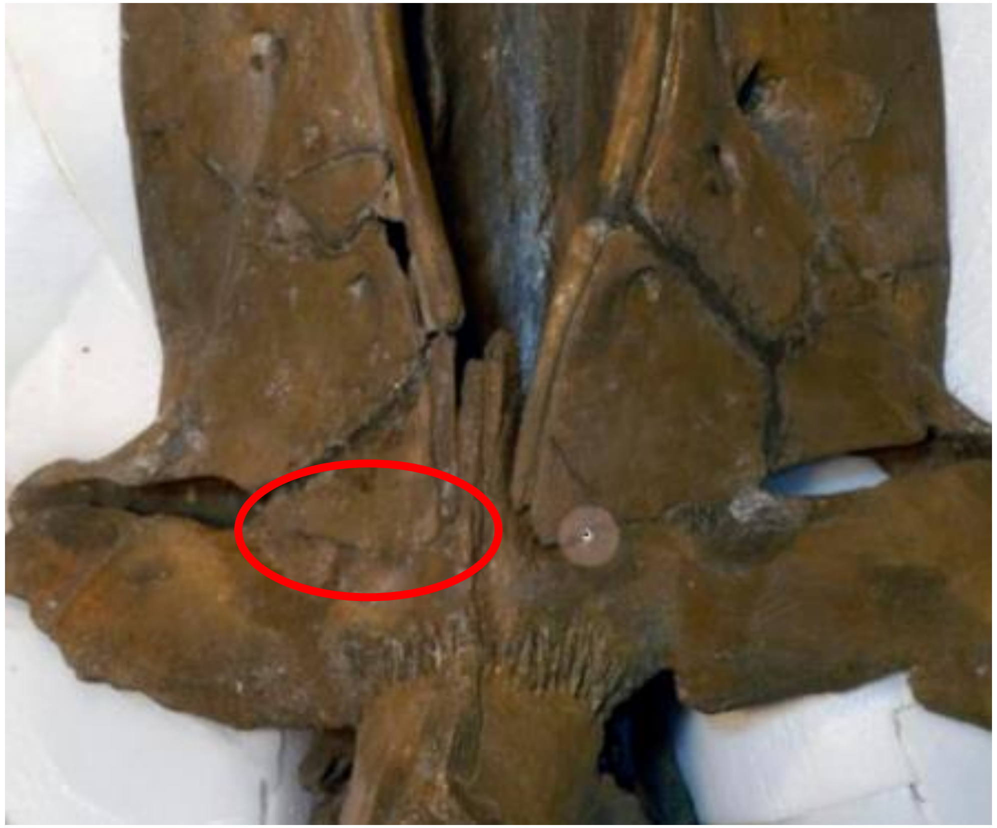

Diorocetus hiatus

Copyright holder: Felix G. Marx/ United States National Museum of Natural History, Washington DC, USA

(1)

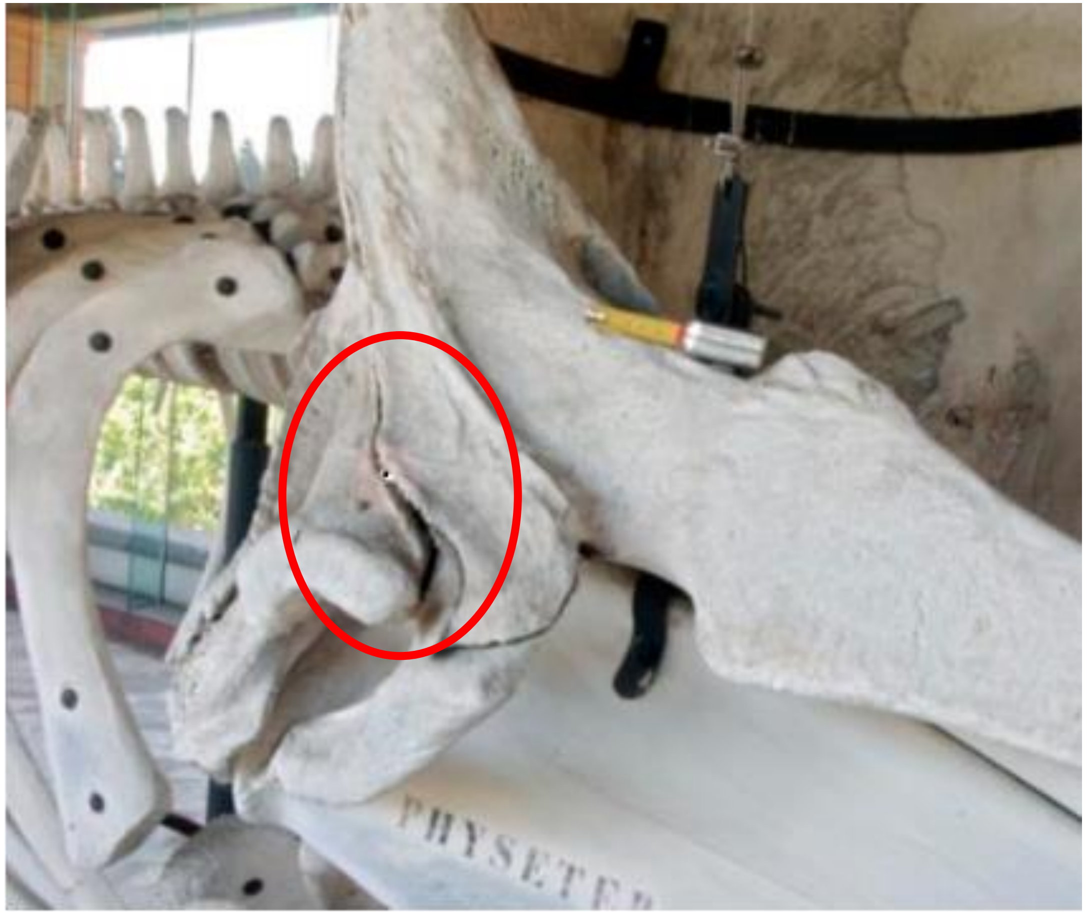

Physeter macrocephalus

Copyright holder: Felix G. Marx/Museo di Storia Naturale e del Territorio, Università di Pisa, Italy

[64] 'Shape of ascending process of maxilla'

(0)'triangular and tapering posteriorly'

(1)'approximately parallel-sided'

(2)'posteriorly divergent and squared'

(0)

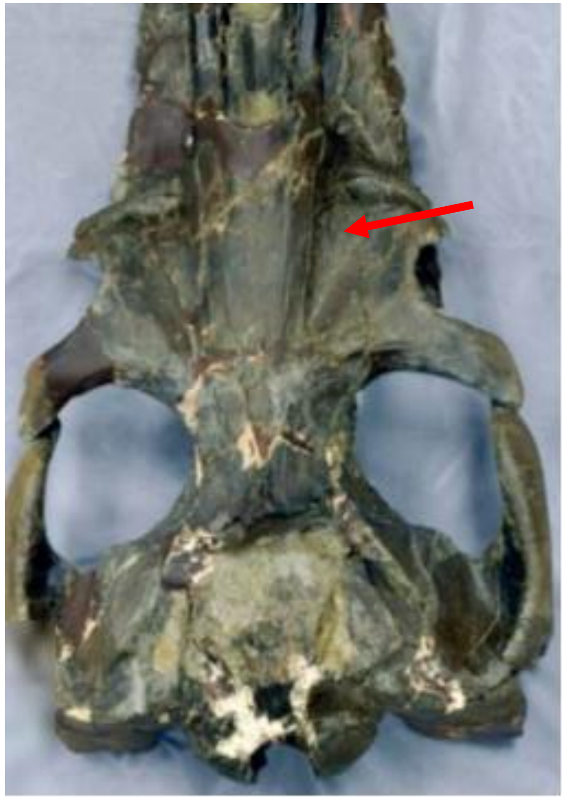

Aetiocetus weltoni

Copyright holder: Felix G. Marx/ University of California Museum of Paleontology, Berkeley, USA

(1)

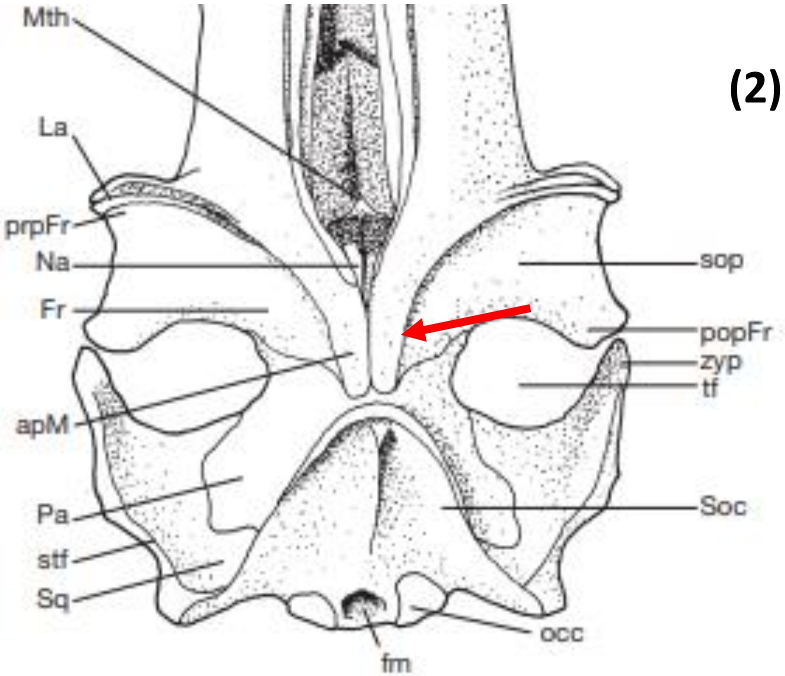

Piscobalaena nana

Adapted from: “The anatomy and relationships of *Piscobalaena nana* (Cetacea, Mysticeti), a Cetotheriidae s.s. from the early Pliocene of Peru”, Bouetel and Muizon, 2006. *Geodiversitas* 28.2 (2006): 319-395.

(2)

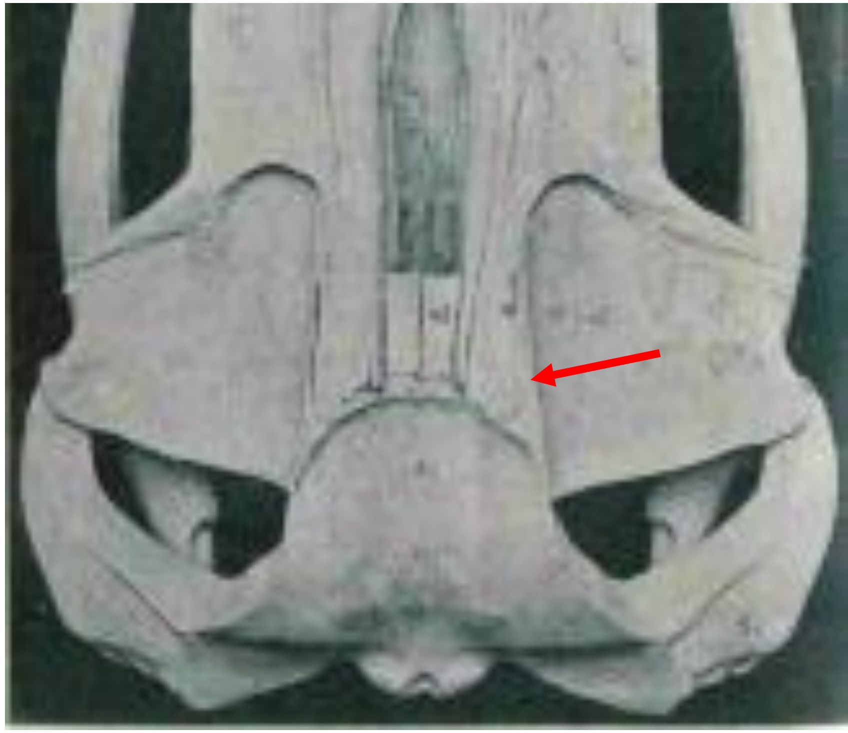

Balaenoptera musculus

Adapted from: “Catalogue of marine mammals of the Mammalogical collection of the Museo de La Plata, Argentina”, Olivares et al, 2016. (original plate II of Lahille (1898))

[65] 'Lateral flange at base of ascending process of maxilla'

(0) 'absent'

(1) 'present'

(0)

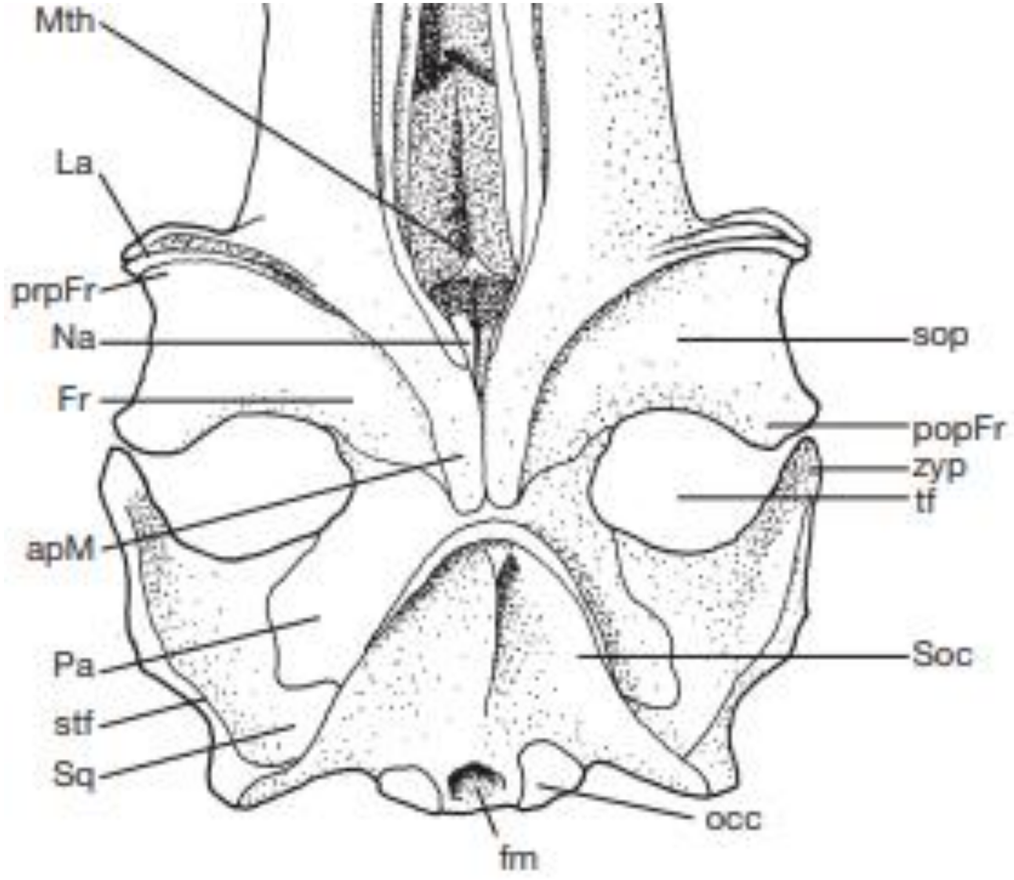

Piscobalaena nana

Adapted from: "The anatomy and relationships of *Piscobalaena nana* (Cetacea, Mysticeti), a Cetotheriidae s.s. from the early Pliocene of Peru", Bouetel and Muizon, 2006. *Geodiversitas* 28.2 (2006): 319-395.

(1)

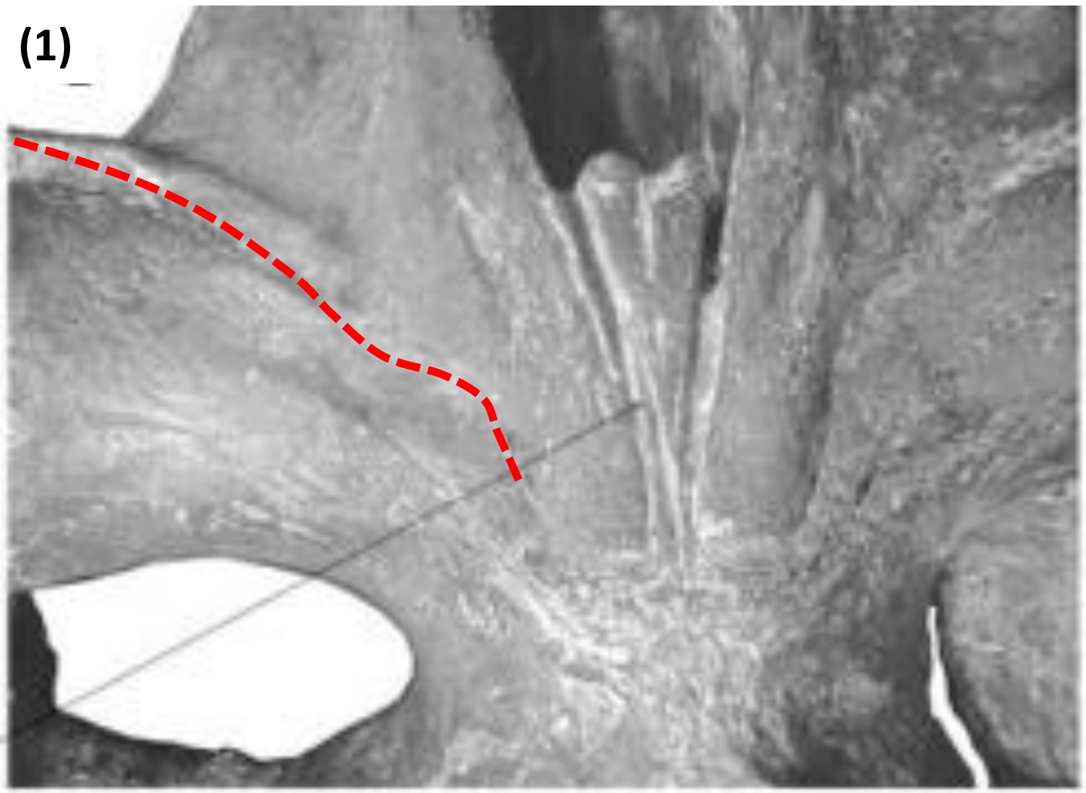

Cetotherium rathkii

Adapted from: "The anatomy of the Late Miocene baleen whale Cetotherium riabinini from Ukraine." Gol'din et al., 2013. *Acta Palaeontologica Polonica* 59.4: 795-814.

[66] 'Triangular wedge of frontal separating ascending process of maxilla from nasal or premaxilla'

(0)'absent'

(1)'present'

(0)

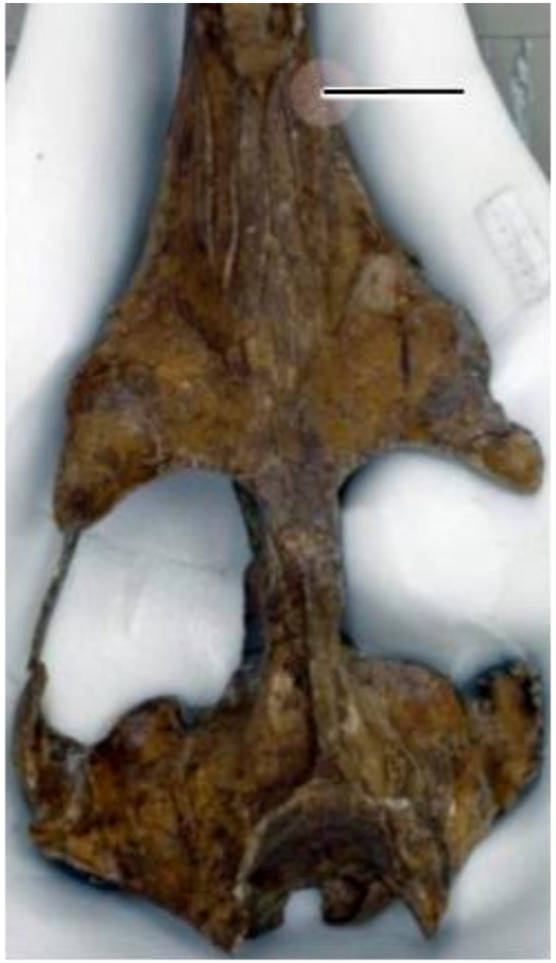

Zygorhiza kochii

Copyright holder: Felix G. Marx/ United States National Museum of Natural History, Washington DC, USA

(1)

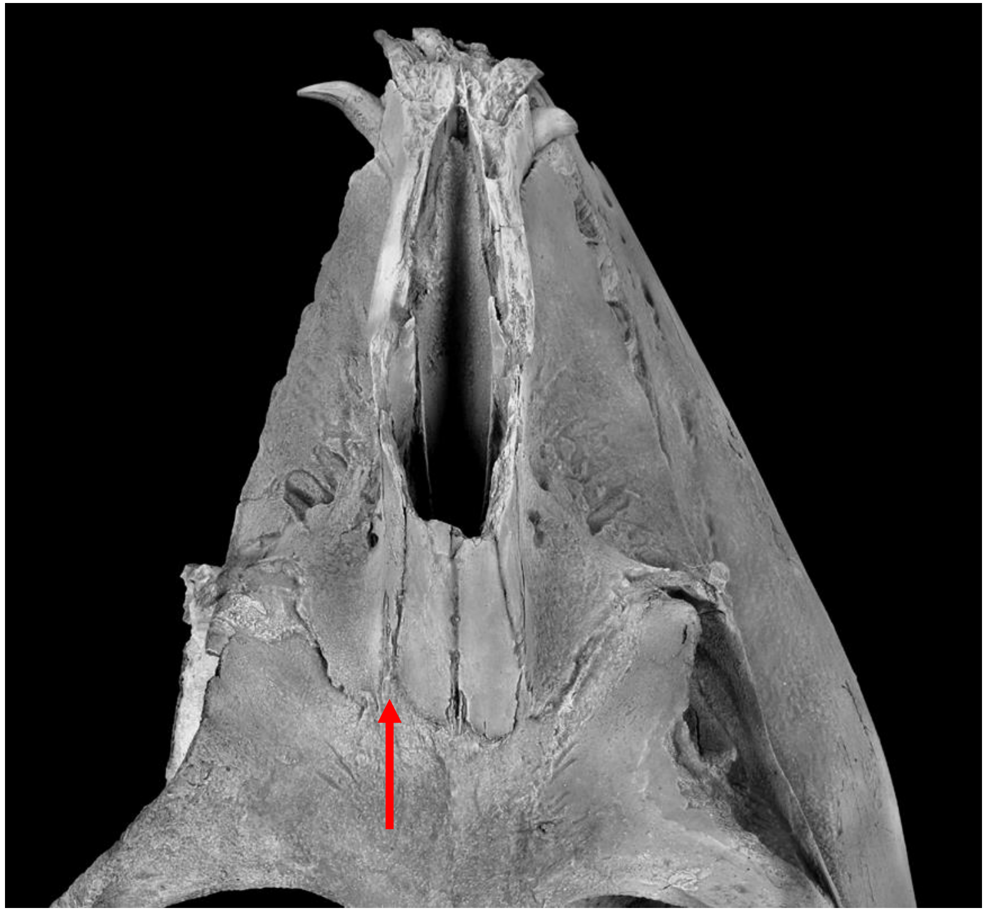

Janjucetus hunderi

Copyright holder: Erich M. G. Fitzgerald/ Museums Victoria, Melbourne, Australia

[67] 'Posterior ends of ascending processes of maxillae in dorsal view'

(0) 'separated by both nasals and premaxillae'

(1) 'separated by nasals only'

(2) 'effectively contact each other medially with nasal being compressed into a thin sheet'

(0)

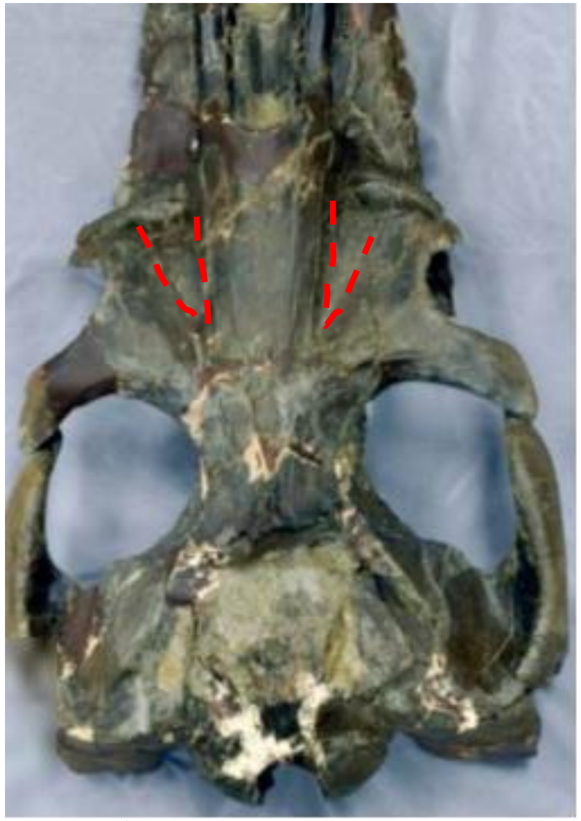

Aetiocetus weltoni

(1)

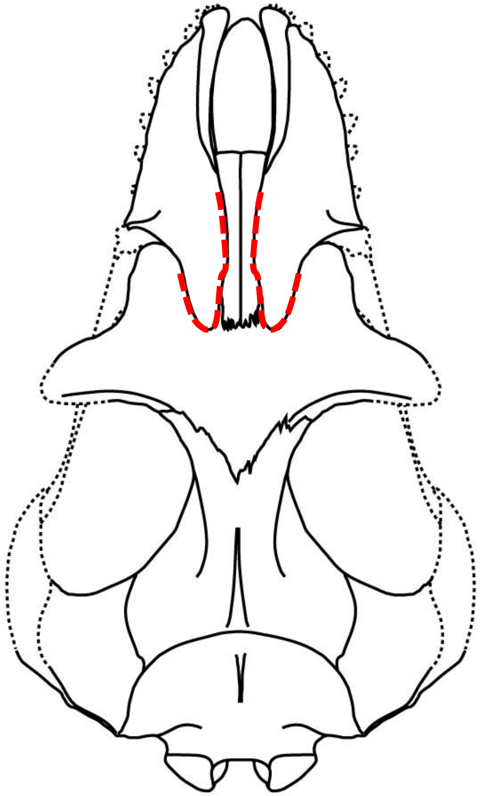

Mammalodon colliveri

(2)

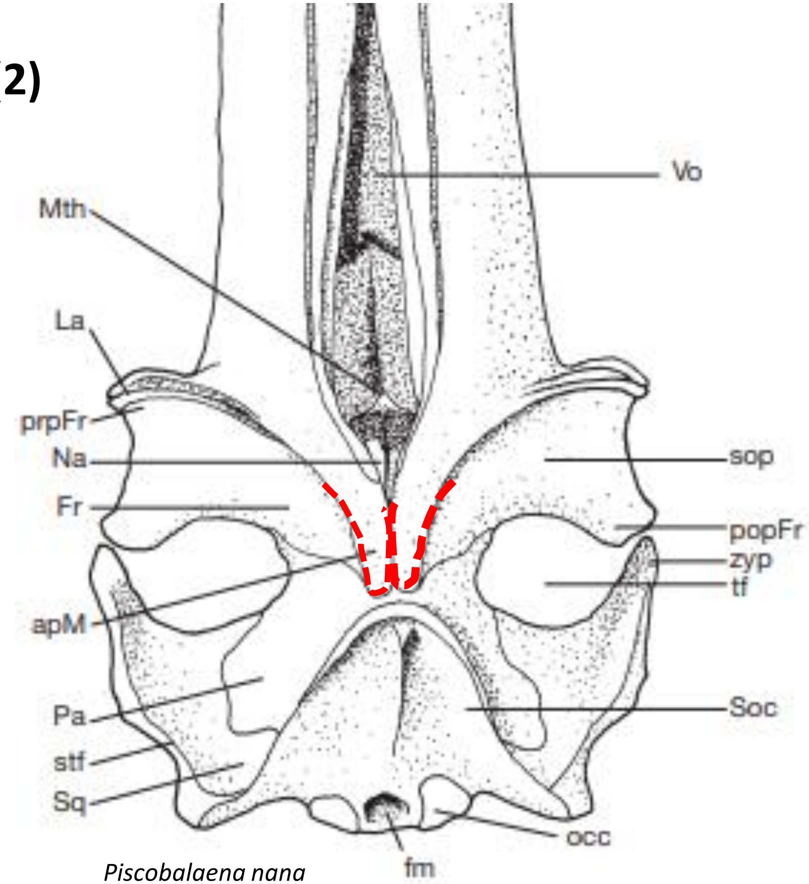

Piscobalaena nana

Copyright holder: Felix G. Marx/ University of California Museum of Paleontology, Berkeley, USA

Copyright holder: Erich M. G. Fitzgerald/ Museums Victoria, Melbourne, Australia

Adapted from: "The anatomy and relationships of *Piscobalaena nana* (Cetacea, Mysticeti), a Cetotheriidae s.s. from the early Pliocene of Peru", Bouetel and Muizon, 2006. *Geodiversitas* 28.2 (2006): 319-395.

[68] 'Relative position of posteriormost edge of ascending process of maxilla in dorsal view'

(0)'approximately in transverse line with or posterior to posterior edge of nasal'

(1)'anterior to posterior edge of nasal'

(0)

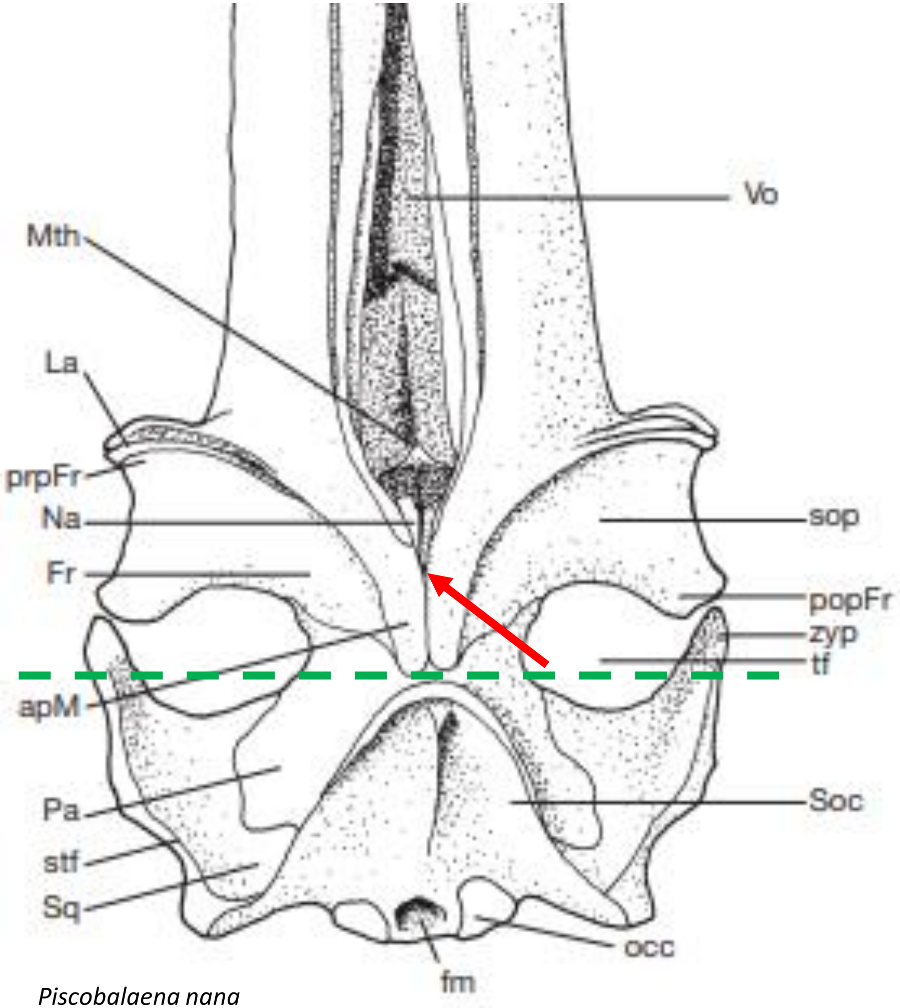

Piscobalaena nana

Adapted from: "The anatomy and relationships of *Piscobalaena nana* (Cetacea, Mysticeti), a Cetotheriidae s.s. from the early Pliocene of Peru", Bouetel and Muizon, 2006. *Geodiversitas* 28.2 (2006): 319-395.

(1)

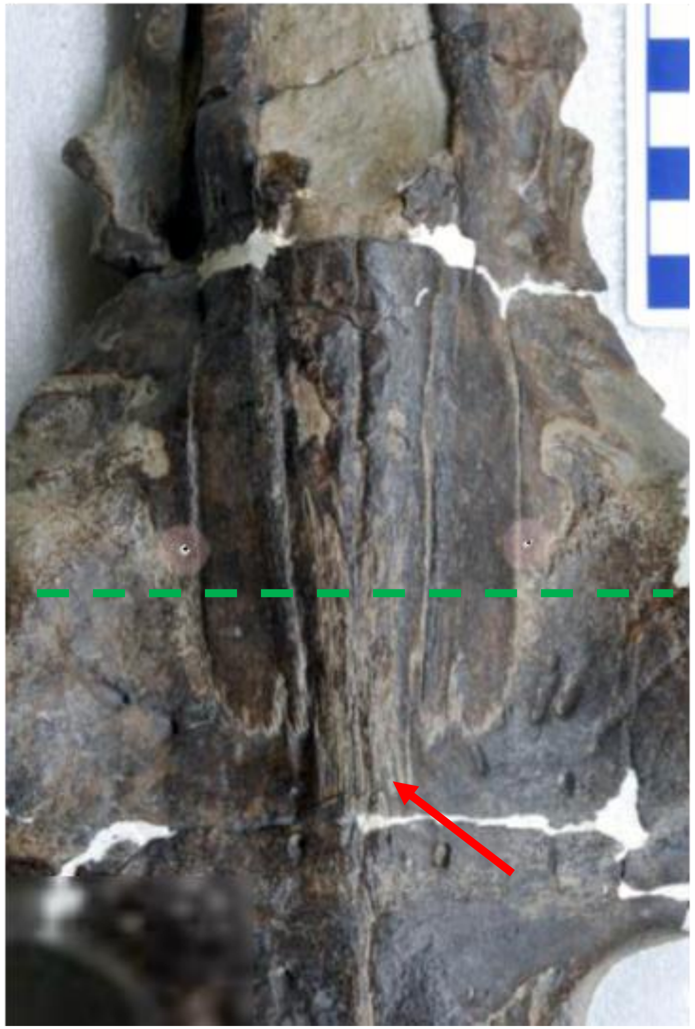

Fucaia goedertorum

Copyright holder: Felix G. Marx/Natural History Museum of Los Angeles County, Los Angeles, USA

[69] 'Length of nasal relative to bizygomatic width'

(0)'less than 50% of bizygomatic width’

(1)'more than 50% of bizygomatic width'

(0)

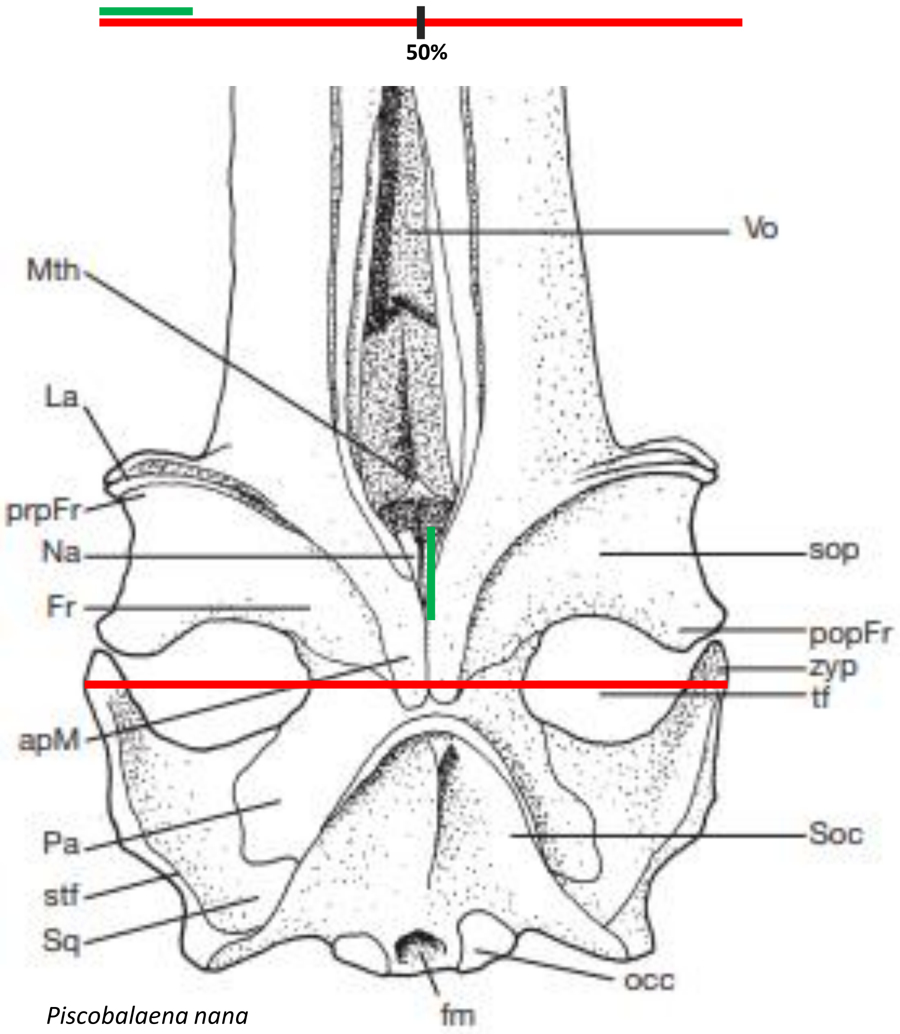

Piscobalaena nana

Adapted from: “The anatomy and relationships of *Piscobalaena nana* (Cetacea, Mysticeti), a Cetotheriidae s.s. from the early Pliocene of Peru”, Bouetel and Muizon, 2006. *Geodiversitas* 28.2 (2006): 319-395.

(1)

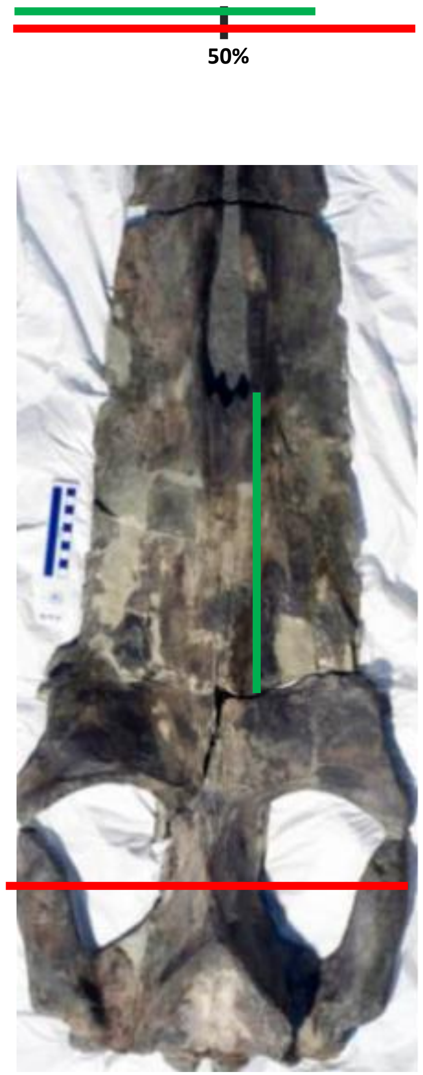

Yamatocetus canaliculatus

Copyright holder: Felix G. Marx/ Kitakyushu Museum of Natural and Human History, Kitakyushu, Kyushu, Japan

[70] 'Shape of nasal'

(0) 'medial and lateral margins parallel'

(1) 'medial and lateral margins posteriorly convergent'

(0)

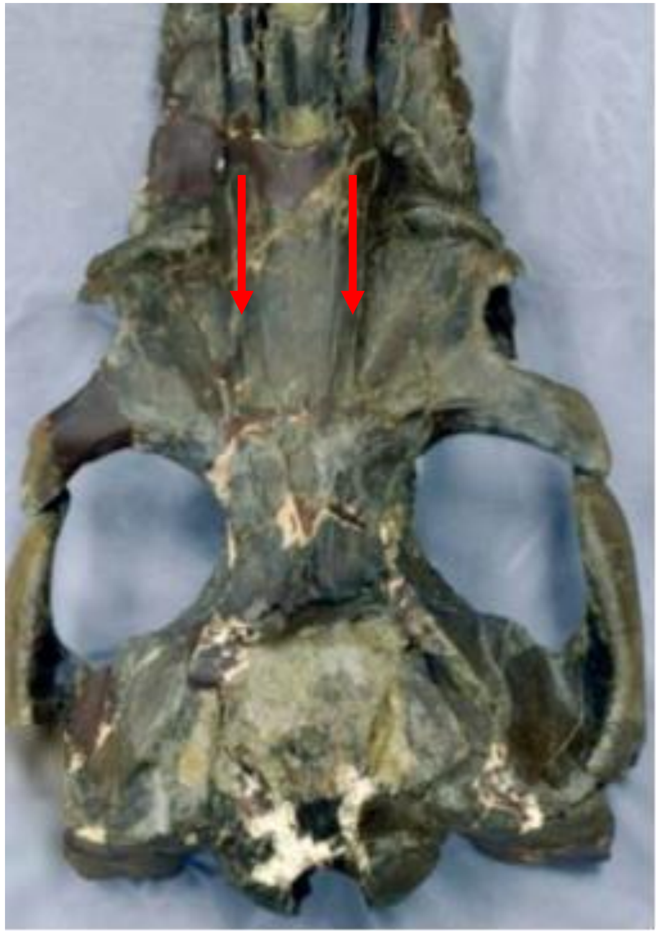

Aetiocetus weltoni

Copyright holder: Felix G. Marx/ University of California Museum of Paleontology, Berkeley, USA

(1)

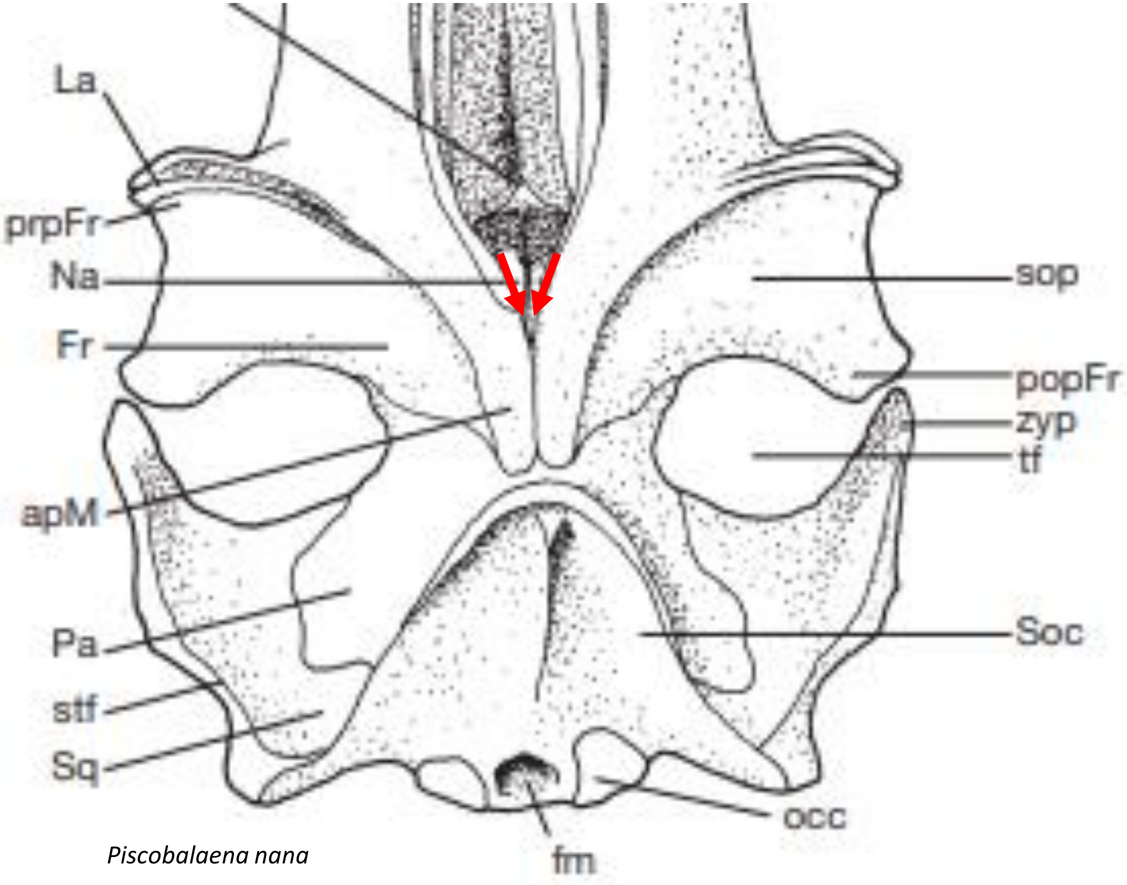

Piscobalaena nana

Adapted from: "The anatomy and relationships of *Piscobalaena nana* (Cetacea, Mysticeti), a Cetotheriidae s.s. from the early Pliocene of Peru", Bouetel and Muizon, 2006. *Geodiversitas* 28.2 (2006): 319-395.

[71] 'Anterior margins of nasals'

(0) 'roughly straight or U shaped'

(1) 'form a distinct, posteriorly pointing W shape'

(2) 'with point on midline and a gap on each side between premaxilla and nasal'

(3) 'form an anteriorly pointing W shape'

(0)

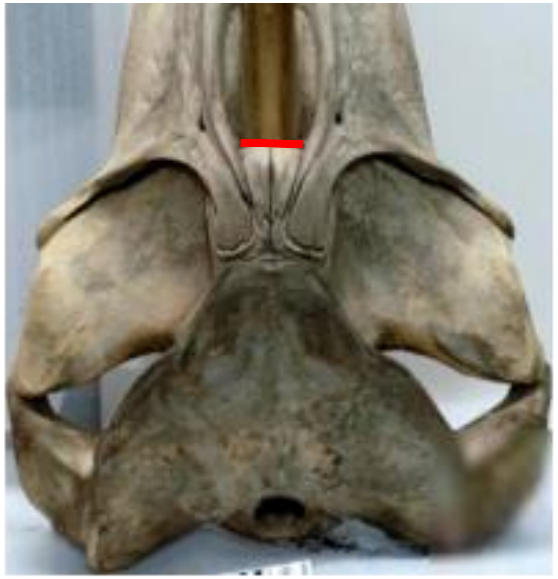

Balaenoptera acutorostrata

Copyright holder: Felix G. Marx/ The Charleston Museum, Charleston, South Carolina, USA

(1)

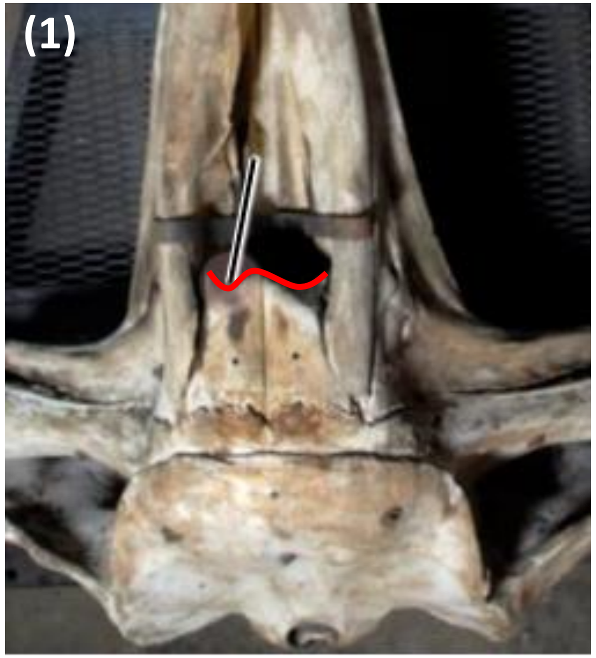

Eubalaena spp. (USNM:267612)

Adapted from: Copyright holder: Felix G. Marx/ United States National Museum of Natural History, Washington DC, USA

(2)

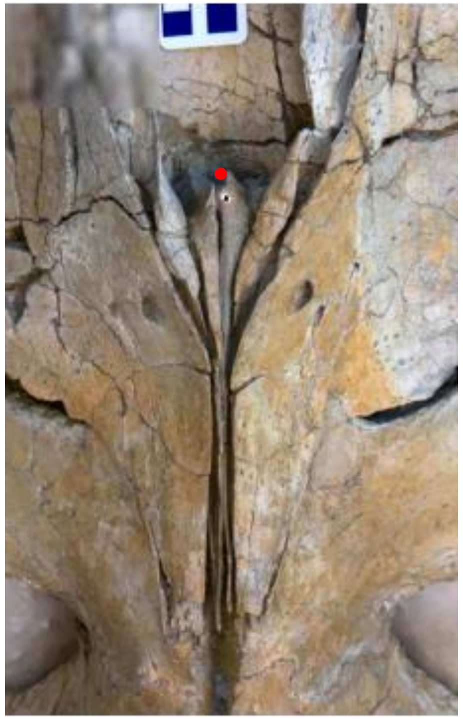

Herpetocetus morrowi

Copyright holder: Felix G. Marx/ University of California Museum of Paleontology, Berkeley, USA

(3)

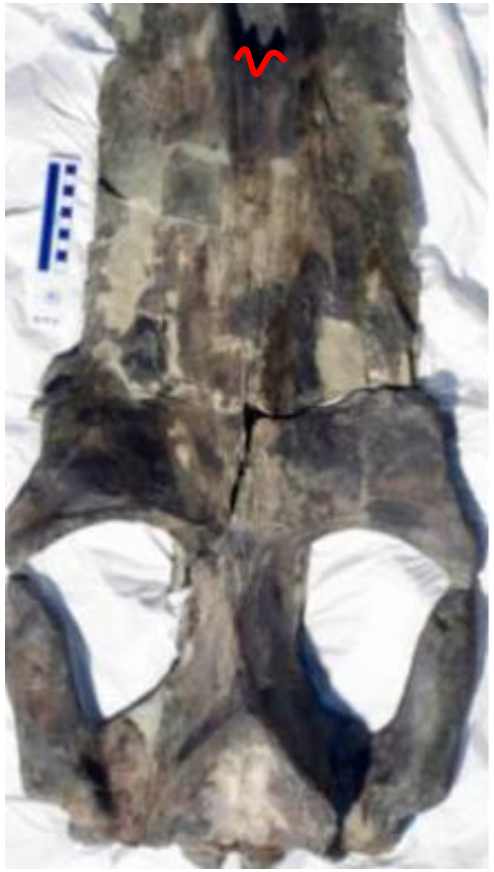

Yamatocetus canaliculatus

Copyright holder: Felix G. Marx/ Kitakyushu Museum of Natural and Human History, Kitakyushu, Kyushu, Japan

[72] 'Dorsal surface of nasals'

(0) 'flattened’

(1) 'developed into a sagittal keel’

(2) 'medial portion raised into a nasal scoop'

(0)

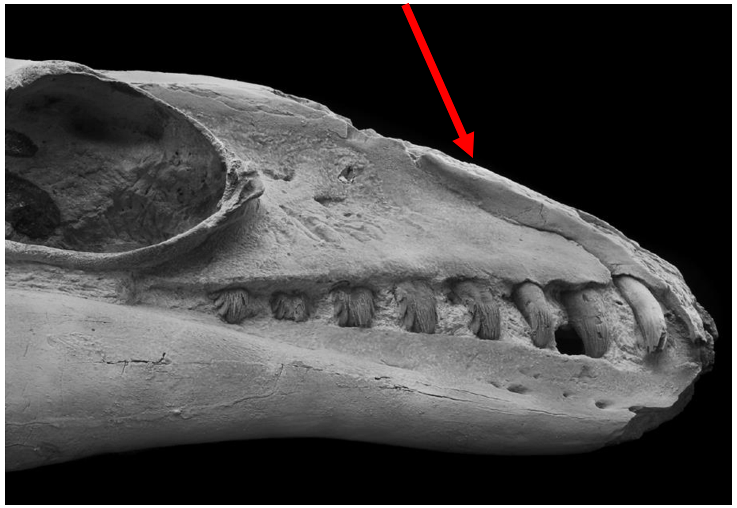

Janjucetus hunderi

Copyright holder: Erich M. G. Fitzgerald/ Museums Victoria, Melbourne, Australia

(1)

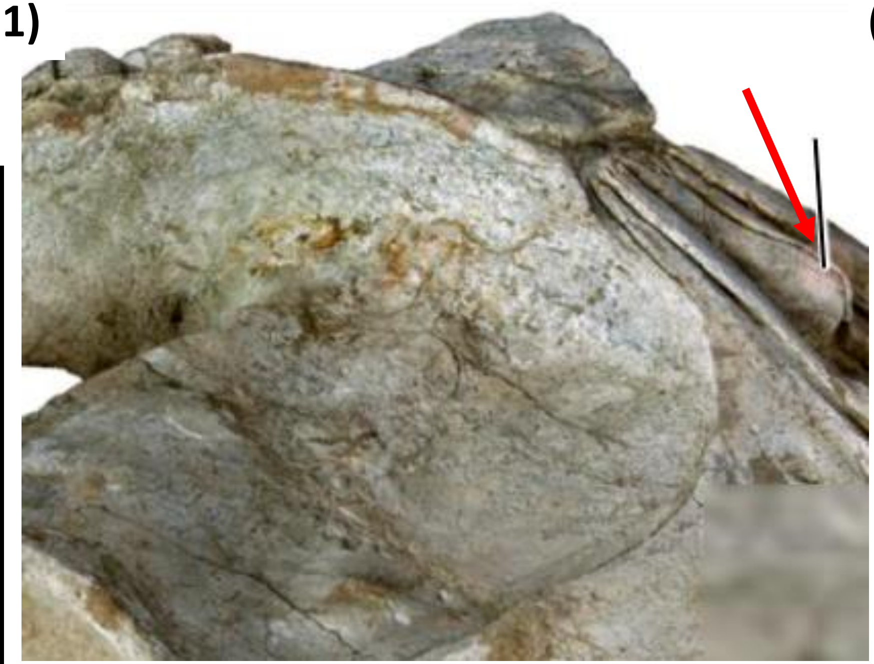

Incakujira anillodefuego

Adapted from: Copyright holder: Felix G. Marx/ Gamagori Museum of Earth, Life and the Sea, Gamagori, Japan

(2)

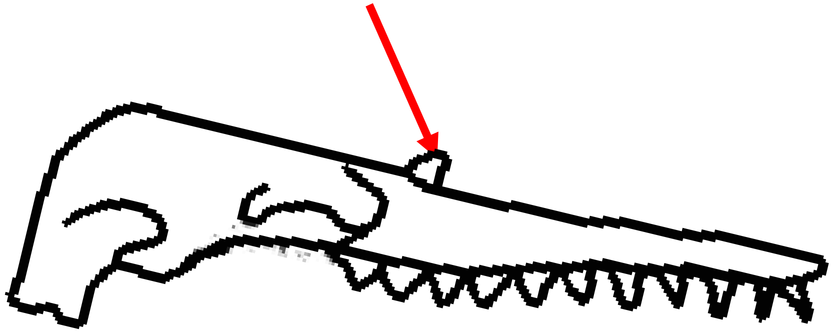

Example based on *Coronodon havensteini*

[73] 'Separation of posterior portions of nasals along sagittal plane by narial process of frontal'

(0)'present'

(1)'absent'

(0)

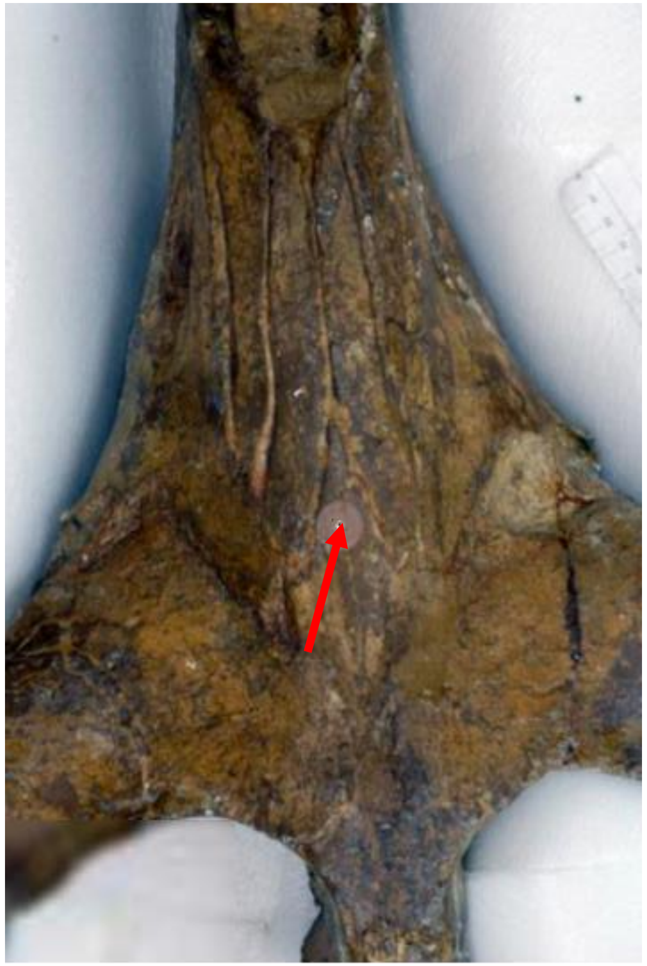

Zygorhiza kochii

Copyright holder: Felix G. Marx/ United States National Museum of Natural History, Washington DC, USA

(1)

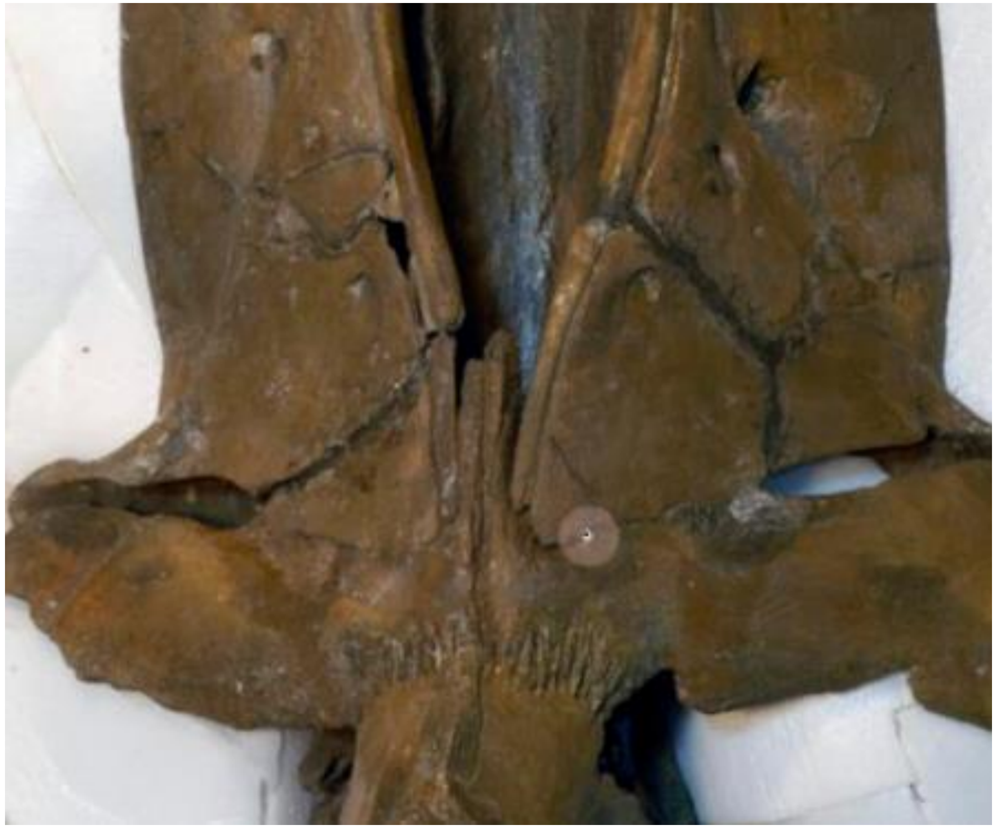

Diorocetus hiatus

Copyright holder: Felix G. Marx/ United States National Museum of Natural History, Washington DC, USA

[74] 'Zygomatic process of squamosal and exoccipital in dorsal view'

(0) 'clearly separated by an angle’

(1) 'posterior border of zygomatic process and lateral edge of exoccipital are confluent forming a continuous or nearly continuous lateral skull border'

(0)

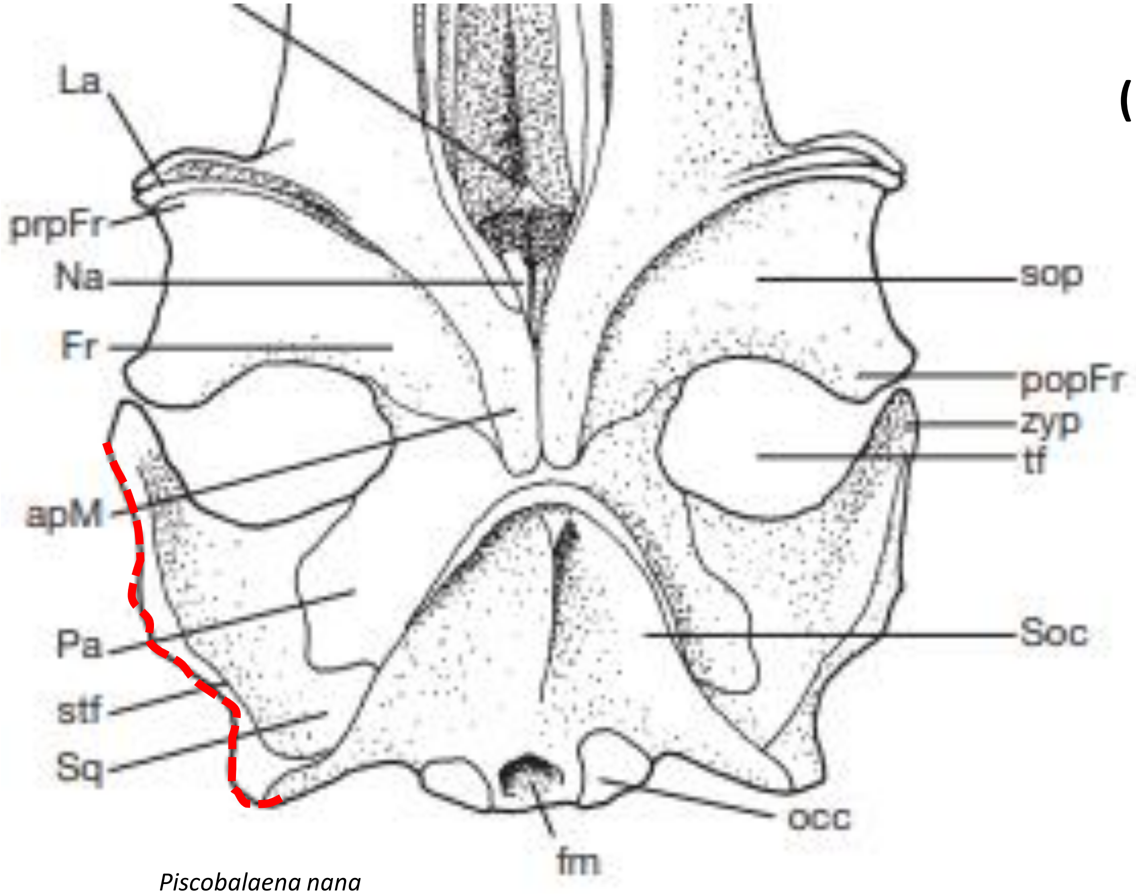

Piscobalaena nana

(1)

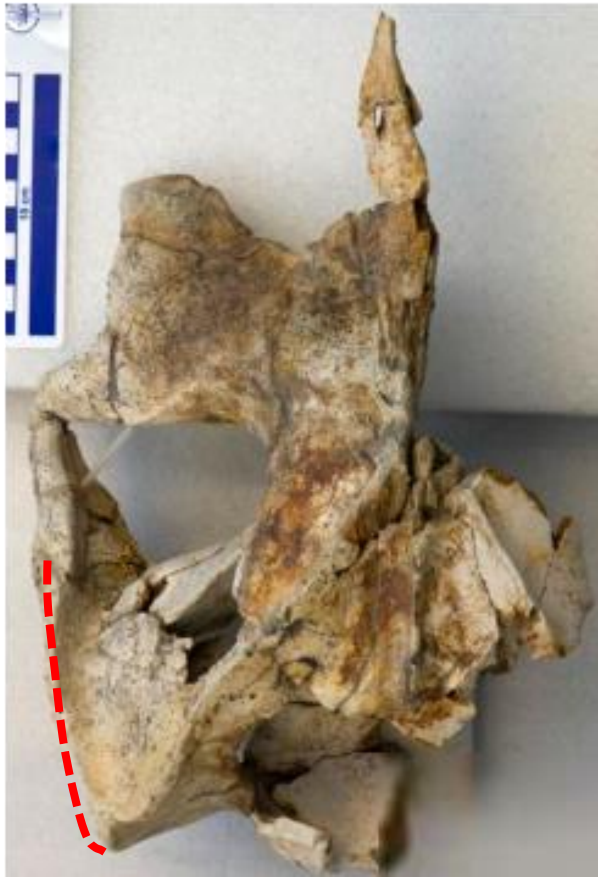

Herpetocetus morrowi

Adapted from: “The anatomy and relationships of *Piscobalaena nana* (Cetacea, Mysticeti), a Cetotheriidae s.s. from the early Pliocene of Peru”, Bouetel and Muizon, 2006. *Geodiversitas* 28.2 (2006): 319-395.

Copyright holder: Felix G. Marx/ San Diego Museum of Natural History, San Diego, USA

[75] 'Orbitotemporal crest'

(0)'positioned along posterior border of supraorbital process with the origin of the temporal muscle facing posteriorly or posteroventrally'

(1)'absent or positioned on the dorsal surface of the supraorbital process with the origin of the temporal muscle facing posterodorsally or dorsally'

(0)

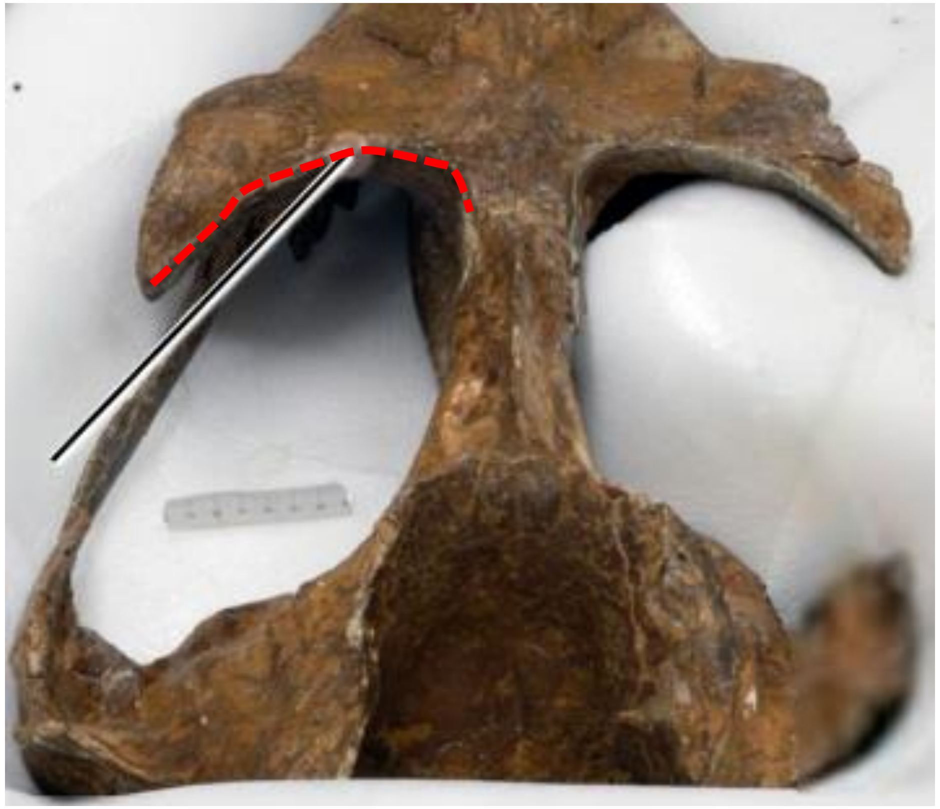

Zygorhiza kochii

Copyright holder: Felix G. Marx/ United States National Museum of Natural History, Washington DC, USA

(1)

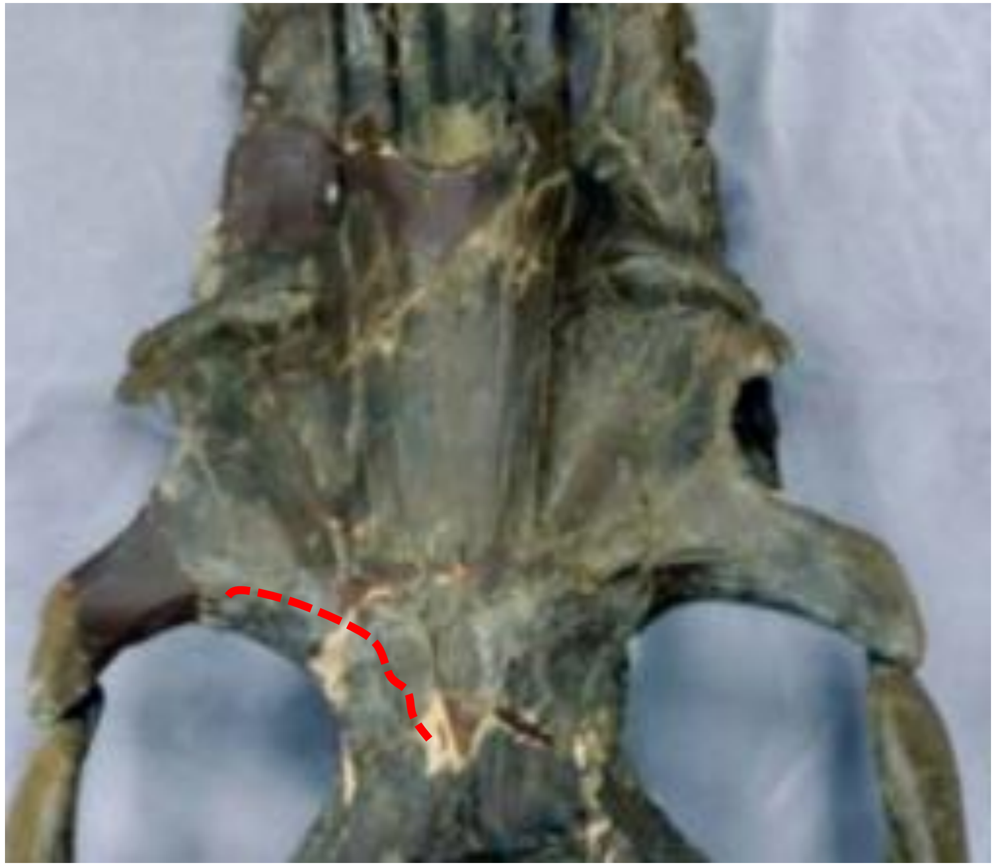

Aetiocetus weltoni

Copyright holder: Felix G. Marx/ University of California Museum of Paleontology, Berkeley, USA

[76] 'Area enclosed by orbitotemporal crest on supraorbital process of frontal'

(0) 'forms less than half of the dorsal surface of the supraorbital process'

(1) 'covers half or more of the dorsal surface of the supraorbital process'

(0)

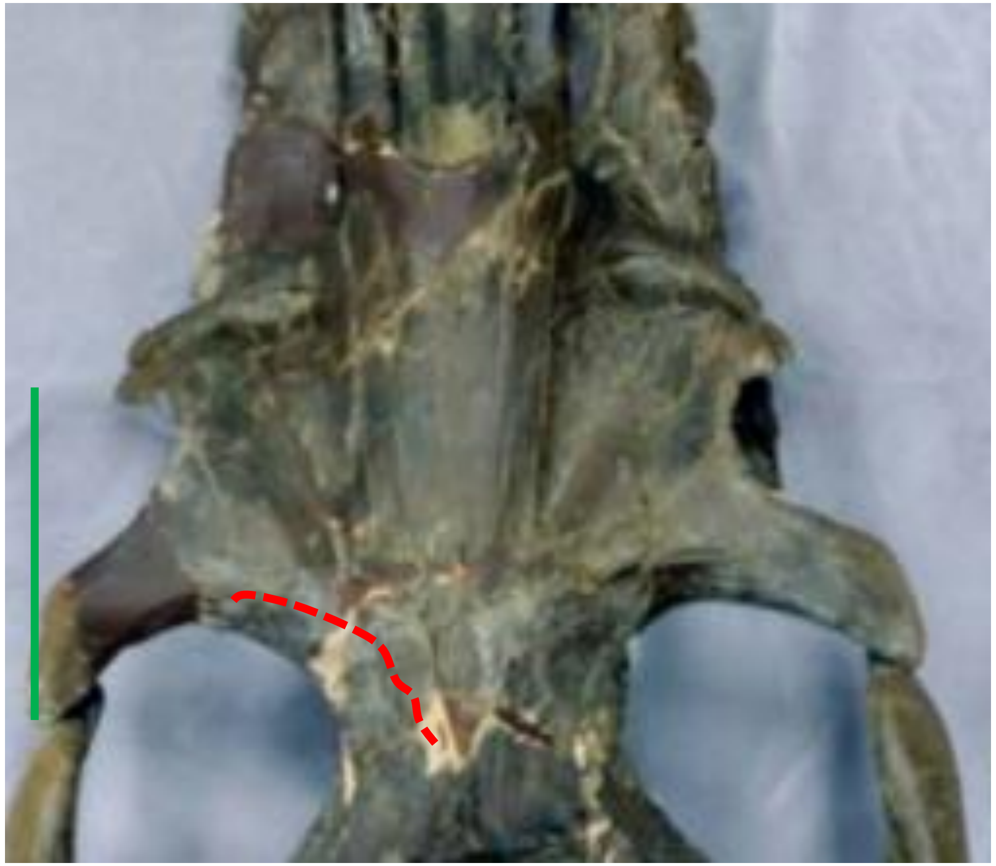

Aetiocetus weltoni

Copyright holder: Felix G. Marx/ University of California Museum of Paleontology, Berkeley, USA

(1)

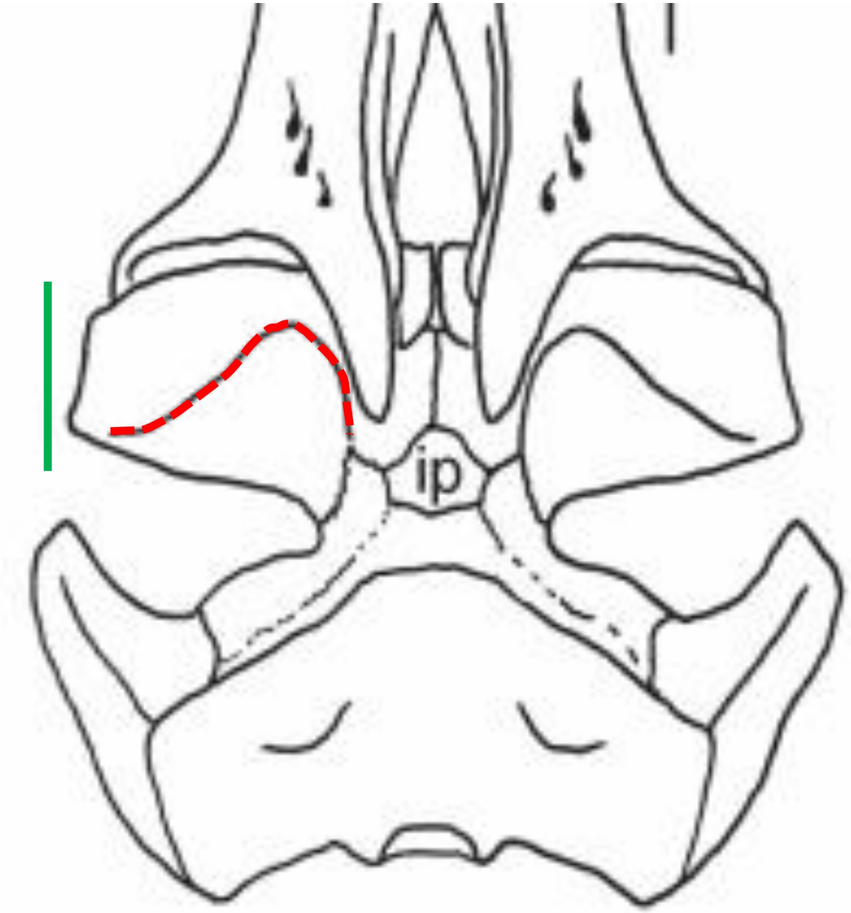

Eschrichtioides gastaldii

Adapted from: "Taxonomy and evolution of the Italian Pliocene Mysticeti (Mammalia, Cetacea): a state of the art." Bisconti, 2009. *Bollettino della Società Paleontologica Italiana* 48.2: 147-156.

[77] 'Outline and orientation of orbitotemporal crest'

(0) 'subparallel to posterior border of supraorbital process'

(1) 'distal half oriented distinctly posterolaterally and approaching the posterolateral corner of the supraorbital process'

(2) 'as state 1 but with the crest terminating halfway along the posterior border of the supraorbital process'

(3) 'as state 1 but with the crest being distinctly U-shaped'

(0)

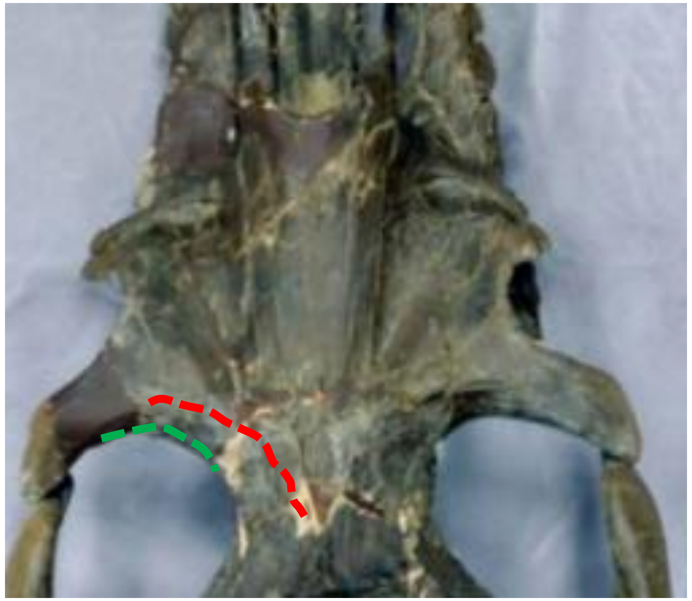

Aetiocetus weltoni

Copyright holder: Felix G. Marx/ University of California Museum of Paleontology, Berkeley, USA

(1)

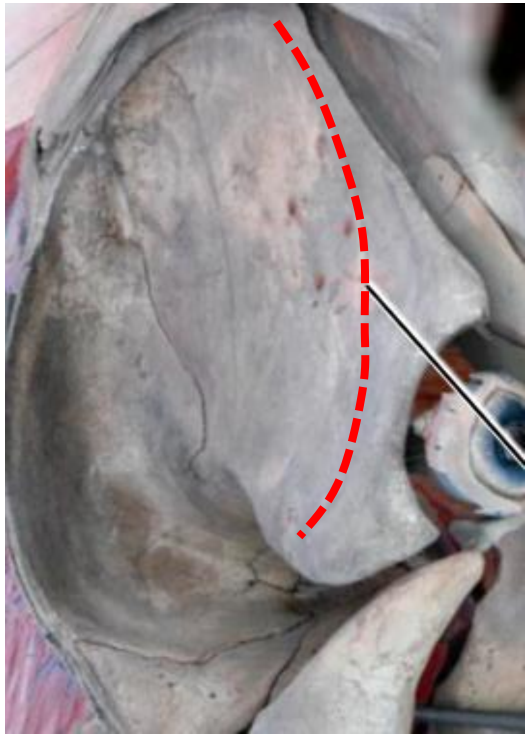

Balaenoptera borealis

Copyright holder: Felix G. Marx/ Staatliches Museum für Naturkunde Stuttgart, Germany

(2)

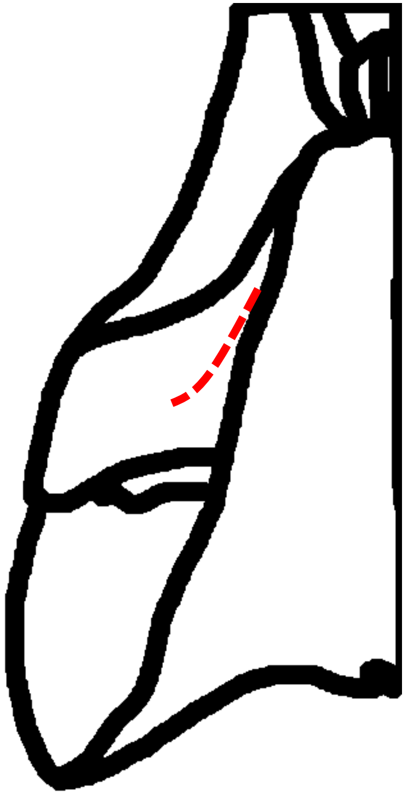

Example based on *Caperea marginata*

(3)

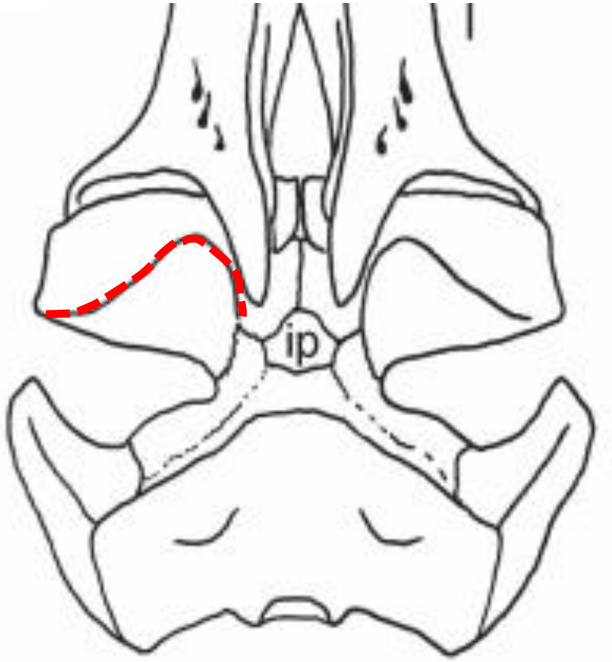

Eschrichtioides gastaldii

Adapted from: "Taxonomy and evolution of the Italian Pliocene Mysticeti (Mammalia, Cetacea): a state of the art." Bisconti, 2009. *Bollettino della Società Paleontologica Italiana* 48.2: 147-156.

[78] 'Shape of temporal fossa'

(0)'longer anteroposteriorly than wide transversely or as wide as long'

(1)'wider than long'

(0)

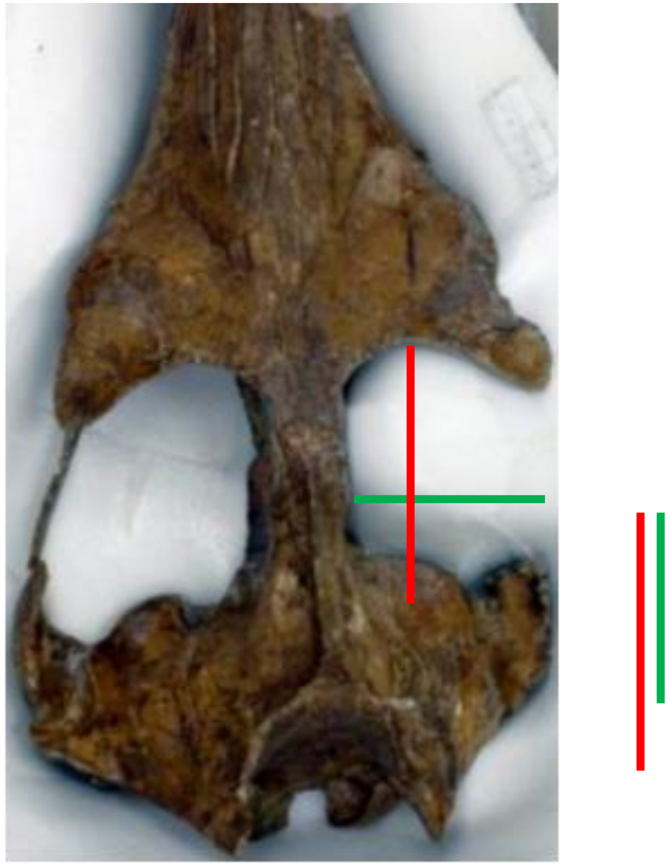

Zygorhiza kochii

Copyright holder: Felix G. Marx/ United States National Museum of Natural History, Washington DC, USA

(1)

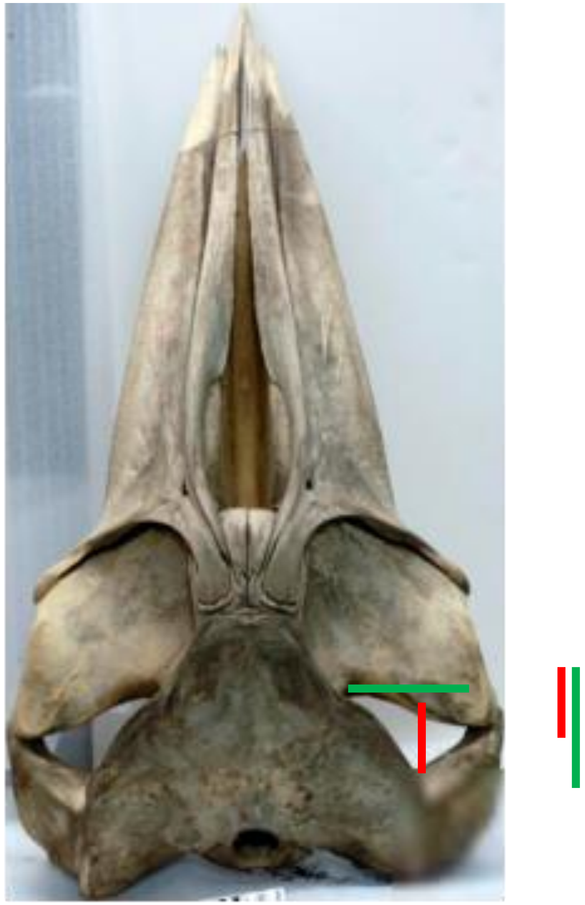

Balaenoptera acutorostrata

Copyright holder: Felix G. Marx/ The Charleston Museum, Charleston, South Carolina, USA

[79] 'Intertemporal constriction'

(0)'longer anteroposteriorly than wide transversely'

(1)'wider than long'

(0)

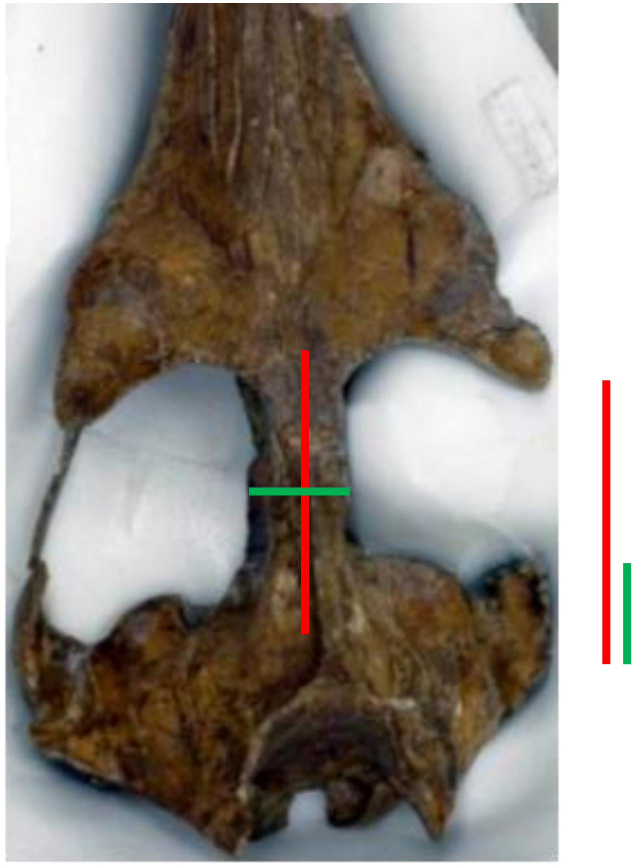

Zygorhiza kochii

Copyright holder: Felix G. Marx/ United States National Museum of Natural History, Washington DC, USA

(1)

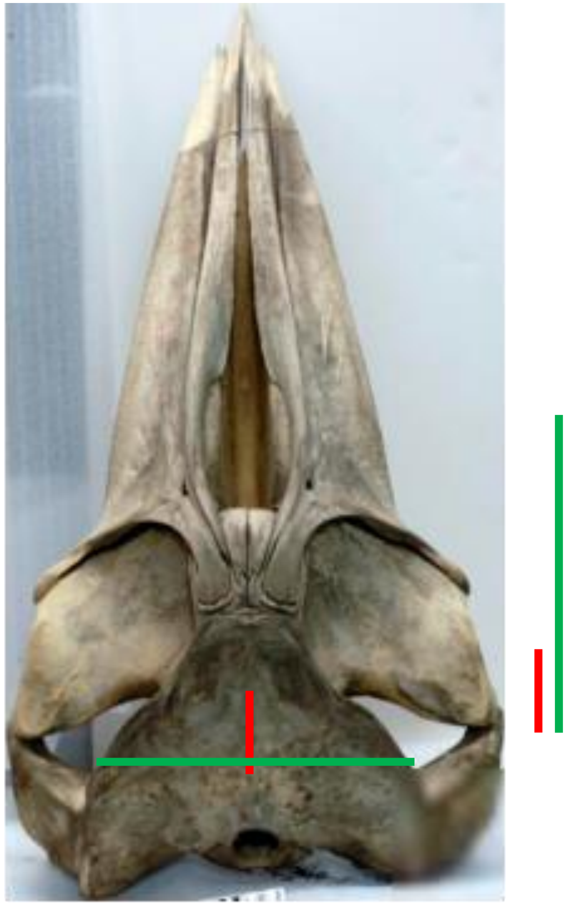

Balaenoptera acutorostrata

Copyright holder: Felix G. Marx/ The Charleston Museum, Charleston, South Carolina, USA

[80] 'Exposure of frontal on skull vertex'

(0) 'broadly exposed'

(1) 'anteroposteriorly compressed or absent'

(0)

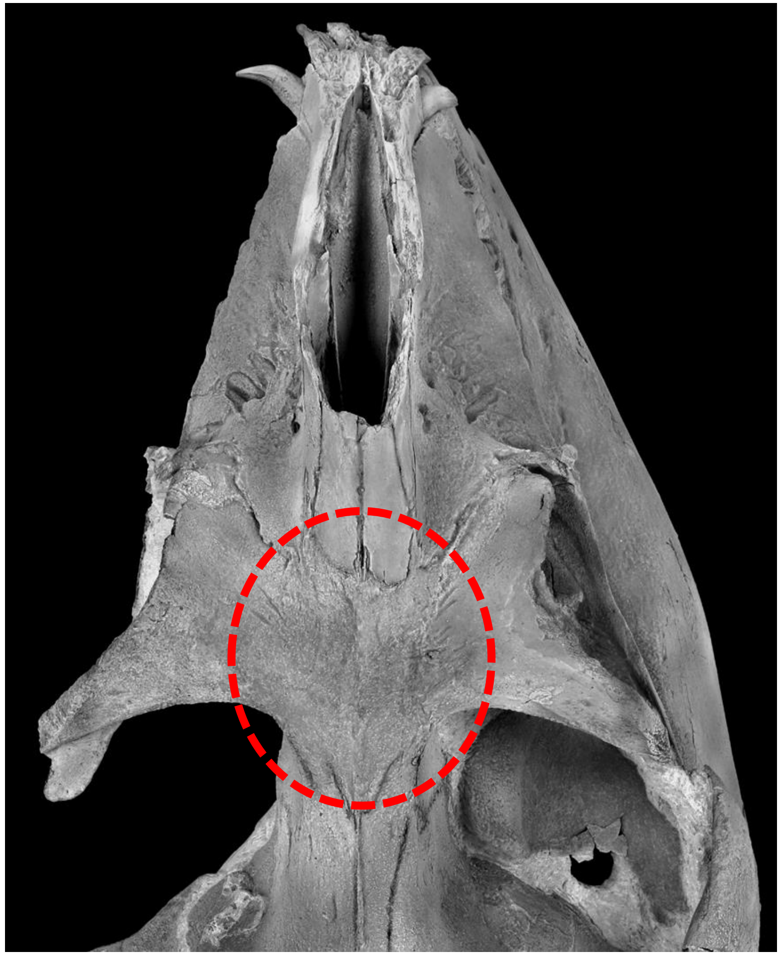

Janjucetus hunderi

Copyright holder: Erich M. G. Fitzgerald/ Museums Victoria, Melbourne, Australia

(1)

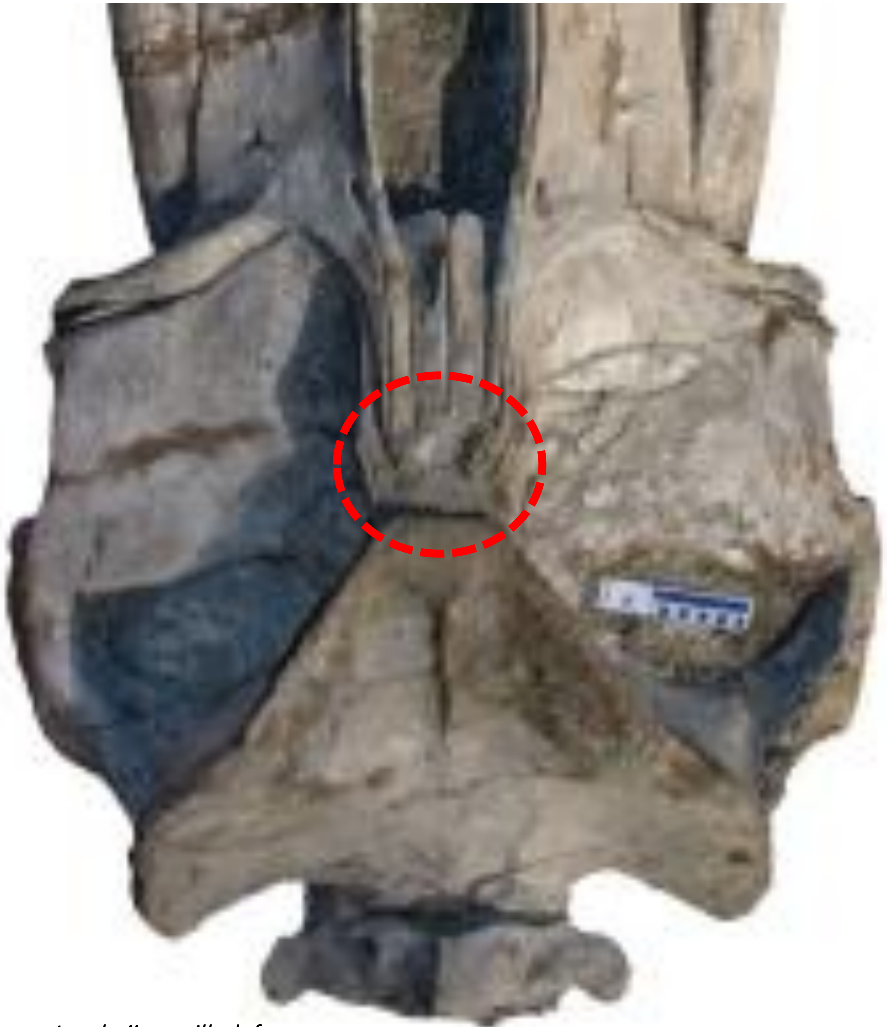

Incakujira anillodefuego

Adapted from: "A new Miocene baleen whale from the Peruvian desert." Marx and Kohno, 2016. *Royal Society Open Science* 3.10 (2016): 160542.

[81] 'Parietal and interparietal'

(0)'anteriormost point located no further forward than postorbital process'

(1)'anteriormost point in line with supraorbital process'

(0)

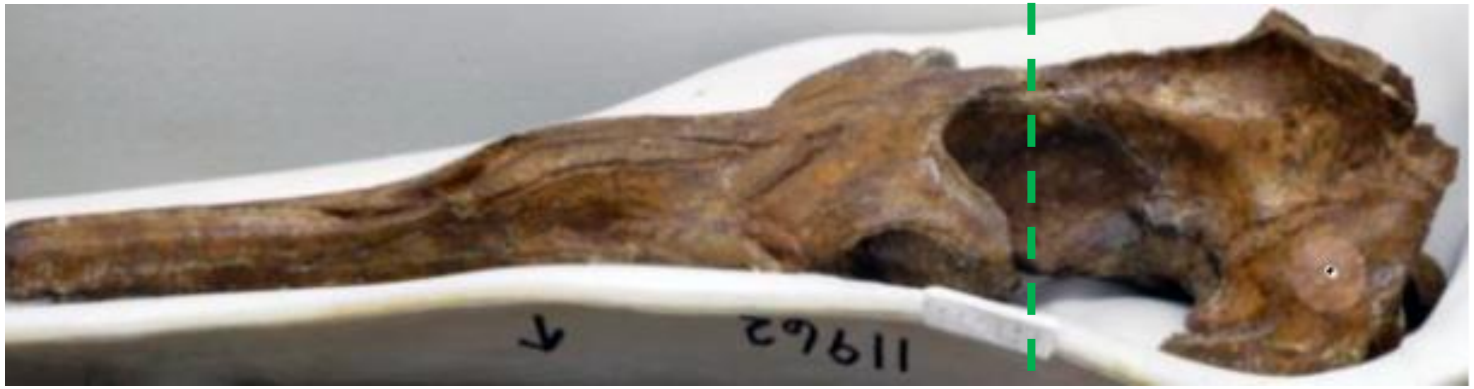

Zygorhiza kochii

Copyright holder: Felix G. Marx/ United States National Museum of Natural History, Washington DC, USA

(1)

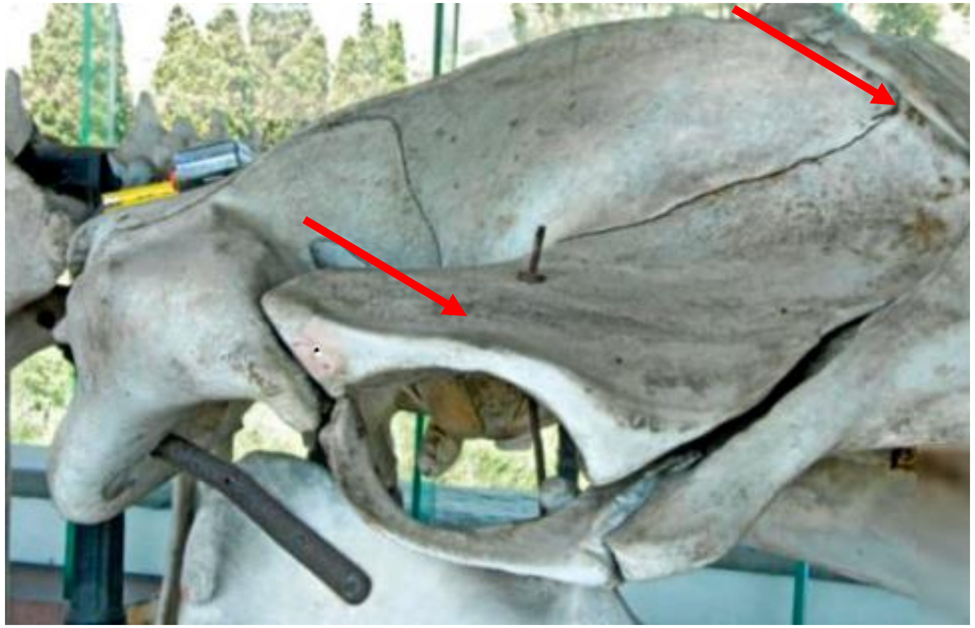

Balaenoptera acutorostrata

Copyright holder: Felix G. Marx/ Museo di Storia Naturale e del Territorio, Università di Pisa, Calci, Italy

[82] 'Sagittal trough on anterior portion of parietal on vertex'

(0)'absent’

(1)'present'

(0)

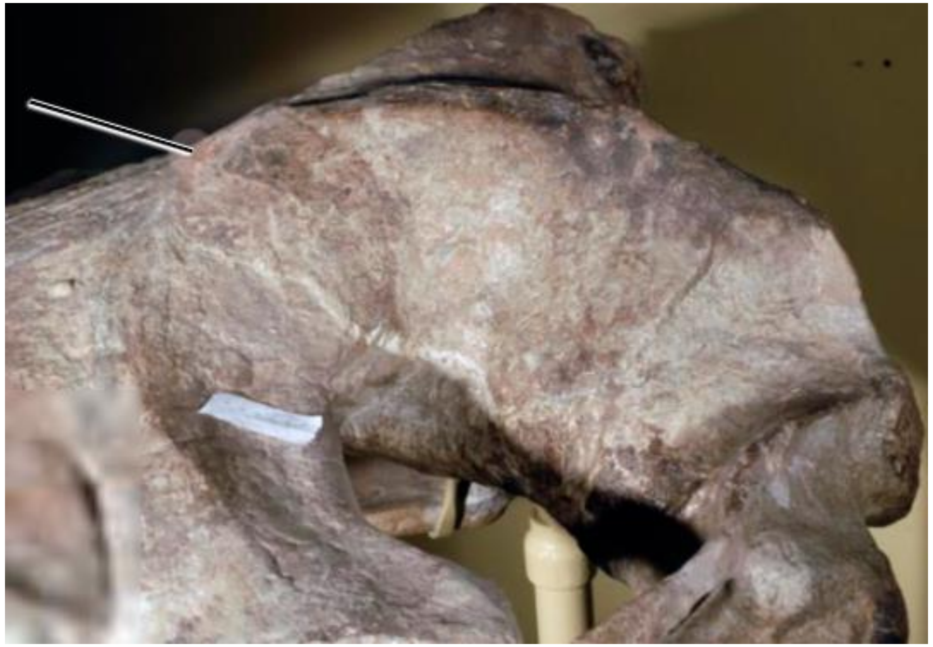

Aglaocetus moreni (mirrored)

Copyright holder: Felix G. Marx/ The Field Museum of Natural History, Chicago, USA

(1)

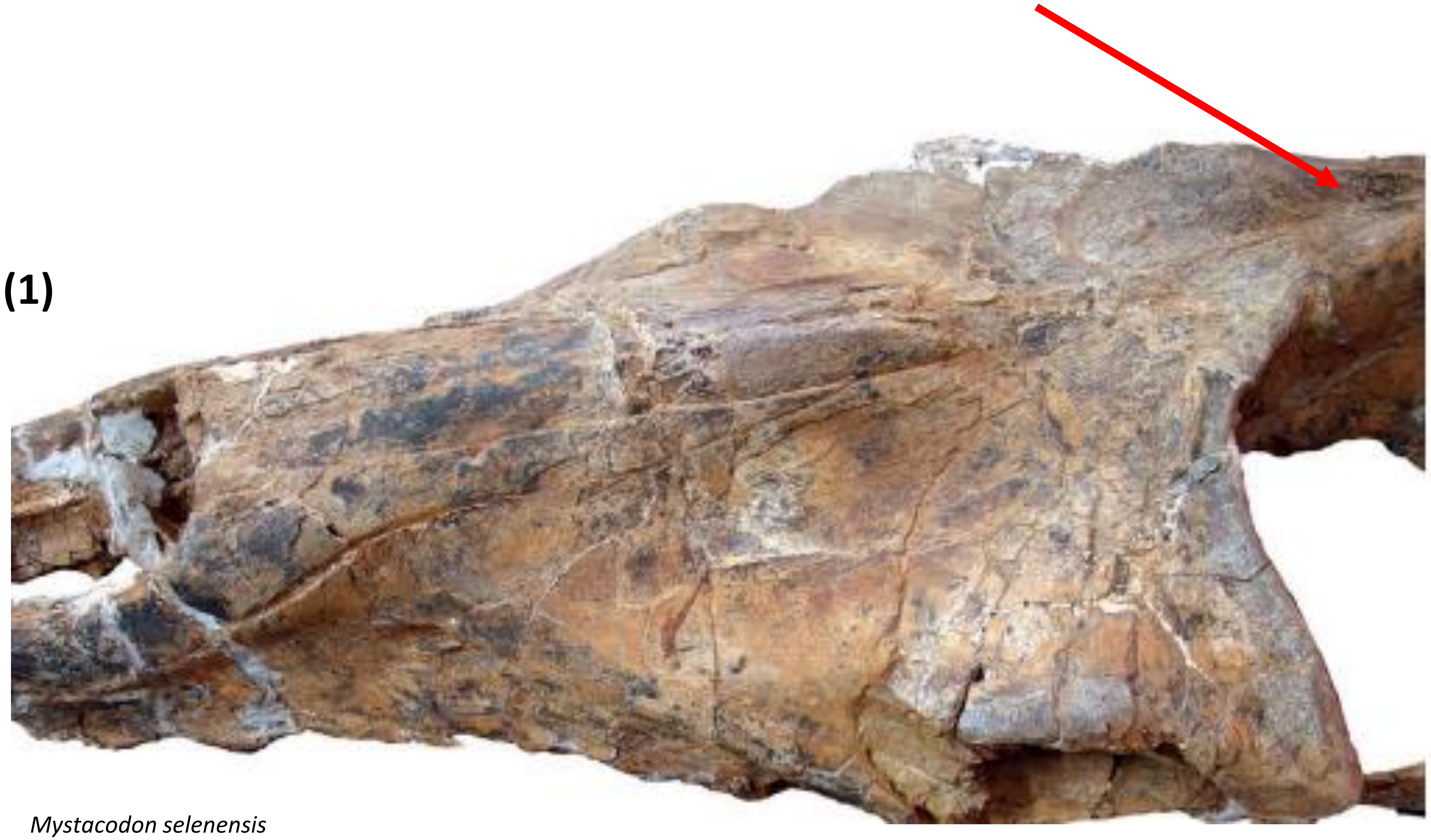

Mystacodon selenensis

Adapted from: *"Mystacodon selenensis, the earliest known toothed mysticete (Cetacea, Mammalia) from the late Eocene of Peru: anatomy, phylogeny, and feeding adaptations." De Muizon et al., 2019. Geodiversitas 41.1: 401-499.*

[83] 'Fronto-parietal suture developed as a sharp crest'

(0)'absent'

(1)'present'

(0)

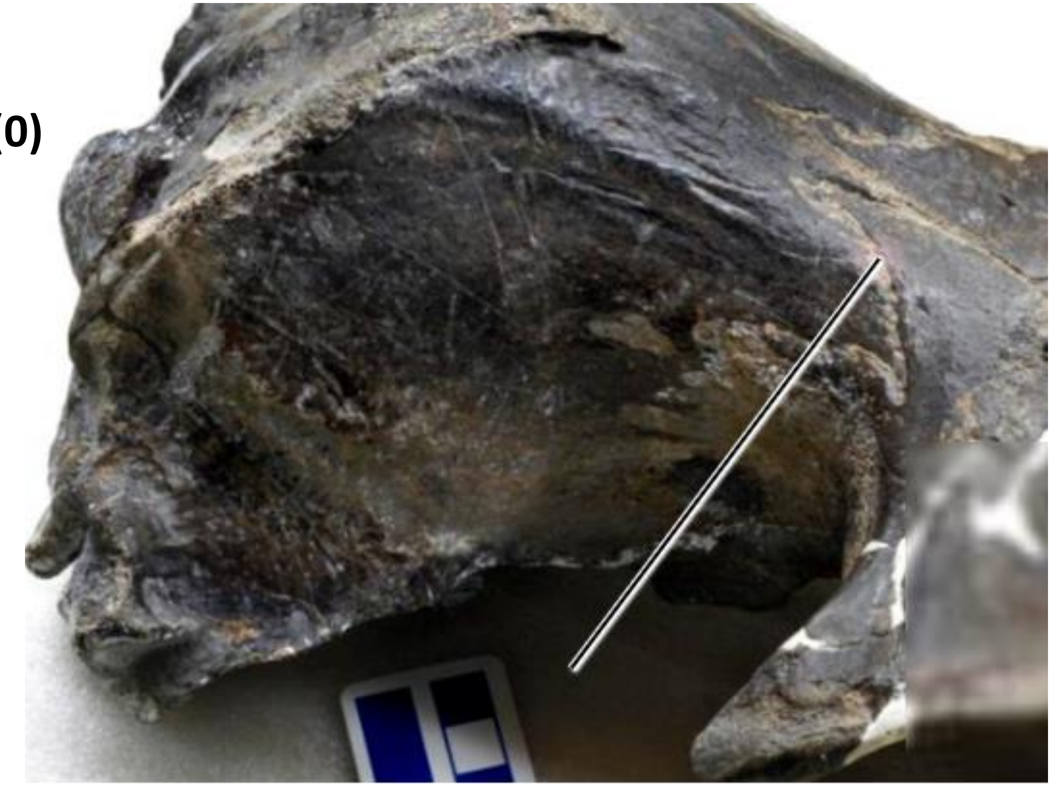

Fucaia goedertorum

Copyright holder: Felix G. Marx/Natural History Museum of Los Angeles County, Los Angeles, USA

(1)

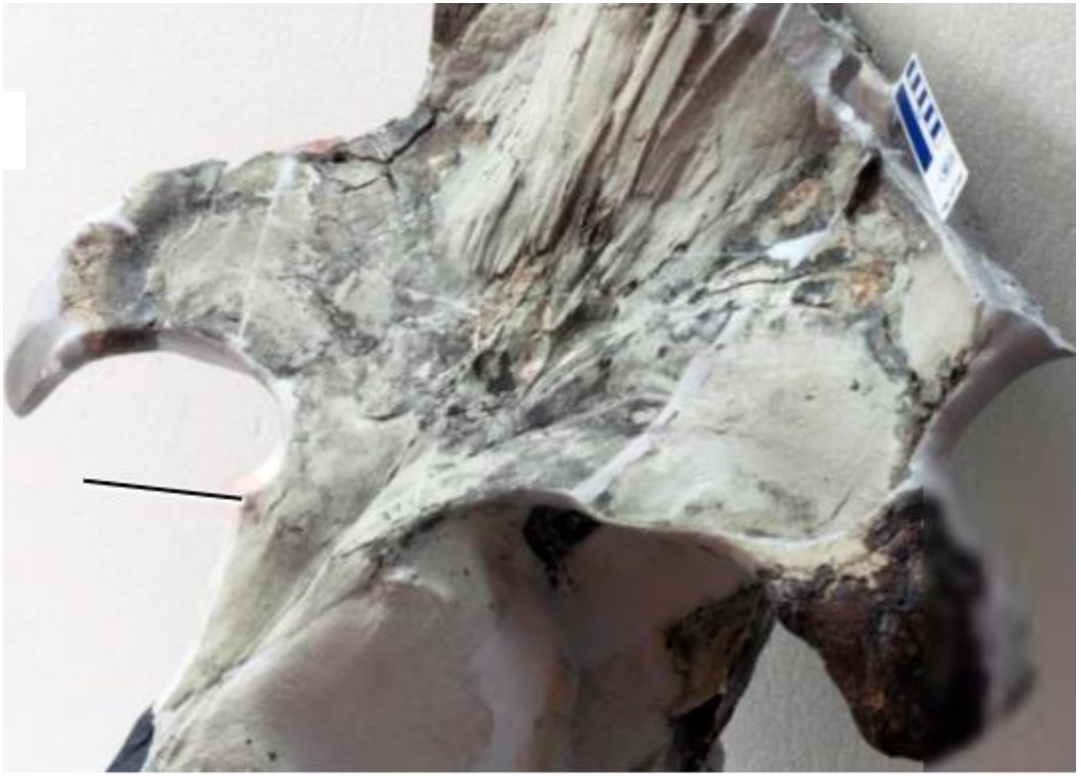

Llanocetus denticrenatus

Copyright holder: Felix G. Marx/ United States National Museum of Natural History, Washington DC, USA

[84] 'Outline of fronto-parietal suture'

(0) 'straight or lobate'

(1) 'frontals project posteriorly along the sagittal plane and partly separate the left and right parietals'

(2) 'highly irregular'

(0)

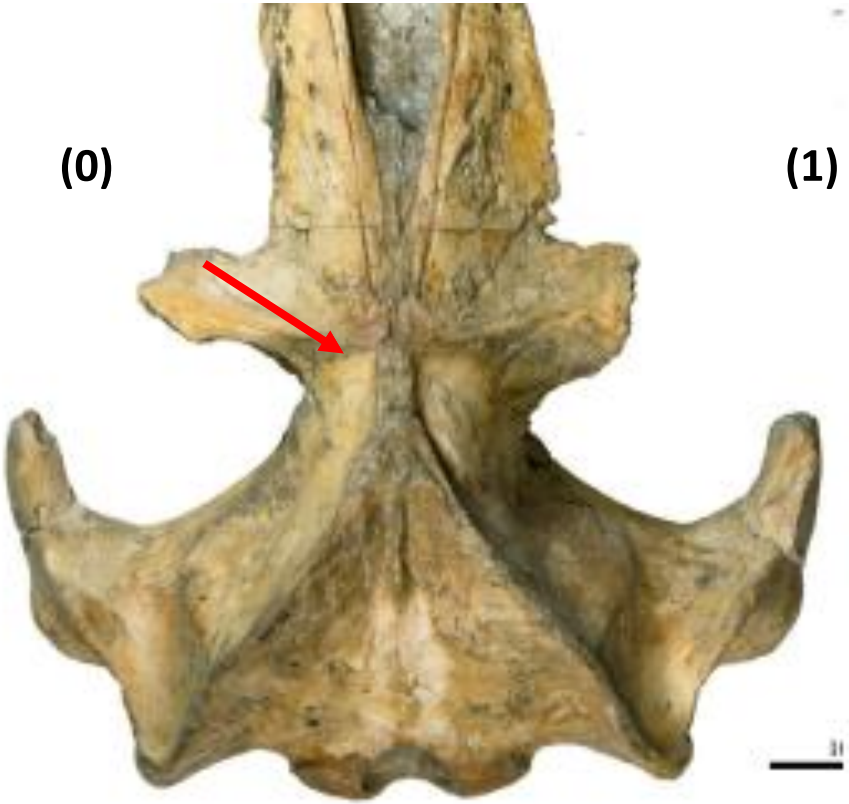

Tiucetus rosae

Adapted from: *"A new Miocene baleen whale from Peru deciphers the dawn of cetotheriids." Marx, Lambert, and De Muizon, 2017. Royal Society Open Science 4.9: 170560.*

(1)

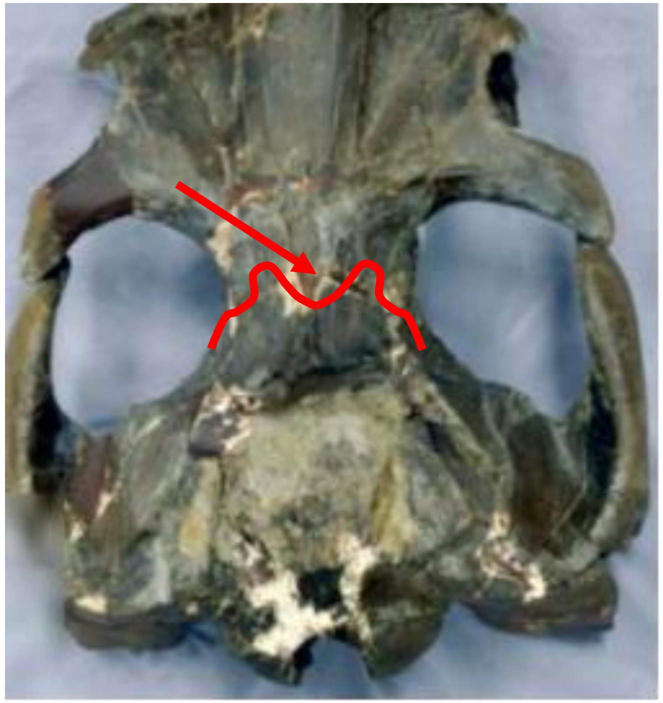

Aetiocetus weltoni

Copyright holder: Felix G. Marx/ University of California Museum of Paleontology, Berkeley, USA

(2)

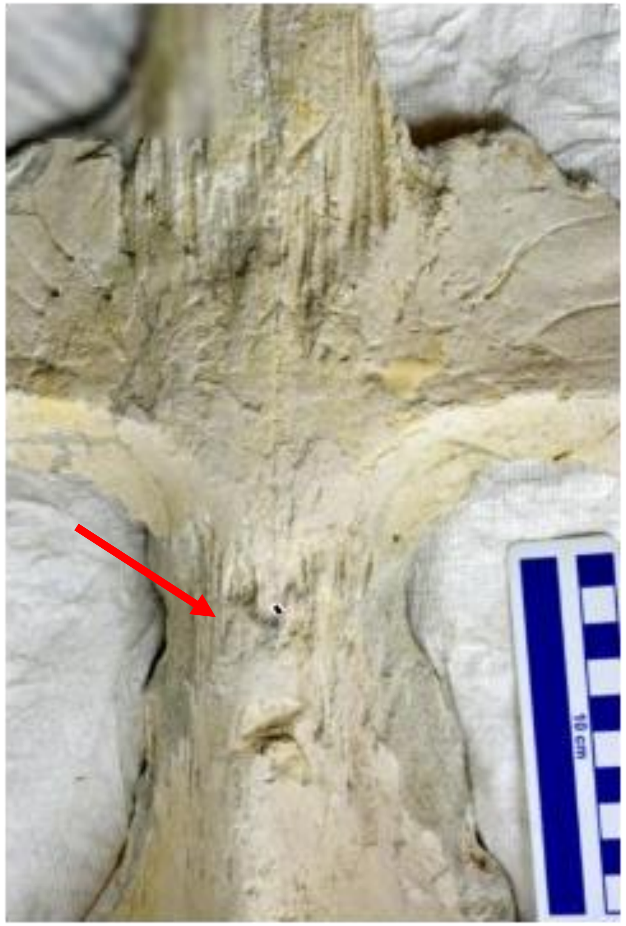

Waharoa ruwhenua

Copyright holder: Felix G. Marx/Otago University Geology Museum, Dunedin, New Zealand

[85] 'Parasagittal crest on parietal'

(0) 'absent'

(1) 'present'

(0)

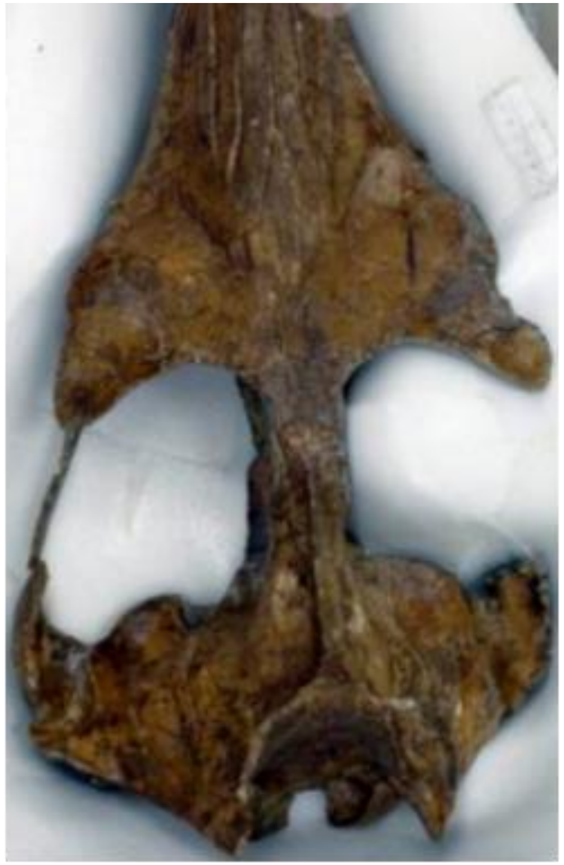

Zygorhiza kochii

Copyright holder: Felix G. Marx/ United States National Museum of Natural History, Washington DC, USA

(1)

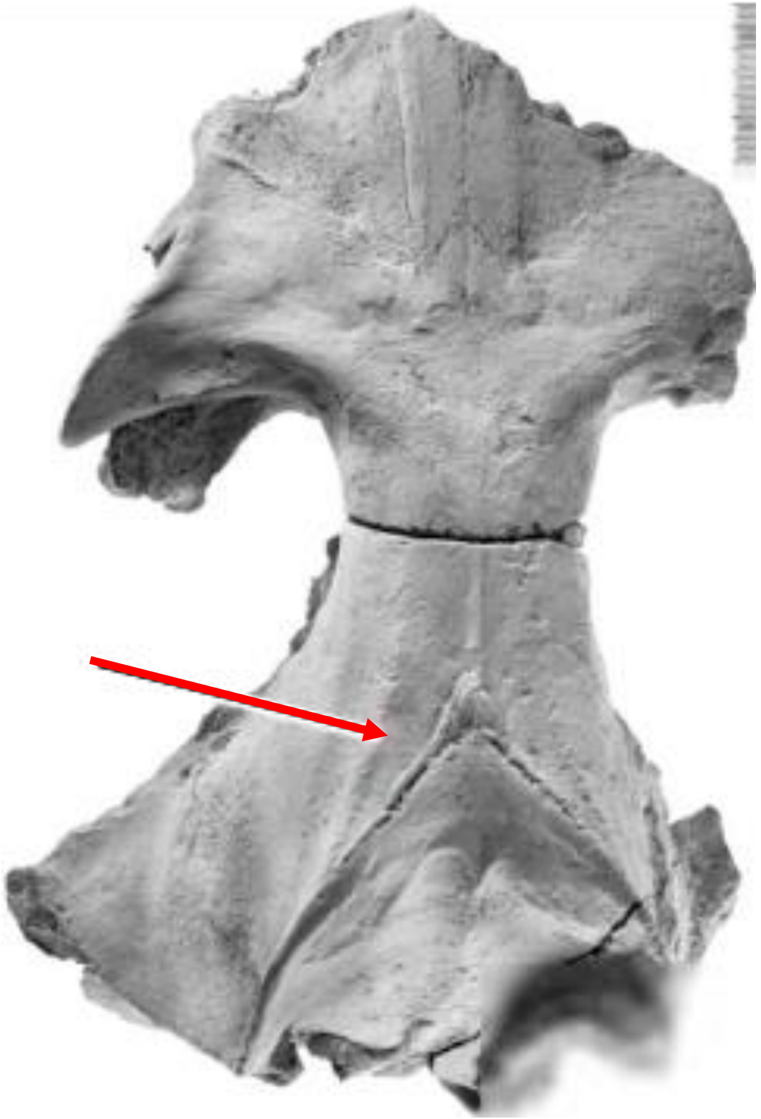

Fucaia buelli

Copyright holder: Felix G. Marx/Burke Museum, University of Washington, Seattle, USA

[86] 'Parietal in lateral view'

(0)'as long as high, or longer'

(1)'higher than long'

(0)

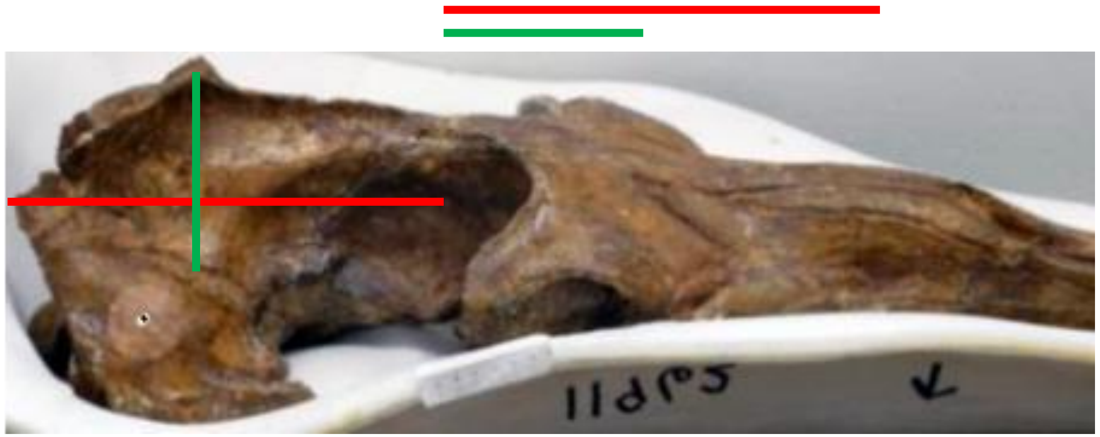

Zygorhiza kochii (mirrored)

Copyright holder: Felix G. Marx/ United States National Museum of Natural History, Washington DC, USA

(1)

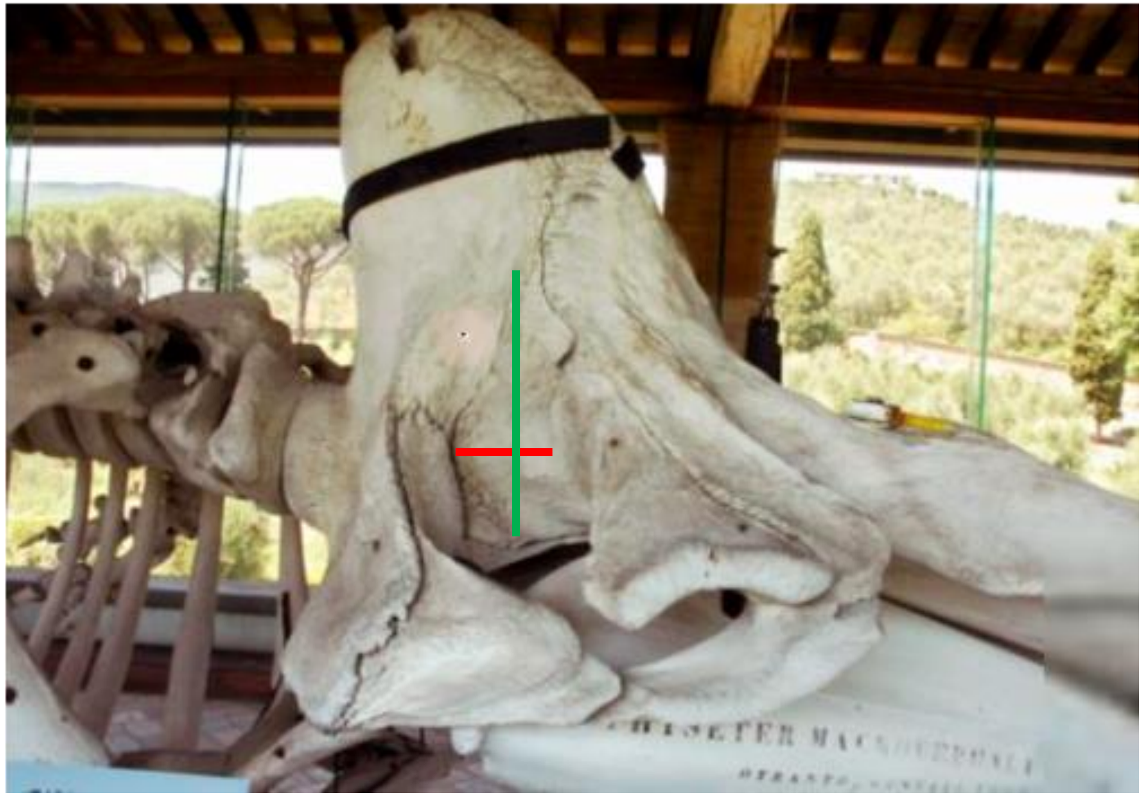

Physeter macrocephalus

Copyright holder: Felix G. Marx/Museo di Storia Naturale e del Territorio, Università di Pisa, Italy

[87] 'Spreading of anterolateral portion of parietal on to posteromedial corner of supraorbital process of frontal'

(0)'absent'

(1)'present'

(0)

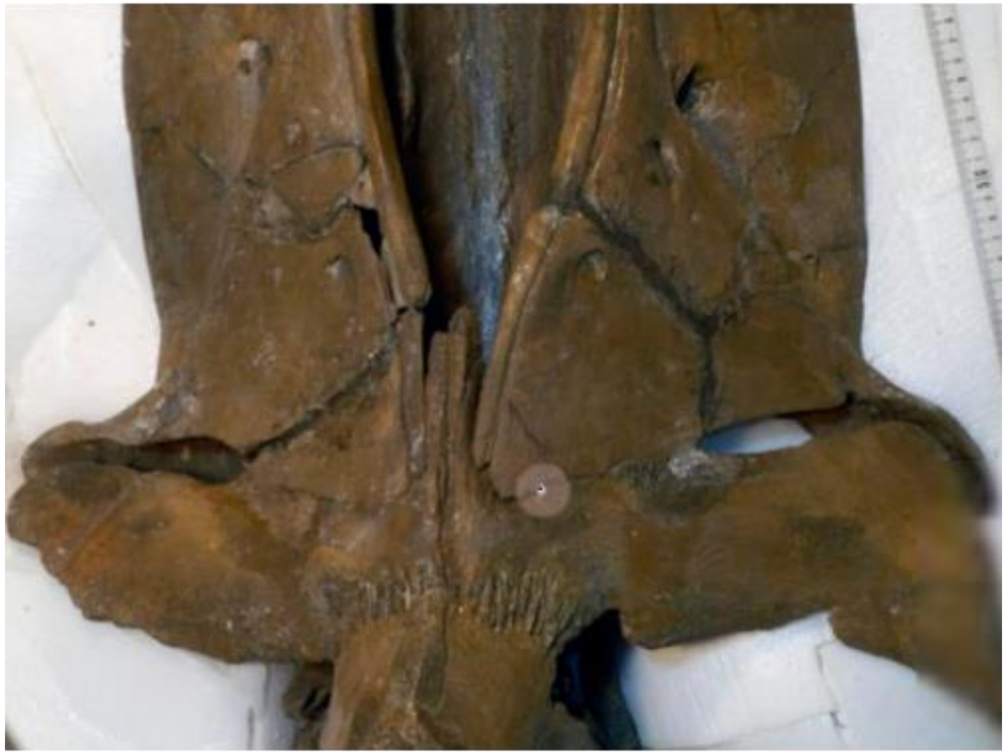

Diorocetus hiatus

Copyright holder: Felix G. Marx/ United States National Museum of Natural History, Washington DC, USA

(1)

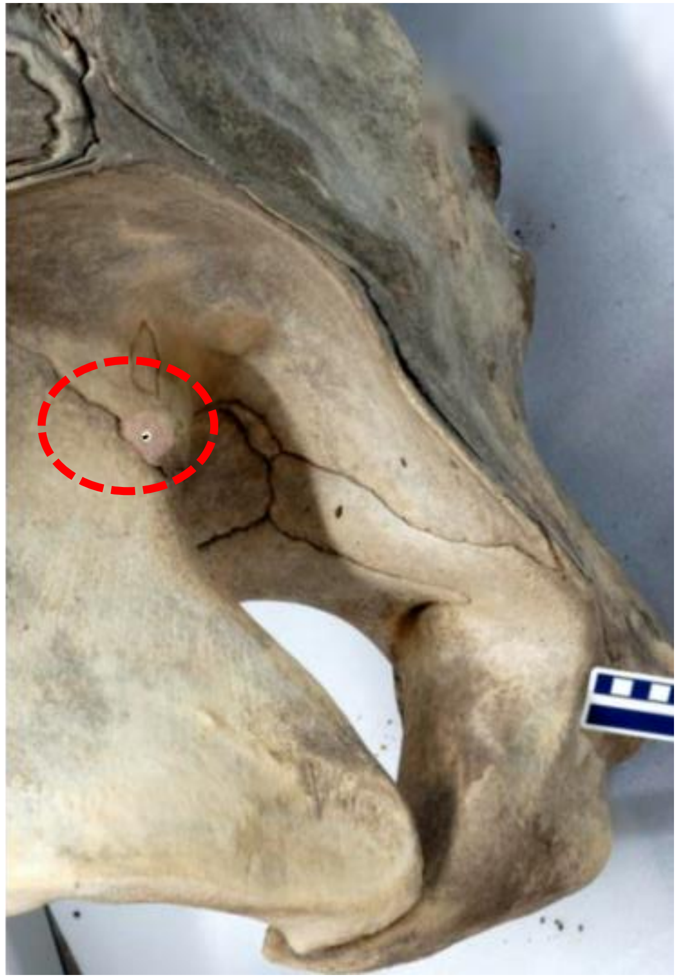

Balaenoptera acutorostrata

Copyright holder: Felix G. Marx/ The Charleston Museum, Charleston, South Carolina, USA

[88] 'Anteriormost point of parietal and interparietal'

(0)'more posterior than the posterior border of the ascending process of the maxilla'

(1)'more anterior than or in line with the posterior border of the ascending process of the maxilla'

(2)'as state 1 but the parietal being dorsoventrally concave and undercutting the maxilla and associated frontal'

(0)

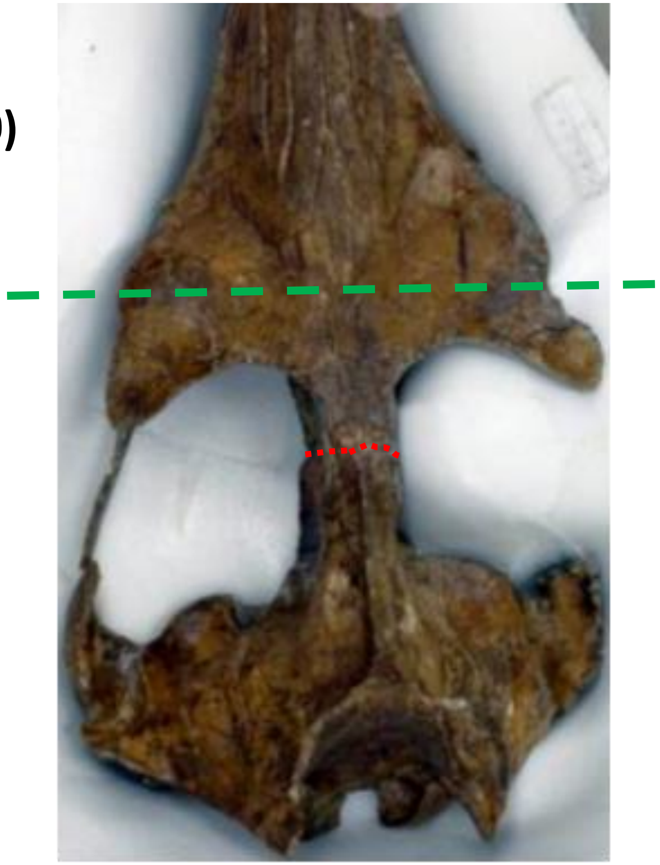

Zygorhiza kochii

Copyright holder: Felix G. Marx/ United States National Museum of Natural History, Washington DC, USA

(1)

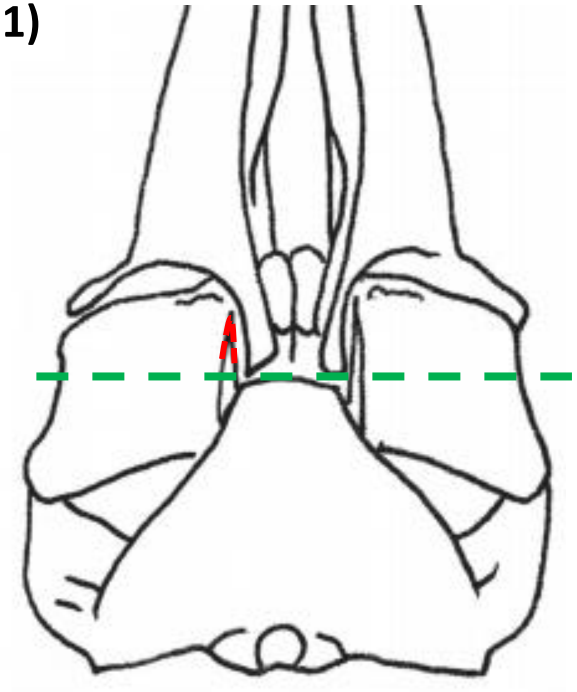

Balaenoptera acutorostrata

Adapted from: "Taxonomy and evolution of the Italian Pliocene Mysticeti (Mammalia, Cetacea): a state of the art", Bisconti, 2009.

(2)

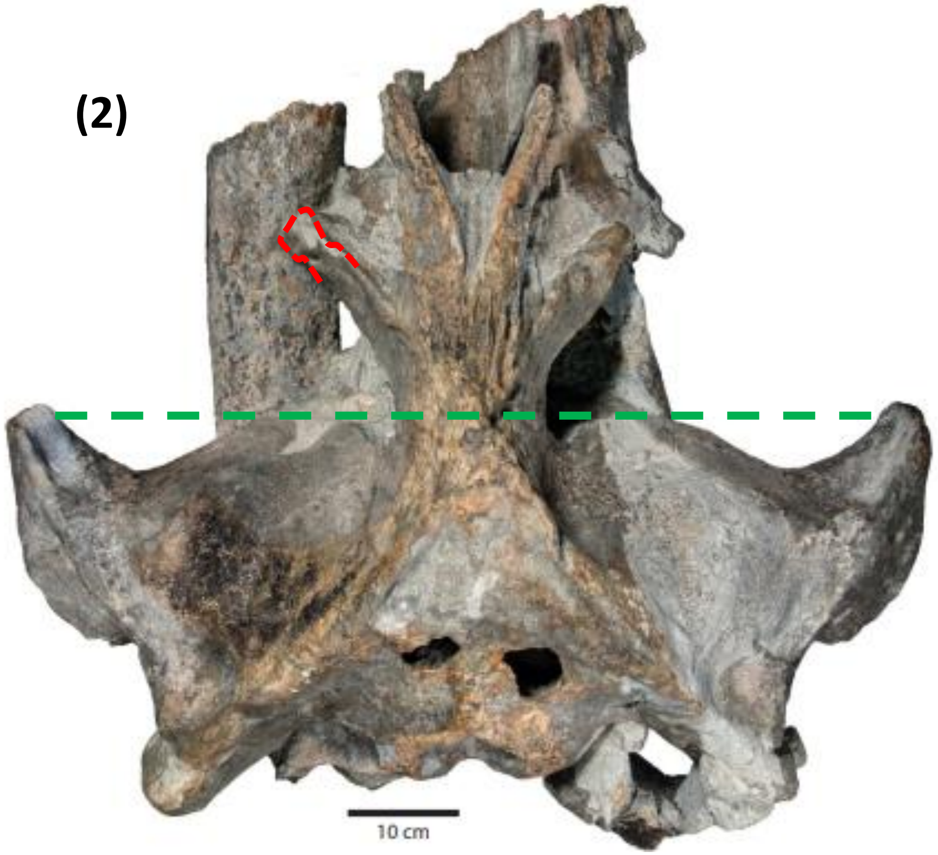

Tranatocetus maregermanicum

Adapted from: "A large Late Miocene cetotheriid (Cetacea, Mysticeti) from the Netherlands clarifies the status of Tranatocetidae."Marx, et al. 2019. *PeerJ* 7: e6426.

[89] 'Anteriormost point of supraoccipital shield in dorsal view'

(0)'located posterior to or in line with the anterior border of the squamosal fossa'

(1)'in line with temporal fossa, but posterior to the apex of the zygomatic process of the squamosal'

(2)'in line with or located anterior to the level of the apex of the zygomatic process of the squamosal'

(3)'in line with the anterior half or anterior edge of the supraorbital process'

(0)

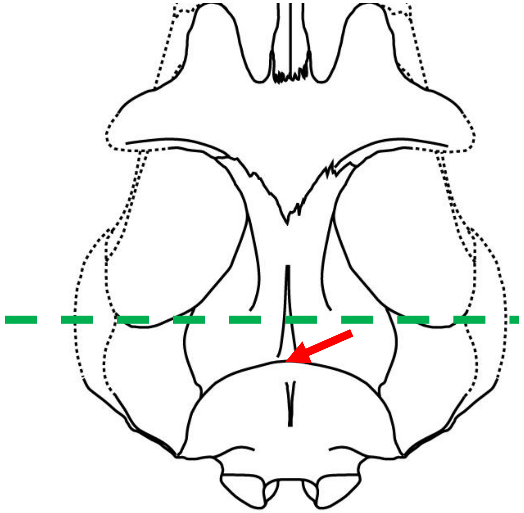

Mammalodon colliveri

Copyright holder: Erich M. G. Fitzgerald

(1)

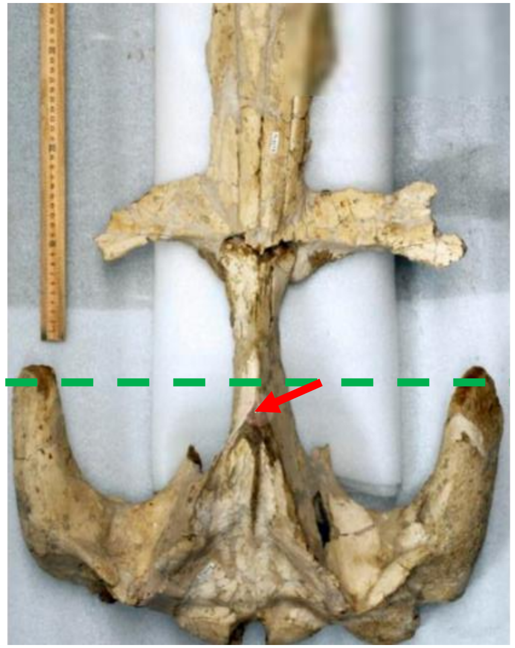

Eomysticetus whitmorei

Copyright holder: Felix G. Marx/ The Charleston Museum, Charleston, South Carolina, USA

(2)

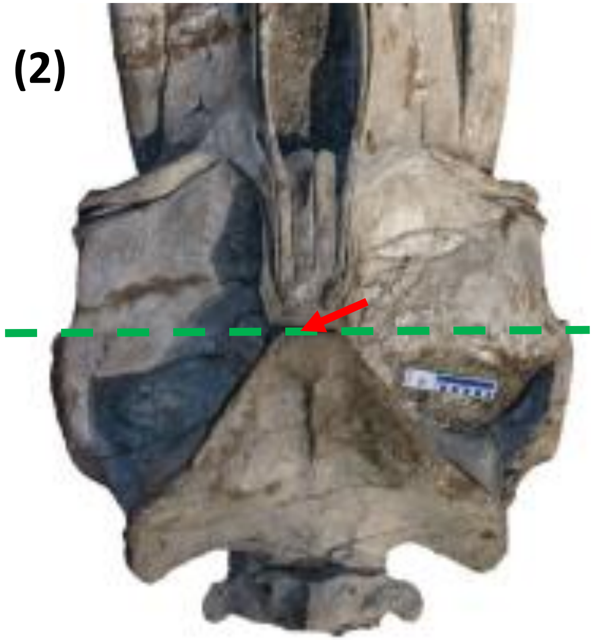

Incakujira anillodefuego

Adapted from: "A new Miocene baleen whale from the Peruvian desert." Marx and Kohno, 2016. *Royal Society Open Science* 3.10 (2016): 160542.

(3)

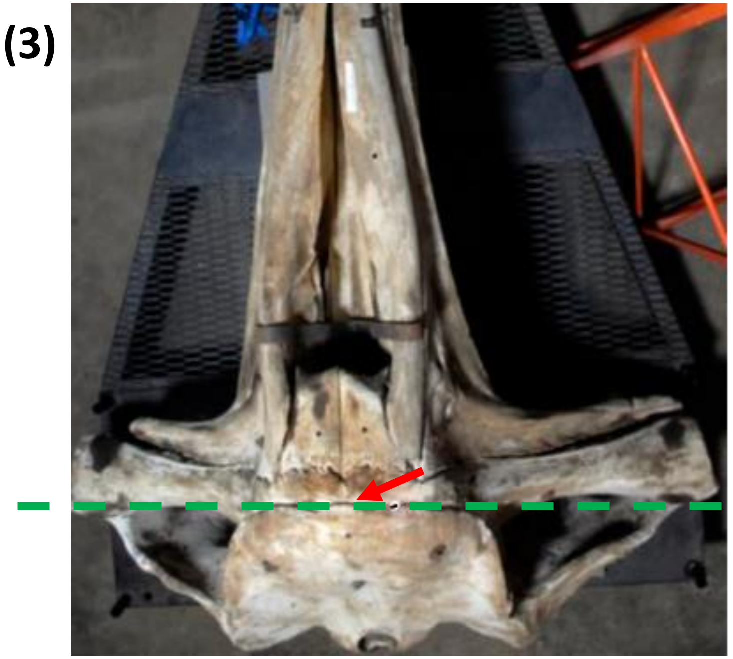

Eubalaena spp.

Copyright holder: Felix G. Marx/ United States National Museum of Natural History, Washington DC, USA

[90] 'Anteroposterior position of posterior apex of nuchal crest'

(0) 'posterior to the occipital condyle’

(1) 'anterior to or in line with the posteriormost point of the occipital condyle'

(0)

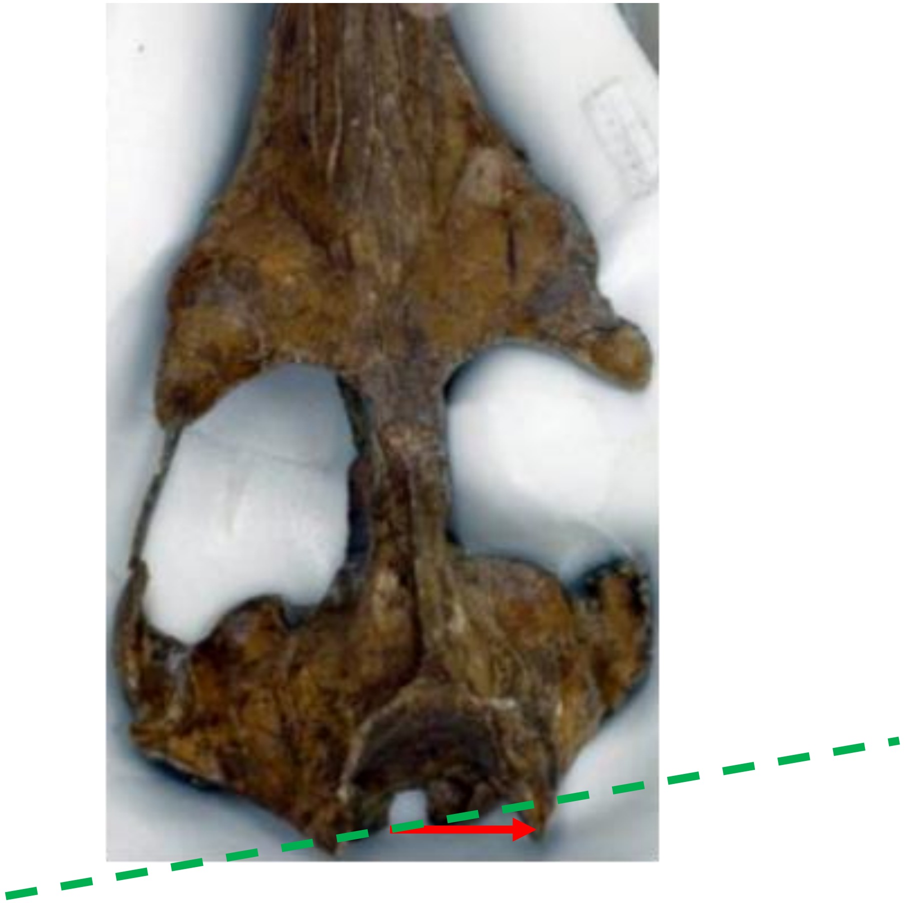

Zygorhiza kochii

Copyright holder: Felix G. Marx/ United States National Museum of Natural History, Washington DC, USA

(1)

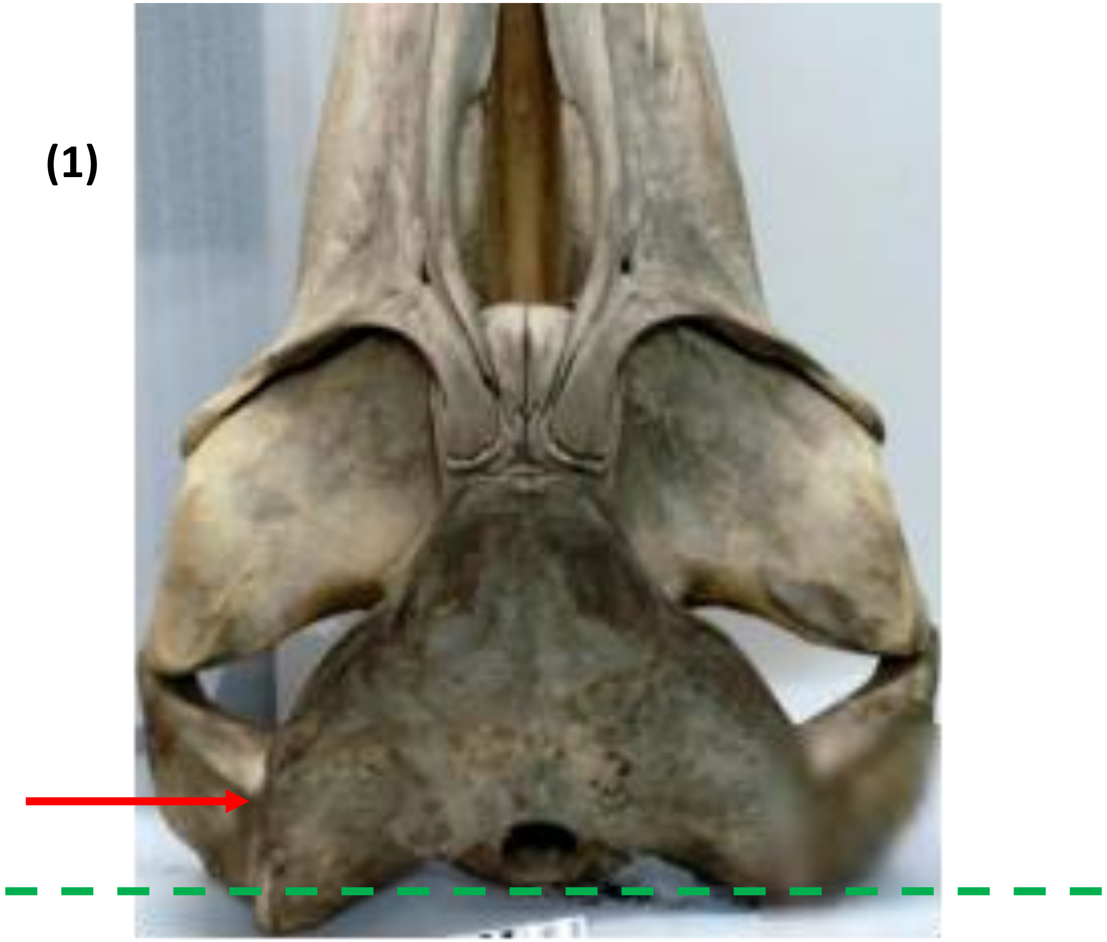

Balaenoptera acutorostrata

Copyright holder: Felix G. Marx/ The Charleston Museum, Charleston, South Carolina, USA

[91] 'Transverse position of posterior apex of nuchal crest'

(0) 'aligned with proximal two thirds of the temporal fossa'

(1) 'aligned with the distal one third of the temporal fossa'

(0)

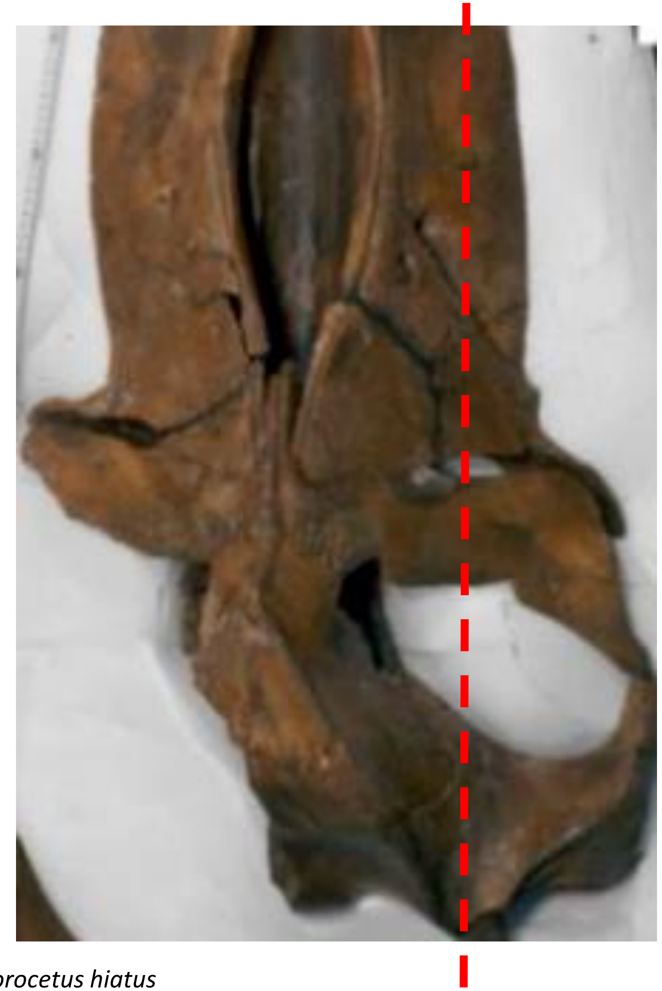

Diorocetus hiatus

Copyright holder: Felix G. Marx/ United States National Museum of Natural History, Washington DC, USA

(1)

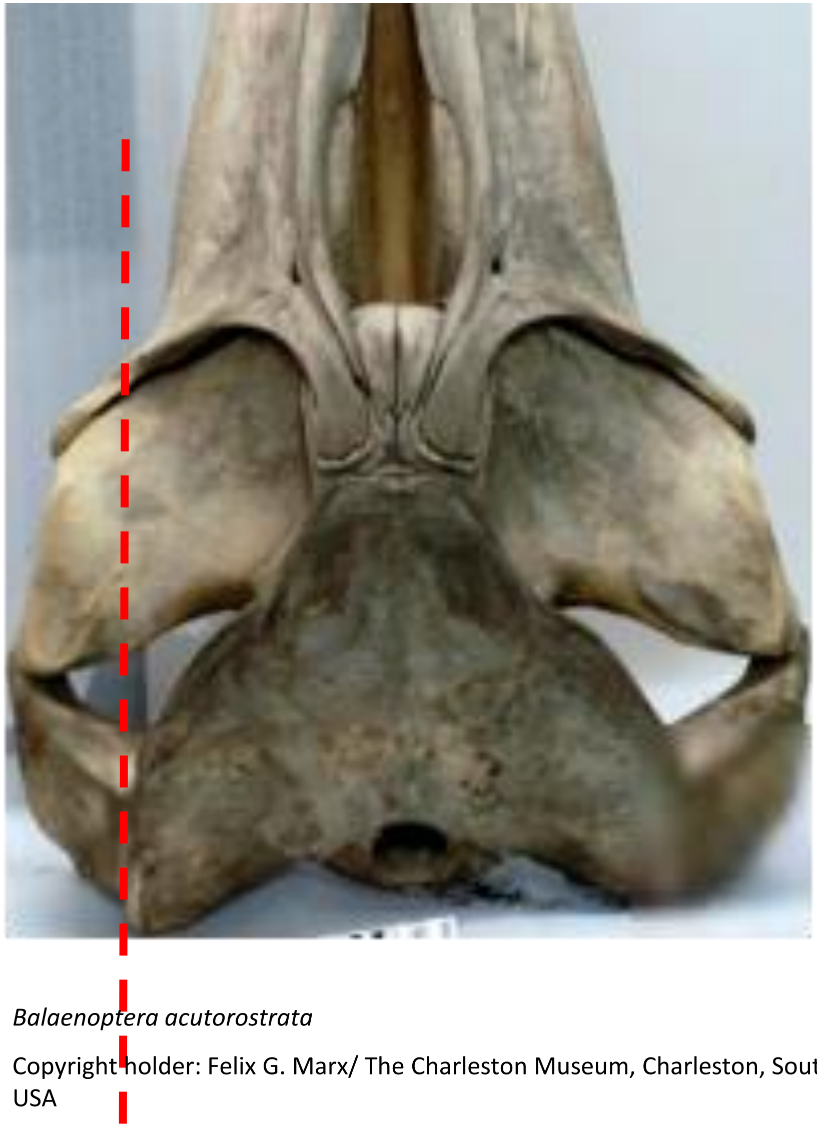

Balaenoptera acutorostrata

Copyright holder: Felix G. Marx/ The Charleston Museum, Charleston, South Carolina, USA

[92] 'Distinct nuchal tubercle at junction of parieto-squamosal suture and supraoccipital'

(0)'absent'

(1)'present'

(0)

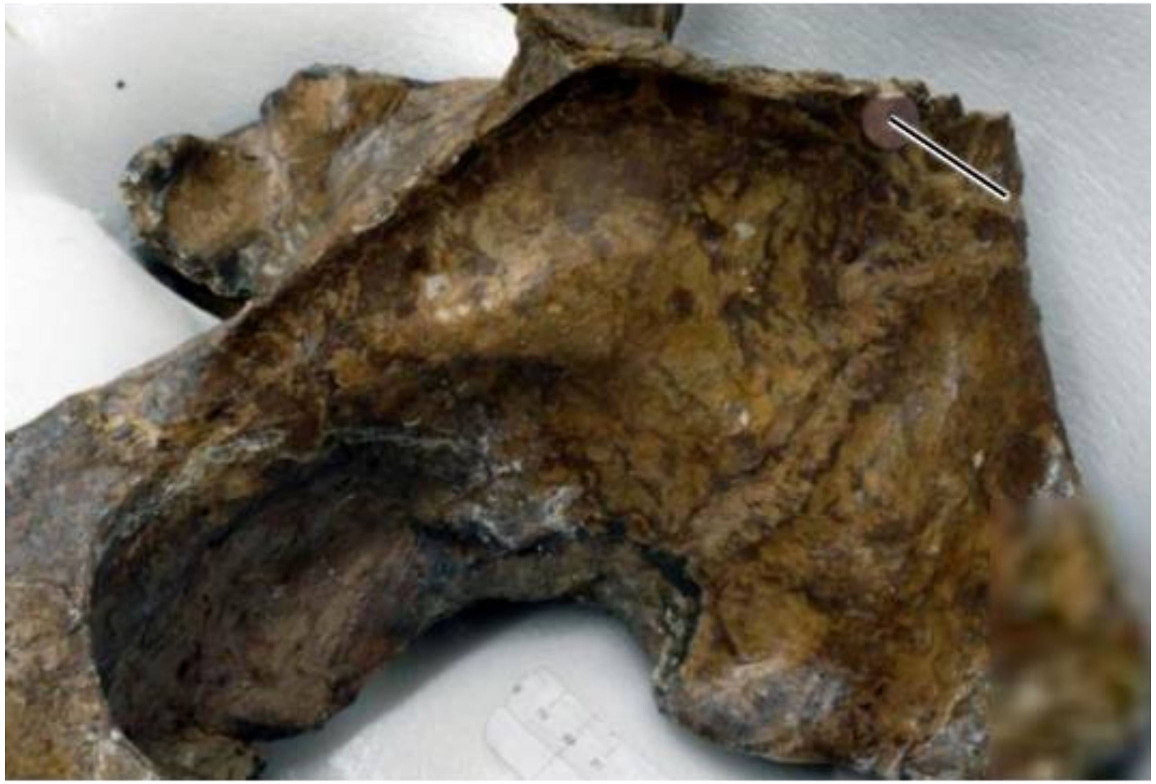

Zygorhiza kochii

Copyright holder: Felix G. Marx/ United States National Museum of Natural History, Washington DC, USA

(1)

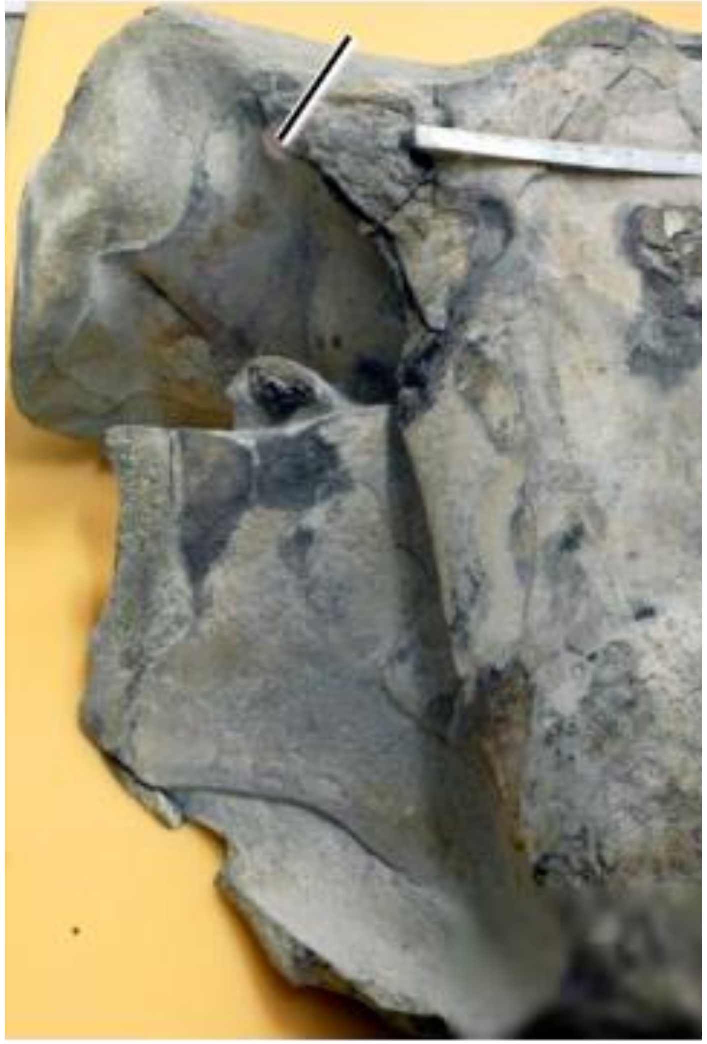

Miocaperea pulchra

Copyright holder: Felix G. Marx/ Staatliches Museum für Naturkunde, Stuttgart, Germany

[93] 'Exposure of alisphenoid in or at ventral border of temporal fossa'

(0)'exposed on temporal wall of skull and contributing to orbital fissure’

(1)'alisphenoid separated from orbital fissure or not exposed on temporal skull wall'

(0)

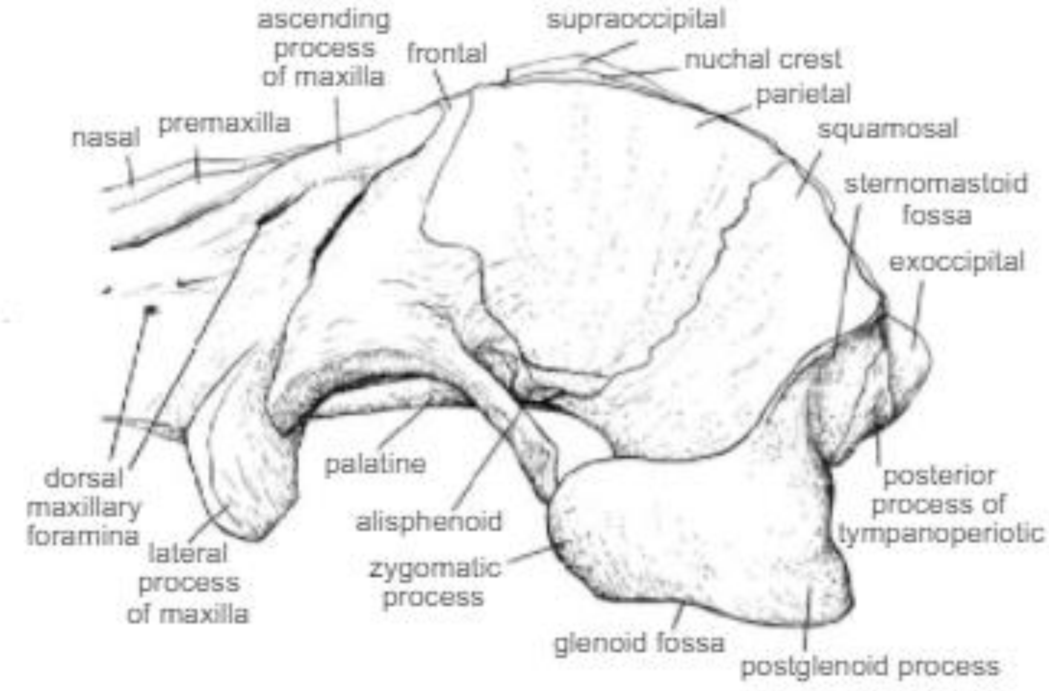

Cetotherium riabinini

(1)

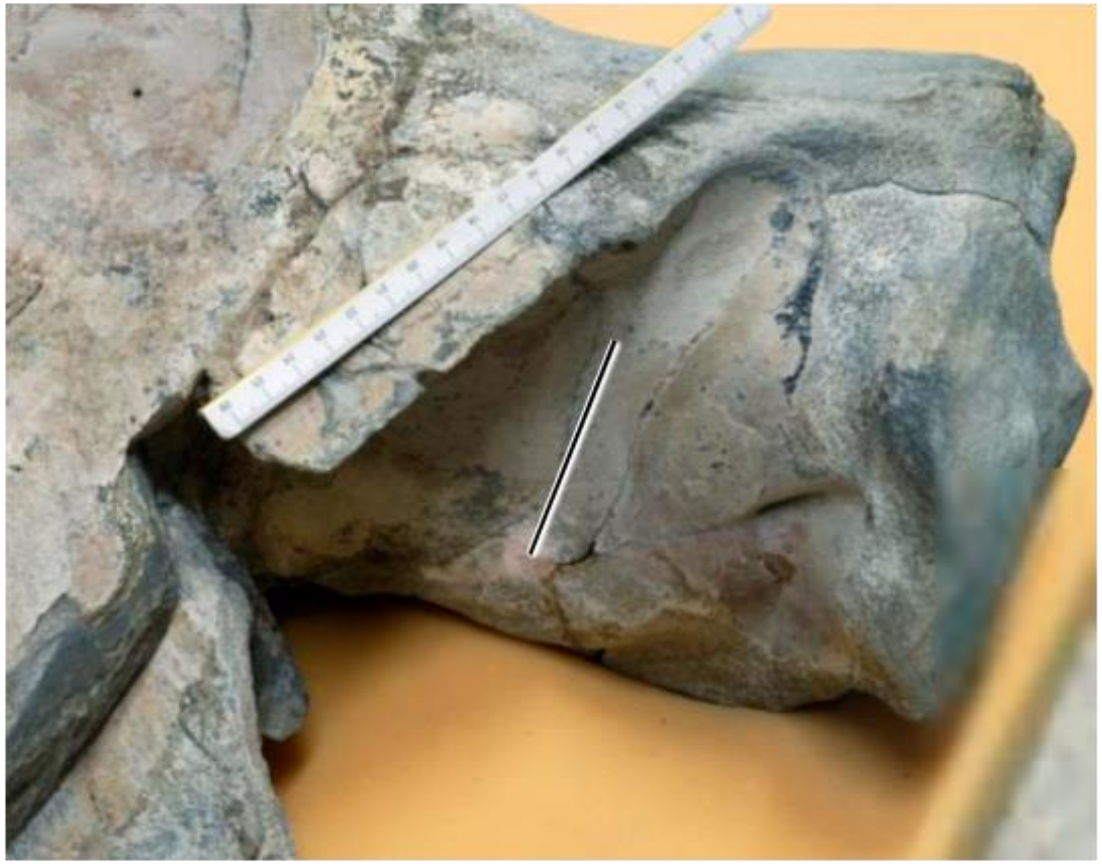

Miocaperea pulchra

Adapted from: "The anatomy of the Late Miocene baleen whale Cetotherium riabinini from Ukraine." Gol’din et al., 2013. *Acta Palaeontologica Polonica* 59.4: 795-814.

Copyright holder: Felix G. Marx/ Staatliches Museum für Naturkunde, Stuttgart, Germany

[94] 'Large opening located at junction of parietal and squamosal'

(0)'absent'

(1)'present'

(0)

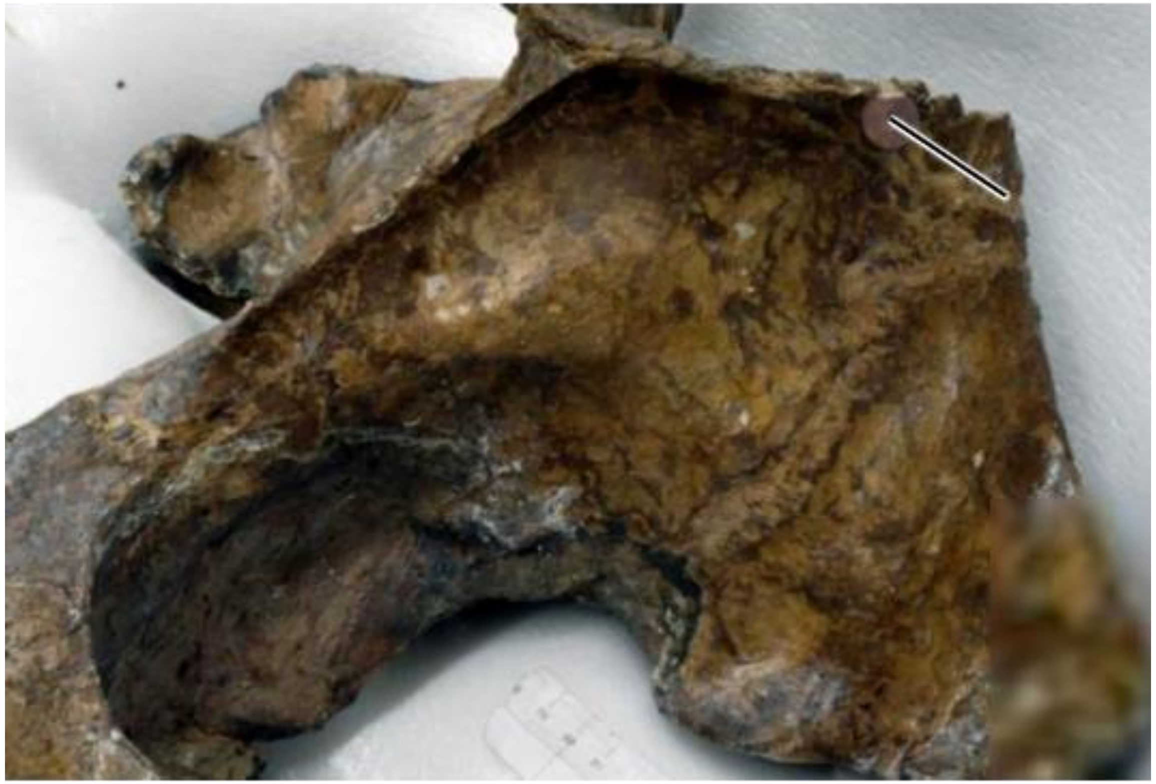

Zygorhiza kochii

Copyright holder: Felix G. Marx/ United States National Museum of Natural History, Washington DC, USA

(1)

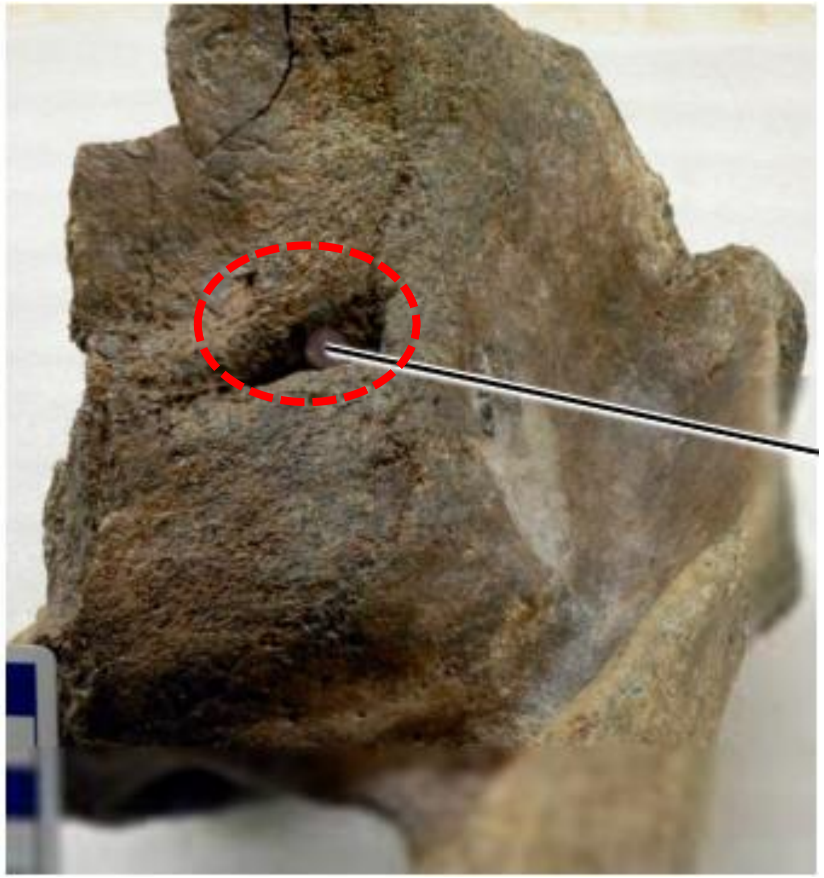

Herpetocetus bramblei

Copyright holder: Felix G. Marx/ University of California Museum of Paleontology, Berkeley, USA

[95] 'Zygomatic process of squamosal extremely well developed and robust in dorsal view'

(0) 'absent'

(1) 'present'

(0)

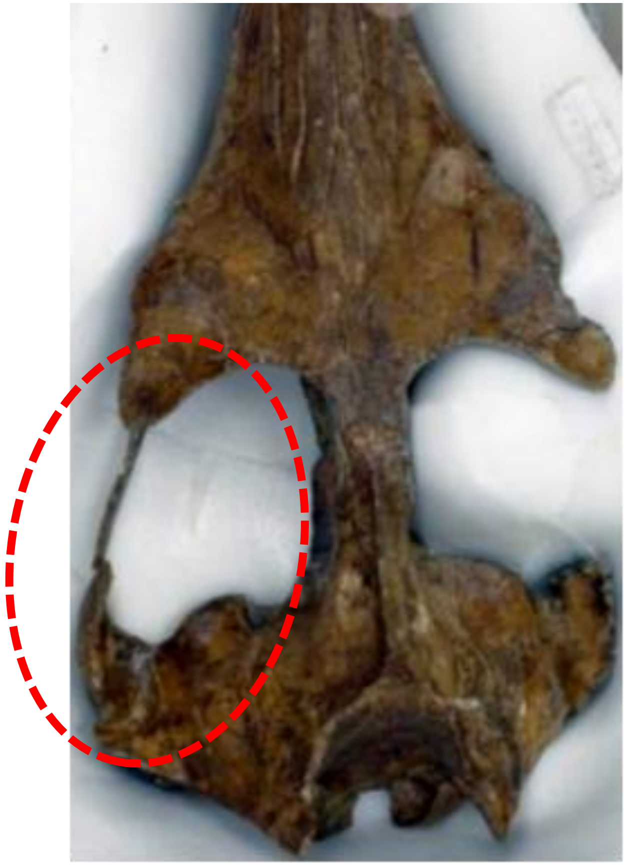

Zygorhiza kochii

Copyright holder: Felix G. Marx/ United States National Museum of Natural History, Washington DC, USA

(1)

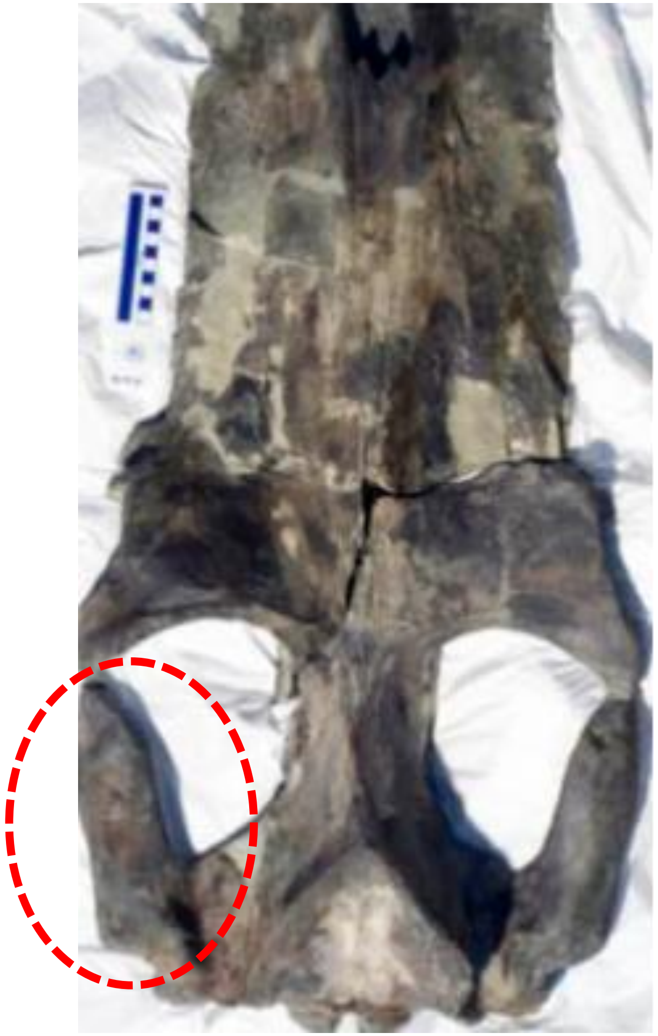

Yamatocetus canaliculatus

Copyright holder: Felix G. Marx/ Kitakyushu Museum of Natural and Human History, Kitakyushu, Kyushu, Japan

[96] 'Zygomatic process of squamosal dorsoventrally expanded in lateral view'

(0) 'absent'

(1) 'present'

(0)

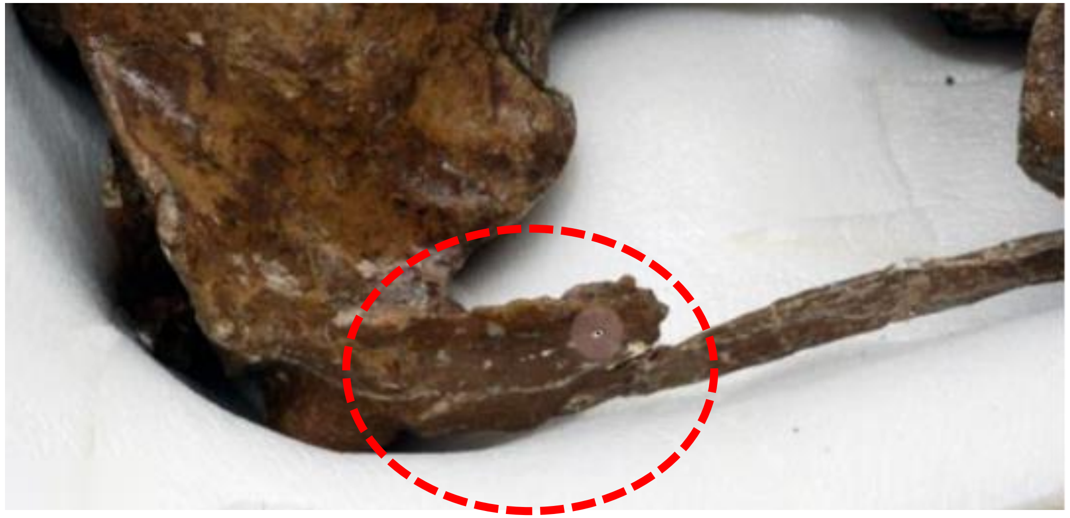

Zygorhiza kochii (mirrored)

Copyright holder: Felix G. Marx/ United States National Museum of Natural History, Washington DC, USA

(1)

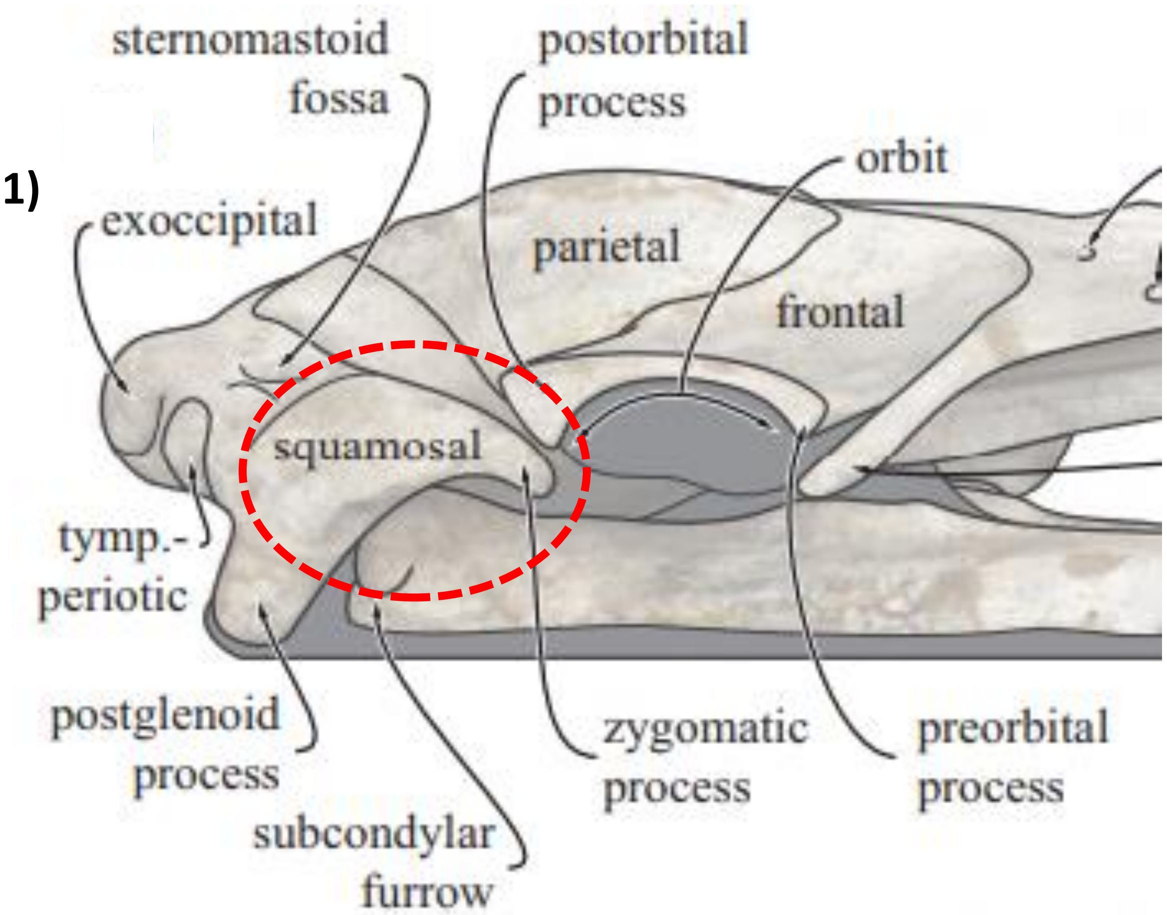

Incakujira anillodefuego

Adapted from: "A new Miocene baleen whale from the Peruvian desert." Marx and Kohno, 2016. *Royal Society Open Science* 3.10 (2016): 160542.

[97] 'Orientation of zygomatic process of squamosal in dorsal view'

(0)'directed anteromedially'

(1)'directed anteriorly or slightly anterolaterally'

(2)'directed anterolaterally'

(0)

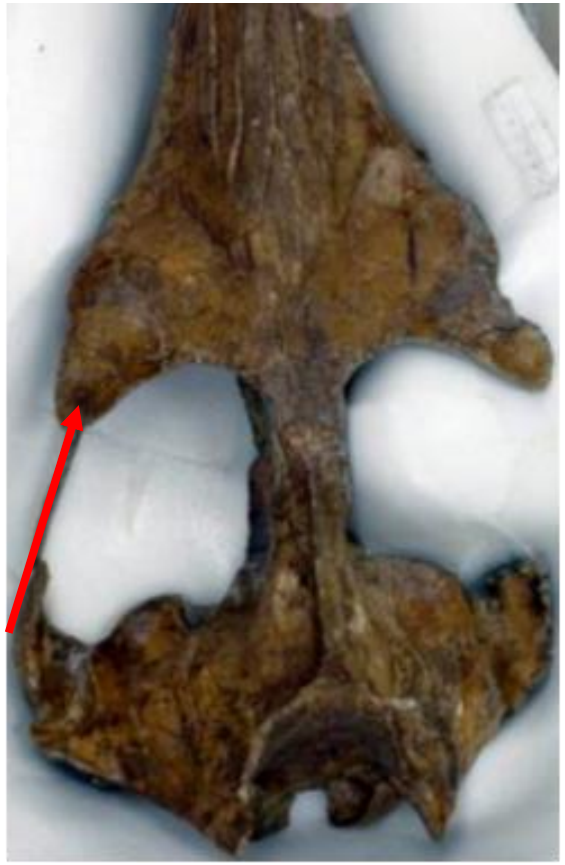

Zygorhiza kochii

Copyright holder: Felix G. Marx/ United States National Museum of Natural History, Washington DC, USA

(1)

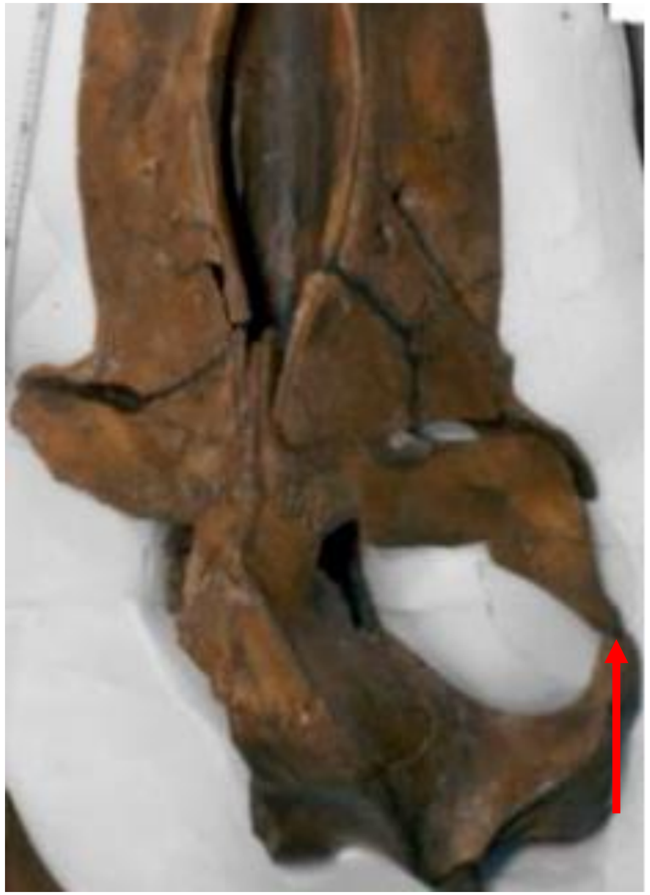

Diorocetus hiatus

Copyright holder: Felix G. Marx/ United States National Museum of Natural History, Washington DC, USA

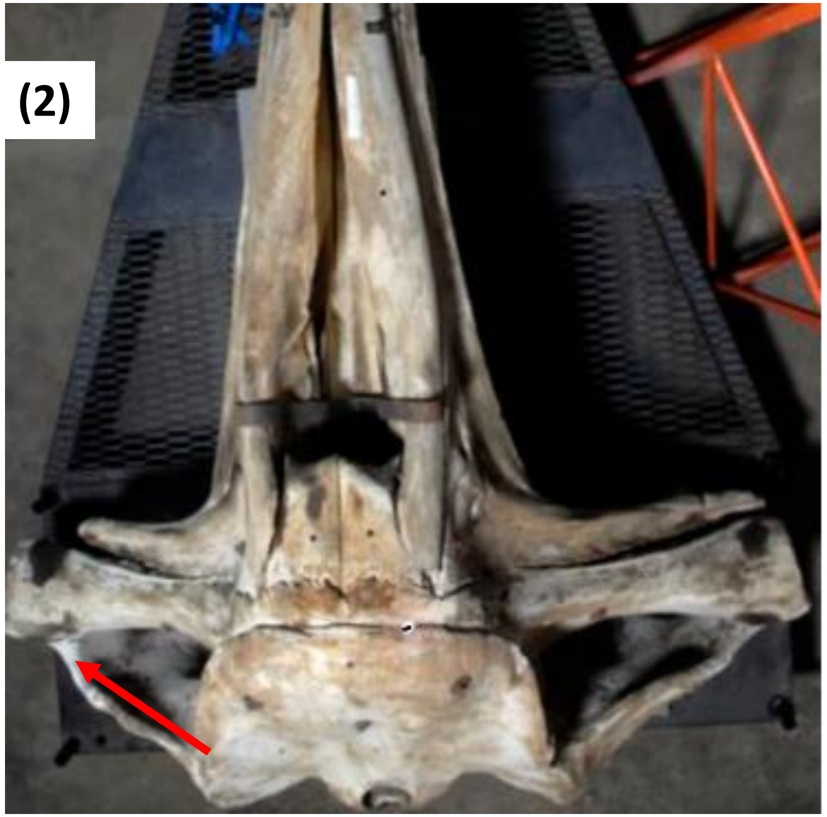

Eubalaena spp.

Copyright holder: Felix G. Marx/ United States National Museum of Natural History, Washington DC, USA

[98] 'Zygomatic process of squamosal in lateral or ventral view'

(0)'tapering anteriorly'

(1)'expanded anteriorly, thus forming a central constriction'

(0)

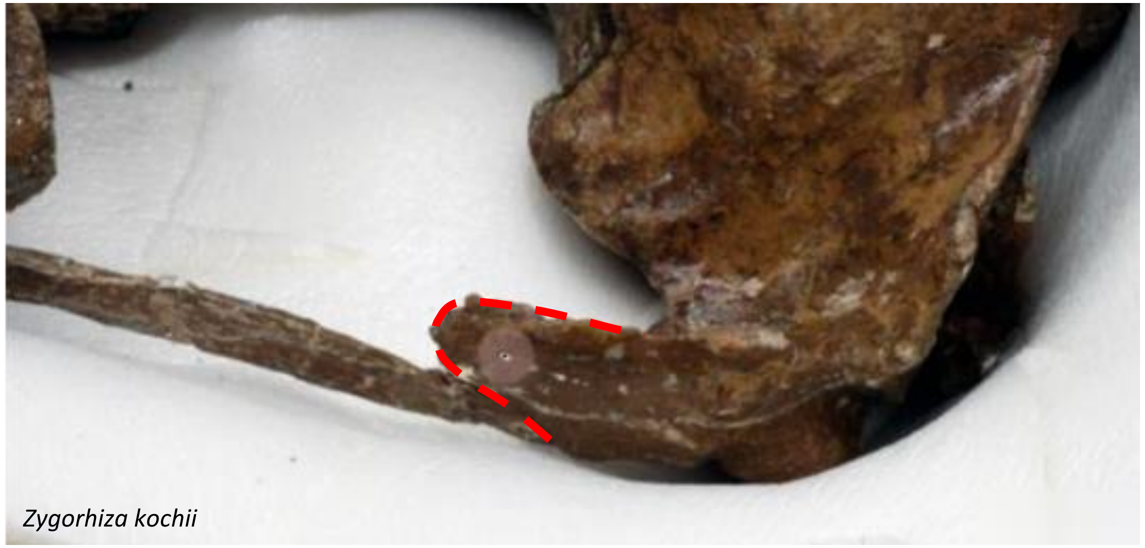

Copyright holder: Felix G. Marx/ United States National Museum of Natural History, Washington DC, USA

(1)

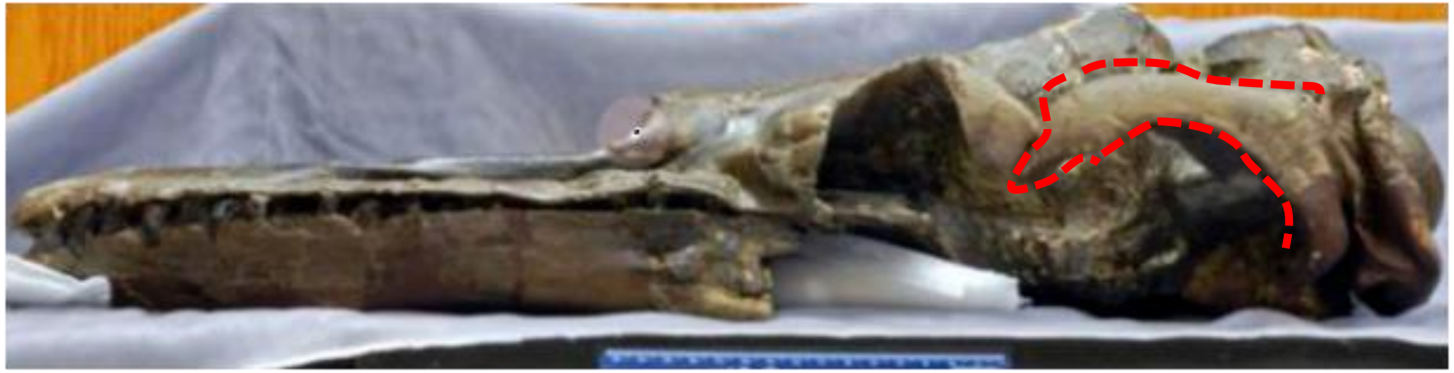

Aetiocetus weltoni

Copyright holder: Felix G. Marx/ University of California Museum of Paleontology, Berkeley, USA

[99] 'Twisting of zygomatic process of squamosal'

(0)'partially twisted anticlockwise on the left and clockwise on the right'

(1)'absent'

(2)'partially twisted clockwise on the left and anticlockwise on the right'

(3)'as state 1 but with the process twisted almost 90 degrees'

(0)

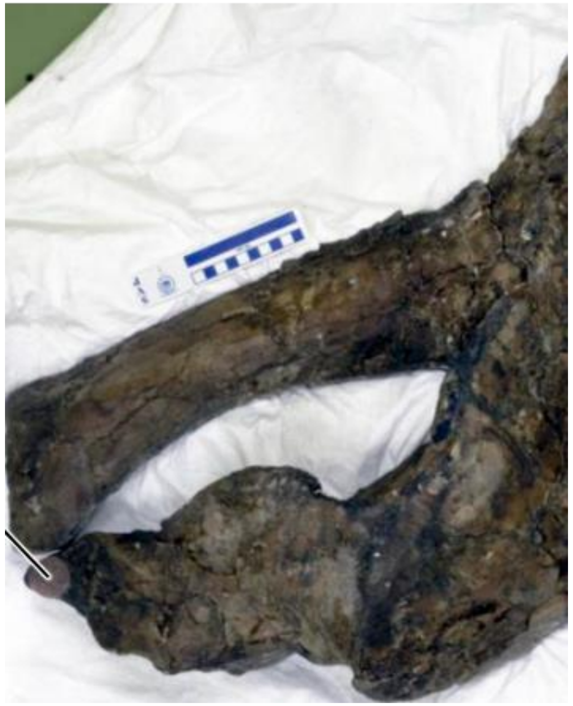

Balaenula sp.

Copyright holder: Felix G. Marx/ Sapporo Museum Activity Centre, Sapporo, Japan

(1)

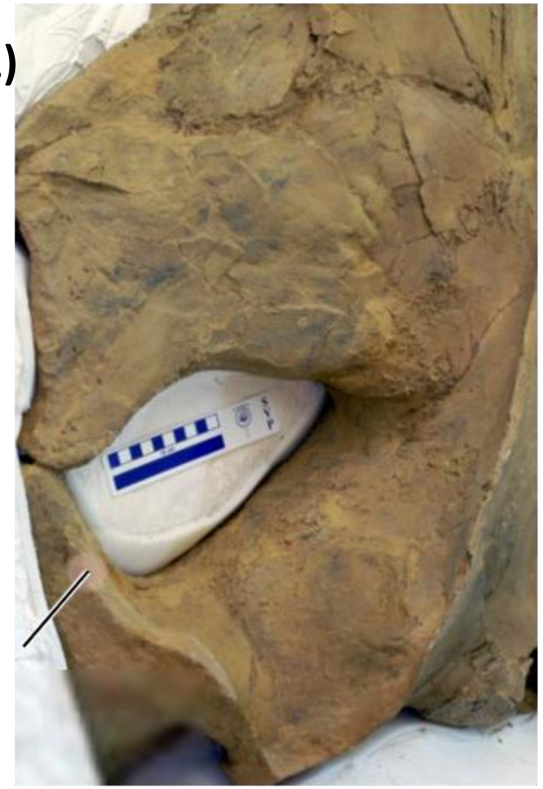

Aglaocetus patulus

Copyright holder: Felix G. Marx/ United States National Museum of Natural History, Washington DC, USA

(2)

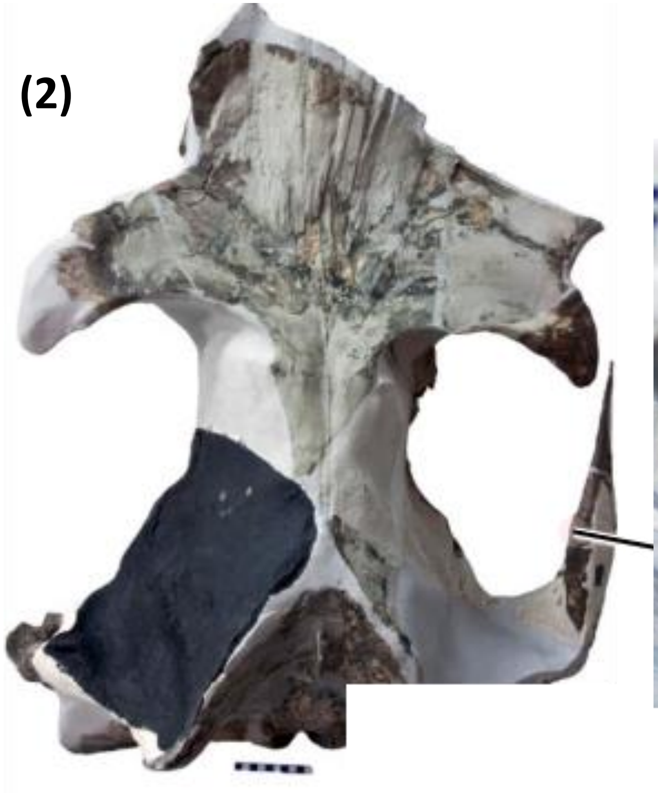

Llanocetus denticrenatus

Copyright holder: Felix G. Marx/ United States National Museum of Natural History, Washington DC, USA

(3)

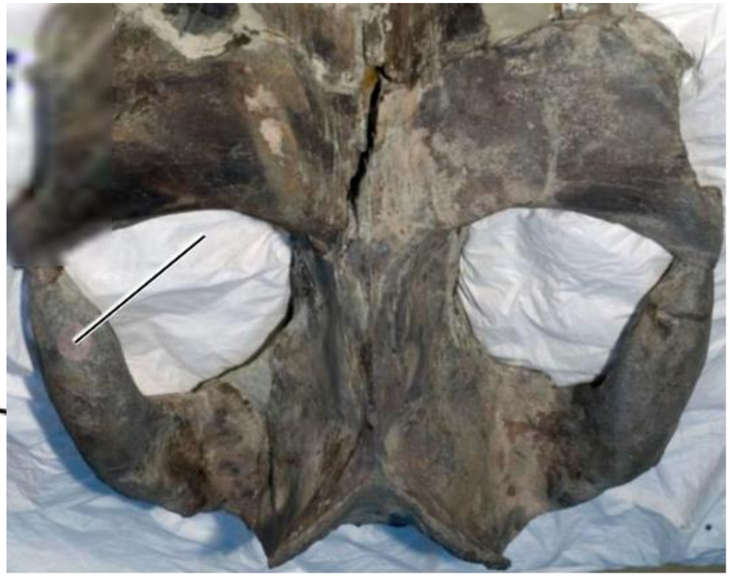

Yamatocetus canaliculatus

Copyright holder: Felix G. Marx/ Kitakyushu Museum of Natural and Human History, Kitakyushu, Kyushu, Japan

[100] 'Apex of zygomatic process of squamosal deflected anteroventrally'

(0) 'absent'

(1) 'present'

(0)

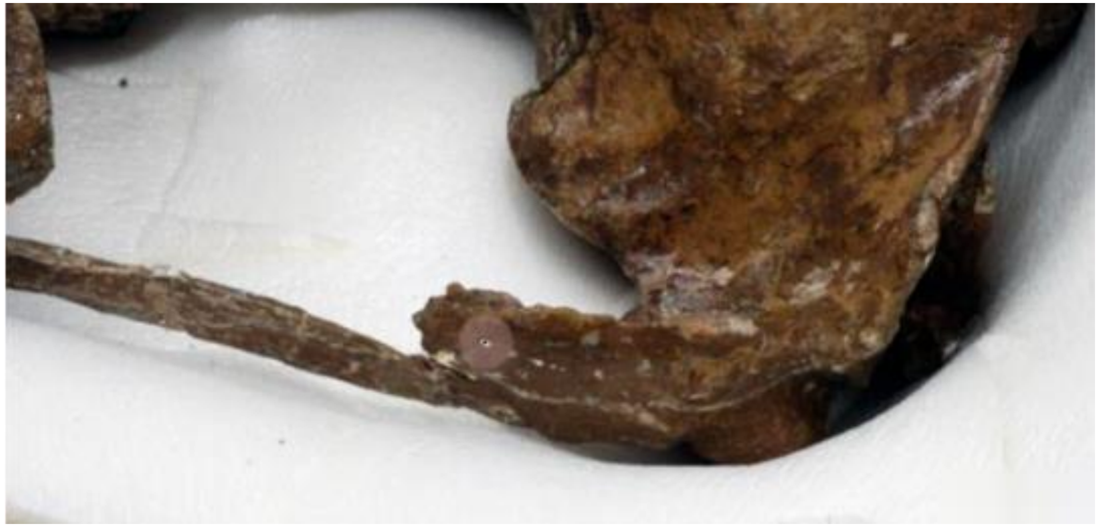

Zygorhiza kochii

Copyright holder: Felix G. Marx/ United States National Museum of Natural History, Washington DC, USA

(1)

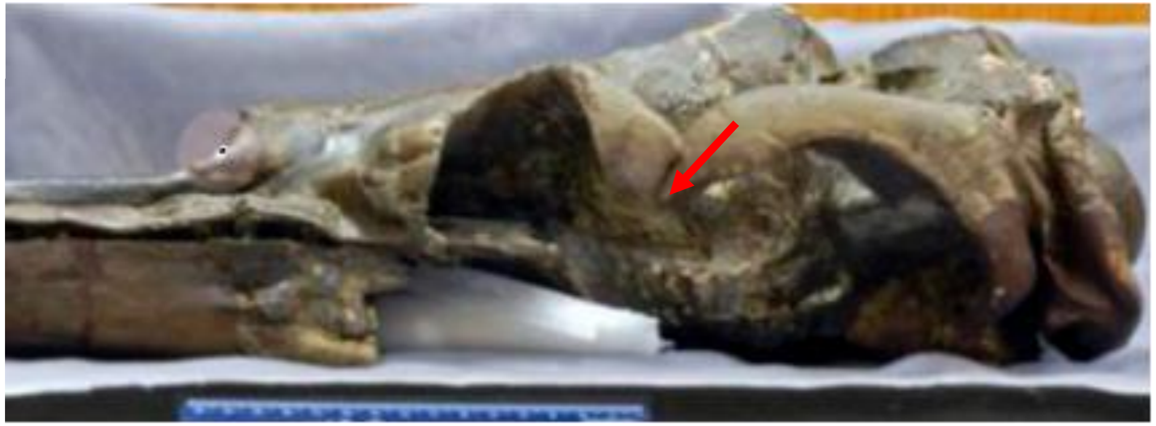

Aetiocetus weltoni

Copyright holder: Felix G. Marx/ University of California Museum of Paleontology, Berkeley, USA

[101] 'Supramastoid crest of zygomatic process of squamosal'

(0) 'present'

(1) 'present on posterior portion of zygomatic process only'

(2) 'absent'

(0)

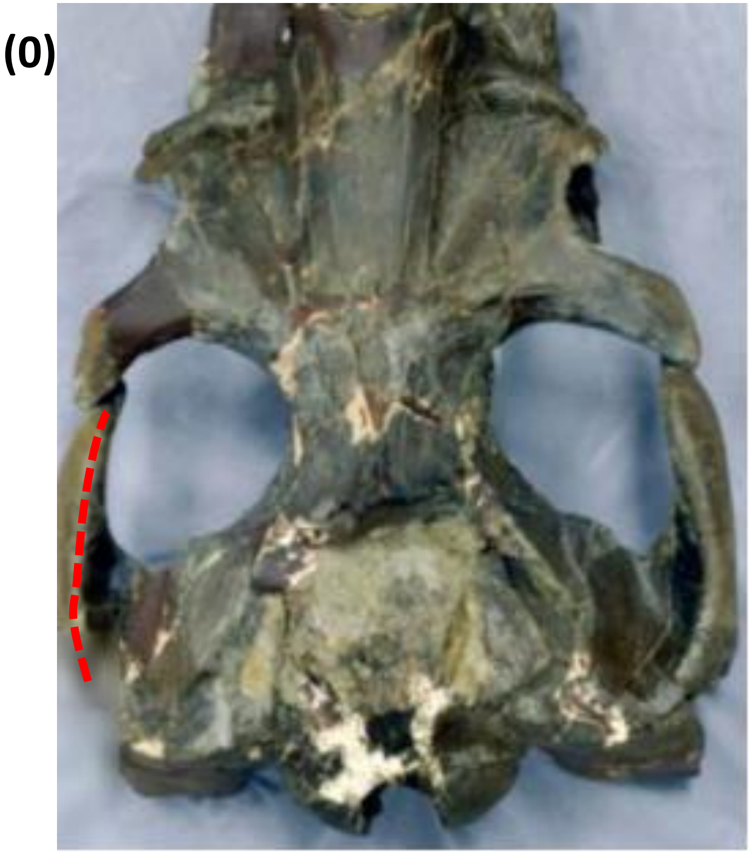

Aetiocetus weltoni

Copyright holder: Felix G. Marx/ University of California Museum of Paleontology, Berkeley, USA

(1)

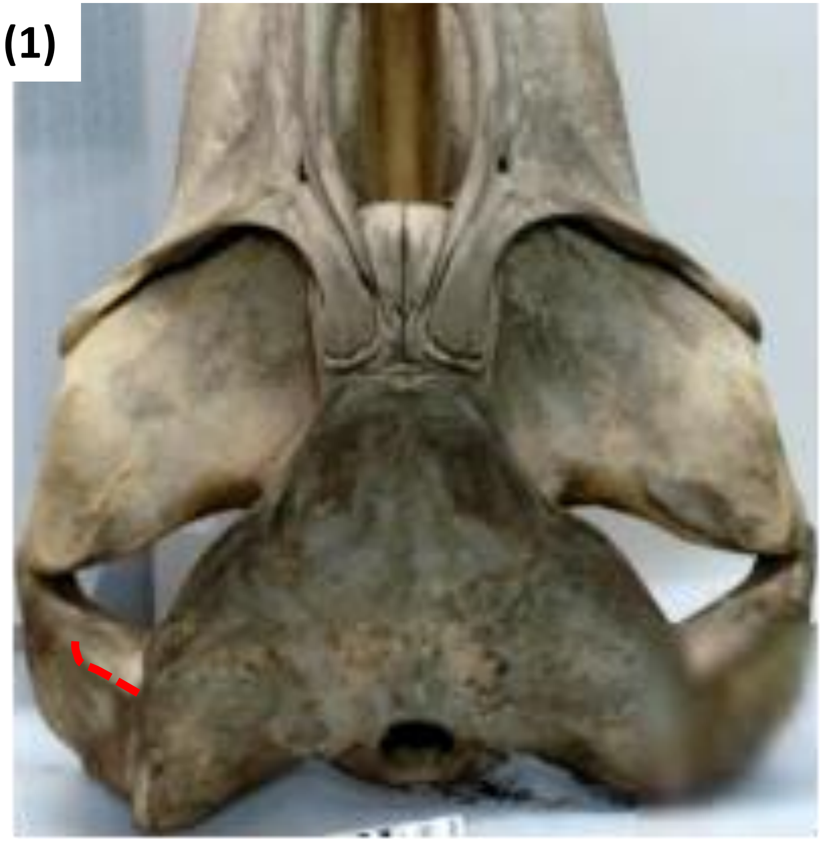

Balaenoptera acutorostrata

Copyright holder: Felix G. Marx/ The Charleston Museum, Charleston, South Carolina, USA

(2)

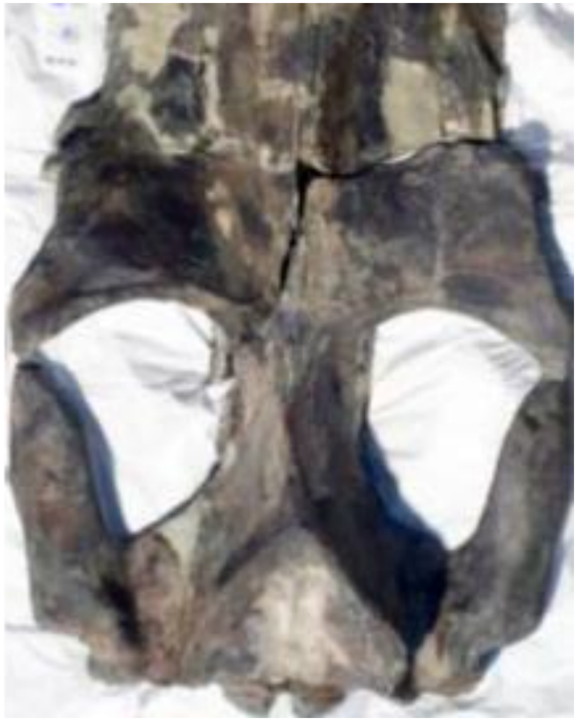

Yamatocetus canaliculatus

Copyright holder: Felix G. Marx/ Kitakyushu Museum of Natural and Human History, Kitakyushu, Kyushu, Japan

[102] 'Size of squamosal including zygomatic and postglenoid processes'

(0)'as long as high, or longer'

(1)'distinctly higher than long'

(0)

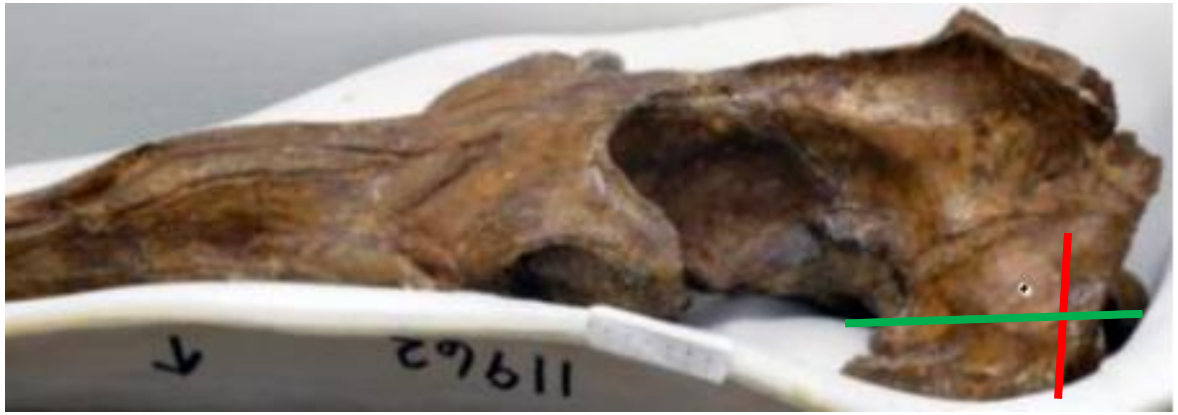

Zygorhiza kochii (mirrored)

Copyright holder: Felix G. Marx/ United States National Museum of Natural History, Washington DC, USA

(1)

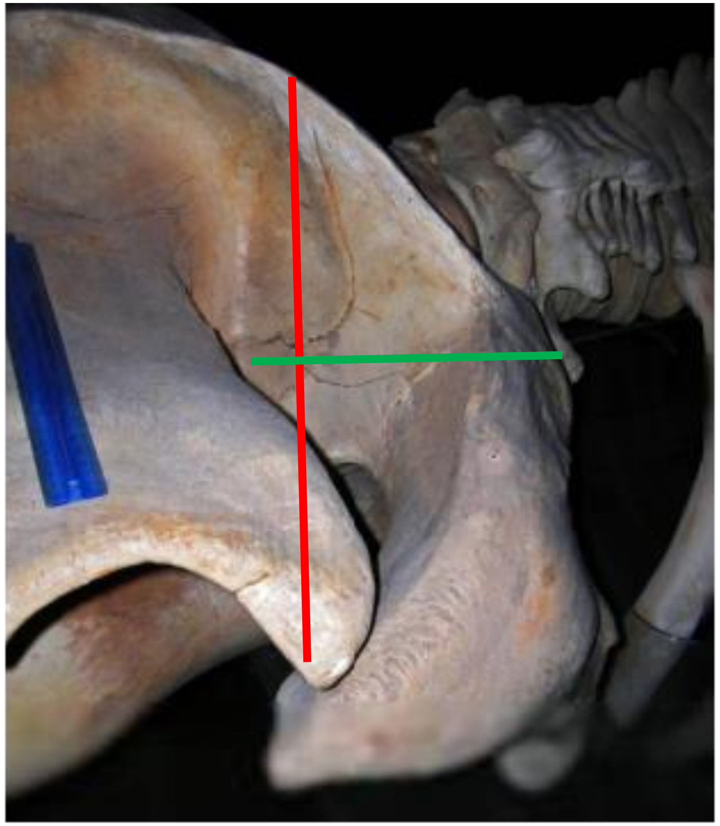

Megaptera novaeangliae

Copyright holder: Felix G. Marx/ Amgueddfa Cymru National Museum of Wales, Cardiff, Wales, United Kingdom

[103] 'Parieto-squamosal suture shaped like a crest or ridge'

(0) 'absent or low'

(1) 'present and distinctly elevated'

(0)

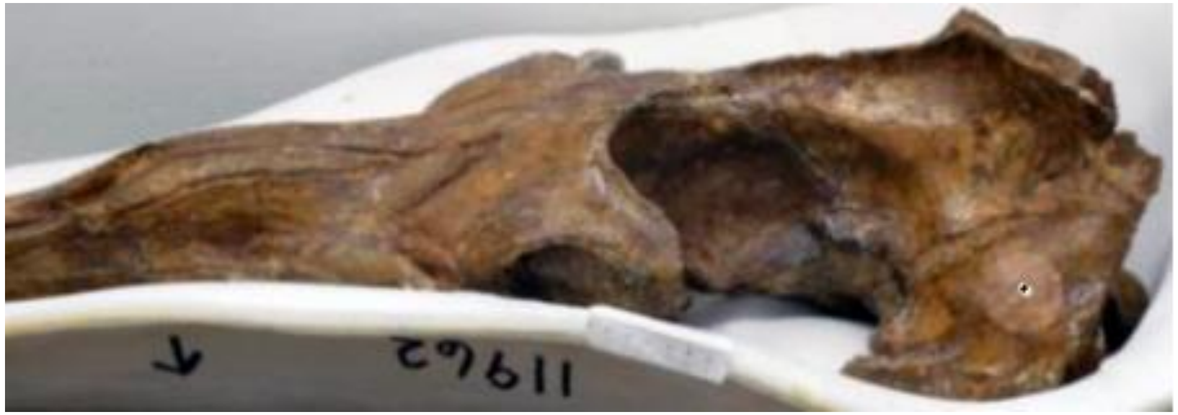

Zygorhiza kochii (mirrored)

Copyright holder: Felix G. Marx/ United States National Museum of Natural History, Washington DC, USA

(1)

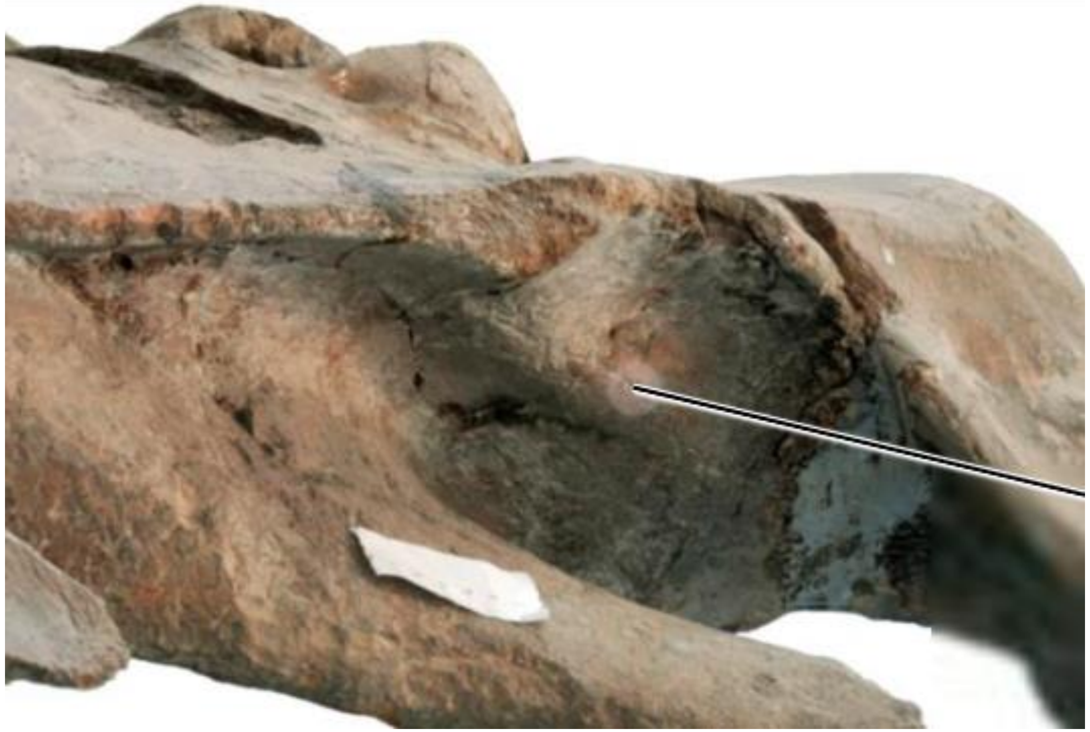

Balaena montalionis

Copyright holder: Felix G. Marx/ Museo di Storia Naturale dell'Università di Pisa, Italy

[104] 'Squamosal prominence'

(0) 'present as a projection on the crest delimiting the lateral or posterolateral edge of the squamosal fossa'

(1) 'absent'

(0)

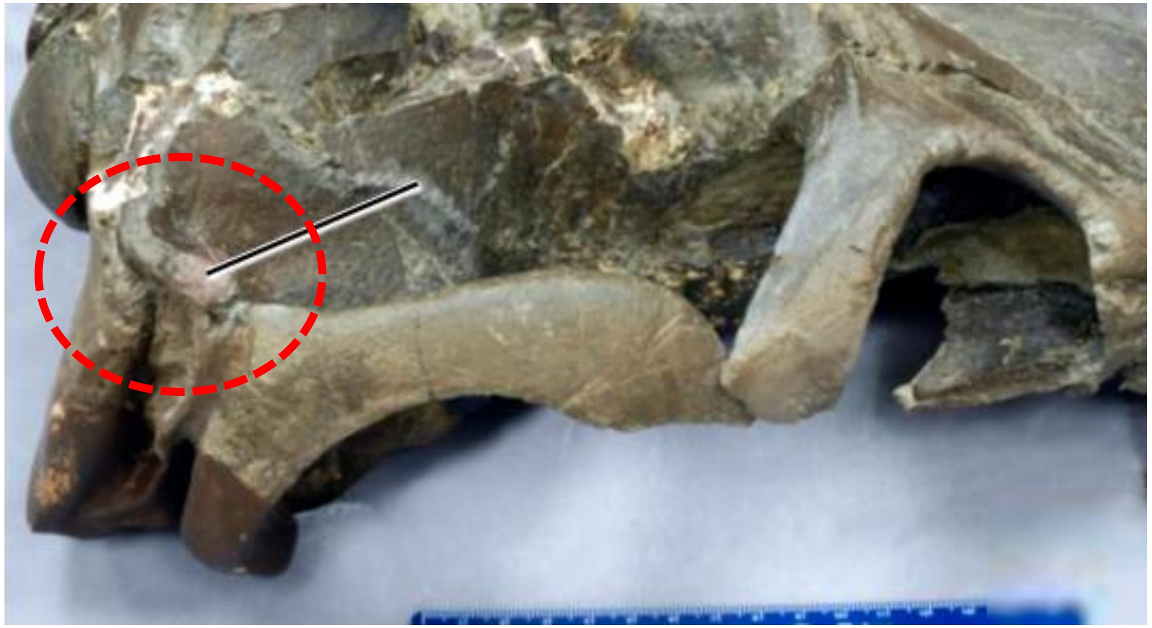

Aetiocetus weltoni

Copyright holder: Felix G. Marx/ University of California Museum of Paleontology, Berkeley, USA

(1)

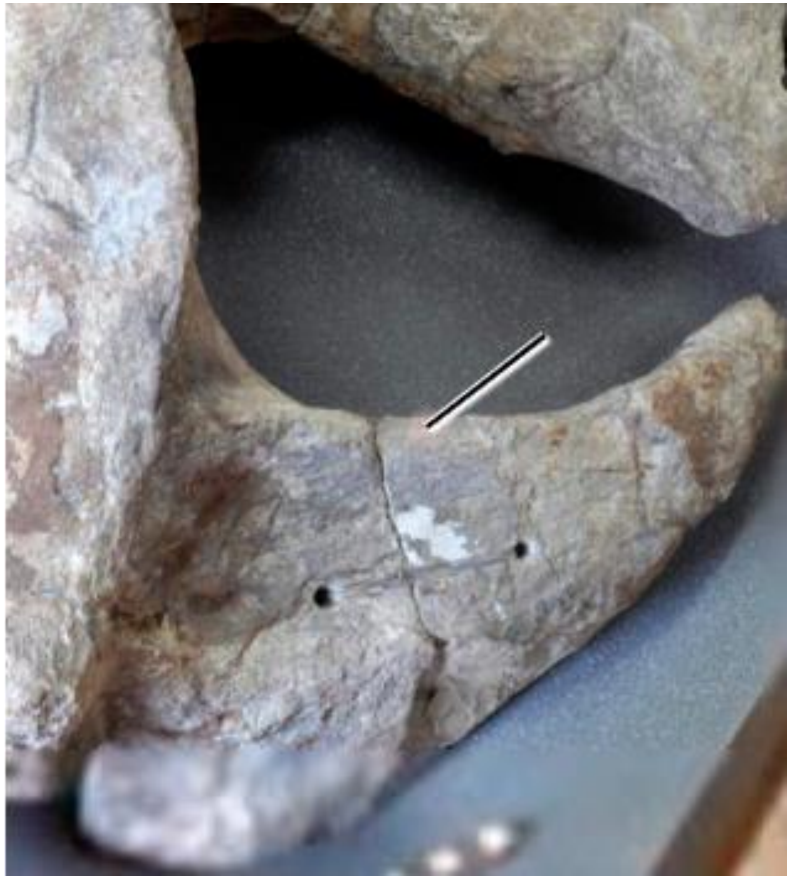

Balaenoptera portisi

Copyright holder: Felix G. Marx/ Museo Regionale di Scienze Naturali, Turin, Italy

[105] 'Transverse width of squamosal lateral to exoccipital'

(0)'width equal to or greater than 15% of the distance between the sagittal plane and the lateral edge of the exoccipital'

(1)'exposed portion of squamosal is less than 15% of that distance'

(0)

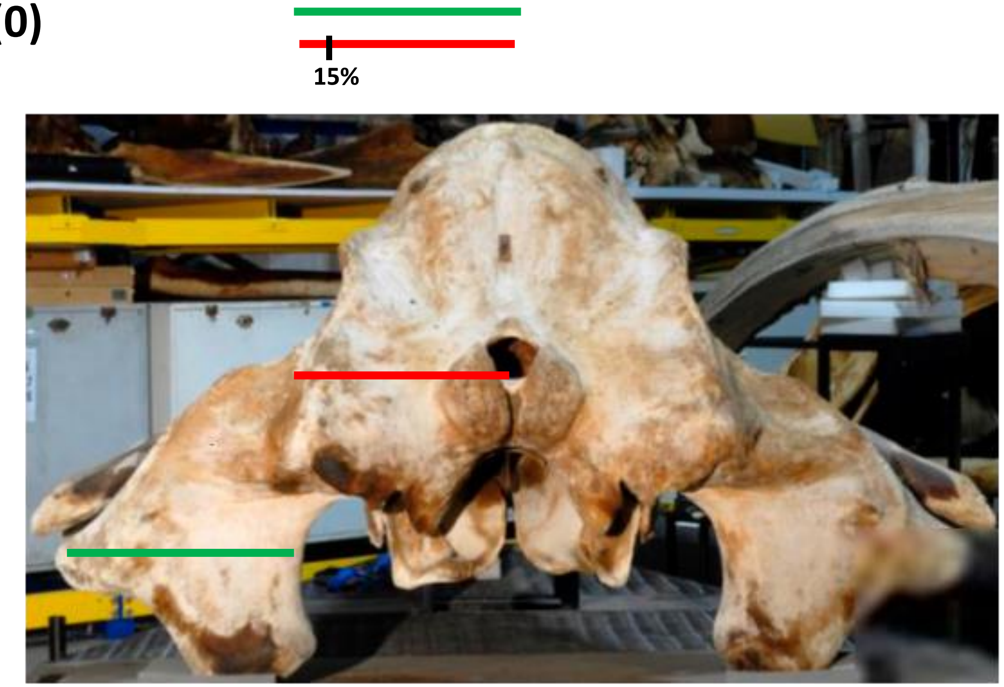

Eubalaena spp.

Copyright holder: Felix G. Marx/ United States National Museum of Natural History, Washington DC, USA

(1)

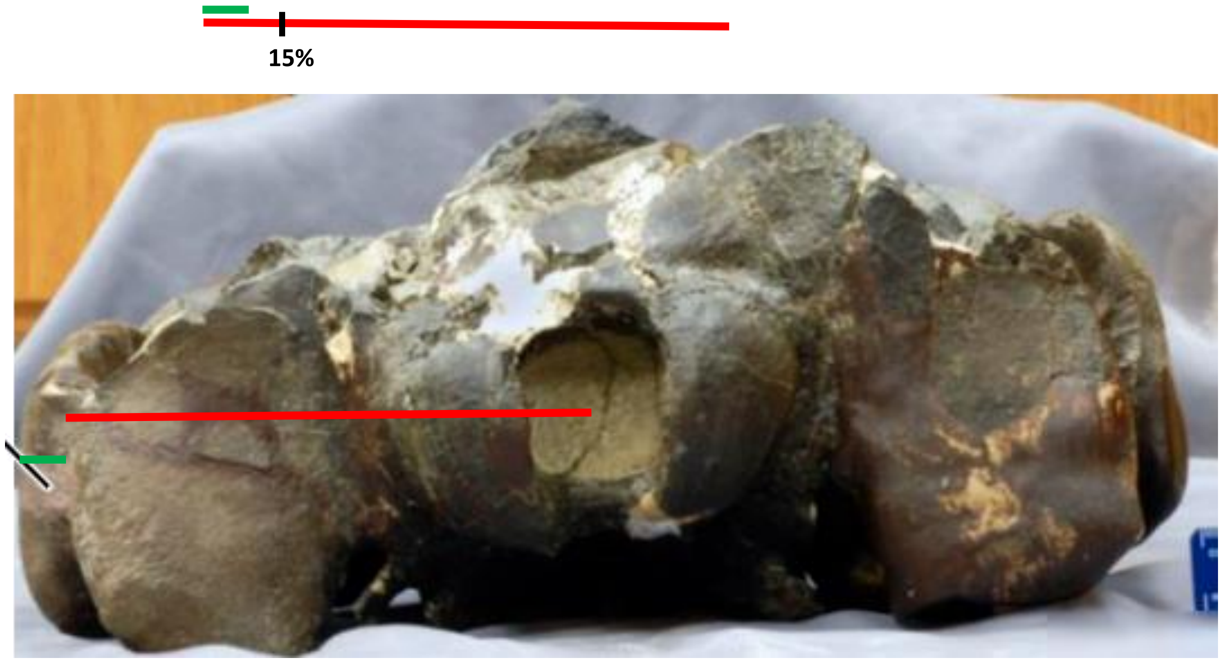

Aetiocetus weltoni

Copyright holder: Felix G. Marx/ University of California Museum of Paleontology, Berkeley, USA

[106] 'Length of squamosal fossa relative to maximum transverse width of temporal fossa as measured in a straight line from the posteriormost point of the temporal fossa to the posteriormost point of the nuchal crest'

(0)'length of squamosal fossa is three quarters the width of the temporal fossa or longer'

(1)'length of squamosal fossa is less than three quarters the width of the temporal fossa'

(0)

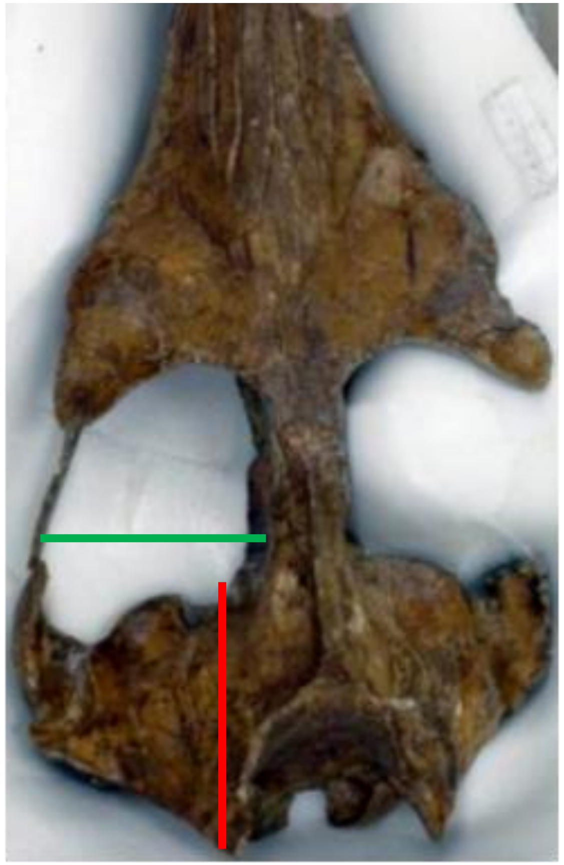

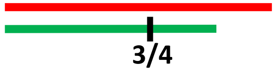

Zygorhiza kochii

Copyright holder: Felix G. Marx/ United States National Museum of Natural History, Washington DC, USA

(1)

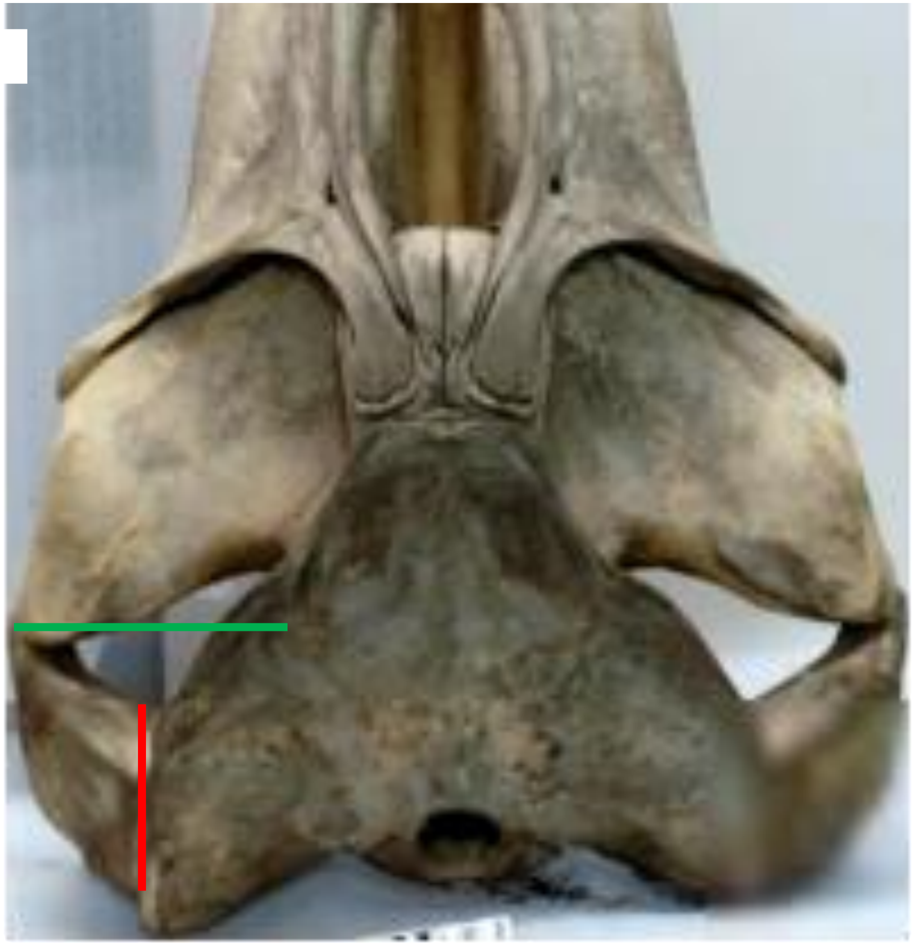

Balaenoptera acutorostrata

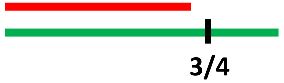

Copyright holder: Felix G. Marx/ The Charleston Museum, Charleston, South Carolina, USA

[107] 'Squamosal cleft'

(0) 'absent'

(1) 'present'

(0)

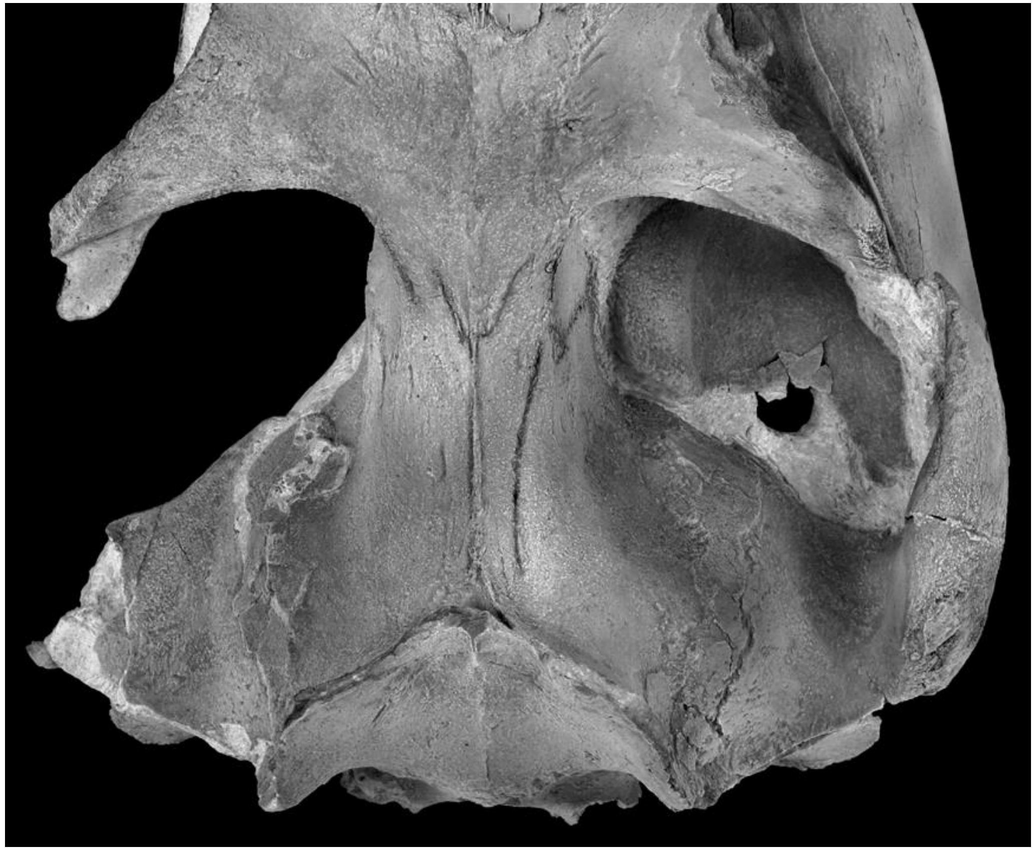

Janjucetus hunderi

Copyright holder: Erich M. G. Fitzgerald/ Museums Victoria, Melbourne, Australia

(1)

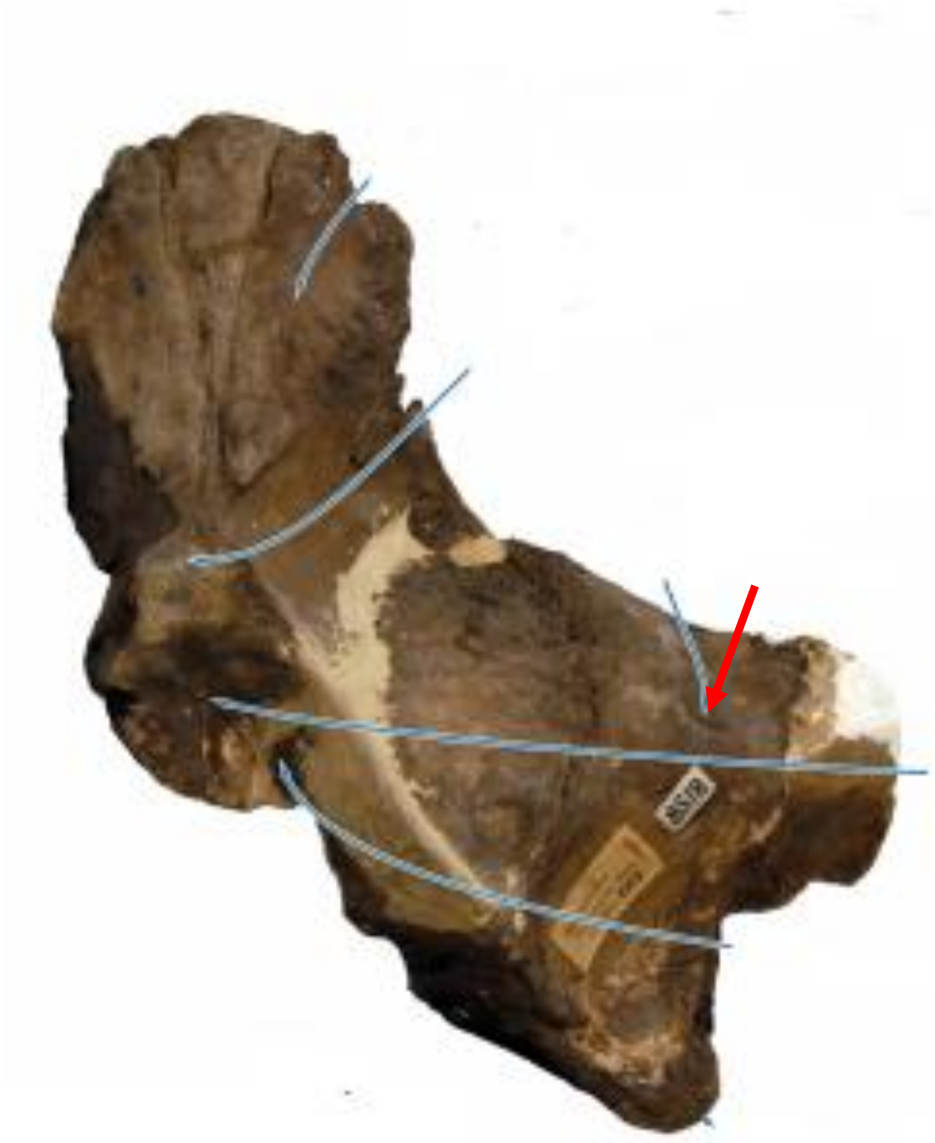

Metopocetus durinasus

Adapted from: "A new species of *Metopocetus* (Cetacea, Mysticeti, Cetotheriidae) from the Late Miocene of the Netherlands." Marx, Bosselaers, and Louwye, 2016. *PeerJ* 4: e1572.

[108] 'Squamosal crease'

(0) 'absent'

(1) 'present'

(0)

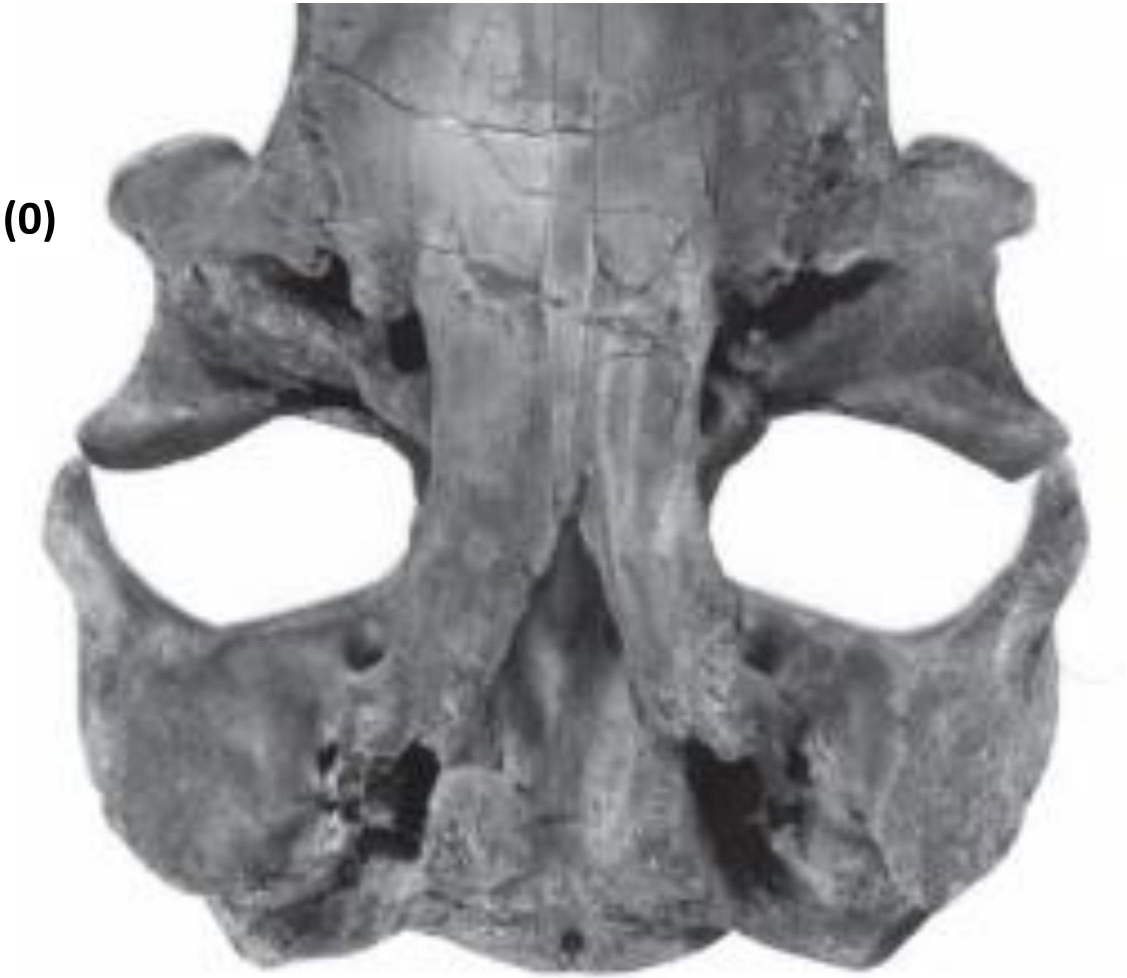

Piscobalaena nana

(1)

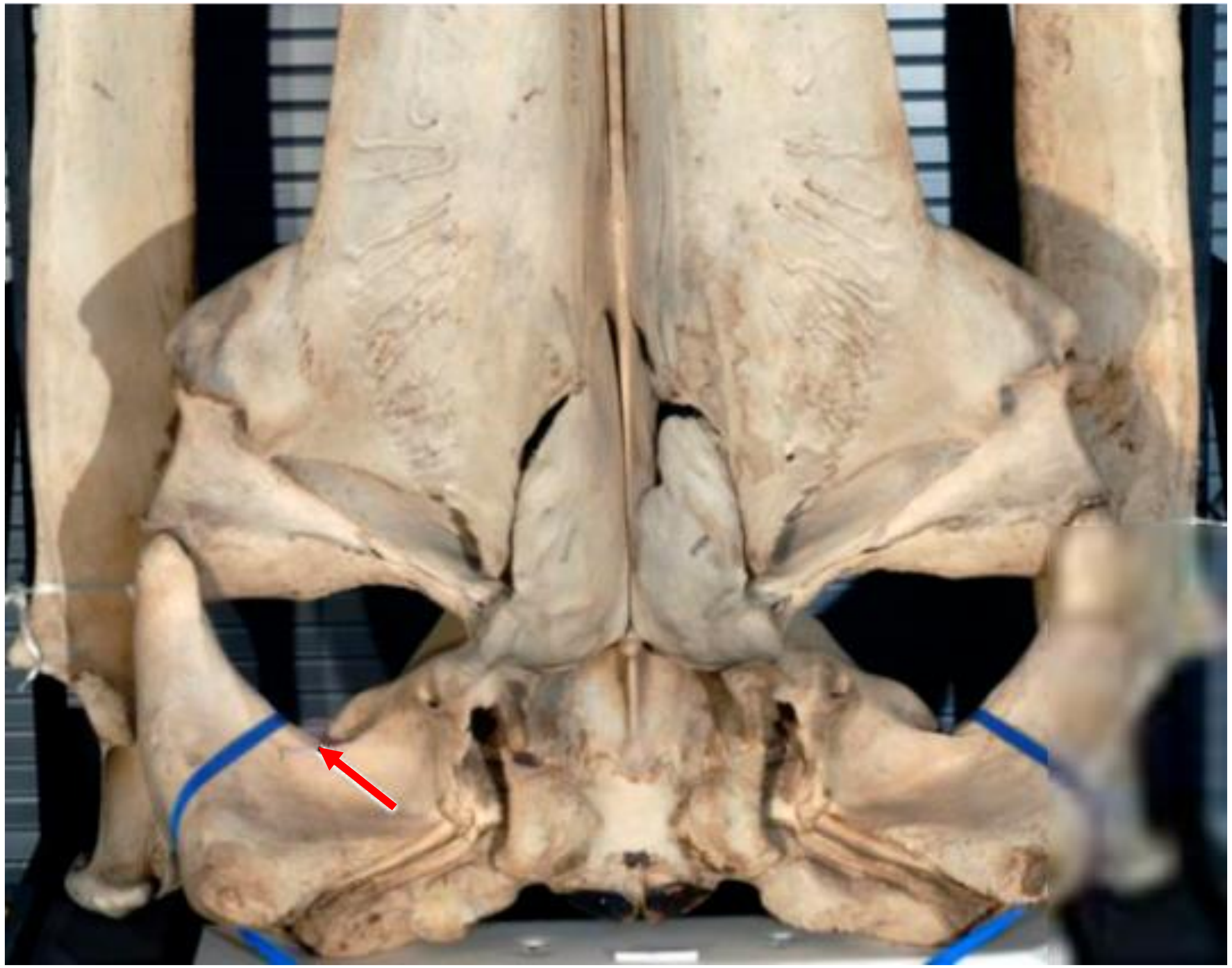

Balaenoptera borealis

Adapted from:"The anatomy and relationships of *Piscobalaena nana* (Cetacea, Mysticeti), a Cetotheriidae s.s. from the early Pliocene of Peru", Bouetel and Muizon, 2006. *Geodiversitas* 28.2 (2006): 319-395.

Copyright holder: Felix G. Marx/ United States National Museum of Natural History, Washington DC, USA

[109] 'Paired tubercles on supraoccipital'

(0) 'absent'

(1) 'limited to low ridges forming the lateral edges of a medial fossa'

(2) 'present'

(0)

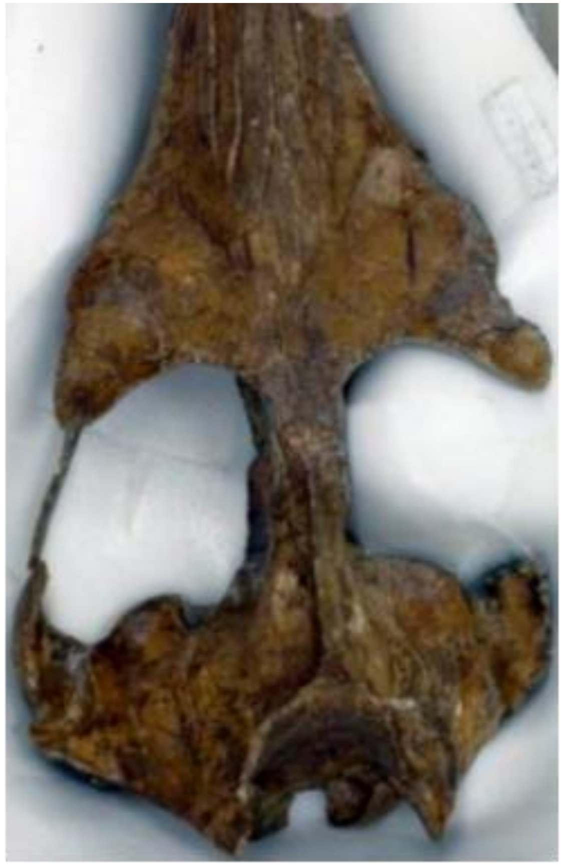

Zygorhiza kochii

Copyright holder: Felix G. Marx/ United States National Museum of Natural History, Washington DC, USA

(1)

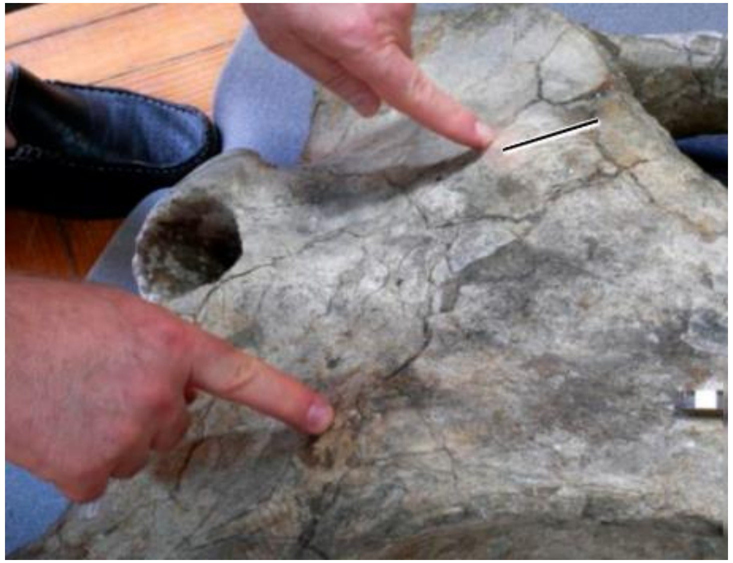

Balaenoptera portisi

Copyright holder: Felix G. Marx/ Museo Regionale di Scienze Naturali, Turin, Italy

(2)

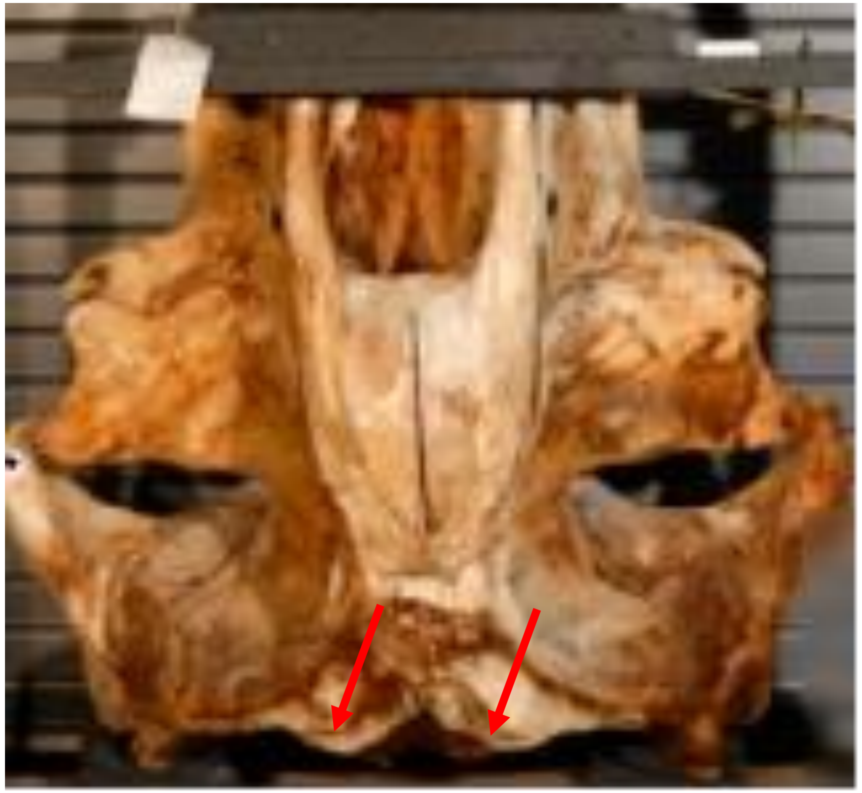

Eschrichtius robustus

Felix G. Marx/ Unites States National Museum of Natural History, Washington DC, USA

[110] 'Lateral edge of supraoccipital in dorsal view'

(0) 'convex'

(1) 'straight'

(2) 'concave'

(3) 'sigmoidal'

(0)

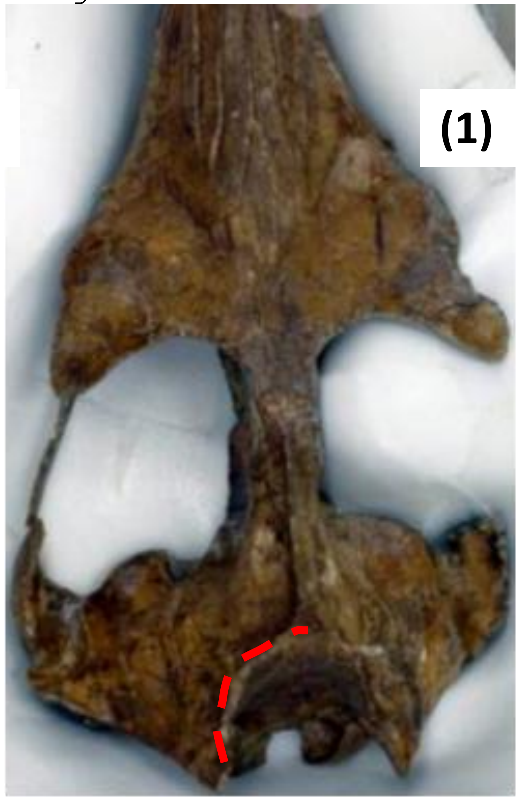

Zygorhiza kochii

Copyright holder: Felix G. Marx/ United States National Museum of Natural History, Washington DC, USA

(1)

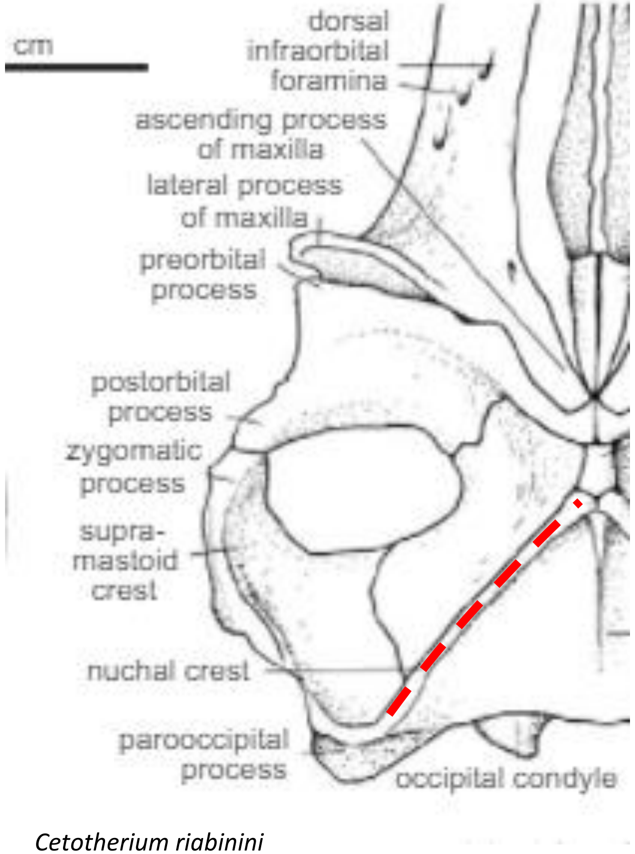

Cetotherium riabinini

Adapted from: "The anatomy of the Late Miocene baleen whale Cetotherium riabinini from Ukraine." Gol'din et al., 2013. *Acta Palaeontologica Polonica* 59.4: 795-814.

(2)

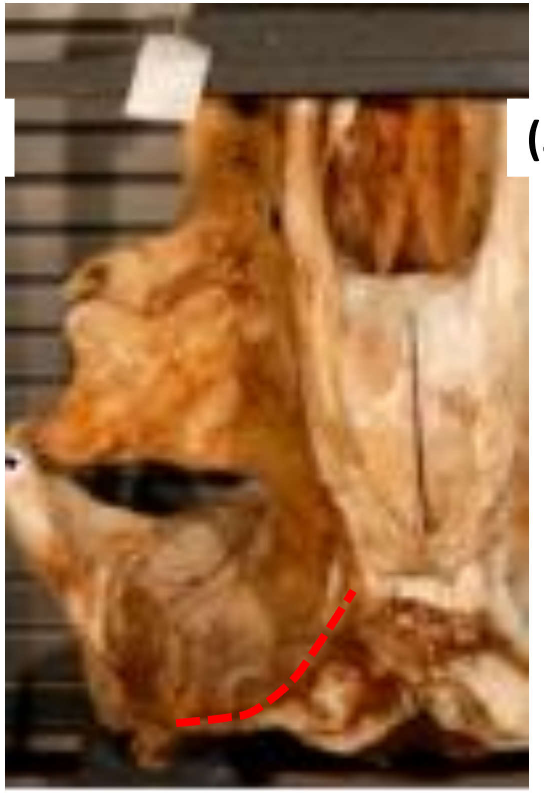

Eschrichtius robustus

Felix G. Marx/ Unites States National Museum of Natural History, Washington DC, USA

(3)

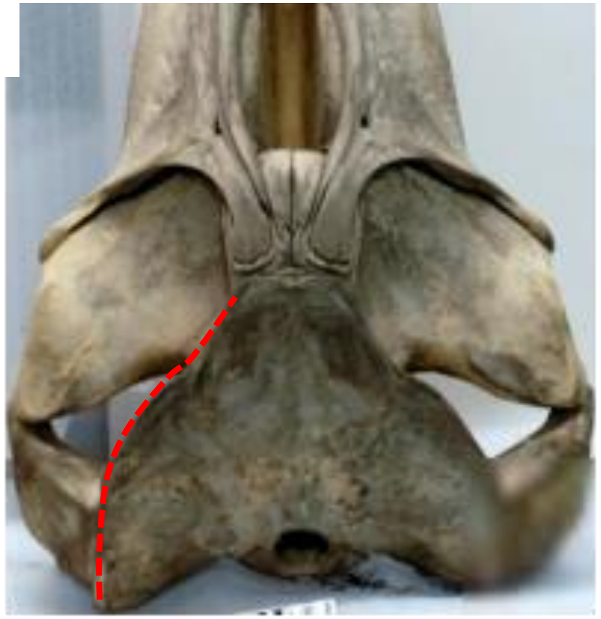

Balaenoptera acutorostrata

Copyright holder: Felix G. Marx/ The Charleston Museum, Charleston, South Carolina, USA

[111] 'Anterior border of supraoccipital shield'

(0) 'rounded or pointed'

(1) 'squared'

(0)

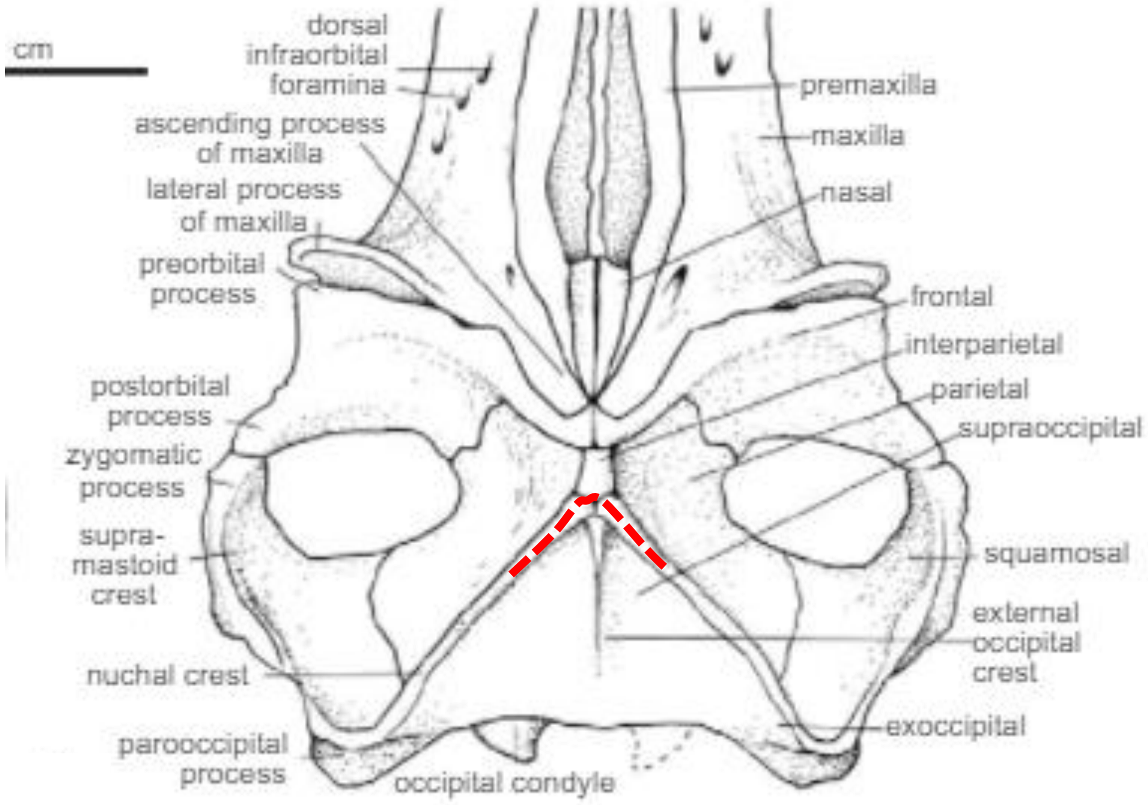

Cetotherium riabinini

Adapted from: "The anatomy of the Late Miocene baleen whale Cetotherium riabinini from Ukraine." Gol'din et al., 2013. *Acta Palaeontologica Polonica* 59.4: 795-814.

(1)

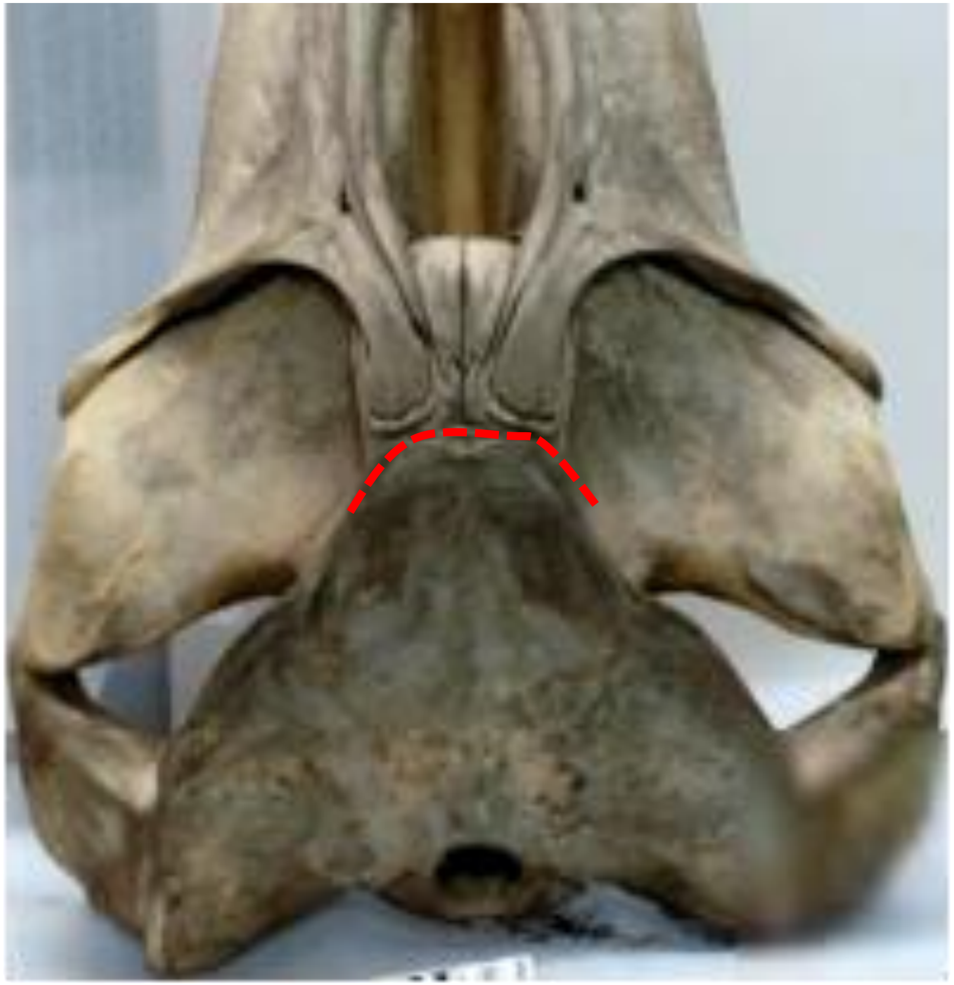

Balaenoptera acutorostrata

Copyright holder: Felix G. Marx/ The Charleston Museum, Charleston, South Carolina, USA

[112] 'Overall outline of supraoccipital in dorsal view'

(0) 'rounded'

(1) 'triangular'

(0)

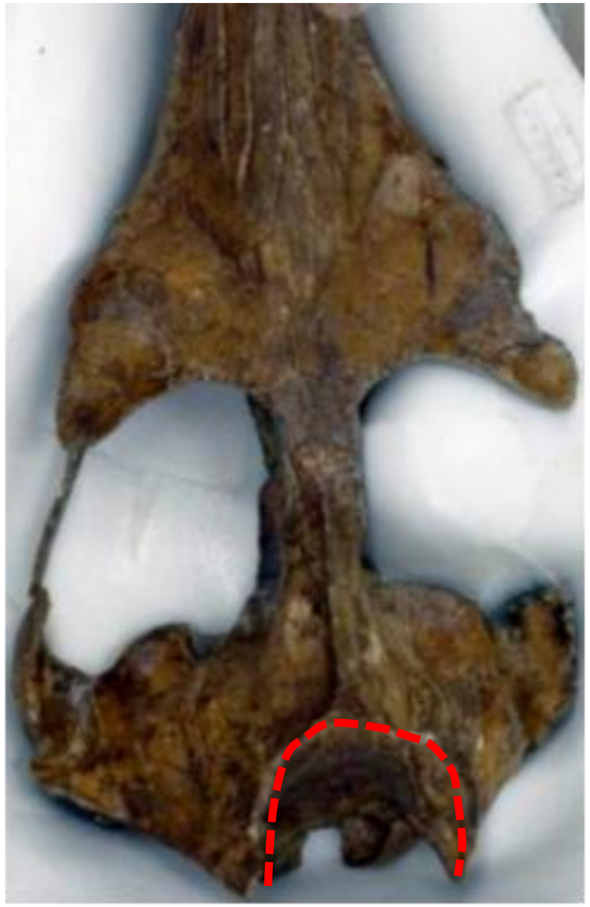

Zygorhiza kochii

Copyright holder: Felix G. Marx/ United States National Museum of Natural History, Washington DC, USA

(1)

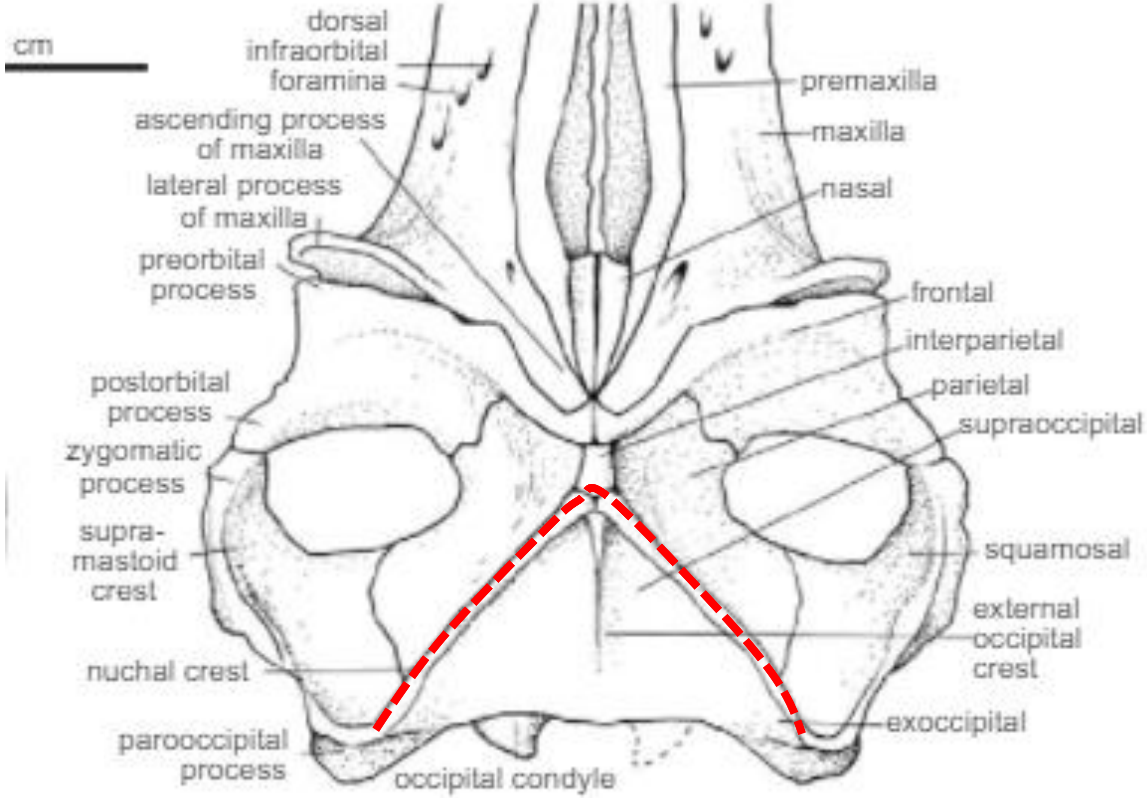

Cetotherium riabinini

Adapted from: "The anatomy of the Late Miocene baleen whale Cetotherium riabinini from Ukraine." Gol'din et al., 2013. *Acta Palaeontologica Polonica* 59.4: 795-814.

[113] 'Anterior half of dorsal surface of supraoccipital'

(0)'concave'

(1)'flat or convex'

(0)

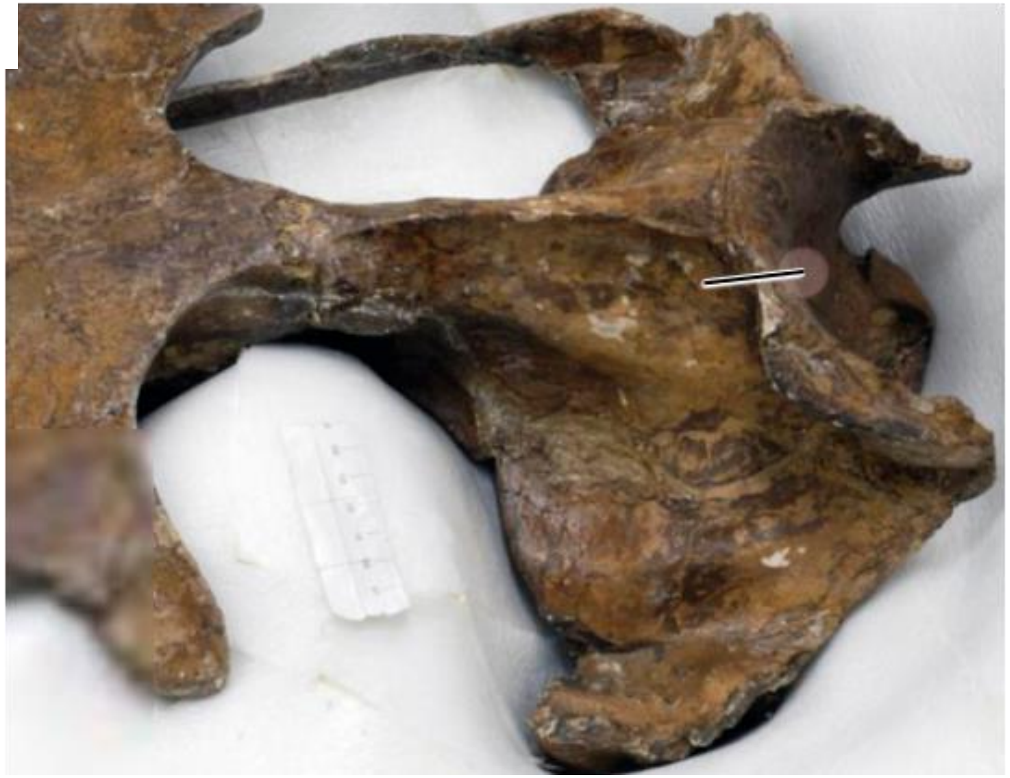

Zygorhiza kochii (mirrored)

Copyright holder: Felix G. Marx/ United States National Museum of Natural History, Washington DC, USA

(1)

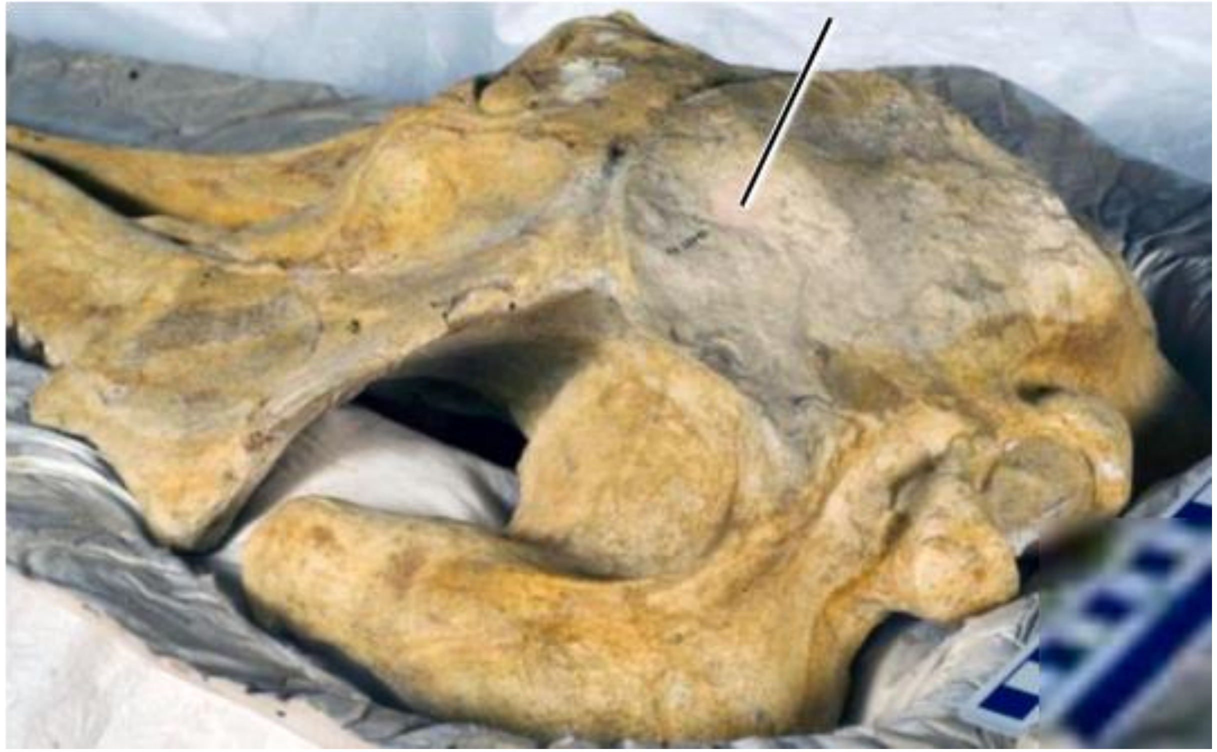

Waipatia maerewhenua

Copyright holder: Felix G. Marx/ University of Otago Museum of Geology, Dunedin, New Zealand

[114] 'External occipital crest'

(0) 'absent or faint'

(1) 'restricted to anterior half of supraoccipital shield'

(2) 'running all the way along the supraoccipital shield'

(0)

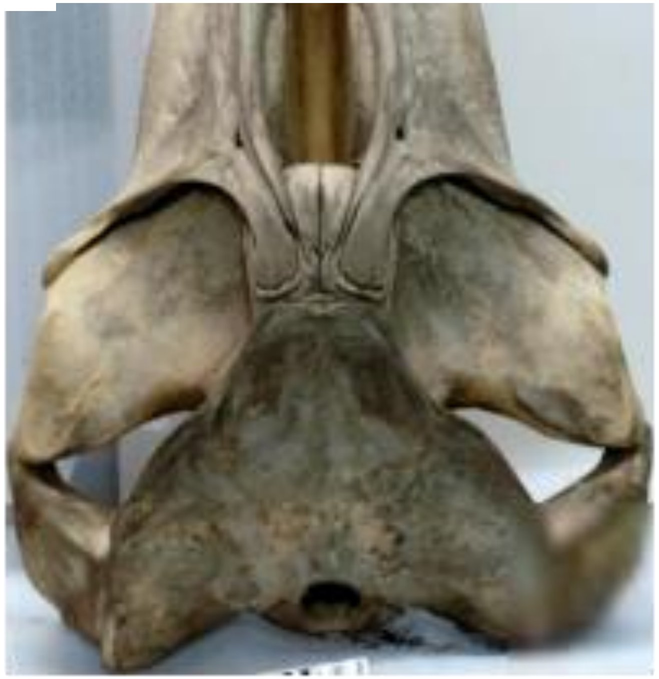

Balaenoptera acutorostrata

Copyright holder: Felix G. Marx/ The Charleston Museum, Charleston, South Carolina, USA

(1)

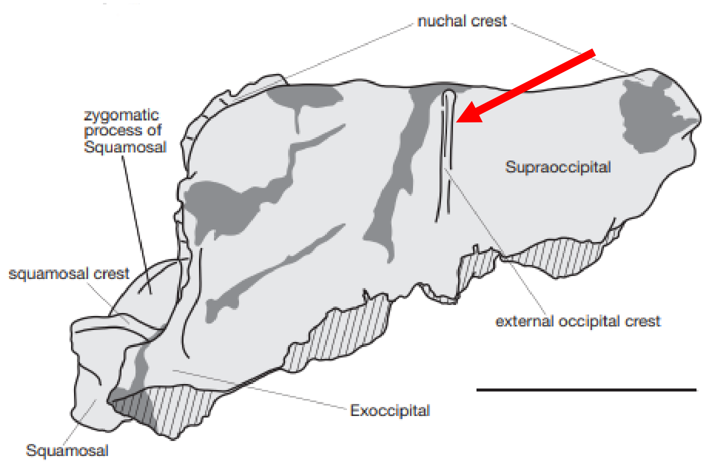

Mystacodon selenensis

Adapted from: *"Mystacodon selenensis, the earliest known toothed mysticete (Cetacea, Mammalia) from the late Eocene of Peru: anatomy, phylogeny, and feeding adaptations." De Muizon et al., 2019. Geodiversitas 41.1: 401-499.*

(2)

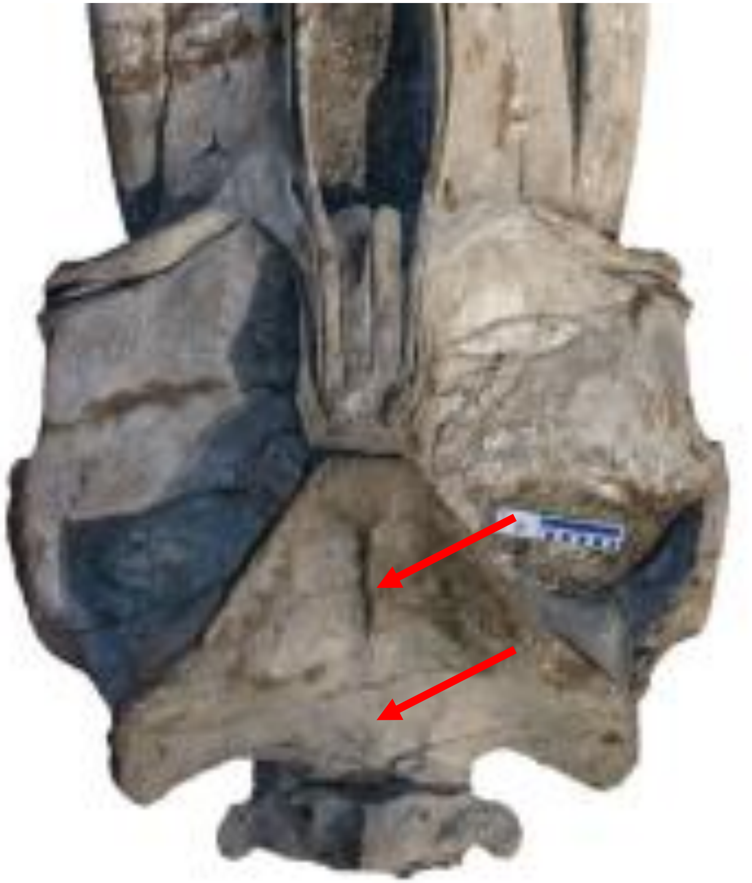

Incakujira anillodefuego

Adapted from: "A new Miocene baleen whale from the Peruvian desert." Marx and Kohno, 2016. *Royal Society Open Science* 3.10 (2016): 160542.

[115] 'Tip of postglenoid process in lateral view'

(0) 'curving anteriorly'

(1) 'pointing ventrally'

(2) 'pointing posteriorly'

(0)

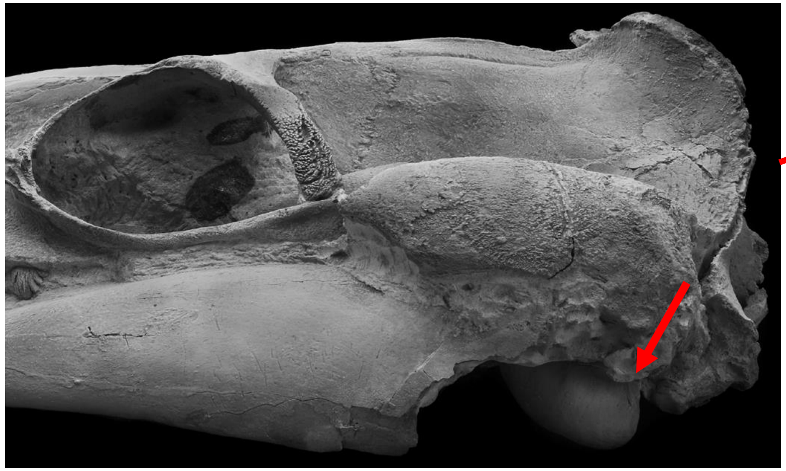

Janjucetus hunderi

Copyright holder: Erich M. G. Fitzgerald/ Museums Victoria, Melbourne, Australia

(1)

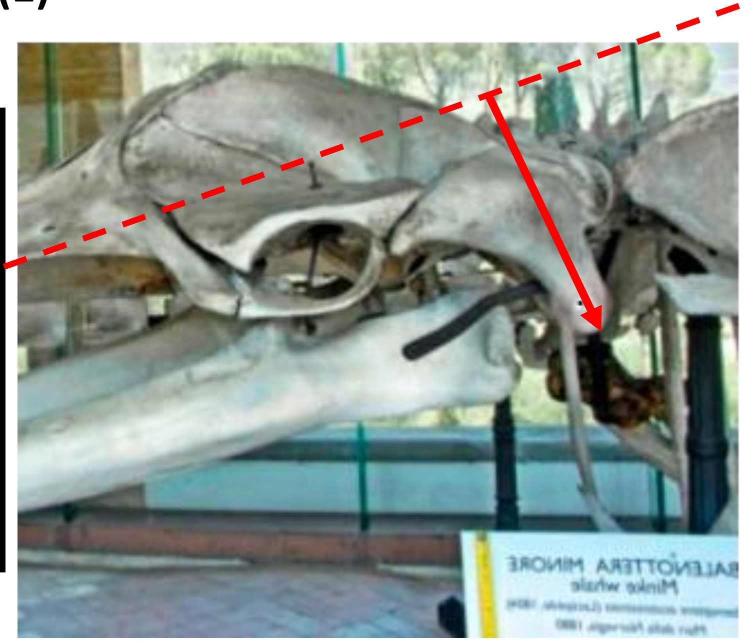

Balaenoptera acurostrata

Copyright holder: Felix G. Marx/ Museo di Storia Naturale e del Territorio, Università di Pisa, Calci, Italy

(2)

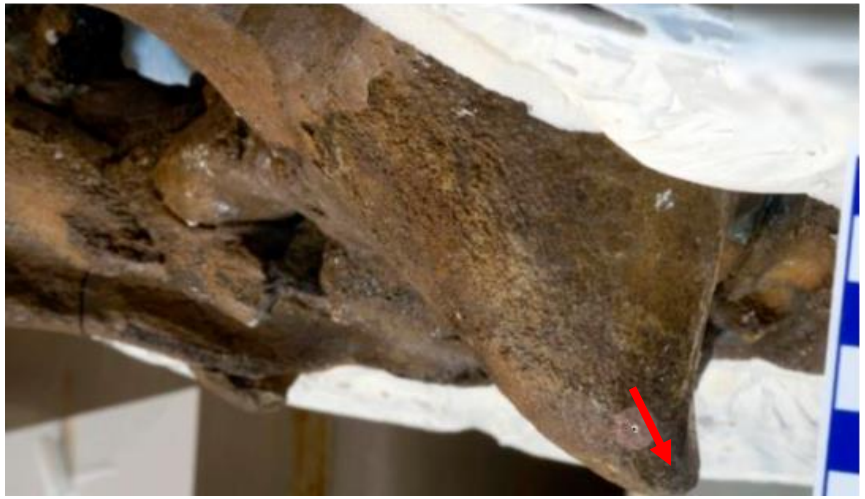

Diorocetus hiatus

Copyright holder: Felix G. Marx/United States National Museum of Natural History, Washington DC, USA

[116] 'Ventral edge of postglenoid process in lateral view'

(0) 'approximately in line with or dorsal to ventral edge of exoccipital'

(1) 'extending well ventral to ventral edge of exoccipital'

(0)

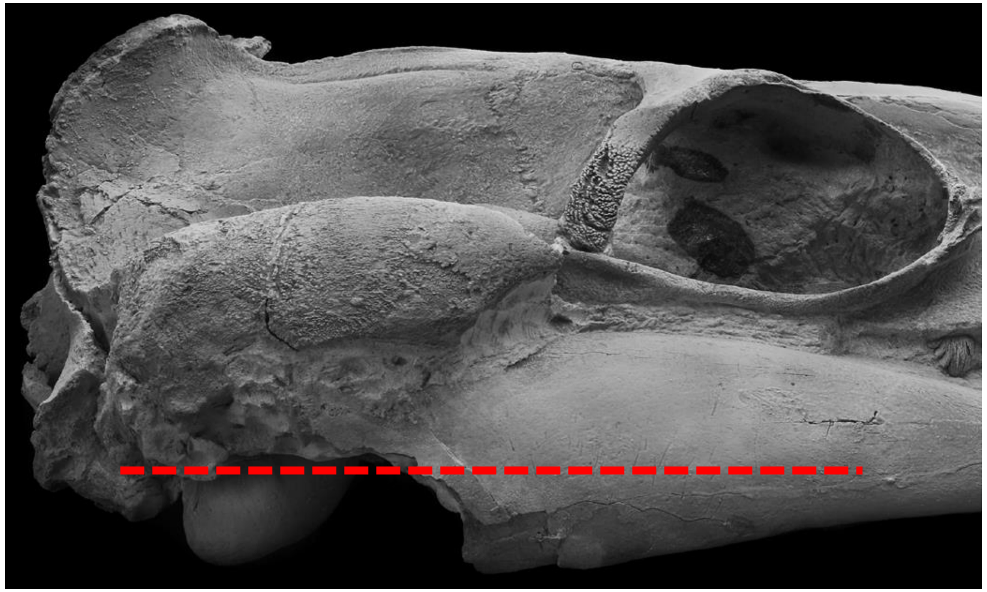

Janjucetus hunderi

Copyright holder: Erich M. G. Fitzgerald/ Museums Victoria, Melbourne, Australia

(1)

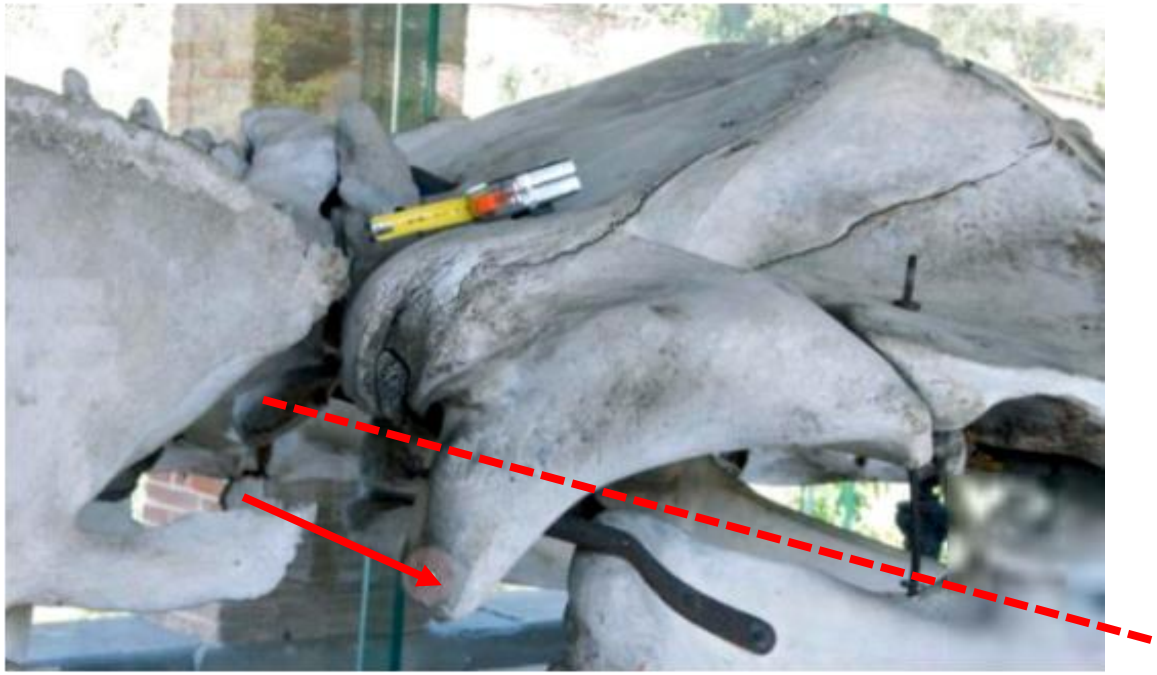

Balaenoptera acurostrata

Copyright holder: Felix G. Marx/ Museo di Storia Naturale e del Territorio, Università di Pisa, Calci, Italy

[117] 'Orientation of postglenoid process in posterior view'

(0) 'ventrolateral'

(1) 'ventral'

(2) 'ventromedial'

(0)

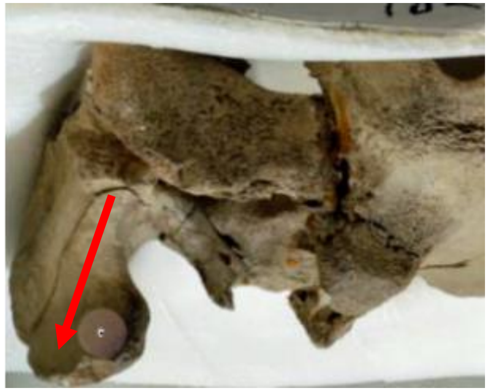

Herpetocetus transatlanticus

Copyright holder: Felix G. Marx/ United States National Museum of Natural History, Washington DC, USA

(1)

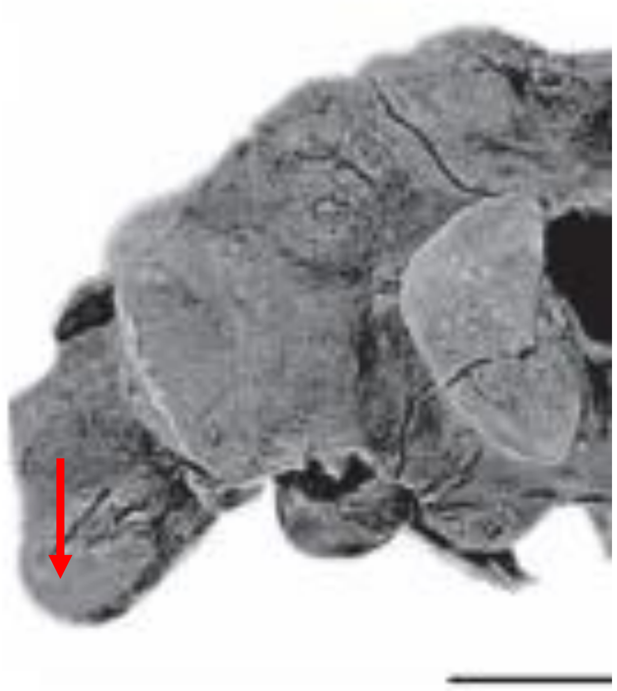

Piscobalaena nana

Adapted from: "The anatomy and relationships of *Piscobalaena nana* (Cetacea, Mysticeti), a Cetotheriidae s.s. from the early Pliocene of Peru", Bouetel and Muizon, 2006. *Geodiversitas* 28.2 (2006): 319-395.

(2)

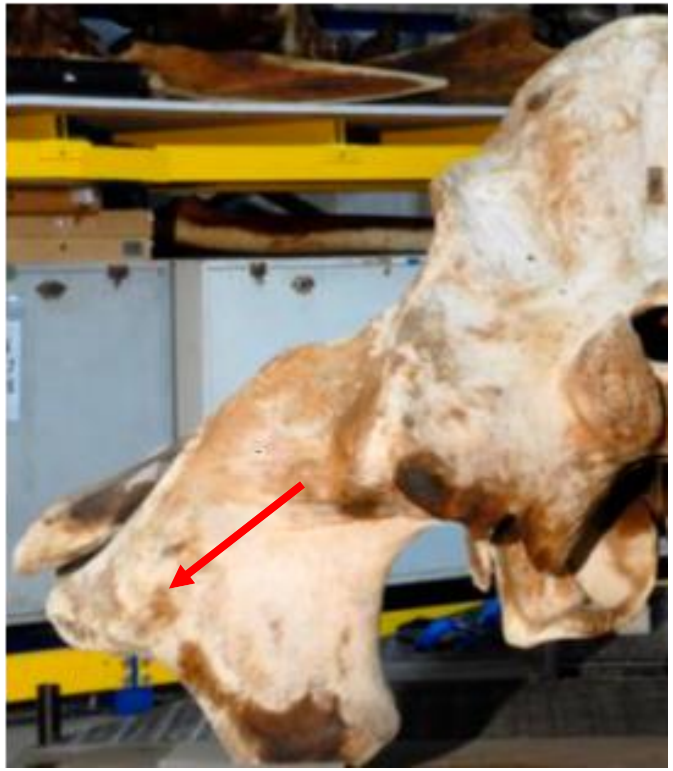

Eubalaena spp.

Copyright holder: Felix G. Marx/ United States National Museum of Natural History, Washington DC, USA

[118] 'Outline of postglenoid process in anterior or posterior view'

(0) 'parabolic'

(1) 'as state 0 but with lateral and medial edges parallel or concave'

(2) 'as state 1 but distinctly wider transversely than high dorsoventrally'

(3) 'triangular'

(4) 'trapezoidal with a ventrally directed medial border'

(0)

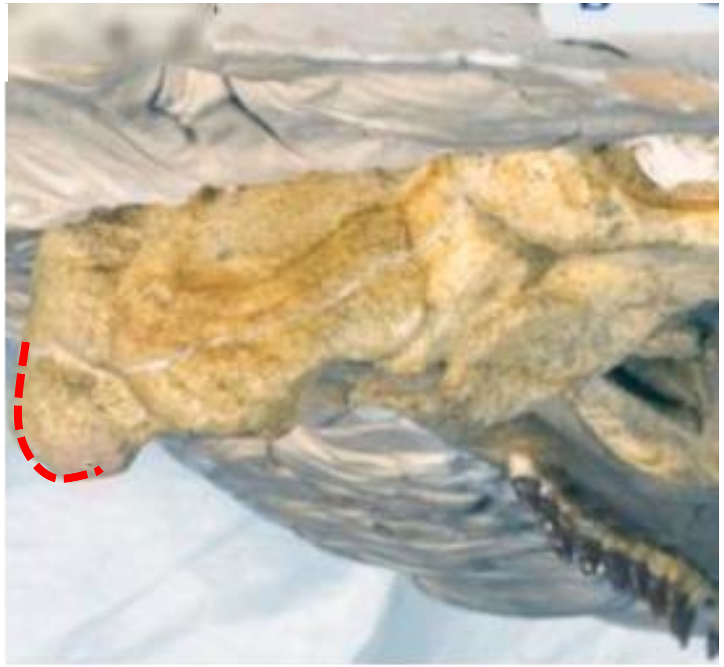

Waipatia maerewhenua

Copyright holder: Felix G. Marx/ University of Otago Museum of Geology, Dunedin, New Zealand

(1)

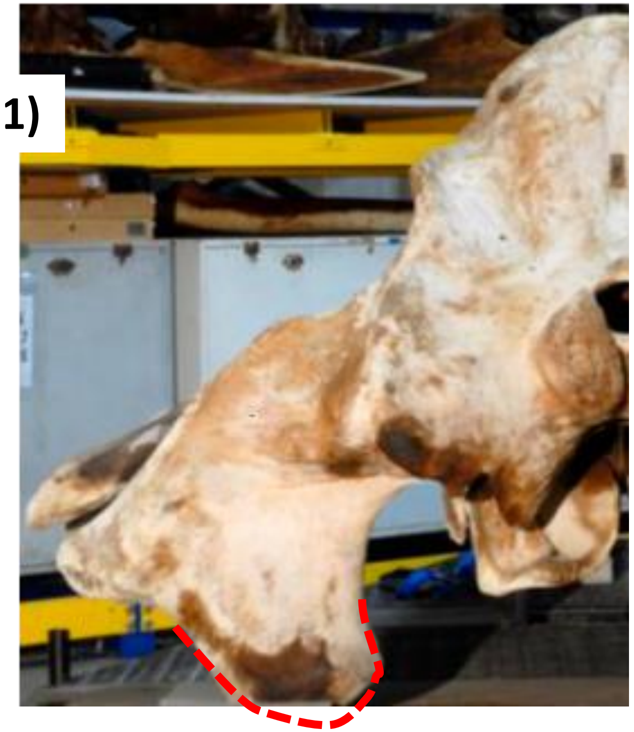

Eubalaena spp.

Copyright holder: Felix G. Marx/ United States National Museum of Natural History, Washington DC, USA

(2)

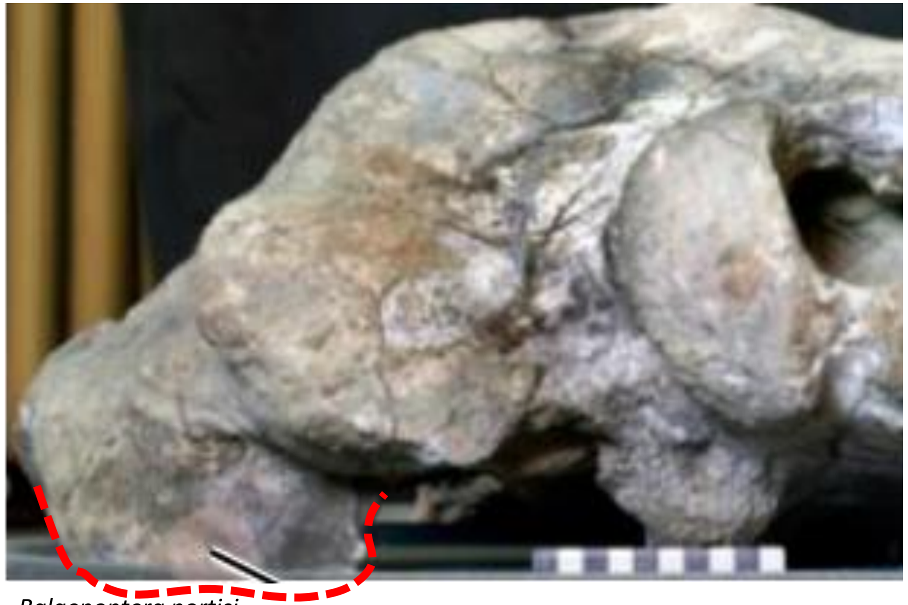

Balaenoptera portisi

Copyright holder: Felix G. Marx/ Museo Regionale di Scienze Naturali, Turin, Italy

[118] 'Outline of postglenoid process in anterior or posterior view'

(0)'parabolic'

(1)'as state 0 but with lateral and medial edges parallel or concave'

(2)'as state 1 but distinctly wider transversely than high dorsoventrally'

(3)'triangular'

(4)'trapezoidal with a ventrally directed medial border'

(3)

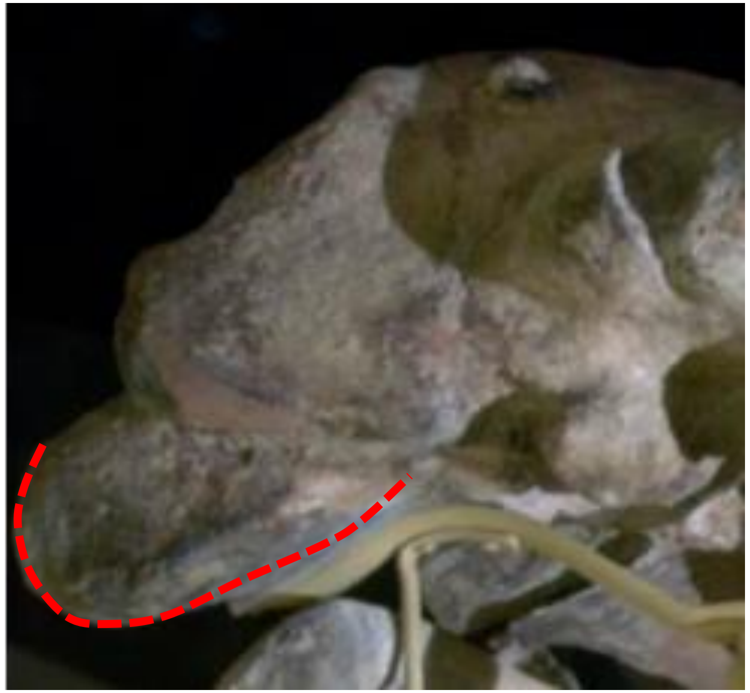

Aglaocetus moreni

Copyright holder: Felix G. Marx/ The Field Museum of Natural History, Chicago, USA

(4)

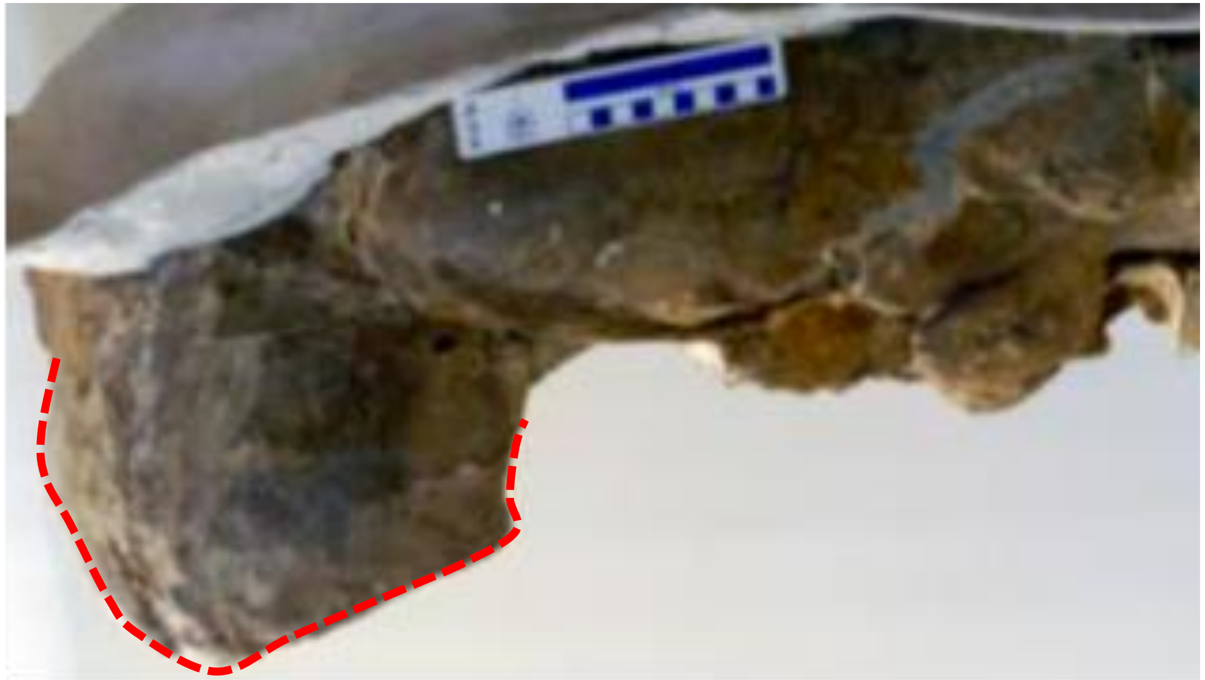

Megaptera miocaena

Copyright holder: Felix G. Marx/United States National Museum of Natural History, Washington DC, USA

[119] 'Twisting of postglenoid process in ventral view'

(0)'absent'

(1)'twisted clockwise on the left side and anticlockwise on the right side so that the glenoid cavity faces anteromedially'

(0)

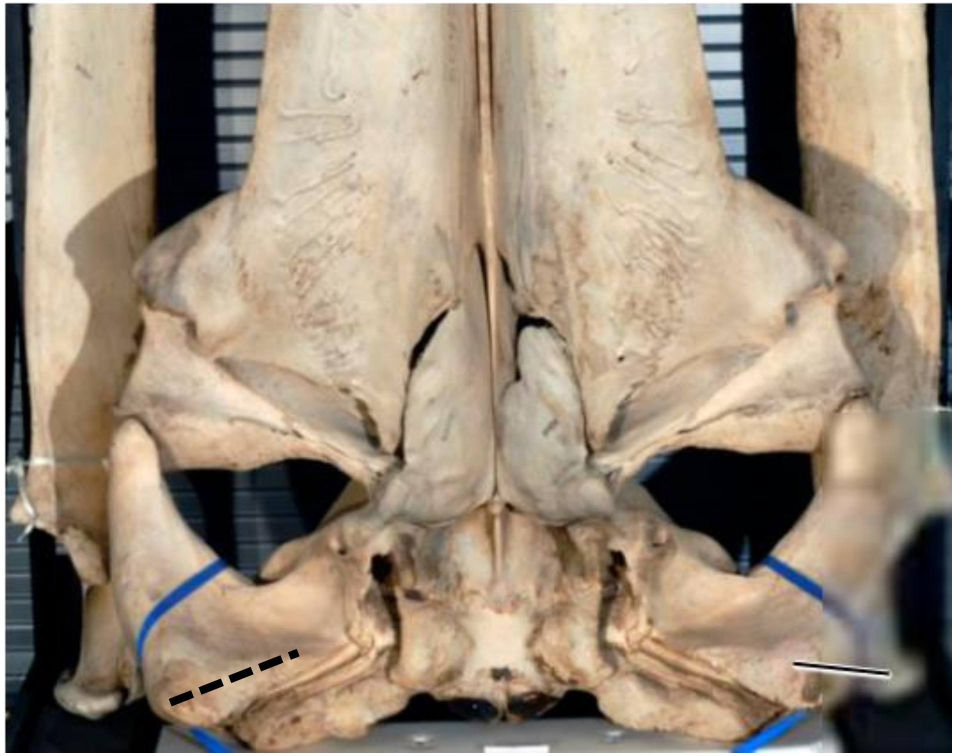

Balaenoptera borealis

Copyright holder: Felix G. Marx/ United States National Museum of Natural History, Washington DC, USA

(1)

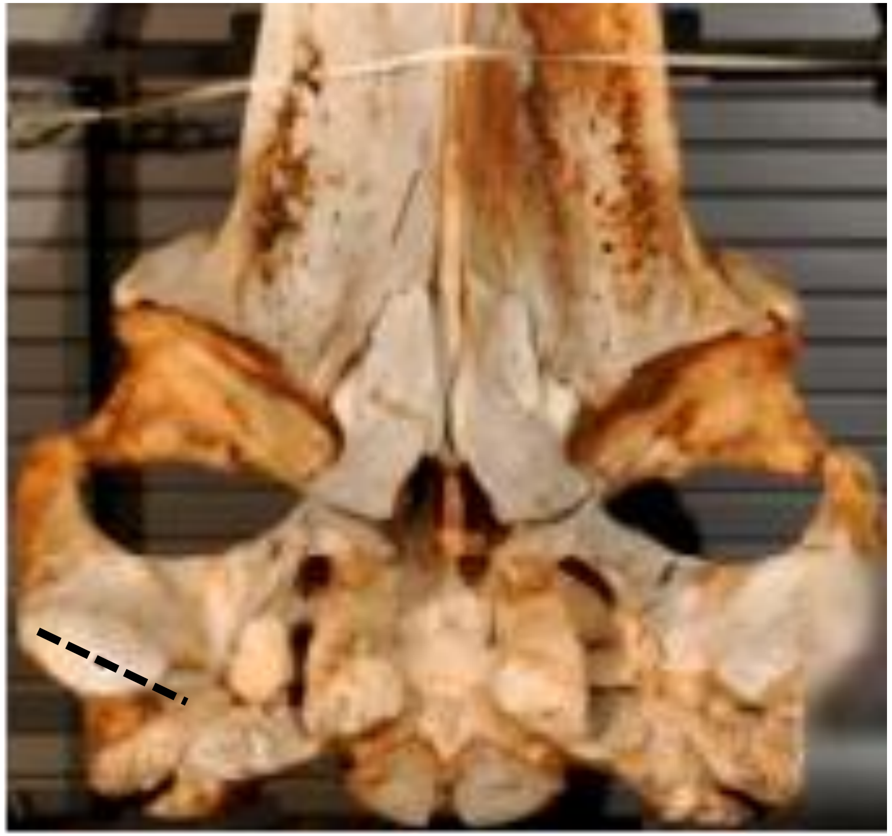

Eschrichtius robustus

Copyright holder: Felix G. Marx/ Unites States National Museum of Natural History, Washington DC, USA

[120] 'Position of base of postglenoid process in ventral or posterior view'

(0)'in line with the lateral edge of the skull'

(1)'shifted away medially from the lateral edge of the skull'

(0)

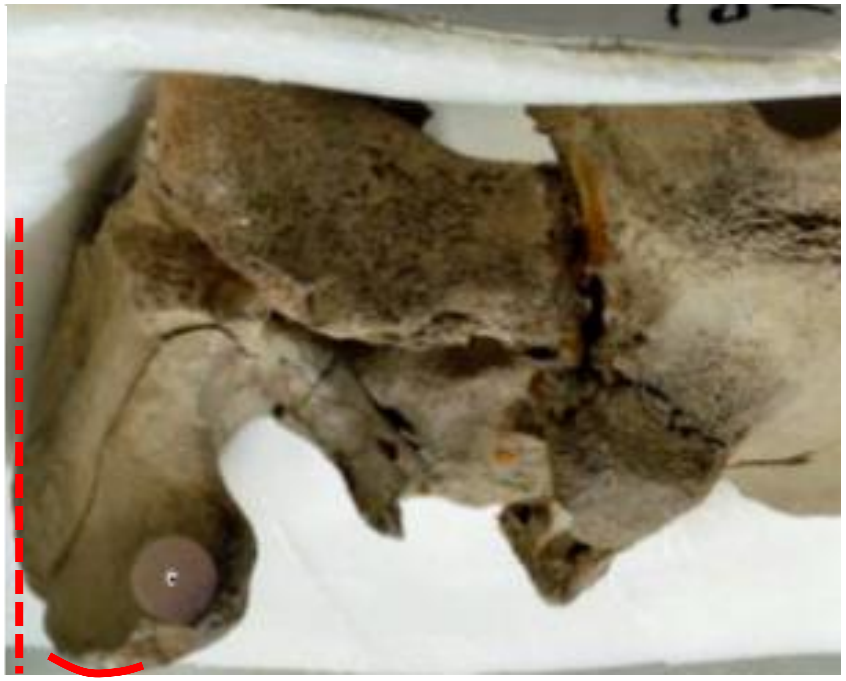

Herpetocetus transatlanticus

Copyright holder: Felix G. Marx/ United States National Museum of Natural History, Washington DC, USA

(1)

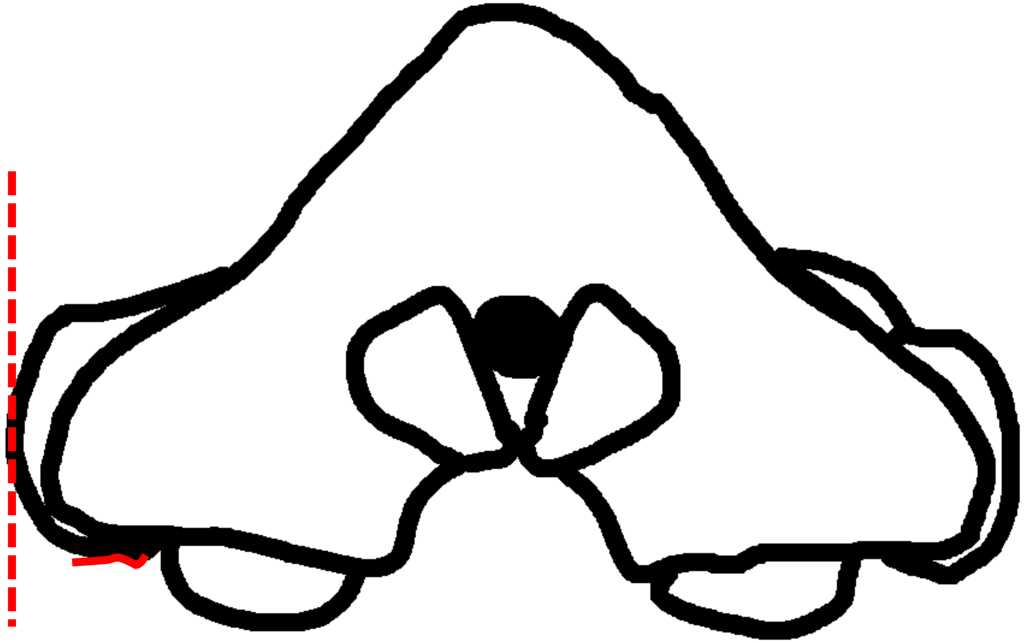

Example based on *Caperea marginata*

[121] 'Medial border of postglenoid process in ventral view'

(0)'confluent with more medial portion of squamosal'

(1)'offset from remainder of squamosal by a distinct ridge'

(0)

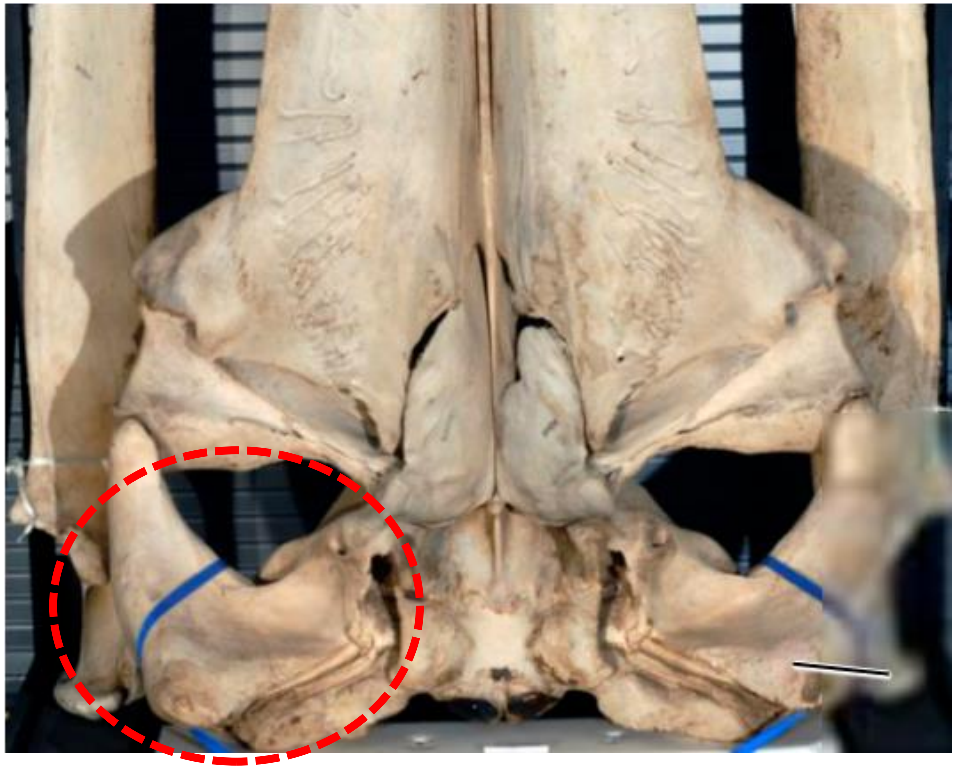

Balaenoptera borealis

Copyright holder: Felix G. Marx/ United States National Museum of Natural History, Washington DC, USA

(1)

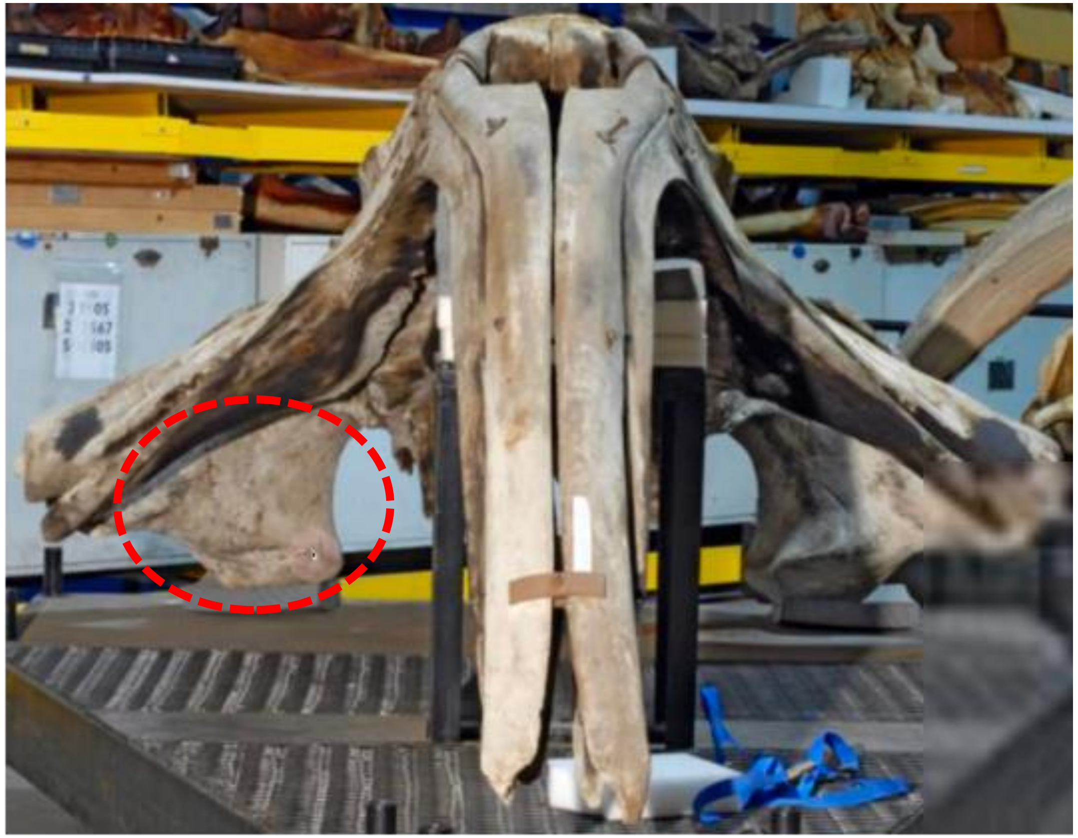

Eubalaena spp.

Copyright holder: Felix G. Marx/ United States National Museum of Natural History, Washington DC, USA

[122] 'Choanal margin of palatine in ventral view'

(0) 'absent'

(1) 'straight or convex'

(2) 'concave'

(3) 'forms a longitudinal notch'

(0)

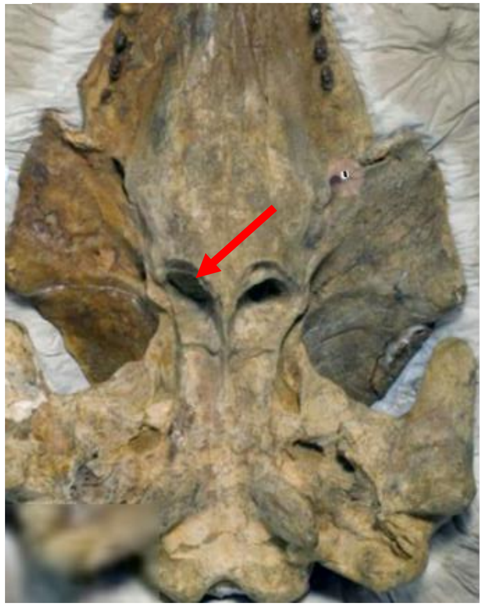

Waipatia maerewhenua

Copyright holder: Felix G. Marx/ University of Otago Museum of Geology, Dunedin, New Zealand

(1)

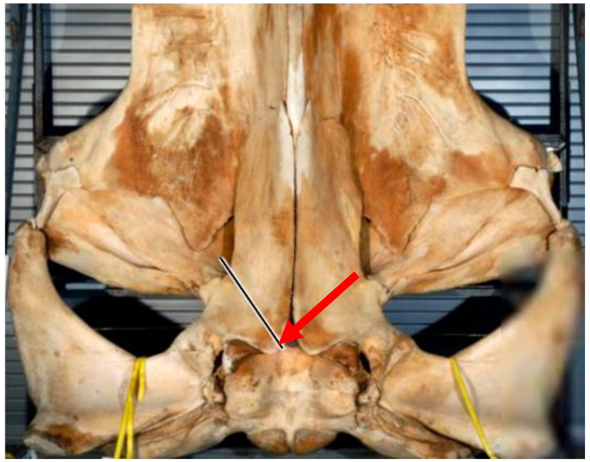

Balaenoptera musculus

Copyright holder: Felix G. Marx/ United States National Museum of Natural History, Washington DC, USA

(2)

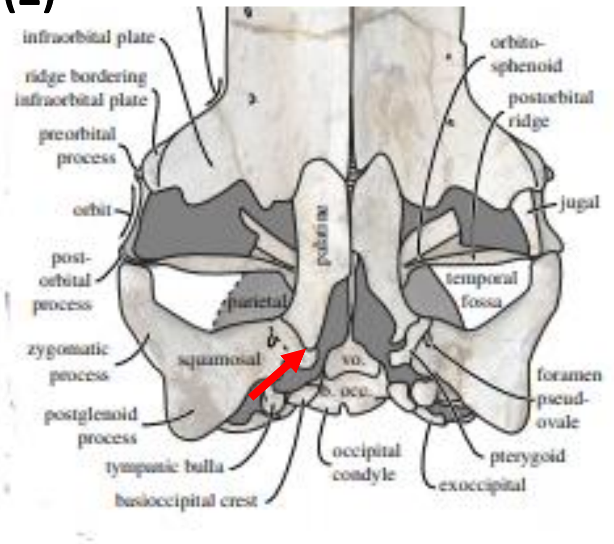

Incakujira anillodefuego

Adapted from: "A new Miocene baleen whale from the Peruvian desert." Marx and Kohno, 2016. *Royal Society Open Science* 3.10 (2016): 160542.

(3)

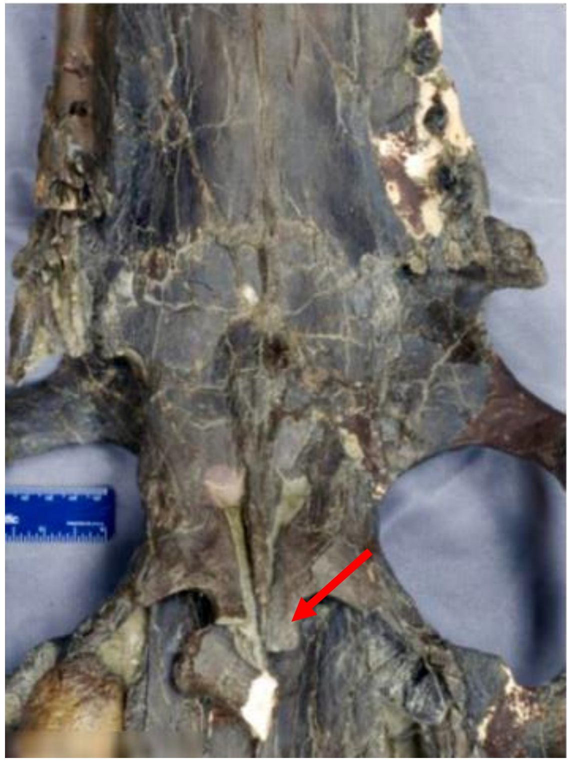

Aetiocetus weltoni

Copyright holder: Felix G. Marx/ University of California Museum of Paleontology, Berkeley, USA

[123] 'Pterygoid in ventral view'

(0)'partially or entirely exposed'

(1)'palatine almost completely covers pterygoid and extends on to the hamular process'

(0)

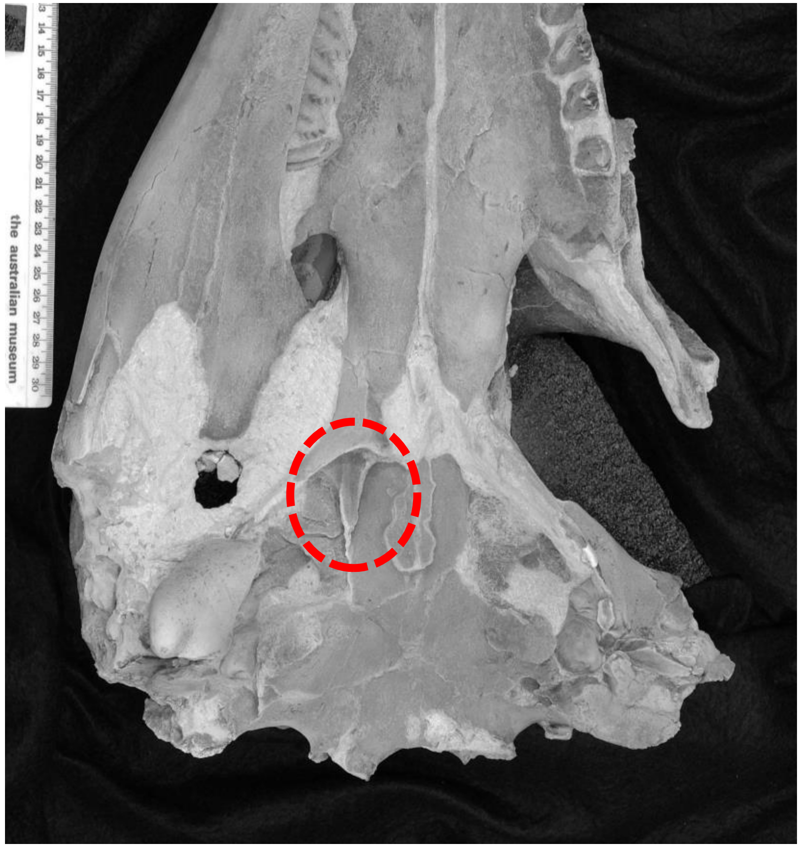

Janjucetus hunderi

Copyright holder: Erich M. G. Fitzgerald/ Museums Victoria, Melbourne, Australia

(1)

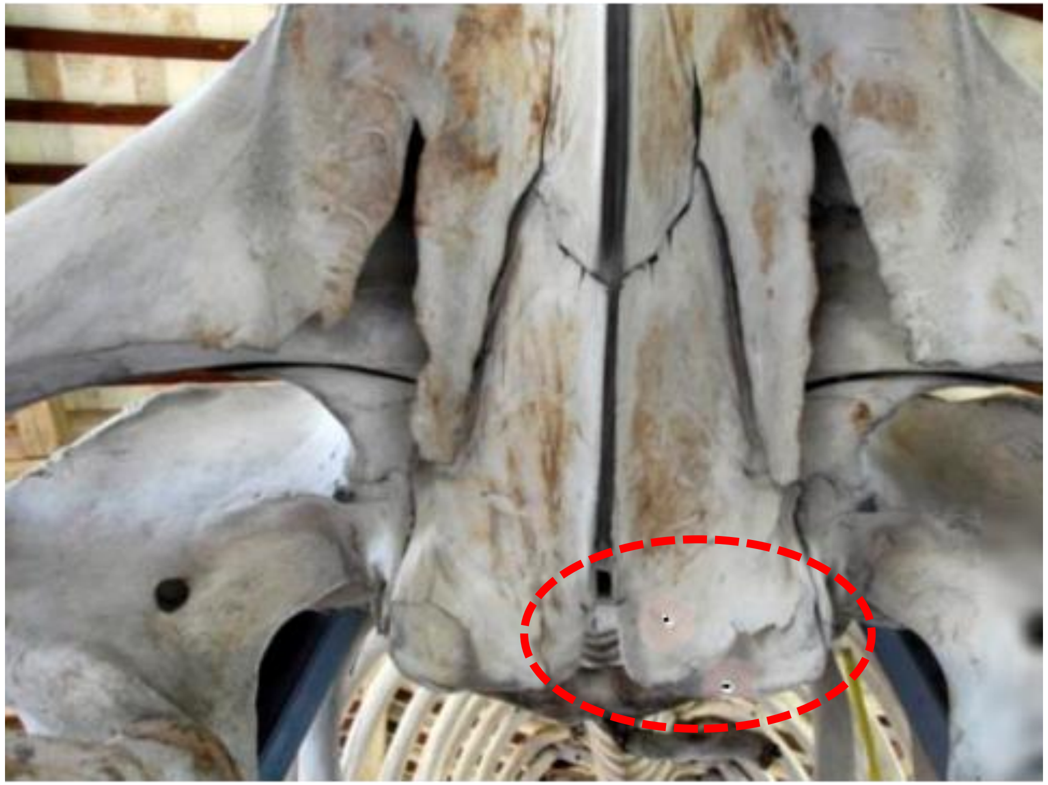

Eubalaena spp.

Copyright holder: Felix G. Marx/Museo di Storia Naturale e del Territorio, Università di Pisa, Italy

[124] 'Anteriomost point of pterygoid sinus fossa'

(0)'located anterior to foramen pseudovale (or path of mandibular branch of trigeminal nerve)'

(1)'approximately in line with anterior edge of foramen pseudovale'

(2)'located posterior to anterior edge of foramen pseudovale'

(0)

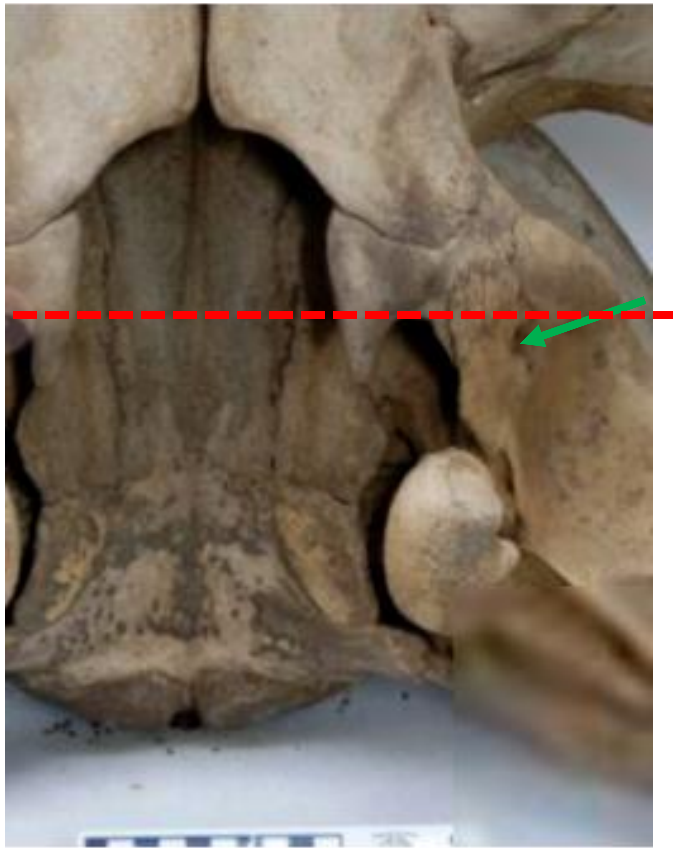

Balaenoptera acurostrata

Copyright holder: Felix G. Marx/ The Charleston Museum, Charleston, South Carolina, USA

(1)

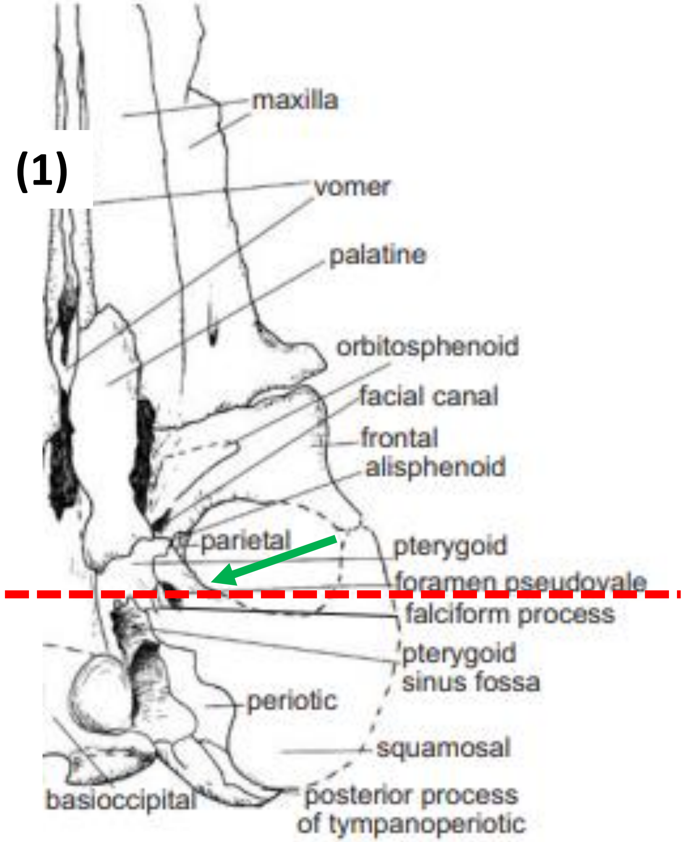

Cetotherium riabinini

Adapted from: "The anatomy of the Late Miocene baleen whale Cetotherium riabinini from Ukraine." Gol'din et al., 2013. *Acta Palaeontologica Polonica* 59.4: 795-814.

(2)

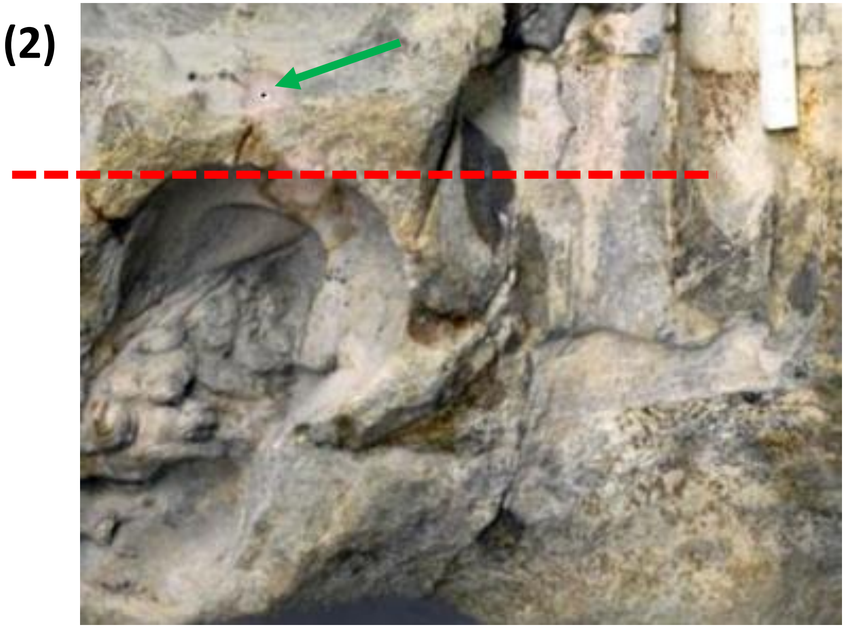

Miocaperea pulchra

Copyright holder: Felix G. Marx/Staatliches Museum für Naturkunde, Stuttgart, Germany

[125] 'Lateral lamina of pterygoid'

(0)'located entirely anterior to the anterior process of the periotic'

(1)'extending on to the anterior process of the periotic'

(0)

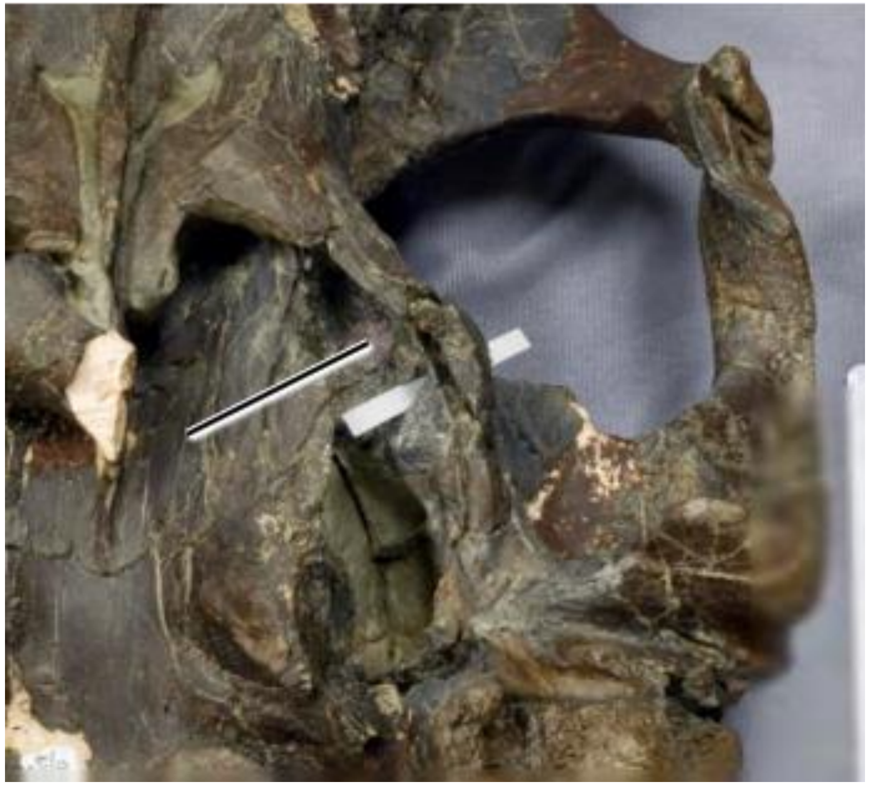

Aetiocetus weltoni

Copyright holder: Felix G. Marx/ University of California Museum of Paleontology, Berkeley, USA

(1)

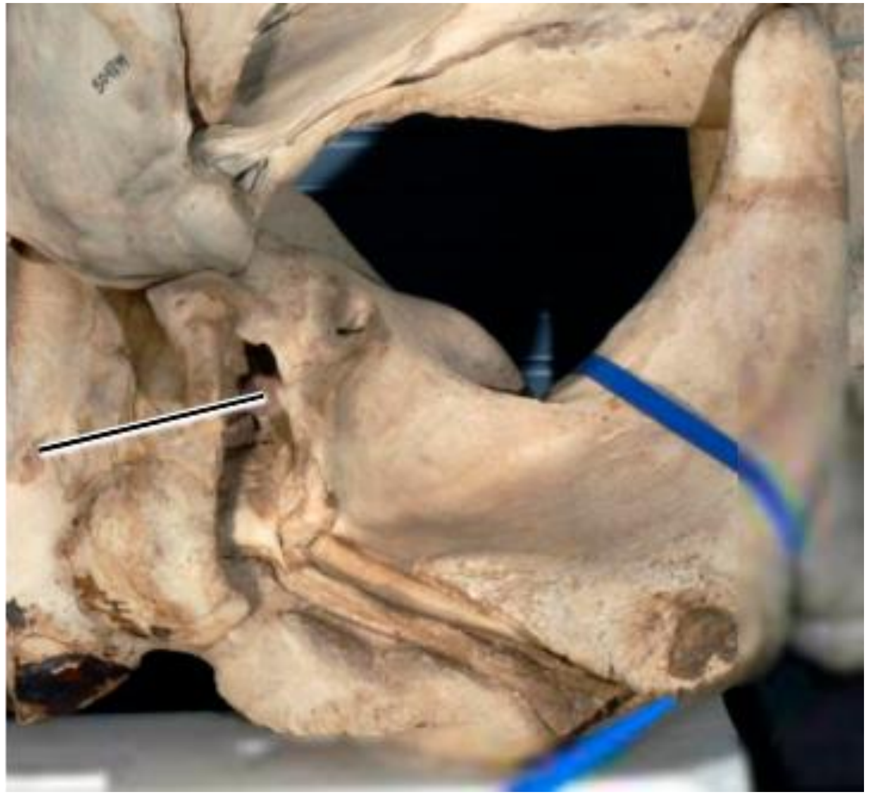

Balaenoptera borealis

Copyright holder: Felix G. Marx/ United States National Museum of Natural History, Washington DC, USA

[126] 'Shape of pterygoid hamulus'

(0)'finger-like'

(1)'expanded into a dorsoventrally flattened plate flooring the pterygoid sinus fossa'

(2)'triangular and wing-like'

(3)'reduced in size or almost absent'

(0)

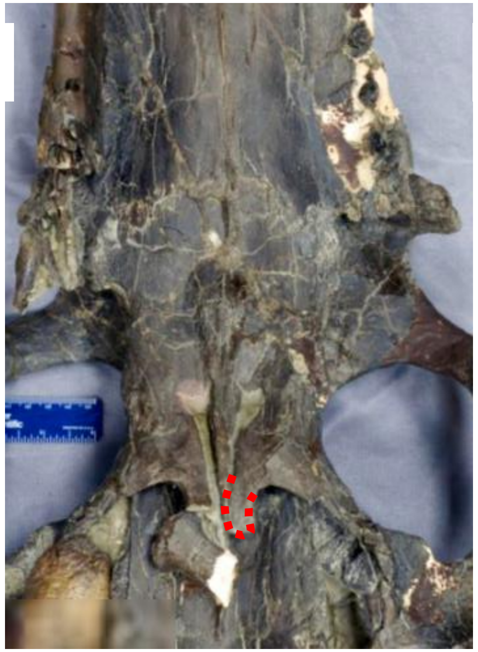

Aetiocetus weltoni

Copyright holder: Felix G. Marx/ University of California Museum of Paleontology, Berkeley, USA

(1)

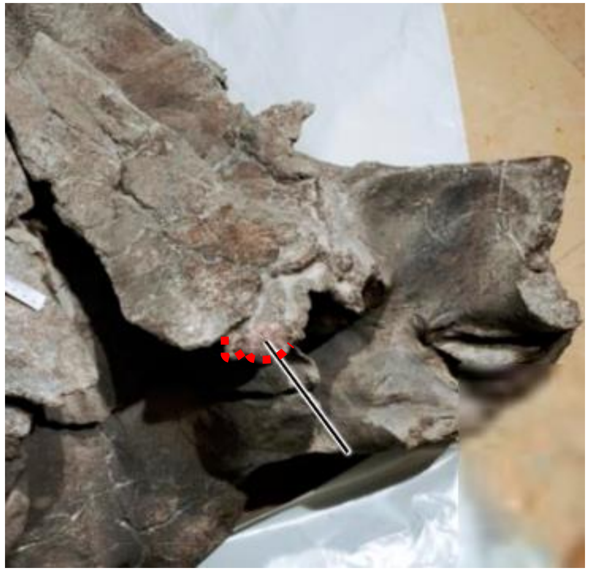

Eubalaena ianitrix

Copyright holder: Felix G. Marx/ Institut Royal des Sciences Naturelles de Belgique, Brussels, Belgium

(2)

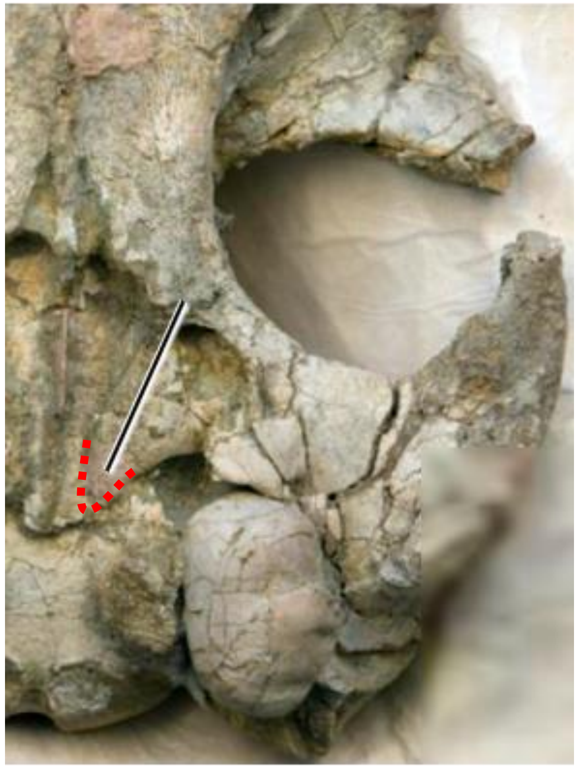

Herpetocetus morrowi

Copyright holder: Felix G. Marx/ University of California Museum of Paleontology, Berkeley, USA

(3)

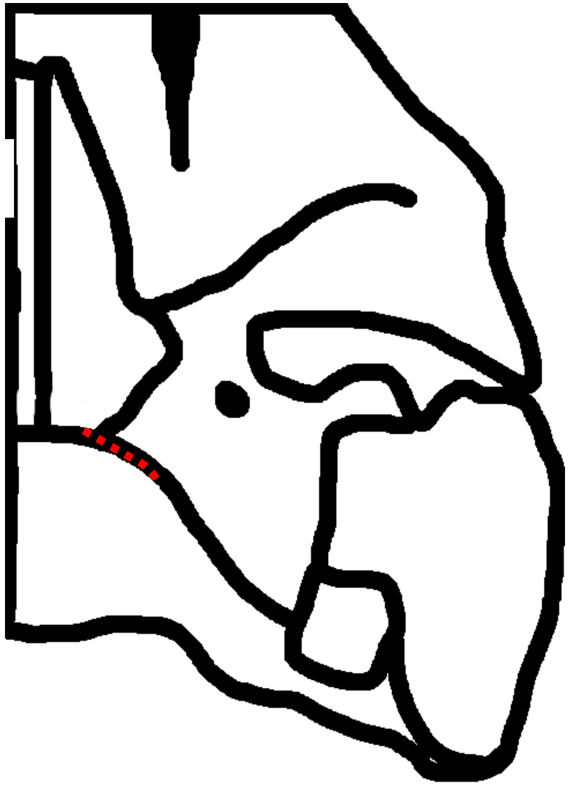

Example based on *Caperea marginata*

[127] 'Position of pterygoid hamuli in ventral view'

(0)'located adjacent to the sagittal plane and almost contacting each other'

(1)'well separated from each other'

(0)

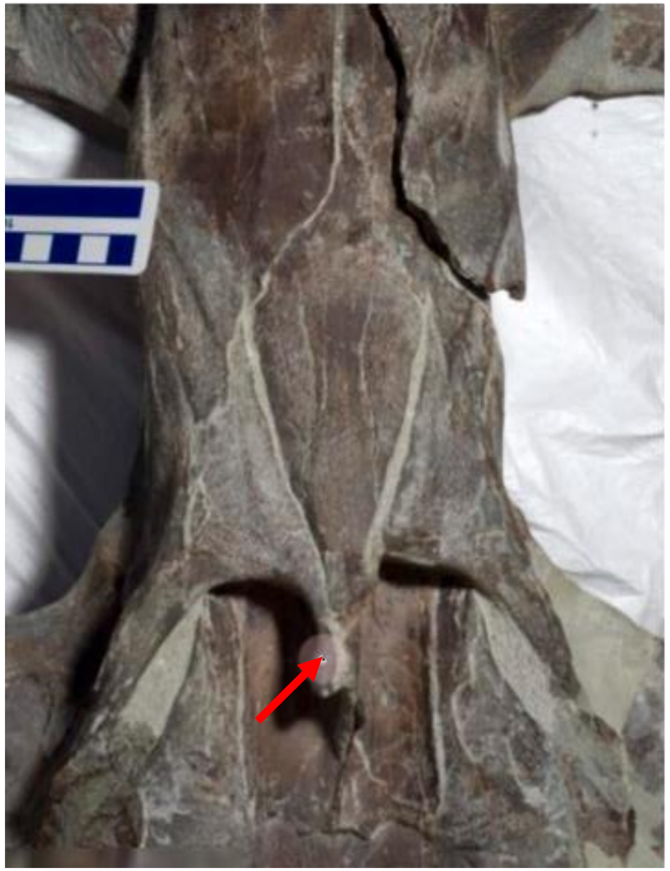

Yamatocetus canaliculatus

Copyright holder: Felix G. Marx/ Kitakyushu Museum of Natural and Human History, Kitakyushu, Kyushu, Japan

(1)

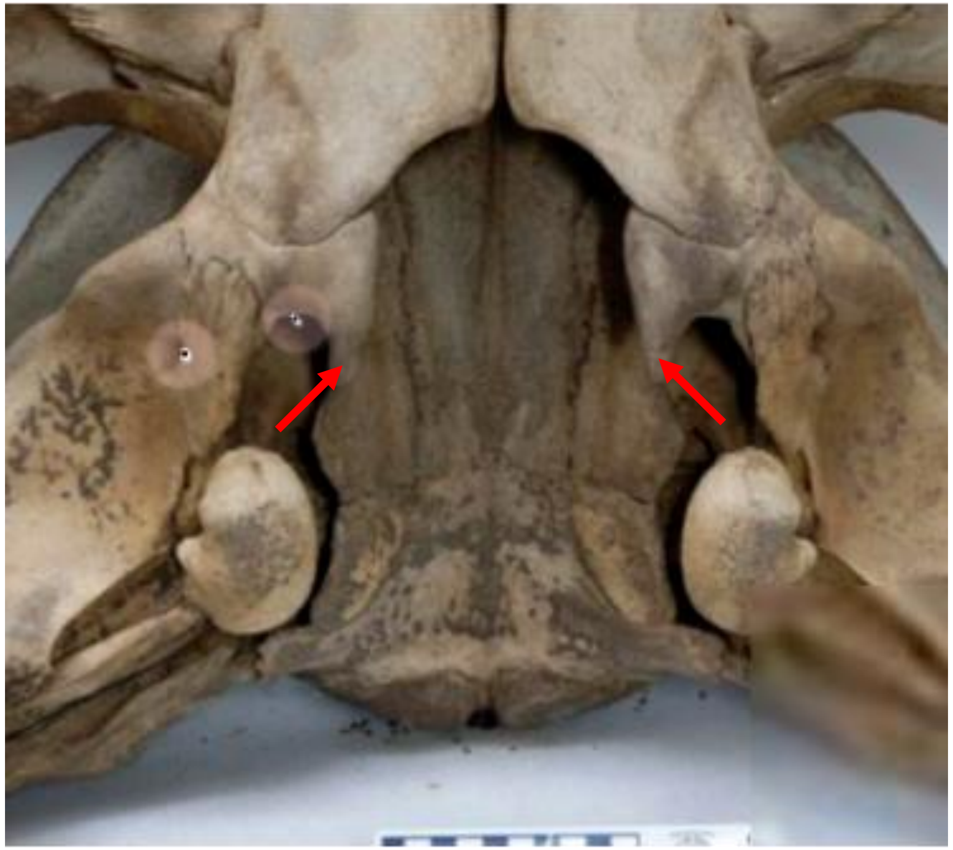

Balaenoptera acurostrata

Copyright holder: Felix G. Marx/ The Charleston Museum, Charleston, South Carolina, USA

[128] 'Position of foramen pseudovale (or path of mandibular branch of trigeminal nerve)'

(0)'located within squamosal or between squamosal and pterygoid'

(1)'as state 0 but with foramen opening posteriorly'

(2)'foramen lies within pterygoid'

(0)

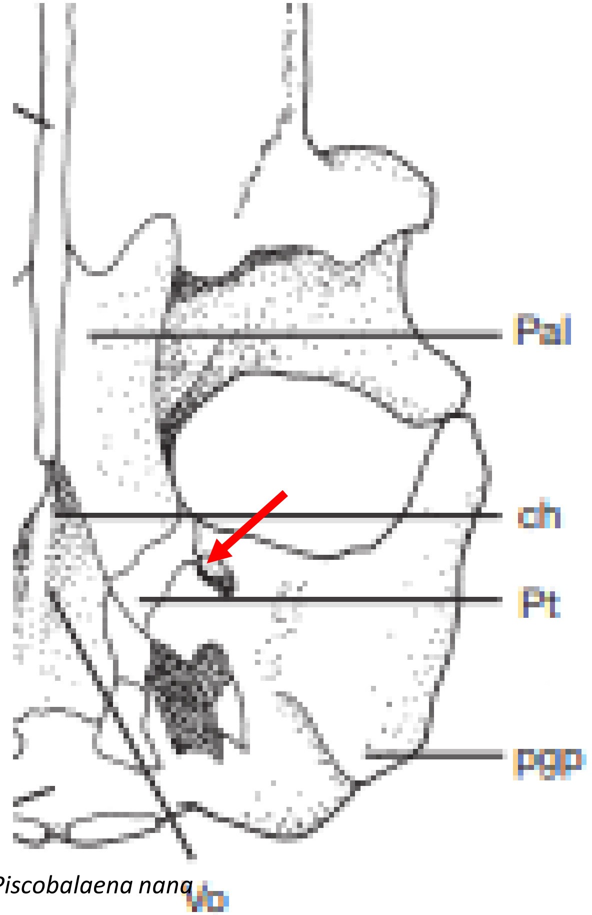

Piscobalaena nana

Adapted from: "The anatomy and relationships of *Piscobalaena nana* (Cetacea, Mysticeti), a Cetotheriidae s.s. from the early Pliocene of Peru", Bouetel and Muizon, 2006. *Geodiversitas* 28.2 (2006): 319-395.

(1)

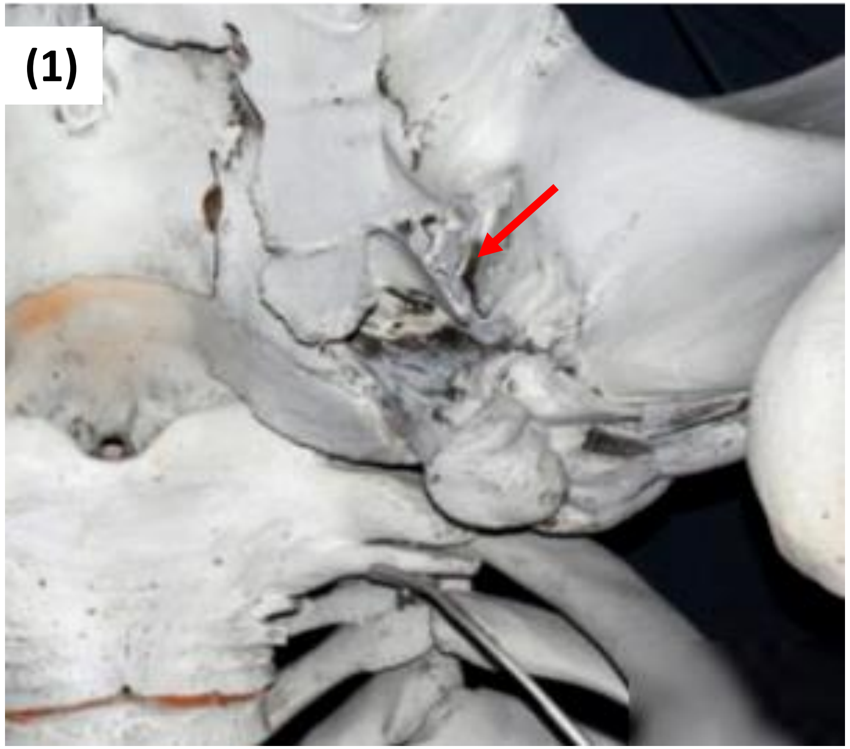

Eubalaena spp.

Copyright holder: Felix G. Marx/ Field Museum of Natural History, Chicago, USA

(2)

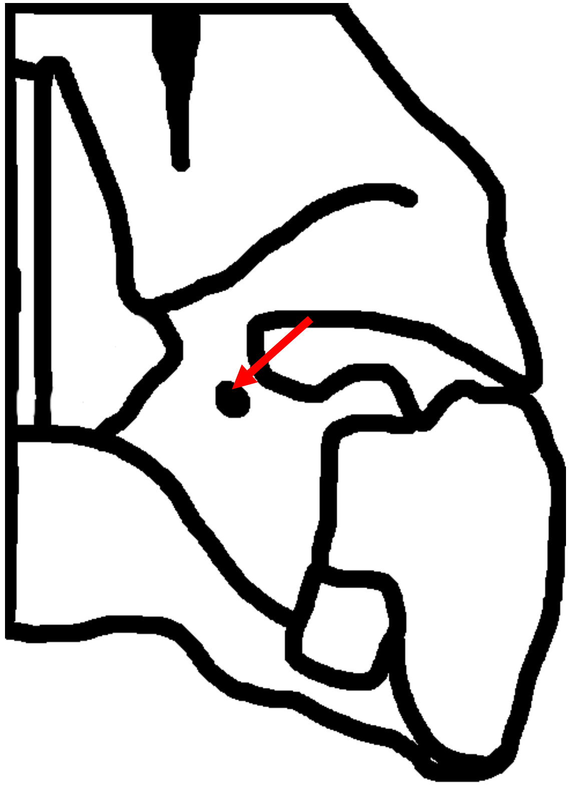

Example based on *Caperea marginata*

[129] 'Foramen pseudovale (or path of mandibular branch of trigeminal nerve) raised above more lateral portions of squamosal in ventral view'

(0)'absent'

(1)'present'

(0)

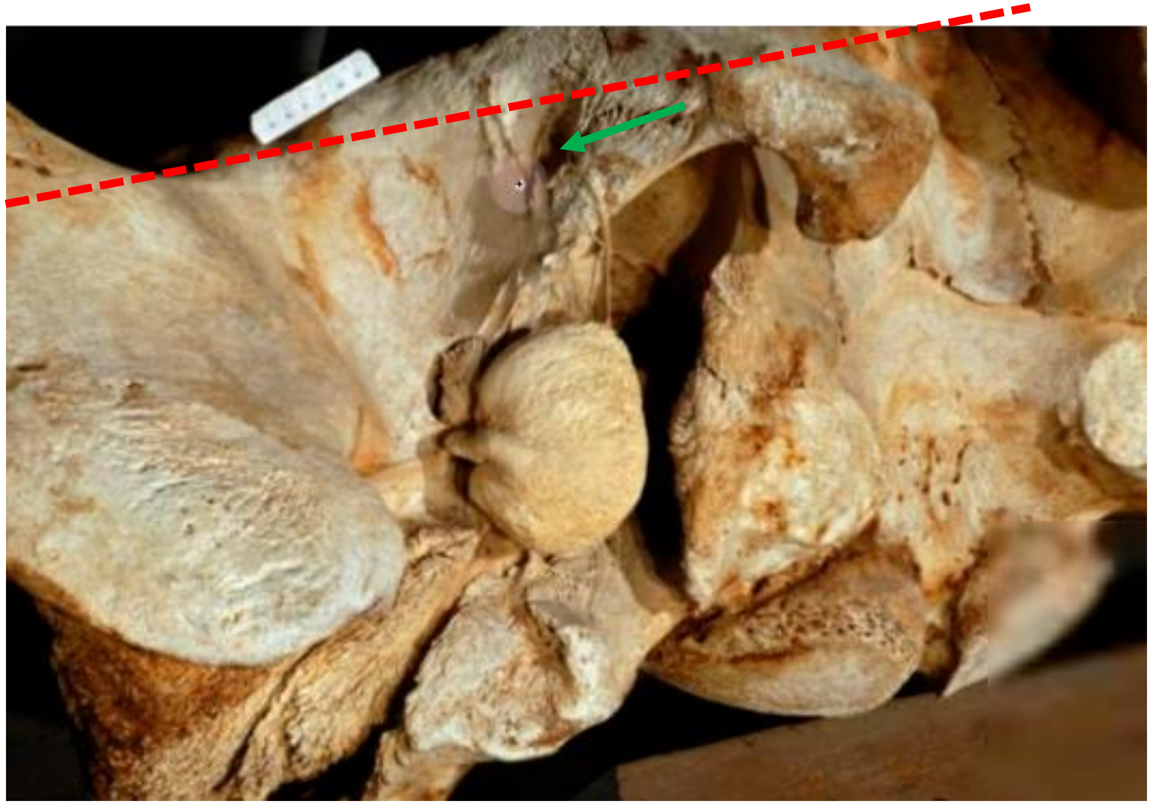

Eschrichtius robustus

Copyright holder: Felix G. Marx/ Unites States National Museum of Natural History, Washington DC, USA

(1)

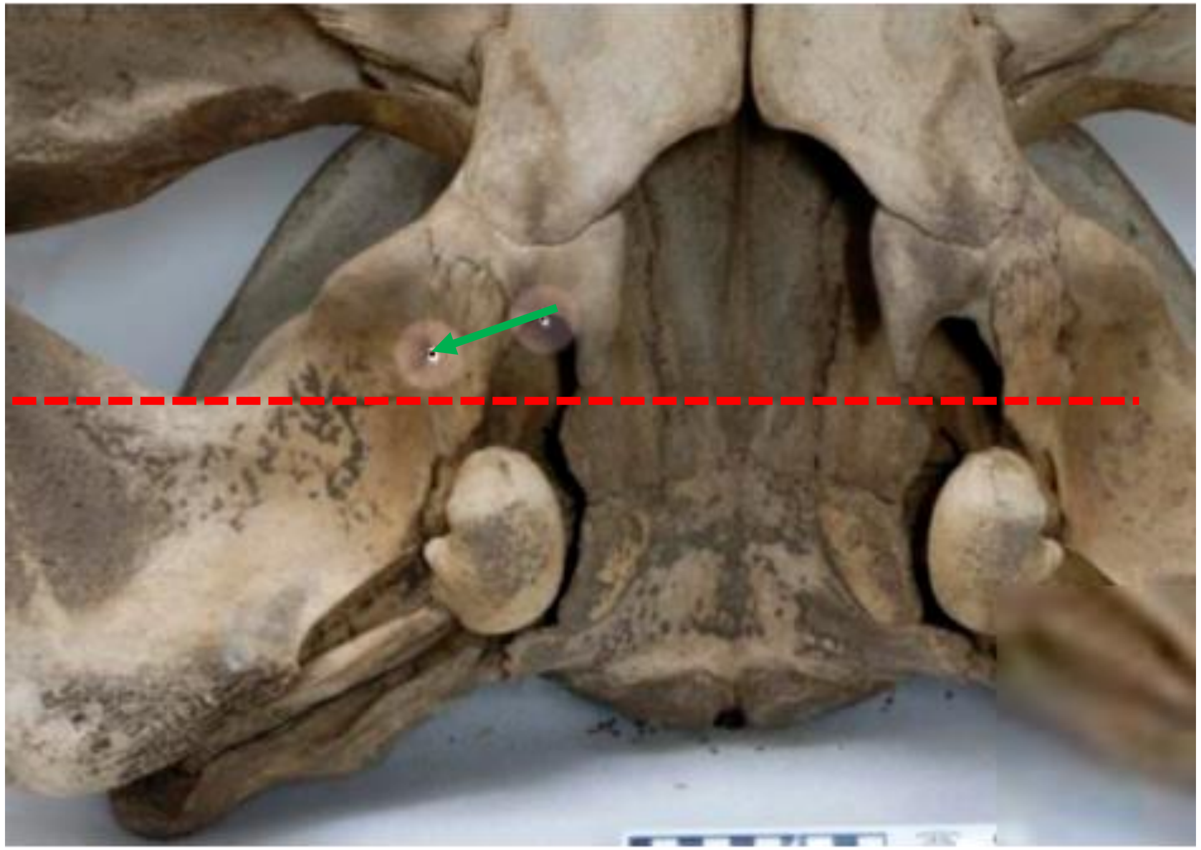

Balaenoptera acurostrata

Copyright holder: Felix G. Marx/ The Charleston Museum, Charleston, South Carolina, USA

[130] 'Fossa on squamosal for reception of sigmoid process of tympanic bulla'

(0) 'present'

(1) 'absent or poorly defined'

(0)

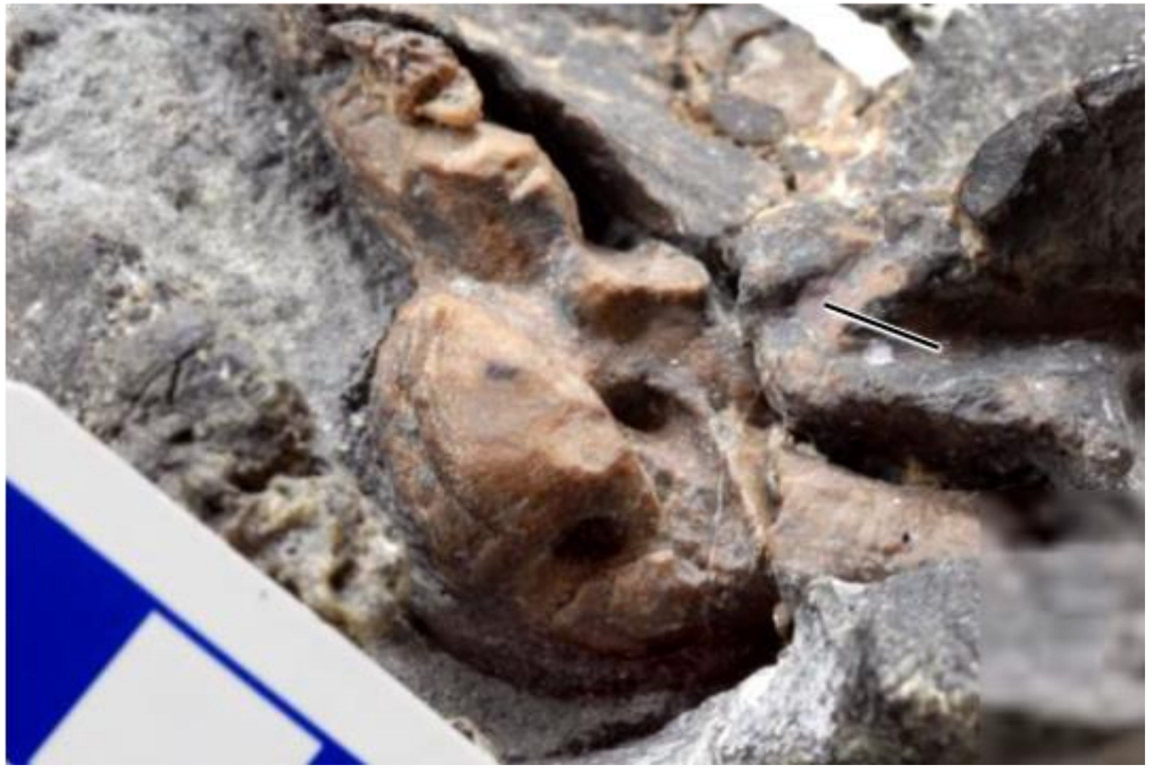

Chonecetus sookensis

Copyright holder: Felix G. Marx/ Canadian Museum of Nature, Ottawa, Canada

(1)

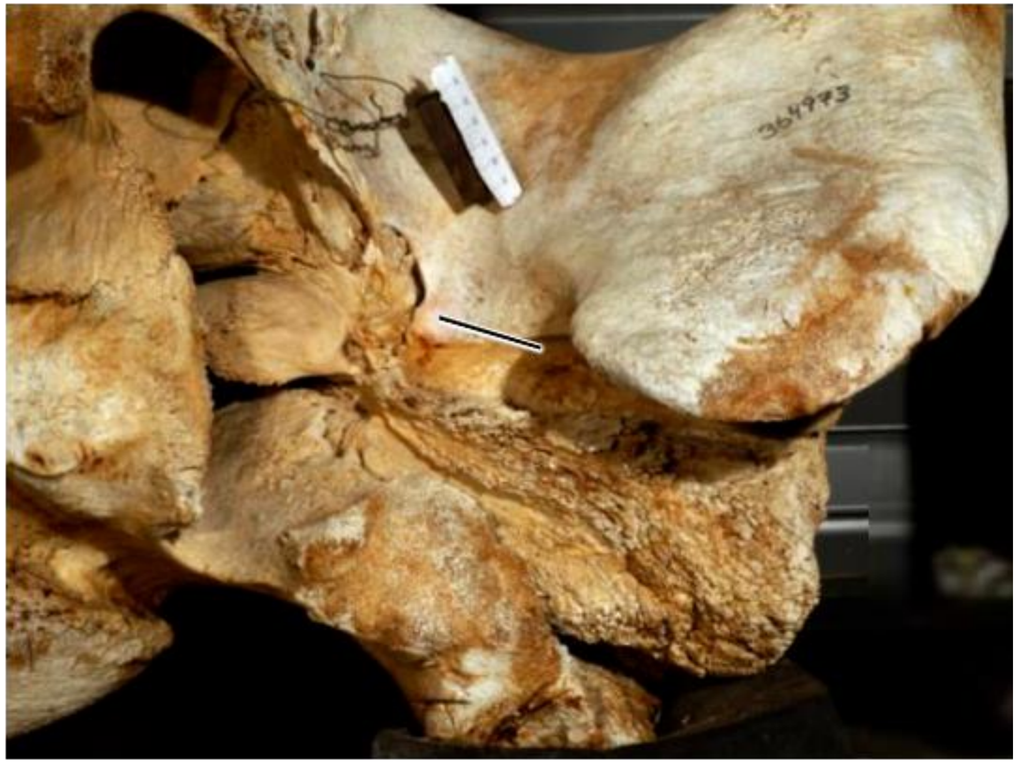

Eschrichtius robustus

Copyright holder: Felix G. Marx/ Unites States National Museum of Natural History, Washington DC, USA

[131] 'Base of postglenoid process in ventral view'

(0) 'in transverse line with or posterior to the posterior half of the tympanic bulla'

(1) 'in transverse line with the centre of the tympanic bulla'

(2) 'in transverse line with or anterior to the anterior half of the tympanic bulla'

(0)

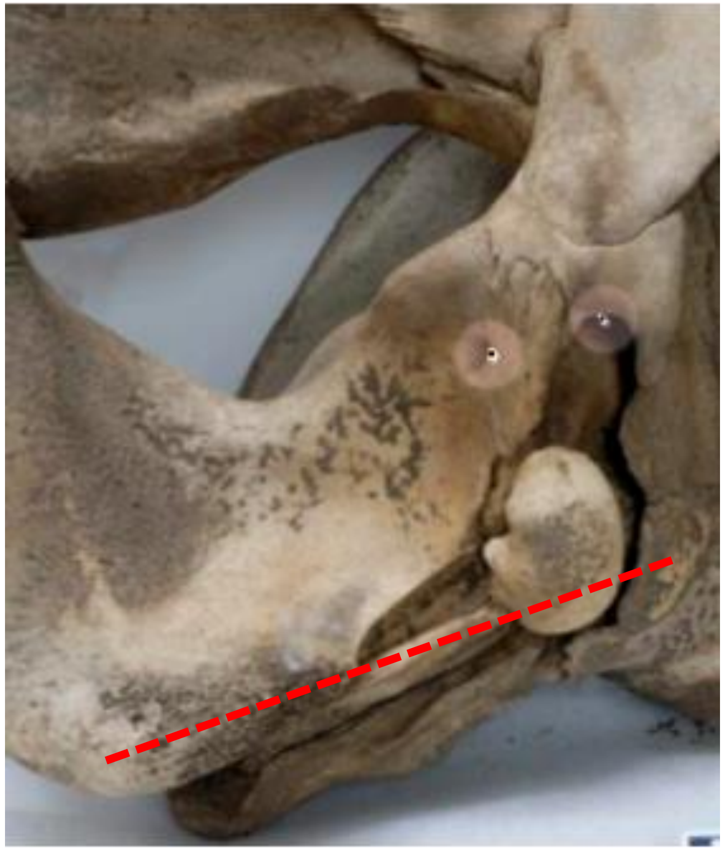

Balaenoptera acurostrata

Copyright holder: Felix G. Marx/ The Charleston Museum, Charleston, South Carolina, USA

(1)

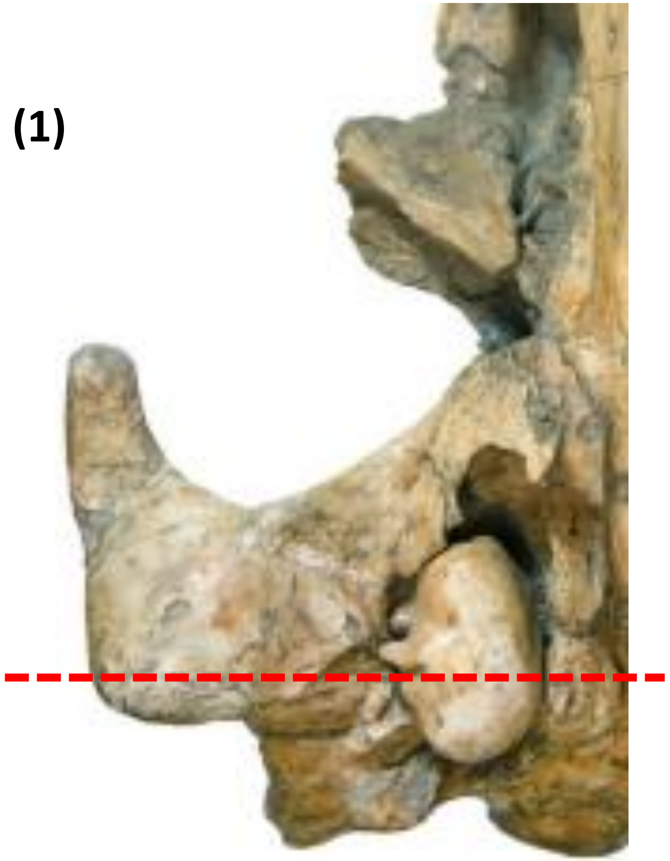

Tiucetus rosae

Adapted from: *"A new Miocene baleen whale from Peru deciphers the dawn of cetotheriids." Marx, Lambert, and De Muizon, 2017. Royal Society Open Science 4.9: 170560.*

(2)

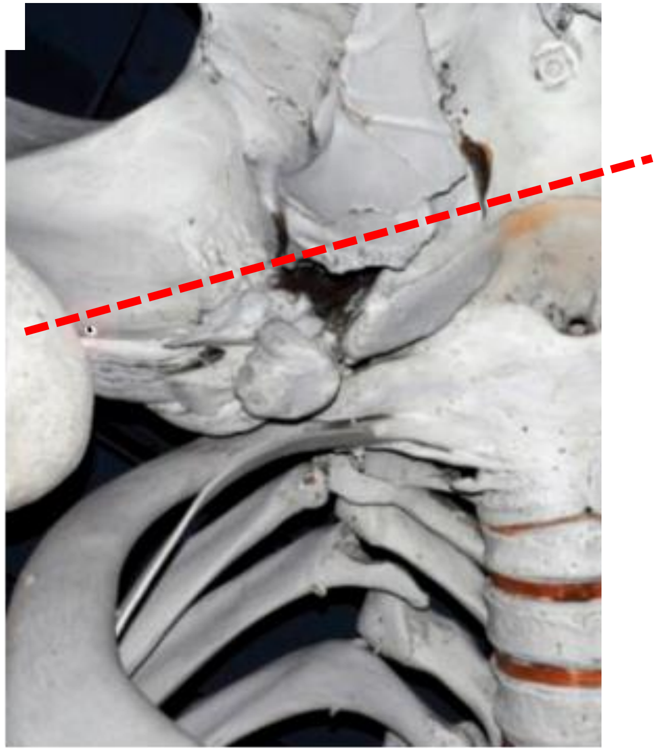

Eubalaena spp.

Copyright holder: Felix G. Marx/ Field Museum of Natural History, Chicago, USA

[132] 'Ventral border of sagittal part of vomer (nasal septum) in ventral view'

(0)'posteriormost portion projects beyond the posterior border of the palatines and is visible in ventral view'

(1)'completely covered by palatines'

(0)

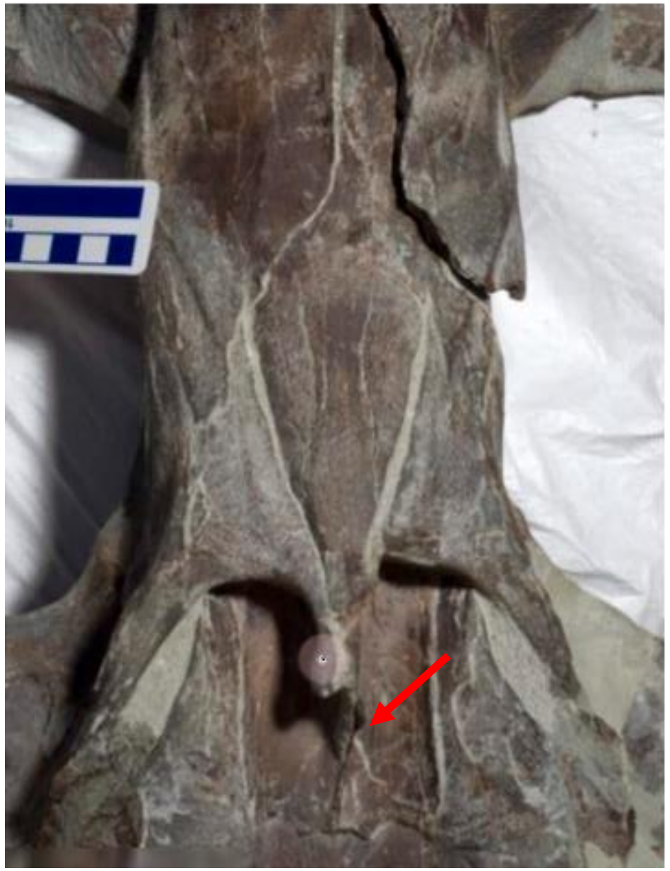

Yamatocetus canaliculatus

Copyright holder: Felix G. Marx/ Kitakyushu Museum of Natural and Human History, Kitakyushu, Kyushu, Japan

(1)

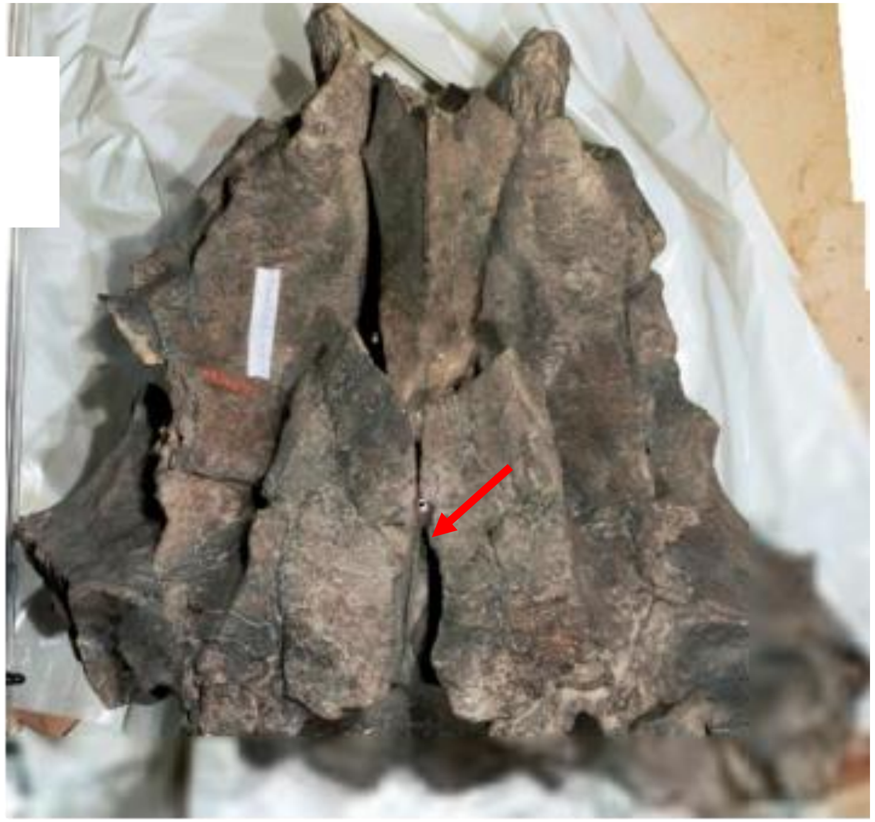

Eubalaena ianitrix

Copyright holder: Felix G. Marx/ Institut Royal des Sciences Naturelles de Belgique, Brussels, Belgium

[133] 'Basioccipital crest'

(0) 'narrow transversely'

(1) 'wide and bulbous'

(0)

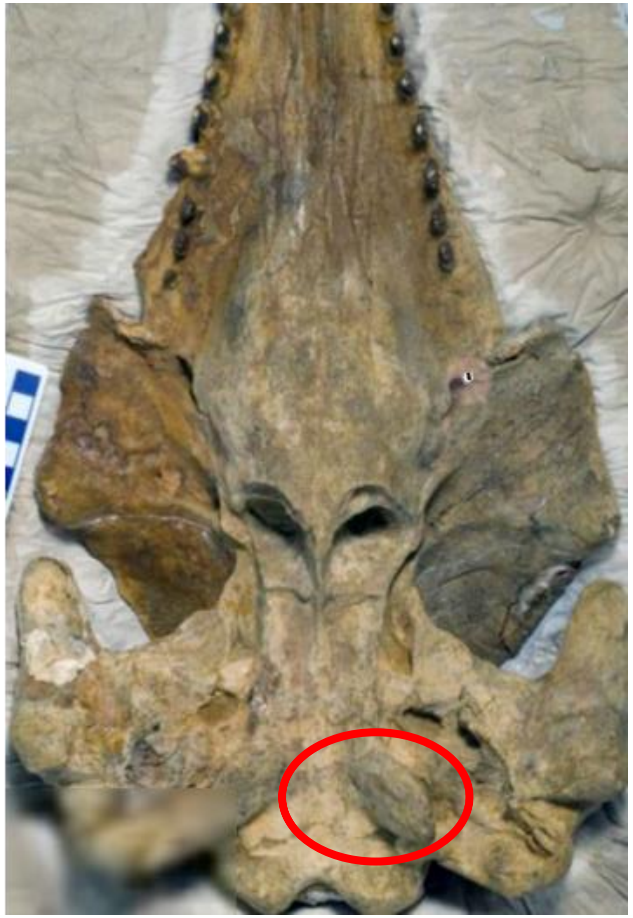

Waipatia maerewhenua

Copyright holder: Felix G. Marx/ University of Otago Museum of Geology, Dunedin, New Zealand

(1)

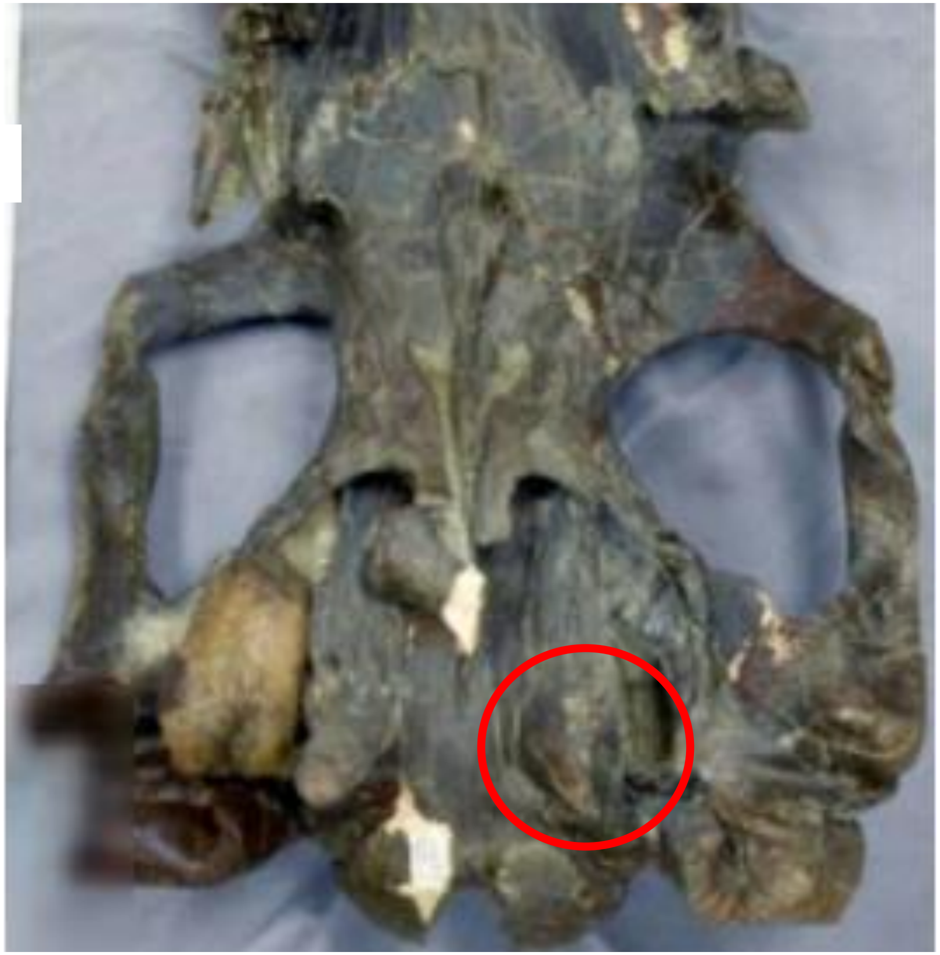

Aetiocetus weltoni

Copyright holder: Felix G. Marx/ University of California Museum of Paleontology, Berkeley, USA

[134] 'Lateral border of basioccipital crest in ventral view'

(0)'straight'

(1)'concave'

(0)

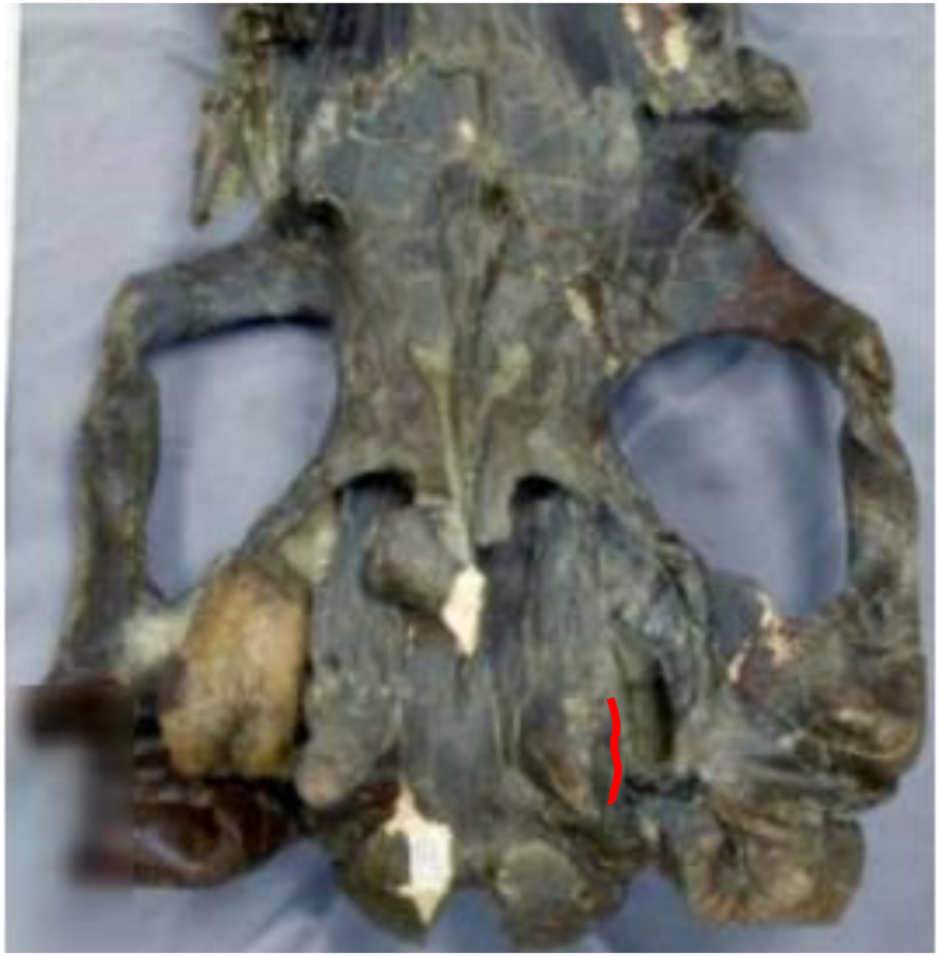

Aetiocetus weltoni

Copyright holder: Felix G. Marx/ University of California Museum of Paleontology, Berkeley, USA

(1)

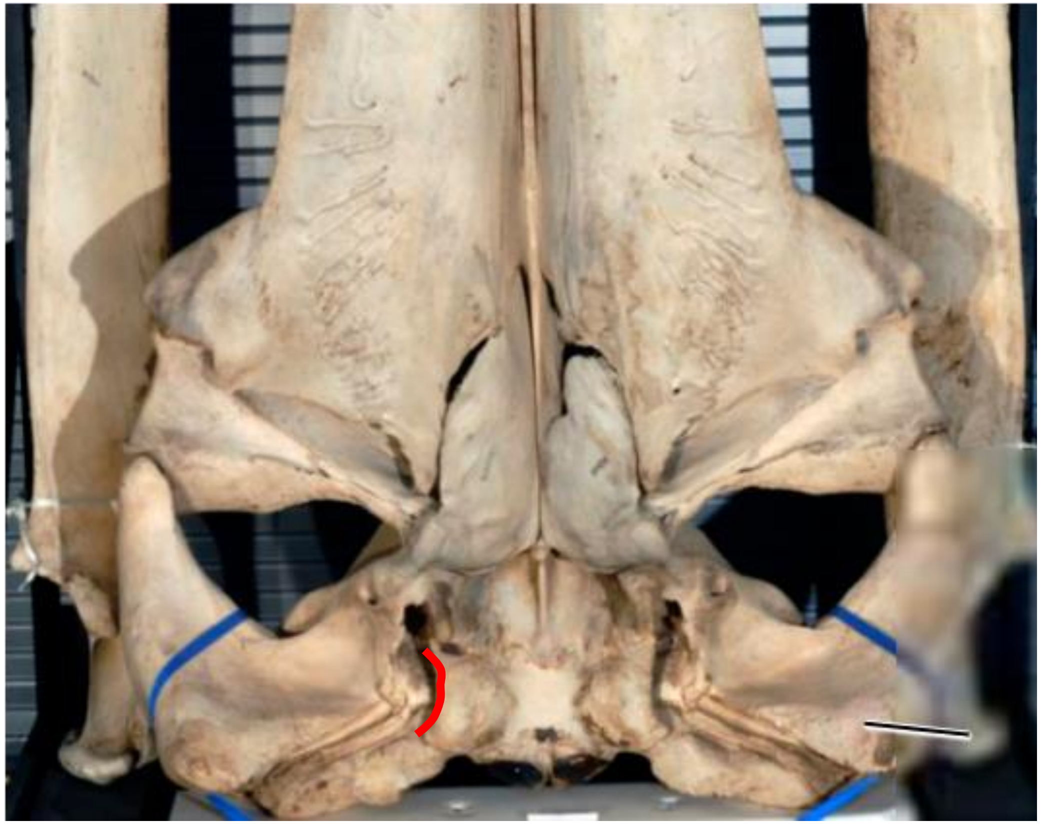

Balaenoptera borealis

Copyright holder: Felix G. Marx/ United States National Museum of Natural History, Washington DC, USA

[135] 'Ventromedial corner of paroccipital process in posterior view'

(0)'located more ventrally than the basioccipital crest'

(1)'level with or more dorsal than the basioccipital crest'

(0)

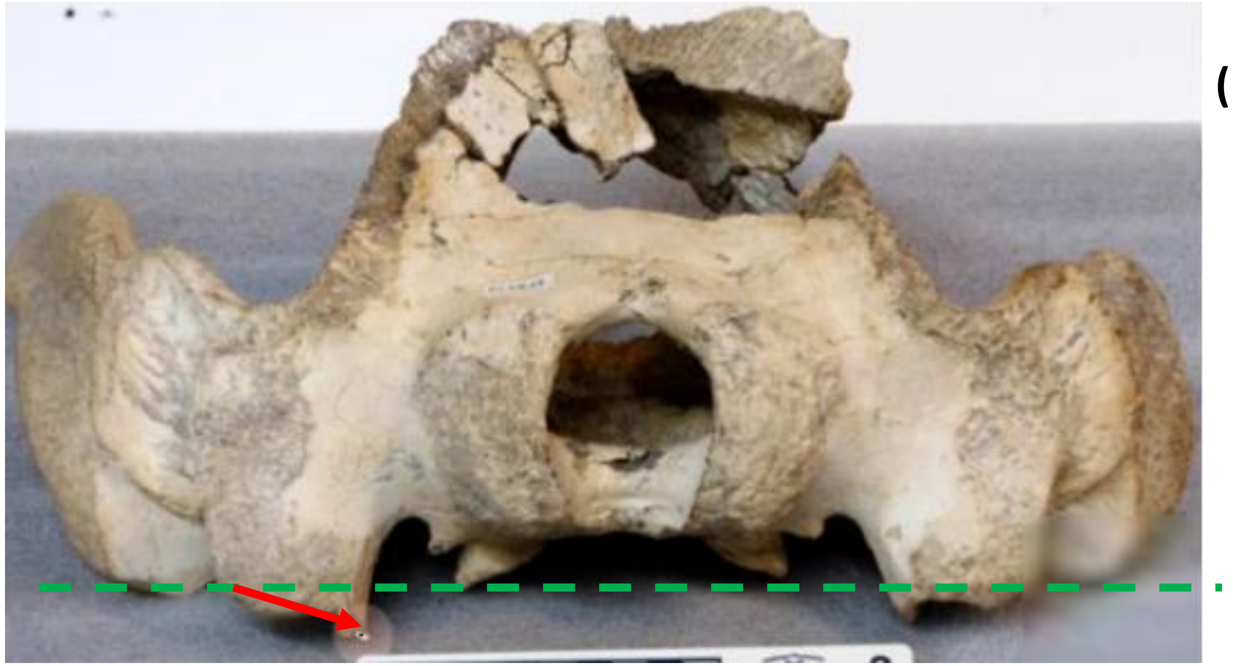

Micromysticetus rothauseni

Copyright holder: Felix G. Marx/ The Charleston Museum, Charleston, USA

(1)

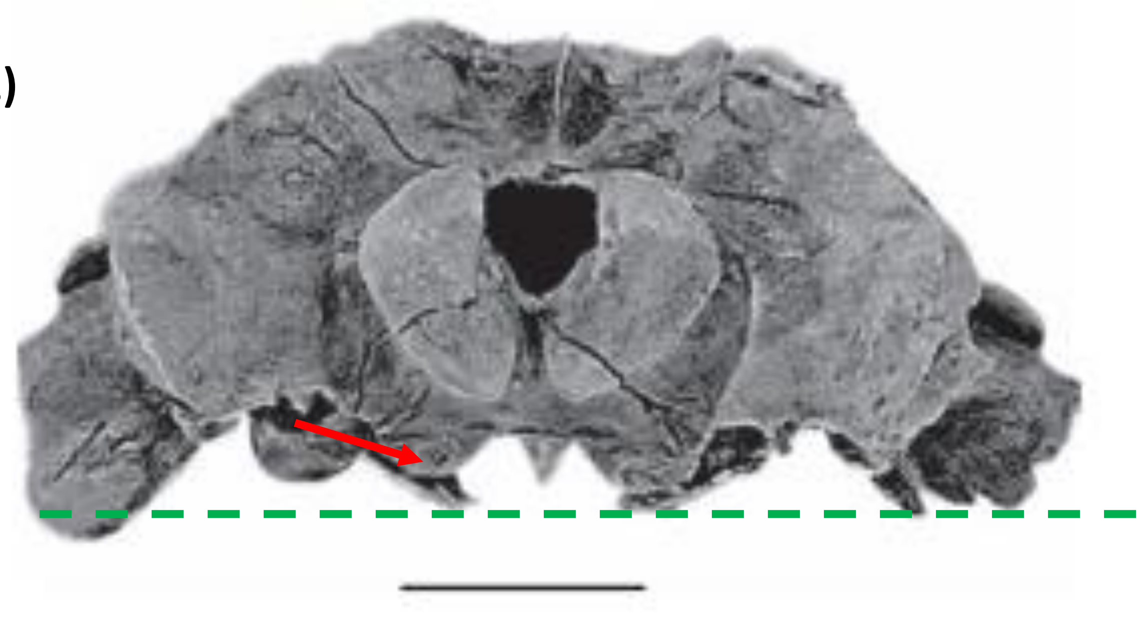

Piscobalaena nana

Adapted from: "The anatomy and relationships of *Piscobalaena nana* (Cetacea, Mysticeti), a Cetotheriidae s.s. from the early Pliocene of Peru", Bouetel and Muizon, 2006. *Geodiversitas* 28.2 (2006): 319-395.

[136] 'Posteriormost point of paroccipital process'

(0)'located more anteriorly than posterior edge of occipital condyle'

(1)'level with or posterior to posterior edge of condyle'

(0)

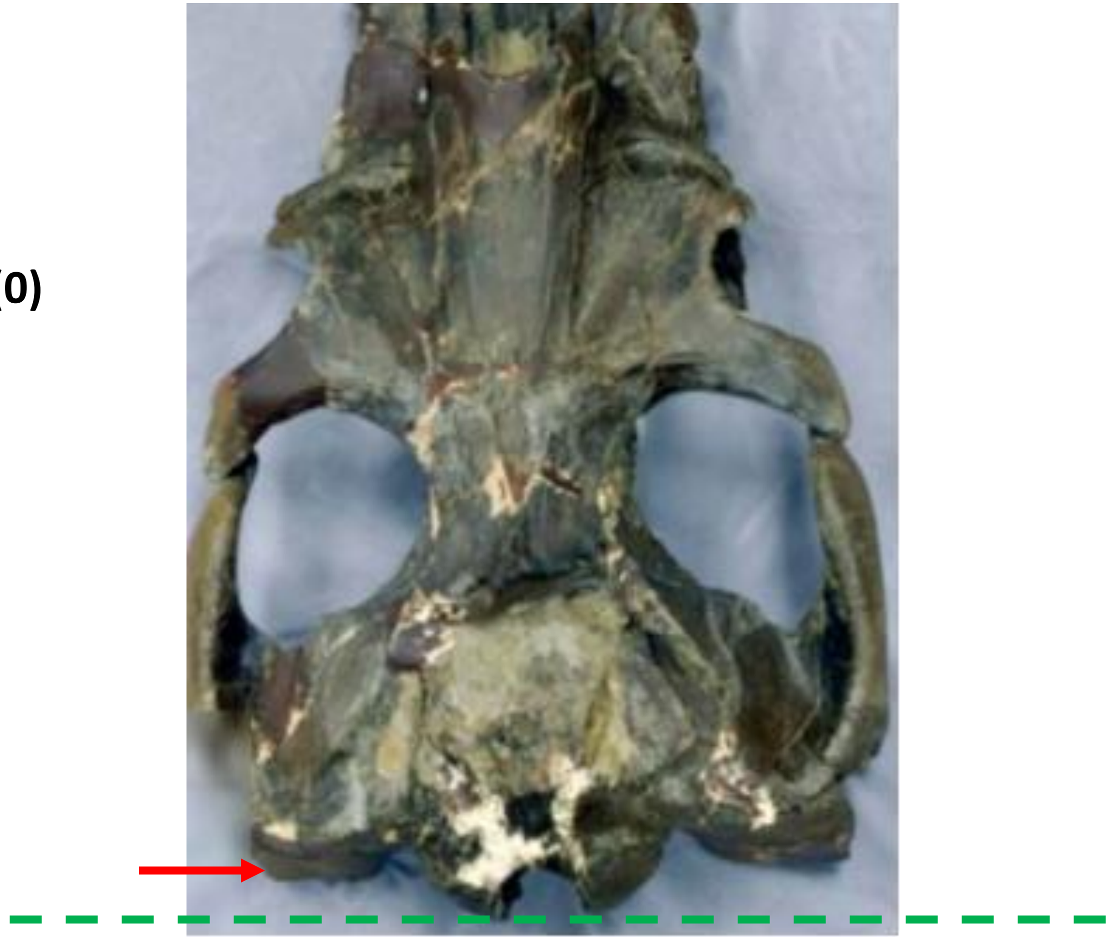

Aetiocetus weltoni

Copyright holder: Felix G. Marx/ University of California Museum of Paleontology, Berkeley, USA

(1)

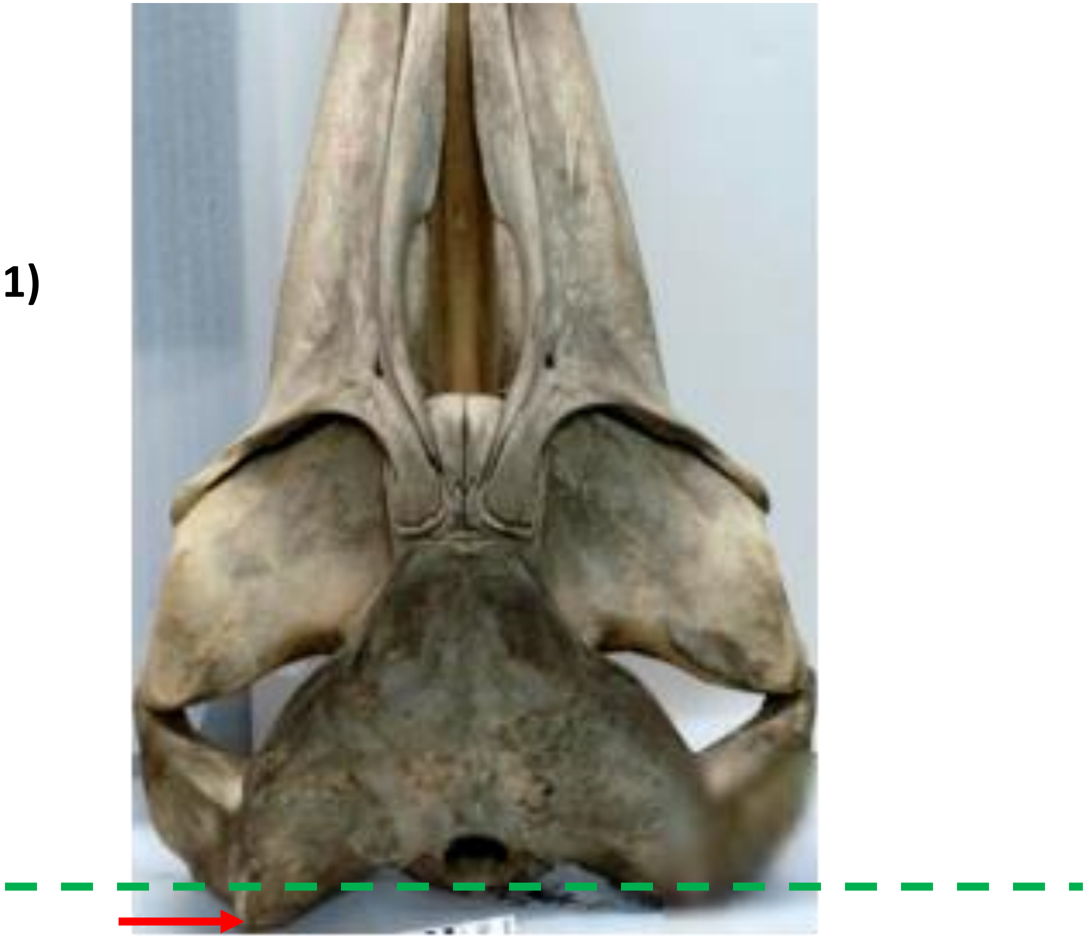

Balaenoptera acutorostrata

Copyright holder: Felix G. Marx/ The Charleston Museum, Charleston, South Carolina, USA

[143] 'Tympanohyal'

(0)'indistinct'

(1)'clearly differentiated trumpet-shaped element fused to crista parotica'

(0)

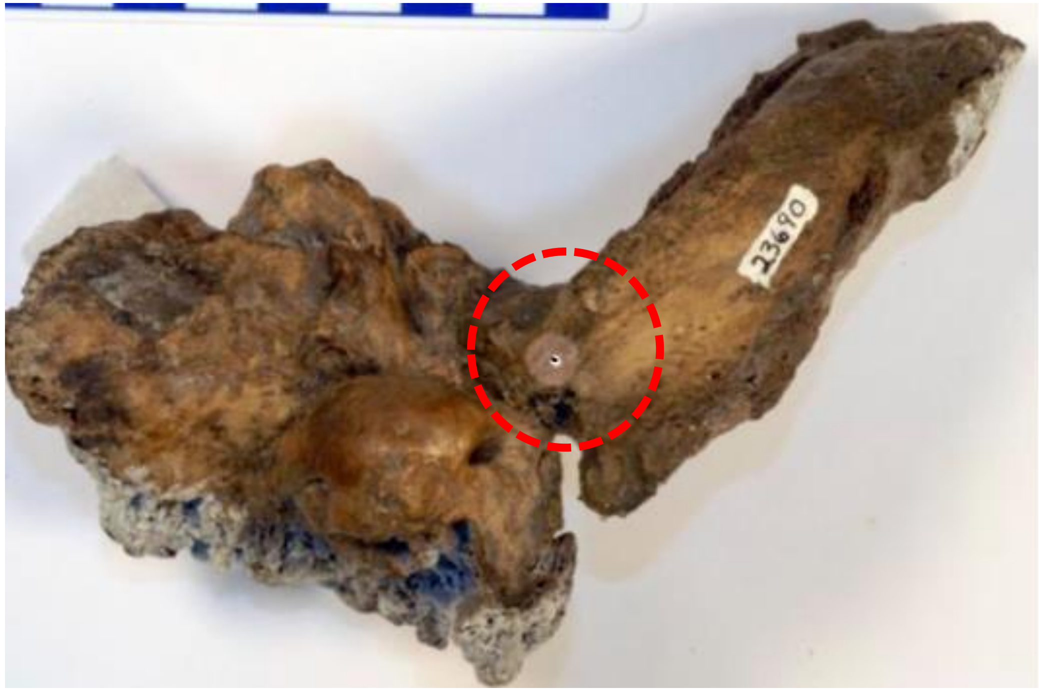

(1)

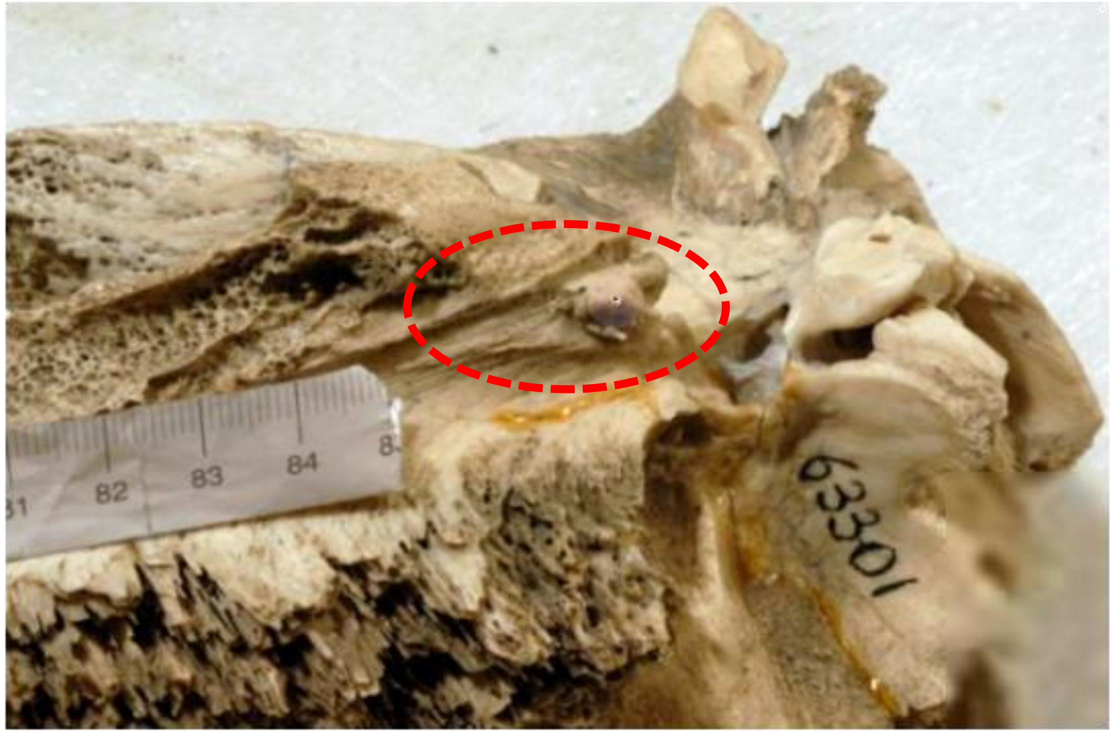

Aglaocetus patulus

Balaena mysticetus

Copyright holder: Felix G. Marx/ United States National Museum of Natural History, Washington DC, USA

Copyright holder: Felix G. Marx/United States National Museum of Natural History, Washington DC, USA

[144] 'Cranial elongation of pars cochlearis towards cranial cavity'

(0) 'absent'

(1) 'present'

(2) 'as state 1, but with only the anterior side of the pars cochlearis being elongated'

(0)

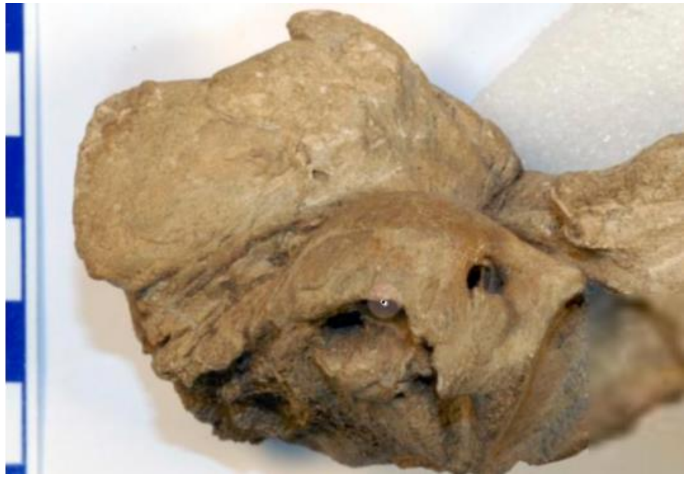

Aglaocetus patulus

Copyright holder: Felix G. Marx/ United States National Museum of Natural History, Washington DC, USA

(1)

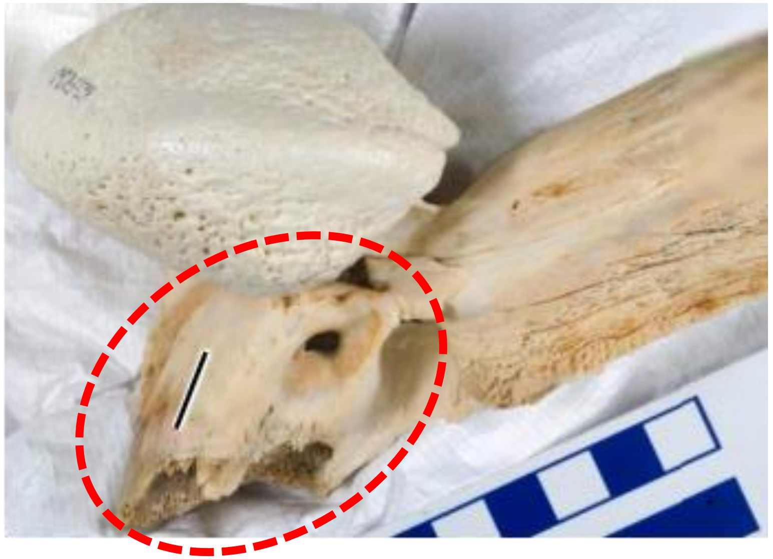

Balaenoptera acutorostrata

Copyright holder: Felix G. Marx/ National Museum of Nature and Science, Tokyo, Japan

(2)

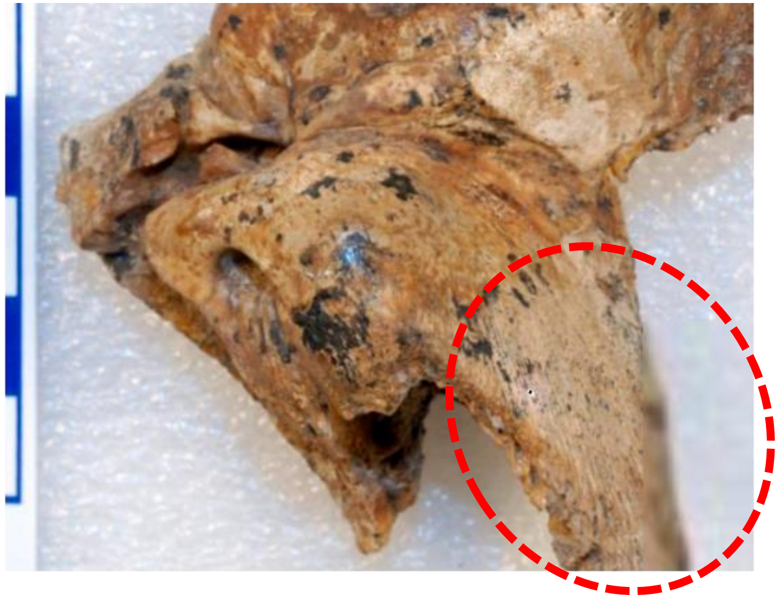

Megaptera miocaena

Copyright holder: Felix G. Marx/United States National Museum of Natural History, Washington DC, USA

[145] 'Attachment of anterior process to pars cochlearis'

(0) 'absent'

(1) 'present'

(0)

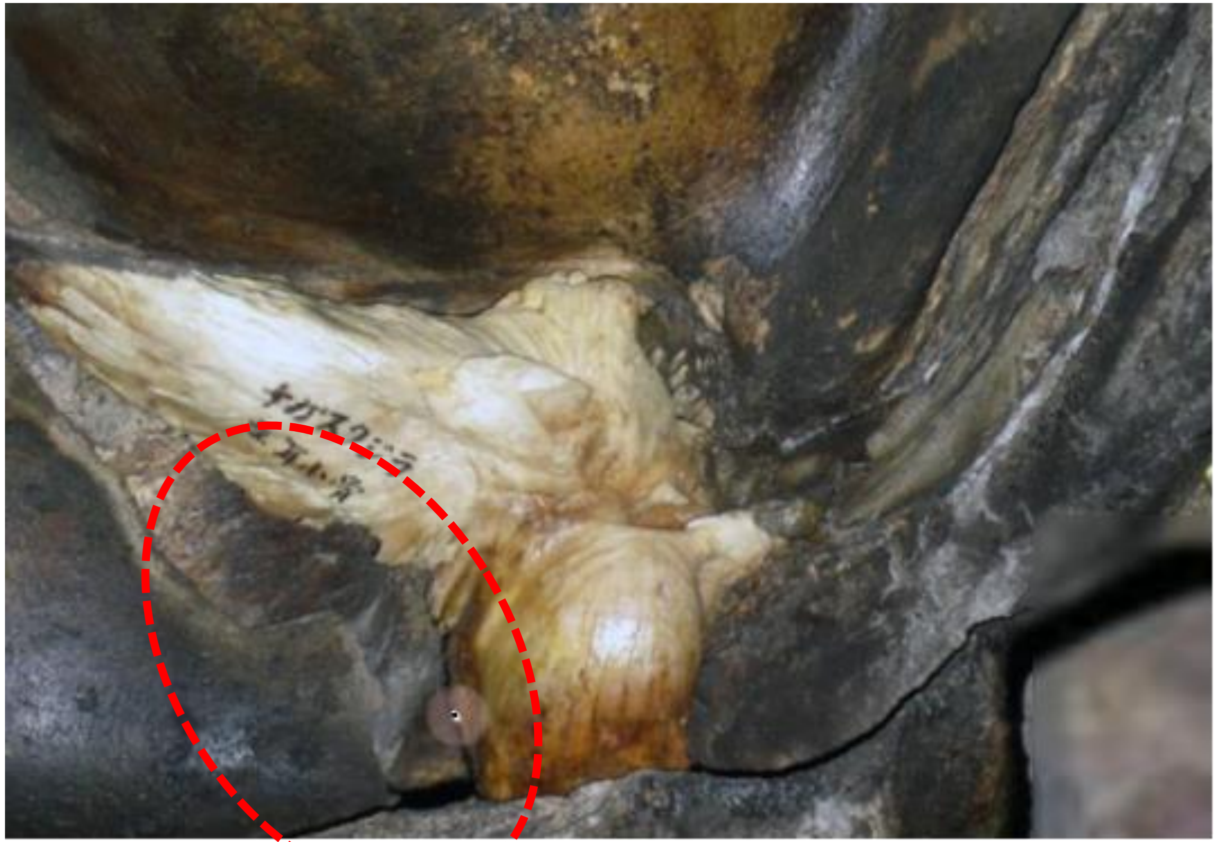

Balaenoptera physalus

Copyright holder: Felix G. Marx/ Osaka Museum of Natural History, Osaka, Japan

(1)

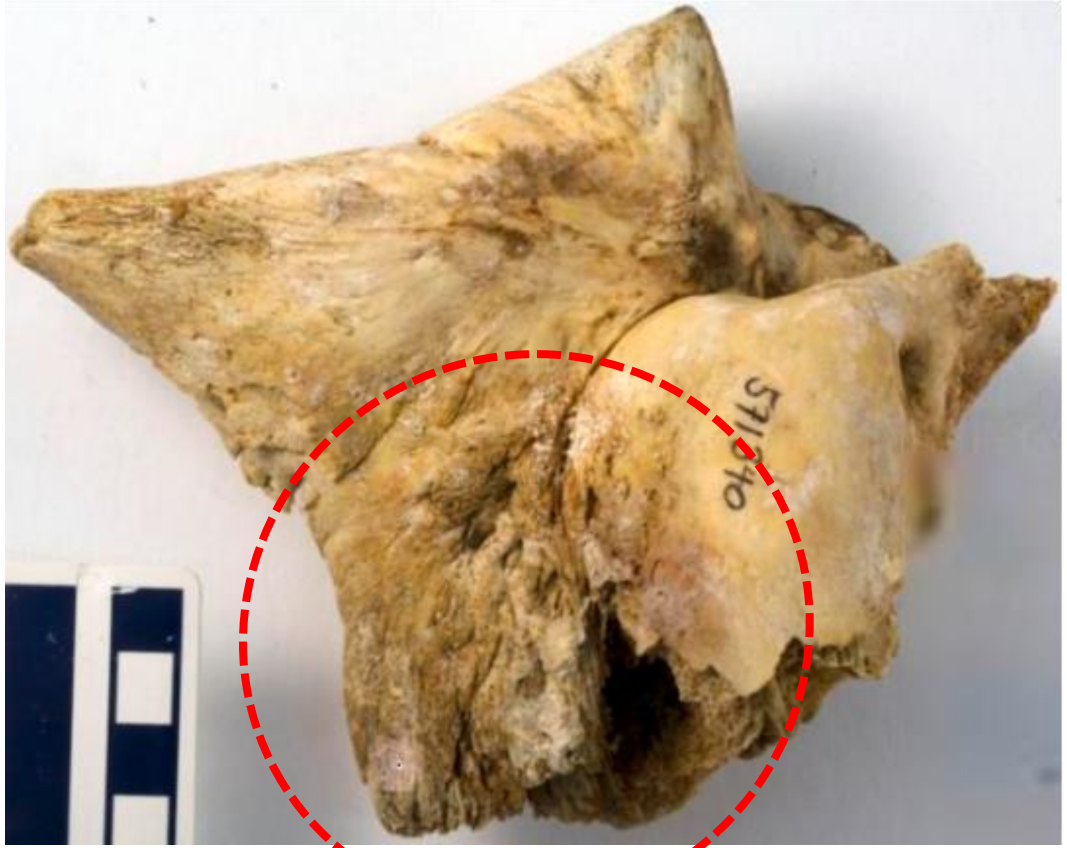

Balaenoptera borealis

Copyright holder: Felix G. Marx/United States National Museum of Natural History, Washington DC, USA

[146] 'Anterior process of periotic in lateral view'

(0)'squared off or rounded'

(1)'triangular'

(2)'anterior border of process is two-bladed and L-shaped'

(0)

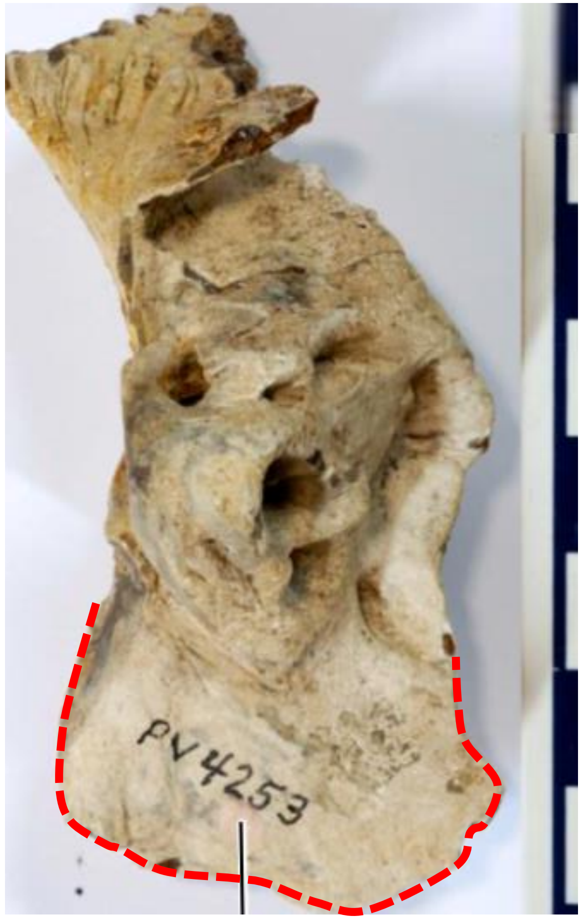

Eomysticetus whitmorei

Copyright holder: Felix G. Marx/ The Charleston Museum, Charleston, South Carolina, USA

(1)

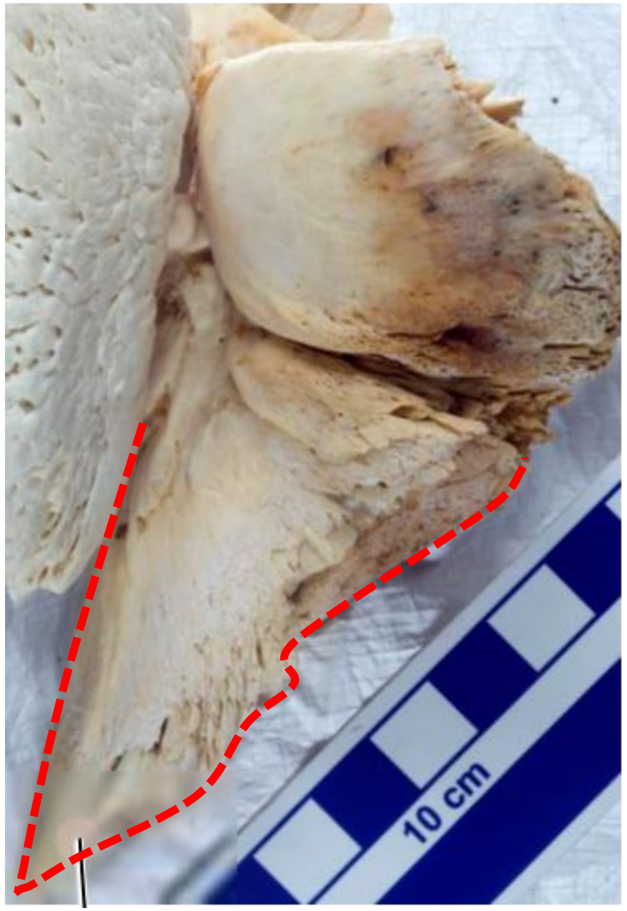

Balaenoptera acutorostrata

Copyright holder: Felix G. Marx/ National Museum of Nature and Science, Tokyo, Japan

(2)

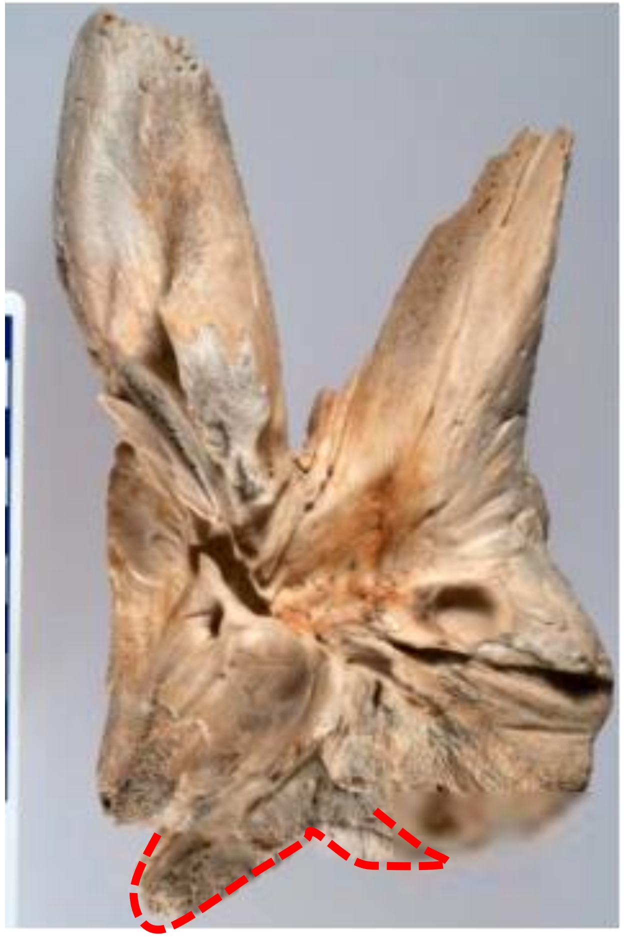

Eubalaena spp.

Copyright holder: Felix G. Marx/ Museum of Geology, University of Otago, Dunedin, New Zealand

[147] 'Shape of anteroventral angle of anterior process of periotic in medial or lateral view'

(0)'rounded or forms a relatively blunt angle’

(1)'slender and tapering to a point'

(0)

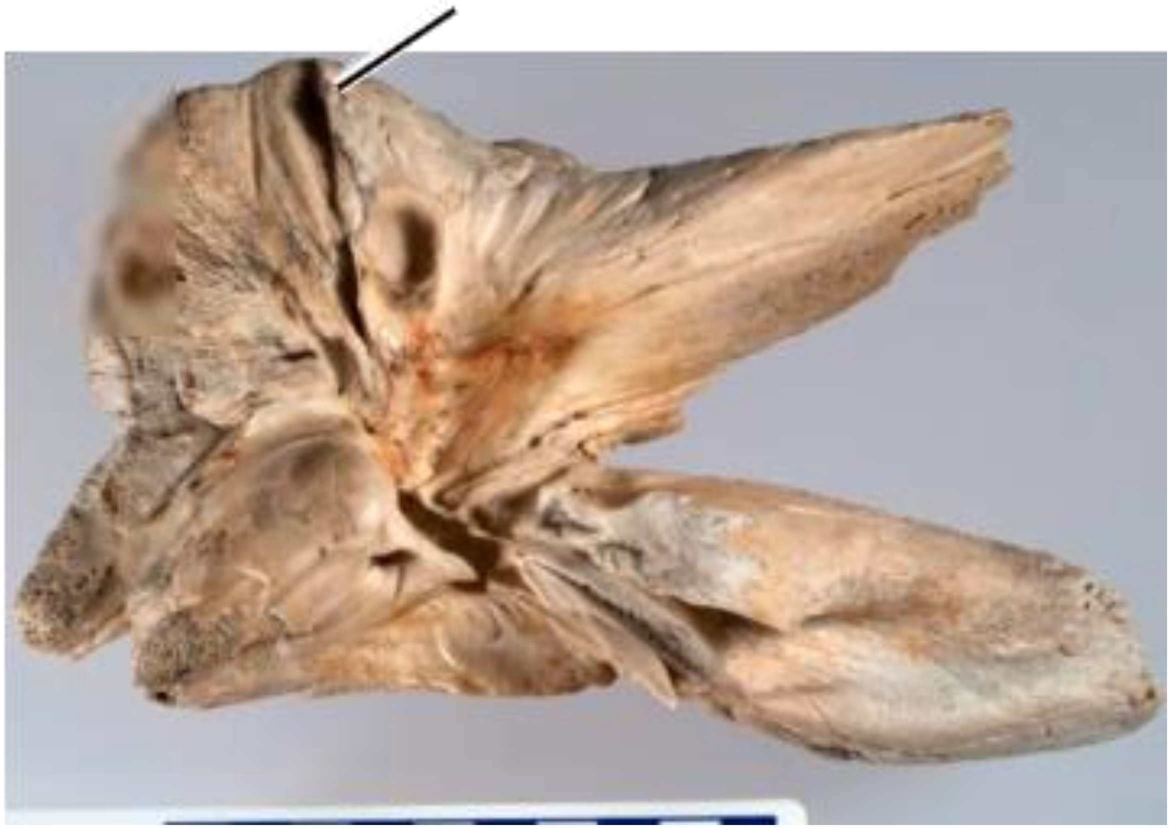

Eubalaena spp. (mirrored)

Copyright holder: Felix G. Marx/ Museum of Geology, University of Otago, Dunedin, New Zealand

(1)

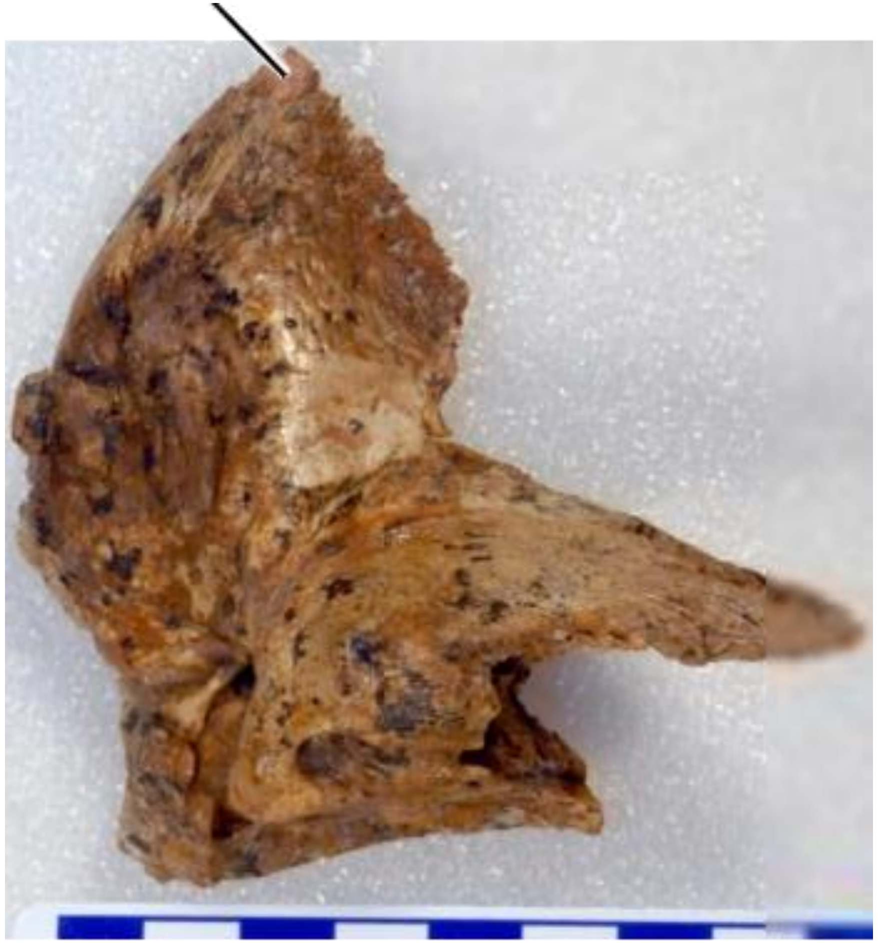

Megaptera miocaena

Copyright holder: Felix G. Marx/United States National Museum of Natural History, Washington DC, USA

[148] 'Ventral edge of anterior process of periotic in medial view'

(0)'at the same level or dorsal to ventral edge of pars cochlearis'

(1)'ventral to ventral profile of pars cochlearis'

(0)

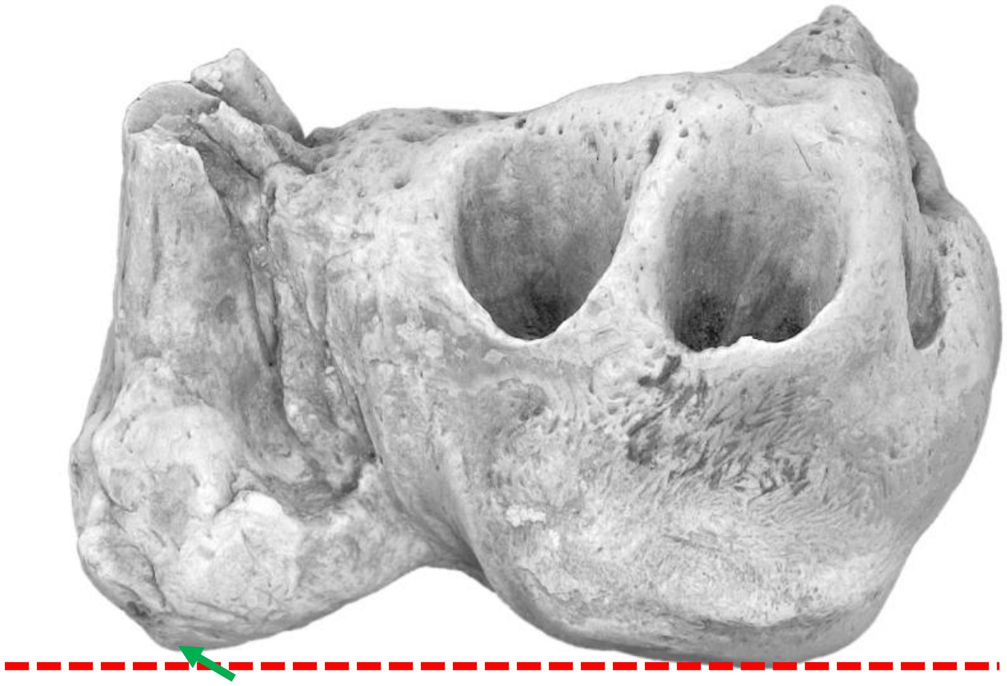

Mammalodon colliveri

Copyright holder: Erich M. G. Fitzgerald/ Museums Victoria, Melbourne, Australia

(1)

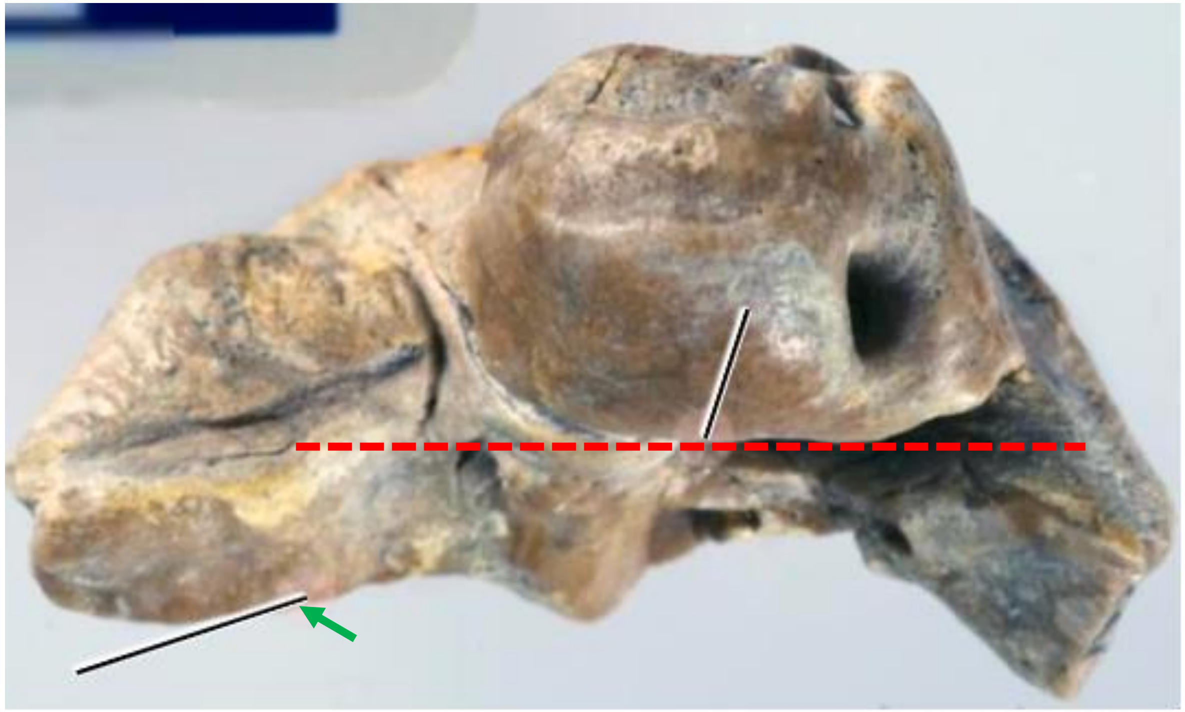

Waipatia maerewhenua

Copyright holder: Felix G. Marx/ University of Otago Museum of Geology, Dunedin, New Zealand

[149] 'Anterior process transversely compressed and blade-like'

(0)'absent’

(1)'present'

(0)

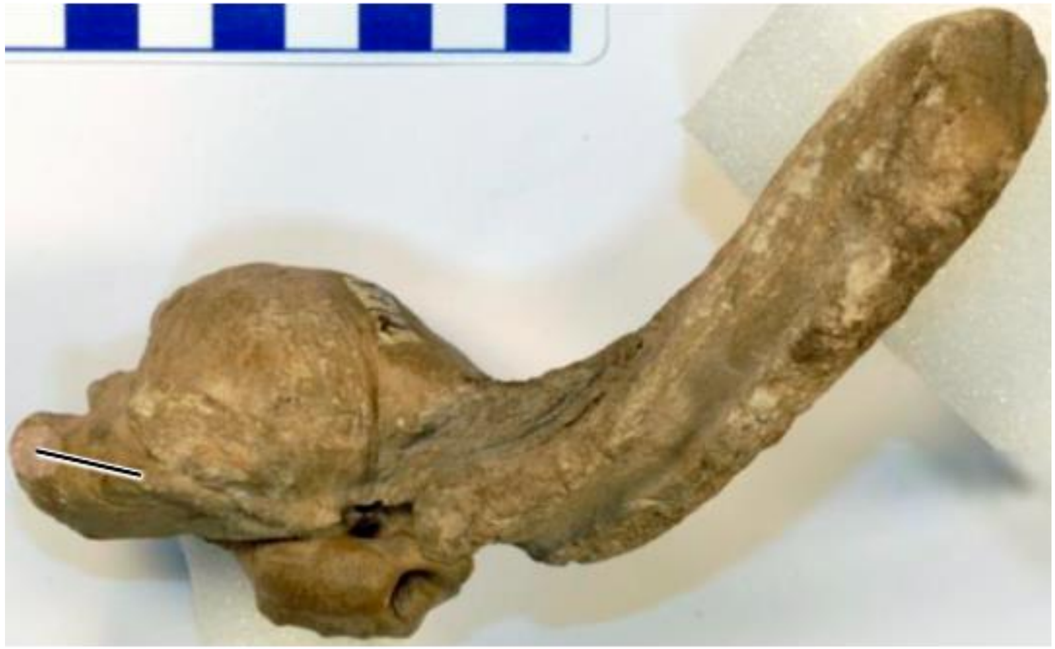

Aglaocetus patulus

Copyright holder: Felix G. Marx/ United States National Museum of Natural History, Washington DC, USA

(1)

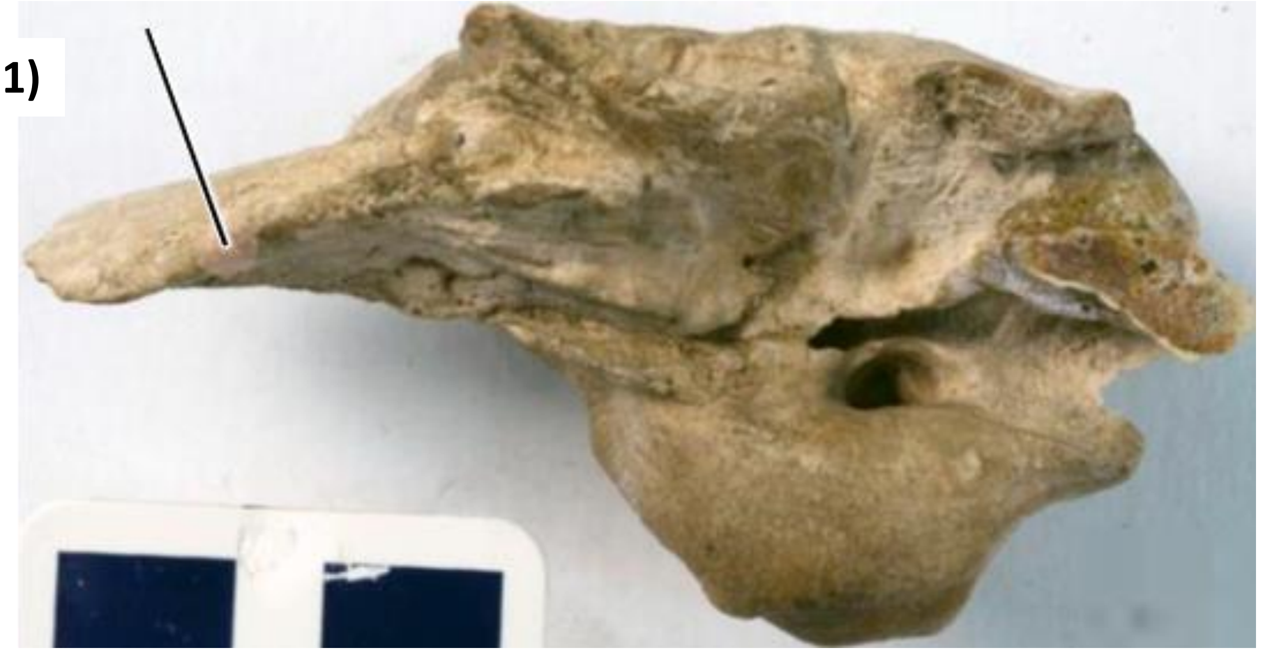

Herpetocetus transatlanticus

Copyright holder: Felix G. Marx/United States National Museum of Natural History, Washington DC, USA

[150] 'Length of anterior process of periotic'

(0)'shorter than the anteroposterior length of the pars cochlearis, as measured from the anterior border of the pars cochlearis to the medial border of the fenestra cochleae'

(1)'same length or longer'

(0)

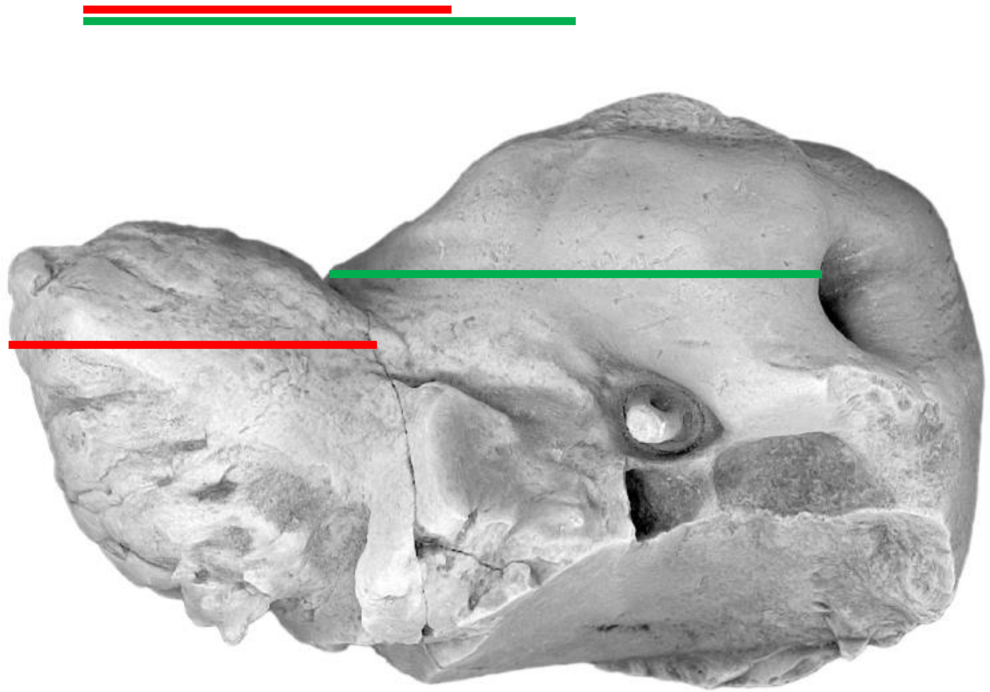

Mammalodon colliveri

Copyright holder: Erich M. G. Fitzgerald/ Museums Victoria, Melbourne, Australia

(1)

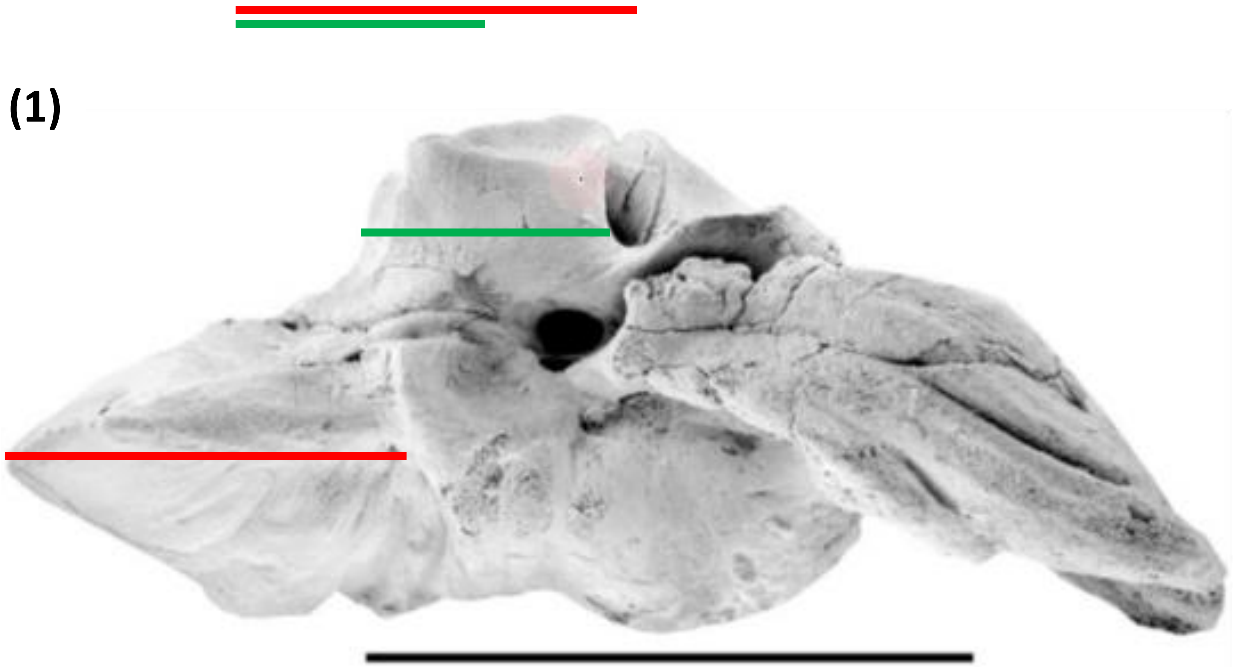

Whakakai waipata (mirrored)

Copyright holder: Cheng-Hsiu Tsai/University of Otago Geology Museum, Dunedin, New Zealand

[151] 'Anteroexternal sulcus'

(0)'forms an oblique or vertical groove on lateral side of anterior process, immediately anterior to lateral tuberosity'

(1)'absent'

(0)

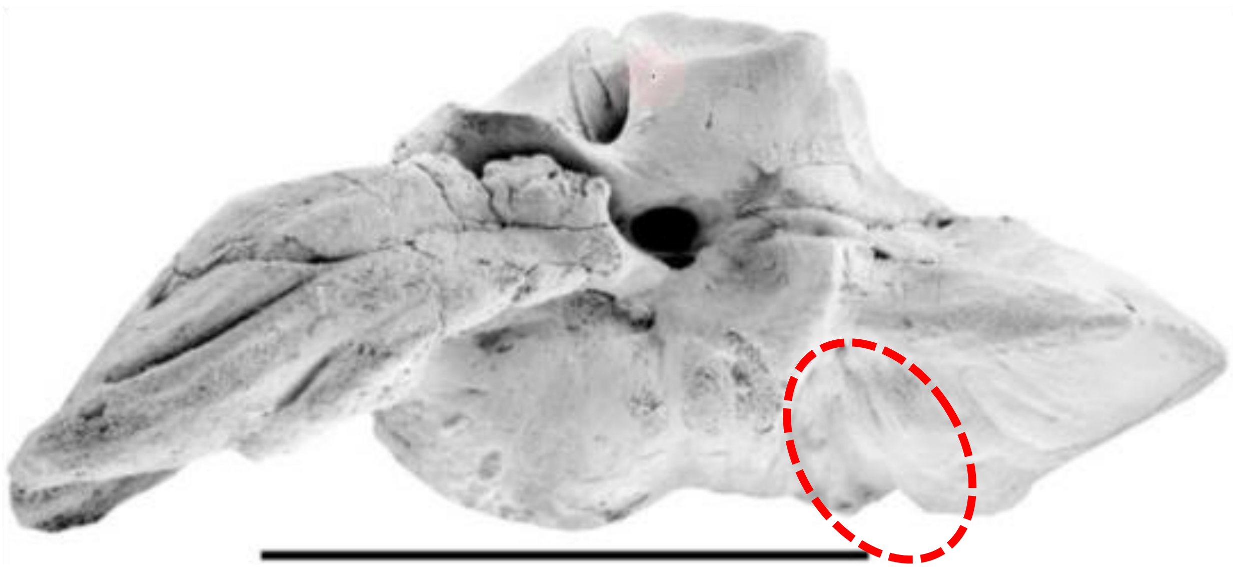

Whakakai waipata

Copyright holder: Cheng-Hsiu Tsai/University of Otago Geology Museum, Dunedin, New Zealand

(1)

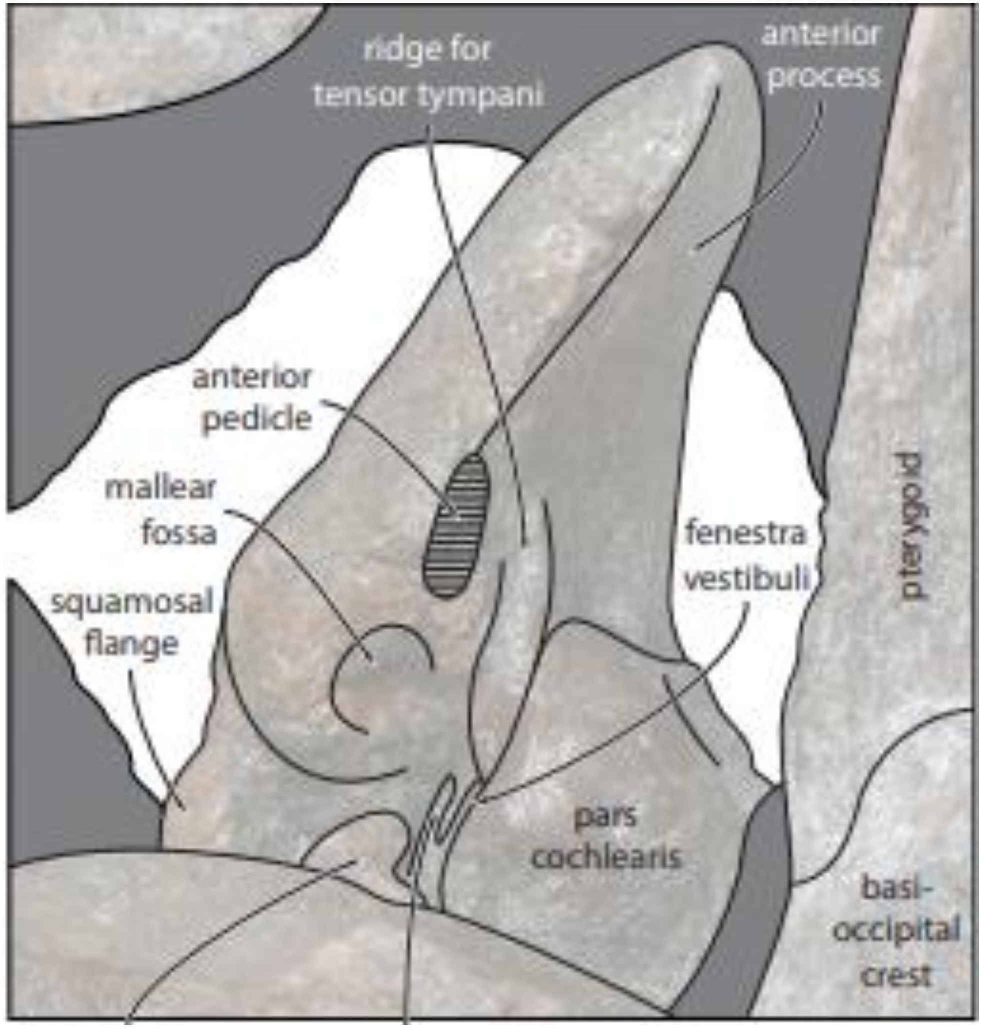

Tranatocetus maregermanicum

Adapted from: "A large Late Miocene cetotheriid (Cetacea, Mysticeti) from the Netherlands clarifies the status of Tranatocetidae."Marx, et al. 2019. *PeerJ* 7: e6426.

[152] 'Pyramidal process'

(0) 'absent'

(1) 'present'

(0)

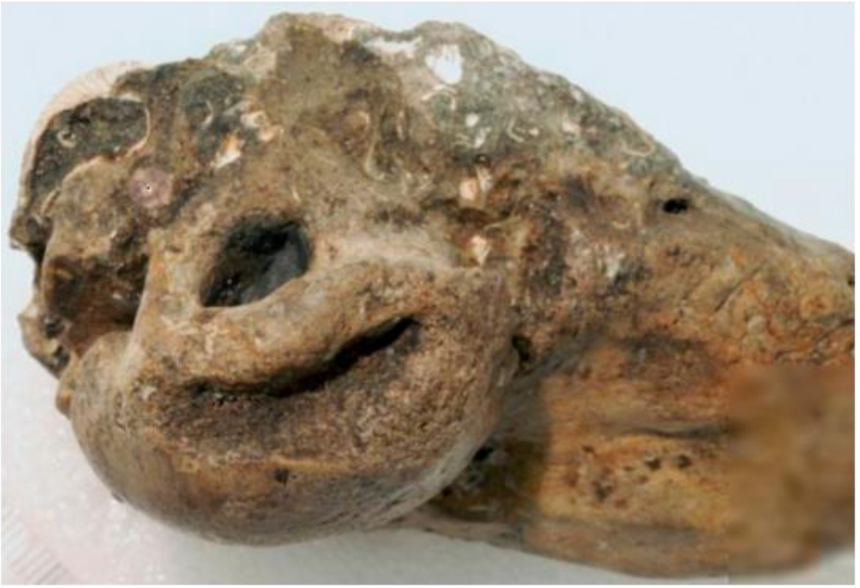

(1)

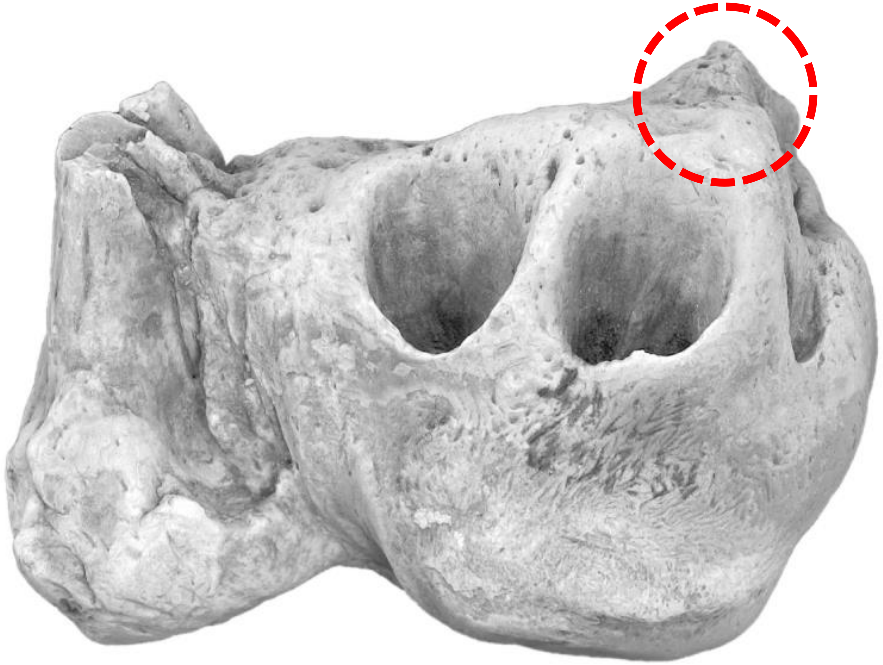

Diunatans luctoretemergo

Copyright holder: Felix G. Marx/ Natuurhistorische collectie van het Zeeuws Genootschap der Wetenschappen, Middelburg, the Netherlands

Mammalodon colliveri

Copyright holder: Erich M. G. Fitzgerald/ Museums Victoria, Melbourne, Australia

[153] 'Articulation of anterior process of periotic and tympanic bulla'

(0)'accessory ossicle of tympanic bulla contacts fovea epitubaria on the anterior process of the periotic'

(1)'accessory ossicle fused to periotic but still clearly defined anteriorly'

(2)'accessory ossicle or homologous region fused to periotic'

(0)

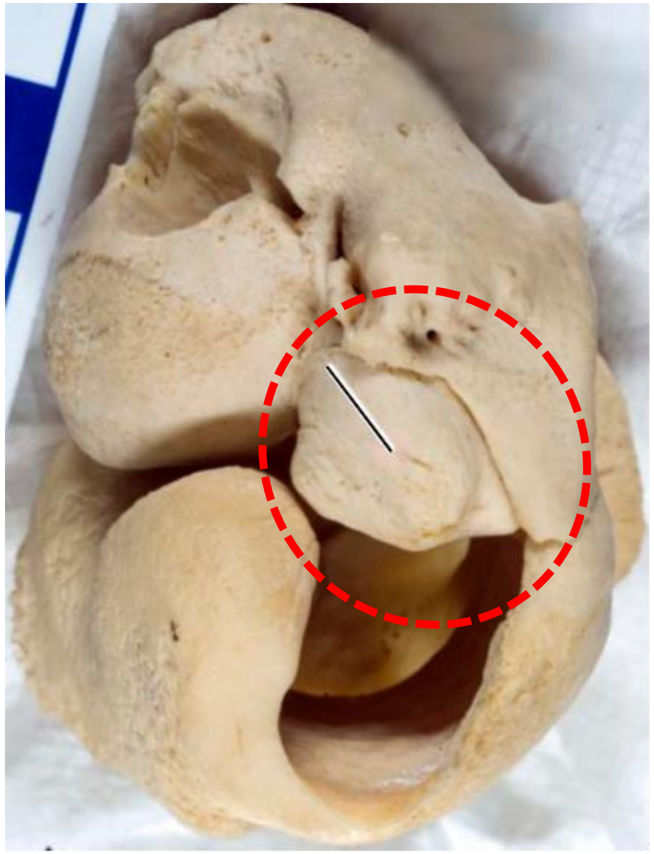

Physeter macrocephalus

Copyright holder: Felix G. Marx, National Museum of Nature and Science, Tokyo, Japan

(1)

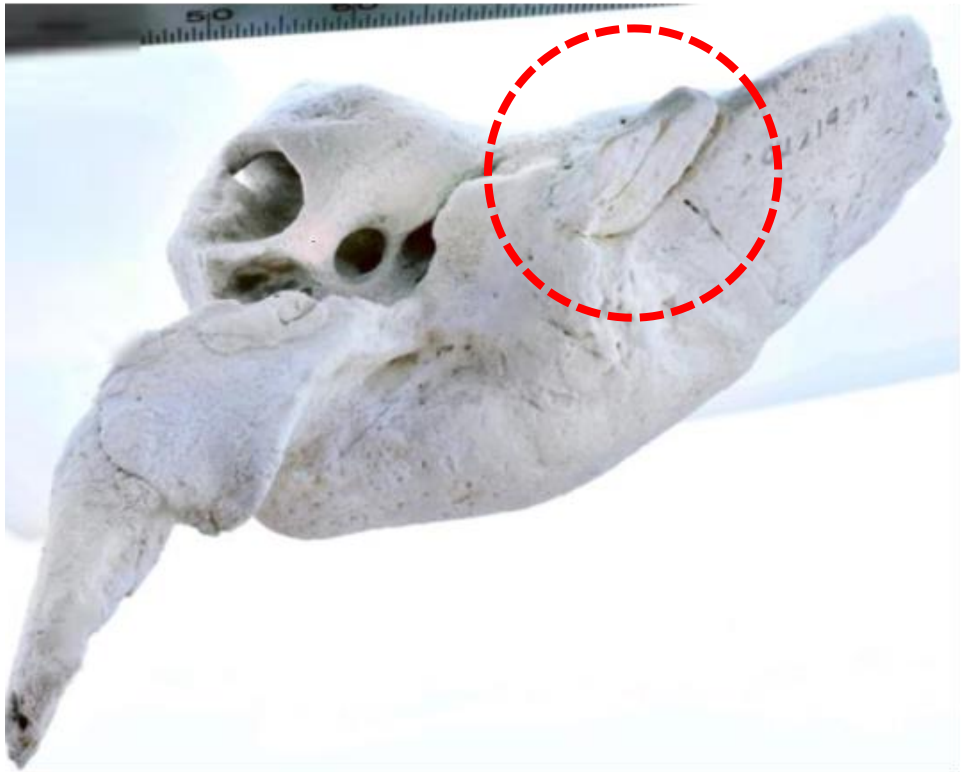

Horopeta umarere

Copyright holder: Cheng-Hsiu Tsai/ Otago University Geology Museum, Dunedin, New Zealand

[153] 'Articulation of anterior process of periotic and tympanic bulla'

(0) 'accessory ossicle of tympanic bulla contacts fovea epitubaria on the anterior process of the periotic'

(1) 'accessory ossicle fused to periotic but still clearly defined anteriorly'

(2) 'accessory ossicle or homologous region fused to periotic'

(2)

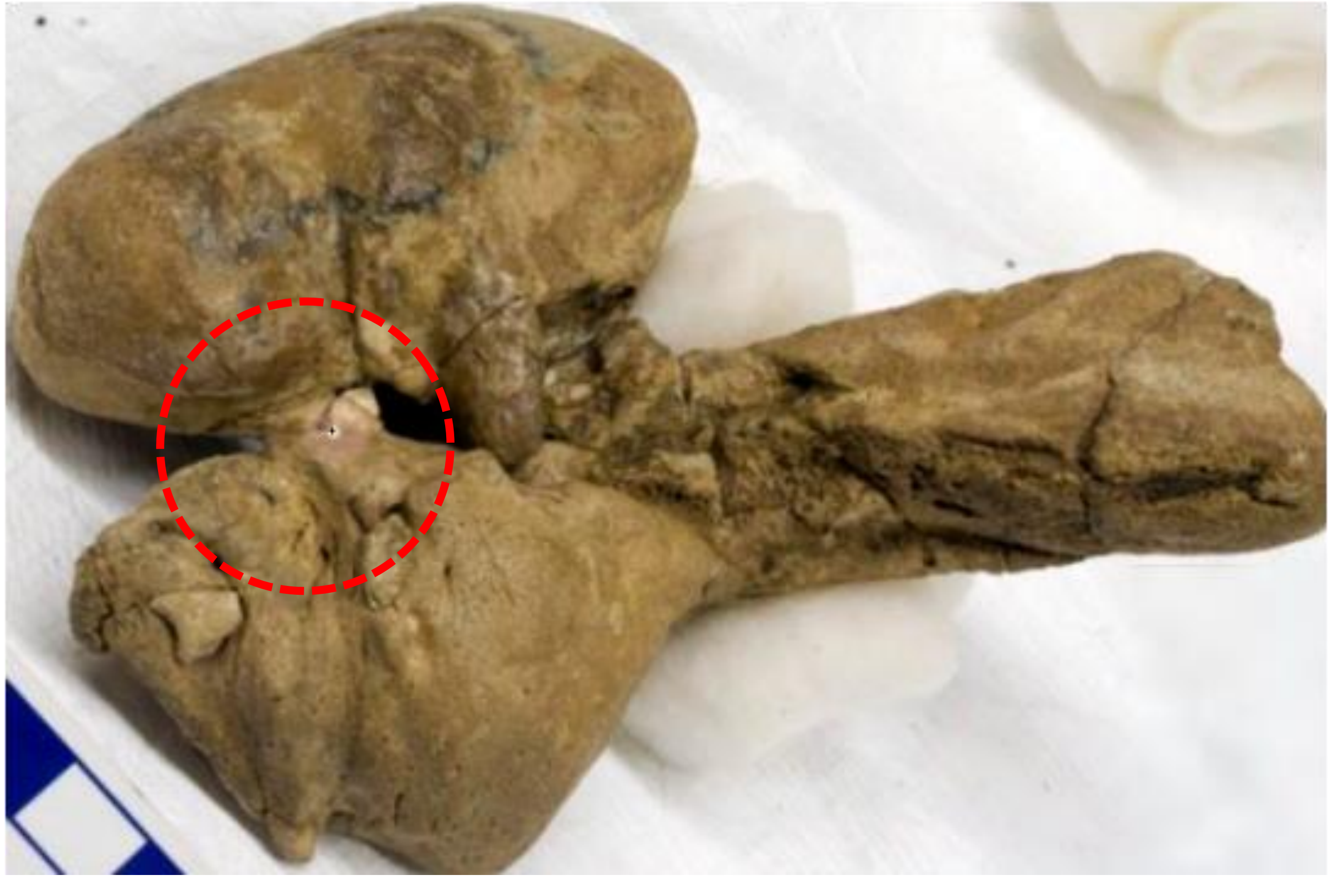

Isanacetus laticephalus

Copyright holder: Felix G. Marx/ Mizunami Fossil Museum, Gifu, Japan

[154] 'Anterior bullar facet'

(0)'well-defined and transversely concave'

(1)'flattened and not clearly distinguishable from fovea epitubaria'

(2)'absent'

(0)

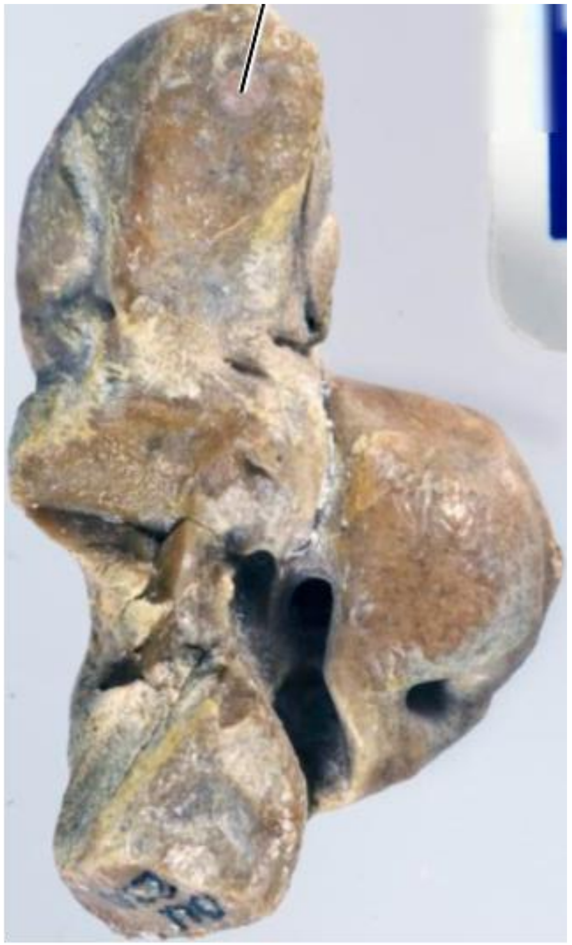

Waipatia maerewhenua

Copyright holder: Felix G. Marx/ University of Otago Museum of Geology, Dunedin, New Zealand

(1)

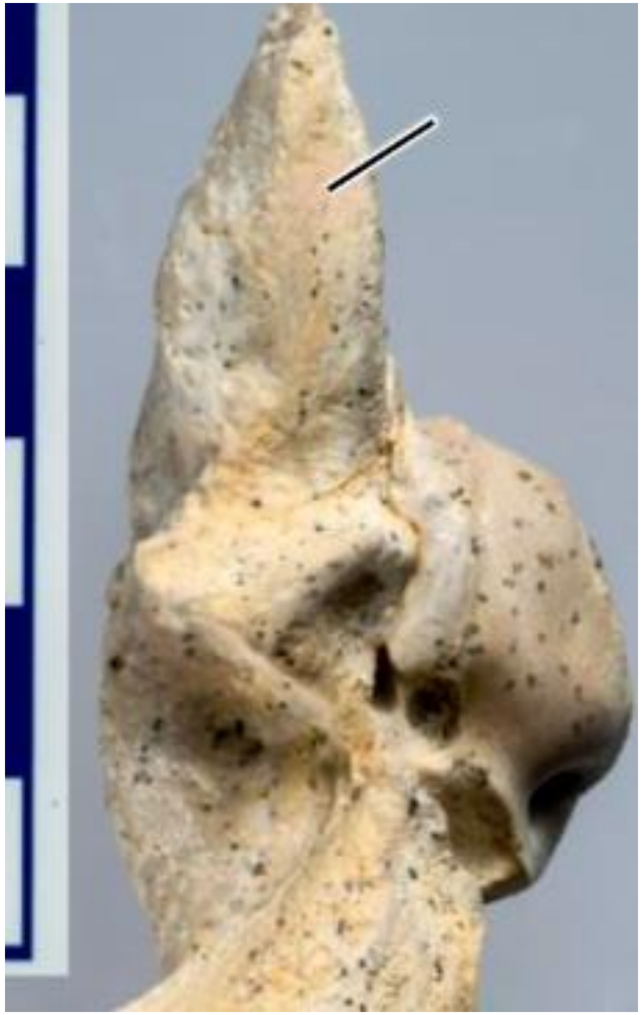

Tokarahia kauaeroa

Copyright holder: Felix G. Marx/ University of Otago Geology Museum, Dunedin, New Zealand

(2)

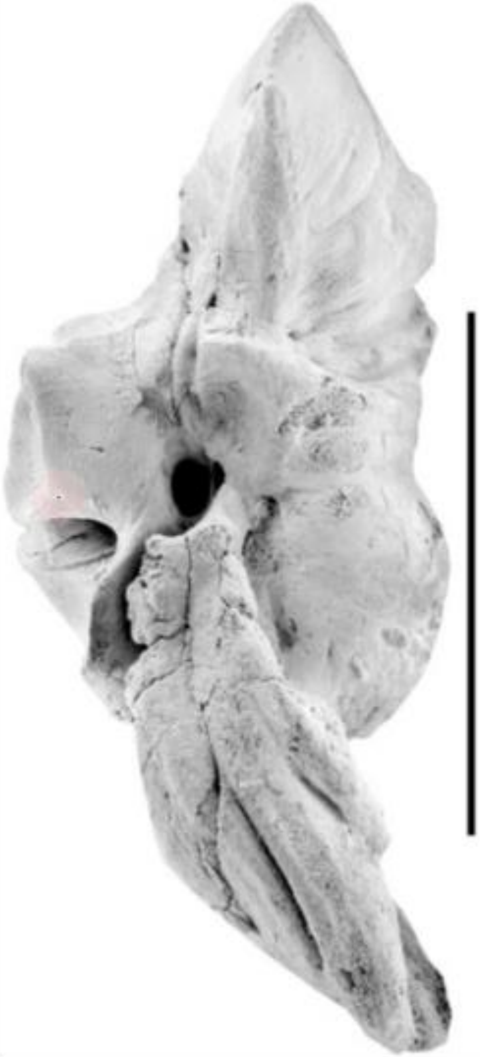

Whakakai waipata

Copyright holder: Cheng-Hsiu Tsai/University of Otago Geology Museum, Dunedin, New Zealand

[155] 'Shape of lateral tuberosity'

(0) 'absent or poorly developed'

(1) 'well-defined tubercle'

(2) 'broadly triangular and longer than wide'

(3) 'hypertrophied'

(4) 'forms a distinct shelf'

(0)

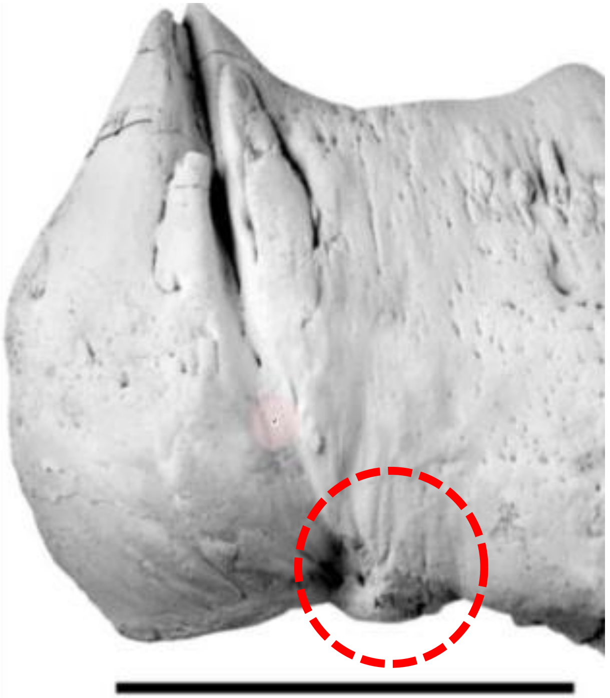

Whakakai waipata

Copyright holder: Cheng-Hsiu Tsai/University of Otago Geology Museum, Dunedin, New Zealand

(1)

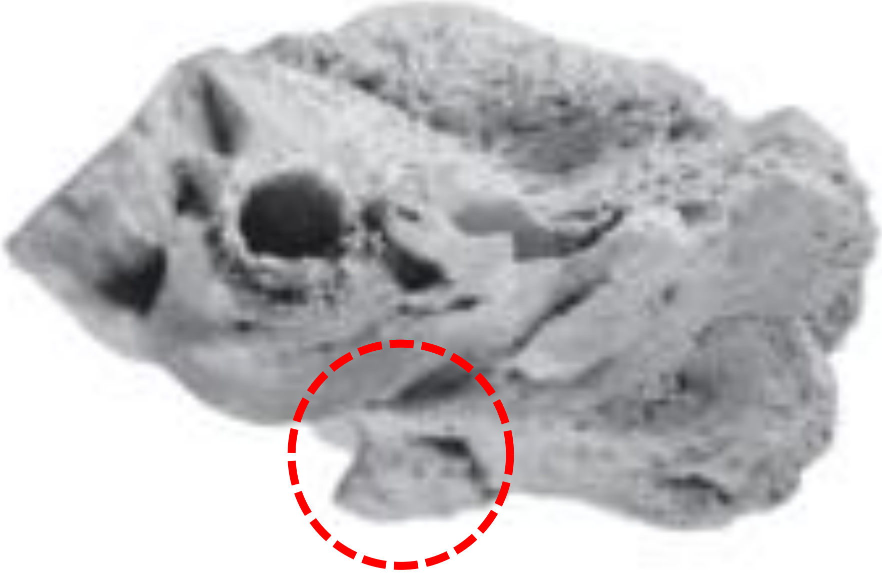

Piscobalaena nana

Adapted from: "The anatomy and relationships of *Piscobalaena nana* (Cetacea, Mysticeti), a Cetotheriidae s.s. from the early Pliocene of Peru", Bouetel and Muizon, 2006. *Geodiversitas* 28.2 (2006): 319-395.

(2)

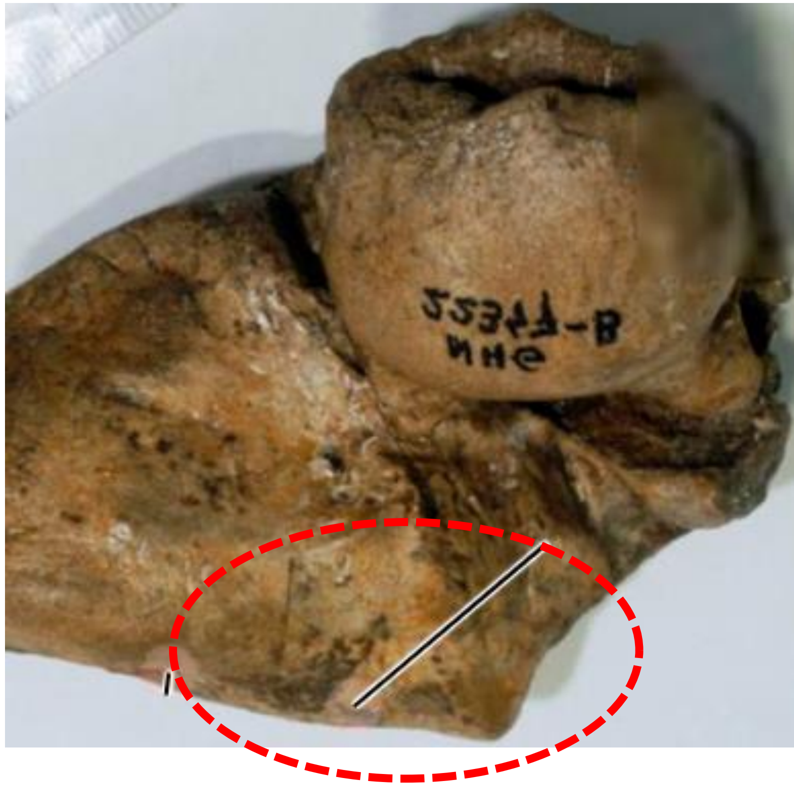

Diunatans luctoretemergo

Copyright holder: Felix G. Marx/ Natuurhistorische collectie van het Zeeuws Genootschap der Wetenschappen, Middelburg, the Netherlands

[155] 'Shape of lateral tuberosity'

(0) 'absent or poorly developed'

(1) 'well-defined tubercle'

(2) 'broadly triangular and longer than wide'

(3) 'hypertrophied'

(4) 'forms a distinct shelf'

(3)

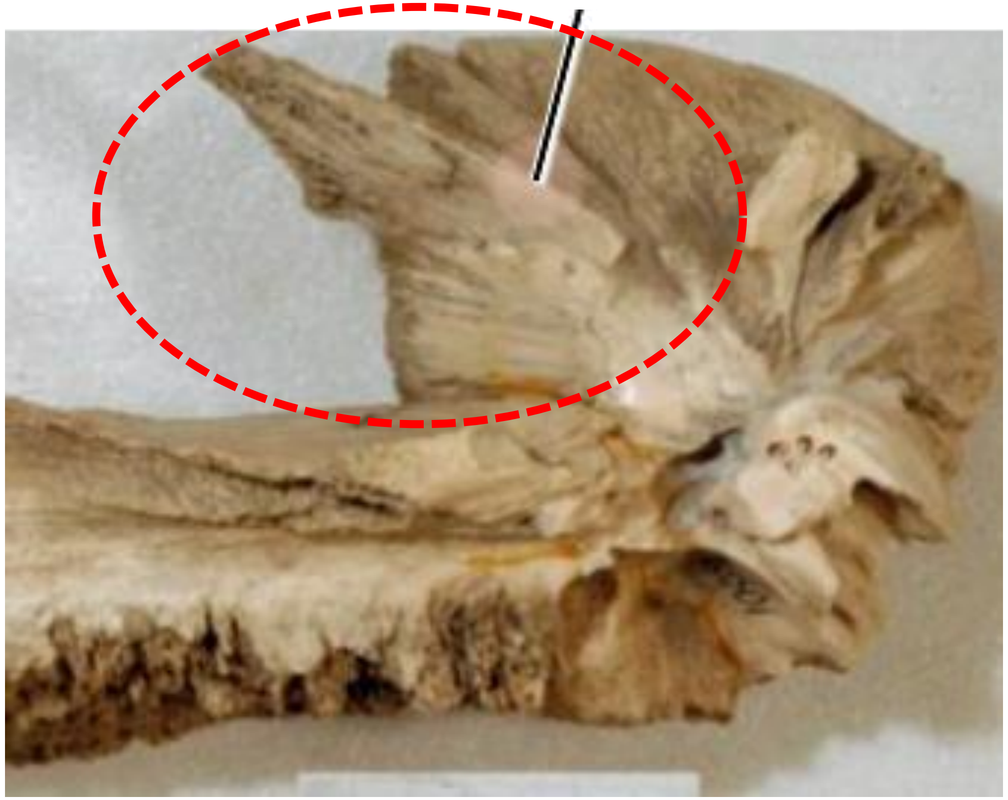

Balaena mysticetus

Copyright holder: Felix G. Marx/United States National Museum of Natural History, Washington DC, USA

(4)

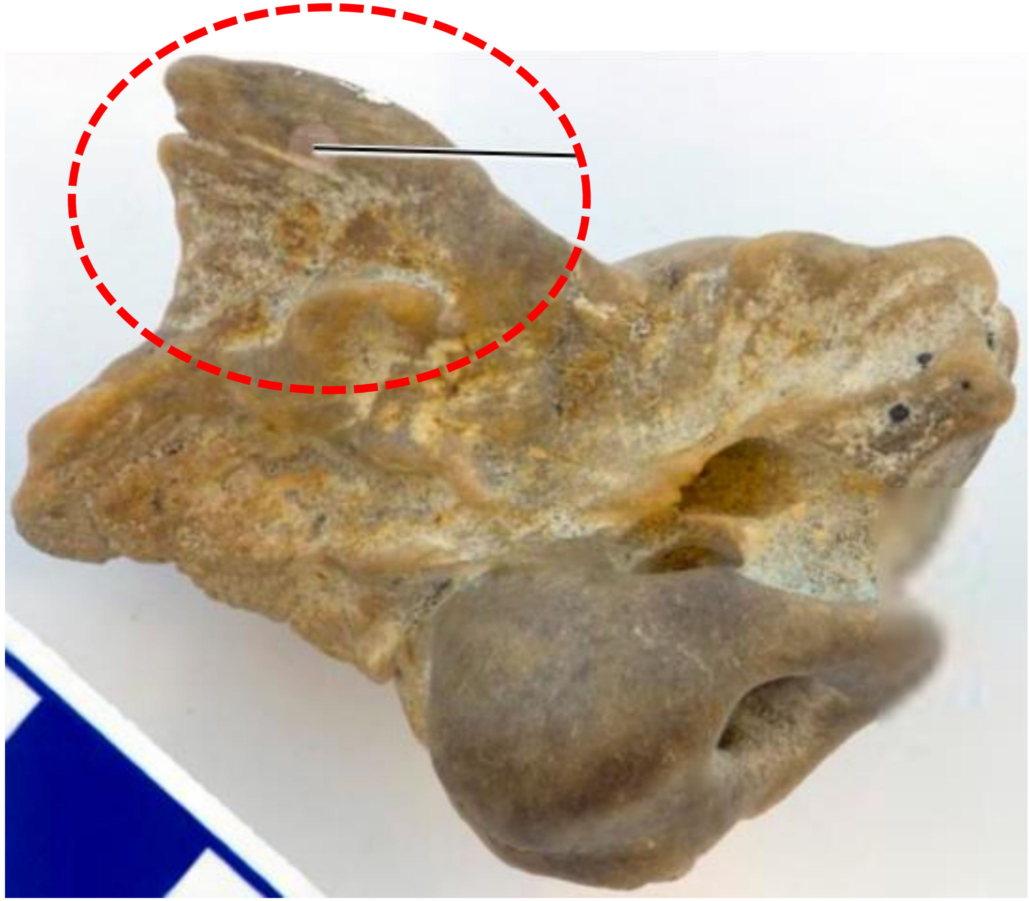

Herpetocetus morrowi

Copyright holder: Felix G. Marx/ San Diego Museum of Natural History, San Diego, USA

[156] 'Blade-like flange of lateral tuberosity'

(0) 'absent'

(1) 'present'

(0)

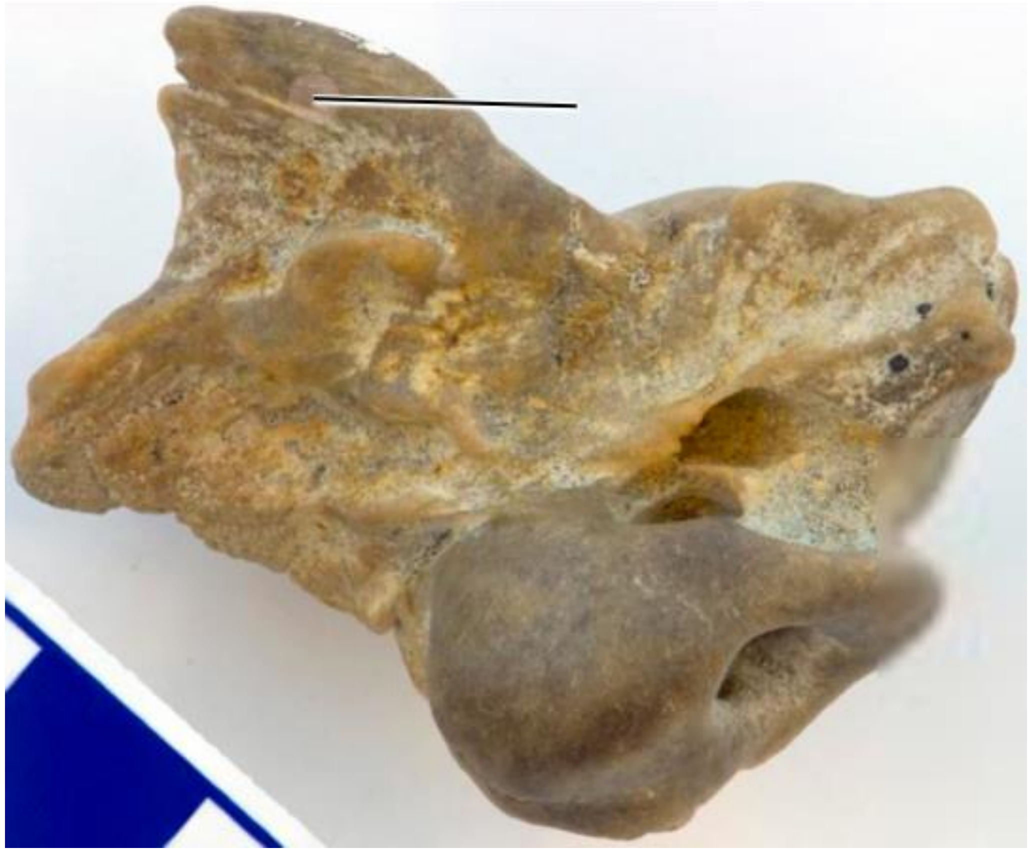

Herpetocetus morrowi

Copyright holder: Felix G. Marx/ San Diego Museum of Natural History, San Diego, USA

(1)

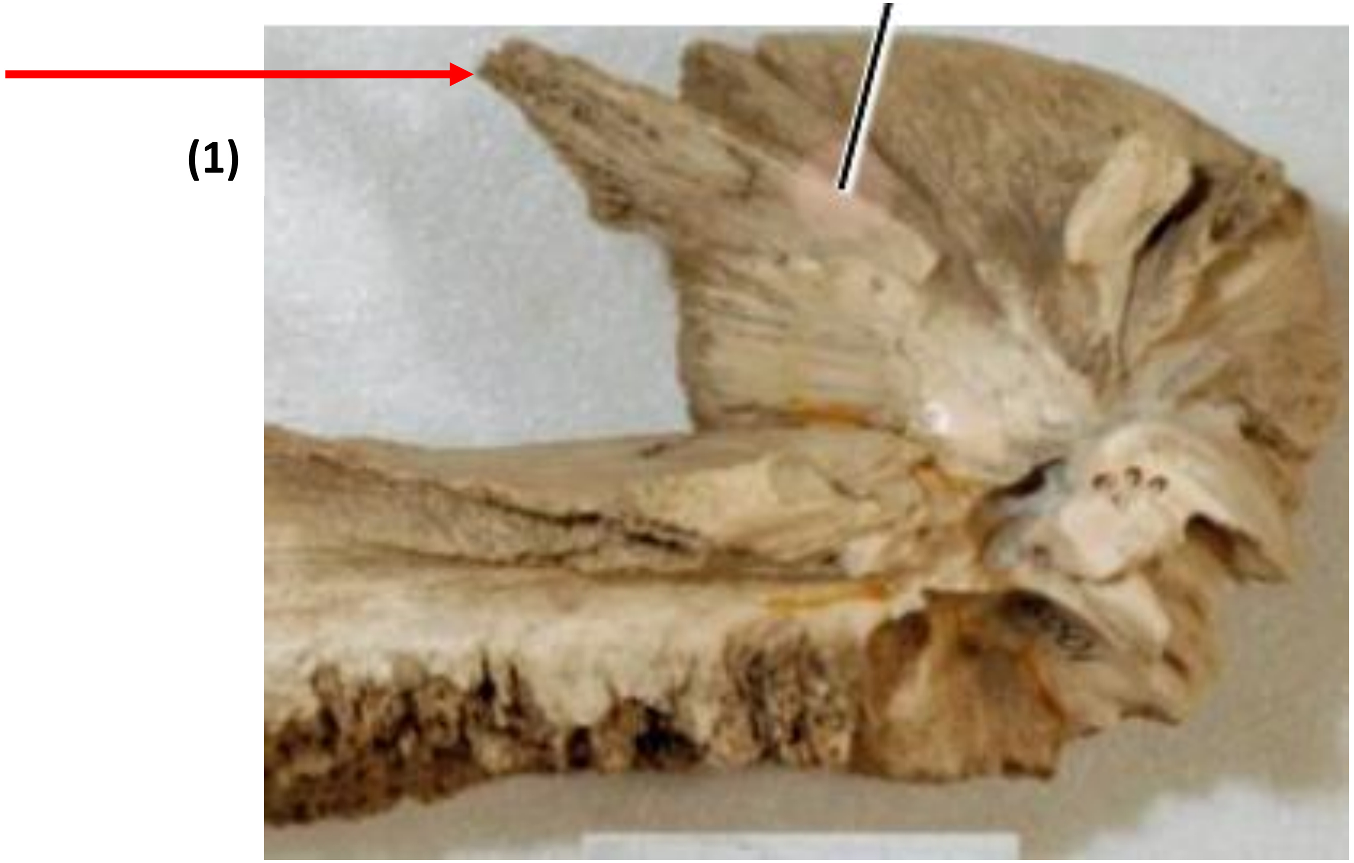

Balaena mysticetus

Copyright holder: Felix G. Marx/United States National Museum of Natural History, Washington DC, USA

[157] 'Position of lateral tuberosity'

(0)'situated posterolateral to anterior pedicle of tympanic bulla or fovea epitubaria’

(1)'situated lateral or anterolateral to anterior pedicle of tympanic bulla or fovea epitubaria

(0)

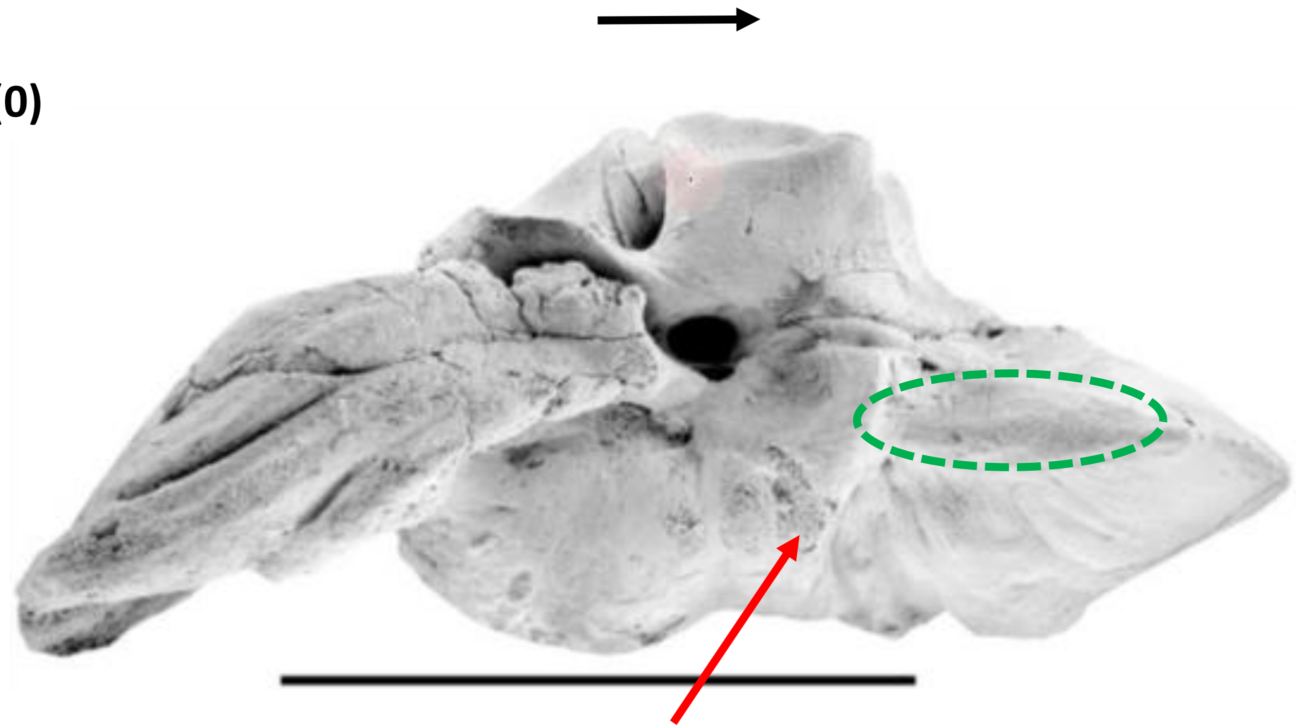

Whakakai waipata

Copyright holder: Cheng-Hsiu Tsai/University of Otago Geology Museum, Dunedin, New Zealand

(1)

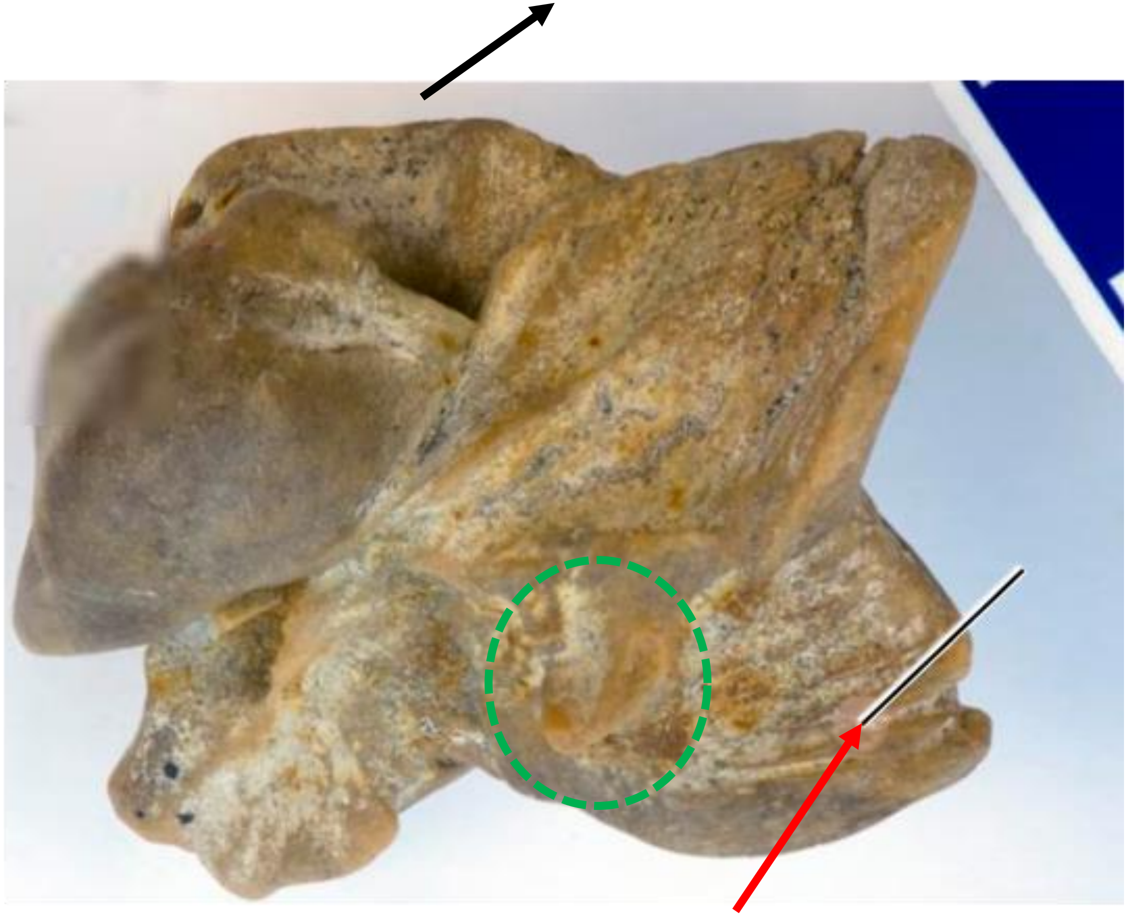

Herpetocetus morrowi

Copyright holder: Felix G. Marx/ San Diego Museum of Natural History, San Diego, USA

[158] 'Body of periotic lateral to pars cochlearis hypertrophied'

(0) 'absent'

(1) 'present laterally and ventrally'

(2) 'present laterally only'

(0)

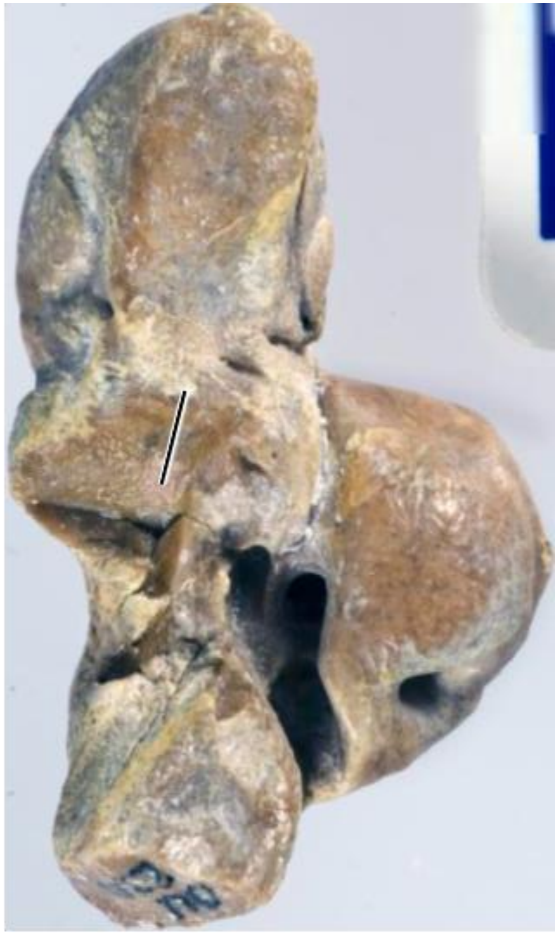

Waipatia maerewhenua

Copyright holder: Felix G. Marx/ University of Otago Museum of Geology, Dunedin, New Zealand

(1)

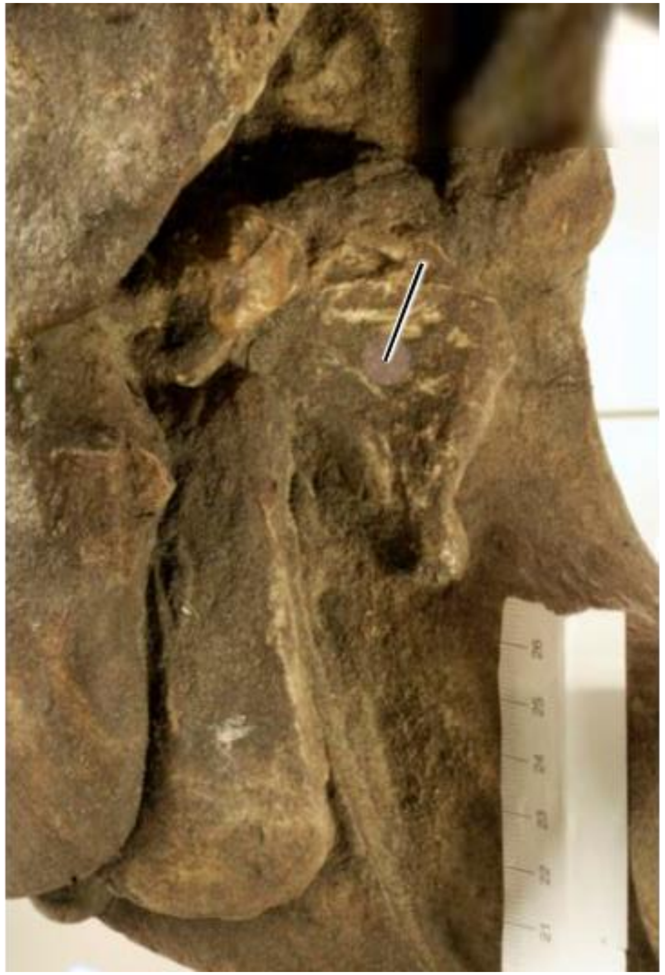

Balaenella brachyrhynus

Copyright holder: Felix G. Marx/Natuurmuseum Brabant, Tilburg, the Netherlands

(2)

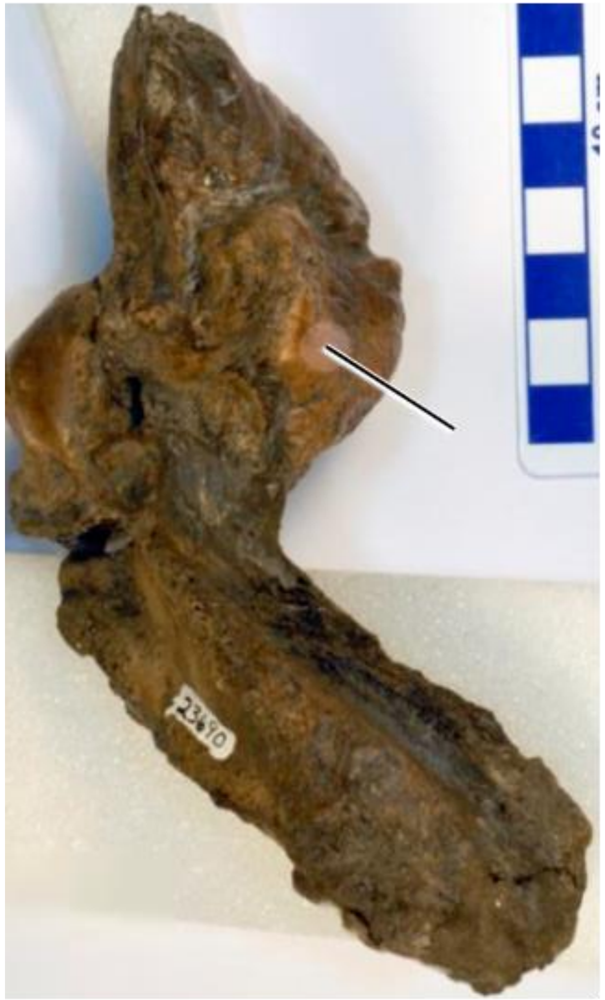

Aglaocetus patulus

Copyright holder: Felix G. Marx/ United States National Museum of Natural History, Washington DC, USA

[159] 'Mallear fossa'

(0) 'well excavated with a clearly defined rim'

(1) 'present only as a depression with diffuse edges'

(0)

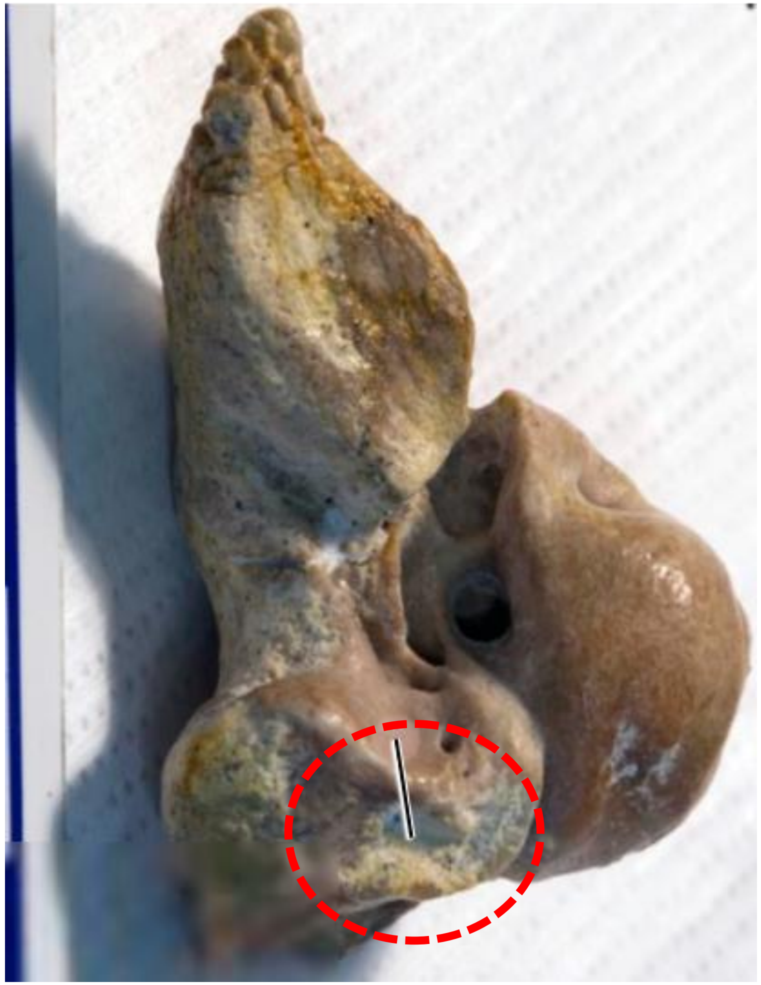

Waipatia maerewhenua

Copyright holder: Felix G. Marx/ University of Otago Museum of Geology, Dunedin, New Zealand

(1)

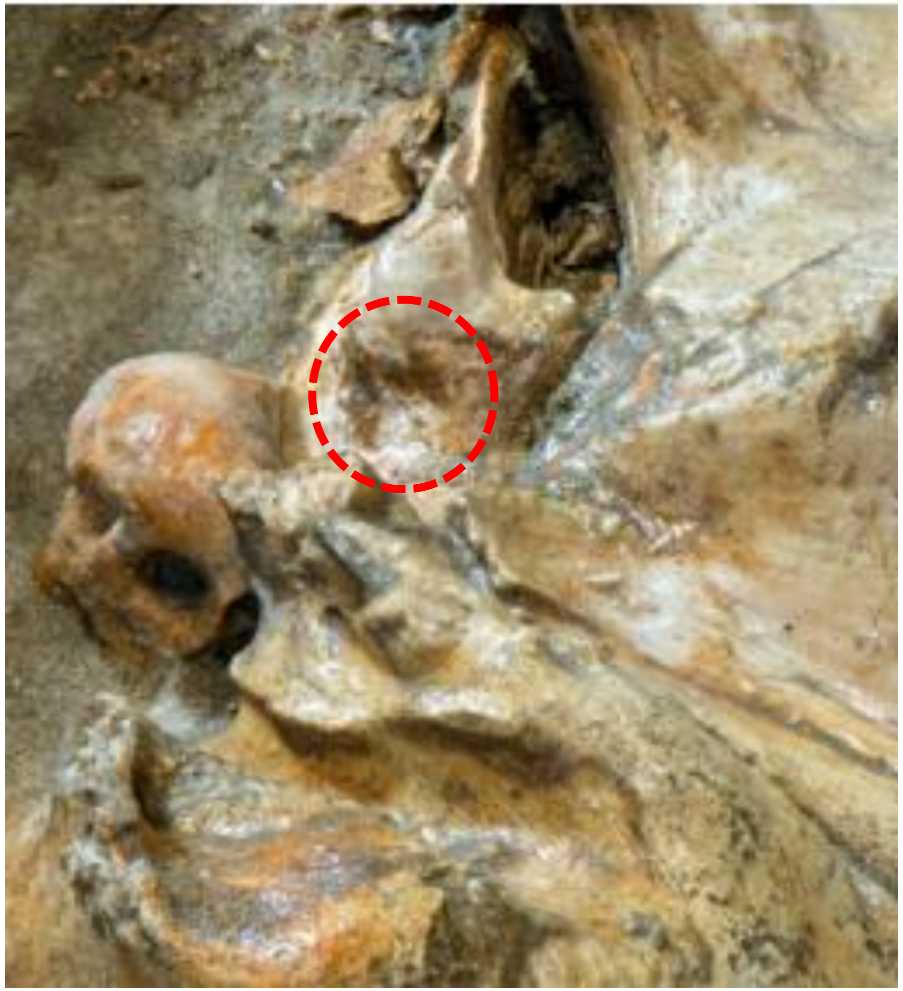

Tiucetus rosae

Adapted from: *"A new Miocene baleen whale from Peru deciphers the dawn of cetotheriids." Marx, Lambert, and De Muizon, 2017. Royal Society Open Science 4.9: 170560.*

[160] 'Distinct ridge delimiting insertion surface of tensor tympani on medial side of anterior process'

(0) 'absent'

(1) 'absent, but insertion surface distinctly excavated'

(2) 'present'

(0)

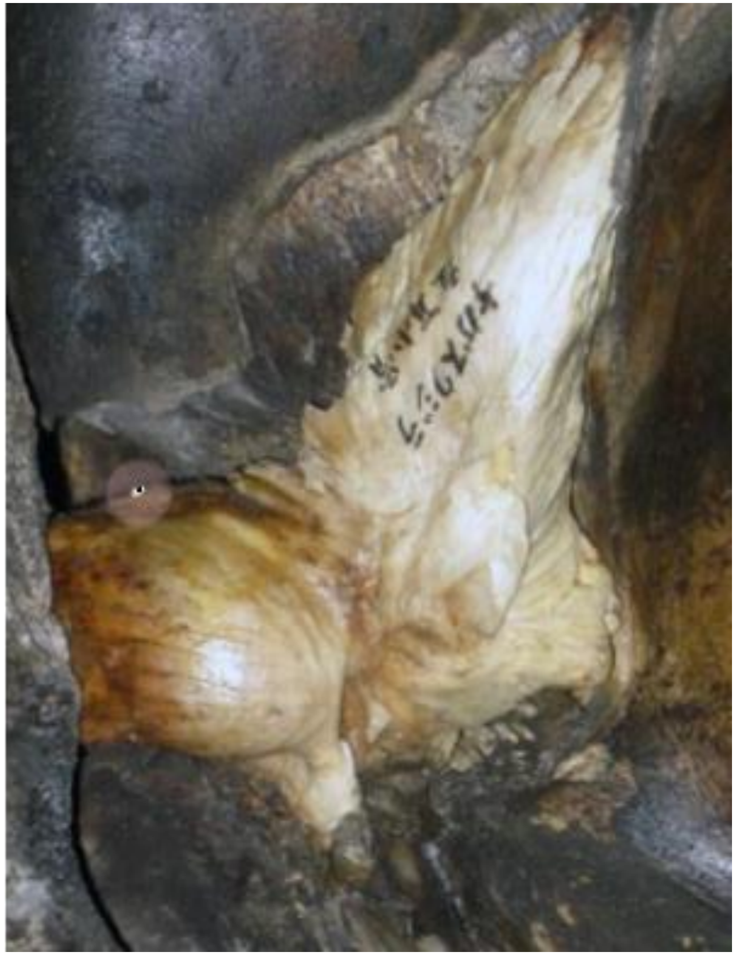

Balaenoptera physalus

Copyright holder: Felix G. Marx/ Osaka Museum of Natural History, Osaka, Japan

(1)

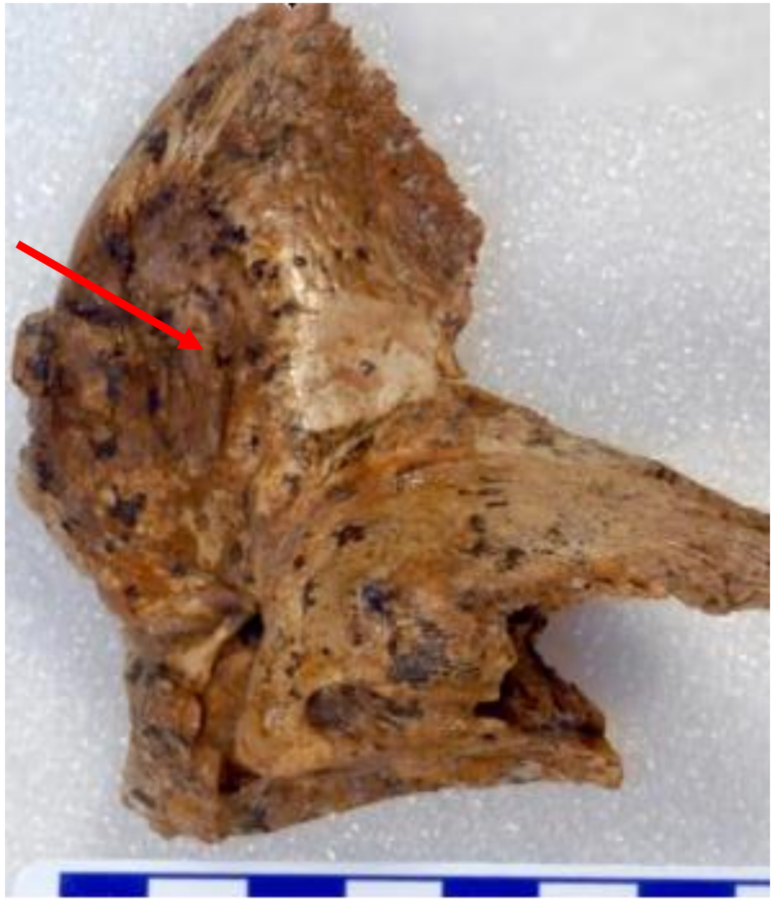

Megaptera miocaena

Copyright holder: Felix G. Marx/United States National Museum of Natural History, Washington DC, USA

(2)

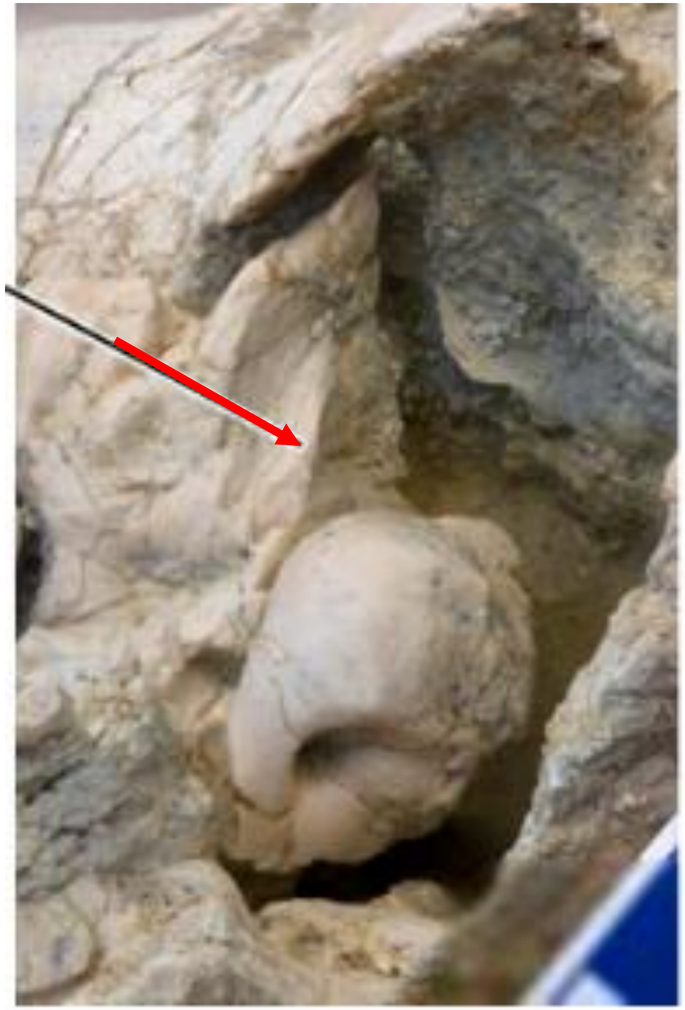

Herpetocetus morrowi

Copyright holder: Felix G. Marx/ University of California Museum of Paleontology, Berkeley, USA

[161] 'Dorsal extension of attachment area for tensor tympani on medial side of anterior process'

(0)'absent or indistinct'

(1)'present as a deeply excavated canal'

(0)

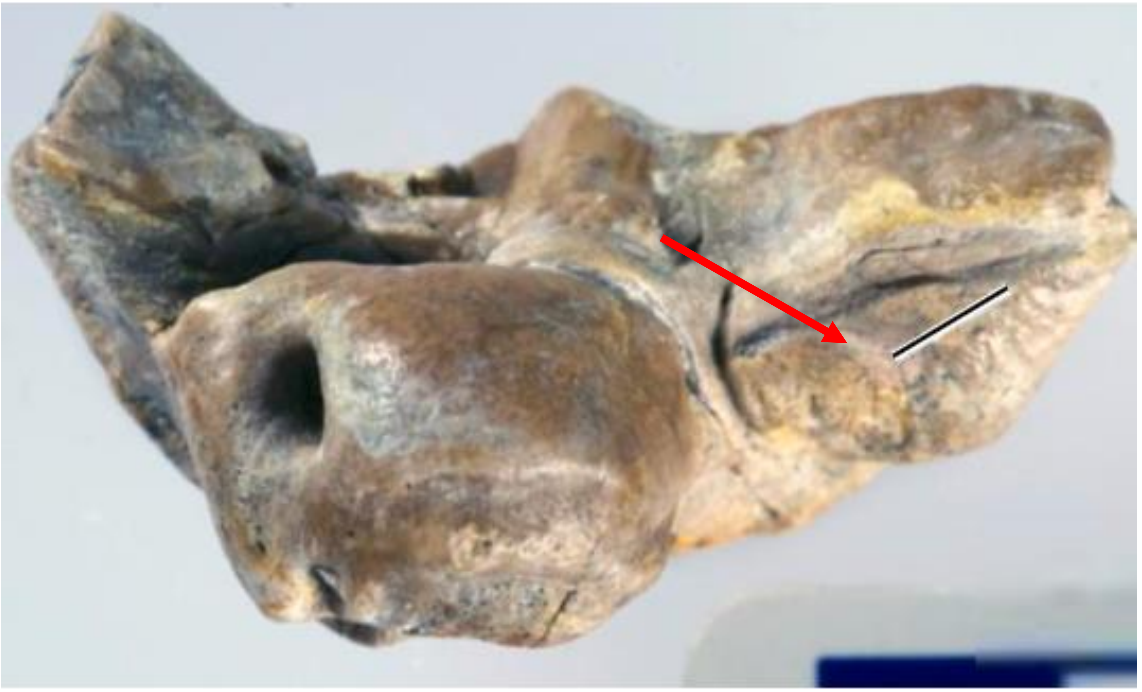

Waipatia maerewhenua

Copyright holder: Felix G. Marx/ University of Otago Museum of Geology, Dunedin, New Zealand

(1)

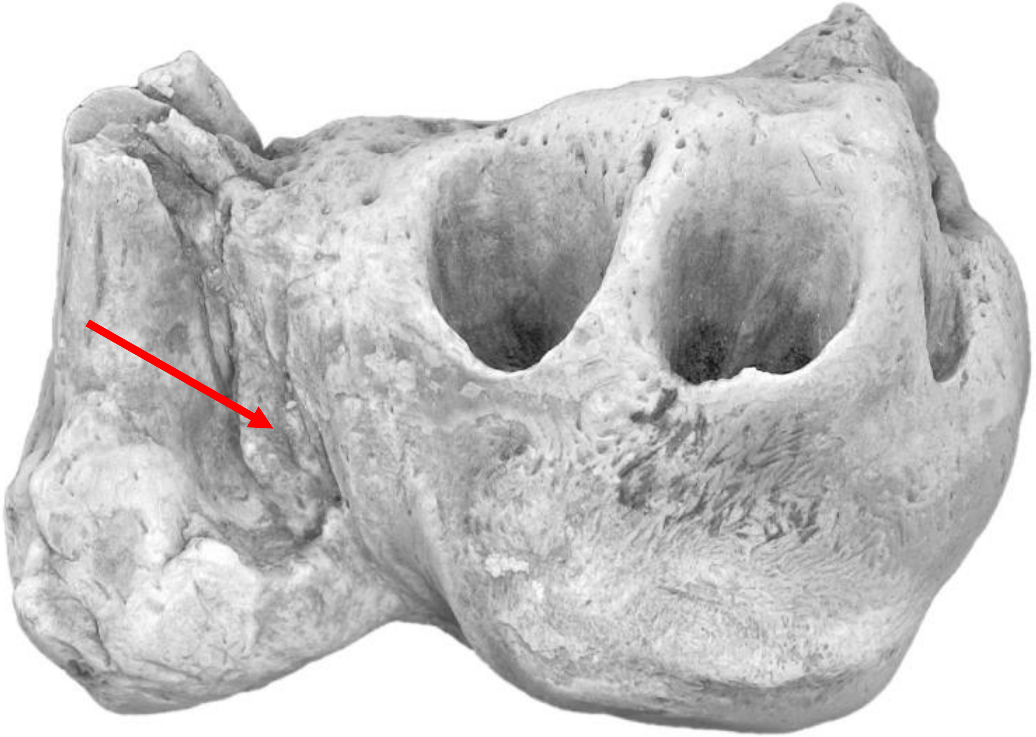

Mammalodon colliveri

Copyright holder: Erich M. G. Fitzgerald/ Museums Victoria, Melbourne, Australia

[162] 'Anteromedial corner of pars cochlearis in ventral view'

(0)'developed as a rounded, anteroposterior ridge'

(1)'angular and projecting medially'

(2)'smooth and rounded'

(0)

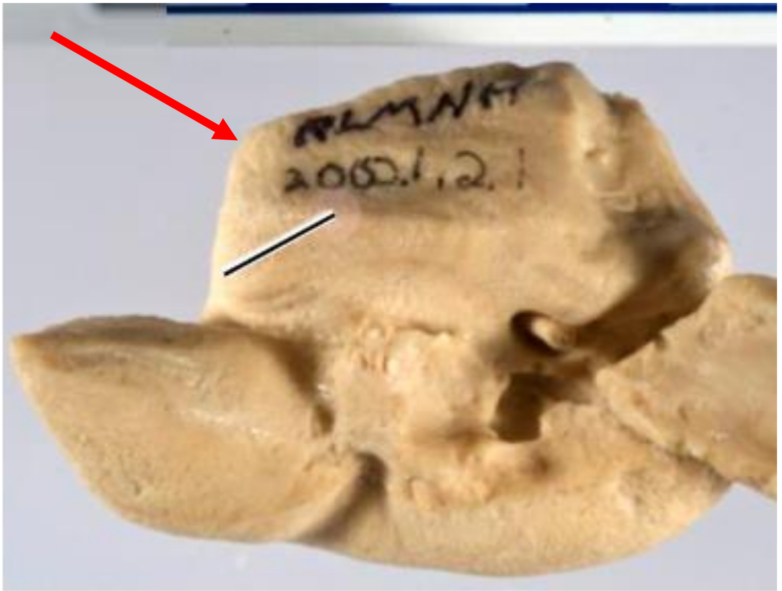

Zygorhiza kochii

Copyright holder: Felix G. Marx/ Alabama Museum of Natural History, Tuscaloosa, Alabama, USA

(1)

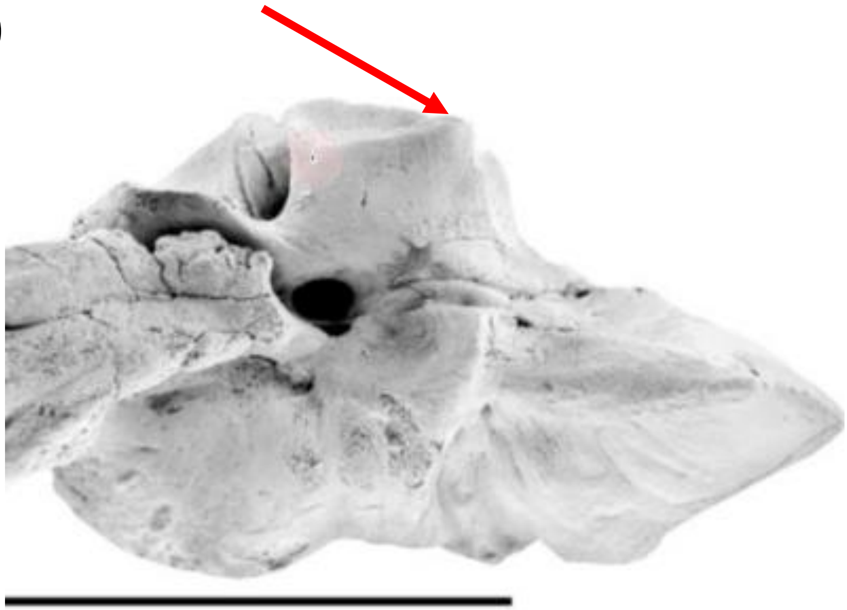

Whakakai waipata

Copyright holder: Cheng-Hsiu Tsai/University of Otago Geology Museum, Dunedin, New Zealand

(2)

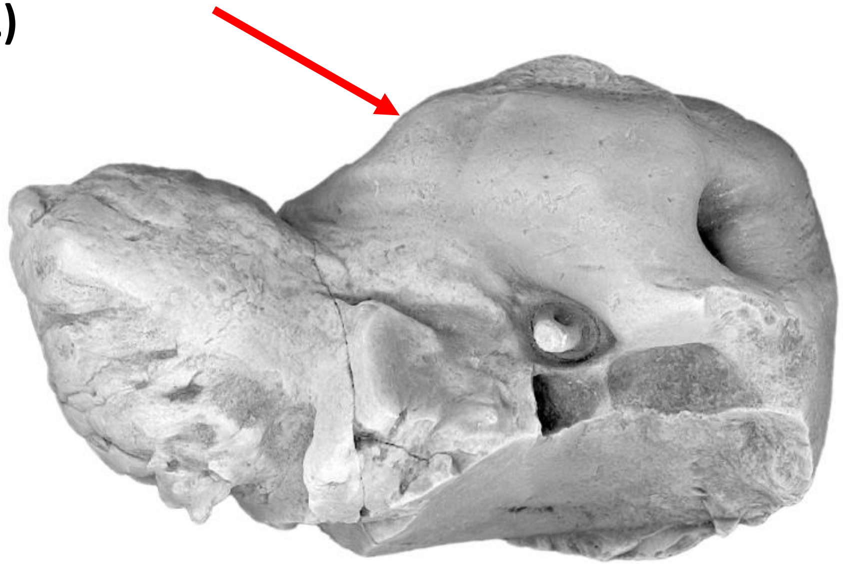

Mammalodon colliveri

Copyright holder: Erich M. G. Fitzgerald/ Museums Victoria, Melbourne, Australia

[163] 'Promontorial groove on medial side of pars cochlearis'

(0)'present, but relatively shallow'

(1)'present and deeply excavated'

(2)'present and forming a distinct constriction, separating a smooth and rounded ventral portion of the pars cochlearis from a flattened and striated dorsal one'

(3)'absent'

(0)

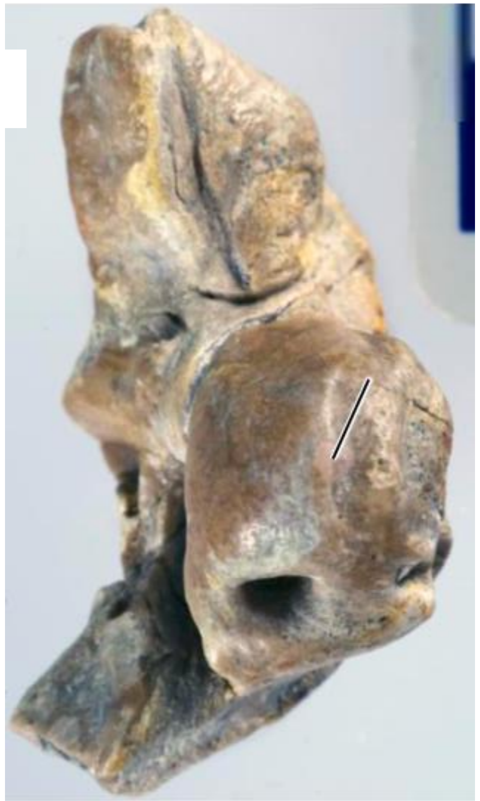

Waipatia maerewhenua

Copyright holder: Felix G. Marx/ University of Otago Museum of Geology, Dunedin, New Zealand

(1)

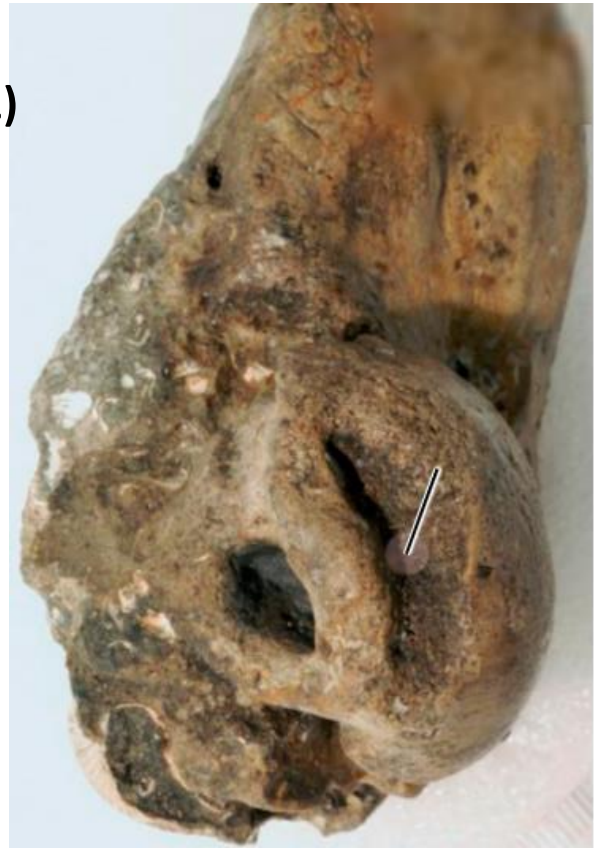

Diunatans luctoretemergo

Copyright holder: Felix G. Marx/ Natuurhistorische collectie van het Zeeuwsch Genootschap der Wetenschappen, Middelburg, the Netherlands

(2)

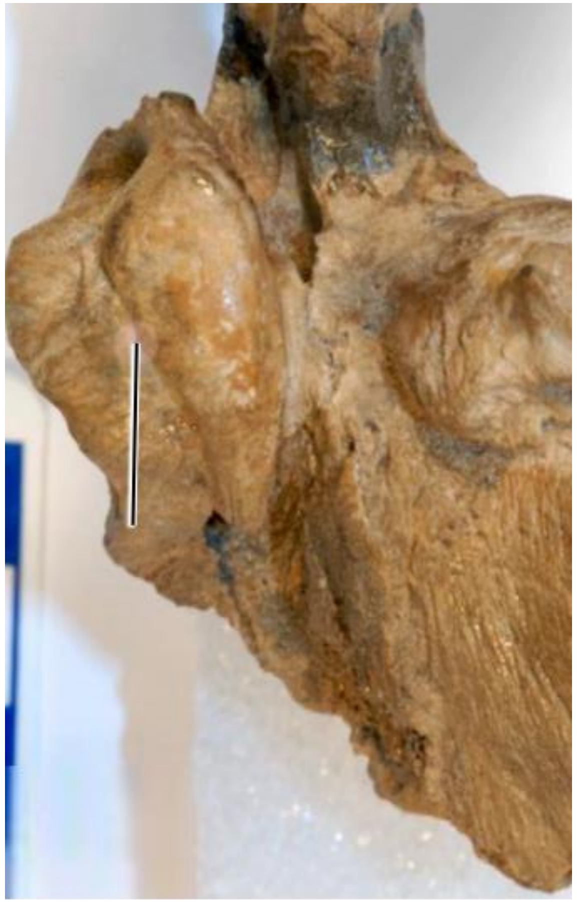

Diorocetus hiatus

Copyright holder: Felix G. Marx/ United States National Museum of Natural History, Washington DC, USA

(3)

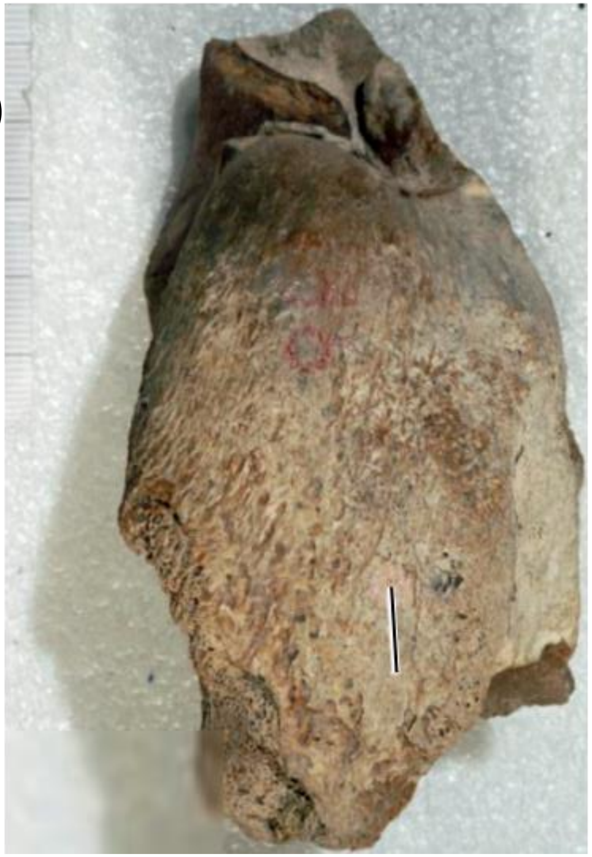

Gricetoides aurorae

Copyright holder: Felix G. Marx/ United States National Museum of Natural History, Washington DC, USA

[164] 'Posterior cochlear crest in posteromedial view'

(0)'well separated from crista parotica’

(1)'narrow separation or contact'

(0)

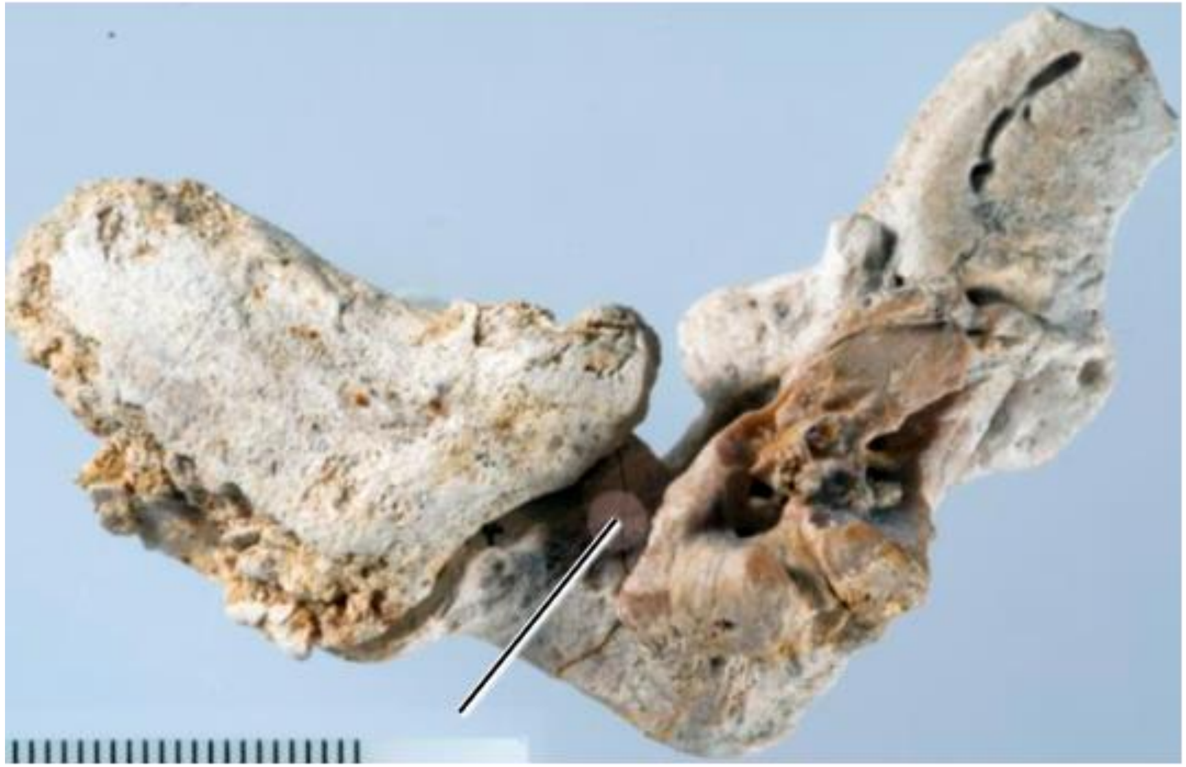

Waharoa ruwhenua

Copyright holder: Felix G. Marx/ University of Otago Geology Museum, Dunedin, New Zealand

(1)

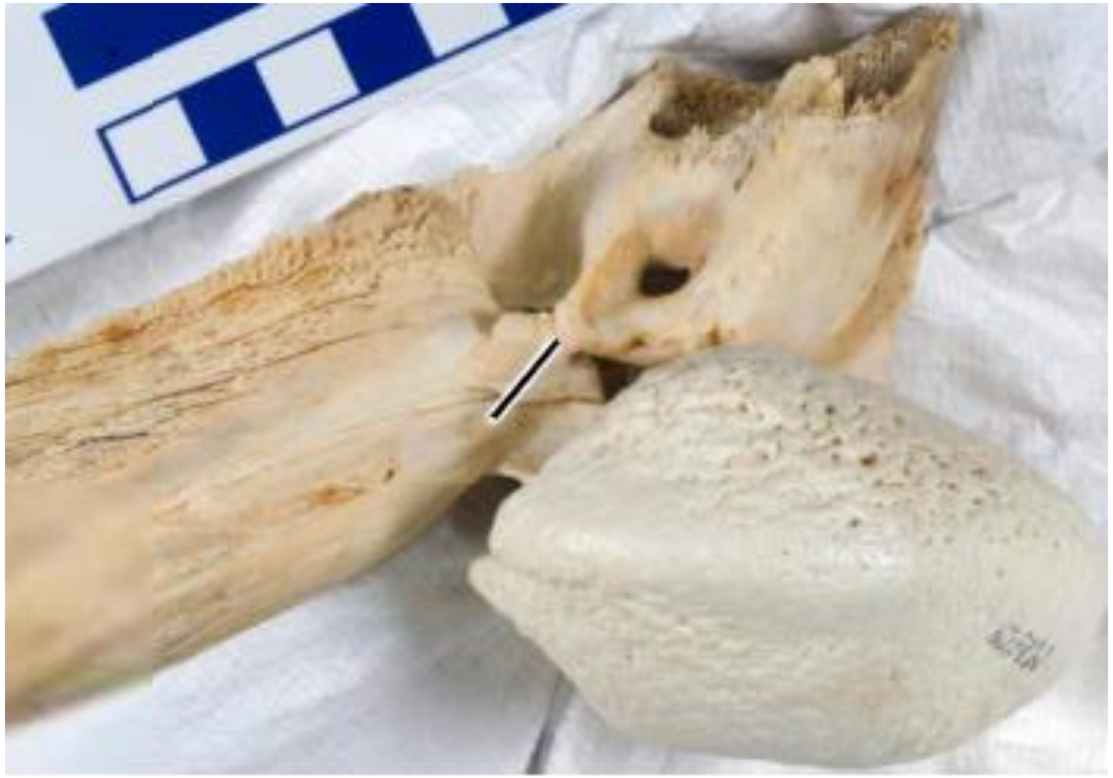

Balaenoptera acutorostrata

Copyright holder: Felix G. Marx/ National Museum of Nature and Science, Tokyo, Japan

[165] 'Posteromedial corner of pars cochlearis medial to fenestra cochleae'

(0)'rounded and level with fenestra cochleae'

(1)'inflated and projecting posteriorly so that the fenestra cochleae appears recessed into the posterior face of pars cochlearis'

(0)

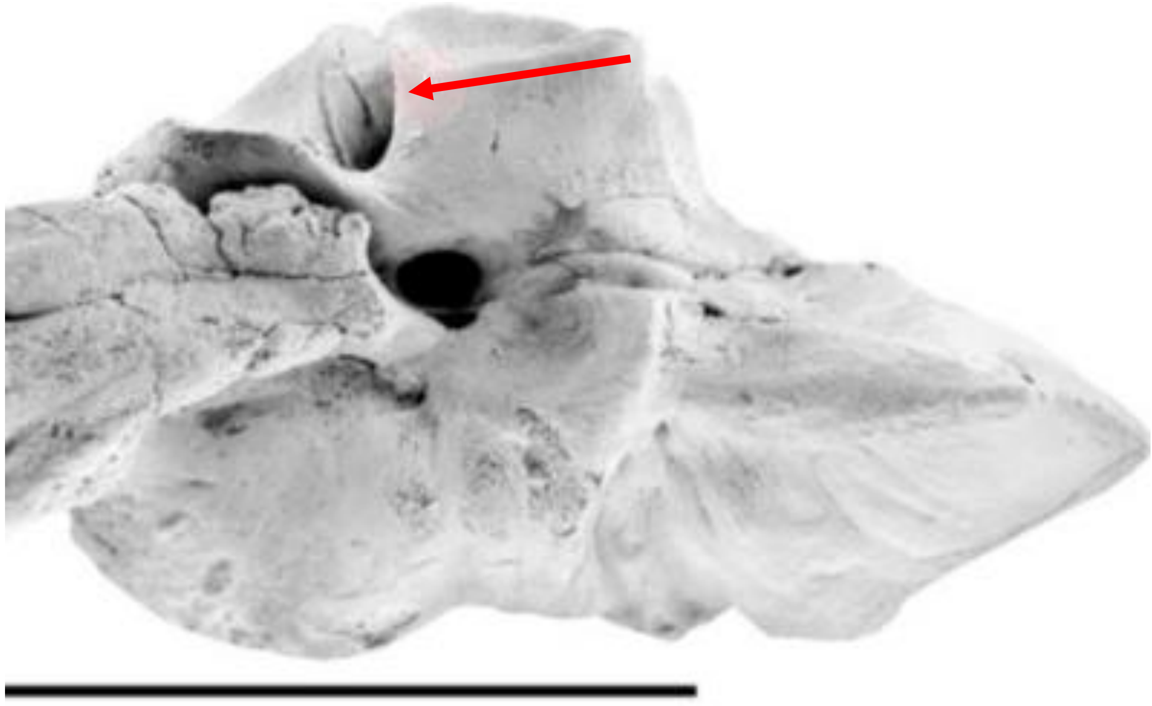

Whakakai waipata

Copyright holder: Cheng-Hsiu Tsai/University of Otago Geology Museum, Dunedin, New Zealand

(1)

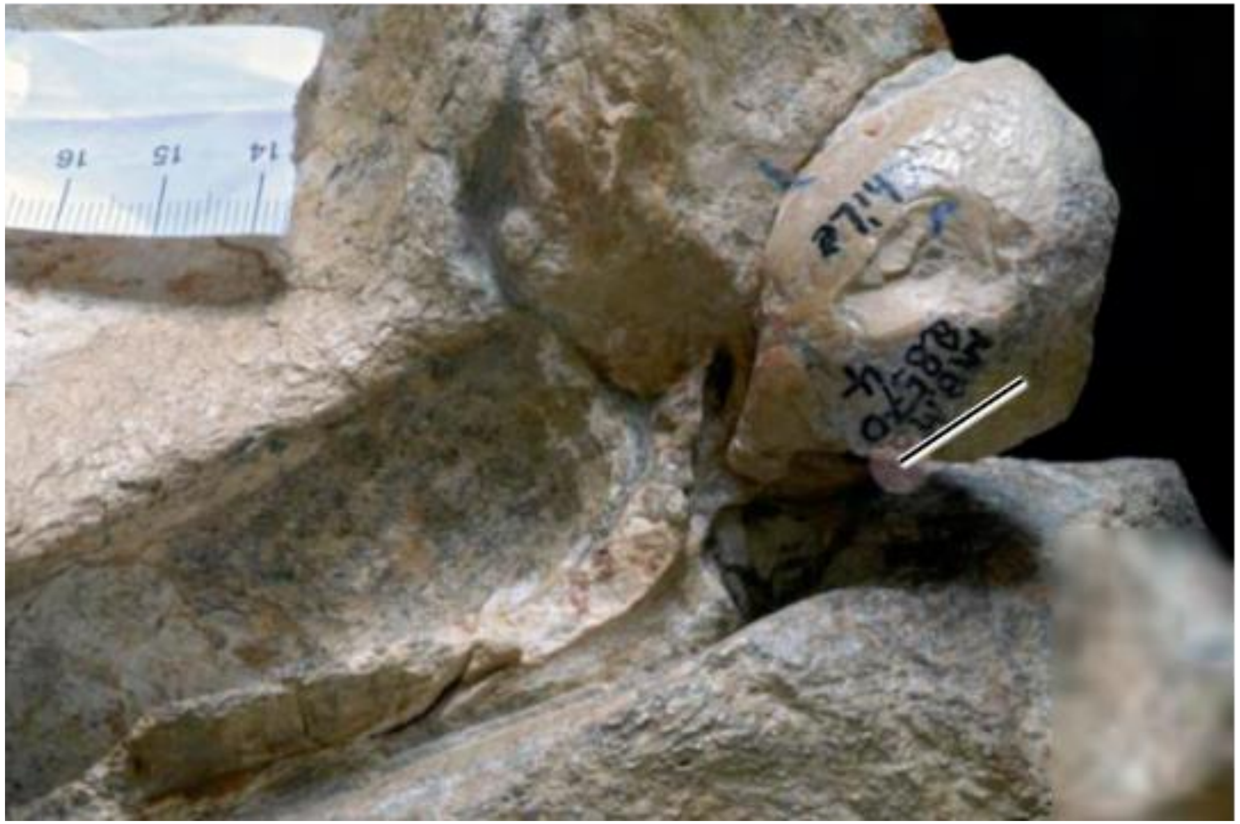

Megaptera hubachi

Copyright holder: Felix G. Marx/Museum fÃ¼r Naturkunde, Berlin, Germany

[166] 'Morphology of posterior cochlear crest'

(0) 'developed as a posteriorly extending triangular shelf'

(1) 'as state 0, but with the crest appearing sigmoidal in ventral view'

(2) 'as state 0, but with the ventral border bulging ventrally'

(3) 'as state 0, but pointing posterodorsally'

(4) 'developed as a robust, ventrally directed projection'

(5) 'absent or poorly developed'

(0)

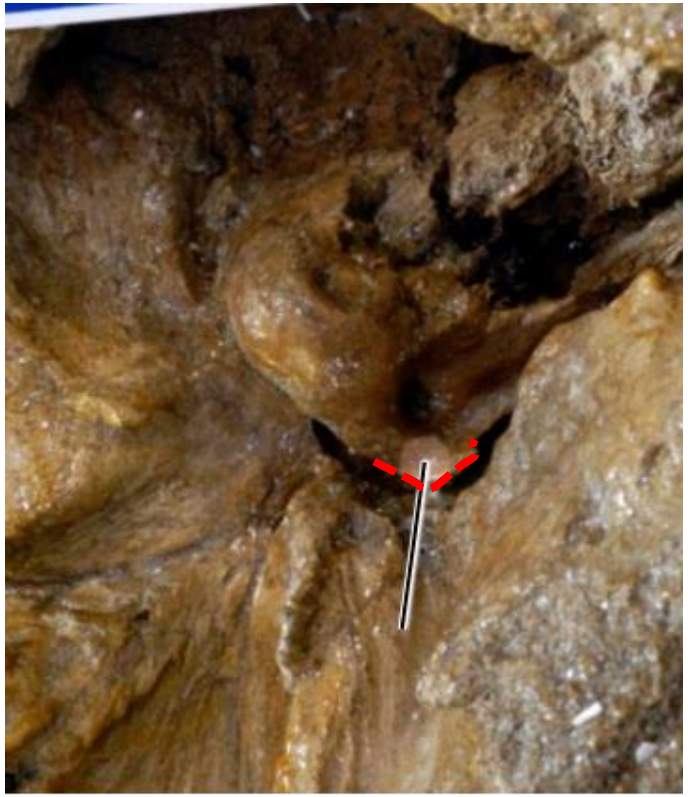

Pelocetus calvertensis

Copyright holder: Felix G. Marx/ United States National Museum of Natural History, Washington DC, USA

(1)

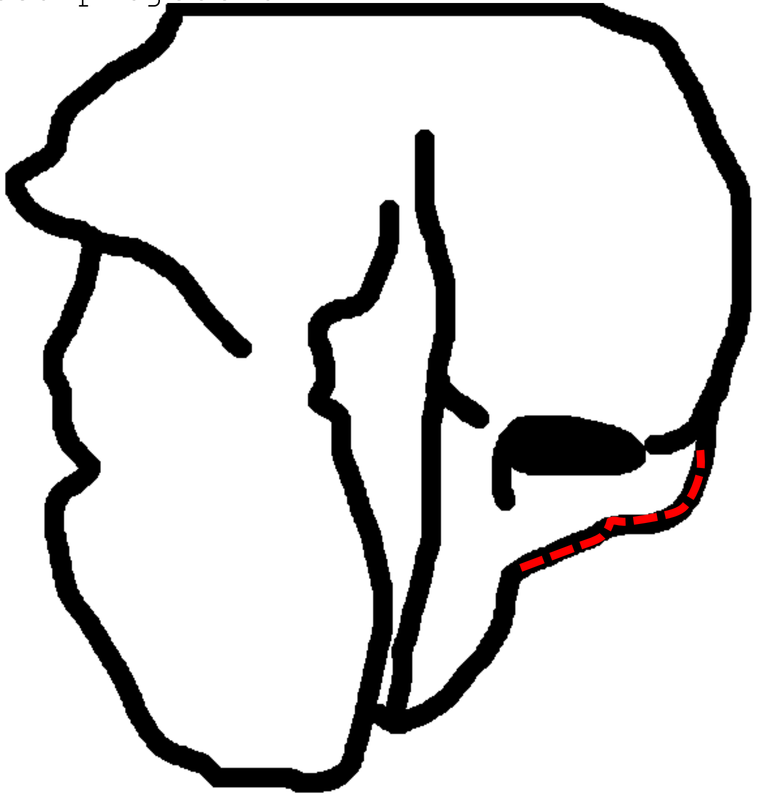

Example based on *Albertocetus meffordorum*

(2)

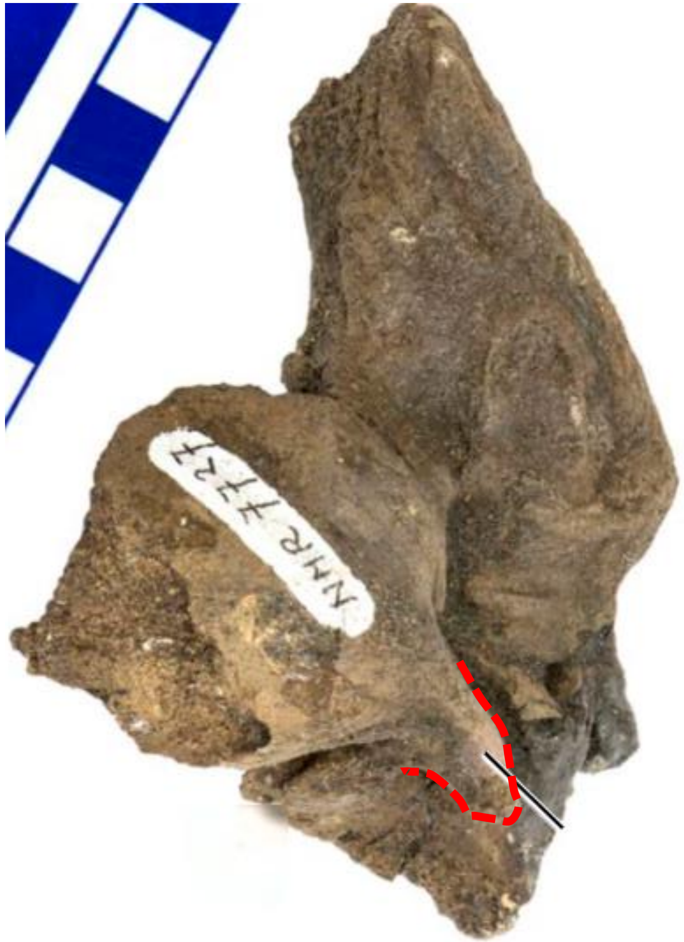

Fragilicetus velponi

Copyright holder: Felix G. Marx/ Natural History Museum Rotterdam, The Netherlands

[166] 'Morphology of posterior cochlear crest'

(0) 'developed as a posteriorly extending triangular shelf'

(1) 'as state 0, but with the crest appearing sigmoidal in ventral view'

(2) 'as state 0, but with the ventral border bulging ventrally'

(3) 'as state 0, but pointing posterodorsally'

(4) 'developed as a robust, ventrally directed projection'

(5) 'absent or poorly developed'

(3)

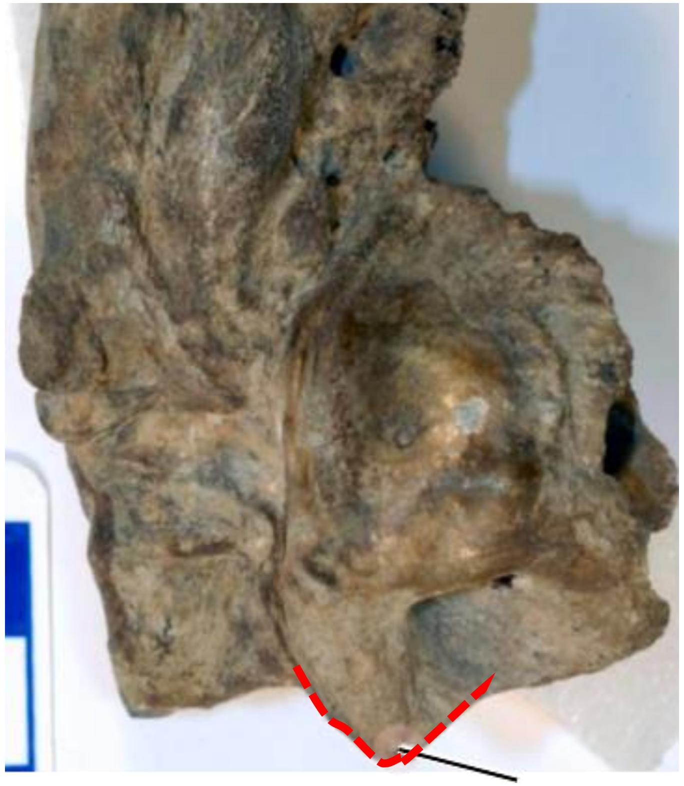

Metopocetus durinasus

Copyright holder: Felix G. Marx/United States National Museum of Natural History, Washington DC, USA

(4)

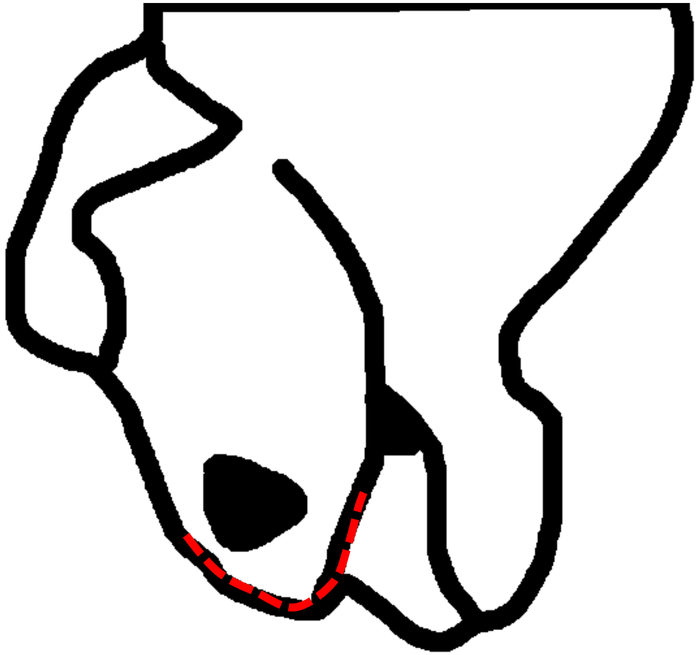

Example based on *Herpetocetus morrowi*

(5)

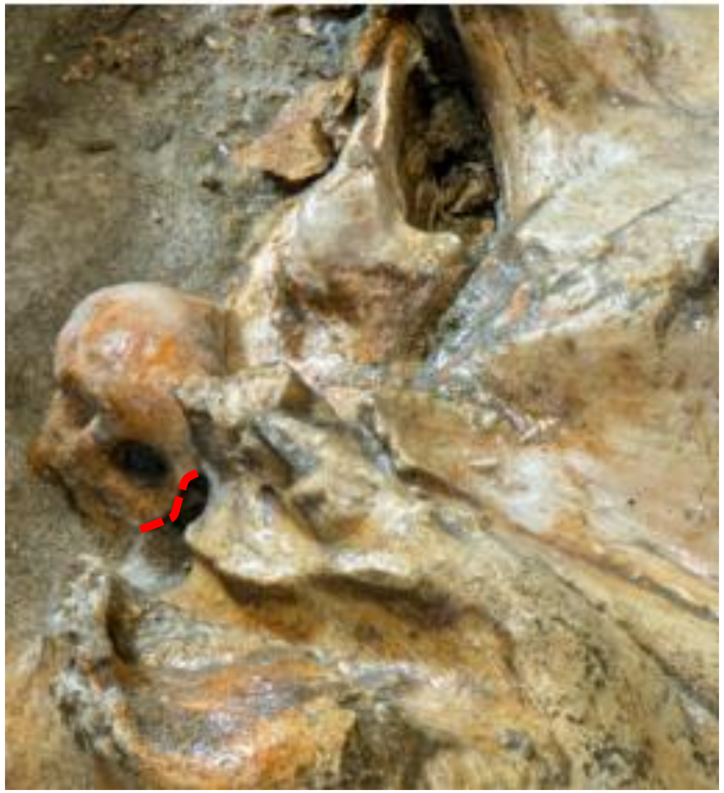

Tiucetus rosae

Adapted from: *"A new Miocene baleen whale from Peru deciphers the dawn of cetotheriids." Marx, Lambert, and De Muizon, 2017. Royal Society Open Science 4.9: 170560.*

[167] 'Elongate lobe and fossa extending posteromedially from fenestra vestibuli in ventral view'

(0)'absent'

(1)'present'

(0)

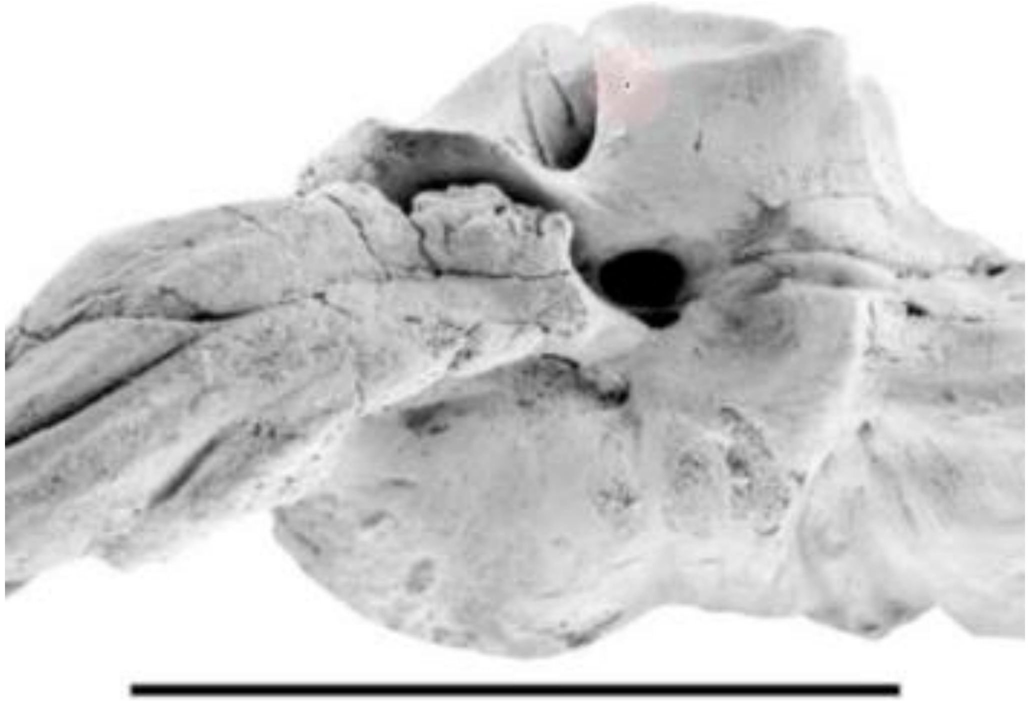

Whakakai waipata

Copyright holder: Cheng-Hsiu Tsai/University of Otago Geology Museum, Dunedin, New Zealand

(1)

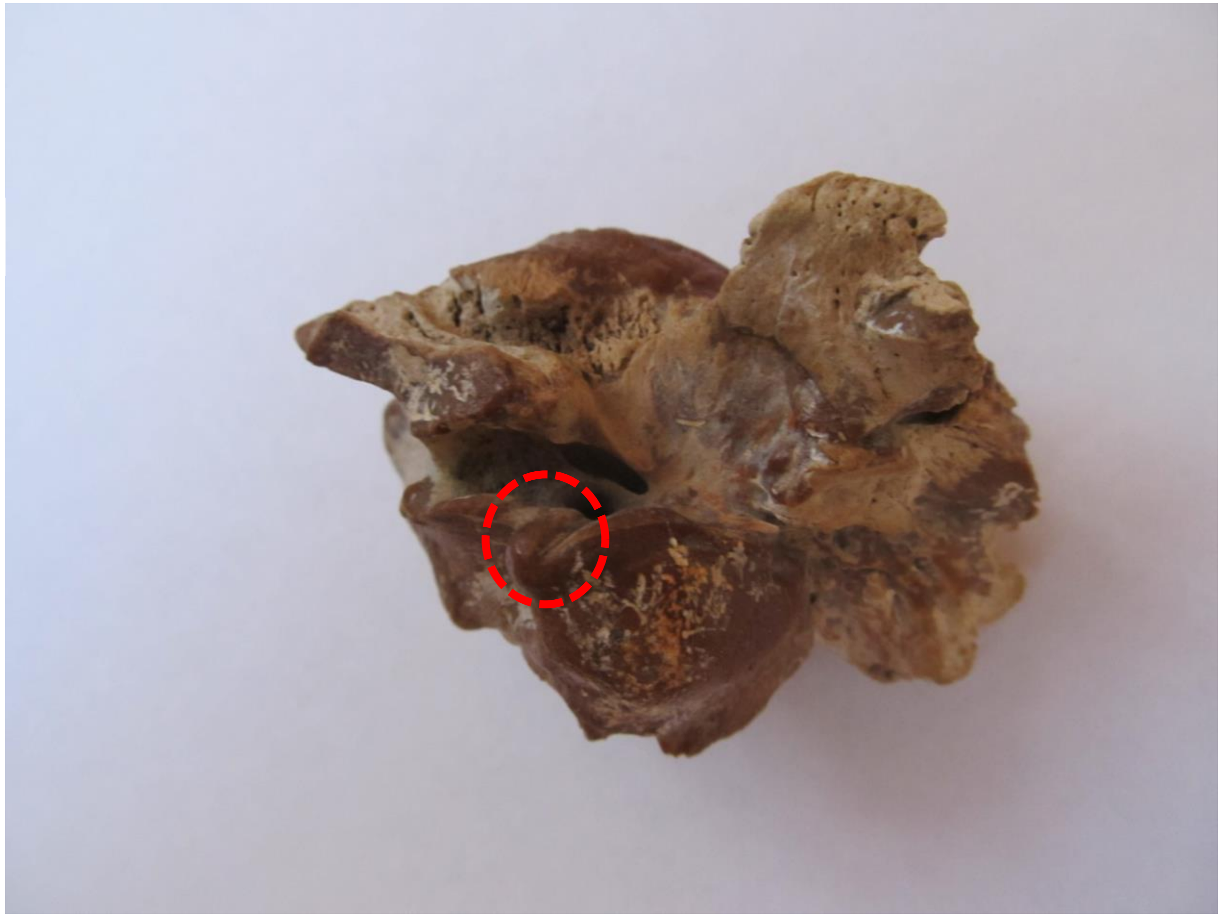

Brandtocetus chongulek

Copyright holder: Pavel Gol'din

[168] 'Anteroposterior alignment of proximal opening of facial canal, dorsal vestibular area and aperture for cochlear aqueduct'

(0) 'present'

(1) 'absent'

(0)

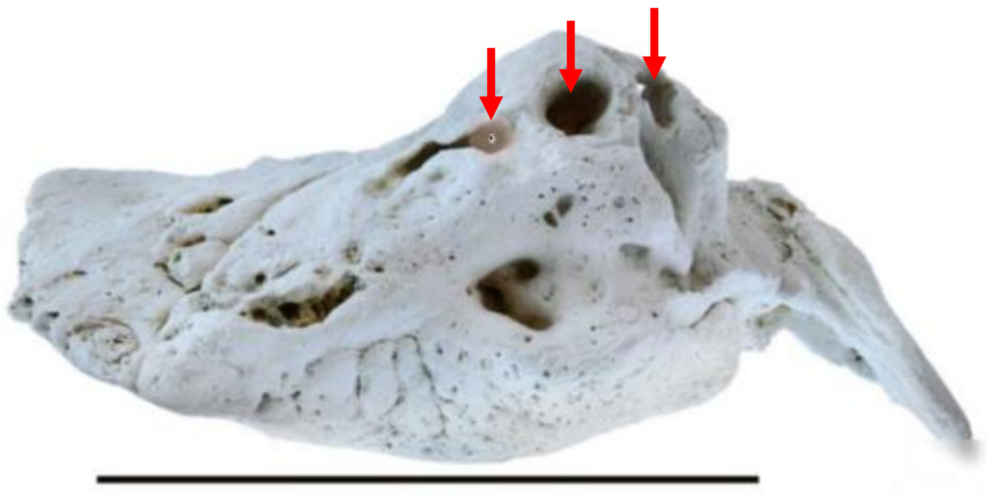

Horopeta umarere

Copyright holder: Cheng-Hsiu Tsai/ Otago University Geology Museum, Dunedin, New Zealand

(1)

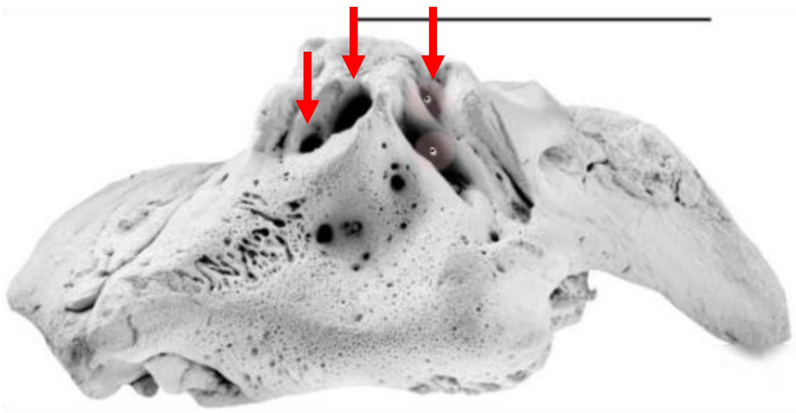

Whakakai waipata

Copyright holder: Cheng-Hsiu Tsai/University of Otago Geology Museum, Dunedin, New Zealand

[169] 'Anteroposterior alignment of aperture for cochlear aqueduct and aperture for vestibular aqueduct'

(0)'absent'

(1)'present'

(0)

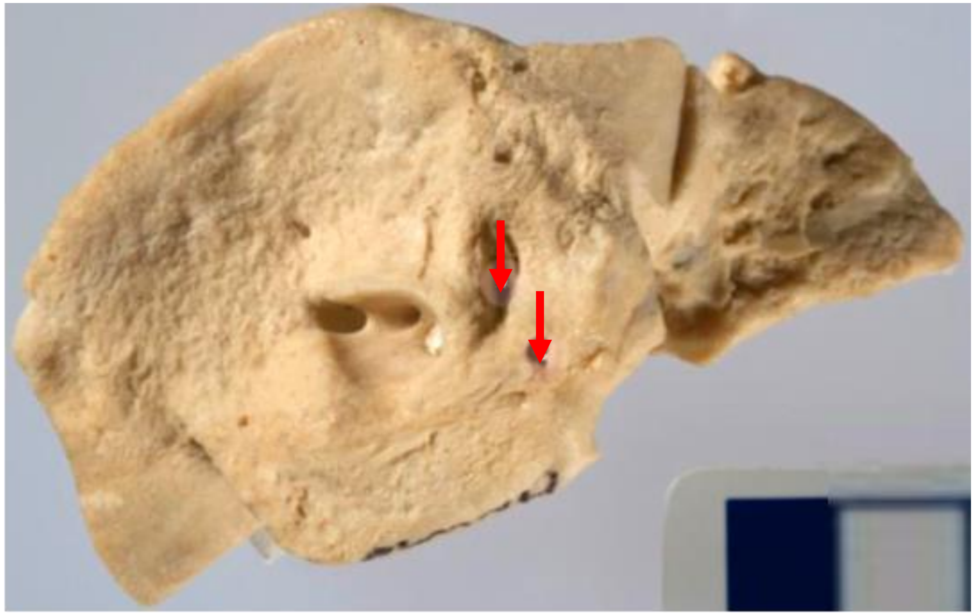

Zygorhiza kochii

Copyright holder: Felix G. Marx/ Alabama Museum of Natural History, Tuscaloosa, Alabama, USA

(1)

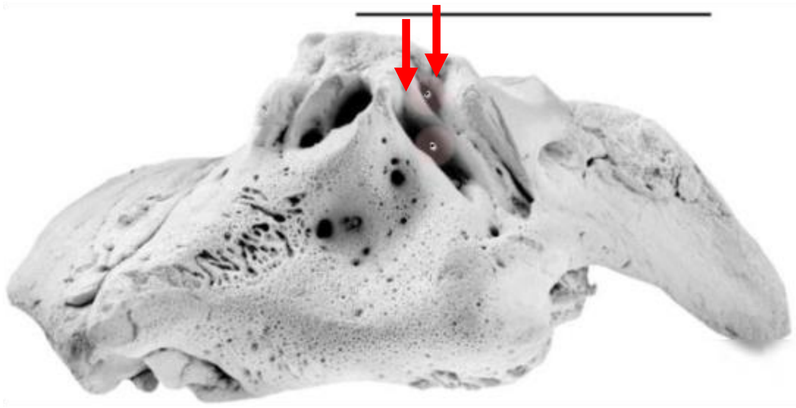

Whakakai waipata

Copyright holder: Cheng-Hsiu Tsai/University of Otago Geology Museum, Dunedin, New Zealand

[170] 'Prominent septum dividing foramen singulare from spiral cribriform tract'

(0) 'present'

(1) 'absent'

(0)

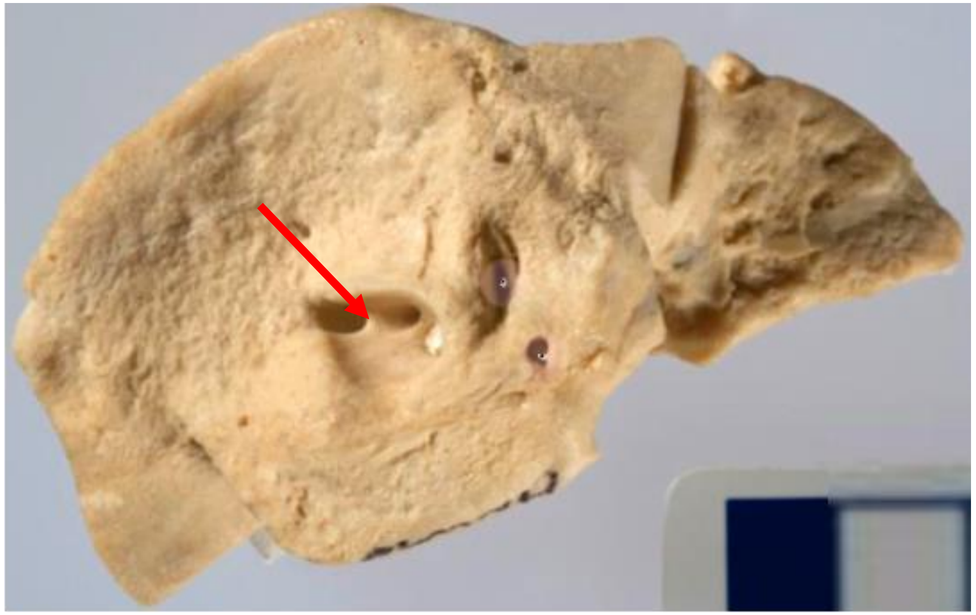

Zygorhiza kochii

Copyright holder: Felix G. Marx/ Alabama Museum of Natural History, Tuscaloosa, Alabama, USA

(1)

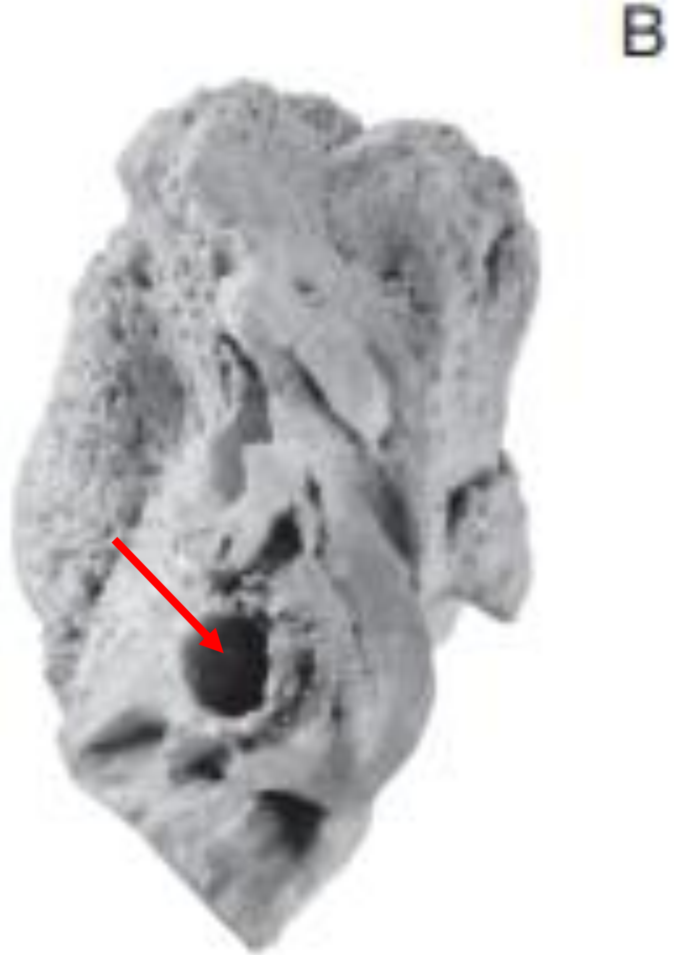

Piscobalaena nana

Adapted from: "The anatomy and relationships of *Piscobalaena nana* (Cetacea, Mysticeti), a Cetotheriidae s.s. from the early Pliocene of Peru", Bouetel and Muizon, 2006. *Geodiversitas* 28.2 (2006): 319-395.

[171] 'Sharp cranial rim surrounding proximal opening of facial canal'

(0) 'absent'

(1) 'present'

(0)

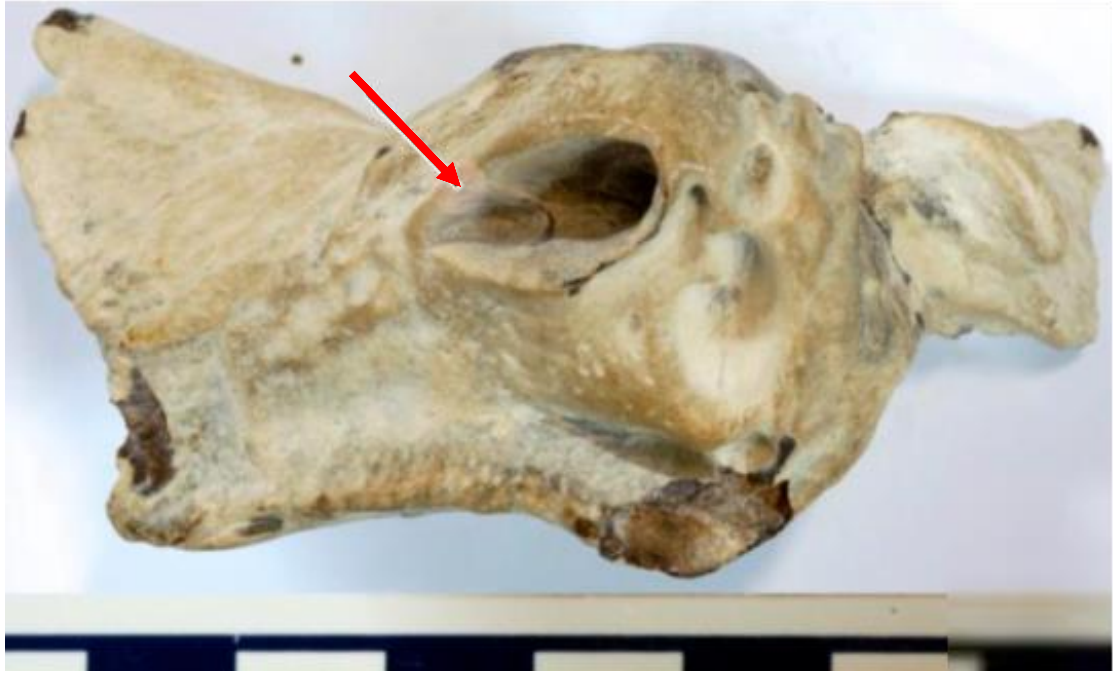

Micromysticetus rothauseni

Copyright holder: Felix G. Marx/ The Charleston Museum, Charleston, USA

(1)

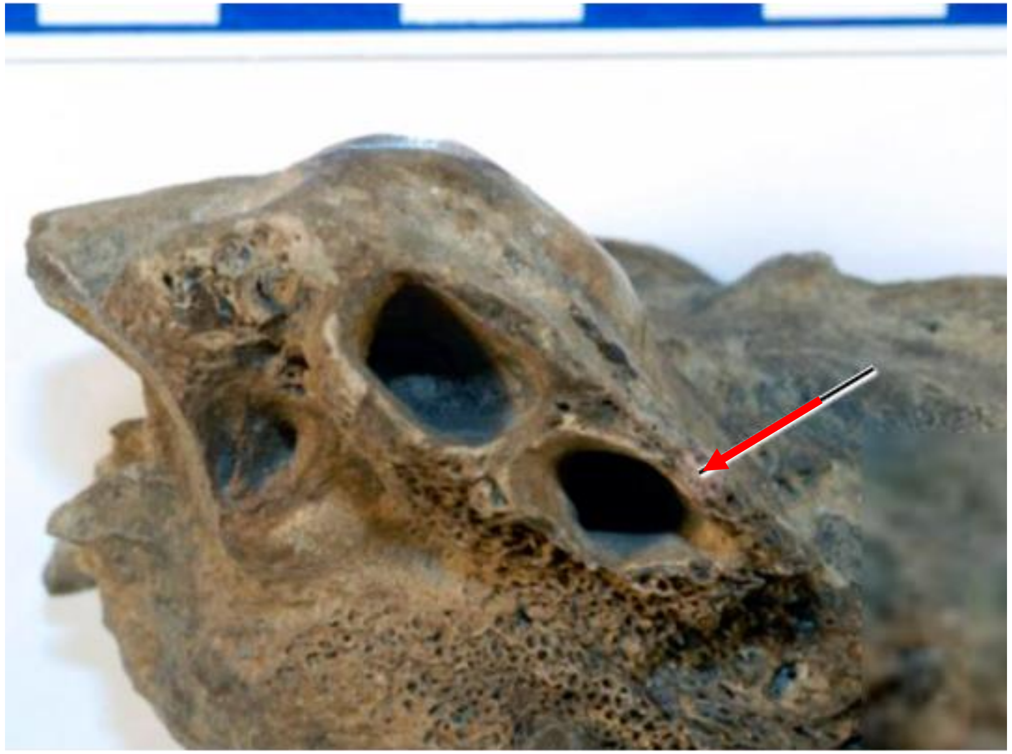

Metopocetus durinasus

Copyright holder: Felix G. Marx/United States National Museum of Natural History, Washington DC, USA

[172] 'Shape of aperture for cochlear aqueduct'

(0)'round with sharply defined dorsal margins'

(1)'slit-like'

(0)

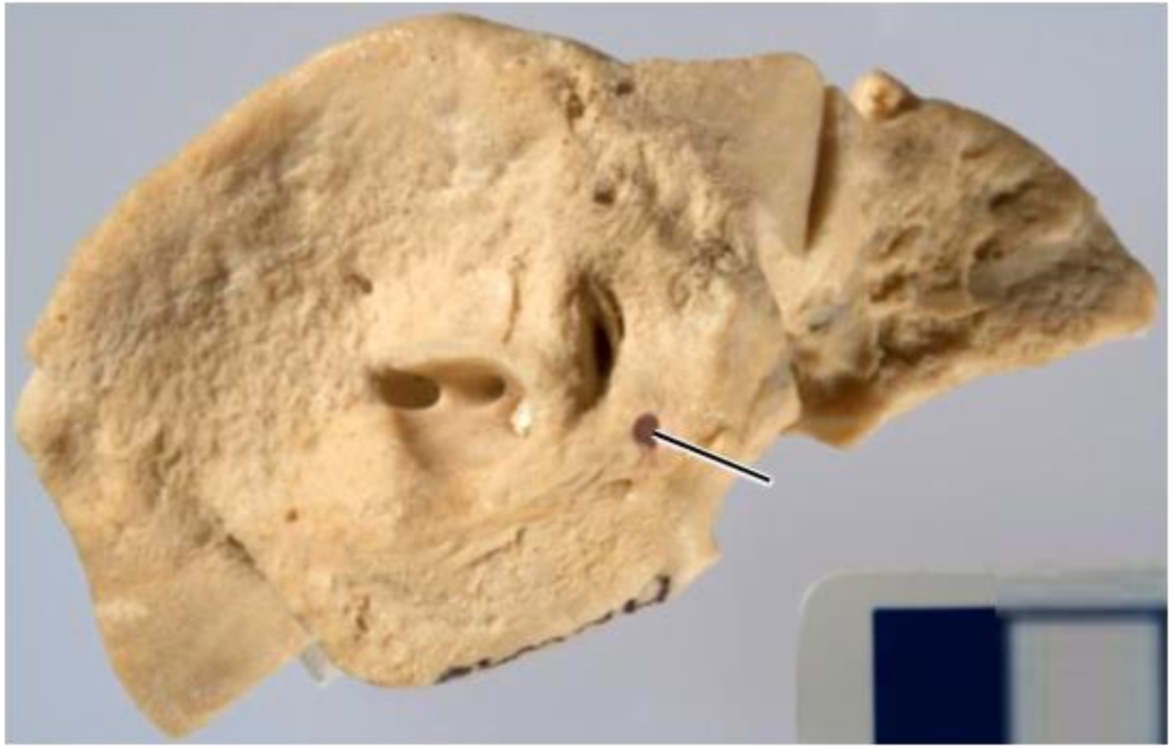

Zygorhiza kochii

Copyright holder: Felix G. Marx/ Alabama Museum of Natural History, Tuscaloosa, Alabama, USA

(1)

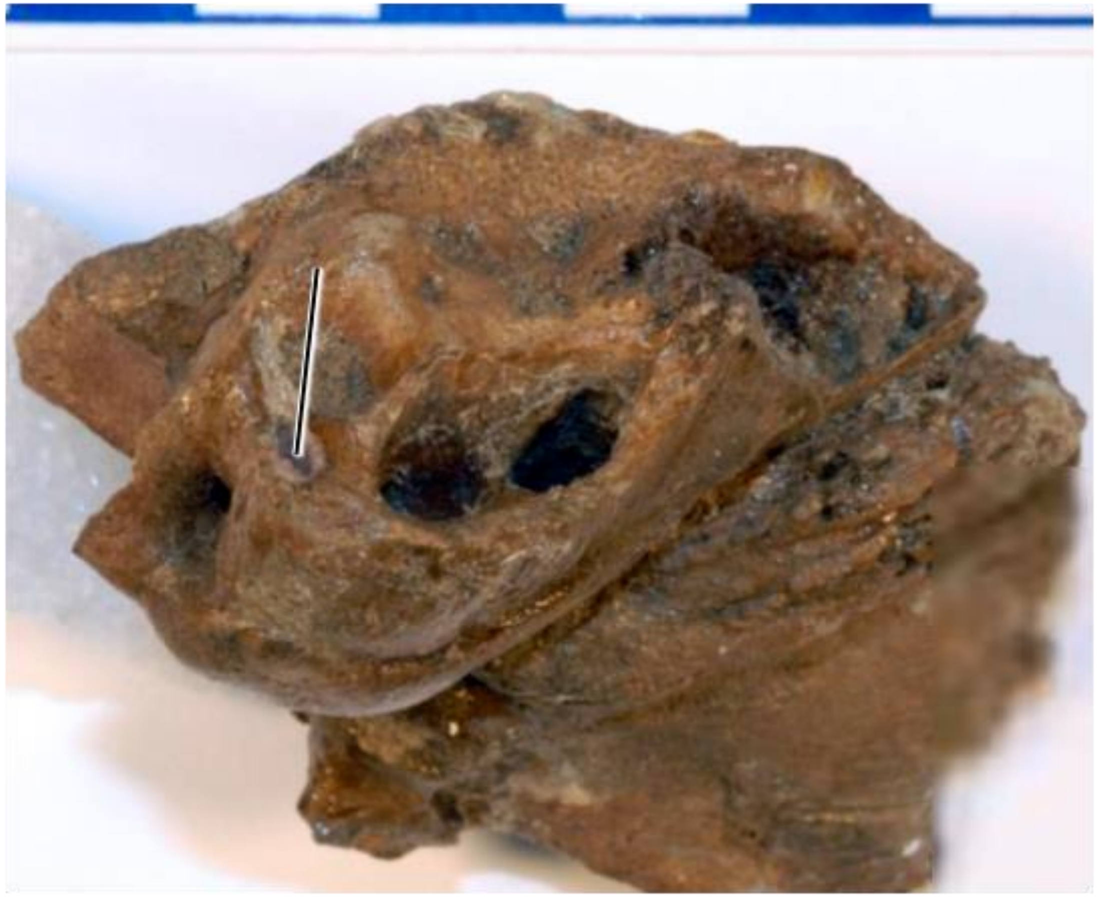

Diorocetus hiatus

Copyright holder: Felix G. Marx/ United States National Museum of Natural History, Washington DC, USA

[173] 'Size of aperture for cochlear aqueduct'

(0) 'smaller than aperture for vestibular aqueduct'

(1) 'approximately the same size'

(0)

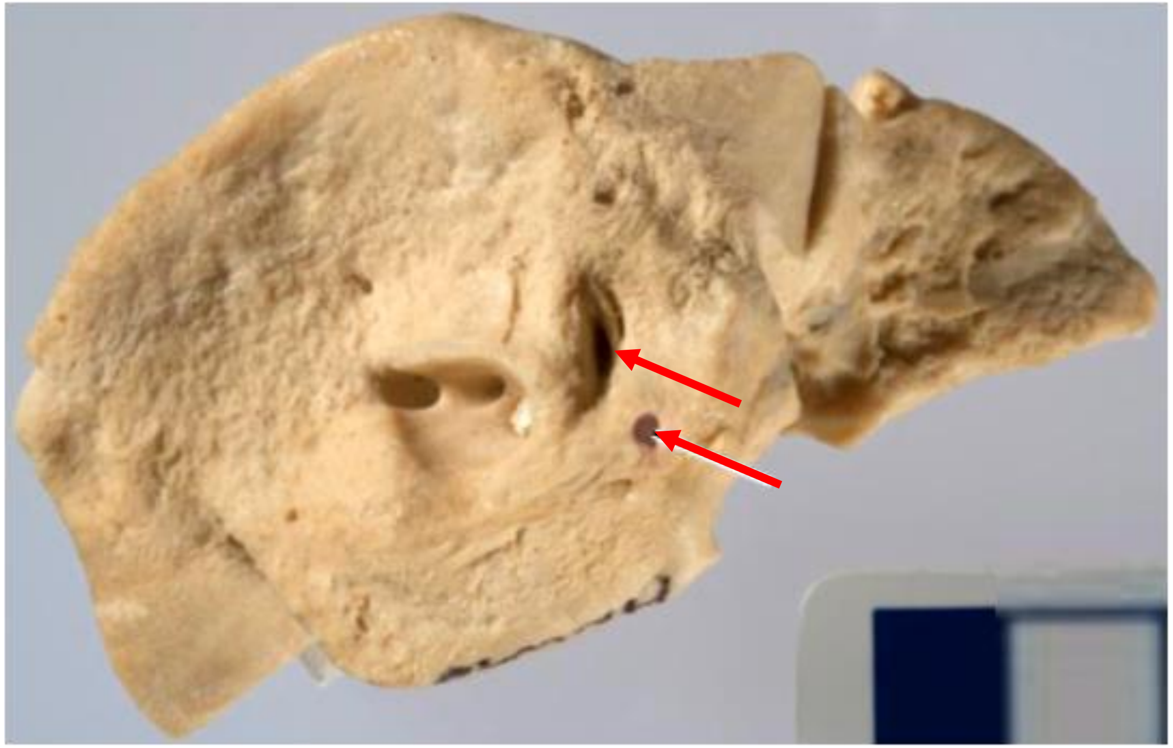

Zygorhiza kochii

Copyright holder: Felix G. Marx/ Alabama Museum of Natural History, Tuscaloosa, Alabama, USA

(1)

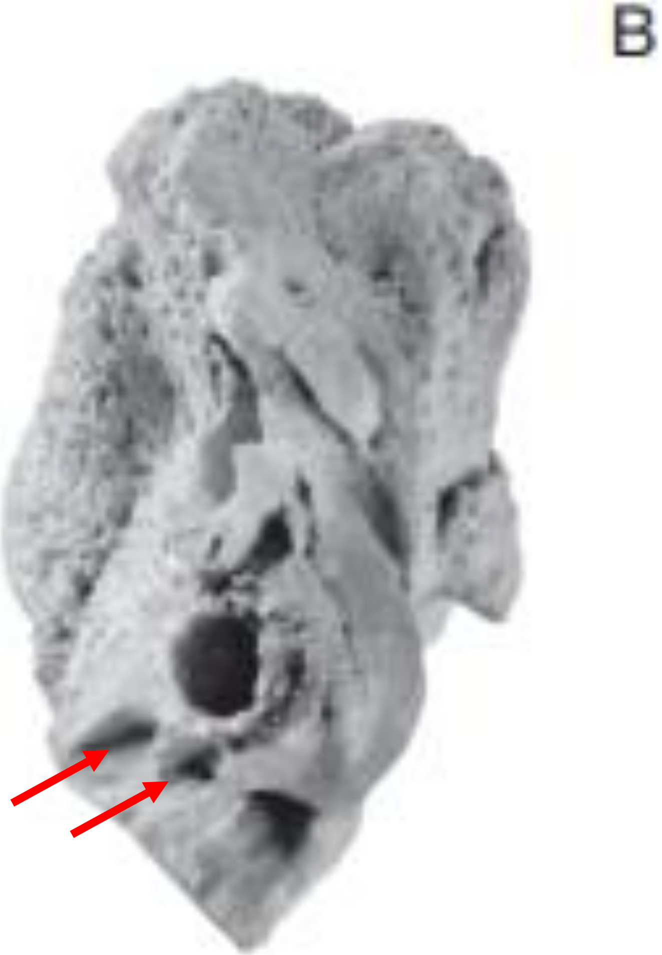

Piscobalaena nana

Adapted from: “The anatomy and relationships of *Piscobalaena nana* (Cetacea, Mysticeti), a Cetotheriidae s.s. from the early Pliocene of Peru”, Bouetel and Muizon, 2006. *Geodiversitas* 28.2 (2006): 319-395.

[174] 'Aperture for cochlear aqueduct and fenestra cochleae'

(0)'separate'

(1)'confluent'

(0)

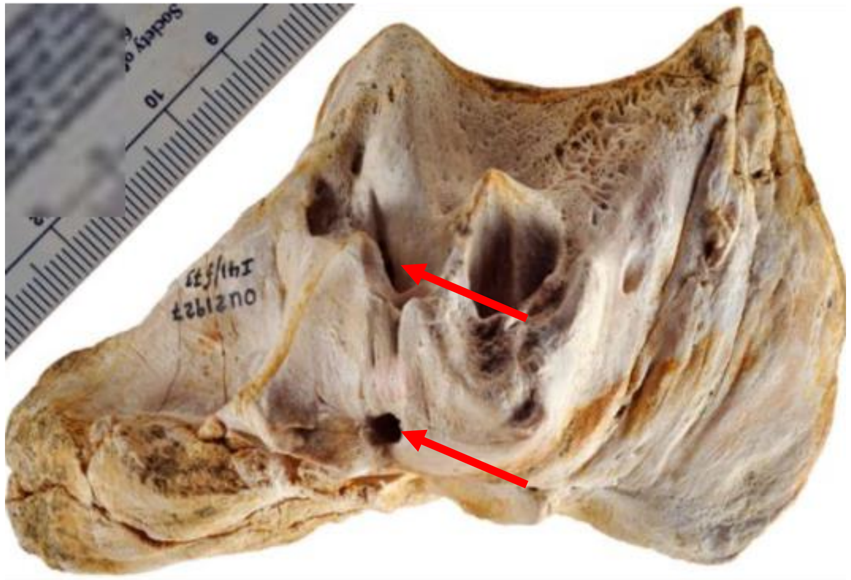

Whakakai waipata

Copyright holder: Cheng-Hsiu Tsai/University of Otago Geology Museum, Dunedin, New Zealand

(1)

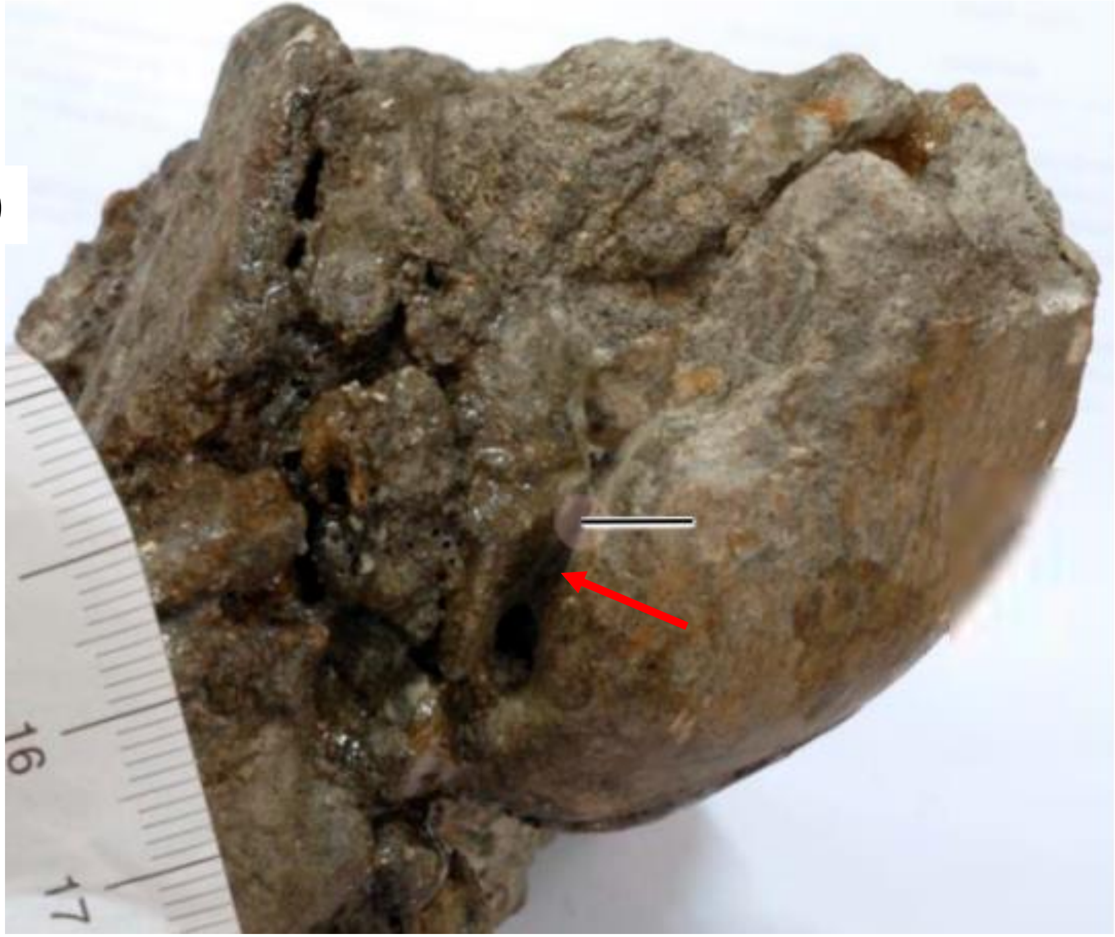

Plesiobalaenoptera quarantellii

Copyright holder: Felix G. Marx/ Museo Paleontologico di Salsomaggiore Terme/ Soprintendenza Per I Beni Archeologici Dell™Emilia Romagna, Italy

[175] 'Superior process'

(0)'present as a distinct crest forming the lateral wall of the suprameatal fossa'

(1)'present but low, especially along its central portion'

(2)'absent'

(0)

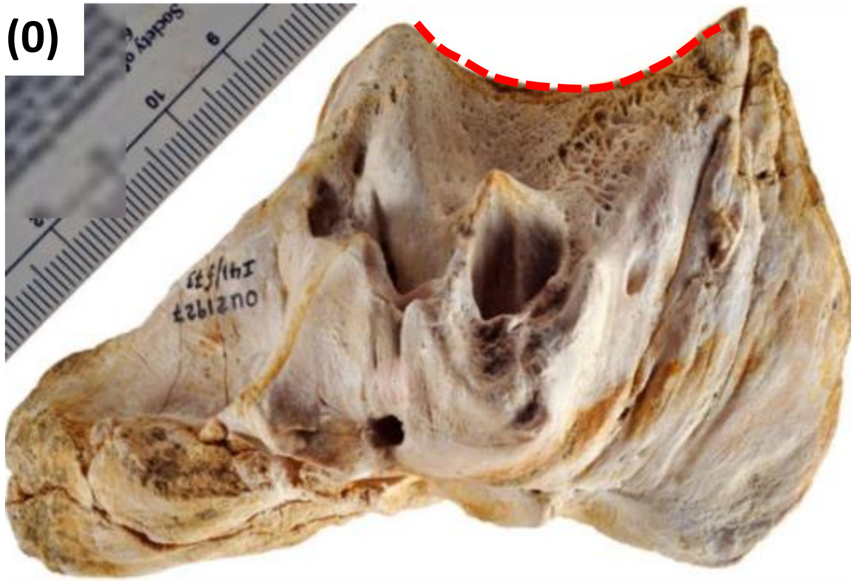

Whakakai waipata

Copyright holder: Cheng-Hsiu Tsai/University of Otago Geology Museum, Dunedin, New Zealand

(1)

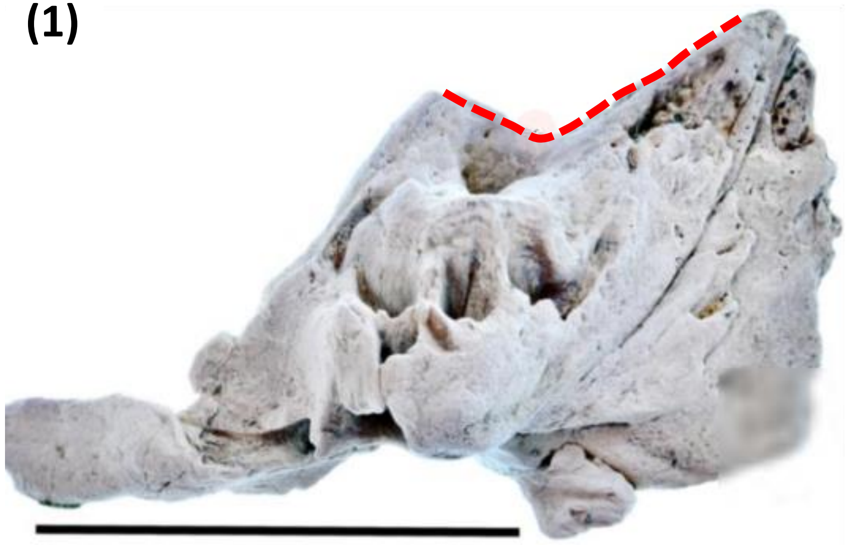

Horopeta umarere

Copyright holder: Cheng-Hsiu Tsai/ Otago University Geology Museum, Dunedin, New Zealand

(2)

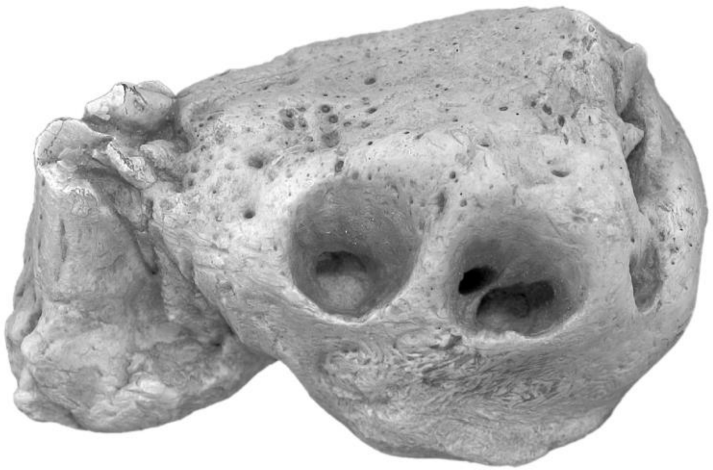

Mammalodon colliveri

Copyright holder: Erich M. G. Fitzgerald/ Museums Victoria, Melbourne, Australia

[176] 'Suprameatal area hypertrophied'

(0)'absent’

(1)'present'

(0)

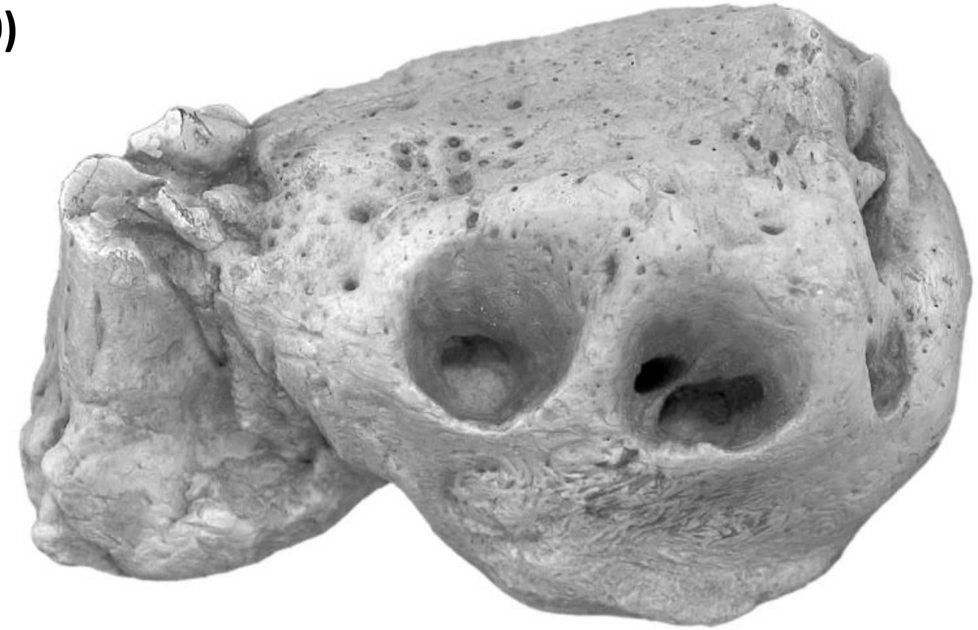

Mammalodon colliveri

Copyright holder: Erich M. G. Fitzgerald/ Museums Victoria, Melbourne, Australia

(1)

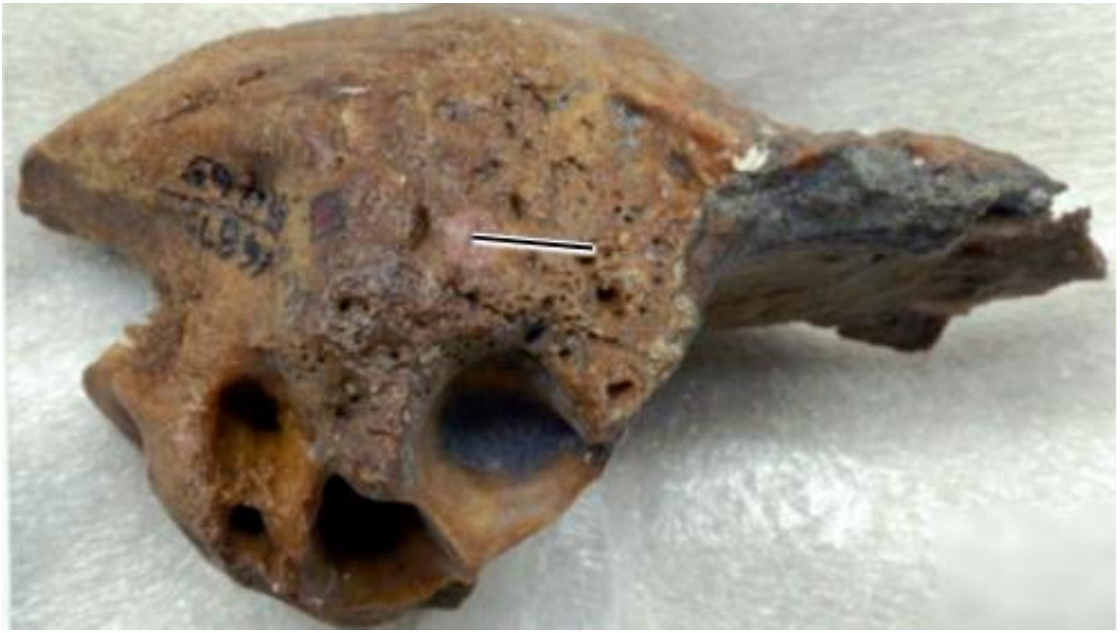

Herpetocetus bramblei

Copyright holder: Felix G. Marx/ University of California Museum of Paleontology, Berkeley, USA

[177] 'Development of transverse crest'

(0) 'depressed well below the rim of the internal acoustic meatus’

(1) 'well developed and reaching the cerebral surface of the pars cochlearis'

(0)

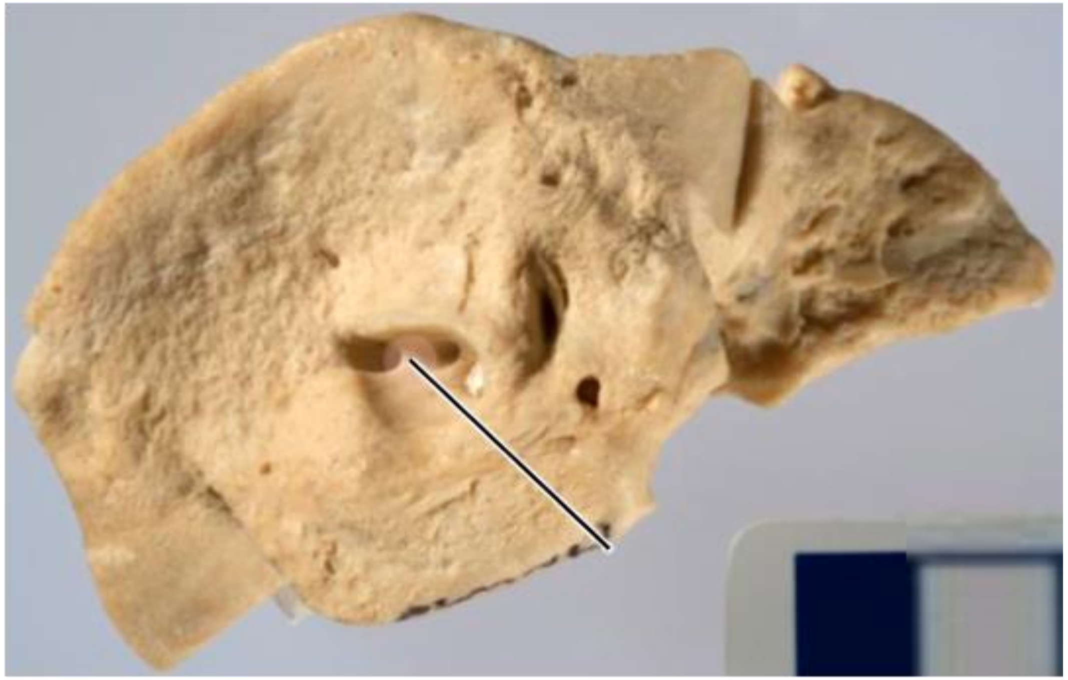

Zygorhiza kochii

Copyright holder: Felix G. Marx/ Alabama Museum of Natural History, Tuscaloosa, Alabama, USA

(1)

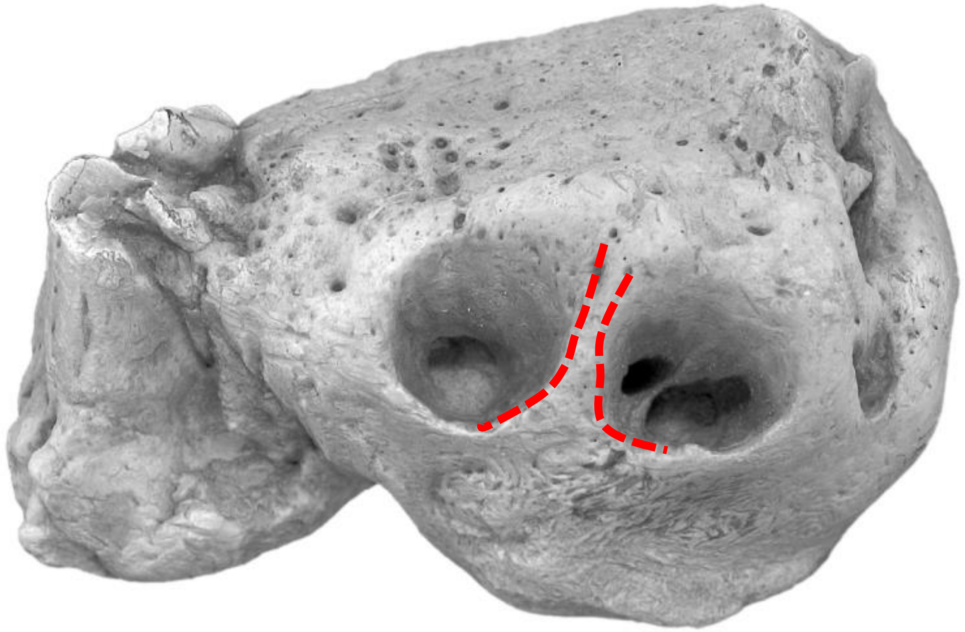

Mammalodon colliveri

Copyright holder: Erich M. G. Fitzgerald/ Museums Victoria, Melbourne, Australia

[178] 'Morphology of transverse crest'

(0)'developed as a septum of varying thickness’

(1)'proximal opening of facial canal is widely separated from internal acoustic meatus and connected to the latter via a distinct sulcus'

(0)

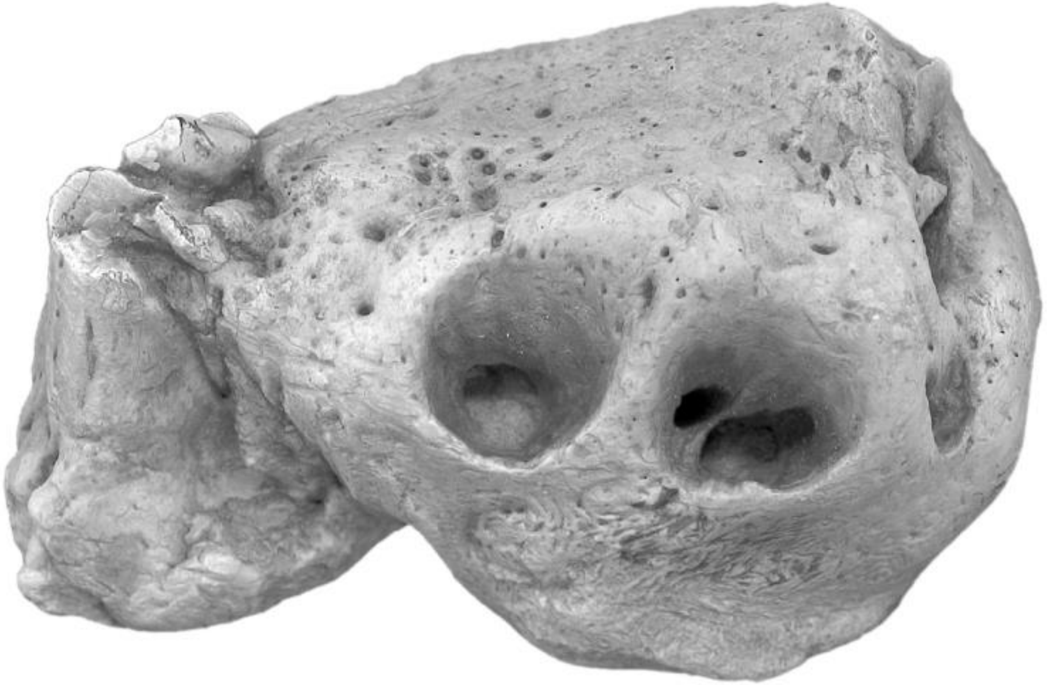

Mammalodon colliveri

Copyright holder: Erich M. G. Fitzgerald/ Museums Victoria, Melbourne, Australia

(1)

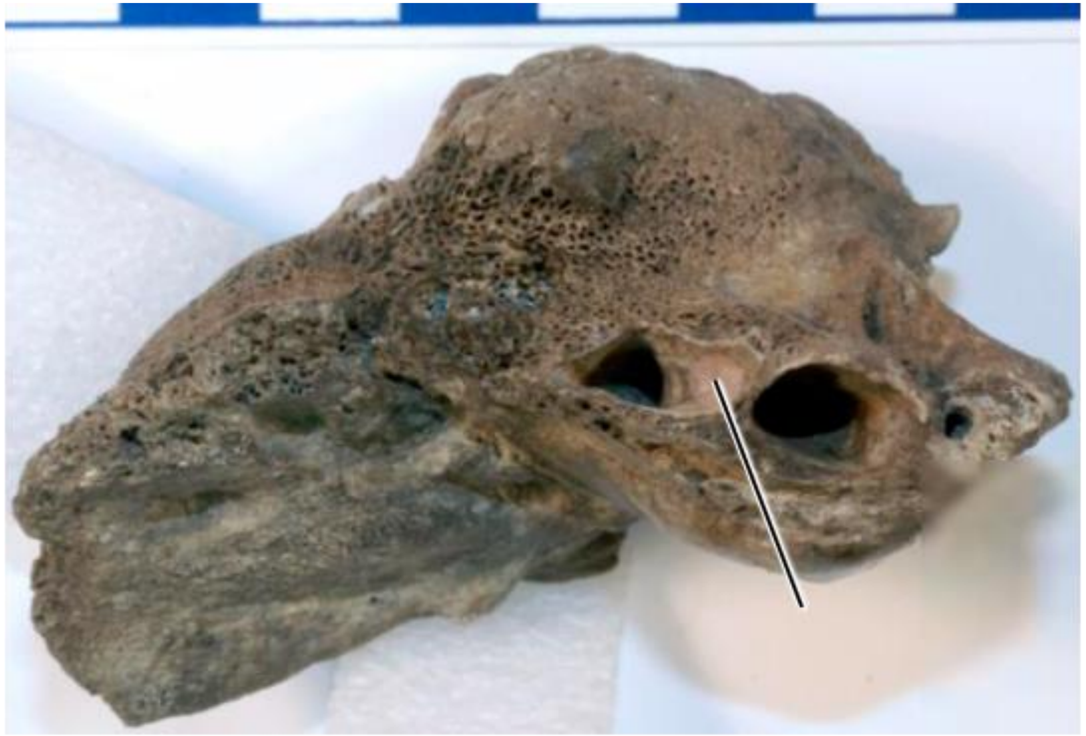

Metopocetus durinasus

Copyright holder: Felix G. Marx/United States National Museum of Natural History, Washington DC, USA

[179] 'Hiatus Fallopii'

(0)'absent or small opening located anterior or anteroventral to proximal opening of facial canal'

(1)'as state 0, but with the hiatus Fallopii being well developed and large'

(2)'anterior border of proximal opening of facial canal is continuous with the hiatus Fallopii and shaped like a fissure'

(0)

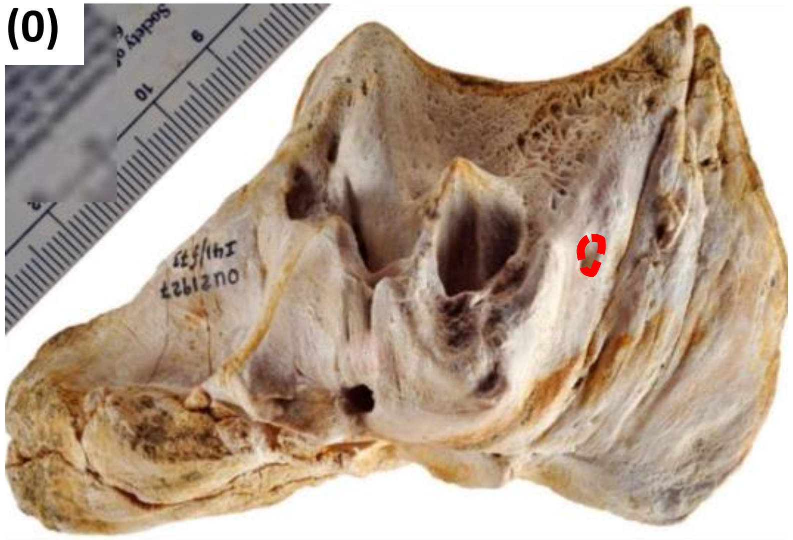

Whakakai waipata

Copyright holder: Cheng-Hsiu Tsai/University of Otago Geology Museum, Dunedin, New Zealand

(1)

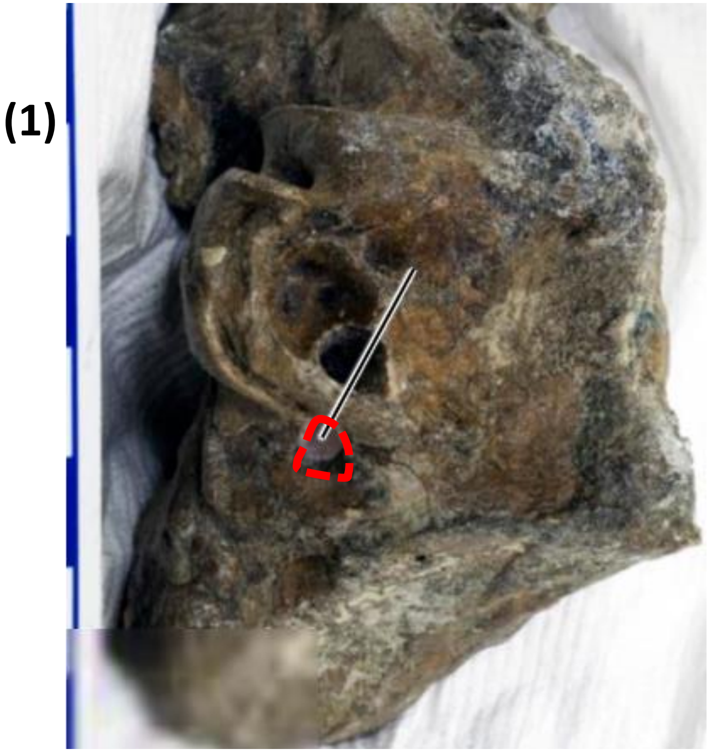

Balaenula sp.

Copyright holder: Felix G. Marx/ Sapporo Museum Activity Centre, Sapporo, Japan

(2)

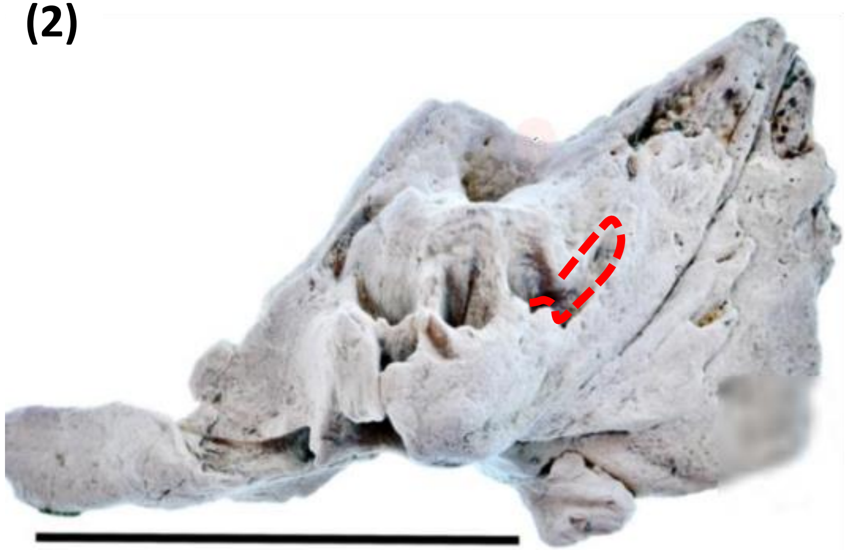

Horopeta umarere

Copyright holder: Cheng-Hsiu Tsai/ Otago University Geology Museum, Dunedin, New Zealand

[180] 'Size of proximal opening of facial canal'

(0)'no more than half the size of the internal acoustic meatus'

(1)'more than half the size of the internal acoustic meatus'

(0)

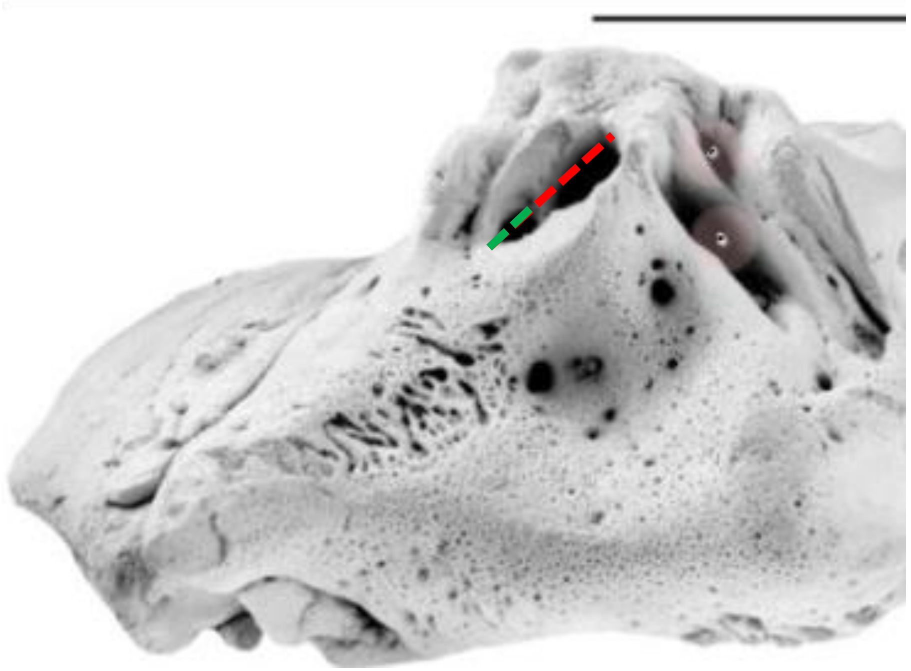

(1)

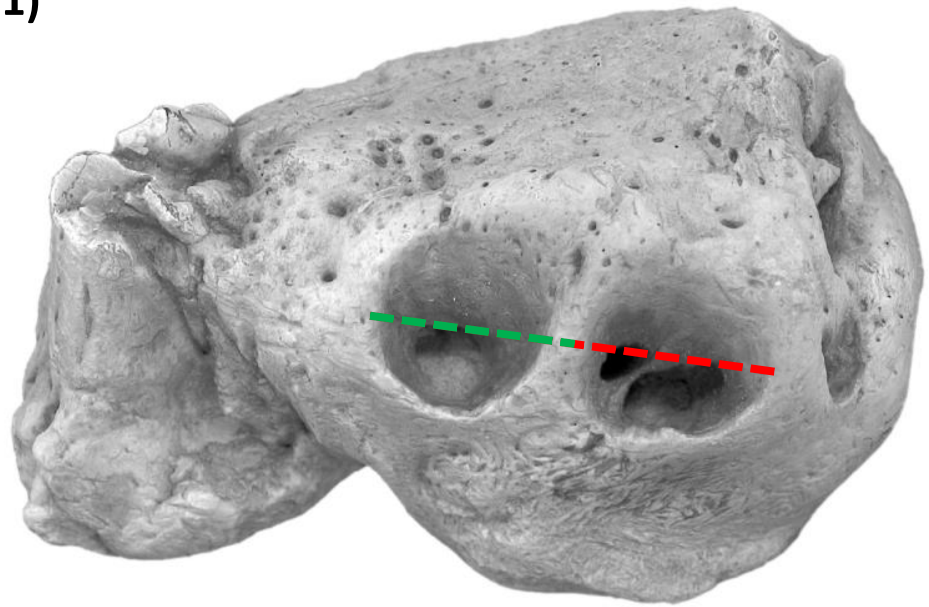

Whakakai waipata

Copyright holder: Cheng-Hsiu Tsai/University of Otago Geology Museum, Dunedin, New Zealand

Mammalodon colliveri

Copyright holder: Erich M. G. Fitzgerald/ Museums Victoria, Melbourne, Australia

[182] 'Squamosal flange located posterior to lateral tuberosity'

(0)'absent'

(1)'present'

(0)

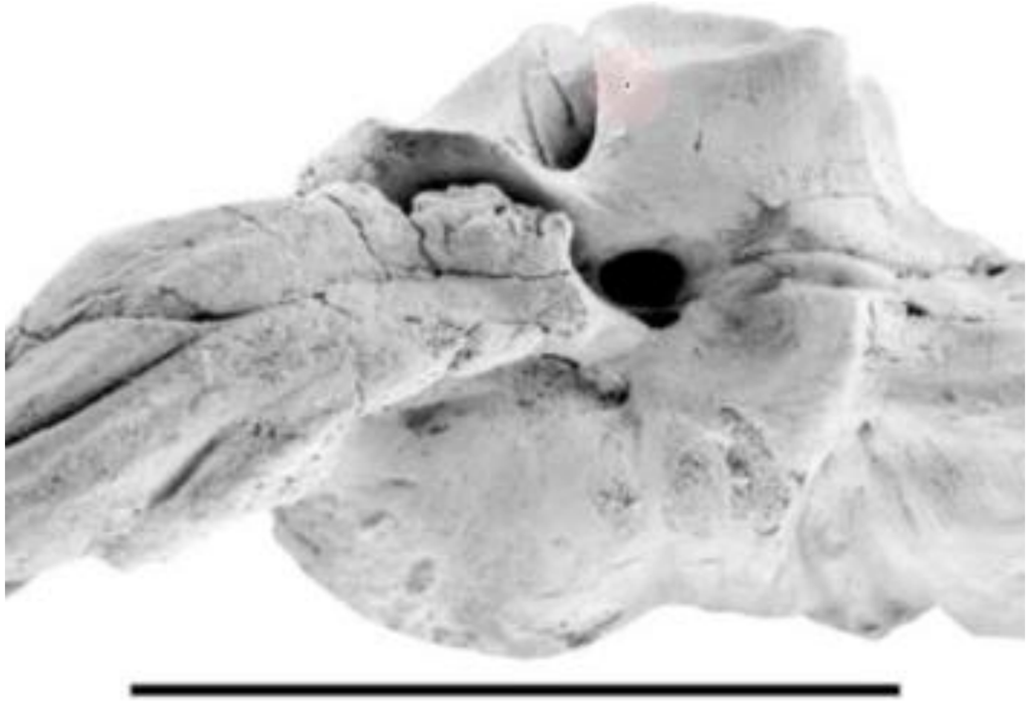

Whakakai waipata

Copyright holder: Cheng-Hsiu Tsai/University of Otago Geology Museum, Dunedin, New Zealand

(1)

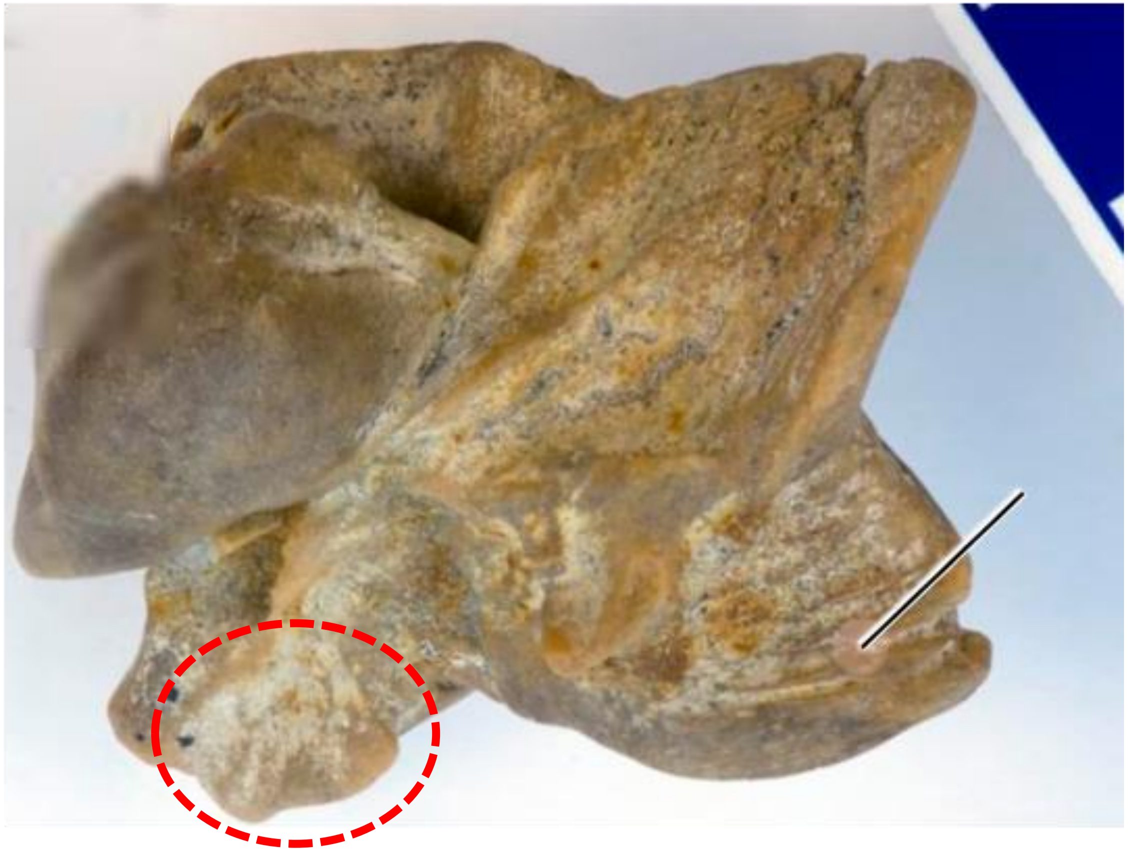

Herpetocetus morrowi

Copyright holder: Felix G. Marx/ San Diego Museum of Natural History, San Diego, USA

[183] 'Articulation surfaces on posterior processes of tympanic bulla and periotic'

(0)'unfused'

(1)'fused in adults to form compound posterior process'

(0)

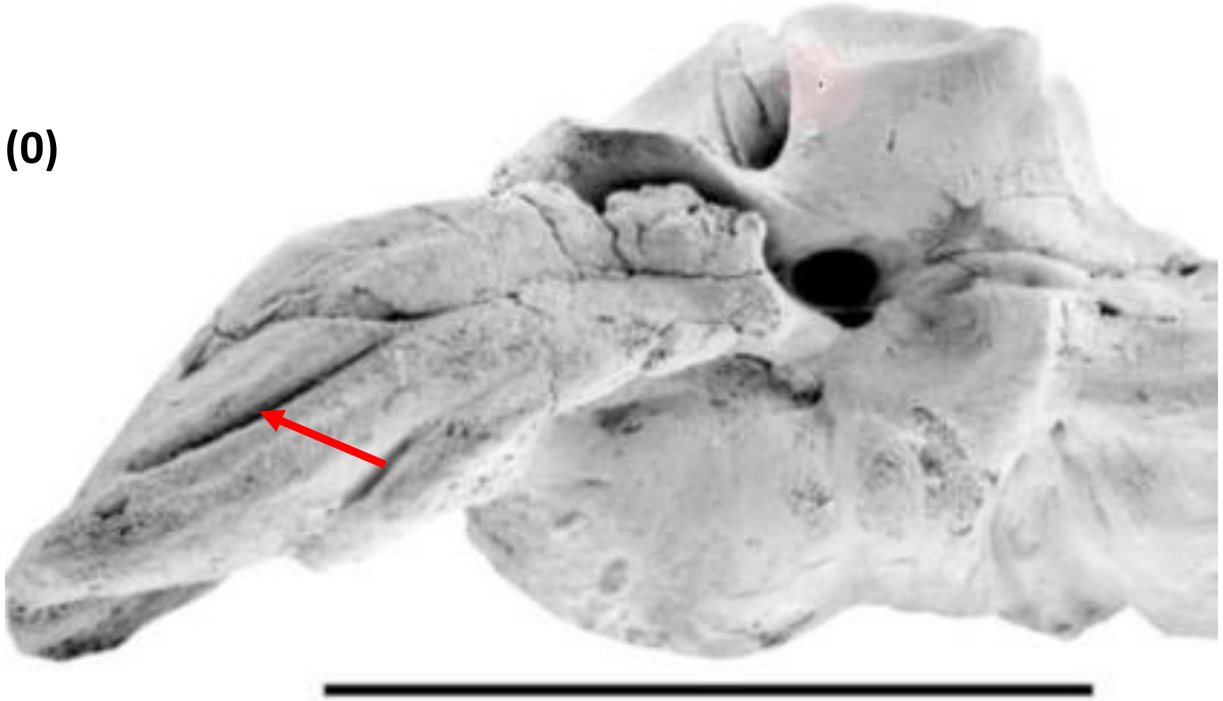

Whakakai waipata

Copyright holder: Cheng-Hsiu Tsai/University of Otago Geology Museum, Dunedin, New Zealand

(1)

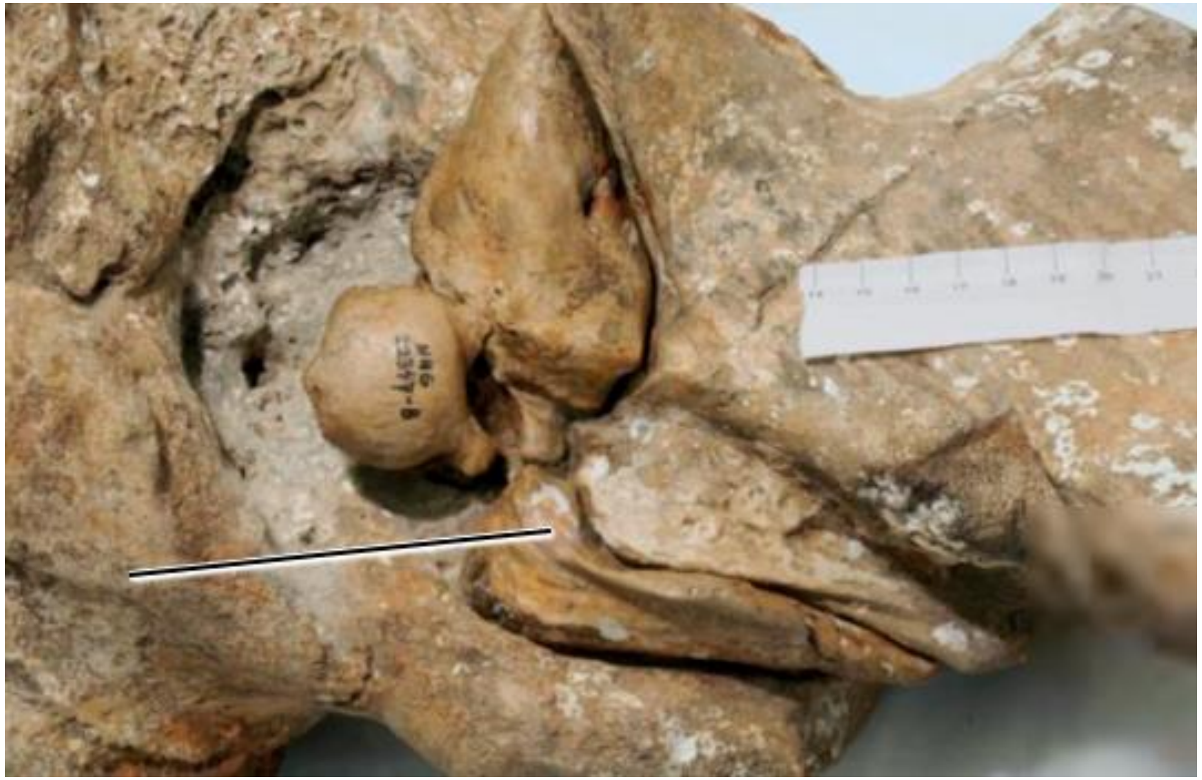

Diunatans luctoretemergo

Copyright holder: Felix G. Marx/ Natuurhistorische collectie van het Zeeuws Genootschap der Wetenschappen, Middelburg, the Netherlands

[184] 'Facial sulcus on compound posterior process'

(0) 'ventrally open'

(1) 'partially or entirely floored by a posteroventral flange'

(2) 'as state 1, but with the posteroventral flange being markedly enlarged'

(0)

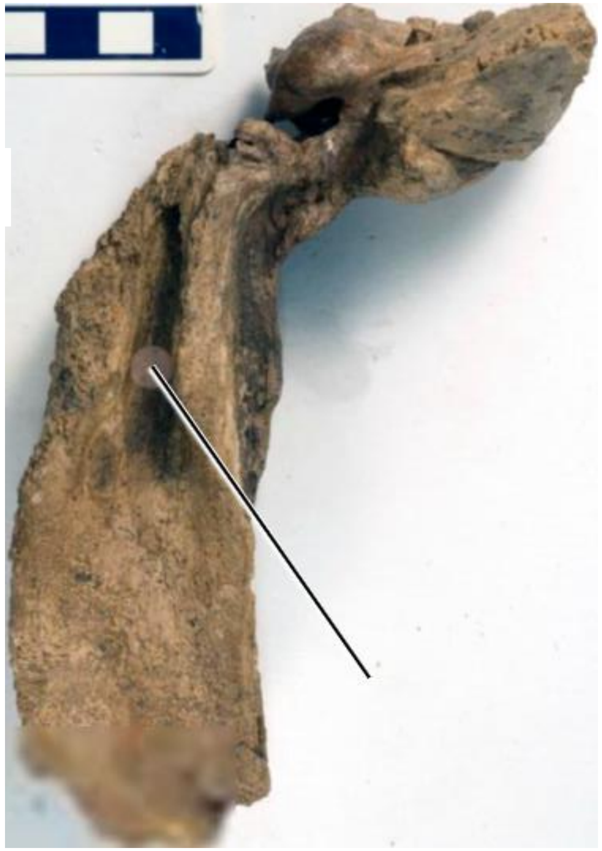

Thinocetus arthritus

Copyright holder: Felix G. Marx/ United States National Museum of Natural History, Washington DC, USA

(1)

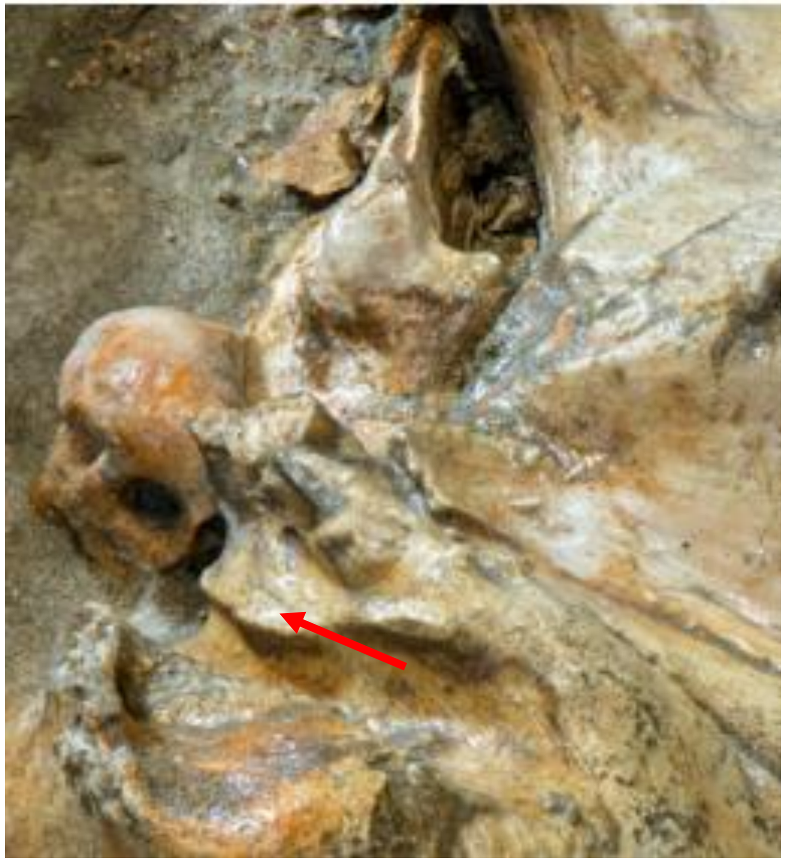

Tiucetus rosae

Adapted from: *"A new Miocene baleen whale from Peru deciphers the dawn of cetotheriids." Marx, Lambert, and De Muizon, 2017. Royal Society Open Science 4.9: 170560.*

(2)

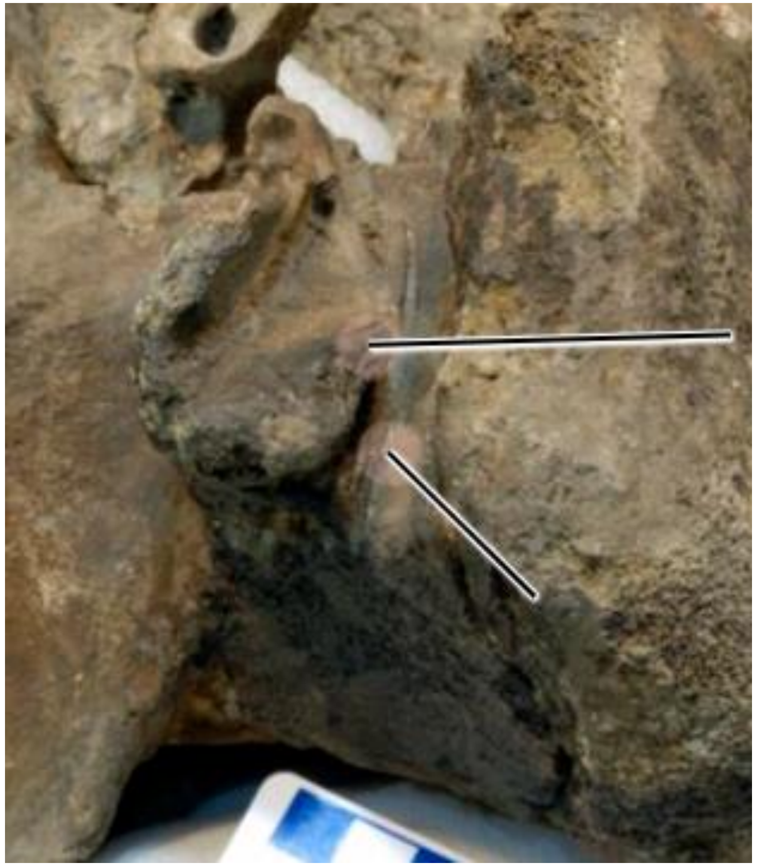

Metopocetus durinasus

Copyright holder: Felix G. Marx/United States National Museum of Natural History, Washington DC, USA

[185] 'Position of facial sulcus on compound posterior process in ventral view'

(0)'facial sulcus is posteriorly open and runs close to or along the posterior border of the compound posterior process'

(1)'facial sulcus located centrally on the ventral surface of the compound posterior process and posteriorly bounded by a distinct ridge'

(0)

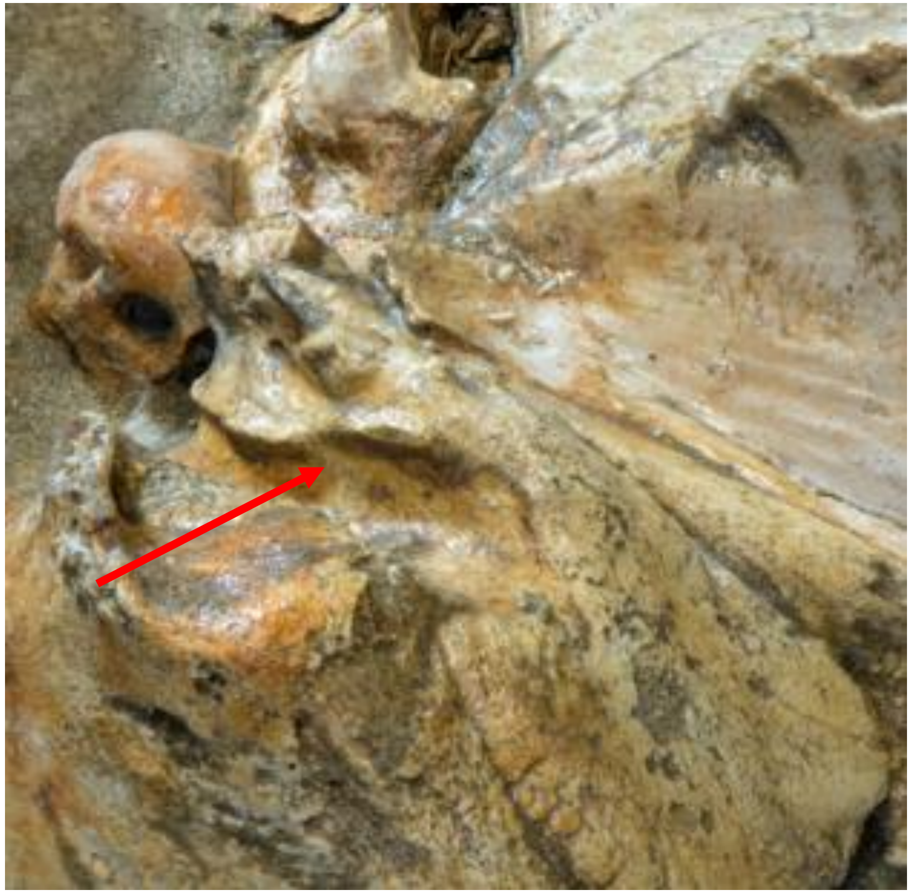

Tiucetus rosae

(1)

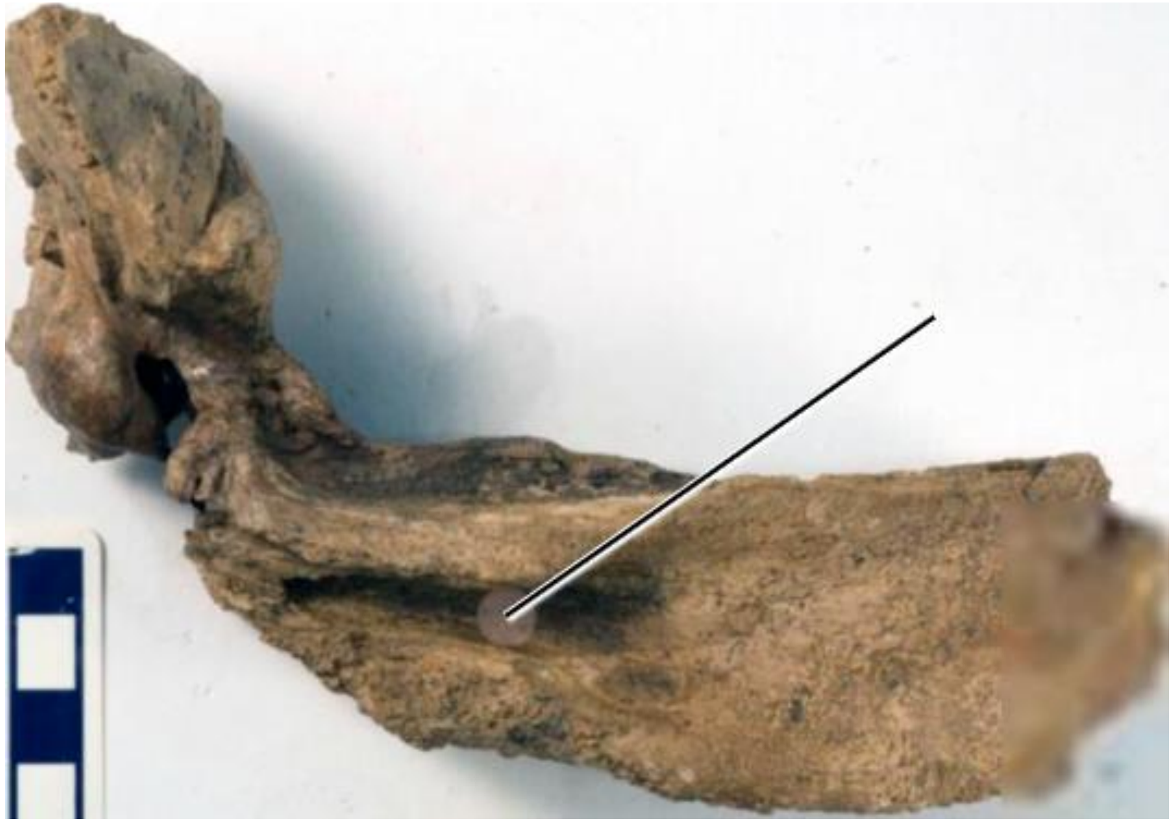

Thinocetus arthritus

Adapted from: *"A new Miocene baleen whale from Peru deciphers the dawn of cetotheriids." Marx, Lambert, and De Muizon, 2017. Royal Society Open Science 4.9: 170560.*

Copyright holder: Felix G. Marx/ United States National Museum of Natural History, Washington DC, USA

[186] 'Orientation of compound posterior process in ventral view, with periotic in situ'

(0)'oriented posterolaterally with respect to the longitudinal axis of the anterior process of the periotic'

(1)'oriented at a right angle to the axis of the anterior process'

(0)

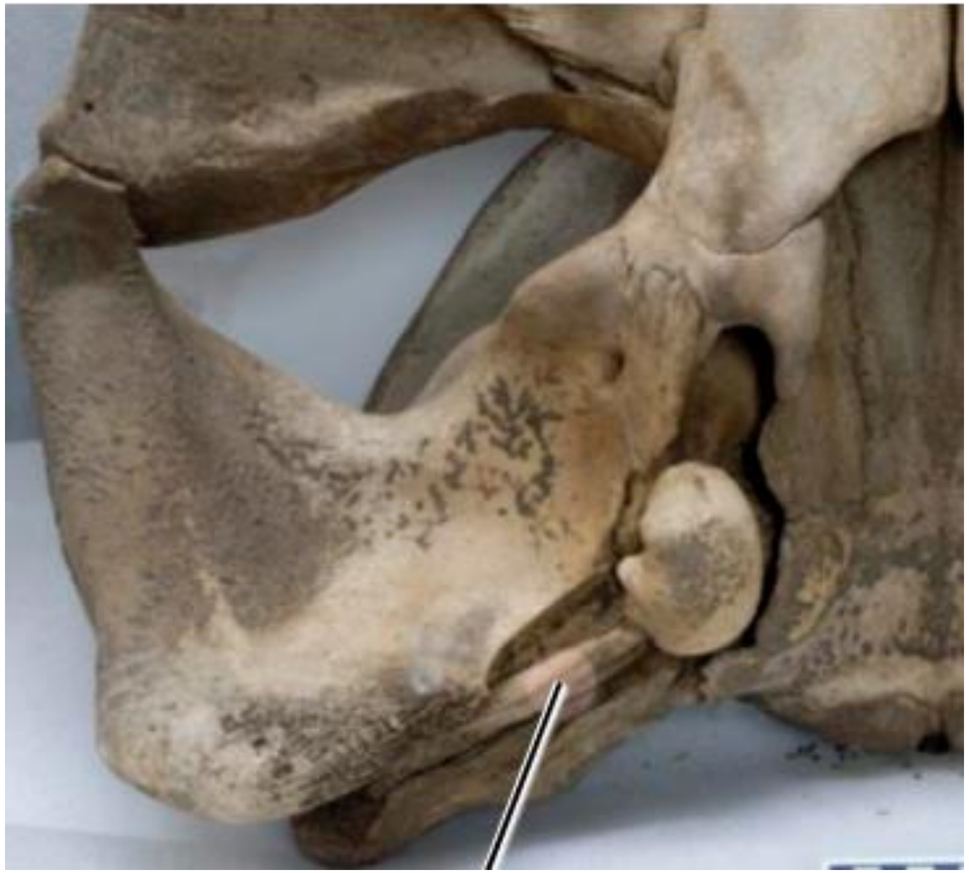

Balaenoptera acutorostrata

Copyright holder: Felix G. Marx/ The Charleston Museum, Charleston, South Carolina, USA

(1)

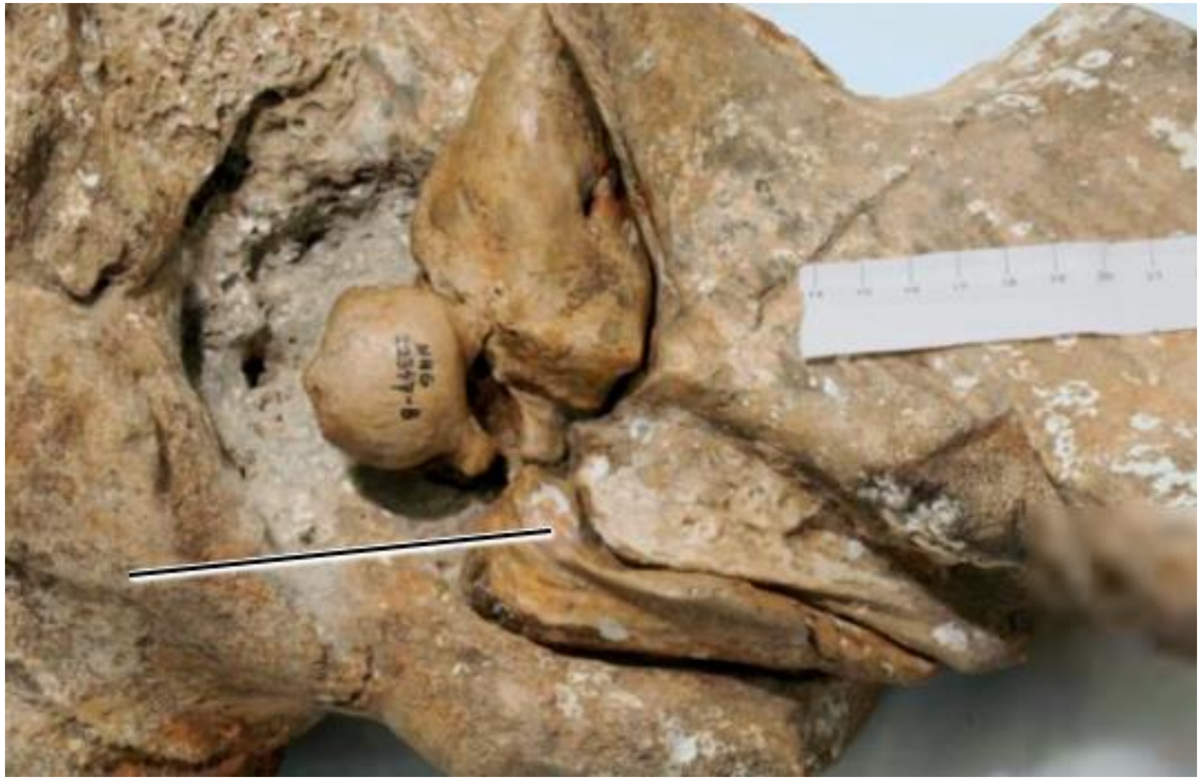

Diunatans luctoretemergo

Copyright holder: Felix G. Marx/ Natuurhistorische collectie van het Zeeuws Genootschap der Wetenschappen, Middelburg, the Netherlands

[187] 'Shape of compound posterior process'

(0)'cylindrical or slightly conical'

(1)'short and stocky'

(2)'flattened anteroposteriorly'

(3)'forms a distinct plug'

(0)

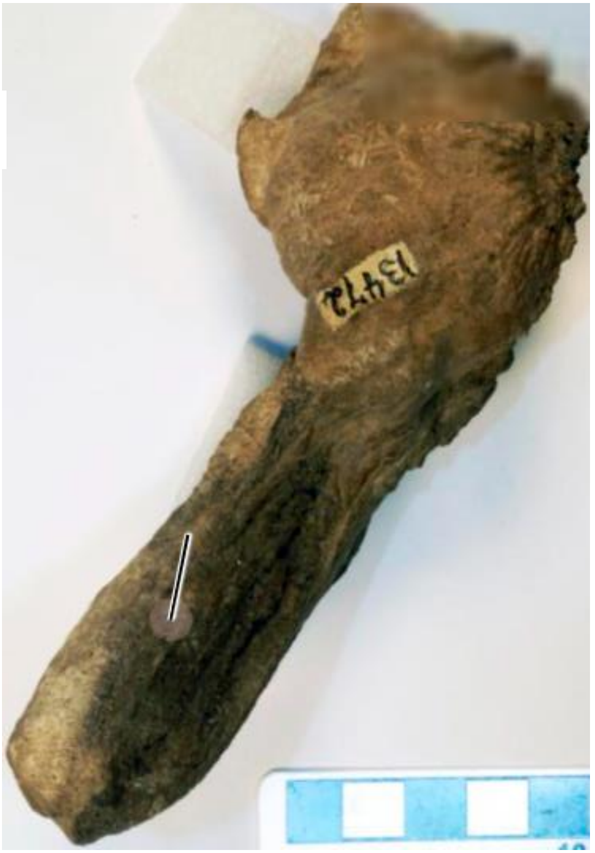

Aglaocetus patulus

Copyright holder: Felix G. Marx/ United States National Museum of Natural History, Washington DC, USA

(1)

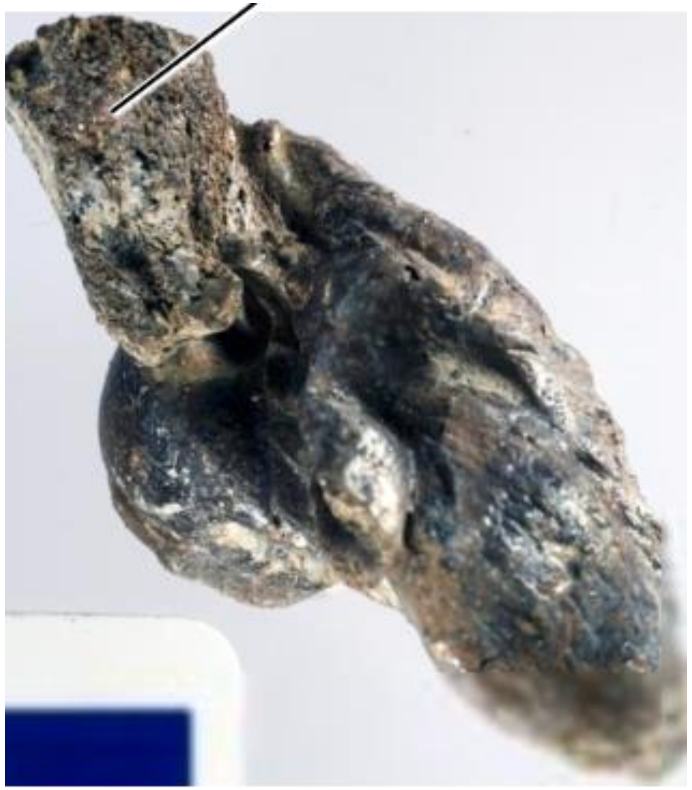

Fucaia buelli

Copyright holder: Felix G. Marx/ Burke Museum, University of Washington, Seattle, USA

(2)

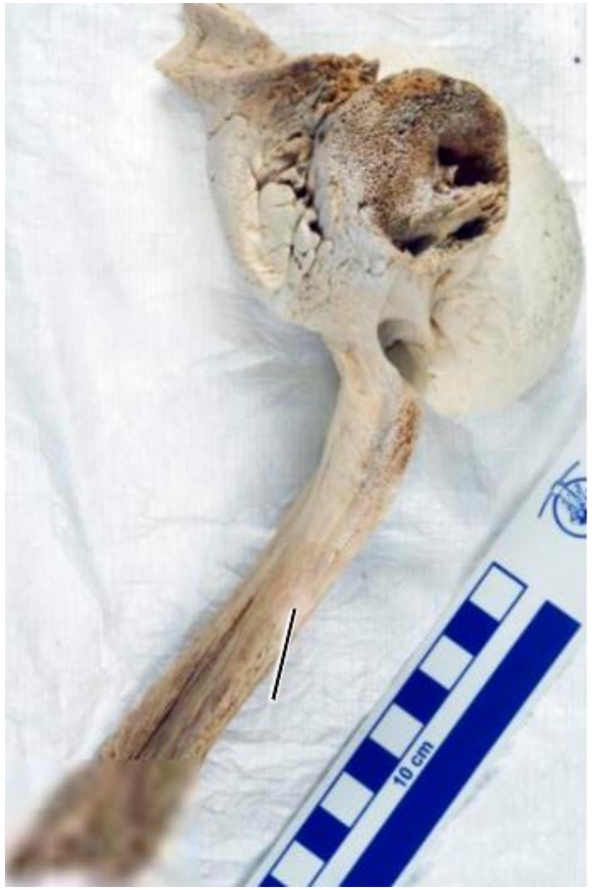

Balaenoptera acutorostrata

Copyright holder: Felix G. Marx/ National Museum of Nature and Science, Tokyo, Japan

[187] 'Shape of compound posterior process'

(0)'cylindrical or slightly conical'

(1)'short and stocky'

(2)'flattened anteroposteriorly'

(3)'forms a distinct plug'

(3)

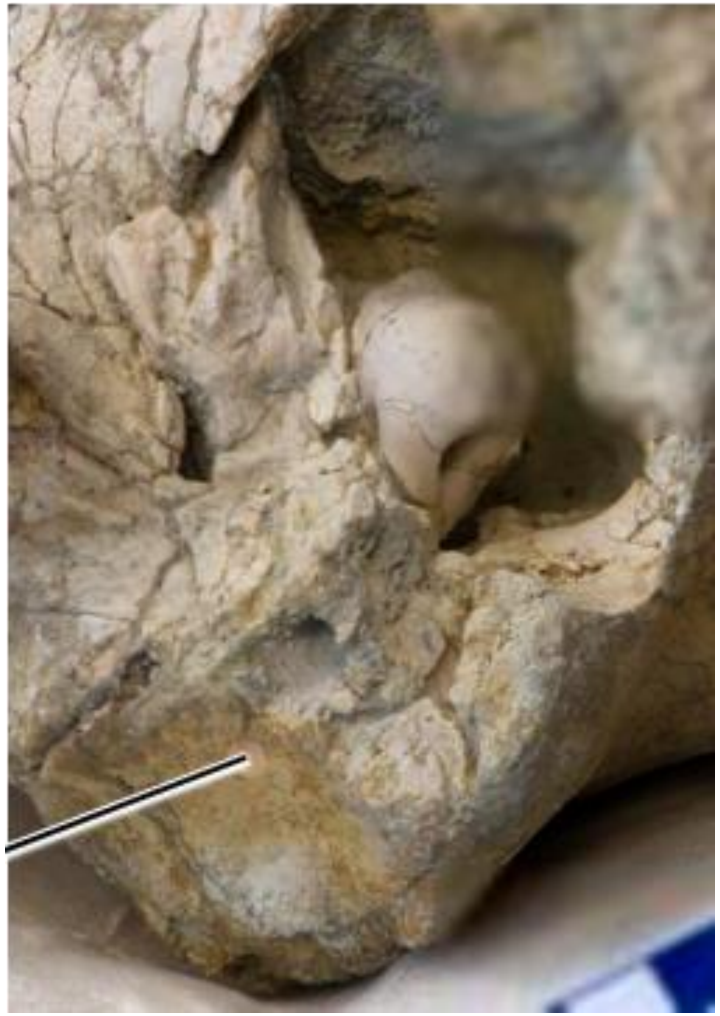

Herpetocetus morrowi

Copyright holder: Felix G. Marx/ University of California Museum of Paleontology, Berkeley, USA

[188] 'Exposure of compound posterior process on lateral skull wall'

(0)'external surface of compound posterior process is absent or poorly defined’

(1)'external surface is present but distinct from lateral skull wall’

(2)'external surface is expanded and firmly integrated into the lateral skull wall’

(3)'as state 2, but with the external surface of the posterior process being oriented at a 90 degree angle relative to its ventral surface'

(0)

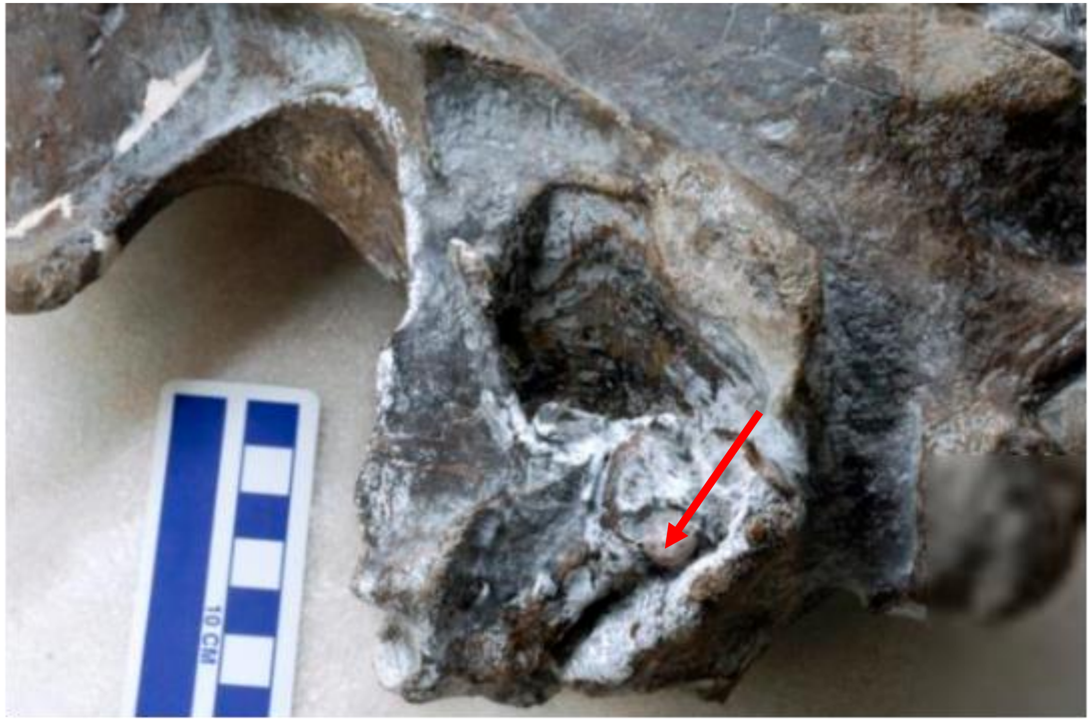

Fucaia goedertorum

Copyright holder: Felix G. Marx/Natural History Museum of Los Angeles County, Los Angeles, USA

(1)

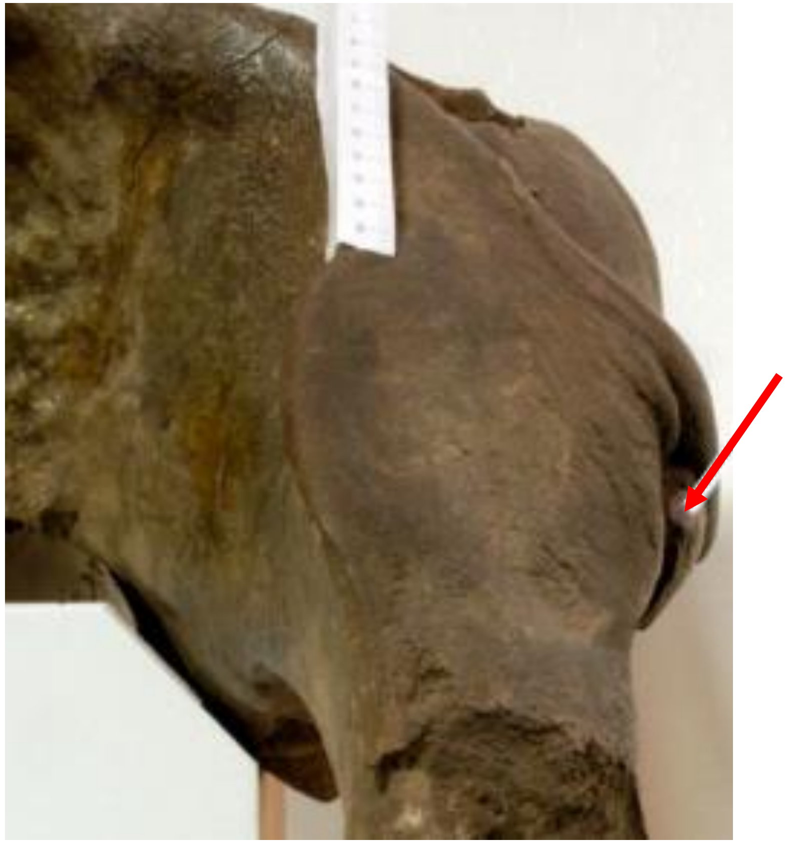

Balaenella brachyrhynus

Copyright holder: Felix G. Marx/Natuurmuseum Brabant, Tilburg, the Netherlands

[188] 'Exposure of compound posterior process on lateral skull wall'

(0)'external surface of compound posterior process is absent or poorly defined'

(1)'external surface is present but distinct from lateral skull wall'

(2)'external surface is expanded and firmly integrated into the lateral skull wall'

(3)'as state 2, but with the external surface of the posterior process being oriented at a 90 degree angle relative to its ventral surface'

(2)

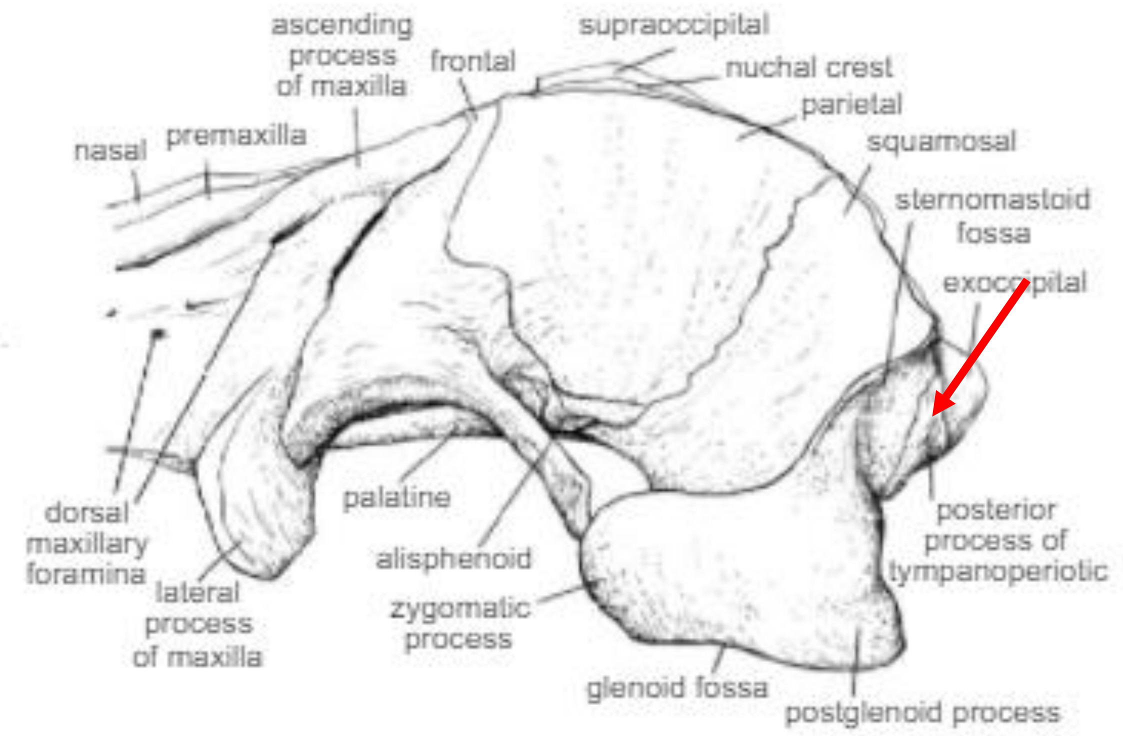

Cetotherium riabinini

Adapted from: "The anatomy of the Late Miocene baleen whale Cetotherium riabinini from Ukraine." Gol'din et al., 2013. *Acta Palaeontologica Polonica* 59.4: 795-814.

(3)

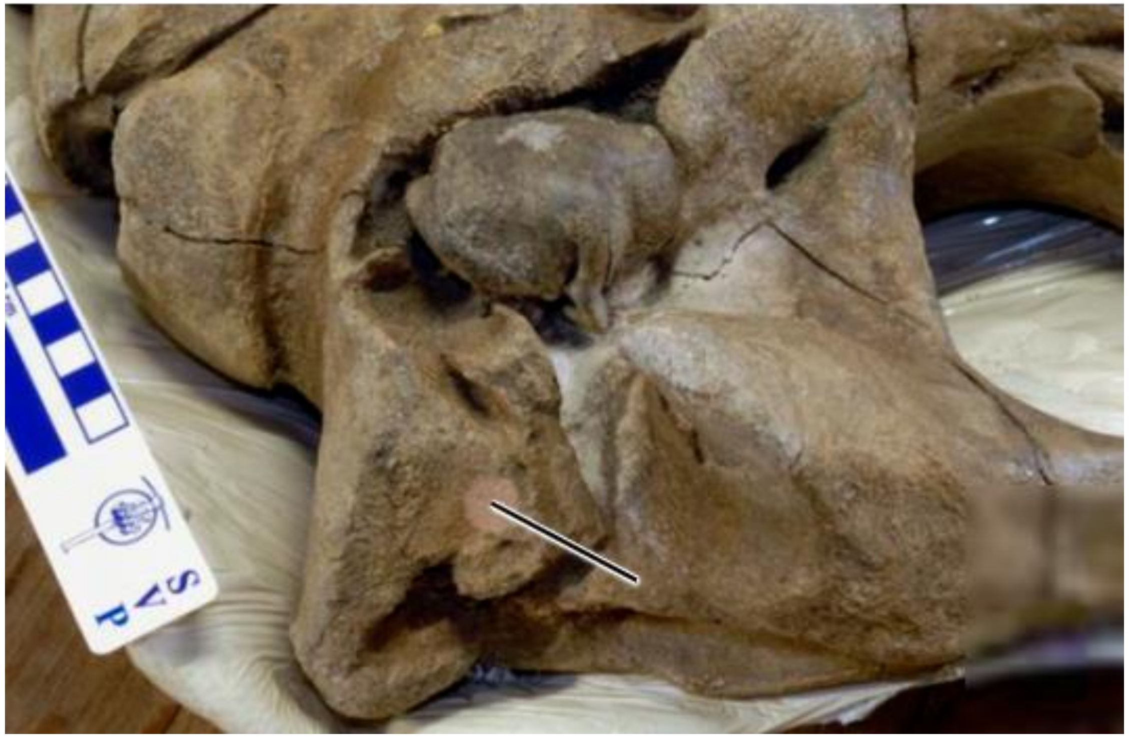

Piscobalaena nana

Copyright holder: Felix G. Marx/Museum National d'Histoire Naturelle, Paris, France

[189] 'Bony texture of ventral surface of compound posterior process'

(0) 'massive'

(1) 'fibrous'

(0)

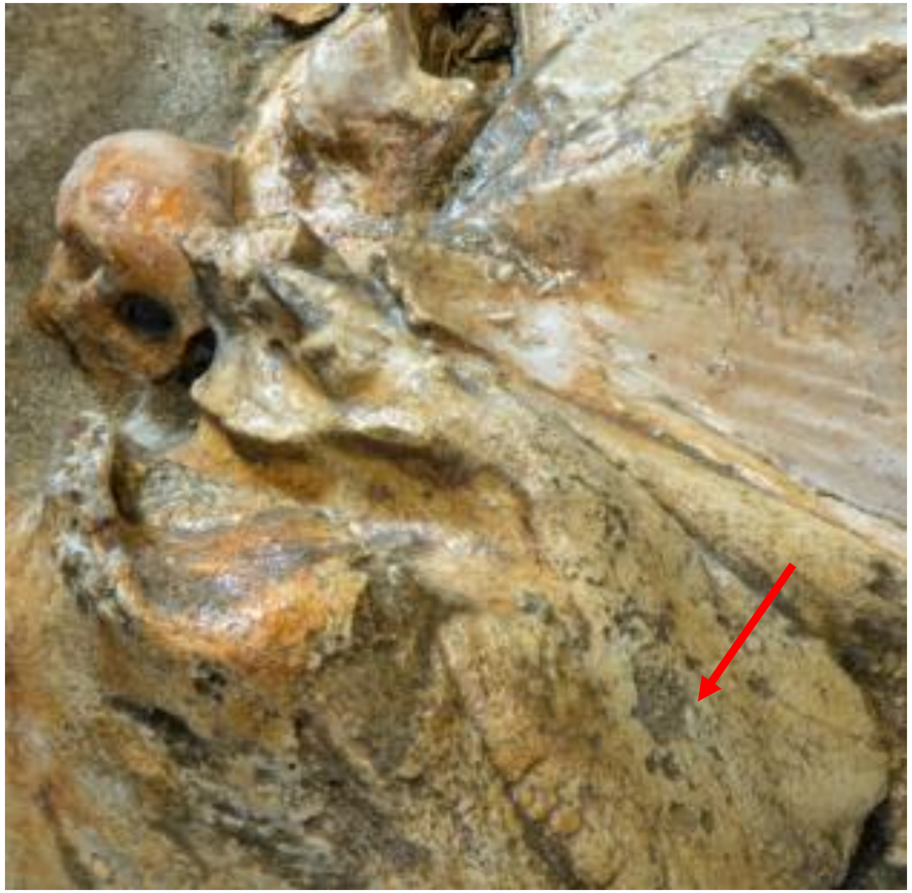

Tiucetus rosae

Adapted from: *"A new Miocene baleen whale from Peru deciphers the dawn of cetotheriids." Marx, Lambert, and De Muizon, 2017. Royal Society Open Science 4.9: 170560.*

(1)

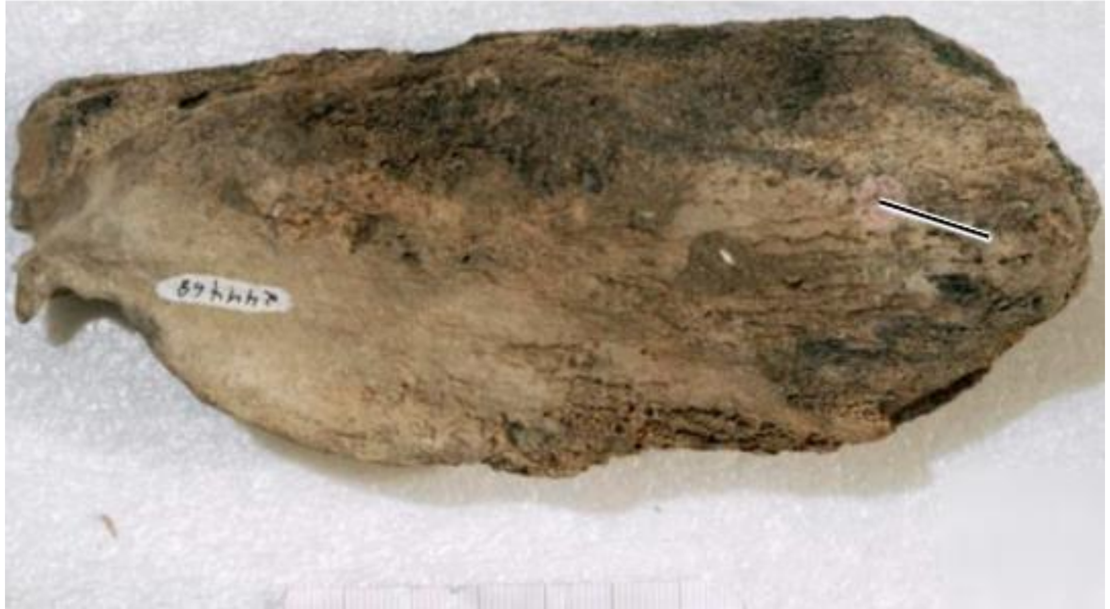

Gricetoides aurorae

Copyright holder: Felix G. Marx/ United States National Museum of Natural History, Washington DC, USA

[190] 'Neck of compound posterior process markedly constricted'

(0) 'absent'

(1) 'present'

(0)

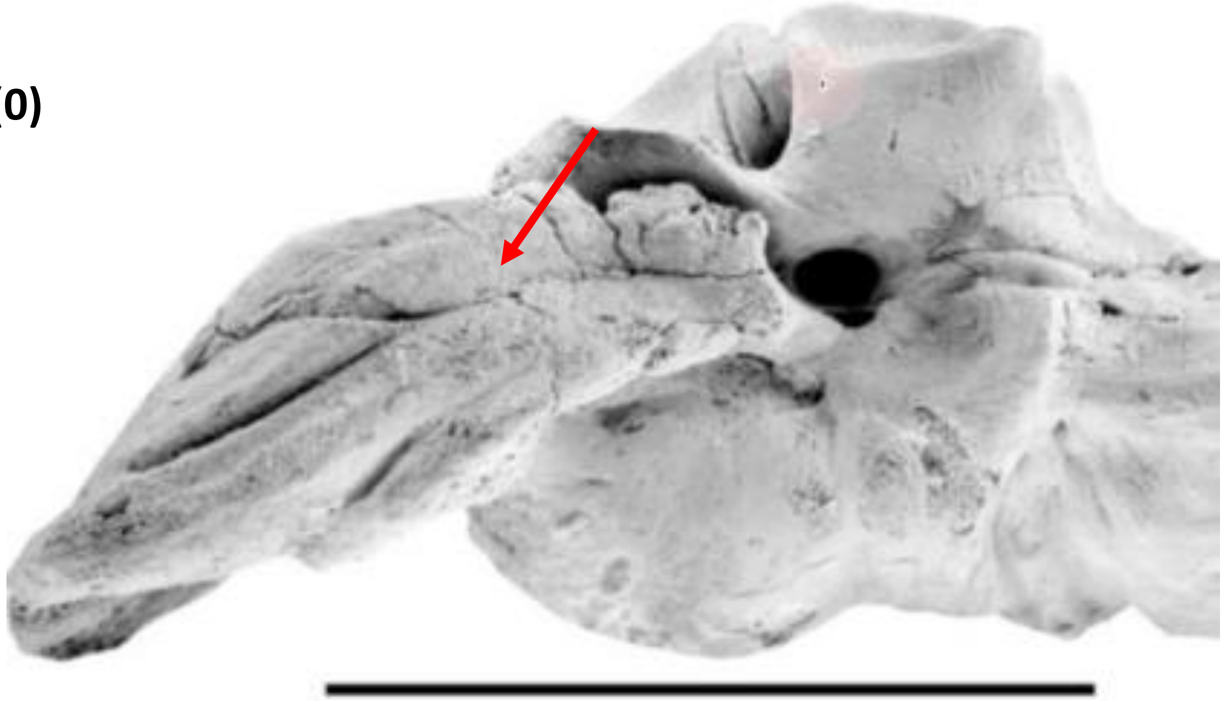

Whakakai waipata

Copyright holder: Cheng-Hsiu Tsai/University of Otago Geology Museum, Dunedin, New Zealand

(1)

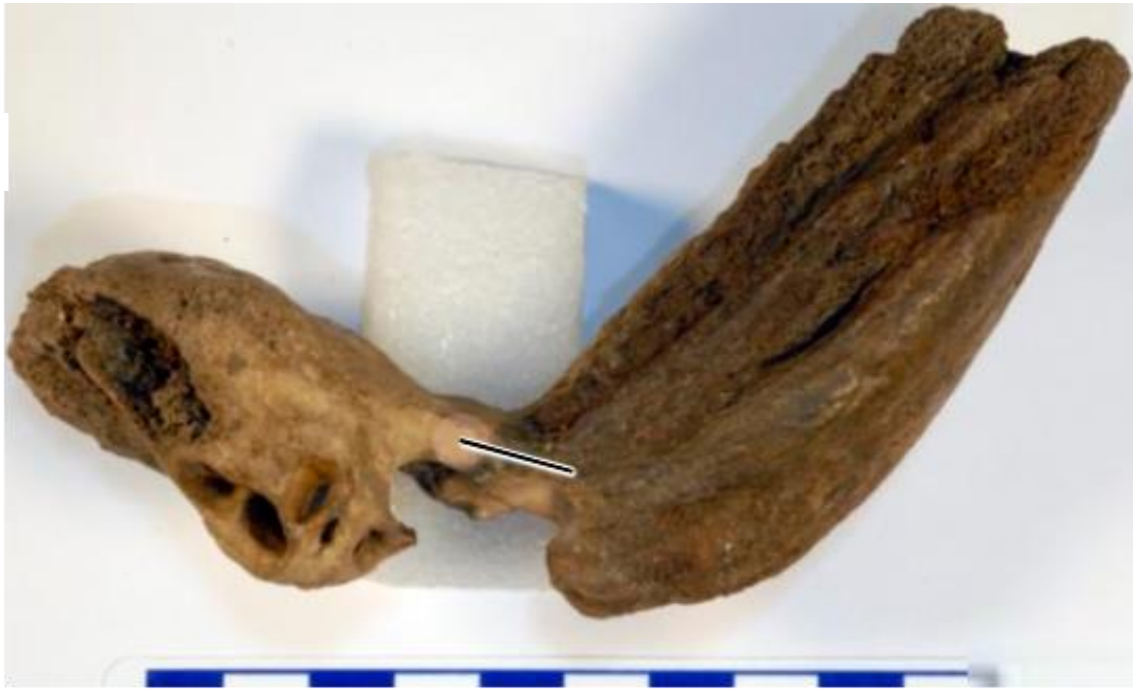

Diorocetus hiatus

Copyright holder: Felix G. Marx/ United States National Museum of Natural History, Washington DC, USA

[191] 'Anterior border of bulla in dorsal or ventral view'

(0)'obliquely truncated'

(1)'squared'

(2)'rounded'

(3)'pointed'

(0)

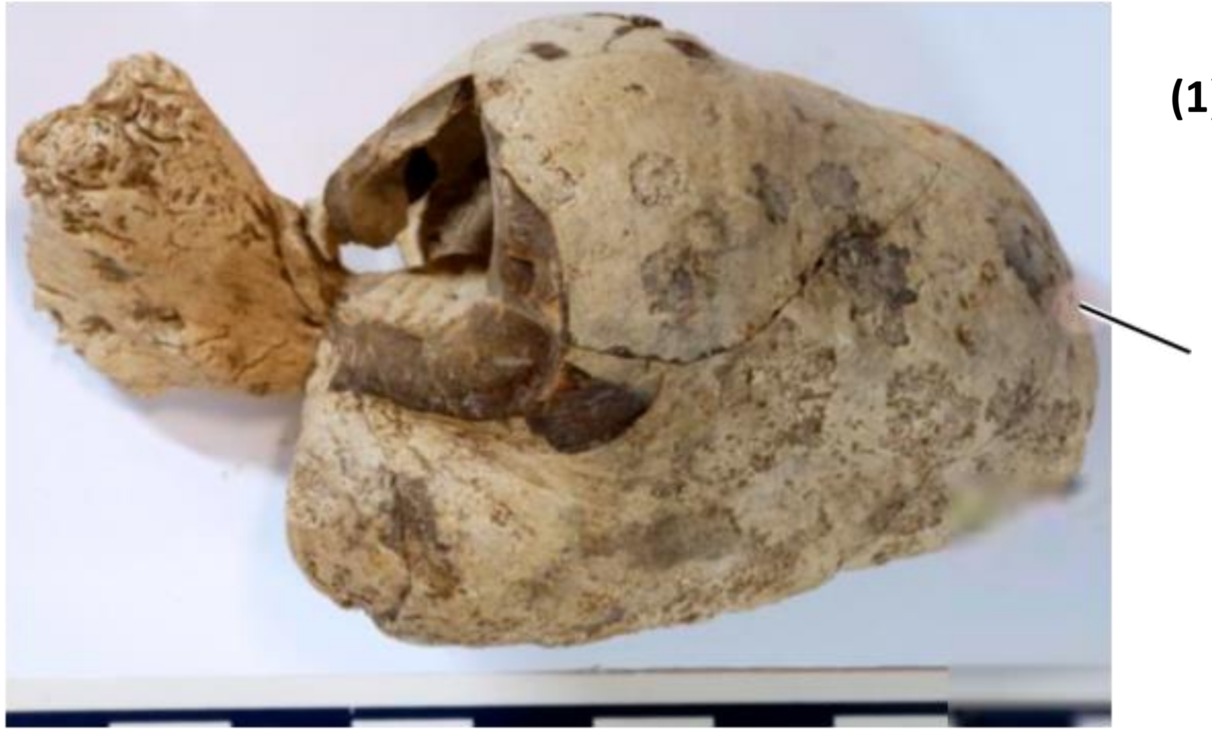

Eomysticetus whitmorei

Copyright holder: Felix G. Marx/ The Charleston Museum, Charleston, South Carolina, USA

(1)

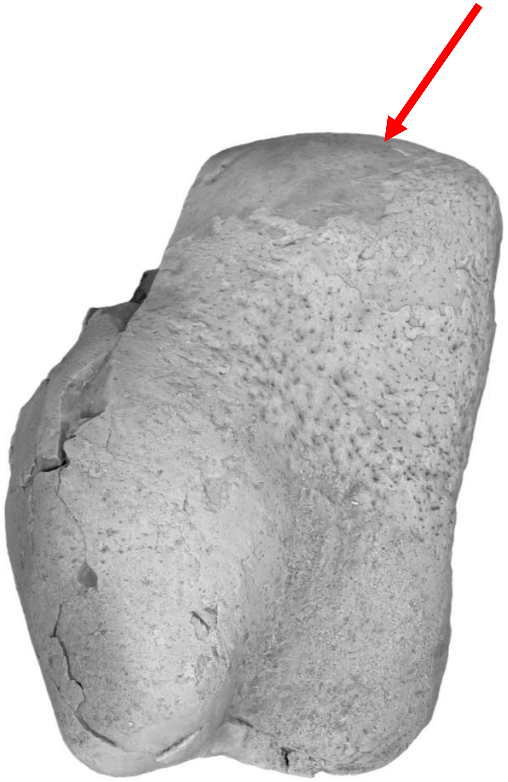

Mammalodon colliveri

Copyright holder: Erich M. G. Fitzgerald/ Museums Victoria, Melbourne, Australia

[191] 'Anterior border of bulla in dorsal or ventral view'

(0)'obliquely truncated'

(1)'squared'

(2)'rounded'

(3)'pointed'

(2)

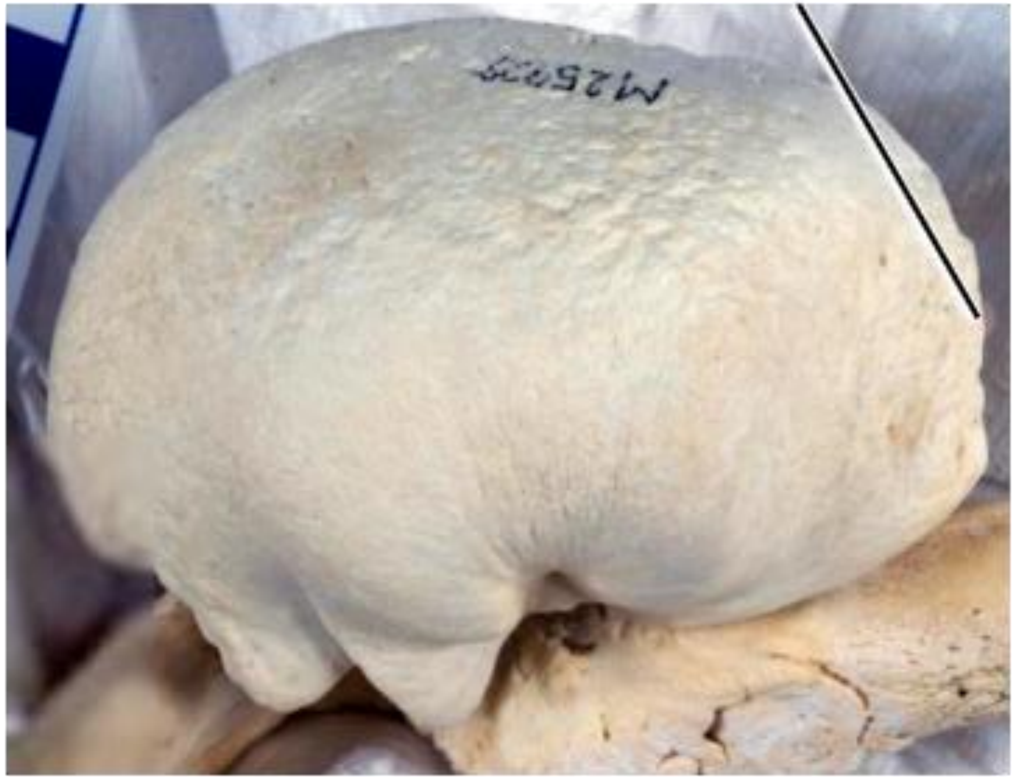

Balaenoptera acutorostrata

Copyright holder: Felix G. Marx, National Museum of Nature and Science, Tokyo, Japan

(3)

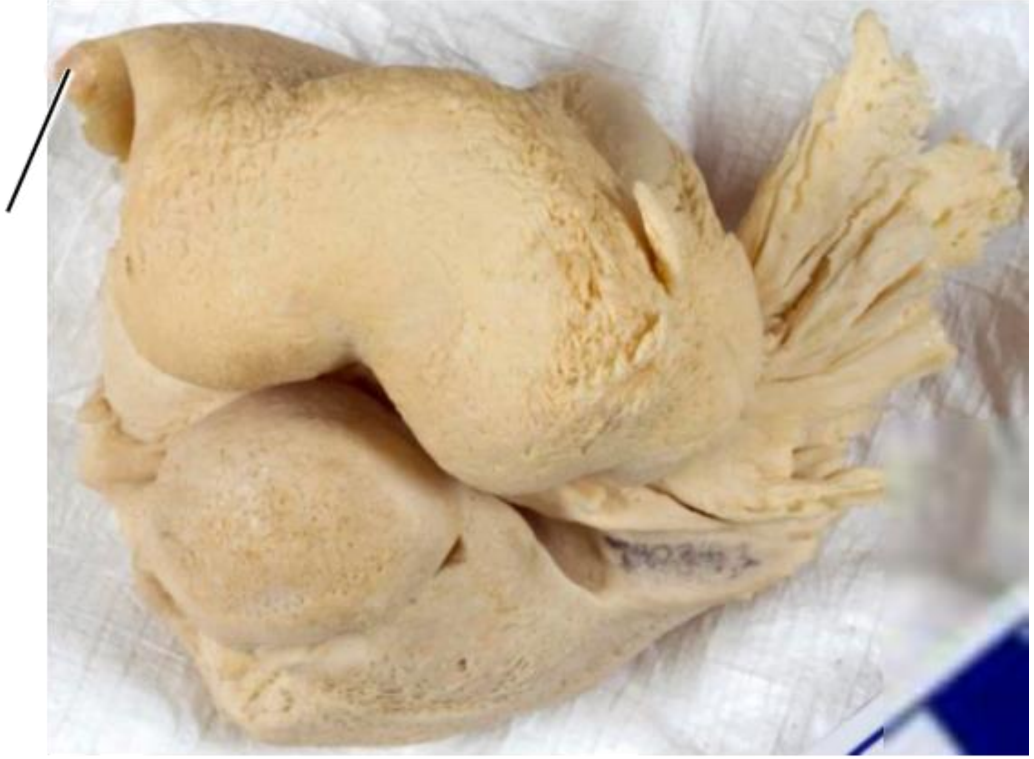

Physeter macrocephalus

Copyright holder: Felix G. Marx, National Museum of Nature and Science, Tokyo, Japan

[192] 'Anterior portion of bulla transversely wider than posterior portion in ventral view'

(0)'absent'

(1)'present'

(0)

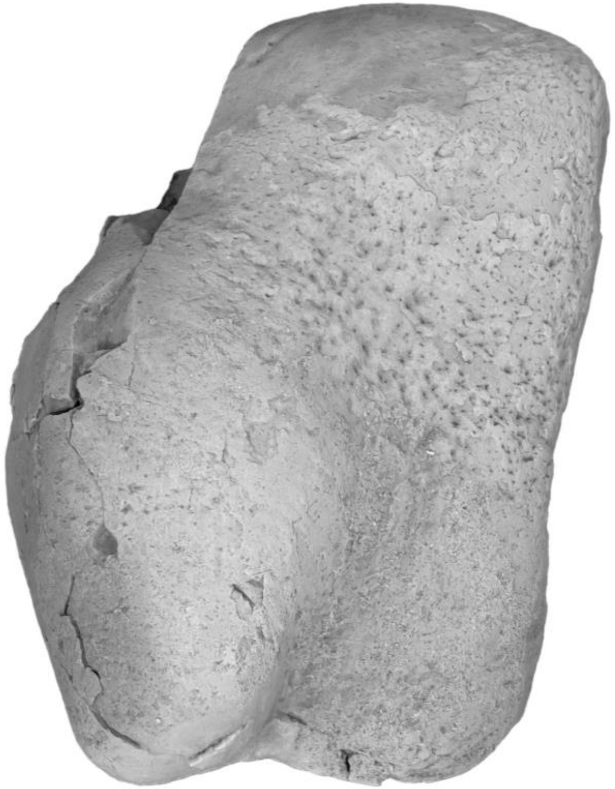

Mammalodon colliveri

Copyright holder: Erich M. G. Fitzgerald/ Museums Victoria, Melbourne, Australia

(1)

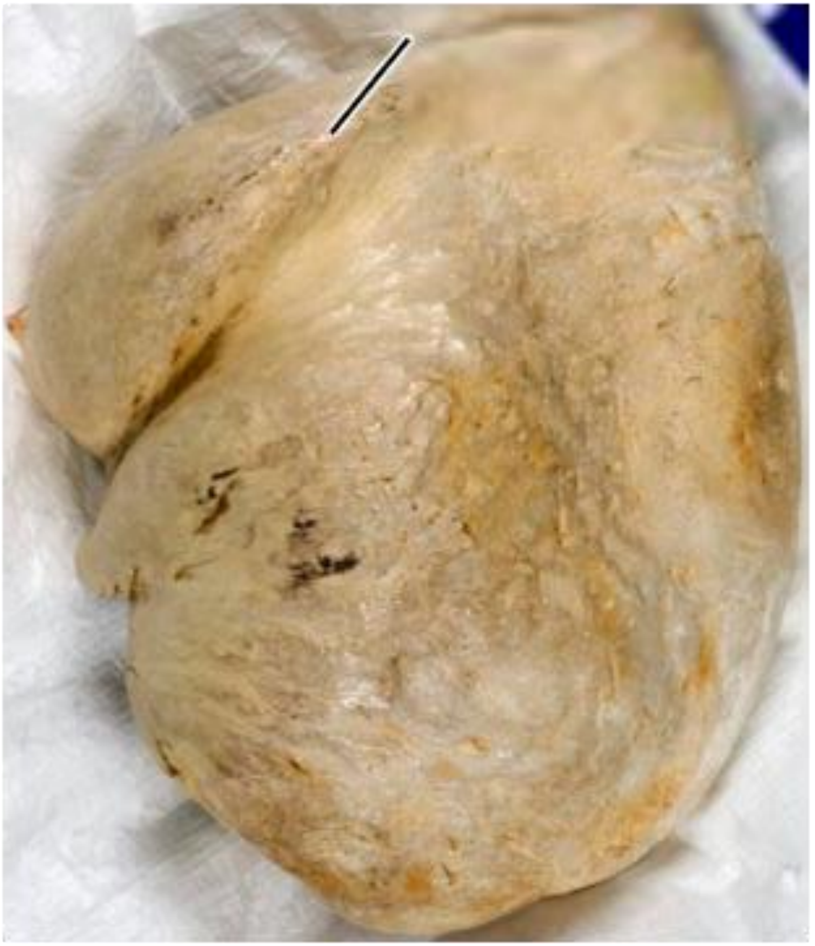

Balaena mysticetus

Copyright holder: Felix G. Marx/ National Museum of Nature and Science, Tokyo, Japan

[193] 'In situ orientation of main axes of tympanic bullae in ventral view'

(0)'diverging posteriorly'

(1)'parallel'

(2)'diverging anteriorly'

(0)

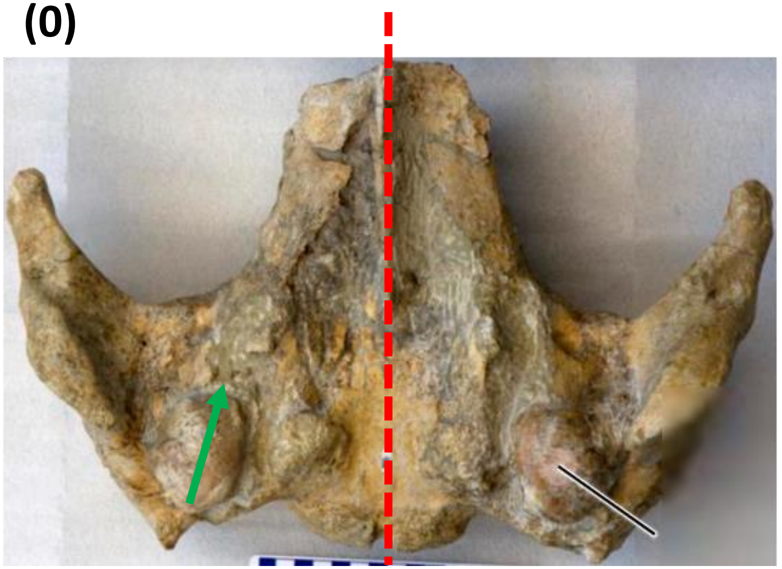

Herpetocetus morrowi

Copyright holder: Felix G. Marx/ San Diego Museum of Natural History, San Diego, USA

(1)

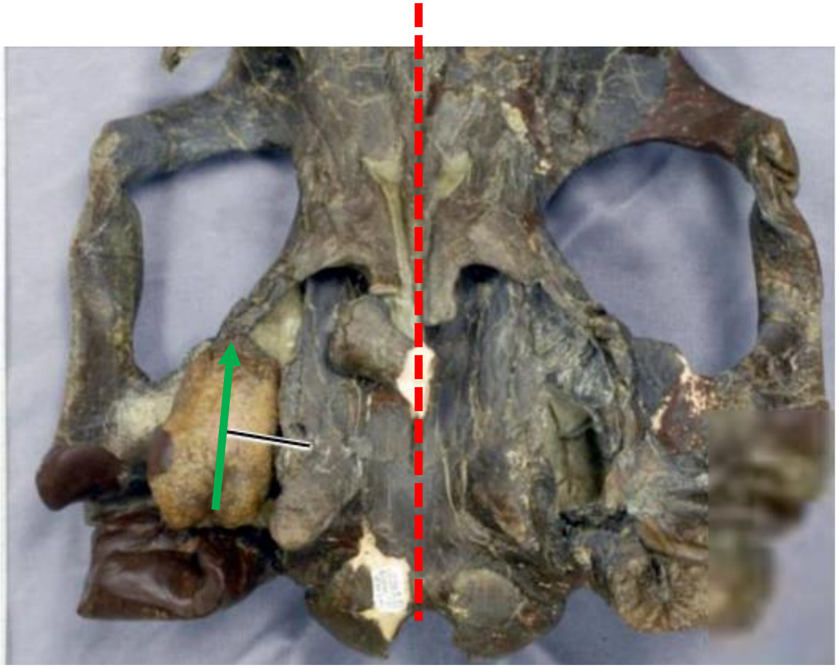

Aetiocetus weltoni

Copyright holder: Felix G. Marx/ University of California Museum of Paleontology, Berkeley, USA

(2)

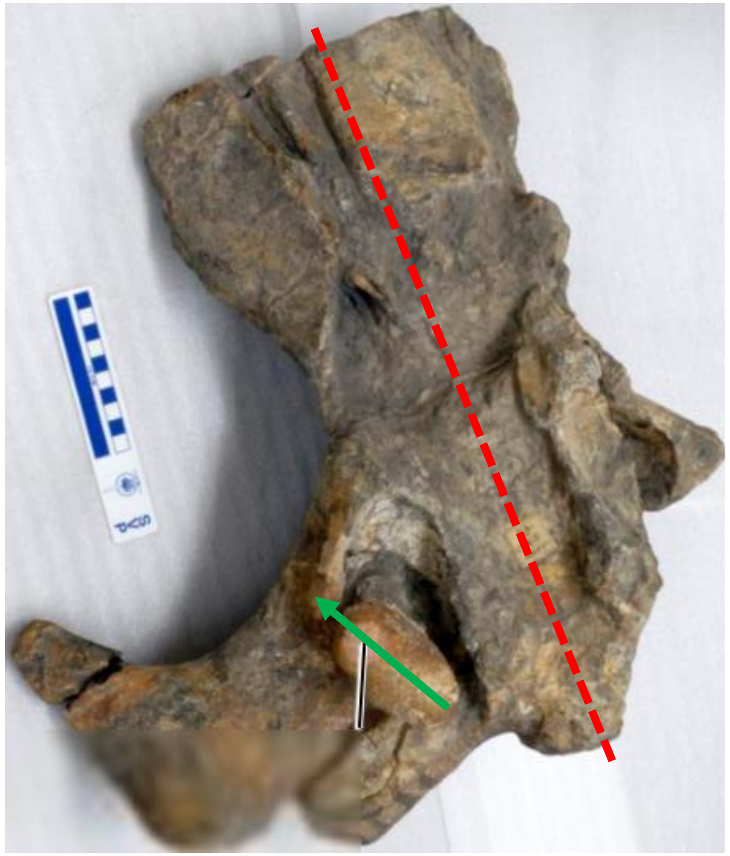

Tiphyocetus temblorensis

Copyright holder: Felix G. Marx/ California Academy of Sciences, San Francisco, USA

[194] 'Position of dorsal origin of lateral furrow'

(0)'located along posterior two thirds of the anteroposterior length of the bulla'

(1)'located at roughly one third of the anteroposterior length of the bulla'

(0)

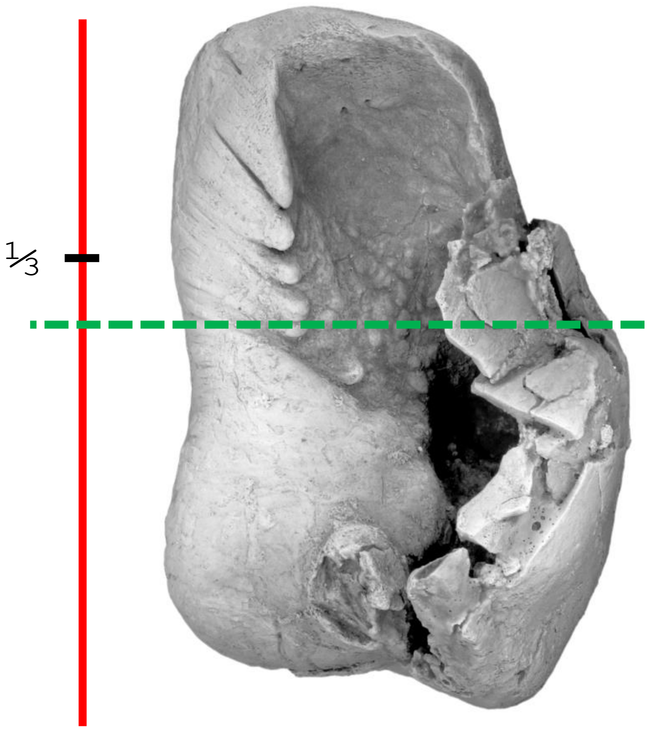

Mammalodon colliveri

Copyright holder: Erich M. G. Fitzgerald/ Museums Victoria, Melbourne, Australia

(1)

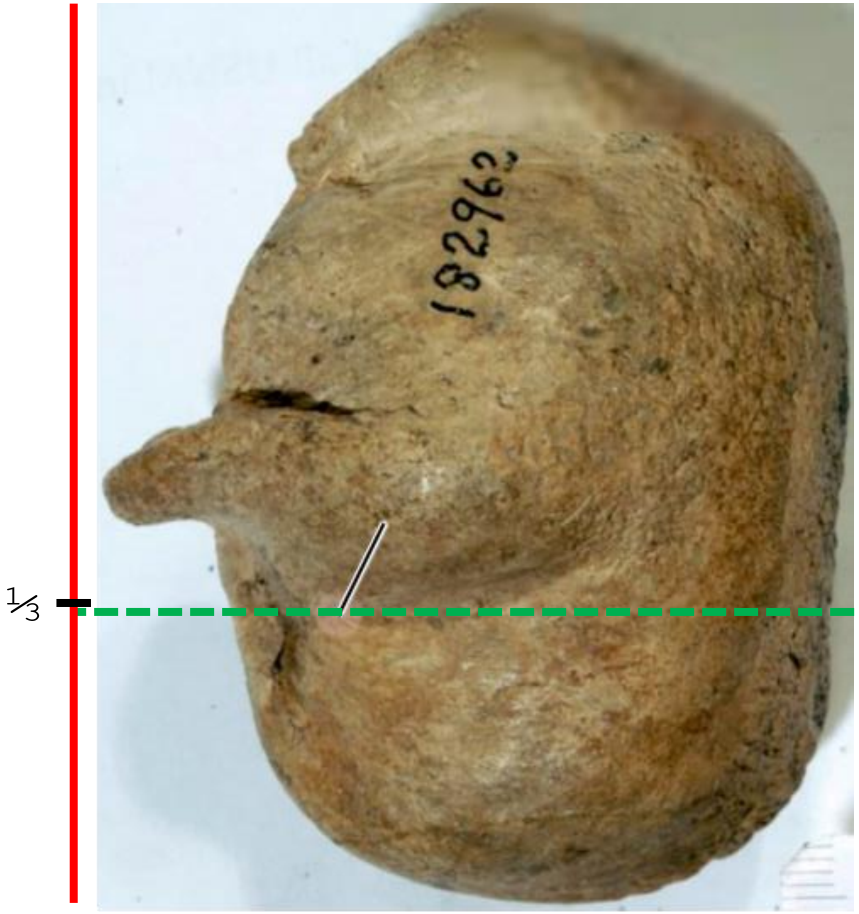

Herpetocetus transatlanticus

Copyright holder: Felix G. Marx/ United States National Museum of Natural History, Washington DC, USA

[195] 'Orientation of lateral furrow in lateral view'

(0) 'ventral'

(1) 'distinctly anteroventral'

(0)

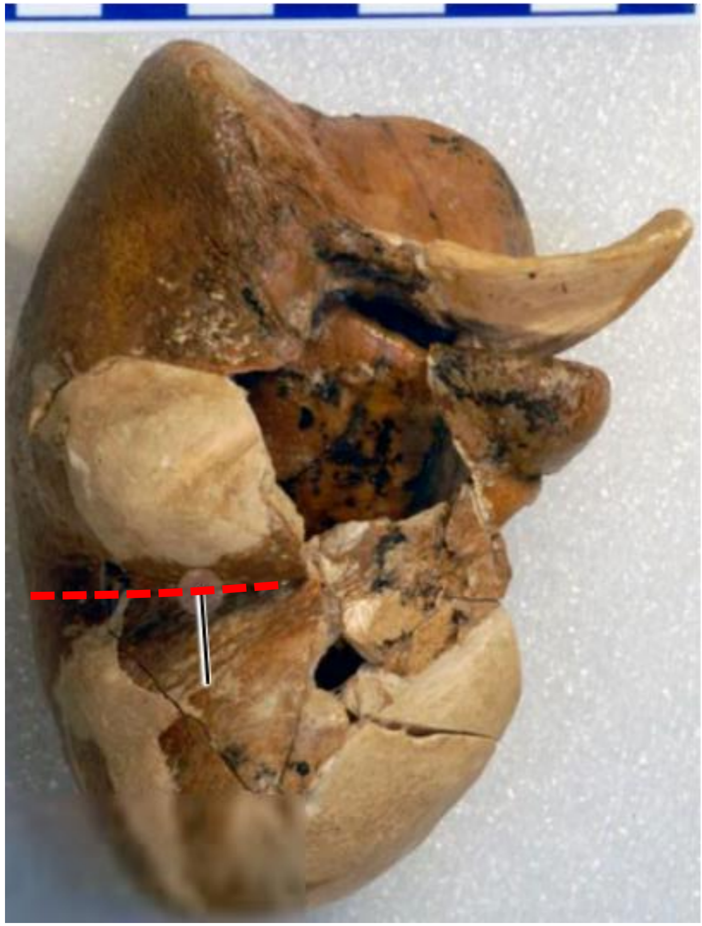

Megaptera miocaena

Copyright holder: Felix G. Marx/United States National Museum of Natural History, Washington DC, USA

(1)

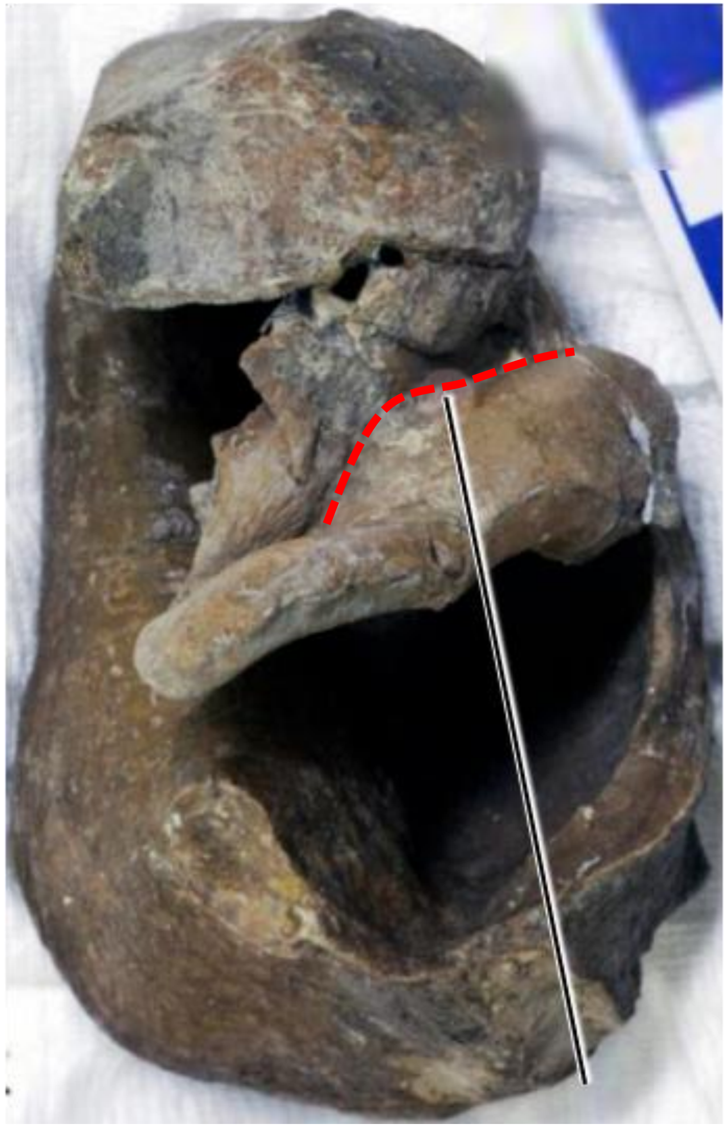

Balaenula sp.

Copyright holder: Felix G. Marx/ Sapporo Museum Activity Centre, Sapporo, Japan

[196] 'Orientation of ventral keel of outer posterior prominence'

(0)'faces ventrally'

(1)'faces ventromedially or medially'

(0)

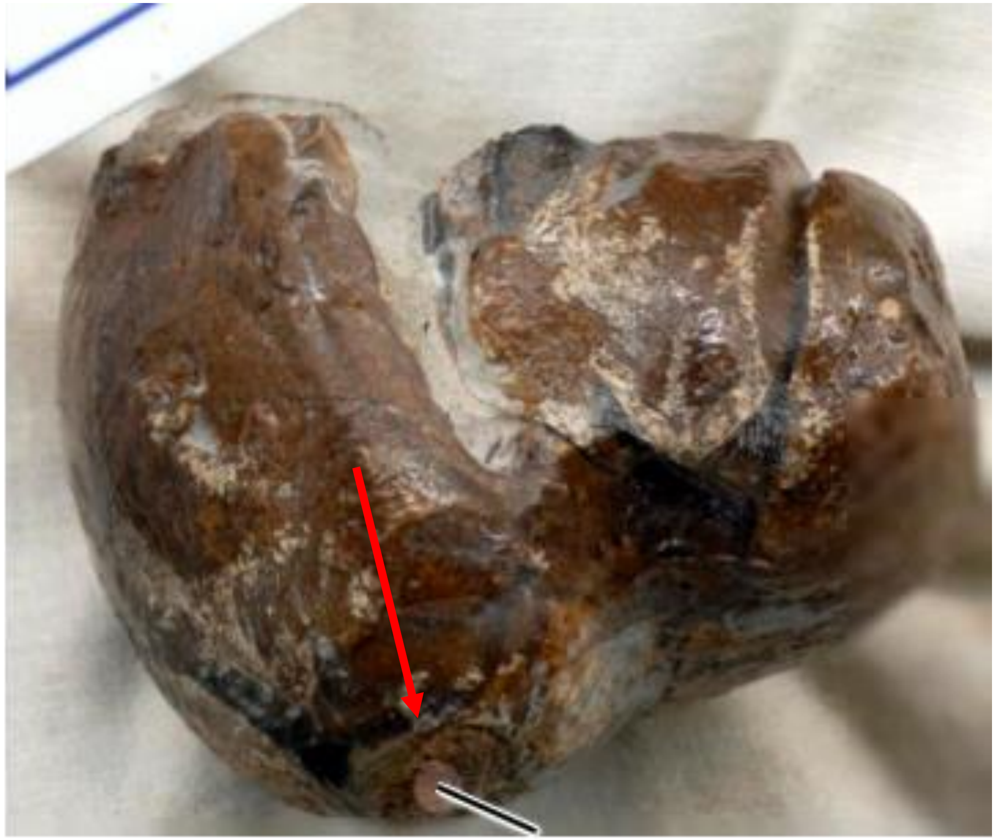

Fucaia goedertorum

Copyright holder: Felix G. Marx/Natural History Museum of Los Angeles County, Los Angeles, USA

(1)

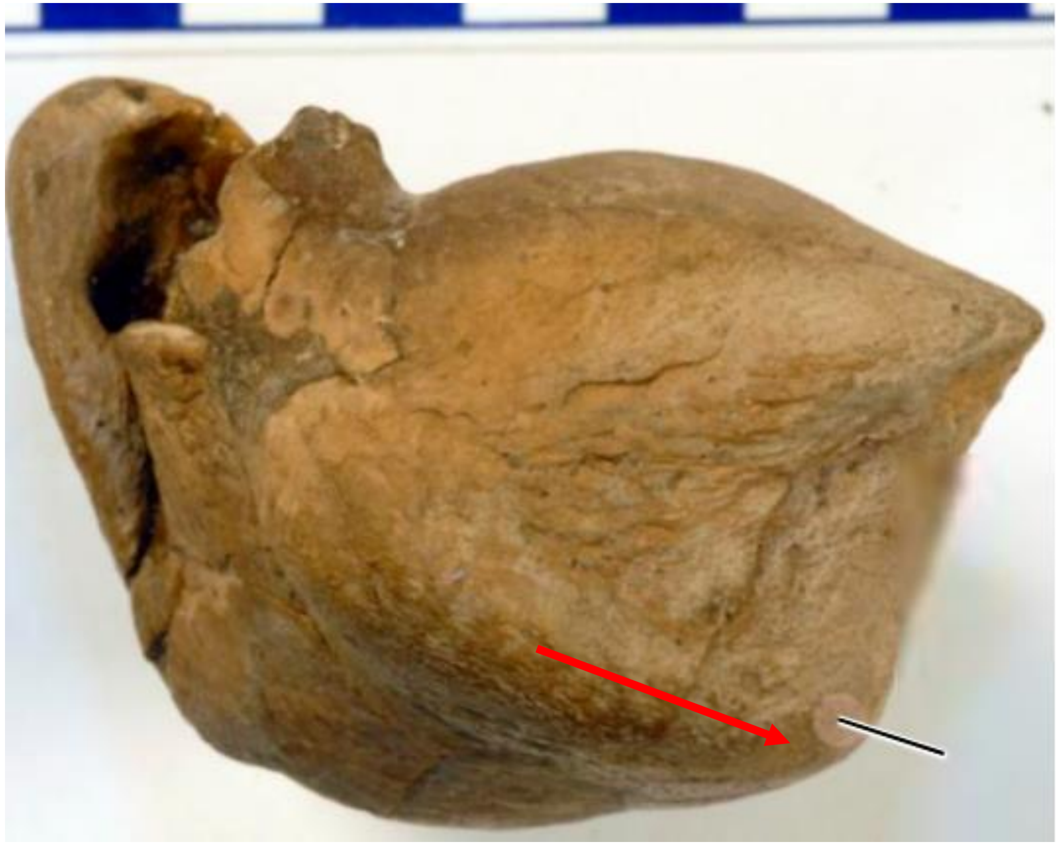

Aglaocetus patulus

Copyright holder: Felix G. Marx/ United States National Museum of Natural History, Washington DC, USA

[197] 'Anteroposterior outline of main ridge'

(0) 'concave'

(1) 'straight or convex'

(0)

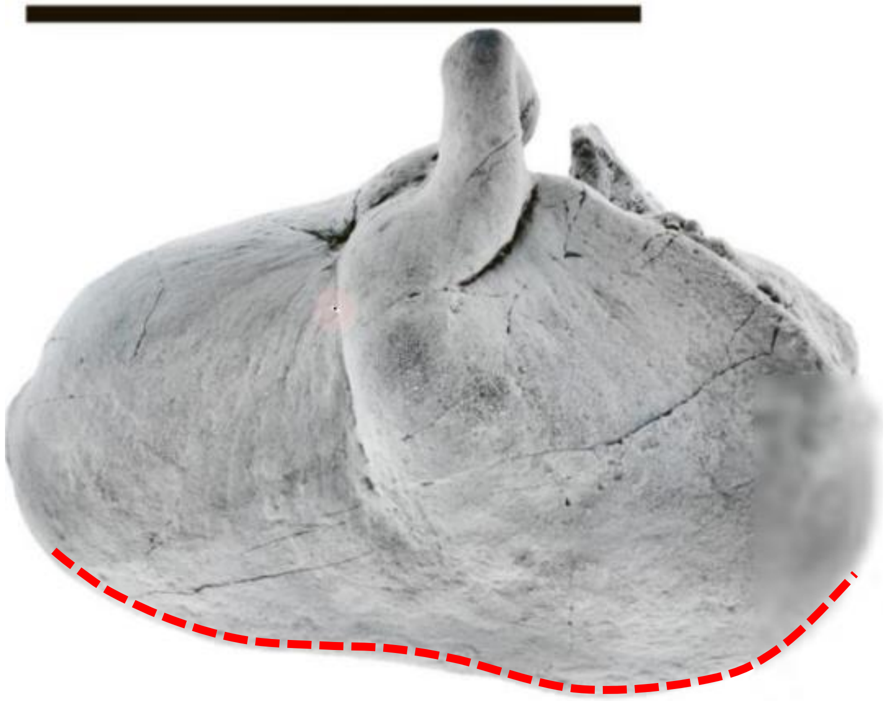

Horopeta umarere

Copyright holder: Cheng-Hsiu Tsai/ Otago University Geology Museum, Dunedin, New Zealand

(1)

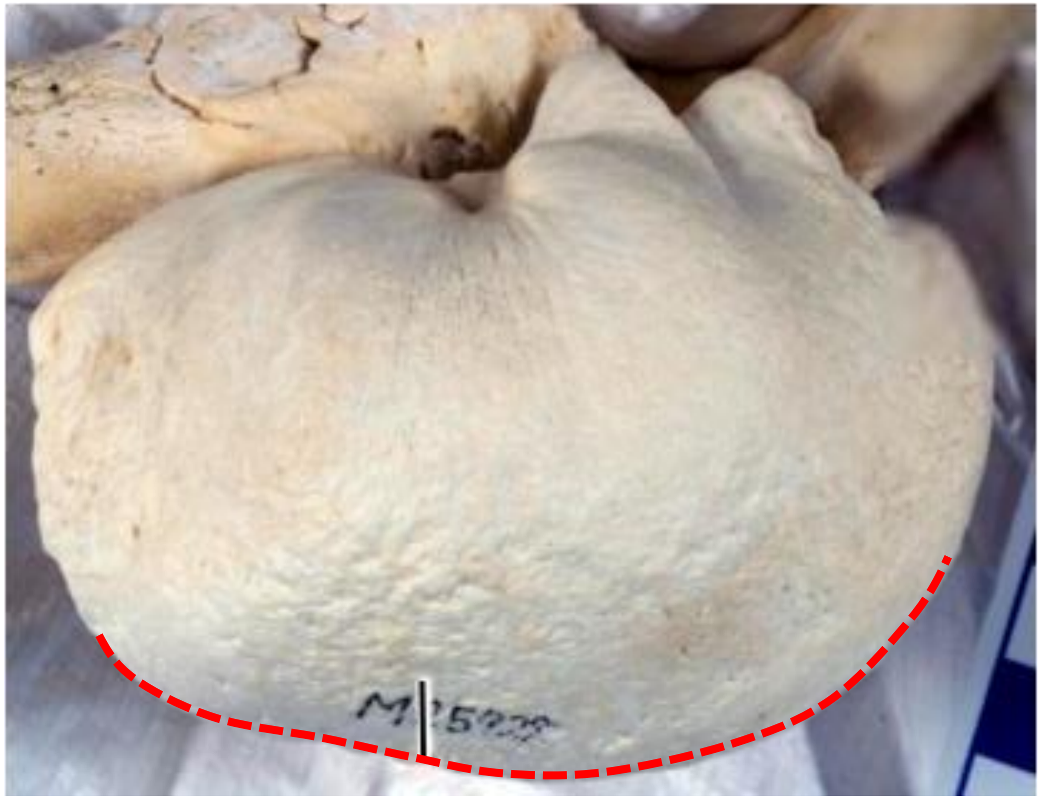

Balaenoptera acutorostrata

Copyright holder: Felix G. Marx, National Museum of Nature and Science, Tokyo, Japan

[198] 'Position of involucral ridge relative to medial margin of bulla'

(0)'coincident with medial margin'

(1)'retracted laterally'

(0)

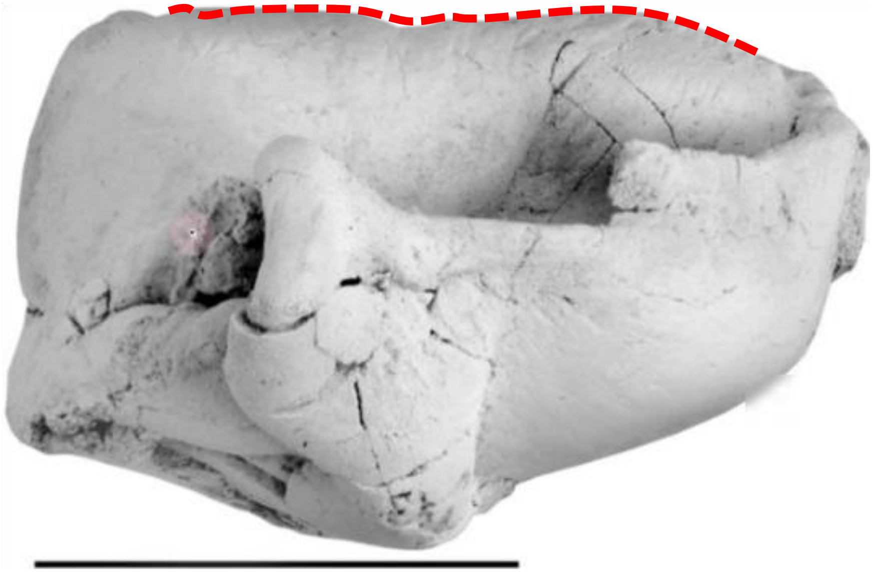

Whakakai waipata

Copyright holder: Cheng-Hsiu Tsai/University of Otago Geology Museum, Dunedin, New Zealand

(1)

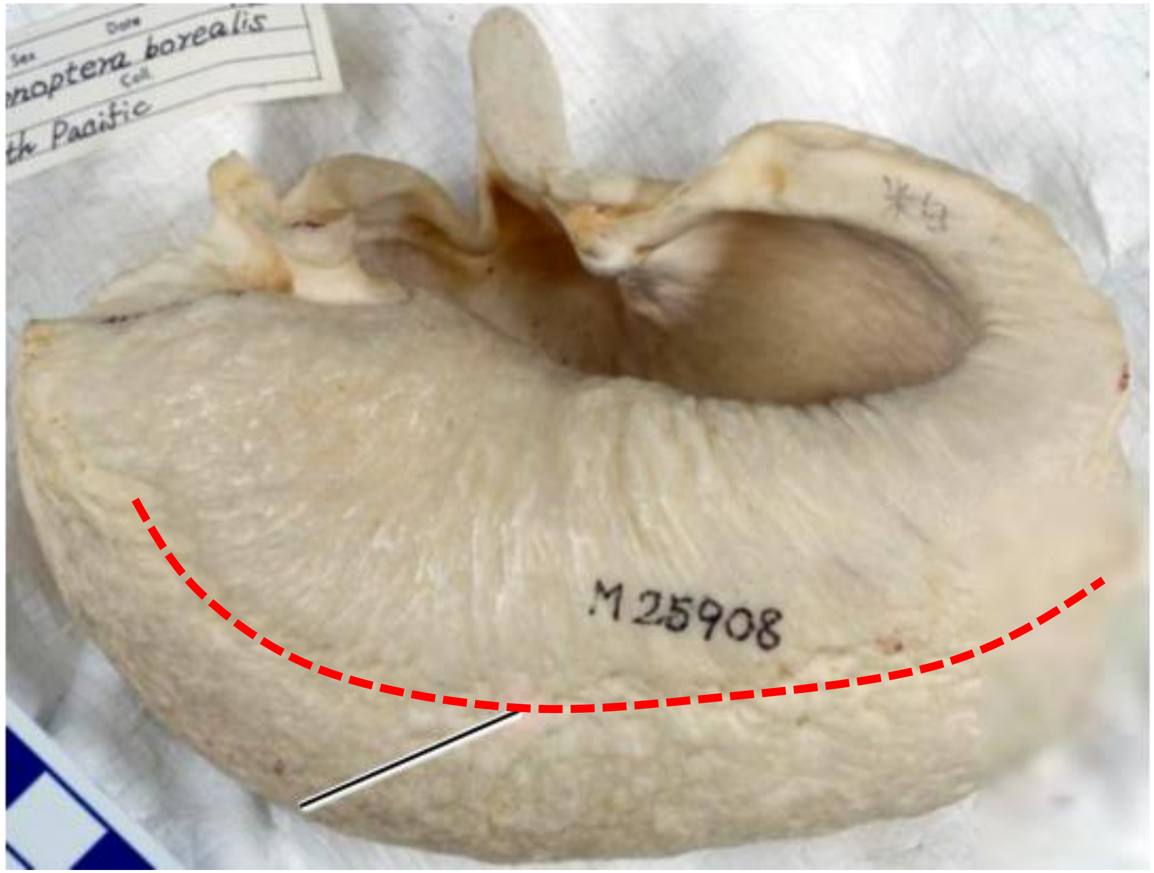

Balaenoptera borealis

Copyright holder: Felix G. Marx/ National Museum of Nature and Science, Tokyo, Japan

[199] 'Position of involucral ridge relative to posterior margin of bulla'

(0)'coincident with posterior margin'

(1)'retracted anteriorly'

(0)

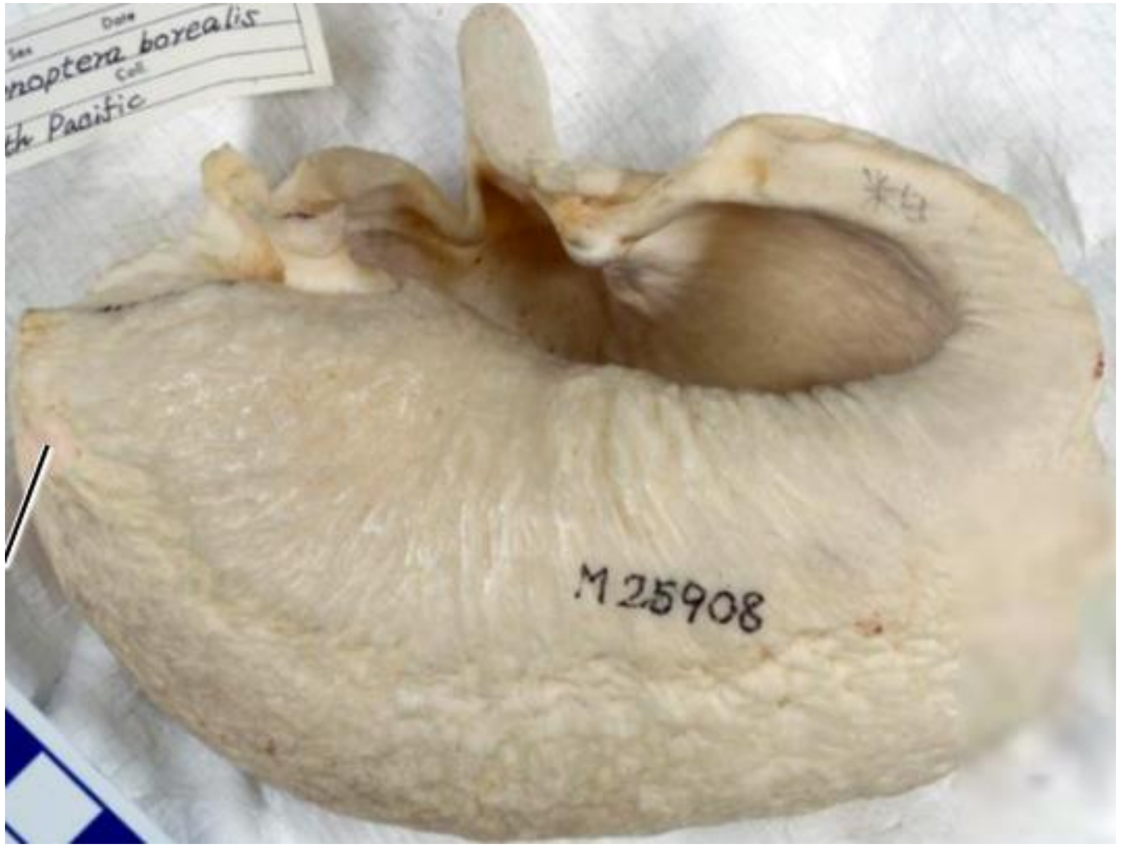

Balaenoptera borealis

Copyright holder: Felix G. Marx/ National Museum of Nature and Science, Tokyo, Japan

(1)

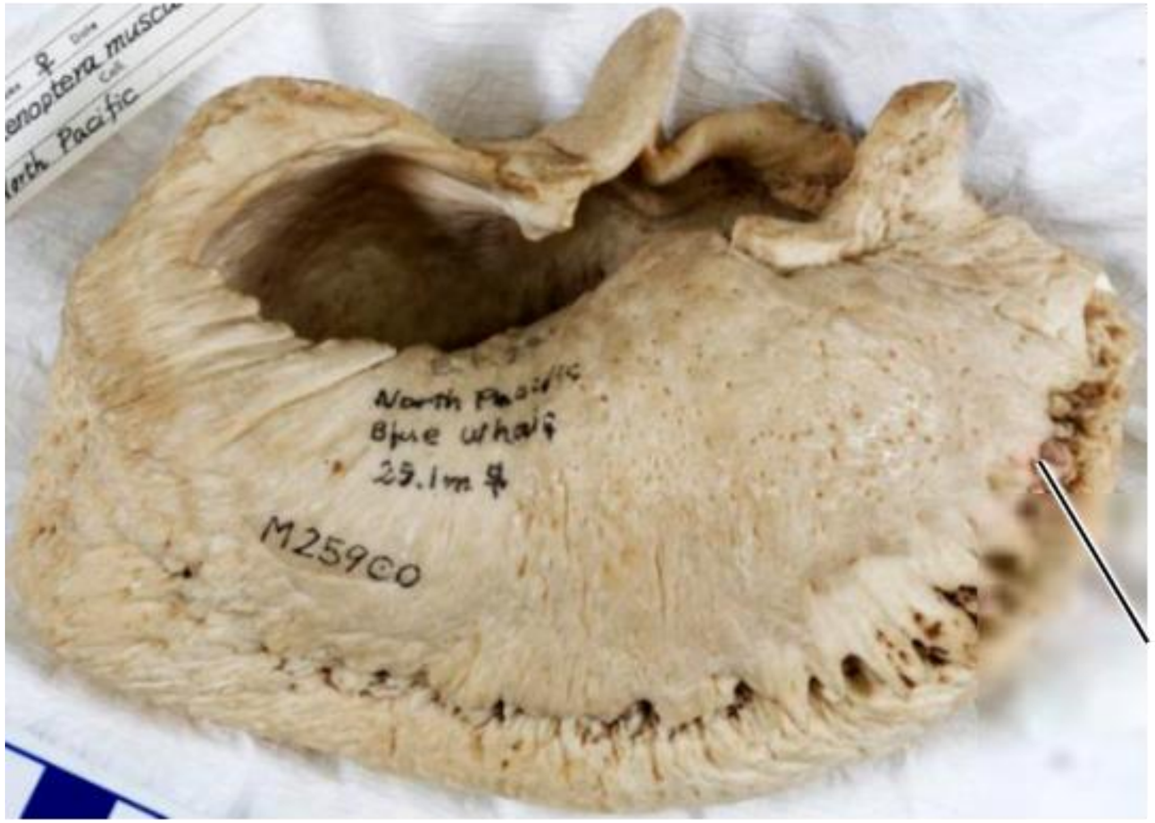

Balaenoptera musculus

Copyright holder: Felix G. Marx/ National Museum of Nature and Science, Tokyo, Japan

[200] 'Sigmoid process deflected laterally in anterior or posterior view'

(0)'absent'

(1)'present'

(2)'as state 1, but with the sigmoid process being nearly horizontal'

(0)

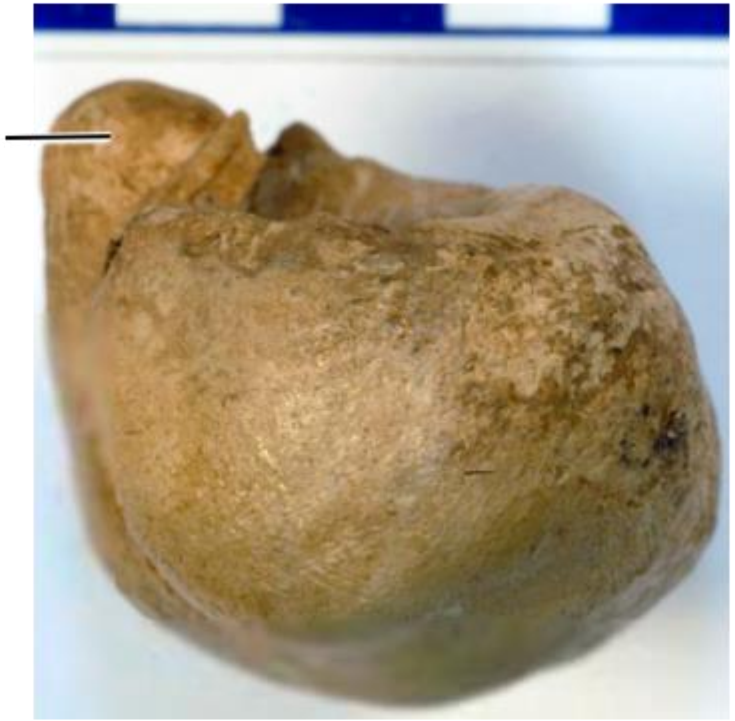

Parietobalaena palmeri
(mirrored)

Copyright holder: Felix G. Marx/ United States National Museum of Natural History, Washington DC, USA

(1)

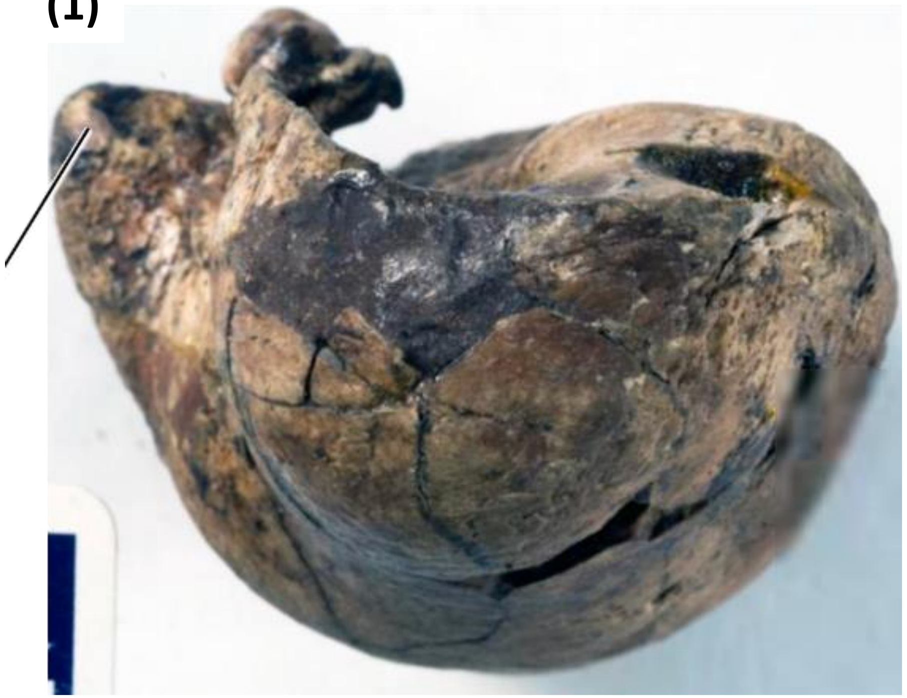

Thinocetus arthritus

Copyright holder: Felix G. Marx/ United States National Museum of Natural History, Washington DC, USA

(2)

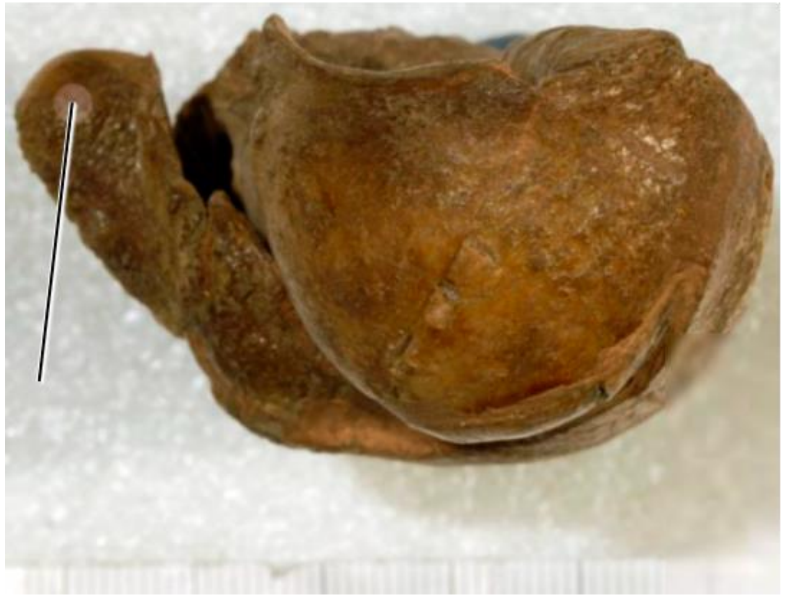

Pelocetus calvertensis

Copyright holder: Felix G. Marx/ United States National Museum of Natural History, Washington DC, USA

[201] 'Dorsomedial corner of sigmoid process in anterior view'

(0)'separated from anterior process of the malleus’

(1)'confluent with anterior process of the malleus'

(0)

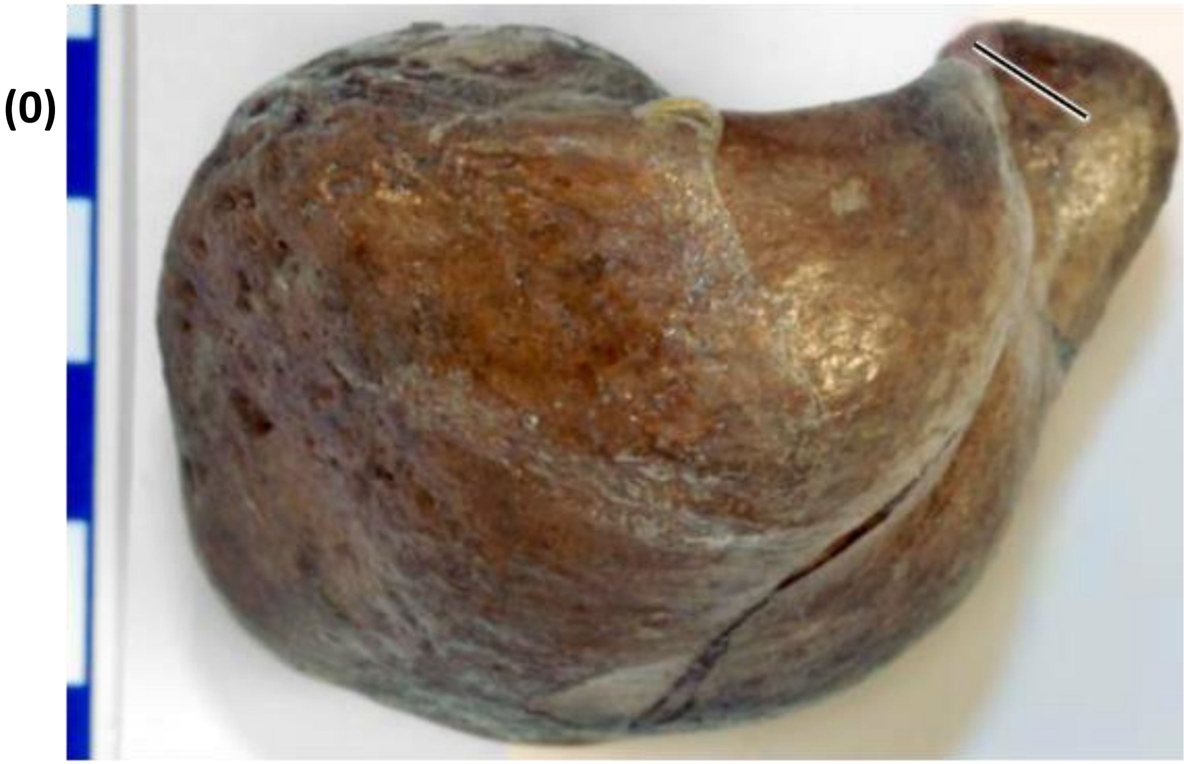

Diorocetus hiatus

Copyright holder: Felix G. Marx/ United States National Museum of Natural History, Washington DC, USA

(1)

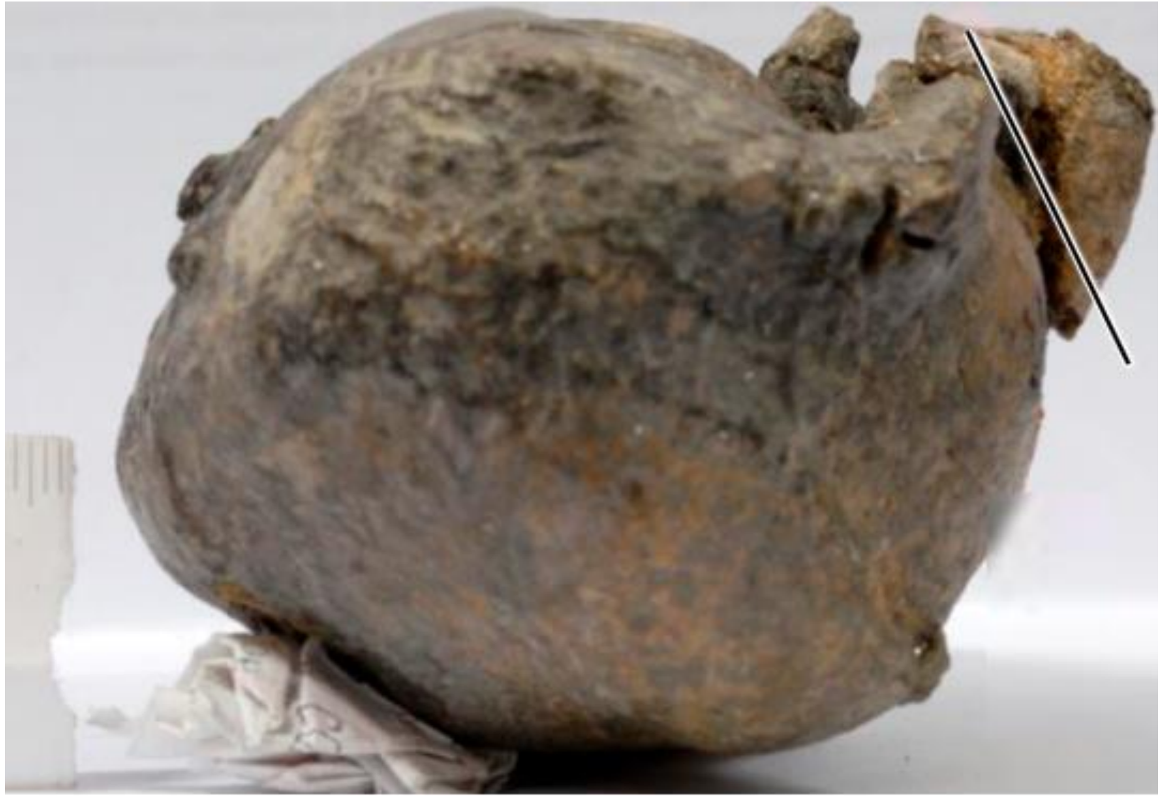

Plesiobalaenoptera quarantellii

Copyright holder: Felix G. Marx/ Museo Paleontologico di Salsomaggiore Terme/ Soprintendenza Per I Beni Archeologici Dell™Emilia Romagna, Italy

[202] 'Ventral margin of sigmoid process in lateral view'

(0)'present’

(1)'absent, with the lateral margin of the sigmoid process turning smoothly into a sulcus on the lateral side of the bulla'

(0)

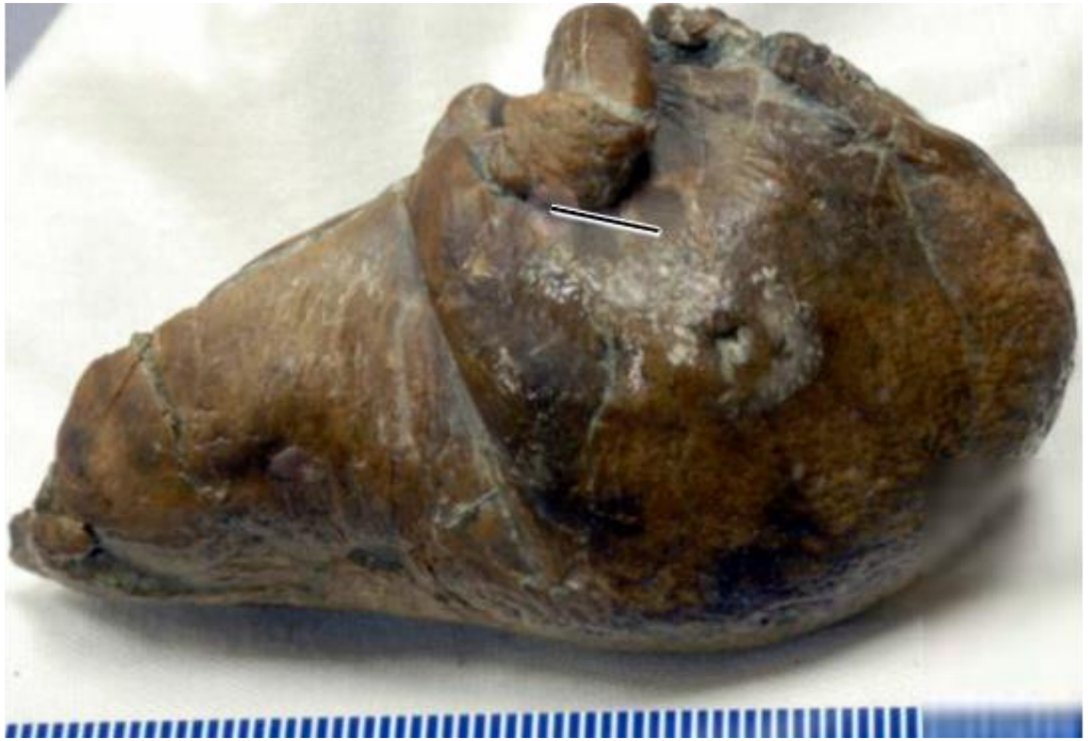

Aetiocetus weltoni

Copyright holder: Felix G. Marx/ University of California Museum of Paleontology, Berkeley, USA

(1)

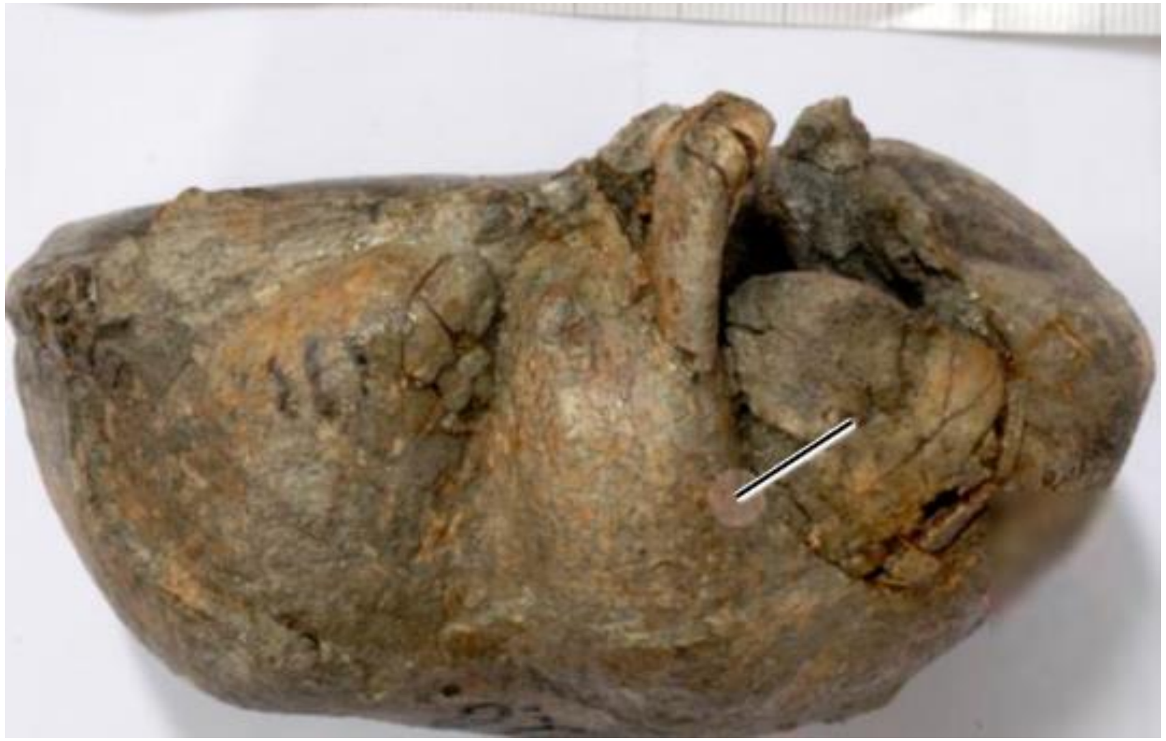

Plesiobalaenoptera quarantellii

Copyright holder: Felix G. Marx/ Museo Paleontologico di Salsomaggiore Terme/ Soprintendenza Per I Beni Archeologici Dell™Emilia Romagna, Italy

[203] 'Shape of conical process in lateral view'

(0) 'well developed and dorsally convex'

(1) 'reduced to a low ridge or absent'

(0)

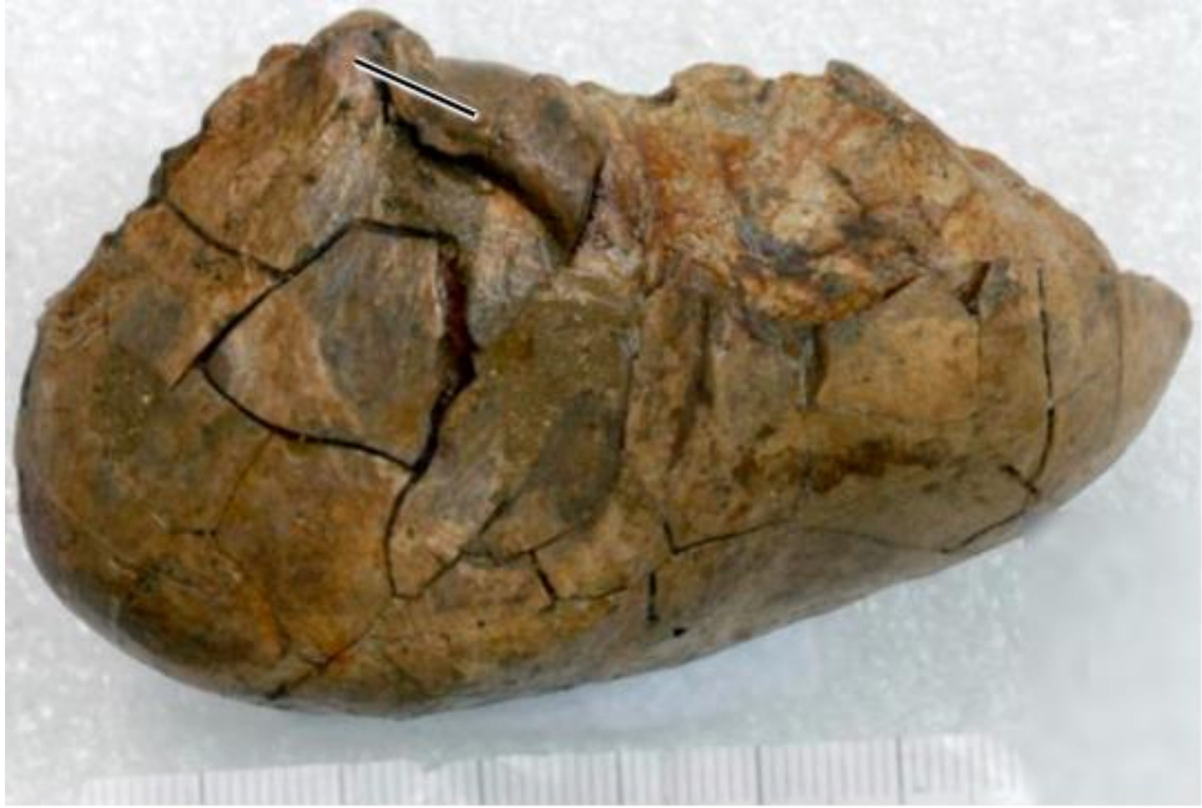

Zygorhiza kochii

Copyright holder: Felix G. Marx/ United States National Museum of Natural History, Washington DC, USA

(1)

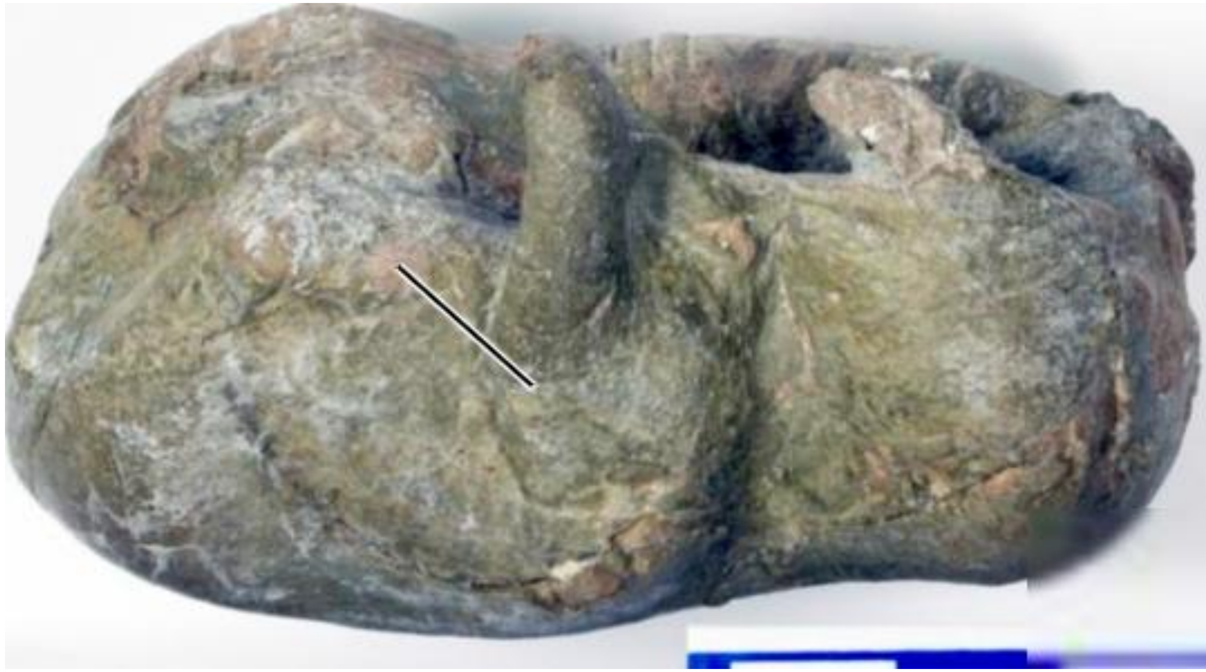

Diorocetus chichibuensis

Copyright holder: Felix G. Marx/ Saitama Museum of Natural History, Saitama, Japan

[204] 'Elliptical foramen'

(0)'present'

(1)'absent'

(0)

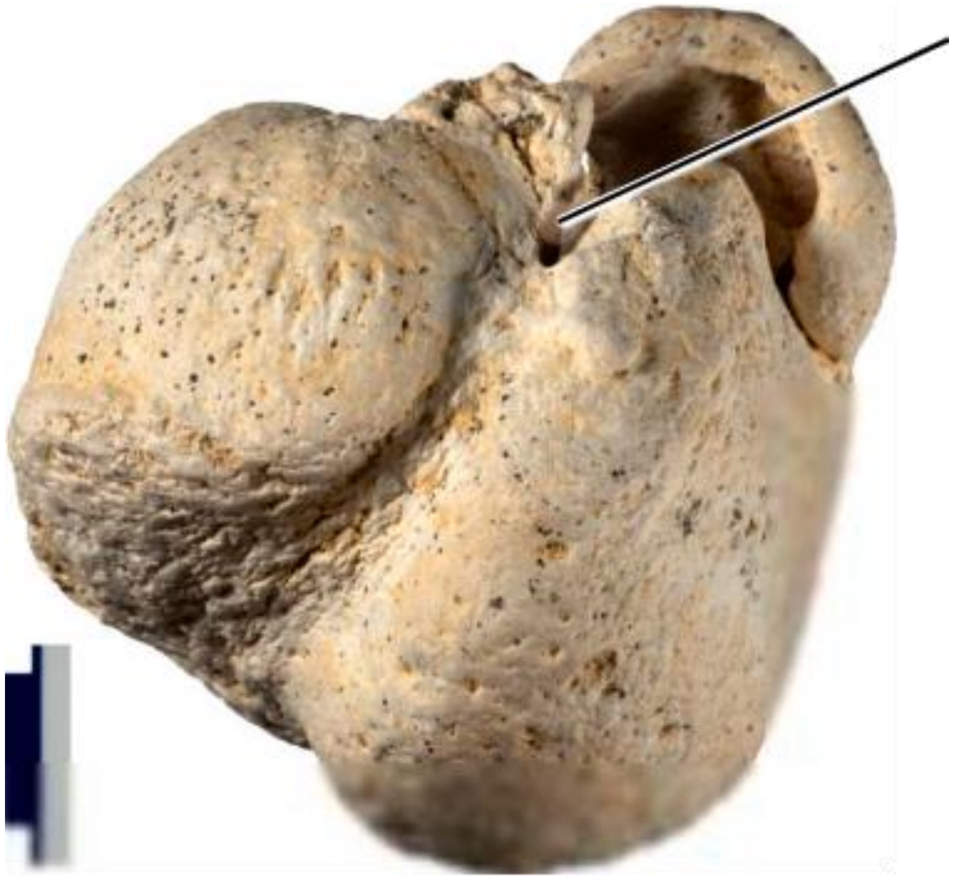

Tokarahia kauaeroa

Copyright holder: Felix G. Marx/ University of Otago Geology Museum, Dunedin, New Zealand

(1)

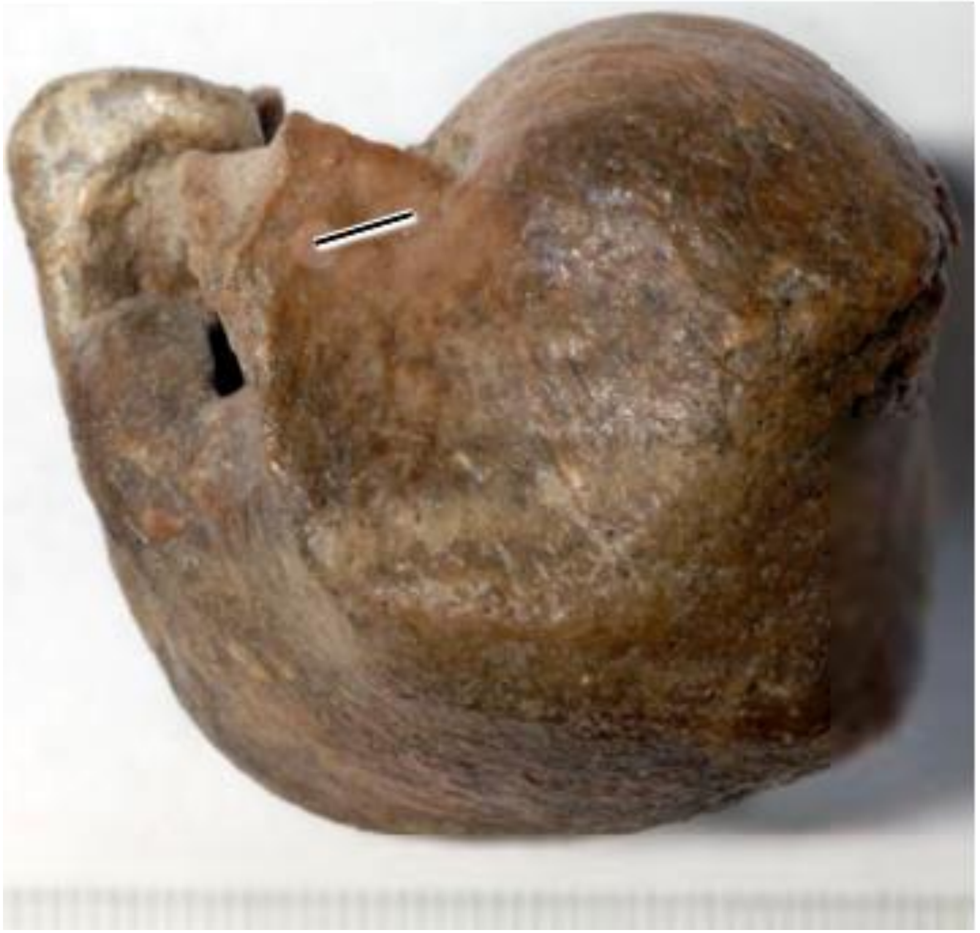

Piscobalaena nana

Copyright holder: Felix G. Marx/Museum National d'Histoire Naturelle, Paris, France

[205] 'Inner posterior prominence'

(0)'present as distinct lobe and transversely wider than its lateral counterpart'

(1)'present but subequal in width to outer posterior prominence or smaller'

(2)'absent or indistinct'

(0)

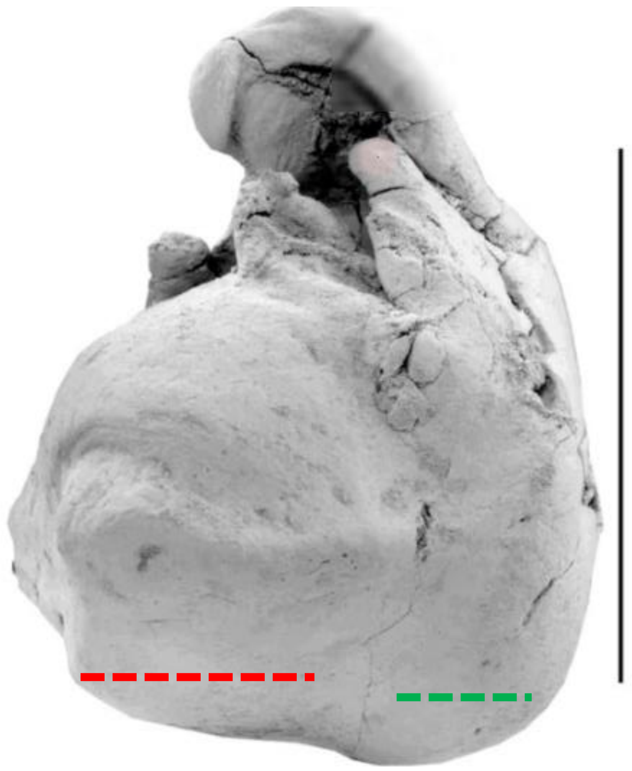

Whakakai waipata

Copyright holder: Cheng-Hsiu Tsai/University of Otago Geology Museum, Dunedin, New Zealand

(1)

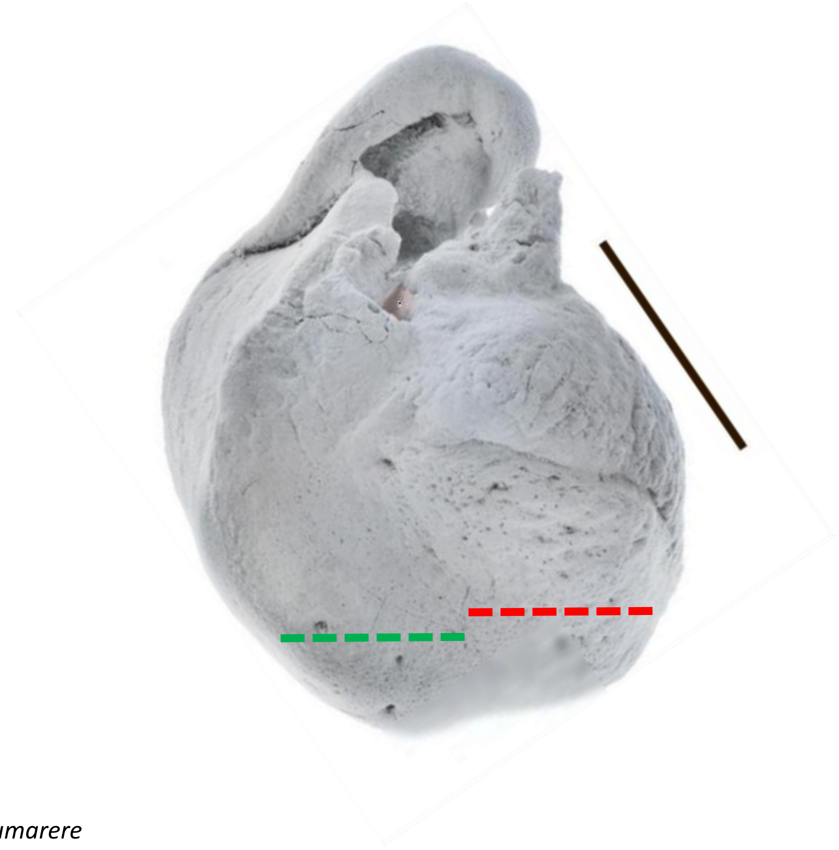

Horopeta umarere

Copyright holder: Cheng-Hsiu Tsai/ Otago University Geology Museum, Dunedin, New Zealand

[205] 'Inner posterior prominence'

(0)'present as distinct lobe and transversely wider than its lateral counterpart'

(1)'present but subequal in width to outer posterior prominence or smaller'

(2)'absent or indistinct'

(2)

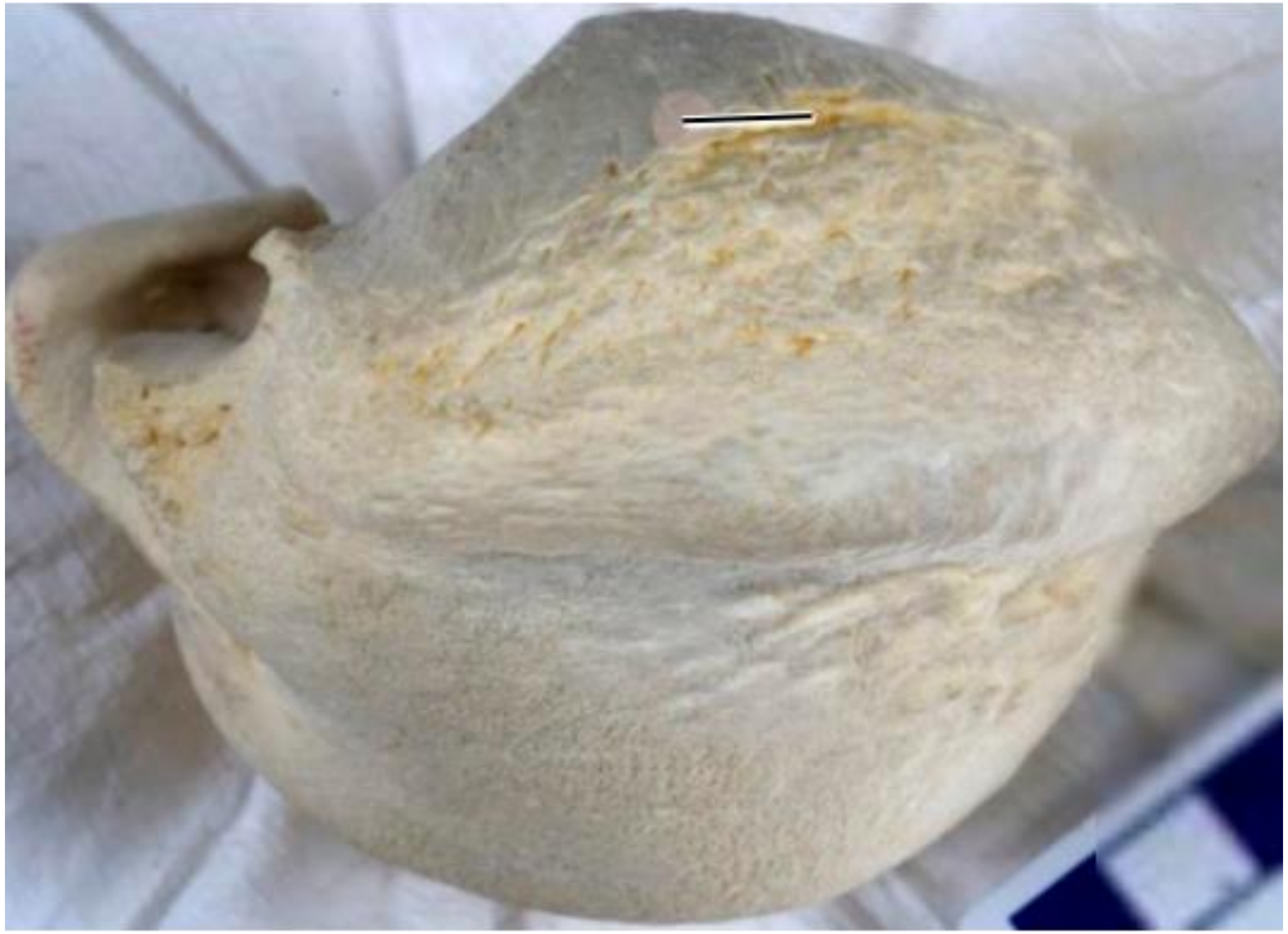

Balaenoptera physalus

Copyright holder: Felix G. Marx/ National Museum of Nature and Science, Tokyo, Japan

[206] 'Interprominential ridge'

(0) 'present'

(1) 'absent'

(0)

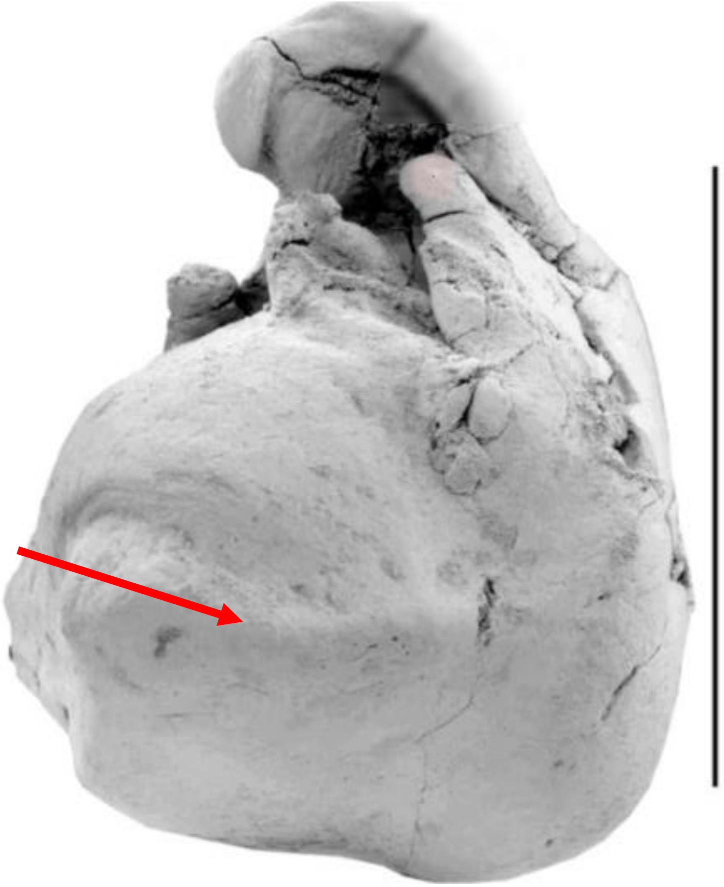

Whakakai waipata

Copyright holder: Cheng-Hsiu Tsai/University of Otago Geology Museum, Dunedin, New Zealand

(1)

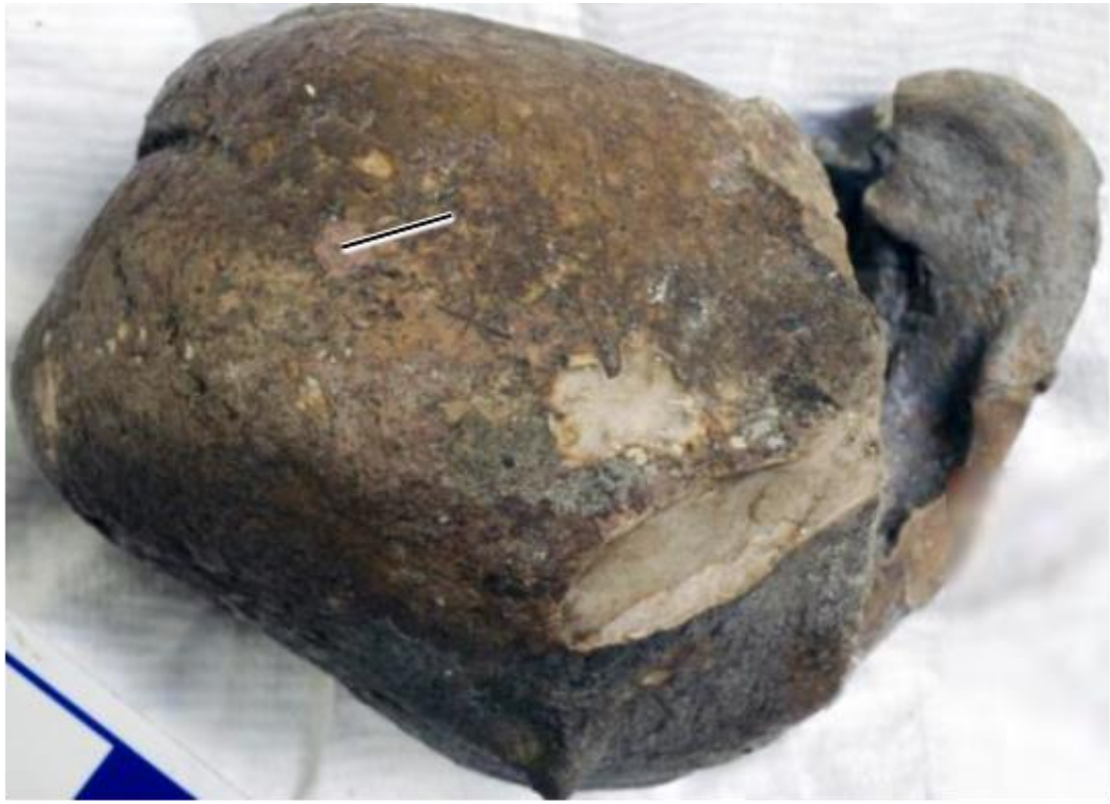

Balaenula sp.

Copyright holder: Felix G. Marx/ Sapporo Museum Activity Centre, Sapporo, Japan

[207] 'Transverse creases on dorsal surface of involucrum'

(0)'poorly developed or absent'

(1)'well defined and deep'

(0)

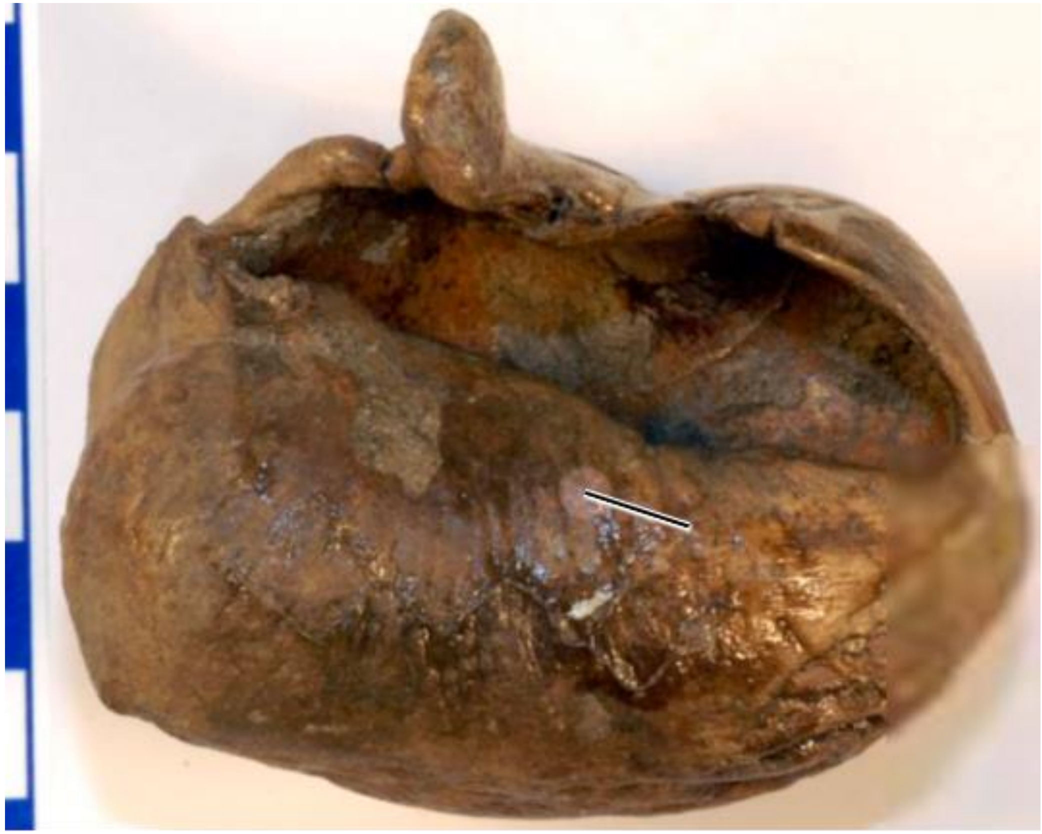

Diorocetus hiatus

Copyright holder: Felix G. Marx/ United States National Museum of Natural History, Washington DC, USA

(1)

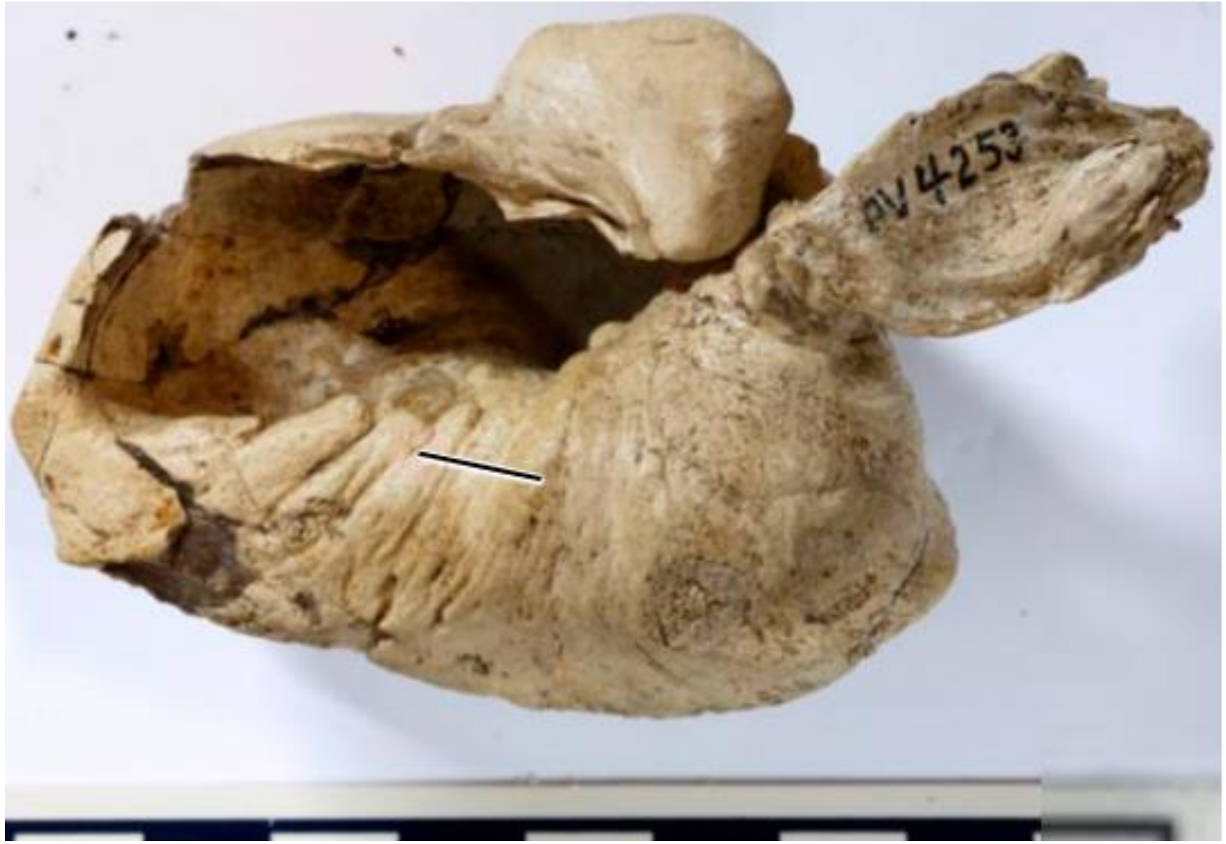

Eomysticetus whitmorei

Copyright holder: Felix G. Marx/ The Charleston Museum, Charleston, South Carolina, USA

[208] 'Ridge on inside of bulla'

(0)'extends laterally from involucrum across the floor of the tympanic cavity'

(1)'absent'

(0)

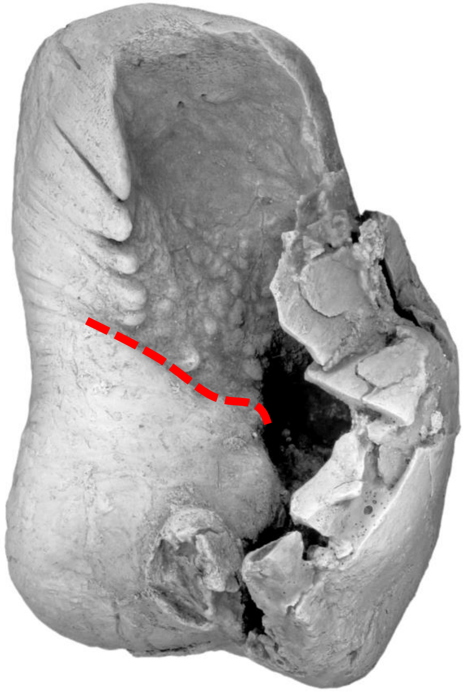

Mammalodon colliveri

Copyright holder: Erich M. G. Fitzgerald/ Museums Victoria, Melbourne, Australia

(1)

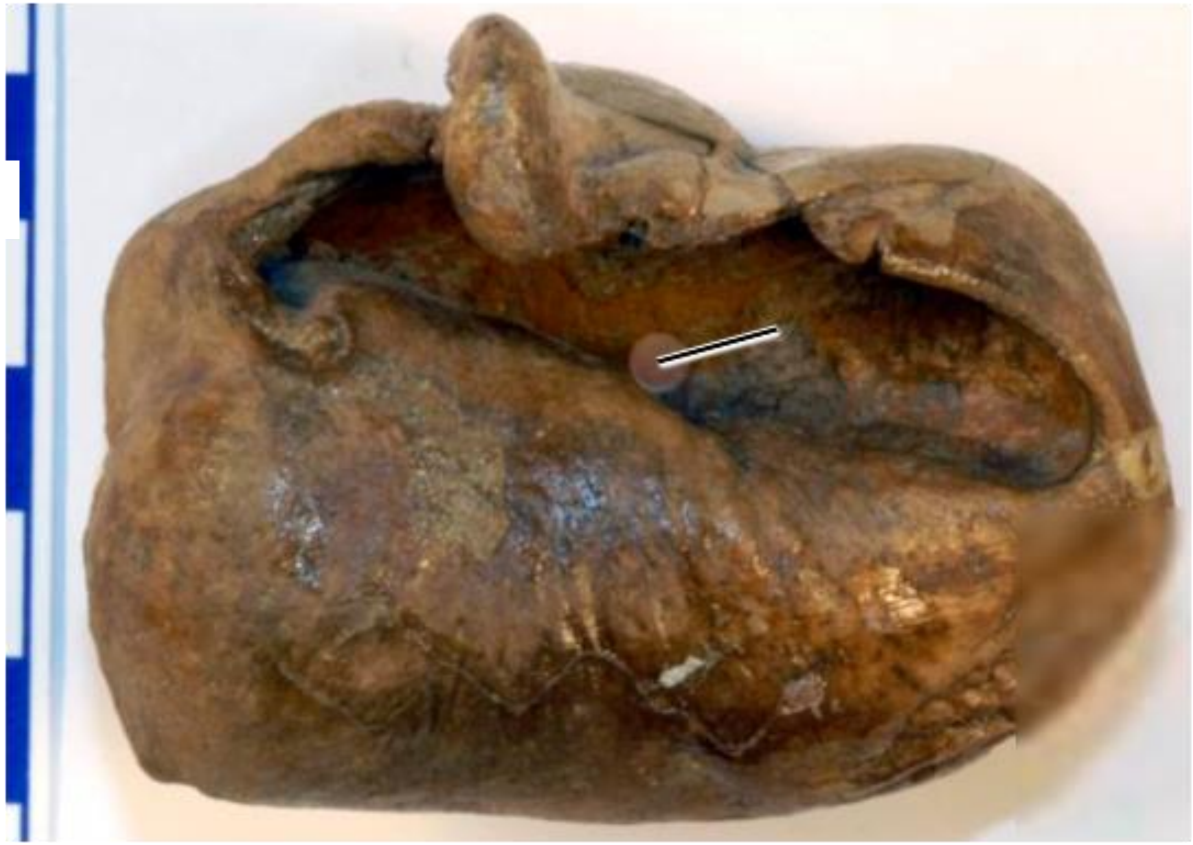

Diorocetus hiatus

Copyright holder: Felix G. Marx/ United States National Museum of Natural History, Washington DC, USA

[209] 'Development of tympanic sulcus'

(0)'faint line or low ridge'

(1)'distinct crest or sulcus'

(0)

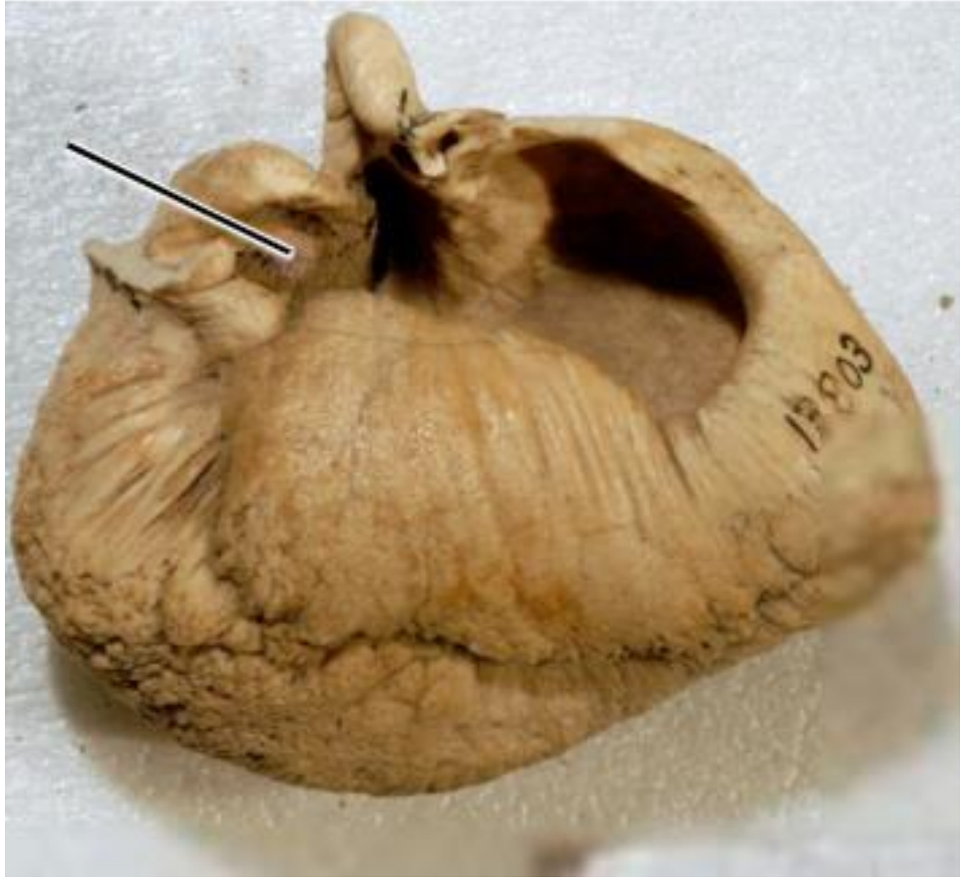

Eschrichtius robustus

Copyright holder: Felix G. Marx/ United States National Museum of Natural History, Washington DC, USA

(1)

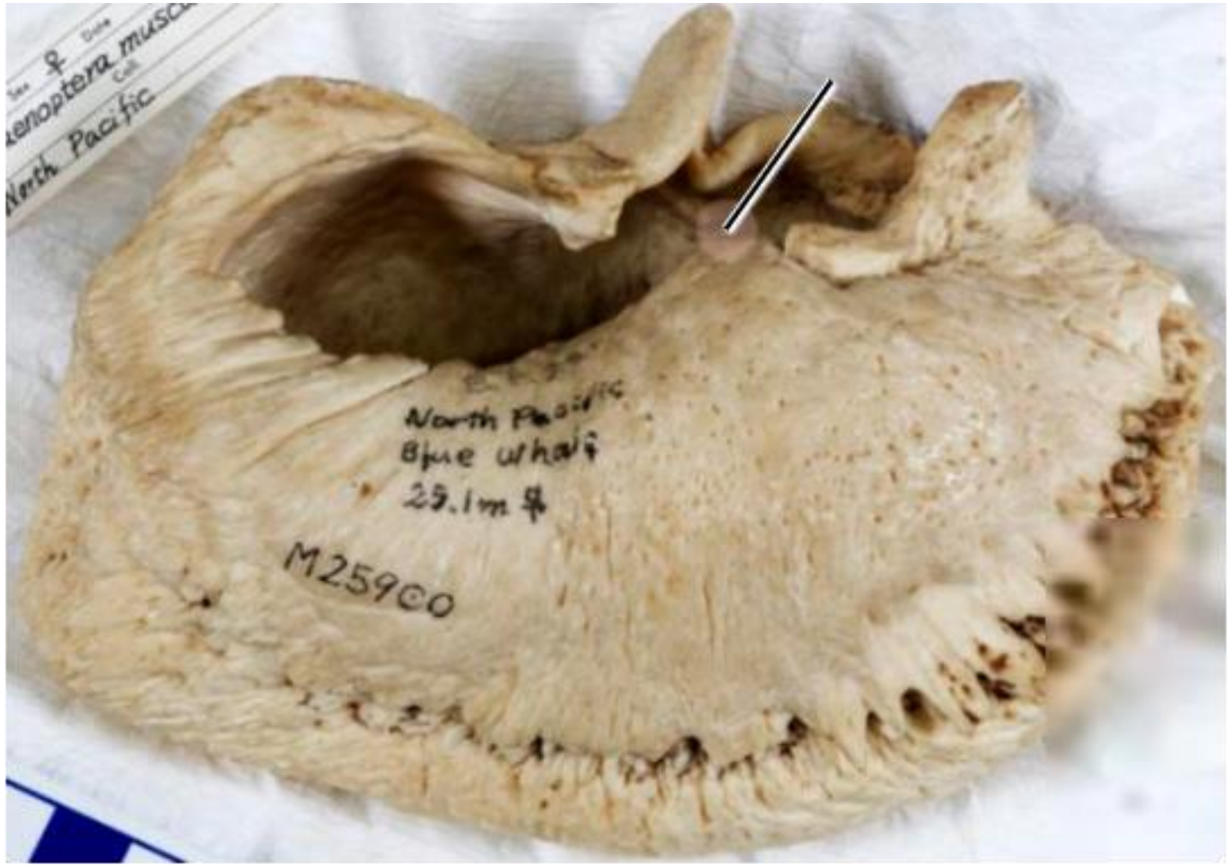

Balaenoptera musculus

Copyright holder: Felix G. Marx/ National Museum of Nature and Science, Tokyo, Japan

[210] 'Outline and position of tympanic sulcus'

(0)'forms a semicircular and ventrally curved line well separated from the intersection of the conical and sigmoid processes'

(1)'forms a roughly horizontal line at or close to the level of the intersection of the conical and sigmoid processes'

(0)

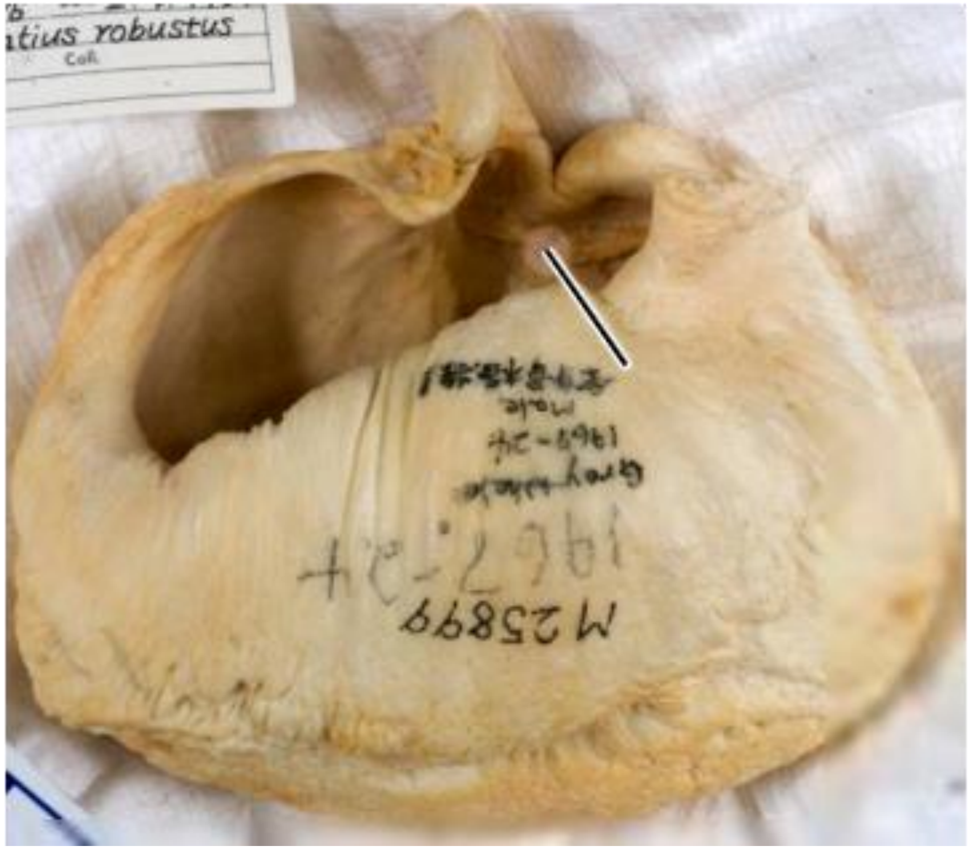

Eschrichtius robustus

Copyright holder: Felix G. Marx/ National Museum of Nature and Science, Tokyo, Japan

(1)

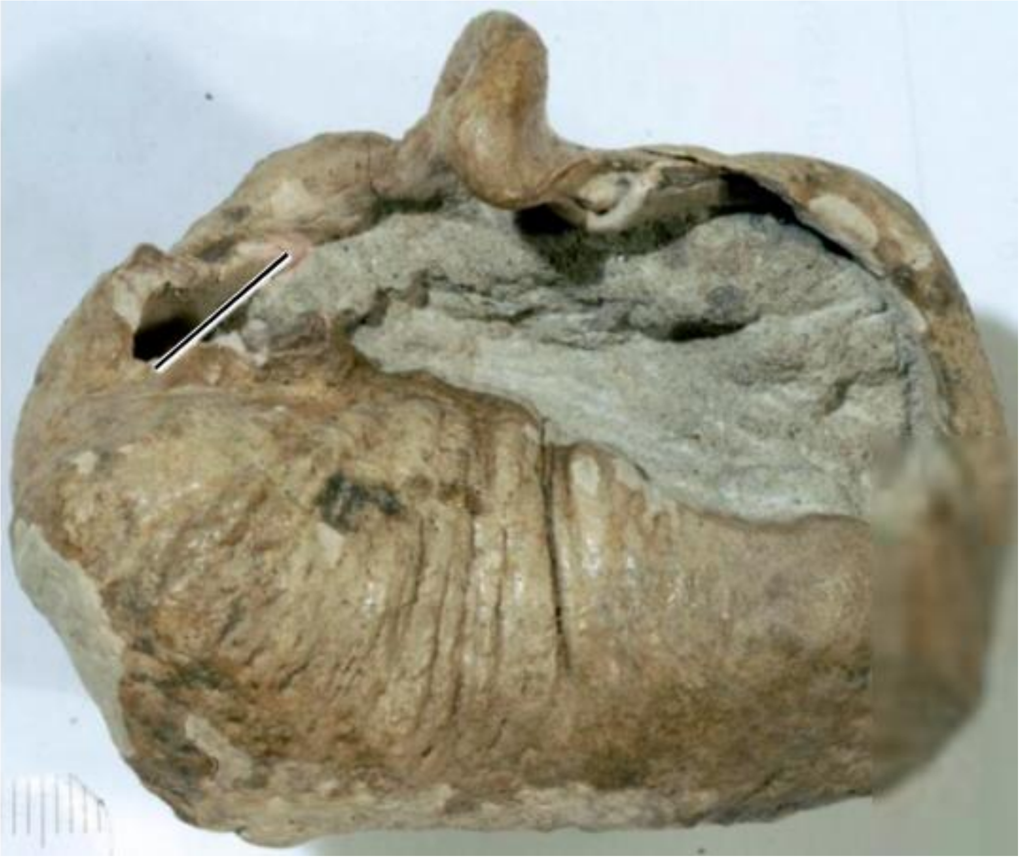

Herpetocetus transatlanticus

Copyright holder: Felix G. Marx/ United States National Museum of Natural History, Washington DC, USA

[211] 'Anteromedial portion of ventral surface of tympanic bulla'

(0)'transversely convex'

(1)'distinctly flattened or slightly concave'

(0)

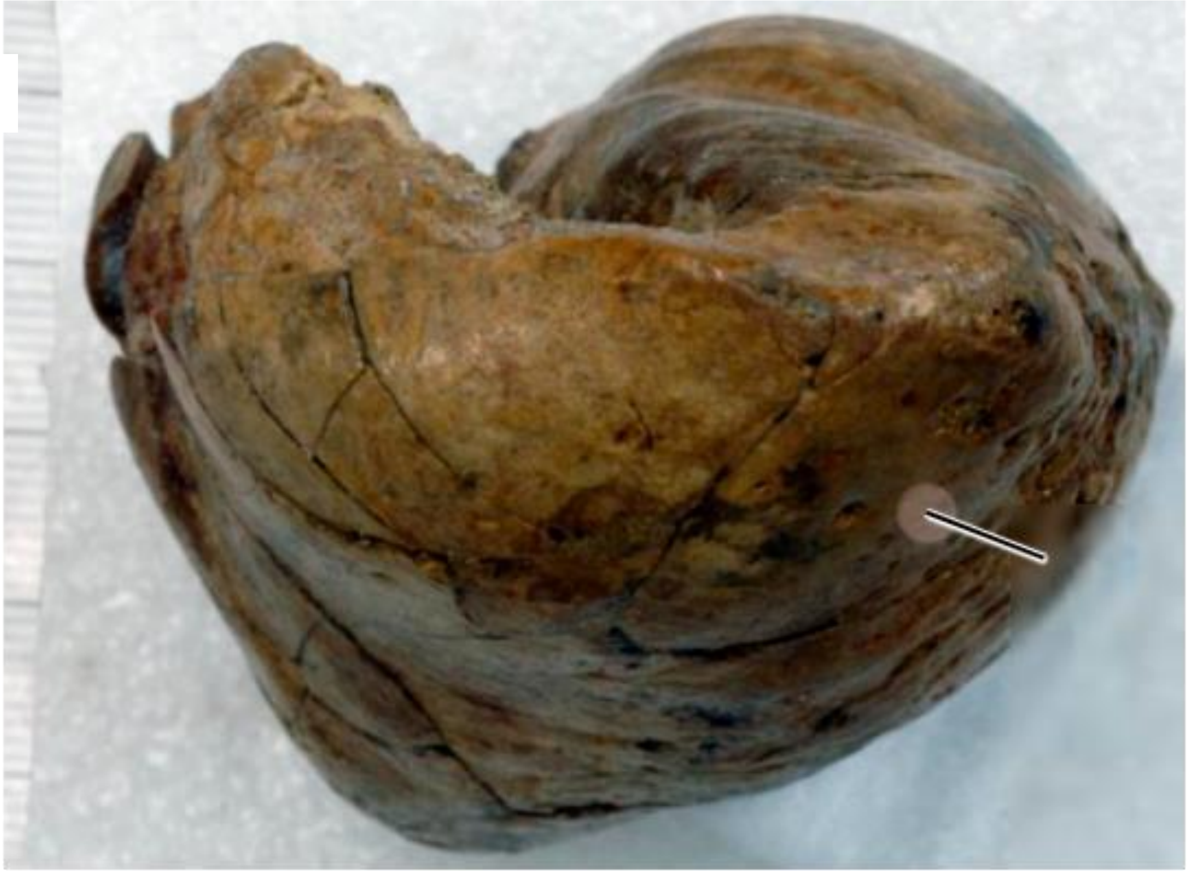

Zygorhiza kochii

Copyright holder: Felix G. Marx/ United States National Museum of Natural History, Washington DC, USA

(1)

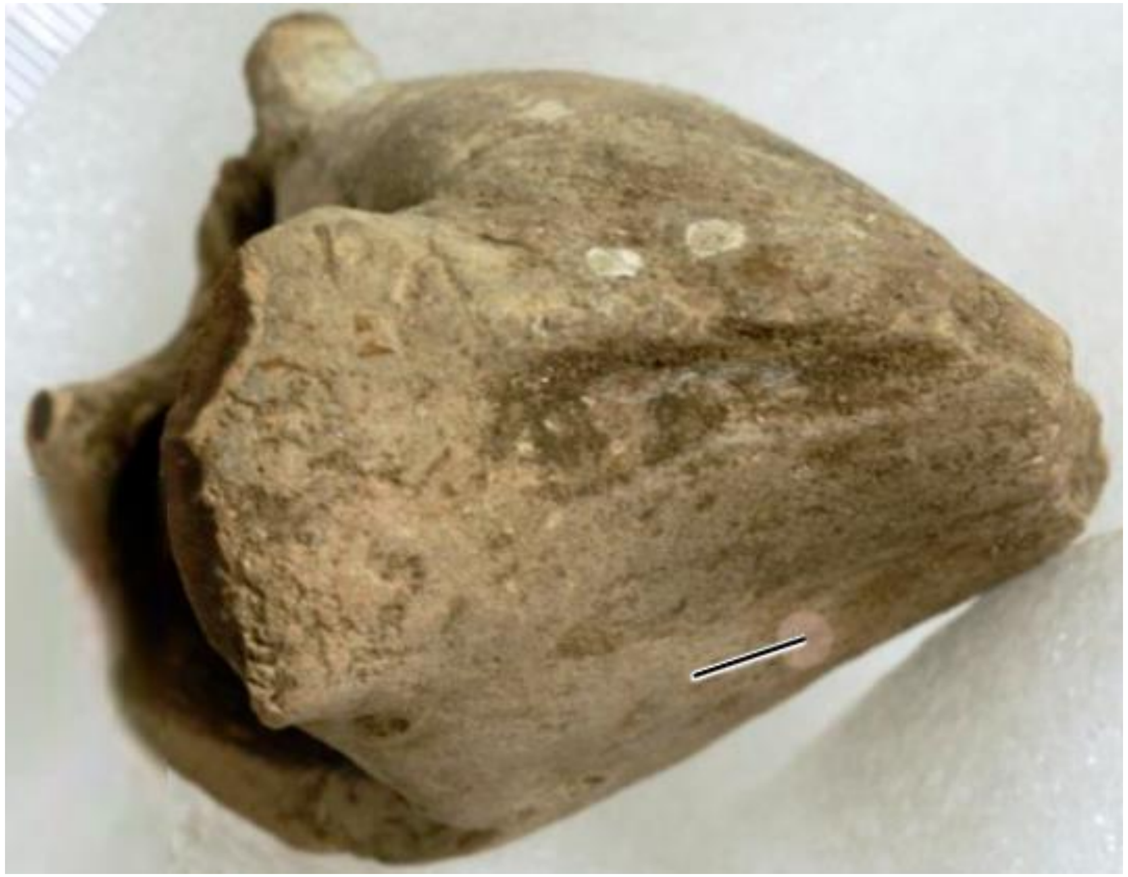

Balaenella brachyrhynus (mirrored)

Copyright holder: Felix G. Marx/Natuurmuseum Brabant, Tilburg, the Netherlands

[212] 'Anterolateral corner of bulla'

(0)'rounded or flattened'

(1)'inflated and forming a distinct lobe'

(0)

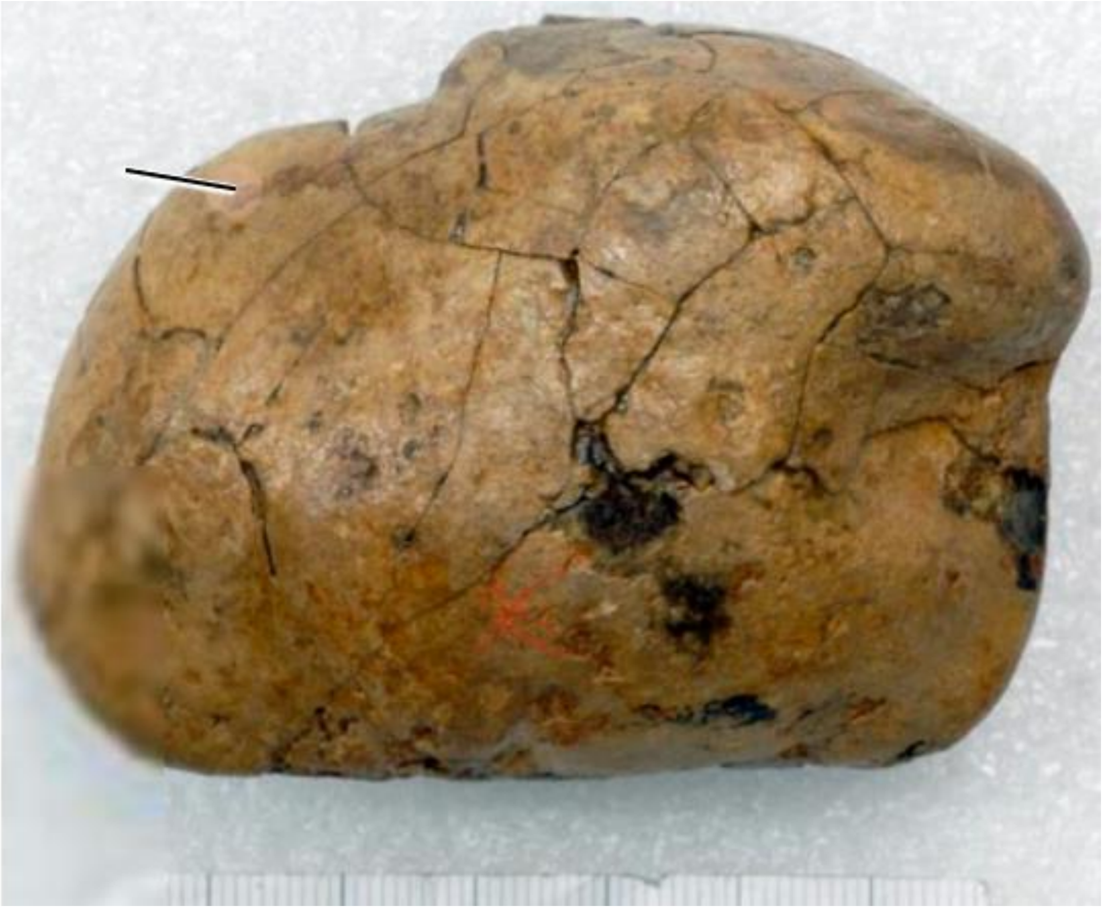

Zygorhiza kochii (mirrored)

Copyright holder: Felix G. Marx/ United States National Museum of Natural History, Washington DC, USA

(1)

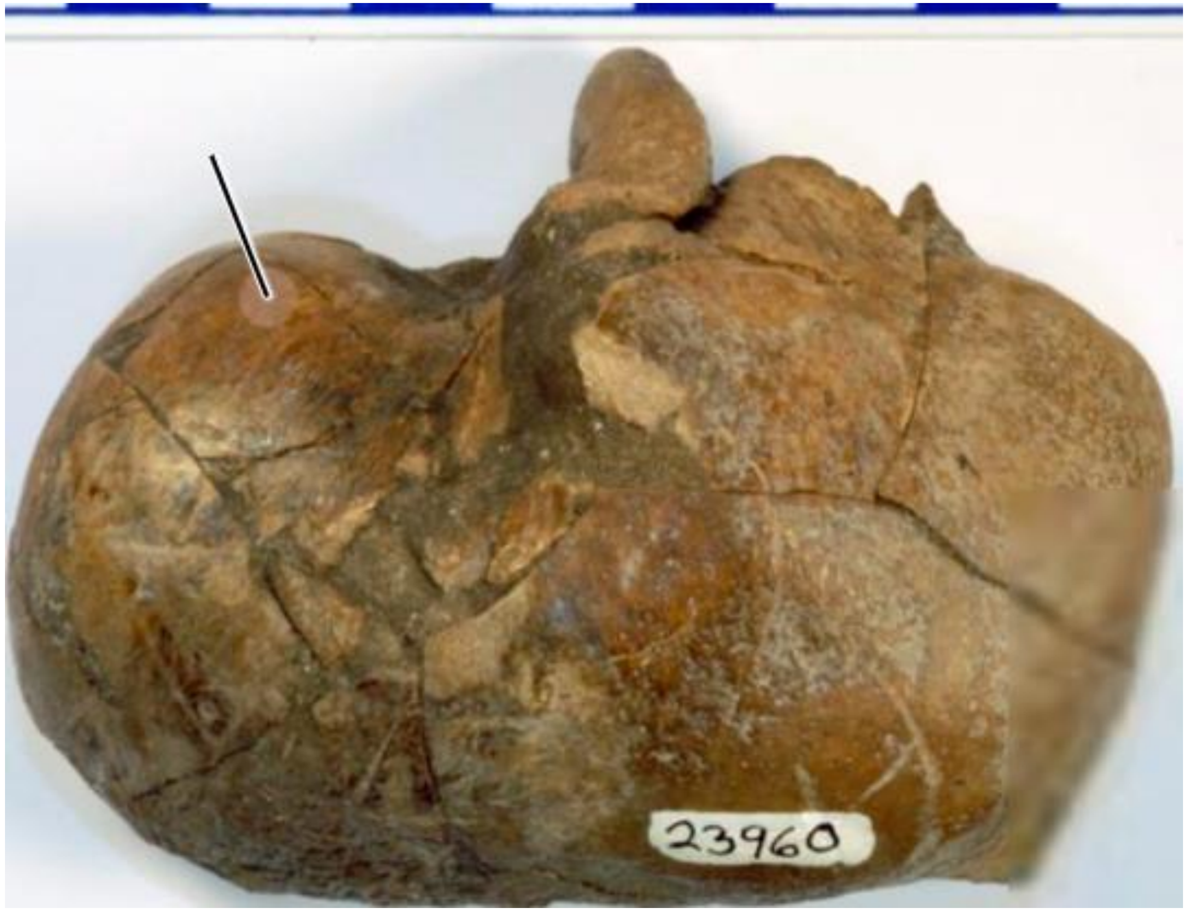

Aglaocetus patulus

Copyright holder: Felix G. Marx/ United States National Museum of Natural History, Washington DC, USA

[213] 'Anterolateral ridge or shelf'

(0)'absent'

(1)'present'

(0)

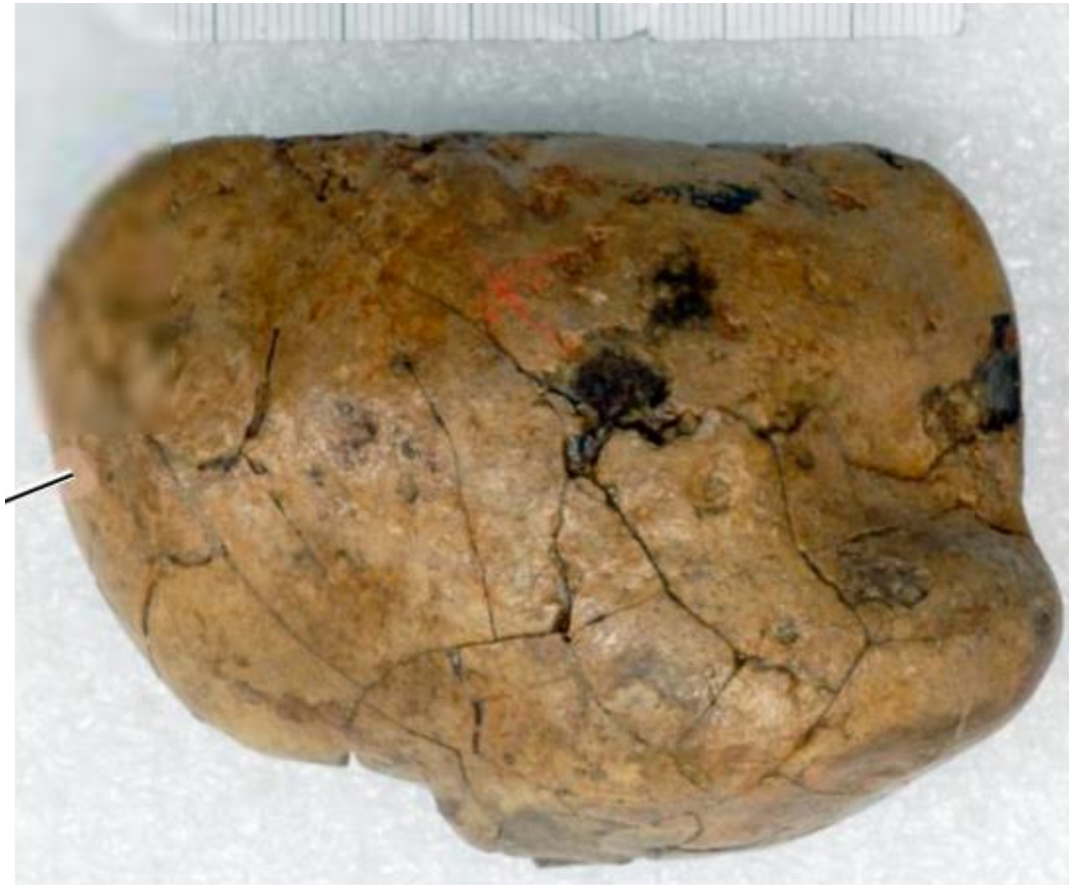

Zygorhiza kochii

Copyright holder: Felix G. Marx/ United States National Museum of Natural History, Washington DC, USA

(1)

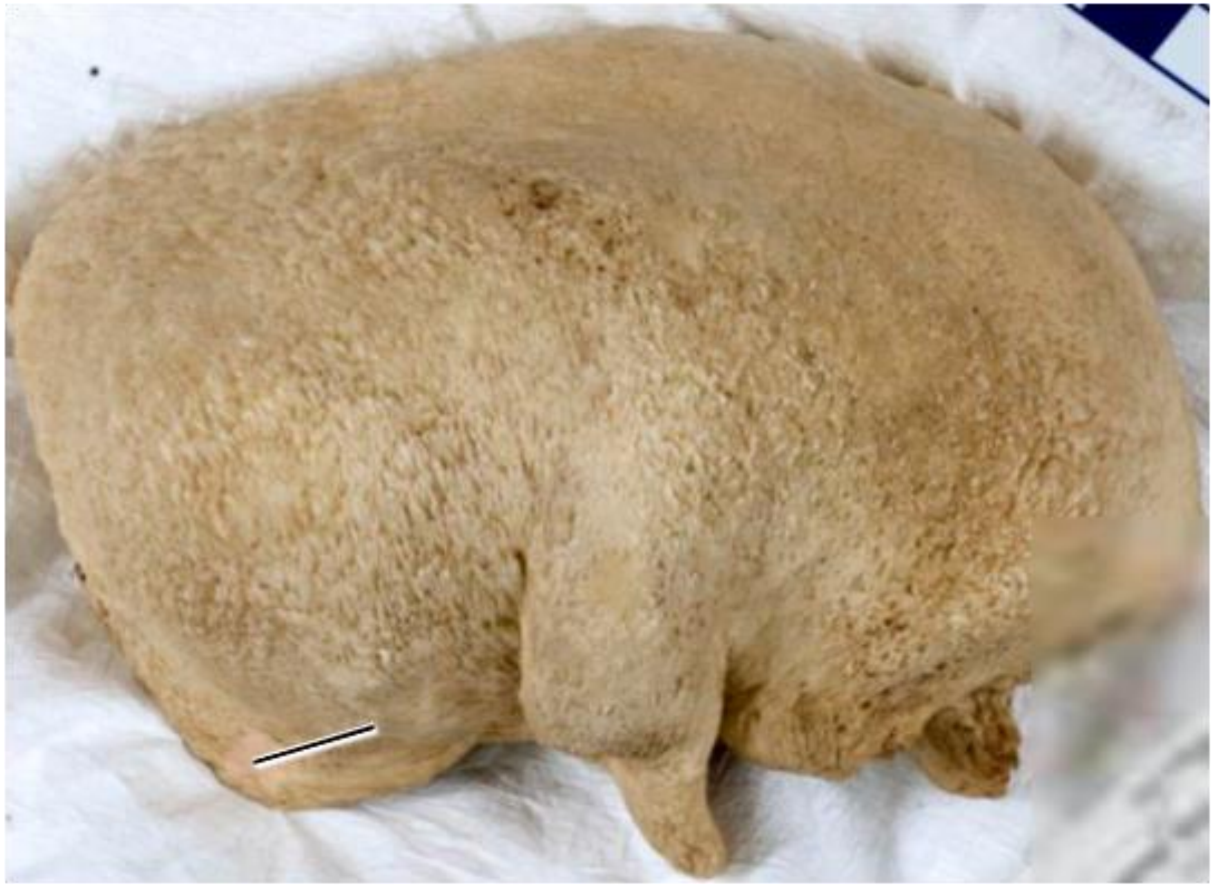

Balaenoptera musculus

Copyright holder: Felix G. Marx/ National Museum of Nature and Science, Tokyo, Japan

[214] 'Position of posterior pedicle in dorsal view'

(0)'situated at or near the posterior border of the bulla’

(1)'located far anterior to the posterior end of the bulla'

(0)

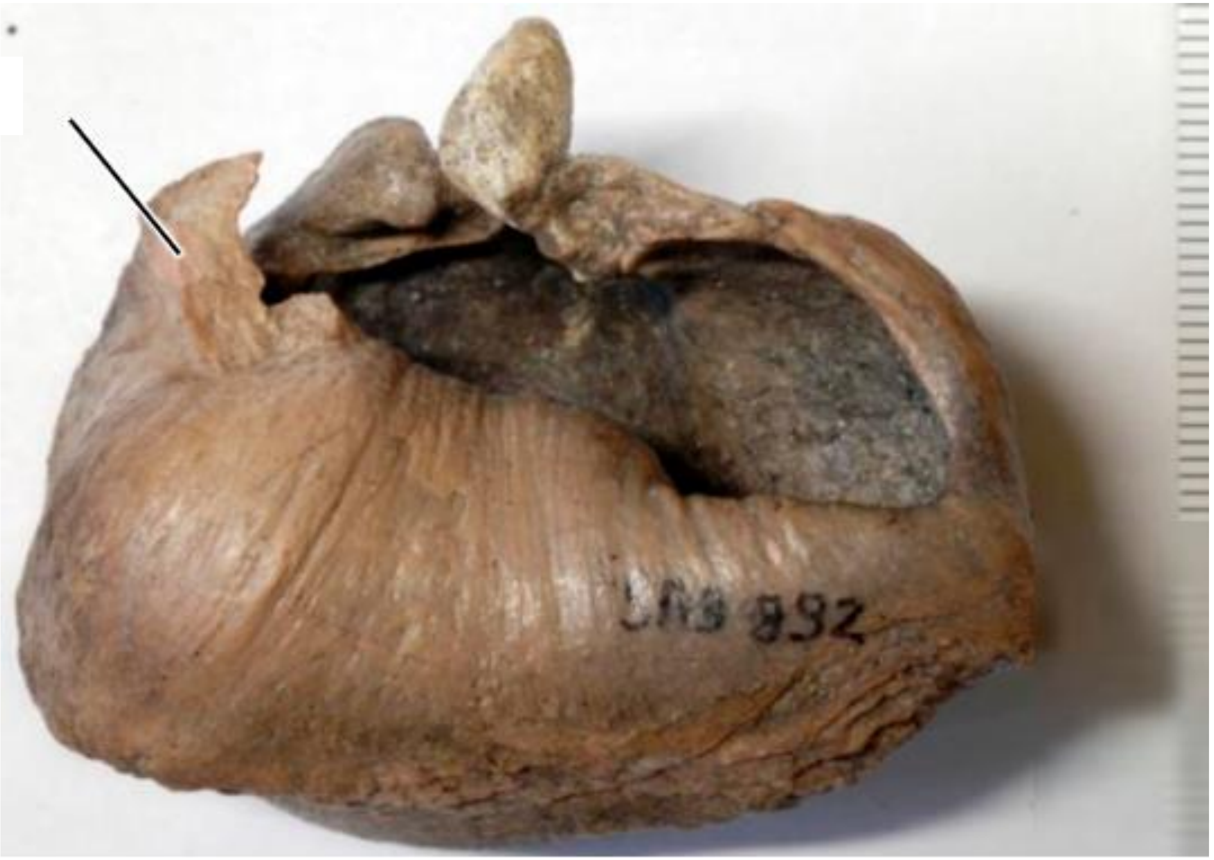

Piscobalaena nana

Copyright holder: Felix G. Marx/Museum National d'Histoire Naturelle, Paris, France

(1)

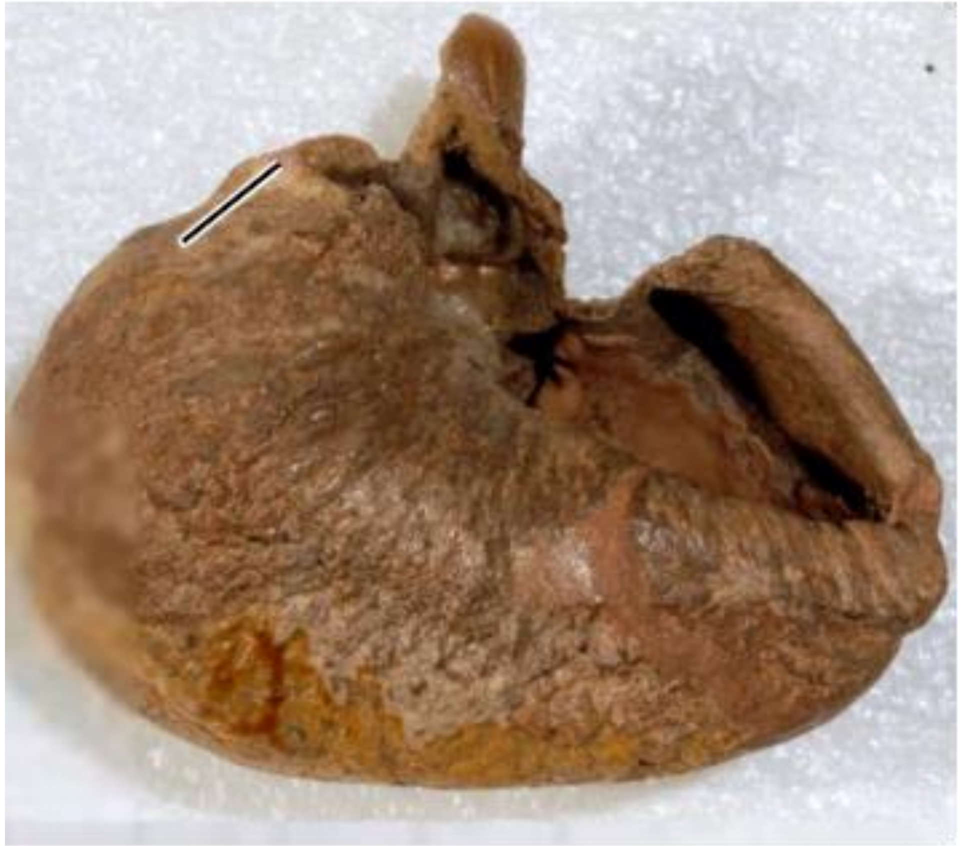

Pelocetus calvertensis (mirrored)

Copyright holder: Felix G. Marx/ United States National Museum
of Natural History, Washington DC, USA

[243] 'Vertebrarterial foramen in axis'

(0)'absent'

(1)'present'

(0)

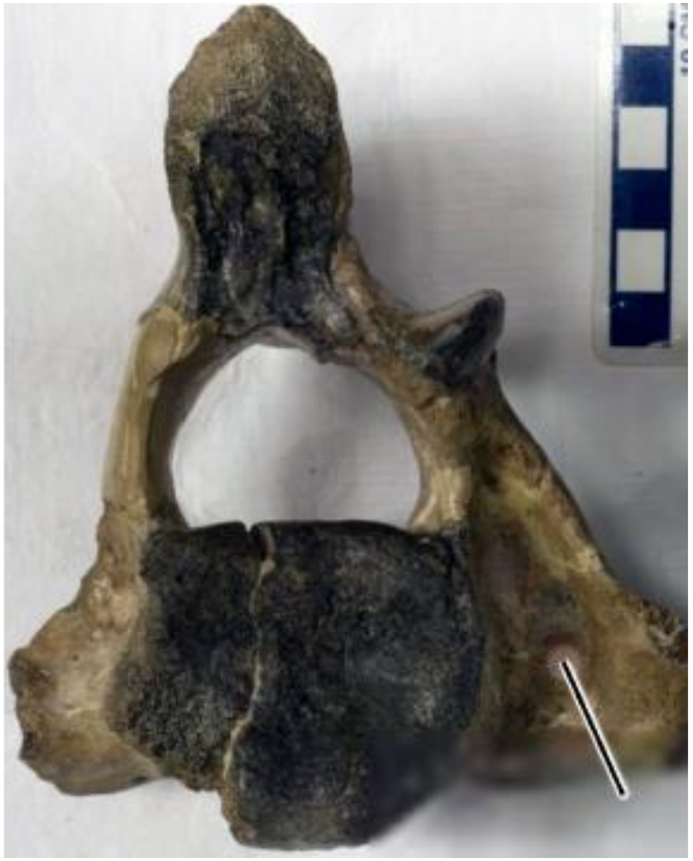

Zygorhiza kochii

Copyright holder: Felix G. Marx/ United States National Museum of Natural History, Washington DC, USA

(1)

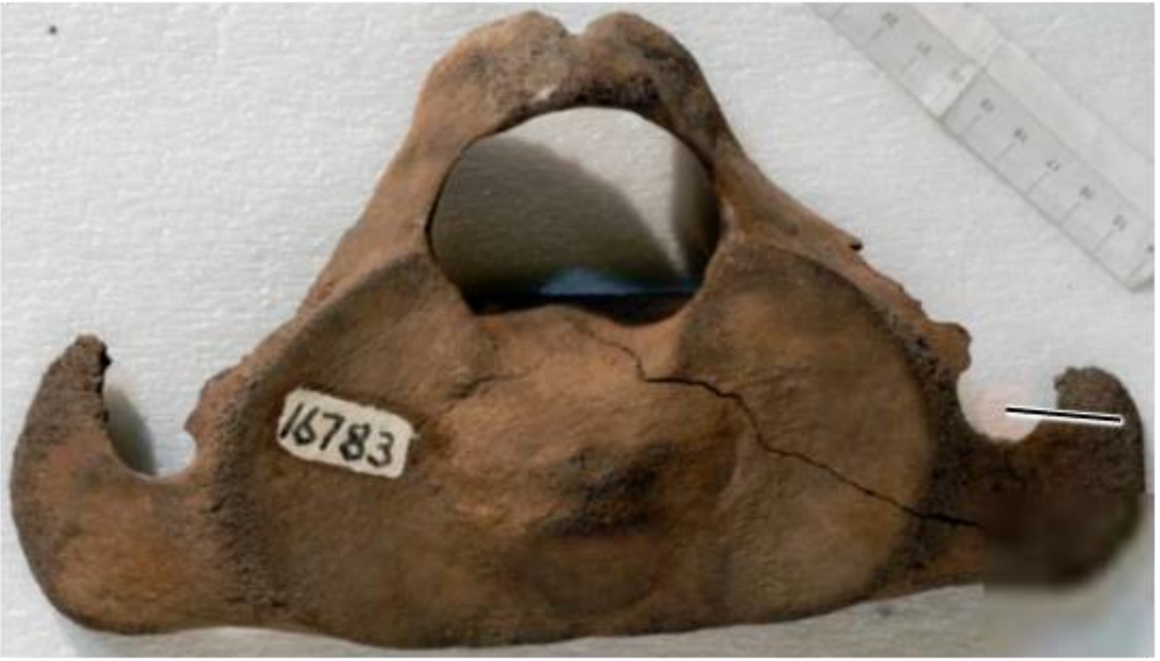

Diorocetus hiatus

Copyright holder: Felix G. Marx/ United States National Museum of Natural History, Washington DC, USA

[244] 'Development of parapophysis and diapophysis on axis in anterior or posterior view'

(0)'parapophysis considerably more robust than diapophysis'

(1)'parapophysis and diapophysis are similar in size'

(0)

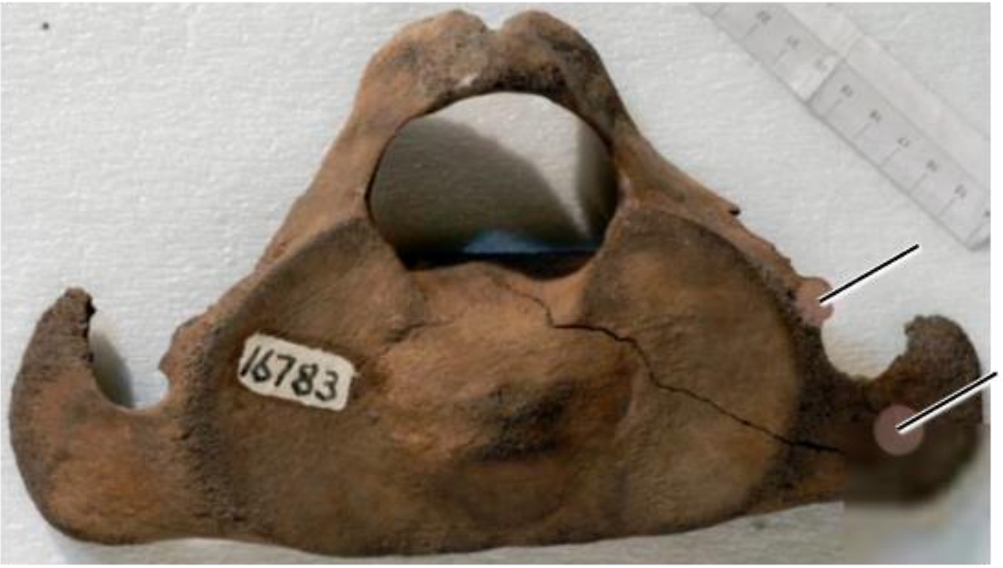

(1)

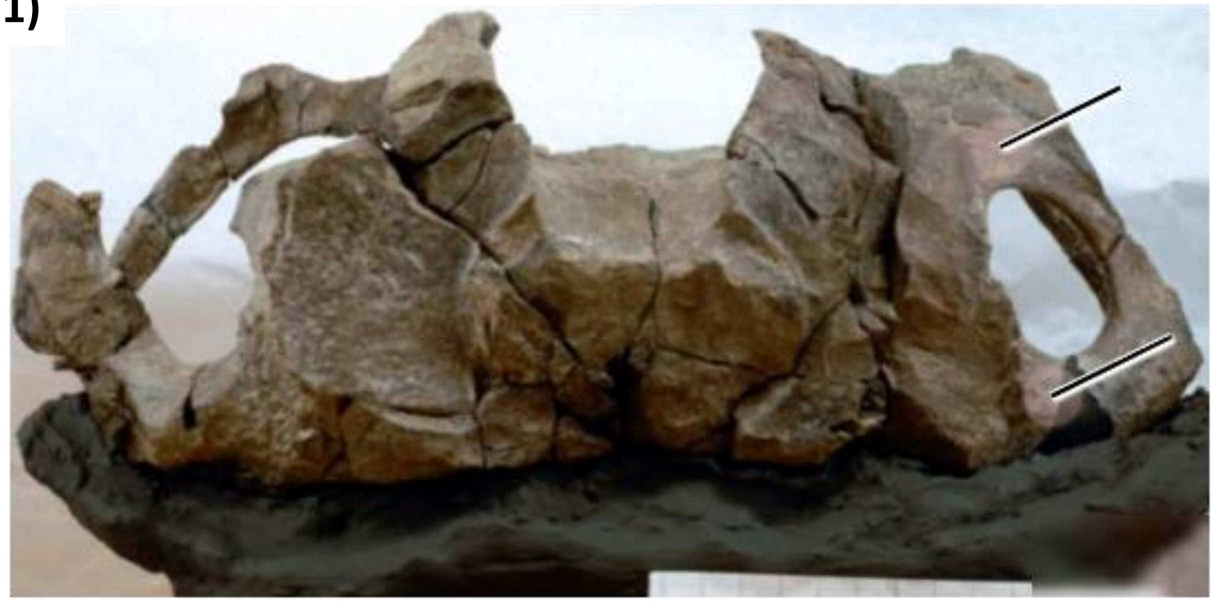

Diorocetus hiatus

Copyright holder: Felix G. Marx/ United States National Museum of Natural History, Washington DC, USA

Uranocetus gramensis

Copyright holder: Felix G. Marx/ Museum Sønderjylland, Gram, Denmark

[245] 'Cervical vertebrae'

(0) 'separate'

(1) 'partially fused starting from the axis'

(2) 'completely fused'

(0)

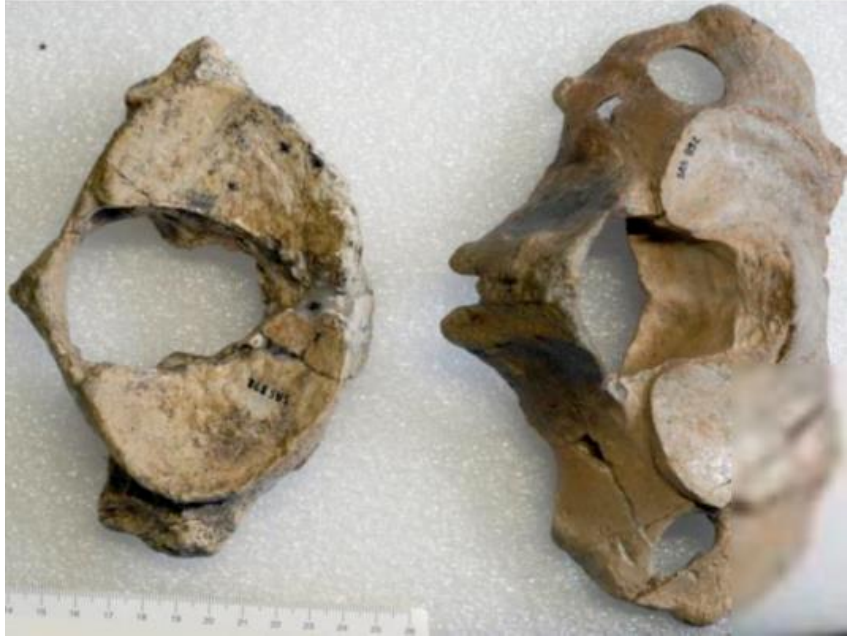

Piscobalaena nana

Copyright holder: Felix G. Marx/Museum National d'Histoire Naturelle, Paris, France

(1)

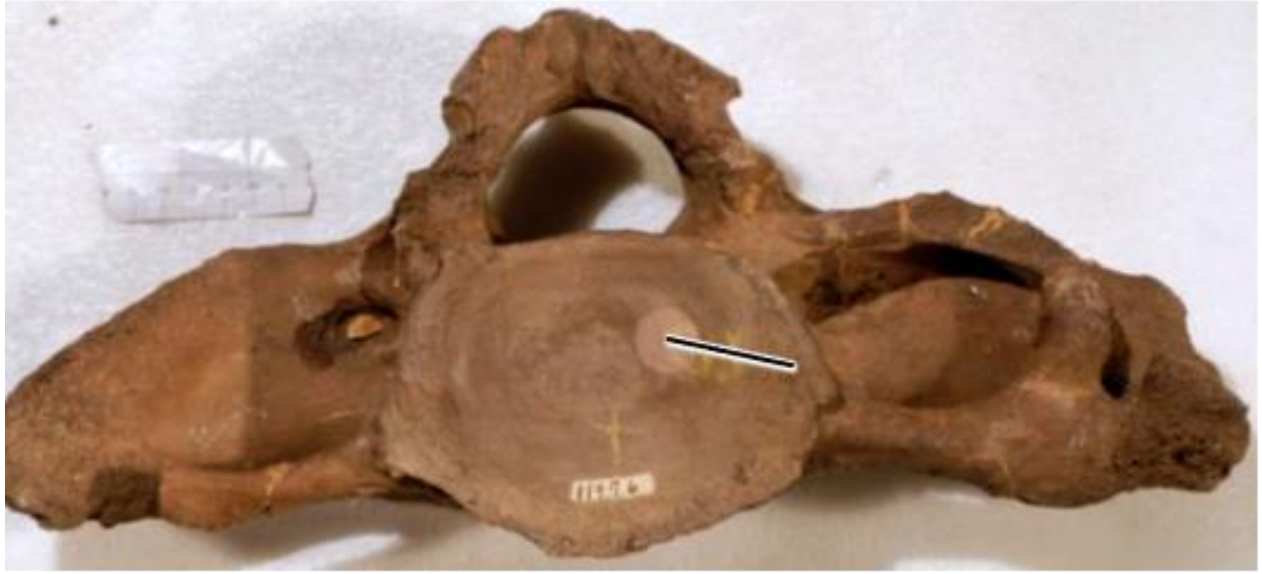

Pelocetus calvertensis

Copyright holder: Felix G. Marx/ United States National Museum of Natural History, Washington DC, USA

[245] 'Cervical vertebrae'

(0) 'separate'

(1) 'partially fused starting from the axis'

(2) 'completely fused'

(2)

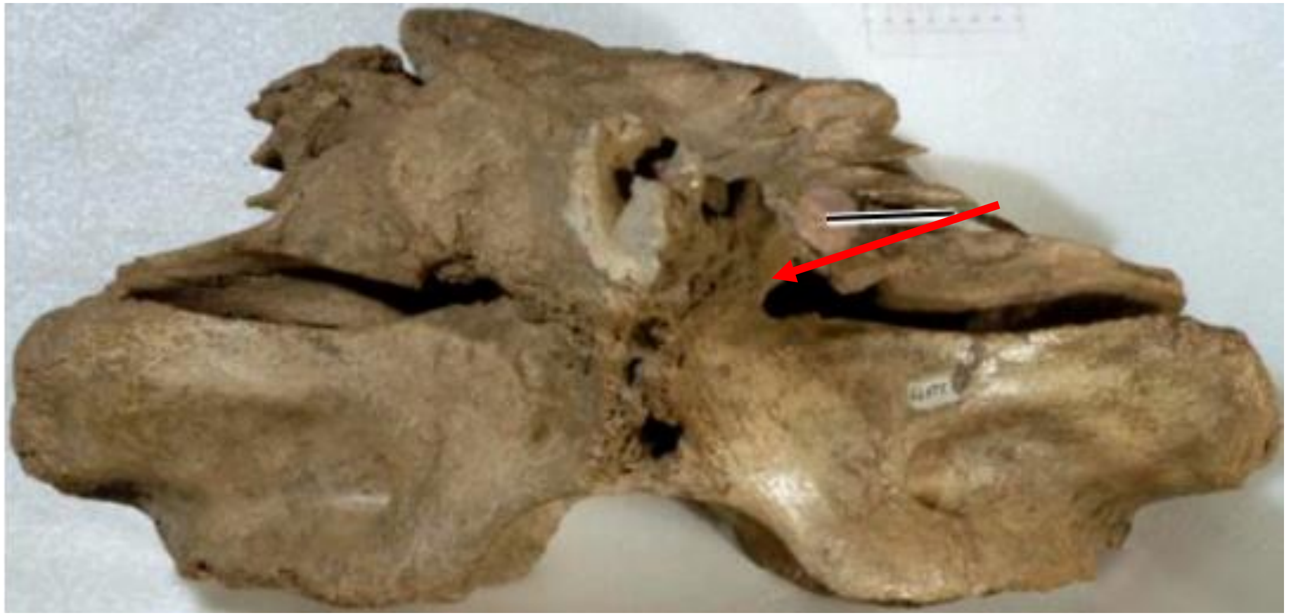

Balaena ricei (ventral view)

Copyright holder: Felix G. Marx/ United States National Museum
of Natural History, Washington DC, USA

[247] 'Centra of cervical vertebrae in anterior or posterior view'

(0) 'rounded'

(1) 'squared'

(0)

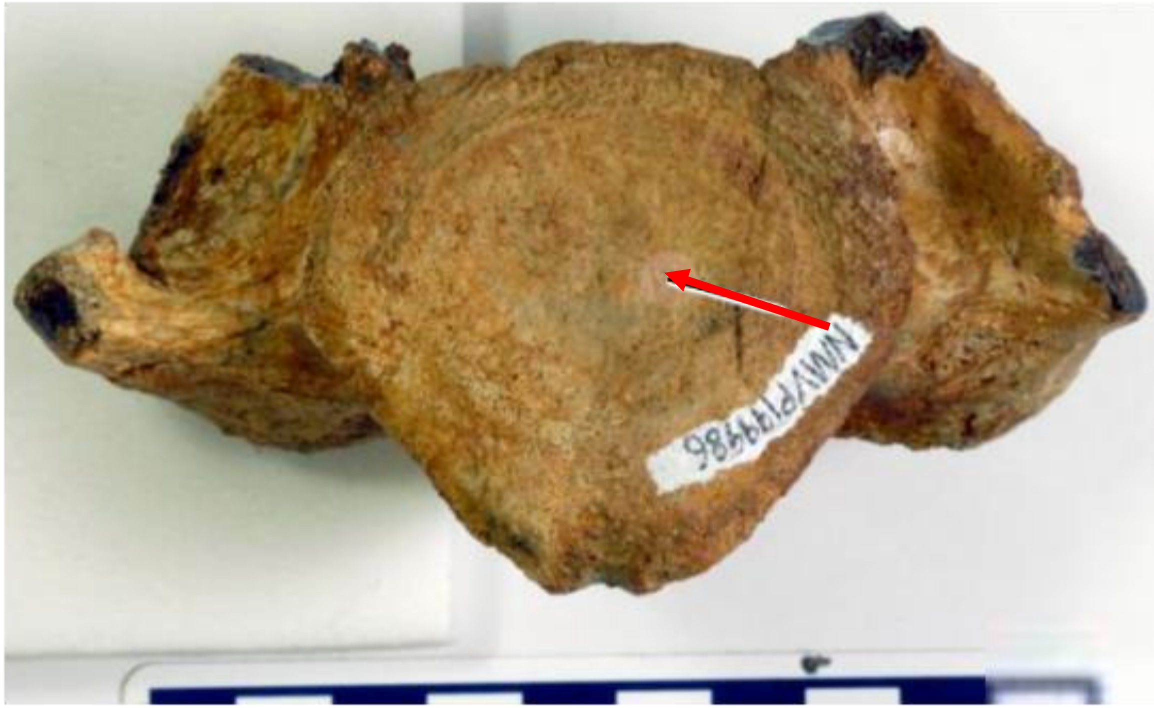

Mammalodon colliveri

Copyright holder: Felix G. Marx/ Museum Victoria, Melbourne, Australia

(1)

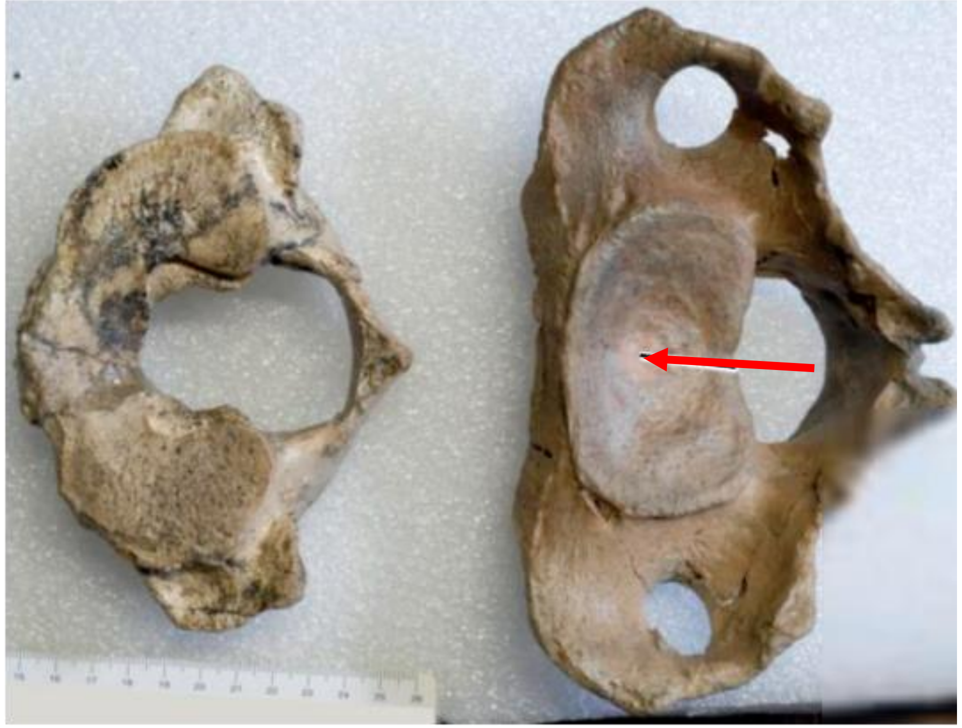

Piscobalaena nana

Copyright holder: Felix G. Marx/Museum National d'Histoire Naturelle, Paris, France
