# Supplementary material for: Redescription of three fossil baleen whale skulls from the Miocene of Portugal reveals new cetotheriid phylogenetic insights
Source: PLoS One. 2024 Mar 13;19(3):e0298658. doi: 10.1371/journal.pone.0298658 (PMC10936793; doi:10.1371/journal.pone.0298658)
Supplement: S2 File — (PDF) [file pone.0298658.s002.pdf]

Supporting information

Data matrix adapted from Duboys et al., 2020.

Zygorhiza kochii

00000000000000000000000000000000000000000000000000000000000000000100000000--000000000-000000000001000000000000000000100000000??0000?00000000?000-000000000000000000?000000000000000000000000000000000000?000000???0000?00000?000000000-000000000000-000000000000000000-0000000???????

Albertocetus meffordorum

?00???00?020000???110?0?0?1?????01???0?--100??0020200000--?0111--0000000100--010000000-01100?0001020000000000000100010000100??0000000000??????00-0000001011000020200010000000001000000?000001000000000000000001010??0000?00?????0??000?0-0??0???00?00-0?0000?0??0????????????????????????

Archaeodelphis patrius

?0????00??200?????100?0????????????????0--0000000002??000?-101?1--0000000100--0100????0-0?10?????????000?0000???????0??000100001000?00000??????00-000000?011000021200010????????????????00001000??

Olympicetus avitus

?00?1?00??20000?00000?0?0?001000?000?00--00000000000?0000-10111--0000000000--010000100-00100?01010200001000000000100?100000000??000?000?00???20?0000000000100???0000??0????????00??0-0?????????????0?0????????????????????????????????

Waipatia maerewhenua

10002000102000000000100000111100001 01?11--1110300201?0000--10111--0000000000--
110000--1-1010000001010200100000000100010000000???001?00000???????0-
0010100000000020200050100000010000 0?00??0???0300000000000?0110???1000000000
0000000000-00000000000???????????00000001000000??????????

Physeter macrocephalus

1-0020--1120000020210--00---------02110--010030220000010--10111---0000---10--1100----
1-11100000010100101110000001020--100000000--?21000000210000-
11000100000000??2310501000010200000?0001000103000000020010110???100000-
000000(0,1)0??000-000010000000-1-?1002000000010001011100(0,1)1000?000

Aetiocetus cotylalveus

0011100100100100010000000110101121020011000001000010100100000000000010100?0
100010000100-
011000001012100001000200001001000 0301?0000?001000???????????????????????????
?????????????????????00?000?00100000?0?0??00????0000???????????????????????????
0-01010011000???????????????????????

Aetiocetus polydentatus

101110010010010??????000011010112102?0110000010000101001000000000?0010000?0
100010000110-
011000001?1210000100000000200100 00301???00?001000000100????????????????????
????????????????????????0??00100000?0?0??10????000?00010103??0000?2-
00?????00000-010?????0001??000??00000??????????

Aetiocetus weltoni

0011100100100100010000000011010??2102?0110000010000101001000000000000100?0?0
100010000?00-0?10?0001012100001000??????001000030000000000001000??????0?-
0?0010?0?0000001??0?00???????1???????0001000?3010000000000001000??00000001010
3000000?2-00?01??0000????????????????????????????????????

Aglaocetus moreni

101121101111011121???0101-----------
?00110101100000??00100000000000000000101101111000000020001001101000000000003
01001103000??????0???01011??????00-
???????????????????0????????????????????020??????????????????????????-
111030000?01011011111100010???????????????????????????????????

Aglaocetus patulus

1011201011110111111??00101-----------?00110101000000??00100?0000000000100101--
111100000?02100?0011010000000?01301002103000?00???001101011??????00-
1010101221012120200000111010021000110100000000020?0011(0,1)00010111(0,1)100010
0????????????????????????????????0?110??0??????????????????????????

Antwerpibalaena liberatlas

?????????????????????????1-----------
??0??????????????00???????1
??????????????1???????00?0100-
001000122311110020002010100100000100010012001110011101001112-11101100-
1010310???0?1?????1??0????111111?????110?001110111100??????????

Archaebalaenoptera castriarquati

1011101011111111?2????0101-----------?00001211300200????1?100000100000100011--
1111----
0112?00??00101?000001102201122 10?00??????????????1????????????????????????????
???????????????????????010?????????????????????????-
1?11300????????????????00??????????????????????????????????????

Balaena montalionis

?1????????110?????????1?1-----------???0??1?????-10??????0?0??00-0-00010011001101---
-
1003101?0??2????1100100000010?11?001??10111?1??1010?????????????????????1????
???????????????????????000?0????????????????????????-
???

Balaena mysticetus

2111010011110111111000101-----------100100102202-10111011000000(0,1)-0-
00010011001101----100310000002-
0011100100000010212110111?01110?2110110001(0,1)110-
0010101223111120(1,2)0012010100100100010010000000110111100001112-11101100-
101031010000100—1111003101112111000011000001001121000000000010

Balaena ricei

?111??????????1??1?????01-----------????????????-1????????????????-??????1???11?1----
1?03100?0???????1100?00000010????????????????????10???????0-

??101012231111202000200010000010000?0??????????????????????????????-
1?1?310???????????????????11121110???11?0001100112100??????????

Balaenella brachyrhynus

00110100?111011?111000101-----------?00000102202-10??00110000000-0-
0????011001101----100310100??2????1000100000010211100111??111??2?1010???????0-
0?1????223111?2?2000????????0????????1001010010???1111????12-01??1?00-
??

Balaenoptera acutorostrata

1011111011111111210000101-----------
000001201311100111101010002000001001011111111----
01121101001101110000111131110110400020010101101111111100101111101002220101
202311(0,2)010101002000011010002000201001110111012-01000110-10113001-
10013101111100000011011201101201001111011101111111101

Balaenoptera bertae

??????????????????????????????????????01012013?0?0????1???0???0???-??????01--
1111----
0013110?0011010100001112301002100000?01??1011??1111?????????????????????????
????????????????????100?210??
?????????????????????????????????????

Balaenoptera bonaerensis

10111110111111111211000101-----------

000001201311100111110101000200000100101111111----

001211010011011100001111311101104000200101011011111111011011101010022220101

0023110010101002010001?01000200020100111011101110100110-10113001-

1001310111110000001101120110120100111101110111?11??10?

Balaenoptera borealis

10111110111111111210010101-----------

000001211311100111110100000200000100101111111----

01121101001101110010111131110110400020110101100111111101101111101102220101

00231100111011020100110100020(0,1)020?001110111012-01000111-10113001-

10013101111100000010011201001201001111011101111111101

Balaenoptera musculus

10121010111111111210000101-----------

000001211311200111110100000200000110101111111----

01111001001101110010111131110110400010110101101111110101101011101102220101

2020012011101102000001?01000201010?001111101012-11100111-10113001-

100131011111000000100(0,1)120100120100111110111011?2111101

Balaenoptera omurai

10111201011111111210000101-----------

000001211311200111110100000200100110001111111----

01121101101101110010111131110210000020010101100111111101101111101?02220101

2023010011101102010001?01000201010100111010101012-01000111-

101130010100131011111000000110112011012010011101011101?21??1??

Balaenoptera physalus

1011111011111111210000101-----------
000001211311200111110100000200000111101111111----
0(0,1)121001001101110010111131110110400020110101101111111101101011101102220
(0,1)01002011201110110201021?010002000201001110111012-01000111-10113001-
100131011111000000100112010012010011111011101112111101

Balaenoptera portisi

101111?01111?11??1???0101-----------
?00001211200?00???110?0?000??????????011111?100000002110?001101000010110130
1102112100??0???011??1010???????1011100002220001202001201110110200001?0???0
010?201??1110?????2-01??0??1-
1011310101001?1111111000100100?120??????1001??????????????????

Balaenoptera ryani

??2???????????????????0??000????1?????11
1----011(2,3)??010?????????0???1?130100???????200?010???01011???????0-
111010022000012020?000111001020102010????????1????111??????1?1???01??????????
???

Balaenoptera siberi

10122010111111112?0000101-----------
00000121131020011111?10(0,1)00020000010(0,1)001111111----
0112100?0011011000101101111001104000?0?????1??????1??????????????????????????
??????????????????????????10?20?001110???012-01??0111-
1?11300??????3?????????00??110??20110??010011101011001?????????

Balaenula astensis

10???100111101????1000101-----------?00000102202-10??001??000000-0-

00010011001101----

1003100?0002000010001000000102121001 1??0????12?1010??????0????10??1?????11??

?????????????10?????01001010010?011111???112-11001100-

1???3?01?????0????111003101????????????????????????????????????

***Balaenula* sp.**

????????????0????????????1-----------??0000102200-1???10???0???0???-??????110011?1---

-1003100?0002001?1000100000010????????????????????0??????00-

2010101223111120200020101000010001 0?01001010011?11111100???1101??1100-

101?310???????????????????1???????????11????????????????????????

Brandtocetus chongulek

??????????????????????????????????????0?10?101???0????00???????0?????0100?011?11

10----0-2?00000?0?????00000000301022112000?01??100???1011???????0-

211?1?12?400212(0,1)20003100?0010200001?11110020111?10110000101101?110100???

???

Caperea marginata

001111101111011121(0,1)010101-----------000000111000-101110101000001-0-

00021111021111----010301100-01--1-

0(0,1)110100210121121111 2021312?121111100000102020101 0(0,1)22401010-

100050(0,1)0100002010(0,1)11110003201101101100001112-1111(0,1)101-10103111-

1001111111110011011?2-020021111100111110110011 1000000?

Cephalotropis coronatus

2011101011110111?10010101-----------
?00100100000000??00100000000?01001001111?011000000-
02110000110100001101003010121101002 01?0100?101011??????????????????????????
?????????????????1?1110320?10100110?0?10???????010?-
???

"Cetotherium" megalophysum

?01?????????0????1200?2?1-----------?0?1??101???000??0010??1??0100100100-0???1110-
---0-
1110000011010000010010(0,1)101012110000101?01001101010????????????????2200?010
??????????????????????1100010?1011011??0?10110????010?-
???

Cetotherium rathkii

?0?0??????1101??21010?101-----------?00100101200200??0011011??011?0001?0-
01101110----0-21100?0001010000000?00101022112100201?010011010?1???????0-
2?1????2?400012120003????????20??????1?0002011110011??0?10?1?????010?-
1???3?0??

Cetotherium riabinini

201010001111011?210100101-----------
100100101100000?10010001000100000100101101111----0-
211000000101000000000010102211200020 1??100?1?1011?00001?????????????????????

?????????????????????10020?1111011??0010?1?????110?-1010304101000111-1121011100???1?1102111100011111011 00??????????

Chonecetus sookensis

??0??????????????????????????????010?010000100-0110000001120000000000000100010000??00??000??1000??????0-000000?0?000000020?0?0??????0????????0??0???0??

Ciuciulea davidi

?0?????0?1????1?2?0?1?2??????????????0????1????????????????100000?0?010???????11?0--00?1?1?0?0???????00?????010102???????201???00???10?????????0-?0111??22000?1??20?03????????????????????????2011011000011?101???0100????????????????????????????????1??0??1???????1?110??????????????

Coronodon havensteini

0001100010100000?001?000010000110001?0000000100200???00??0000000?0010002100--000000100-0000000001????000000000010{1,2}0010000??0???000??1001???????0-000000102000000100?000010000010102 0?000?0100010?000000??0000001?000000000000000000?0-00?????000010011???????0???????????????????????

Diorocetus chichibuensis

101120101111011121?100101-----------?0011010110000 0??00100000000-00000000011011110000000210010011010000000010030100????0??20100100??010?0????

???0-??101??22101210020???0??????0???????????0????20?10111110111100???0101-

1110300011010110111111000?0??0?1????????????????????????????????

Diorocetus hiatus

1011201011110111110000101-----------000100101000000??00100000000-

000?1??101??11110000000002100?0011010000000001301012113000?00???0011010?1????

??00-0010101221000120022?0301110100200011?010110?0120?0011001010110010?0100-

101030000?0112101112111000010?1110?11???1001????????0??????????

Diorocetus shobarensis

1011201011??0????1??00101------------0????1??????0?????????00?00-

000010010110111100000002100?0011??0?00?00?0010100210?000?01???0011?1010????

?????????????????(1,2)???????????????????????????0010?2010011??2?1??11????010?-

111?30010?0?0?10111??1????0?????20?????01001??????????????????

Diunatans luctoretemergo

????????????????????0????????????????0????2??????????????????02?000111?0???11

11----

0?1(2,3)100?001101?100101100?11101110000??01??0110?11111????010111010022201

01102101200?101?1201100?0100100012010?111010?012?0??????0??????????????????

???????????0?1??????????????????????????????

Eomysticetus whitmorei

20112?00?????????????0001-----------

?0???00?????00?0????????0000??011000?0100000000200-

01100??101131200000001201012010000??0???000??1000??????00-

0001001020000000000?0?000000001010000000000000000?000000000001010??0000-

1010200000000?0-10?????1??00-00000?????0?0??001110000?????????

Eschrichtioides gastaldii

?0??2?00??1?0?1?210???1?1------------

00101201???0001?111000100010?0??11??111311?1----0-

??110?00?1????000?1?022011221??000201?010111?1011???????10??1?1??2????????2??

???????????????????0000???20100110???1012-0???0100?10113131-

11013111111002100100?1???????0????11110???0??????????

Eschrichtius robustus

1011110011110111210000101-----------

00000(0,1)2012000001111100000000000010111111131101----0-

0111010011010000000102201112112100201101001101011100(0,1)110101110100220000

120230140111011120000010100000011(0,2)01001110101012-11(0,1)00100-

10113111011013111111100210110001201(0,1)012010011101011101011001001

Eubalaena ianitrix

??????????????????100?????????????????0000102202-1????0???0???0???-

??????110011?1----

10?310?00002000?11001000000010212100111??11110?11010?????????????????????????

??
?????????????????????????????????????

Eubalaena shinshuensis

101111011111011?1110?0101-----------?0??????????-????????0?00000-?-?0010?1???11?1--
--
1??3101??????????1?0100000010?????????????????????1????????????????????????????
???-
??

Eubalaena spp.

1011010011110111111000101-----------100(0,1)00102202-101110110000000-0-
00010(0,1)11001101----
100310(0,1)0000200011100100(0,1)0001021210011110111112110110(0,1)011110-
201010-22311112020000000 1(0,1)000-10000001001000010011111000 1012-11101100-
101031010000100-1111100310111211100101100101100112111000001 0010

Fragilicetus velponi

??02?1????0???01???????????(0,1)??????01??
11?1----
0?12100100???1??0??011003?100?1??000??0???0?1??10?1??????010001010022200010 0
21012011?0111201001??1000200???
??

Fucaia buelli

?0?????????00???????????0??001112?01?01100000100001??00???10??000000?0?000100
010000110-0?100??????????0?000?230101??????????????????1000000100?0-
0000001020000000120005010000000100020?00??0100000?000000000001000??0000??????
?????????????????????0-01020??0?????01??????????????????

Fucaia goedertorum

00111?01?0100000??000000011??????10??01100000100001??0010010000000010000001
00010000110-00100000110200000000023011100100003?00??000?01000???????0-
00000?10200000?02?0050??????0????????0??010?0?0?000000??000100???00000011010
300?0?0?2-?0?0???0??00-0?020????0?000?00100???0??????????

Gricetoides aurorae

???????????????????0??11?
????????????????????????0??0??????111?0?02001??011101011??????010111?1?02200?0
10023?1?01110111211000??1000011120?0?1110??1112-
01100?00???

Herentalia nigra

????????????0????????????????????????0????1????????????????1??010-20?1?0-
?????11?----0?21100?0????????0???10020102??????0??01??000??1011??????00-
??1010?221000121200030????????????????1000101????????????????????????????????
??

Herpetocetus bramblei

?0?????0??1101???1??1?2?1-----------??01?1101200?0?????1?001???10?200121-
11001110----??0?100010110210001000??????1121100??0???001??1011??????00-
20111112240101201?0?0301010000211021?111103301????????????????????????-
101030410000111111121111100????????????????????????????????????

Herpetocetus morrowi

1011201011110111?210010201-----------?01111101200000??001000100010-200121-
110(0,2)11?0----0-?1100010110210001000003010111211002 00?210012?1011??????00-
1011111224010120100030001000021102111111033011000011(0,1)00011110111?0100-
101030410000111111121111101101?110??1???????1??11??????????????

***Herpetocetus* sp.**

?0????????110????????????????????????0?10010????0?0??????0?1??011?2??1??-
?100?1?0----0-?110001????????0??0????0101????????????100???1011???????0-
??11????24010?2?10?030??????0????????1??03?01??????????????????????????????????
??

Herpetocetus transatlanticus

???1??????????00???????01??2?????-
1???11?0----0-0110?0101102000?10000??????1121100??0???0012?1011??????00-
2011111224010120100030001000021102101111033011 0?10110000111101111010 0?????
???

Horopeta umarere

?01?????????????????????1-----------
??????1????????????????????0???0???????1???10?00000?0?1????????????????00130100?

?????0???????????10?0???????0-

0010101120000120200?00001?0000101020?00000000000?0010100000010001000000-

???03??0?00?0?1?1??????????10011??0??100?001?????0100??????????

Incakujira anillodefuego

1011201011111111?11000101-----------

00000(0,1)2113011001111101010000000000011001111111----

0112110100110111001011013110221041002 0?101001101110???????1???1????22201?10

?2?1100??????0???????????0210?201001110101012-0?1?0111-

10113001??0?13??111110000??110??201(1,2)1??01001111011001?????????

Isanacetus laticephalus

10112010111101112100000101-----------?001101011000000??00100000000-0000100101--

1111000000021001001101010000000000?01002110000200101001101O1(0,1)??????00-

1010100221012120200000111010020001 0?0100001002011011110011111O1??0101-

1?1????????????????????????1001120?????0???????????????????????

Janjucetus hunderi

00112000001000010000011100111011100 1?012-

000010000101??1001000000011000?0100--000000100-

00100?00010200001000000000010010?0?1000??000001000?????0?0-

00??00??200000?12??050??????0????????0000000?000?00000????000????000?1000000

20000?0?0-000???00??0????????????????????????????????????

Joumocetus shimizui

10112010111101112100?0101-----------?0?11?101???000??00100010000-0000100-
011?111100?00-01100??????????0?????010101?????????????????????????????0-
??101????00????????0?0?0??010????????1??0010?20?1?1100??1?1100???010?-
1?1?300???????????????????0100?1??????????????????????????????

Kurdalagonus mchedlidzei

?0101?00??1101????????201-----------
?00100101100?00??00??00100010?00?????01101111----0-
21100?0001010000000100301022112000??????0011?1011???????0-
21111?12240000212000?1??????02000?1???1100201????????????????????????????????
???????????????????????11??1???0001??????????????????

Llanocetus denticrenatus

001???0??01??1???1000???001101000000?0???00?101?00?0?001???10000?01010?0000--
010011000-01000?000?0200000000000301010010000?001??000??1001000?0000-
00000??01??0??000?0050?0??00000??????0000000?00?000000000000000010000000??0??
?00?000?0-000010?00?0??0?0?????000???????????????????????

Mammalodon colliveri

00122000?11?000??000?1010111?01?1011?012-
1000200000??00100100000001100000100--000000100-
00100?0????????0?0000000001??????????0????0??10?0?00??0?0-
00000010200000012 0?0500000000201001 0?????????1000000000???01010??0?00100?01
02000000?0-00??1??00000-0?0?????00???????????????????????

Mammalodon hakataramea

????????????????????????0???????1?1(0,1)???
????0??00??01?0??????????0?????000001??
??????????????????????????????????10?000000000?01010??00?0?????????????????????
???

Matapanui waihao

2?1??????1??????????????1-----------
?????00???????????????????0???01?????01000000??2?0-
0110???????????00000???????10??000????????0??1?00??????00-
200110101000000010000000000000100020?00000000030?00000000000100???0000-
?010?0?00?0?0?0-?????0????00-010?????????001??????????????????

Mauicetus parki

10112110111??11121???0101------------
00???10?10000?0?????00?000??00000??10???111100000?02100??01101000?00?0003010
01103000?00???000??10?1??????00-
101(0,1)101120000100100050000000110001?01000200000?001110000001000000100-
11103000000?1?1011?11??10?0100?1??????????????????????????????

Megaptera hubachi

101???1011?11?112100?0101-----------000001211200100??00101010001000001111111--
1111----
0012100100010100000110?311?01114000??00??011??1011?00001?11111?0??22???011

02?0100??????120??????1000210?????????????????????????-
10?13??1010013101111100000011011 20?1112010011111011001?????????

Megaptera miocaena

??1?2?????11?11??1????1?1-----------??01002010?0-00??0010000??00-0-00000?01111111-
---
01131001000101000000110131010110400 02?01??011??1011??????0201110101220 00011
023?000111011010000100100 0210???1001100????1110???0101-
??

Megaptera novaeangliae

10112010111111111210000101-----------
0000012112(0,1)1200111110101000100000111001111111----
00121001011101101010110130112110400010110101100111111001101120101002221101
0023110011101002010011010002 0(0,1)12010011111110111011 00110-
10113001010013101111100000111011201101201111110101111101211110?

Metopocetus durinasus

??0?0100100-
0????11?----0?2110000???????00?0010??010??1??0?0??????001?????1??????10-
00101112200020012 0?03000110002011211012003301????????????????????????????????
???

Metopocetus hunteri

?0?????????????????0?1??????????????????????????????????????1??00?0100100-
0???111?----0?2?10000???????000001000010(1,2)2110000??11??0011?1011??????10-

??101??2200-
?0002?00?000??0?0?01??1??1200330?2011011000010111??110100????????????????????
??

Metopocetus vandelli

101??01011(1,2)101112?200?20?????????????0?10?10???000???001000100010?1?0100-
0100111?----0-
1110000???????0010000110100211?000201???001?01010??????0????1????2?????1?????
???????????????????100010???
??????????????????????????????????

Micromysticetus rothauseni

???0???00?
00?200??1100?01111302000000?0010?0010000??00??000??1100???????0-
00010010200000000?0500000000100020000??0???0????????????????????????????????
????????????????????0-0?0???????????????????????????????

Miocaperea pulchra

001111001?1101112???10101-----------?00000111200-10??101?1000001-0-00121101--
1111----010311110001011-
00?101002001211211110?021?1001201111??????02120101??22401?100120030??????0???
?????11003(2,3)01??????????0?????????????-
???

Morawanocetus yabukii

0?1?????????0?0001000??0011100101001??????00?20000?0?001??1???0???01?0?0?00--
010????00-
?1100?0001121000000?000001000100030??00??0001000?????????????????????????????
?????????????????????100?0010000??0?0??10????000?000?0000000?0002-
00?01??00000-000???????0?0??000000000??????????

Morenocetus parvus

?????????????????????????????????????01001010000????001?00???00?000????111001
101----1002100000020000100110001001021110001??00??1011?1010??????00-
10100??223011121200020??????0100000??10000100??????????????????????????????
???

Mystacodon selenensis

0010000000100000?0???1000011????0010?000000112000000?0010010000000101000000
--000011110-
01?00?00000200000?00?030101??
??0000?000???00??0-0?????????00-
0???????00?0000000000000?0????????

Nannocetus eremus

??????????????????????????????????????1?010?????????00???????0???1?????-
1???11??????????10???01102000?10000??????1121100??0???0012?1010???????0-
??1??00?24??01?????0??0010?00211021?11110330110110110???1111?0???0100???????
???

NMNS PV19540

?0112??0?1110?1??1????201-----------?00100101200000??0010001??011-200121-
11001110----0-
0110001001021 00?100000301012121100?00???0012?101?????????????1?1?1224010120??
?????????????1?????1?110330?1?0??1100????11011??0??0-
10?03?(0,4)1000?1111111211?110010111100?1??01001?????1100??????????

NMNZ MM001630

101111101111111210000101-----------?00001211311200??11101010001000001111 01--
1111----
0112110?00110111001 0111?3011011040002001010110011 11??????011111?1?022201012
02?012011??110200001??1000210?201001111101012-1???0111-
1?????????????3101?111000???????????????0????110111110??????????

OCPC 1178

???????????????????????0?????????????110000010000????0???1???0???0??????010000
0000100-01100?00101200000010001000200 10000????????0??1000???????0-
20000??0200000000200000???????????????0??0??????????????????????????????????????
???

OU 22224

?????0??1??????????????????????????????0???101??0?1????0???0???0???????????110011
000??0?-0010?????2???????0?000000102??1?00????????0??101????????0-
0000001021002020 10?0200000000000000?0????????00000100000100100 0??0001??????
????????????????????????0?1???????0?????0????00???????????

OU 22705

?01120???????????????????1-----------??0110101?0000???00???0???0???0??????01--11?10?000002100100110101000?000301001110000??00??0011?1010??????00-00101??221012120200000??????0200?0????000000?20?0011101011110???0100-11?0??00010?00101111100000????1???????0???????????????????????

OU GS10897

?0111?0??010?1????????000??101000?0(0,1)?011?00001?000???????0?0000?????100?1?0--00?01??00-00???????????????????00010???0????????0???

Parabalaenoptera baulinensis

1012201011?1011121???0101-----------?00001211200100??111??0100010000010110???1111----011210??00110100000011012010121040002?1???00?101111???????11111?1??2????0?202????????????????????1000210?2010011????????1????0?1?-10?13001??00131011110000000100?120???????????1111??????????????

Parietobalaena campiniana

1?11?0???111?11??101???01-----------??0110101100?0???001?00???0??????????01--11??????????10???01101020?001?0??????21100002000??0011010?00?????00-1010000221012100100030111?0000201021?010000?0020???111??0?011101??0?01-11?03?001?01011011111101?00??011??????????????????????????????

Parietobalaena palmeri

1011201011????11210100101-----------001??0101?0000???00???000000-
0000(0,1)001011?111100000002(0,1)00100110100000011003010121100002000??10011010
1(0,1)??????00-
1010100224012100(1,2)0003011100002010211010000100202101111001011101100101-
1110300011010110111(1,2)1100010100?1???1????????1??10??????????????

Parietobalaena **sp.**

1011201??111011??101?0?01-----------??0110101100000??0010001??00-000?????01--
11110000000002100??011010?00001100301002113000??1??10011?1010??????00-
?01?1??22401?100200030??????0????????1000010?20??011??0??????????010?-
11?0???01101011011121101000???????????????????????????????????

Parietobalaena yamaokai

1011201011????11210100101-----------00011010110000???00???0?0000-
000?????01111111???00002100100110100000001003010(0,1)211000020010100110101(0,
1)????????-
??1??0?2????????????0?0???????????????1000010?2(0,1)21011110?1011001??0101-
111030001101011001111100000??0?120?????0?001???????????????????

Pelocetus calvertensis

1012201011110111110000101-----------?001001011000001100100000000-
0000100?011011110000000020001?011010000000001301002103000201?01001101011100
00100-?01?10?22401?120200000??????0????????1000??0?201001111201011101100101-
10?03?0101001310111111000100-11120111??0?01011110110???????????

Peripolocetus vexillifer

??1???????
???????????????2????1?00?00???????1??00?????????12????????????00-
00100?02230?1120??00?????????1000??0?1000010011?111100??1?1110???0100???????
??

Pinocetus polonicus

?01??0??1111?11?21?100101-----------
0?01?0101200000??0010000??0??000?1????1101111??000001?????0110100??????01?01
01???????30??01????010??20
2101111??101110???0101-1?10300???????1?11?????????0-
0?1???????0100?1?110??0???????????

Piscobalaena nana

2011201011110111212000201-----------?00100101100200??001000(0,1)00010-200121-
01001110----0-2110000011010000100000101022110100201101001111011?0000100-
0010101221002101200030001111020112111110033011011011000010110111110100-
1010304101001111111210111011101110011??010011111011001????????

Plesiobalaenoptera quarantellii

1?112010111?011??????0101-----------?0?1??2??????00????1?1?100010???????????????1-
---??12???????????????????????1?10???????????????????????10011010111000-
221002110210100111010100110 1?01000200020?001110111012-0???0111-
101130010?0?131??11110000?0??0?120111120???????????????????????

Thinocetus arthritus

????????????????????????1-----------

?????????????????????????????????????0???????????????100?0????????0?0?00?????1?1

13000??????0011?1011??????00-

0010101221000120220030111010021101 1?1101001012011011001010110110001 00-

10103?0001111210111211100001 01?1101111?0100111010110 0??????????

Tiphyocetus temblorensis

??1???0???0??1???0???111

100000?02100?0011010?0?001?003010?2110000??1???00?101010?????????????????????

?????????????????????????100?010?2121011??0????1?????0101??????????????????????

??

Titanocetus sammarinensis

10111010?111011??1???0101-----------

?00100101?00000????100?10000001000001011011100000000100000011010000000001000

01002103?00??????00???1?11???????????????????????????????????0?10

?201??11?????????????010?-

111030000?001110111111?0000110?1??????????????????????????????

Tiucetus rosae

?01???10?1??0?1?210???10??????????????0????1?????000??00????0000000001??1011?

111100000102100?001101000000010030101111 0000?011?10011?1010?????00-

?0111??22100012?200050??????0?????????10001012011011100010110 01??0100???????
???

Tohoraata raekohao

??????????????????????????????????????100001????????00??0????0????1?????010000
0000200-011??????1???20???00??1010?10??000???????????1??0???????0-
200100101100002020?03000000001000?0?00??????030?00000000?001000??0000????0?
??000??03???001100000?????????????????????????????????????

Tokarahia kauaeroa

201?2000?1???????????0101-----------
?0?1?0001???00??????????000???0110?010100000000200-
0110???11103120??????010102????????????????????????????00-
200100101100002020003000000000100020?0000?0?0030?000000000001000000000-
??10200???????????????????????010??1??110110100?100??0??????????

Tranatocetus argillarius

?0?????????10?1?????1????????????????0????1???????0???????????011?10????-
01?0111?----0-21000?0011010100000??0101002110000???????0?1?1011?10001?0-
??101????00-
0???22?0?0???1??0????????????????10???11???????1?????0?0?????0???0?00?0???1????
??001?1?01110??1??010010?110??????????????

Tranatocetus maregermanicum

?0?????????????1???????(1,2)??????????????0????1?????2???????????0??1??1001??-
01??111?----0-2110000011????00000100101001110000??0???0011?1011???????0-
001011?2200-
012?2?????????????2??????11100330??01?011??0?10?????????0??10?0???000000111111
2111001????11??????????????????????????????

Uranocetus gramensis

10122010?111011??1???0101-----------
?00100101???000??001?0?00?000??001???01111111----
0?1200???01101??0?001?0?3010?21030002???????1101011???????0-
00101???210????020?1?01????00200001??10000?0?201001111?0101110???0101-
01?03?010?0003101111110000011111??????2??001111101100?????????

Waharoa ruwhenua

2011200?111???1??1???0101------------0010000111000???00???0?0000-
?0110?010100000000200-
01100?0111?31200100000101012010000???????????10?0??????00-
2(0,1)0100101100000(0,2)0200030000000010002?00000000030?00000000000100000000
0-1010200000000?0-100??0000?00-01000???110????00?1?0000??????????

Whakakai waipata

??100?10
?0000??01???????????????????010100?????????????????????????????0-

0000101020000000010?00011000000000000?00??????000?000000100000000??0000??????
??????????????????????100????????????0????????????????????

Yamatocetus canaliculatus

2011200011110111010110101-----------
?00??0001110000010010000000000011030?0100000000200-
0110000101131200000000001011010000200?0000?001000000101????????????????????
???????????????????00?000?000000000000?0001???0000-1010200000000030-
100110000?00-000???????00001001010000??????????

ZMT 67

?01???1011???????????0101------------
0??????????????????????00???00001?01??????1?00000?0?1?0????????????0?00?30100?
????????00??000??1011??????00-
0010100120002100200050000??000011001??1000200100?001110100001000000100-
11?030000?0?0?10?1????????010011?????????00100110???0??????????

Adicetus vandelli

101??01011(1,2)101112?2000201-----------?0?10?101??0000??001000100010?1?0100-
0100111?----
0?1?11010??(1,2)????0010000130100211(0,1,2)000201???001?01010?????????00101???2?
0??(0,2)1????1?(0,3,5)?????????????????1000101????????????????????????-
??

Adicetus latus

?01????????1?11?212000201-----------?0?1??100??(0,1)?001?0010001??01??10?1??-

01001110----

0?1(1,2)110100110110001000013010021100002 01???00?101011?????????????1??2?????

??????????????????????????0002011011011000?10?1?????0100-

???

Cephalotropis nectus

(1,2)011(1,2)010?111011?110010101-----------

?00100101000000???0100000000?00001011?110011000200-

0?11000???????001?0??03010?????????01???0???010?1??????????0?????2????????????

?????????????????110002012010011?????0???????010?-

???????????????????????????000?1???????????????????????????????
